# Evolution of naturally arising SARS-CoV-2 defective interfering particles

**DOI:** 10.1038/s42003-022-04058-5

**Published:** 2022-10-27

**Authors:** Samer Girgis, Zaikun Xu, Spyros Oikonomopoulos, Alla D. Fedorova, Egor P. Tchesnokov, Calvin J. Gordon, T. Martin Schmeing, Matthias Götte, Nahum Sonenberg, Pavel V. Baranov, Jiannis Ragoussis, Tom C. Hobman, Jerry Pelletier

**Affiliations:** 1 Department of Biochemistry, McGill University, Montreal, QC H3G 1Y6 Canada; 2 Department of Cell Biology, U Alberta, Edmonton, AB T6G 2H7 Canada; 3 McGill Genome Centre, Department of Human Genetics, McGill University, Montreal, QC Canada; 4 School of Biochemistry and Cell Biology, University College Cork, Cork, Ireland; 5 SFI Centre for Research Training in Genomics Data Science, University College Cork, Cork, Ireland; 6 Department of Medical Microbiology and Immunology, University of Alberta, Edmonton, AB T6G 2E1 Canada; 7 Rosalind and Morris Goodman Cancer Institute, Montreal, QC H3A 1A3 Canada; 8 Department of Bioengineering, McGill University, Montreal, QC Canada; 9 Li Ka Shing Institute of Virology, U Alberta, Edmonton, AB T6G 2E1 Canada; 10 Women & Children’s Health Research Institute, U Alberta, Edmonton, AB T6G 1C9 Canada; 11 Department of Oncology, McGill University, Montreal, QC H3A 1G5 Canada

**Keywords:** Molecular biology, Virology

## Abstract

Defective interfering (DI) particles arise during virus propagation, are conditional on parental virus for replication and packaging, and interfere with viral expansion. There is much interest in developing DIs as anti-viral agents. Here we characterize DI particles that arose following serial passaging of SARS-CoV-2 at high multiplicity of infection. The prominent DIs identified have lost ~84% of the SARS-CoV-2 genome and are capable of attenuating parental viral titers. Synthetic variants of the DI genomes also interfere with infection and can be used as conditional, gene delivery vehicles. In addition, the DI genomes encode an Nsp1-10 fusion protein capable of attenuating viral replication. These results identify naturally selected defective viral genomes that emerged and stably propagated in the presence of parental virus.

## Abstract (web summary)

Genomes from defective interfering (DI) particles following serial passaging of SARS-CoV-2 reveal a fusion protein that attenuates viral replication. Synthetic, recombinant DI genomes are designed to interfere with SARS-CoV-2 replication.

## Introduction

Betacoronaviruses are pathogens with significant medical and economic importance. In the last 20 years, they have been responsible for three major viral outbreaks: the 2003 SARS-CoV and 2012 MERS-CoV outbreaks, and the current SARS-CoV-2 pandemic. Coronaviruses are positive-sense, single-stranded RNA viruses whose genomes are 26–32 kb. The first two-thirds of the coronavirus genome consists of two large open reading frames (ORFs) encoding non-structural proteins that function in viral replication. The rest of the viral genome includes ORFs that are transcribed into subgenomic mRNAs through a process of discontinuous transcription that encodes structural and accessory proteins. The 5′ and 3′ untranslated regions (UTRs) contain structural features essential for replication^1^.

As noted with other positive-strand RNA viruses, coronaviruses show high rates of recombination. Replication-induced errors, coupled with recombination between coronavirus genomes, add to the genetic diversity of the viral pool, are responsible for the emergence of new viral variants^2^, and generate defective viral genomes (DVGs) harboring large deletions^3,4^. On occasion, DVGs that maintain the ability to replicate and be packaged in the presence of helper virus emerge, and these are known as defective interfering (DI) particles. Naturally selected DI genomes have been characterized for several coronavirus members, including murine hepatitis virus^5–14^, transmissible gastroenteritis virus^15,16^, bovine coronavirus^17–21^, and infectious bronchitis virus^22,23^. As well, synthetic DIs based on the human 229E and SARS-CoV-2 coronavirus genomes were recently designed and shown capable of reducing viral genomic RNA (gRNA) levels or viral titers in vitro and in vivo^24–26^; presumably a consequence of competition with the parental virus for limiting cellular and/or viral resources^27^. Characterization of naturally arising DIs is particularly insightful as these define the consequence of selective pressures imposed during the amplification of mutant genomes in the presence of the parental virus, providing insight into the genetic information required for stable co-propagation with the parental virus. Herein, we report on the isolation and functional characterization of SARS-CoV-2 DI particles.

## Results

### Isolation and characterization of SARS-CoV-2 DVGs

**Fig. 1:**
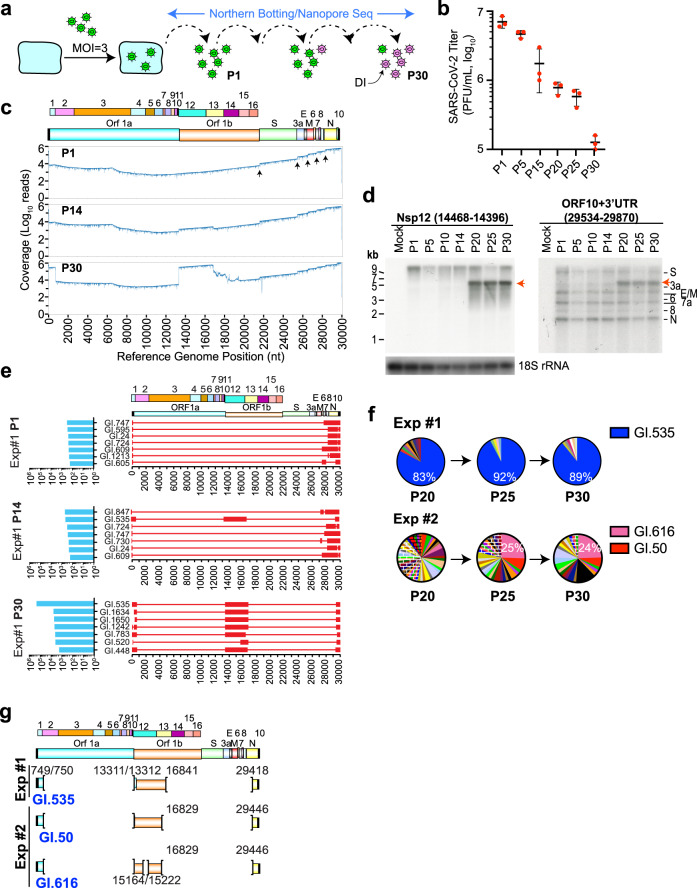
Emergence of SARS-CoV-2 DI particles upon serial passaging in Vero E6 cells. **a** Strategy used to isolate SARS-CoV-2 DIs. Northern blotting and nanopore DRS were used to assess the emergence of prominent DVGs during serial passaging. Parental virus particles are shown in green and emerging DIs in magenta. **b** Quantitation of virus titers obtained from the indicated passages. *n* = 3 biologically independent experiments ± SD. **c** Genome coverage of nanopore RNA sequencing data from P1, P14, and P30. The “step” changes (indicated by upward arrows in P1) occur at the 5′ borders of the S, 3a, E, 6, and N sgRNAs. The reference genome position (nt) is shown at the bottom. **d** Northern blot analysis performed on RNA isolated from the indicated passages of SARS-CoV-2-infected cells. Red arrow highlights prominent DVGs emerging at late passages. Mock, uninfected cells. Probe identity is indicated above the blots. **e** Architecture of the top seven most abundant DVGs obtained from P1, P14, and P30-infected cells from Exp #1 having retained 5′ and 3′ end sequences. Right: DVG architecture. Open red boxes are retained sequences and thin lines correspond to deletions. Left: Read counts corresponding to the transcript model. The SARS-CoV-2 reference genome is shown at the top, along with the encoded polypeptides. Nucleotide position of the reference genome is provided below. **f** Pie chart illustrating relative abundance of DVGs in P20, P25, and P30 from Exp #1 and Exp #2. **g** Genome architecture of the most prevalent DVGs isolated from infected cells at P30. Nucleotide position is based on the SARS-CoV-2 Wuhan-Hu-1 isolate (NC_045512.2).

To select for naturally emerging DIs, a stock of SARS-CoV-2 was serially passaged 30 times at 3 pfu/cell (Fig. 1a). Assessment of viral titers during passaging revealed a drop in infectious virus production; with P30 virus showing a 55-fold reduction compared to P1 (Fig. 1b). Direct RNA sequencing (DRS) of total RNA isolated from P1-, P14-, and P30-infected cells revealed that by P30, a significant proportion of DVGs that arose during serial passaging retained genomic segments spanning Nsp10–Nsp12 (~13.3–16.8 kb) (Fig. 1c). Northern blot analysis of RNA isolated from infected cells at various passages identified prominent ~5 kb DVGs from P20 to P30 that had retained Nsp12 and the ORF10/3′UTR regions (Fig. 1d). Transcript models constructed from the DRS information and retaining 5′ and 3′ end sequences (as these regions harbor essential replication signals^28^), indicated loss of a large proportion (>27 kb) of the SARS-CoV-2 genome in P1-infected cells (Fig. 1e). By P14, a ~4.7 kb DVG (GI.535) retaining nucleotides 13,311/13,312–16,841 had emerged. By P30, GI.535 and two related genomes, GI.1634 and GI.1650 (differing only in the 5′ end starting location), predominated the DVG population (Fig. 1e and Supplementary Data 1). To validate the presence of GI.535, a primer pair targeting the 5′ and 3′ ends (A1 and A2) (Supplementary Fig. 1a and Supplementary Data 2) were used in long-range (LR)-PCRs to amplify the DVGs (Supplementary Fig. 1b) and these were cloned and sequenced. GI.535 harbors: (i) an Nsp1–10 in-frame fusion, (ii) sequences spanning Nsp11, the frameshift signal, and Nsp12, and (iii) an out-of-frame fusion between Nsp13 and the last 116 nts of the N ORF (Supplementary Fig. 1a). We tracked the appearance of the upstream and downstream junctions (USJ and DSJ) identified in GI.535 and found that these appeared to co-emerge during serial passaging (Supplementary Fig. 1c). GI.535 was the dominant DVG (~83%) throughout P20–P30, indicating stable long-term propagation of this genome (Fig. 1f, Exp #1).

To determine whether structural features identified in GI.535 could be independently re-isolated, we repeated the serial passaging of SARS-CoV-2 at high MOI (Exp #2) from the same viral stock used in Exp #1. A 21-fold reduction in viral titers was noted by P30 in Exp #2 (Supplementary Fig. 2a). Tiling of the SARS-CoV-2 genome using Northern blotting with a series of probes (A-G) revealed the emergence of prominent DVGs of ~6 and 7 kb by passage 14 that had retained 5′ proximal sequences (detected by probe A), the region spanning Nsp12 (probe B), and 3′ end sequences (detected by probe G) (Supplementary Fig. 2b). By P20, we observed the emergence of ~5 kb DVGs that were stably maintained for 10 additional passages (Supplementary Fig. 2c). DRS revealed a pool of DVG structures at P15 that differed from those seen in the first experiment (compare Supplementary Fig. 3a, P15 to Fig. 1c, P14). In this experiment, the two most prominent DVGs at P15 were GI.464 (7.2 kb), and GI.384 (5.7 kb) (Supplementary Fig. 3b), and these corresponded in size to the genome species that we had detected by Northern blotting in P14 (Supplementary Fig. 2b, probes B and G, indicated by + and *, respectively). The prevalent DVGs from P29 of Exp #2 were found to be similar in structure to those identified in P30 of Exp #1 (compare Supplementary Fig. 3a (P29) to Fig. 1c (P30)). The top seven most abundant DVGs present in P29 all harbored the identical Nsp1–10 junctions that had been documented in GI.535 (Supplementary Fig. 3b). Amplification of the genomes present in P15 and P29 using LR-PCR, followed by Sanger sequencing confirmed the architectures of GI.464, GI.384, GI.616, and GI.50 (Supplementary Fig. 3 and Supplementary Data 1). A 19 amino acid in-frame deletion in Nsp12 is present in GI.616 and distinguishes it from GI.50 (Supplementary Fig. 4a). LR-PCR revealed the presence of ~7 kb genomes that emerged between P11 and P16 and ~5 kb genomes appearing later between P20 and P30 (Supplementary Fig. 4b). We assessed the abundance of GI.616 and GI.50 at P20, P25, and P30 and found GI.616 to be the major DVG (25%) present in P25 and P30 (Fig. 1f, Exp #2). In sum, the structure of the DVGs with the highest relative fitness from both these experiments (i) have retained 5′ and 3′ end sequences, (ii) harbor the identical Nsp1–10 junction breakpoint, and (iii) maintain Nsp11, the viral frameshift site, and the Nsp12 coding region (Fig. 1g).

### Replication of late passage SARS-CoV-2 DVGs is helper virus-dependent

**Fig. 2:**
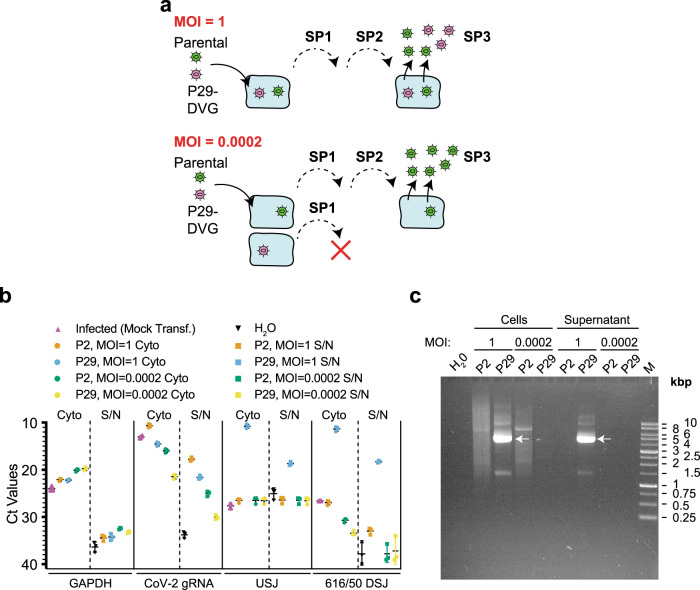
Replication of SARS-CoV-2 DVGs is helper virus-dependent. **a** Schematic diagram showing DVG-dependency on parental virus for replication and propagation. At an MOI = 1, both parental and DI genomes are expected to be maintained upon serial passage. At low MOI (0.0002) in which parental genomes and DIs enter different cells, the DI will be lost upon sequential serial passaging. **b** RT-qPCR analysis of RNA isolated from cells or supernatant infected with the indicated viral passages and MOI. *n* = 3 biologically independent experiments ± SD. **c** Isolation of DIs from Vero cell lysates and media that had been infected with the indicated viral stocks at an MOI of 1 or 0.0002. Amplifications were performed using A1 and A2 primers for 30 cycles. Products were analyzed on a 0.8% agarose/TAE gel. White arrows indicate recovery of 5 kb DVGs. M; 1 kb DNA ladder.

A defining feature of DIs is their dependence on parental virus for propagation. To determine if this was the case for the naturally selected DVGs, we serially infected Vero E6 cells with P2 or P29 stocks from Exp #2 at either an MOI of 1 or 0.0002. Genome contents of infected cells and media were then assessed following the third infection (Fig. 2a). At an MOI of 0.0002, DVGs from P29 are more likely to enter uninfected cells and, therefore, should be lost from the population upon subsequent passages. However, at an MOI of 1, DIs are more likely to enter virus-infected cells and should be maintained during passaging (Fig. 2a). RNA prepared from cells and media (S/N) of the third serial passage (SP3) was analyzed by RT-qPCR. GAPDH transcripts were detected only in cellular RNA preps (Fig. 2b). SARS-CoV-2 gRNA was present in both cells and media from cells exposed to P2 or P29 dilutions. The USJ and DSJ common to GI.616 and GI.50 were readily detected in RNA from media and cells infected with P29 (MOI = 1). In contrast, USJ or DSJ RT-qPCR products were not detected in RNA from cells infected with P29 (MOI = 0.0002) (Fig. 2b). Additionally, infection with the P2 stock showed that no DVGs containing the USJ or DSJ were present in RNA isolated from SP3 cell lysate or media (Fig. 2b). DVGs were recovered only from cells and media infected with the P29 (MOI = 1) stock (Fig. 2c). No DVGs were detected in extracts or media from cells infected at an MOI = 0.0002. Thus, the prominent DVGs are only propagated in the presence of helper virus, and their detection following serial passaging also indicates that they are packaged. Henceforth, we refer to these DVGs as SARS-CoV-2 DI particles.

### Synthetic, recombinant DI genomes exhibit long-term stability and attenuate SARS-CoV-2 replication

**Fig. 3:**
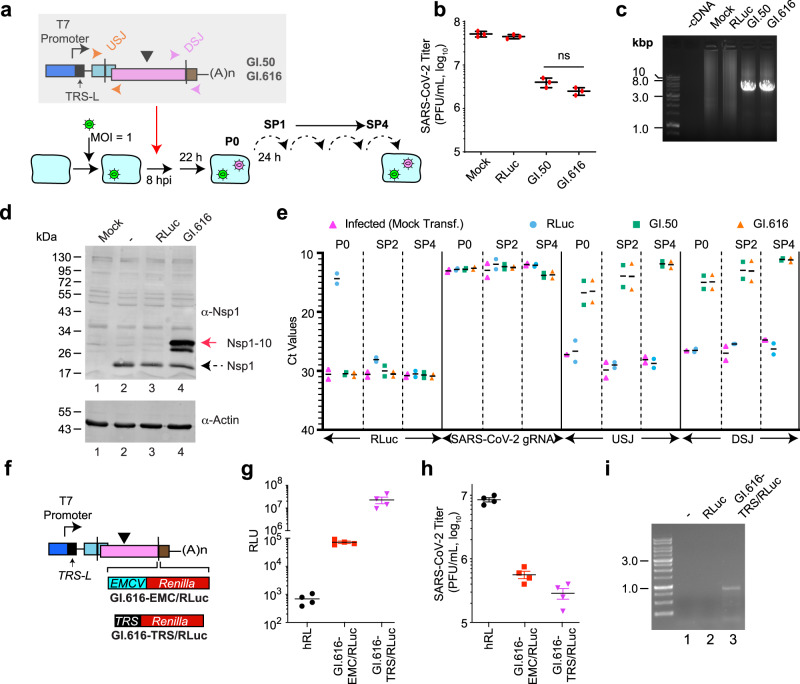
Characterization of synthetic DI genomes. **a** Experimental flow used to generate synthetic DI particles. Following infection of Vero E6 cells with SARS-CoV-2 at an MOI = 1, cells were transfected with in vitro synthesized RLuc, GI.50, or GI.616 RNA. Media was collected 22 h later, clarified, and used in serial infections (four passages) of Vero E6 cells. **b** Quantitation of virus titers obtained from the indicated DIs at SP4. *n* = 3 biologically independent experiments ± SD. ns nonsignificant—*p* > 0.9 (two-way ANOVA). **c** RT/LR-PCR showing recovery of DI genomes from SP4-infected cells. **d** Western blot of extracts probed with α-Nsp1 or α-actin antibodies. Lysates analyzed were prepared from uninfected (mock) Vero E6 cells (lane 1) or Vero cells receiving SP2 from untransfected cells (−) (lane 2), RLuc mRNA-transfected cells (lane 3), or GI.616 RNA-transfected cells (lane 4). Dotted arrow denotes Nsp1 and red arrow denotes Nsp1–10 fusion. **e** RT-qPCR analysis of RNA from P0, SP2-, and SP4-infected cells. RNAs targeted by each oligo pair is shown on the bottom. Obtained Ct values are displayed. *n* = 2 biologically independent experiments, black bar represents the mean. **f** Schematic diagram of DI genomes harboring EMCV/RLuc or TRS/RLuc expression cassettes. **g** RLuc activity obtained from the indicated constructs at SP4. *n* = 4 biologically independent experiments ± SD. **h** Quantitation of virus titers obtained from the indicated DIs at SP4. *n* = 4 biologically independent experiments ± SD. **i** RT-PCR showing the presence of an RLuc sgRNA containing sequences upstream of the 5′ TRS-L site in SARS-CoV-2-infected cells transfected with GI.616-TRS/RLuc.

To formally demonstrate that the DI genomes we had isolated were responsible for attenuating SARS-CoV-2 replication, we placed the sequences corresponding to GI.50 and GI.616 under control of the T7 RNA polymerase promoter and appended a 3′ poly(A) tail (Fig. 3a). Synthetic DI RNAs and RLuc mRNA (negative control) were generated in vitro. Eight hours following infection of Vero E6 cells with SARS-CoV-2, RLuc, GI.50, and GI.616 RNA were transfected into cells. Twenty-two hours later, the virus was collected and serially passaged four times (Fig. 3a). Plaque assays showed that viral titers were reduced by 10–20-fold in cells that received recombinant DI RNA following infection, whereas no reduction was apparent in cells having received RLuc mRNA (Fig. 3b). Full-length synthetic DI genomes were recovered by LR-PCR from RNA of SP4-infected cells (Fig. 3c). Probing cell lysates with α-Nsp1 antibodies revealed the presence of Nsp1 in virus-infected cells (Fig. 3d, compare lanes 2–4 to lane 1). Cells that received the virus from GI.616 transfected cells also expressed an immune-reactive protein whose molecular mass is consistent with it being an Nsp1–10 fusion product (Fig. 3c, compare lane 4 to 3). RNA from P0, SP2, and SP4 infections was analyzed by RT-qPCR for the presence of SARS-CoV-2 gRNA and DI genomes (Fig. 3e [raw Ct values] and Supplementary Fig. 5a [data normalized to GAPDH mRNA levels and expressed relative to CoV-2 gRNA levels]). As expected, RLuc mRNA was present in P0 transfected cells, but not in SP2- or SP4-infected cells (Fig. 3e and Supplementary Fig. 5a). SARS-CoV-2 gRNA was readily detected in all infected cells. The USJ and DSJ, unique characteristics of the DI genomes, were present in transfected (P0) cells, as well as in SP2- and SP4-infected cells (Fig. 3e). GAPDH mRNA and 18 S rRNA levels were similar across samples (Supplementary Fig. 5b). In the absence of SARS-CoV-2 virus, GI.50 and GI.616 genomes were not present in SP2 samples, consistent with DI replication being dependent on the parental virus (Supplementary Fig. 6). These results indicate that synthetic, recombinant DI RNA can conditionally and stably propagate in the presence of parental SARS-CoV-2 where they attenuate viral replication in a post-infection setting.

To assess if DIs could be used as conditional gene delivery vehicles, an EMCV-driven *Renilla* luciferase (EMCV/RLuc) or transcription regulatory sequence (TRS/RLuc) cassette reporter was inserted into the DSJ of GI.616 (Fig. 3f). Vero E6 cells were infected with SARS-CoV-2, and DI RNA transfections performed 1 hpi. This was then followed by four serial passages. In cells receiving SP4 virus from the RLuc transfections, only background levels of luciferase activity were detected (Fig. 3g). In contrast, cells infected with SP4 virus from GI.616-EMCV/RLuc transfections produced significant luciferase activity (Fig. 3g). However, the highest levels of luciferase were from GI.616-TRS/RLuc samples which were 320-fold higher than cells containing GI.616-EMCV/RLuc DIs (Fig. 3g). The presence of recombinant GI.616-EMCV/RLuc or GI.616-TRS/RLuc genomes reduced SARS-CoV-2 titers 15- and 30-fold, respectively (Fig. 3h). We confirmed that GI.616-TRS/RLuc produced a subgenomic mRNA containing the viral 5′ TRS-L end sequences by RT-PCR using primers targeting TRS-L and the renilla ORF (Fig. 3i, lane 3). Taken together, these results indicate that synthetic versions of the DI genomes identified herein can be used as conditional gene delivery vectors to inhibit SARS-CoV-2 replication.

### Recombinant DI genomes interfere with SARS-CoV-2 replication

**Fig. 4:**
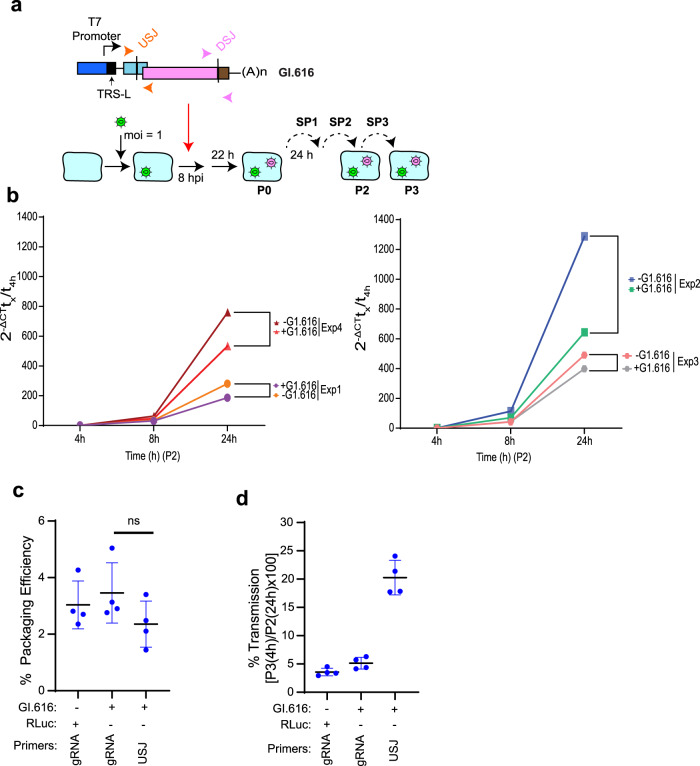
Interference of SARS-CoV-2 replication by GI.616. **a** Schematic diagram showing experimental design for assessing the effect of GI.616 on SARS-CoV-2 replication. SARS-CoV-2 and GI.616 genomes were isolated from P2 and P3 cells, as well as SP3 supernatant. **b** Growth rates (absolute gRNA levels relative to the amount at 4 h) of parental virus propagated in the presence of GI.616 (+) or RLuc (−). The data for four independent experiments is shown. **c** The percent packaged genomes upon propagation of SARS-CoV-2 in the presence or absence of GI.616. *n* = 4 independent biologically experiments ± SD. ns, not significant (*p* = 0.25). **d** Transmission efficiency of SARS-CoV-2 and GI.616. *n* = 4 independent biological experiments ± SD.

We next sought to query the mechanism by which GI.616 restricts viral replication. Following infection and transfection of Vero E6 cells, virus was serially passaged three times, and levels of viral RNA in the media and cells were determined at SP2 and SP3 (Fig. 4a). In P2-infected cells, GI.616 reduced SARS-CoV-2 gRNA levels compared to RLuc controls (Fig. 4b). Levels of SARS-CoV-2 gRNA in P2-infected cells relative to virions present in SP3 media were then compared. The presence of GI.616 did not affect the packaging or release of SARS-CoV-2 gRNA from cells (Fig. 4c). In addition, GI.616 was packaged and released at the same efficiency as SARS-CoV-2 gRNA (Fig. 4c). The presence of GI.616 did not affect the transmission of SARS-CoV-2 gRNA (Fig. 4d). However, GI.616 genomes transmitted at a rate four-fold higher compared to SARS-CoV-2 gRNA (Fig. 4d). Taken together, these results indicate that robust replication of GI.616 during the early stages of infection (by 4 h) is associated with reduced SARS-CoV-2 gRNA levels over the course of infection.

### SARS-CoV-2 DIs encode an Nsp1–10 fusion that inhibits viral replication

**Fig. 5:**
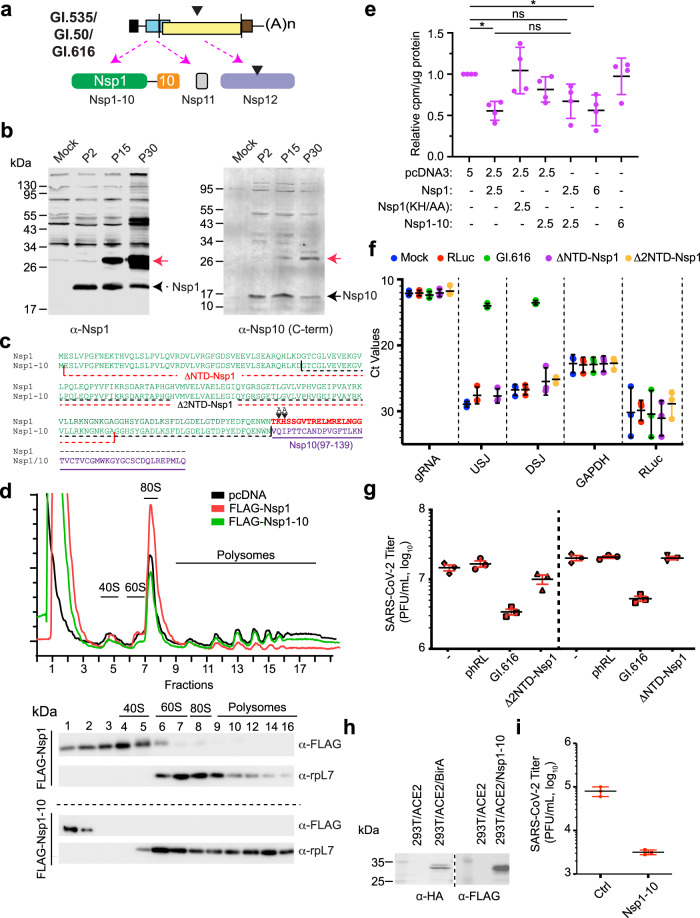
Characterization of the DI-encoded Nsp1–10 fusion product. **a** Coding potential of DIs. Black triangle indicates the 19 amino acid deletion in GI.616. **b** Western blot of extracts probed with α-Nsp1 (left) or α-Nsp10 C-terminal domain (right) antibodies. Lysates analyzed were prepared from uninfected (mock) Vero E6 cells or Vero E6 cells infected with P2, P15, and P30 (Exp #2) viral stocks. Dotted arrow denotes Nsp1, filled arrow denotes Nsp10, red arrow denotes Nsp1–10 fusion. **c** Clustal Omega alignment of Nsp1 and Nsp1–10 fusion. Amino acids indicated in red correspond to the C-terminal region of Nsp1 critical for translation inhibition. Amino acids indicated in purple are derived from Nsp10. The extent of two Nsp1 deletion mutants are indicated by dotted lines. The location of the Nsp1 KH amino acids that were mutated to AA are indicated. **d** Polysome analysis of 293 T cells transfected with the indicated expression vectors (20 µg). Cytoplasmic extracts were prepared 24 h post-transfections and polysomes analyzed by sucrose gradient sedimentation. Western blot analysis was then undertaken of protein samples obtained from individual polysome fractions. Western blots were probed with antibodies shown to the right. **e** Ectopic expression of Nsp1–10 does not inhibit translation in 293 T cells. 293 T cells were transfected with the indicated amounts of expression vector. Twenty-four hours later, cells were metabolically labeled with ^35^S-Met/Cys for 15 min. TCA precipitation was used to determine the amount of radiolabel incorporated into proteins and counts were normalized to total protein content in the extract and expressed relative to cells having received empty vector (pcDNA3; which was set to 1). *n* = 4 biologically independent experiments ± SD. ns, *p* > 0.05; *, 0.01 > *p* > 0.05 (Dunnett’s multiple comparisons test). **f** RT-qPCR analysis of RNA from P2 cells infected with the indicated DIs. RNA preps targeted by each oligo pair is shown at the bottom. Obtained Ct values are displayed. *n* = 3 biologically independent experiments ± SD. **g** Quantitation of virus titers obtained with the indicated DIs at P4. *n* = 3 biologically independent experiments ± SD. **h** Western blot of 293 T/ACE2 cells stably expressing BirA (Ctrl, control) or Nsp1–10. **i** SARS-CoV-2 virus titers obtained in 293 T/ACE2/BirA or 293 T/ACE2/Nsp1–10 cells. *n* = 3 biologically independent experiments ± SD.

SARS-CoV-2 Nsp1 is a multifunctional protein that has been implicated in blocking host translation, degradation of cellular mRNAs, and inhibition of nucleo-cytoplasmic mRNA export^29–33^. To determine if Nsp1–10 could be detected in infected cells (Fig. 5a), Western blots were performed on extracts from Vero E6 cells infected with P2, P15, or P30 stocks. Results from these experiments showed that Nsp1 (~20 kDa) was present in infected cells (Fig. 5b, left panel—black arrow), whereas a larger ~30 kDa protein that cross-reacted with antibodies to Nsp1 was present in P15- and P30-infected cells (red arrow). This protein could also be detected using an antibody targeting the C-terminal domain of Nsp10, which revealed an immuno-reactive protein at ~30 kDa (Fig. 3b, right panel—red arrow). The Nsp1–10 fusion protein encoded by the prominent DIs was confined predominantly to the cytoplasm when overexpressed in uninfected cells (Supplementary Fig. 7a).

The Nsp1 C-terminal domain is essential for blocking translation as it interacts with the mRNA entry channel to inhibit cellular protein synthesis^31–35^. However, this domain is absent from the Nsp1–10 fusion protein (Fig. 5c). Consequently, Nsp1, but not Nsp1–10, inhibited cellular translation as assessed by polysome profiling (Fig. 5d). Nsp1–10, unlike Nsp1, did not co-migrate with 40 S ribosomes in polysome gradients (Fig. 5d). These data are consistent with what we observed for a previously described Nsp1(KH/AA) mutant that does not block translation (Supplementary Fig. 7b)^31^. Nsp1, but neither Nsp1–10 nor Nsp1(KH/AA), inhibited ^35^S-Met/Cys incorporation into the nascent polypeptide chain (Fig. 5e, Supplementary Fig. 7c). Nsp1–10 was unable to rescue Nsp1-mediated inhibition of translation in cells (Fig. 5e) or in vitro (Supplementary Fig. 7d, e).

To assess the requirements of the Nsp1–10 region for DI replication, we generated two deletion mutants, ΔNTD-Nsp1 and Δ2NTD-Nsp1, in GI.616 (Fig. 5c). Propagation of the mutant DIs was compromised after co-passaging with the parental virus for two passages (Fig. 5f [raw Ct values] and Supplementary Fig. 8 [data normalized to GAPDH mRNA levels and expressed relative to CoV-2 gRNA levels]) and parental viral titers were not significantly affected (Fig. 5g). Ectopic expression of Nsp1–10 in 293 T/ACE2 cells reduced SARS-CoV-2 titers by 25-fold (Fig. 5h, i). The effect of Nsp1–10 was selective in that it did not reduce titers of Dengue type 2 virus (Supplementary Fig. 9). Although our data do not allow us to invoke a firm conclusion regarding the role of Nsp1–10 in DI replication, it does support the conclusion that Nsp1–10 is a potent inhibitor of SARS-CoV-2 replication.

### GI.616 encoded Nsp12 (Δ19aa) is inactive for polymerase activity

Lastly, we investigated what impact the Nsp12 (Δ19aa) mutation in GI.616 might have on Nsp12 activity. Based on the structure of Nsp12, the deletion of 19 amino acids is expected to shorten the distance between the finger region and palm domain of the protein and alter RNA binding (Supplementary Fig. 10a). Using a polymerase extension assay where activity is assessed using a 4-mer primer, we monitored the appearance of a 14-nts product (Supplementary Fig. 10b, lane 1). The previously described Nsp12 (SNN) active site mutant was inactive in this assay (compare lanes 13–18 to 1)^36^. Similarly, the Nsp12(Δ19aa) mutant was also compromised for polymerase activity (compare lanes 19–24 to 1). The presence of Nsp12 (SNN) (lanes 2–6) or Nsp12 (Δ19aa) (lanes 8–12) in reactions containing WT RdRP did not compromise RdRP activity, attesting to a lack of dominant-negative behavior.

## Discussion

The development of DI particles as antiviral therapeutics is being explored for several important human pathogens, including influenza A virus^37–39^, Zika virus^40^, and chikungunya virus^41^. Although several naturally arising DIs have been isolated from coronaviruses (see Introduction), our study documents the characterization of naturally arising DIs from SARS-CoV-2. A salient feature present in coronavirus DIs is the retention of sequences at the 5′ and 3′ ends of the genome – a finding consistent with stem-loops at these locations being essential for replication^1^. A common, conserved upstream junction element, fusing Nsp1 to Nsp10 was present in 80–90% percent of late passage DVGs. Since the same viral seed stock was used in both Experiments #1 and #2, we cannot formally rule out the presence of a defective genome containing Nsp1–10 in the initial seed stock, although it was not detectable by RT-qPCR in P1 (Supplementary Fig 1c). Nonetheless, whether present at P0 or arising during propagation, it was clearly positively selected for during serial passaging in two independent instances (Exp #1 and #2).

Gribble et al.^4^ mapped the patterns and frequency of genome recombination of SARS-CoV-2 and reported that >50% recombination frequencies occurred at 26 positions, with 13 of these mapping to TRS positions. Of note, none of these sites mapped to the Nsp1–10 junctions that we identified. We were unable to find primary sequence similarity/complementarity between Nsp1 and Nsp10 that could easily explain the emergence of the Nsp1–10 fusion as a consequence of discontinuous transcription or recombination. The mechanistic details of what drives the formation of this junction fusion await a better understanding of the protein-RNA interactome and elements that drive recombination during coronavirus replication.

Although the Nsp1–10 fusion arose in interferon-deficient Vero cells, its function is not restricted to this context since the expression of Nsp1–10 in ACE2-expressing 293 T cells attenuated viral replication (Fig. 5i). The Nsp1–10 protein thus appears to represent a DI-encoded protein that attenuates helper virus replication. Unlike Nsp1, Nsp1–10 did not interfere with cellular translation (Fig. 5d, e). Nsp1 is a multifunctional protein that has also been implicated in host mRNA cleavage, and blockade of mRNA export^30,31,33–35,42,43^, and current studies are aimed at assessing if these processes are affected by Nsp1–10.

There have been two reports of synthetically designed SARS-CoV-2 DIs. Yao et al.^25^ constructed a synthetic RNA, DI_1_, that has 789 5′ end sequences fused to Nsp14 (nt 19,674), and Nsp15 is fused to the last 1426 nts from the 3′ UTR. Nucleotides 19,674–20,340 were included in the design of DI_1_ since this region of the genome was thought to harbor the putative packaging signal^44^. DI_1_ was shown capable of co-propagating with SARS-CoV-2 after one viral passage^25^. Multiple passages of DI_1_ were not reported, so we do not know if this genome is stable for long-term propagation. Our results clearly indicate that nucleotides 19,674–20,340 are not necessary for packaging since none of our prominent DIs harbored this region. Chaturvedi et al.^26^ reported a second set of synthetic DIs that they termed Therapeutic Interfering Particles 1 and 2 (TIP1 and TIP2). TIP1 contains only 5′ (1–450) and 3′ end (361 nts) nucleotides, whereas TIP2 harbors ~1.5 kb from the viral 5′ end fused to ~713 nts from the 3′ end. Both TIP1 and TIP2 have an embedded EMCV IRES/mCherry inserted at the junction breakpoint. Particles harboring TIP1 could be recovered from Vero cell supernatants that had been nucleofected with TIP1 RNA and subsequently infected with SARS-CoV-2, attesting to the ability of TIP1 to be packaged in the presence of helper virus. The synthetic genomes of TIP1 and TIP2 were able to reduce viral titers in vitro and in vivo^26^. These results suggest that the Nsp1–10 fusion is not an obligate feature of DIs for attenuating parental viral titers. Of note, in our experiments, we did not detect prominent stable defective viral genomes that were <5 kbp in length by Northern blotting (Fig. 1d and Supplementary Fig. 2b, c) nor as prevalent transcript models in the nanopore sequencing data from late passages of both experiments (Fig. 1e and Supplementary Fig. 3b). These results indicate that the ~5 kb genomes we characterized are stable to long-term propagation in the presence of the parental virus.

It is unclear why most of the DI genomes at late passage retained sequences spanning Nsp10–Nsp13. A functional Nsp12 protein was not necessary for DI propagation since GI.616 encodes an Nsp12 mutant that lacks polymerase activity (Supplementary Fig. 10), yet was the most abundant DI present in Exp #2 P30 (Supplementary Fig. 3b). Whether recently identified long-range RNA interactions surrounding the frameshift site^45^ are required to balance translation and replication events remains to be investigated.

Previous work has shown that defective interfering particles can cause cyclical changes in viral titers since they not only compete with but also rely on, parental viruses for propagation. In the presence of DI, parental virus levels will drop and reach a local minimum, DI levels subsequently do the same as they are dependent on the parental virus for their replication. This, in turn, leads to parental virus levels peaking as there are minimal DI particles available for competition. With increasing passage number, this results in cyclic changes in parental virus levels and DI levels where the peak in DI levels is superimposed with a trough in parental virus levels and vice versa^14,46^ In-depth analyses of the kinetics between wild-type poliovirus and a polio DI genome co-replicating have shown that the DI genome replication and encapsidation are the two most critical parameters that affect wt virus outcome^27^. Given the apparent complexities in controlling these events for clinical applications, a more robust therapeutic strategy may be to deliver the RNA encoding the DI particle intranasally, and indeed this has been used as a successful strategy to blunt SARS-CoV-2 infections in mouse models^26,47^.

One limitation of our study is that the described DIs were isolated from interferon-deficient Vero E6 cells, and it will be important to extend these studies in the context of interferon-producing cell lines. Our results warrant further extension and validation in preclinical mouse models to assess if the synthetic genomes disclosed herein, or if the Nsp1–10 fusion protein, can exert prophylactic or therapeutic benefit.

## Methods

### Generation of defective interfering particles

Vero E6 cells (10^6^ cells/well) grown in six-well plates were infected with SARS-CoV-2 at MOI of 3 for 60 min. The virus was removed and replaced with 2 ml of fresh complete DMEM media. Cells were monitored daily, and viral supernatant was harvested after the appearance of cytopathogenic effect (CPE) between 24 and 48 h. Fifty percent of the supernatant containing passage 1 (P1) of the virus was used to infect a new batch of Vero E6 cells, and the remaining P1 virus was frozen as two equal-volume aliquots at −80 °C. After 1 h of infection, the virus was removed and replaced with 2 ml fresh complete DMEM media. Cells were monitored daily, and the supernatant was harvested after the appearance of CPE between 24 and 48 h. This was passage 2 (P2) of the virus. The same procedure was repeated until passage 30 (P30) was reached. Total cellular RNA at each passage was extracted and stored at −80 °C for future use. The same viral seed stock was used in both Experiments 1 and 2. The Institutional Biosafety Committee of the University of Alberta approved the protocol used in these studies.

### Plaque assays

Vero E6 cells were plated in 24-well plates (10^5^ cells/well) and incubated overnight at 37 °C. Virus-containing media was serially diluted (10^−1^–10^−6^) with DMEM media into 96-well plates. Then, 100 μL of each dilution was added in duplicate to Vero E6 cells in the 24-well plates, and samples were incubated at 37 °C in 5% CO_2_ for 1 h with rocking every 15 min. After 1 h incubation, the virus-containing media was removed and 1 mL of pre-warmed plaquing media (MEM media containing 2% FBS and 0.75% methylcellulose) was added to each well. Plates were incubated at 37 °C in 5% CO_2_ for 3 days to allow plaque formation. On day 3, methylcellulose overlays were gently removed, and cells were fixed by adding 1 mL of 4% paraformaldehyde in PBS to each well. After incubation at room temperature for 30 min, the fixative was removed, plates were washed with dH_2_O, and 1 mL of 1% (w/v) crystal violet in 20% methanol was added to each well. The crystal violet solution was removed after 30 min, and the plates were washed with water. Plaques were only counted in wells in which there were 5–30 plaques.

### Immunoblotting (Nsp1–10 fusion)

Vero E6 cells infected with P2, P15, and P30 of SARS-CoV-2 passage were harvested at 24 h post-infection. Cells were washed three times with PBS before lysing with RIPA buffer (50 mM Tris-HCl (pH 7.4), 150 mM NaCl, 1% Triton-X-100, 1% sodium deoxycholate, 0.1% SDS, and 1 mM EDTA) containing fresh protease inhibitors and 1 unit of Benzonase (Millipore; Burlington, MA, USA) per sample. Proteins were denatured at 95 °C for 10 min, separated by SDS-PAGE, and transferred to polyvinylidene difluoride (PVDF) membranes for immunoblotting. Imaging was performed using an Odyssey® CLx Imaging System (LI-COR Biosciences; Lincoln, NE, USA).

### Renilla luciferase assays

Vero E6 cells (10^5^ cells/well) seeded in 24-well plates were infected with SARS-CoV-2 at an MOI = 1 for 1 h and then transfected in duplicate with in vitro synthesized RLuc, GI.616-EMC/RLuc, and GI.616-TRS/RLuc RNA. The supernatant was collected at 24 h post-infection, clarified, and fifty percent of the virus was used in serial infections of Vero E6 cells. At 24 h post-infection of each passage, growth media was removed, cells were washed with PBS, and lysed with 100 μL renilla luciferase assay lysis buffer (Promega, #E2810) for 15 min at room temperature. The lysate was collected and stored at −80 °C until further use. For luciferase assays, 20 μL from each passage was aliquoted into white 96-well microplates (Greiner bio-one) in duplicates for *Renilla* luciferase activity measurements. One hundred microliters of renilla luciferase assay reagent (Promega, #E2810) was added to each well, and luciferase activity was measured immediately using a Synergy HTX plate reader (Biotek; Winooski, VT, USA). In addition to the virus supernatant used in serial infections, the remaining virus was collected for titering. Additionally, total cellular RNA from each passage of cells was also extracted and stored at −80 °C for future use.

### RNA transfections

Vero E6 cells were seeded in a 24-well plate in DMEM supplemented with 10% FBS (Gibco, #12483-020), 1% penicillin-streptomycin (Wisent, # 450-200-EL), and 1× non-essential amino acids (Wisent, # 321-011-EL) at a density of 2 × 10^5^ cells/well. The next day, the medium was changed to 200 uL Opti-MEM (Gibco, 31985070). Cells were transfected with Lipofectamine™ 3000 (Invitrogen, #L3000015), and transfection mixes were prepared as recommended by the manufacturer. Essentially, 500 ng of cap-1 mRNA was added to each well of mock- or SARS-CoV-2-infected cells and left to incubate for 22 h before downstream processing.

### Plasmids construction

The sequences of all plasmids used in the current study are provided in Supplementary Data 3.

### In vitro synthesis of capped mRNAs

Plasmids carrying DI genomes were linearized using BsmBI or Esp3I. DNA was purified by phenol-chloroform extraction, back extracted with H_2_O, passed through a 1 mL column of Sephadex™ G-50 Superfine beads (GE Healthcare, #17-0041-01), precipitated with 100% ethanol, washed with 70% ethanol, and resuspended in RNase-free water at a concentration of 1 µg/µL. Using linearized plasmid as a template, mRNA was synthesized in vitro using the T7 RiboMAX™ Express Large Scale RNA Production System (Promega, P1320) according to the manufacturer’s instructions. RNA was then capped in a one-step reaction using Vaccinia Capping System (NEB, # M2080S) and mRNA Cap 2′-O-Methyltransferase (NEB, #M0366S). RNA cleanup was performed via phenol-chloroform extraction as described above for cleanup of linearized DNA. Concentrations were quantitatively measured by NanoDrop 1000 (Thermo Scientific). Cap 1 mRNA was analyzed alongside ssRNA ladder (NEB, # N0362S) on a 1% agarose-formaldehyde denaturing gel to confirm size and quality.

### Long-range (LR)-qPCRs

To prepare full-length cDNA for LR-PCRs, total RNA was reverse transcribed according to the manufacturer’s instructions using Superscript™ IV Reverse Transcriptase (Invitrogen, #18090010) and a gene-specific primer: (5′CTCCTAAGAAGCTATTAAAATCAC3′) that targets the 3′ UTR of SARS-CoV-2. Single-strand DNA was obtained by RNase H (NEB, #M0297S) treatment and the resulting cDNA was diluted 10-fold. LR-PCRs were performed using LA-Taq DNA Polymerase Hot-Start Version (TaKaRa, #RR042B) and 2 µL of diluted cDNA as a template. Cycling conditions were implemented as recommended by the manufacturer. Primers are indicated in Supplementary Data 2. LR-PCR products were used as templates to generate smaller products that were directly sequenced by Sanger sequencing.

### RT-qPCRs

Complementary DNA was generated either with M-MuLV reverse transcriptase (NEB, M0253L) or SuperscriptTM IV VILO™ Mastermix (ThermoFisher, #11756050) using random hexamer primers. The cDNA was diluted 10-fold and used as a template for qPCR using SsoFast™ Evagreen® Supermix (Bio-Rad, #1725201). Cycling conditions consisted of an initial denaturation of 95 °C/30 s followed by 98 °C/5 s, 60 °C/5 s (40 cycles), and 65 °C to 95 °C incremented at a rate of 0.5 °C/min. for melting curve acquisition. Primer pairs used are listed in Supplementary Data 2.

### Determining the relative abundance of DVGs across viral passages

Cellular RNA from P20, P25, and P30 of Experiments #1 and #2 was reverse transcribed according to the manufacturer’s protocol using Superscript™ IV Reverse Transcriptase (Invitrogen, #18090010). The resulting cDNA was treated with RNase H (NEB, #M0297S) and diluted 10-fold. Diluted cDNA was used as a template for LR-PCR using the A1/A2 primer pair and LA-Taq DNA Polymerase Hot-Start Version (TaKaRa, #RR042A). The major long-range product was gel purified and cloned by TA cloning into pGEM-T Easy using the pGEM®-T Easy Vector System (Promega, # A1360) according to the manufacturer’s instructions or by blunt-end ligation into pBluescript II KS (+) using EcoRV. Minipreps were performed by the alkaline lysis method to obtain plasmid DNA from each clone which was then Sanger sequenced.

### Northern blot analysis

For all Northern blots, total RNA was quantified using the NanoDrop 1000 (Thermo Scientific). RNA from each sample was electrophoresed on a 1.2% formaldehyde-agarose gel. Following electrophoresis, the RNA ladder lane was excised and stained with SYBR™ Gold Nucleic Acid Gel Stain (Invitrogen). Northern blot transfers were performed onto Hybond N + membrane using 10× SSC. Following the transfer, the membrane was UV-crosslinked at 1.2 × 10^5^ µJ/cm^2^. The membrane was prehybridized with hybridization buffer (50% formamide, 10% dextran sulfate, 0.8 M NaCl, 5× Denhardt’s solution, 50 mM Tris 7.5, 0.1% sodium pyrophosphate, 100 µg/mL salmon sperm DNA, 0.5% SDS) for 16 h at 42 °C and then hybridized with radioactively probe for 16 h at 42  °C. Washes were performed at 65 °C twice for 25 min each with 0.1% SDS/2× SSC, 0.1% SDS/1× SSC, and 0.1% SDS/0.5× SSC. Autoradiographs were obtained by exposing the membrane to X-ray film (BioMax XAR, Kodak).

### Determining the growth rate, packaging efficiency, and transmission efficiency of SARS-CoV-2 and DIPs

Vero E6 cells were infected with SARS-CoV-2 at an MOI of 1, and DI mRNA was transfected 8 h post-infection. Twenty-two hours post-transfection, the resulting supernatant was serially passaged three times, once every 24 h. Cellular RNA was extracted at the indicated time points post-infection in P2 (4, 8, and 24 h) and at 4 h in P3. RNA was also extracted from P2 supernatant at 24 h. After performing RT-qPCR, the growth rate of SARS-CoV-2 was then calculated by the 2^−ΔCt^ method using GAPDH as a control. The resulting values were normalized to the 4 h time point.

To calculate the percentage of genomes packaged and transmission efficiency, the DI and SARS-CoV-2 RNA copy numbers were determined after RT-qPCR, from a standard curve established using recombinant RNA standards. The percentage of packaged DI or WT virus mRNAs was then calculated as follows: 100 × (mRNA copy number in P2 supernatant)/(mRNA copy number in P2 supernatant + mRNA copy number in P2 cells).

The transmission percentage was calculated as follows: 100 × (mRNA copy number in P3 cells at 4 hr)/(mRNA copy number in P2 cells at 24 h).

### Differential detergent fractionation

HEK-293T cells were seeded in a six-well plate at a density of 10^6^ cells/well. In each well, cells were transfected by calcium phosphate using 5 µg empty pcDNA3.1 or pcDNA3.1 expressing the indicated proteins. Twenty-four hours post-transfection, cells were scraped in cold PBS and pelleted by centrifugation at 4 °C for 10 min at 300 × *g*. Cells were lysed in 100 µL of digitonin extraction buffer (10 mM PIPES (pH 6.8), 300 mM sucrose, 100 mM NaCl, 3 mM MgCl_2_, 5 mM EDTA, 0.015% digitonin, 1 mM PMSF) on ice for 10 min, and the lysate was centrifuged at 4 °C for 10 min at 480 × *g*. The supernatant was kept as the cytosolic fraction. The digitonin-insoluble pellet was then washed once in the same volume of digitonin extraction buffer and spun at 480 × *g* for 10 min. After the supernatant was discarded, the pellet was resuspended in the same volume of Triton-X-100 extraction buffer (10 mM PIPES (pH 6.8), 300 mM sucrose, 100 mM NaCl, 3 mM MgCl_2_, 5 mM EDTA, 0.5% Triton-X-100, 1 mM PMSF), left on ice for 15 min, and was centrifuged at 5000 × *g* for 10 min. at 4 °C. The supernatant (membrane/organelle fraction) was discarded, and the Triton-insoluble pellet was lysed in 100 µL of 1x sample buffer to obtain the nuclear fraction. The same cell equivalents of cytosolic and nuclear fractions were resolved on a 10% SDS-PAGE gel, and proteins were analyzed by western blotting.

### Polysome fractionation

HEK-293T cells were seeded in 10 cm dishes at 5 × 10^6^ cells/well in DMEM supplemented with 10% BGSS, 1% Pen-Strep, and 1% L-glutamine (Wisent). The next day, cells were transfected by calcium phosphate using 20 µg of each plasmid. Cells were washed, and fresh medium was reapplied 6–8 h post-transfection. Twenty-four hours post-transfection, cells were harvested in ice-cold PBS containing 100 µg/ml cycloheximide. Cells were pelleted at 4 °C and lysed in 425 µL hypotonic lysis buffer (5 mM Tris-Cl (pH 7.5), 2.5 mM MgCl_2_, 1.5 mM KCl). Cycloheximide (100 µg/ml), DTT (2 mM), Triton-X-100 (0.5%), and sodium deoxycholate (0.5%) were each added to the indicated final concentrations and the samples briefly vortexed. Lysates were cleared by centrifugation at 16,000 × *g* for 2 min at 4 °C. Lysate (400 µL) was layered onto a 10–50% sucrose gradient. The gradients were centrifuged at 217,000 × *g* for 2 h at 4 °C in an SW40 Beckman rotor. Fractions were collected using the Teledyne ISCO Foxy R1 collector while monitoring the UV 254 profile. Proteins were precipitated from fractions with 10% trichloroacetic acid and collected by centrifugation at 16,000 × g for 30 min. at 4 °C. The pellet was washed with 500 µL acetone and centrifuged at 4 °C for 10 min. at 16,000 × *g*, and dried under vacuum (Eppendorf Vacufuge). Protein pellets were resuspended in 1× SDS sample buffer and analyzed on a 10% SDS-PAGE gel. Resolved proteins were transferred at 4 °C onto a PVDF membrane (Bio-Rad) and probed by immunoblotting.

### Antibodies

Antibodies used in this study were: anti-Nsp1 (GeneTex, GTX135612), anti-Nsp10 (Pro-Sci Inc, #9179), anti-FLAG (Sigma-Aldrich, #F1804), anti-RPL7 (Novus Biologicals, #NB100-2268), anti-GAPDH (Abcam, #ab8245), anti-β-actin (Abcam, #ab8226), anti-eEF2 (CST, #2332), and anti-hnRNPA1 (CST, #8443).

### Measurement of protein synthesis in vitro and in cellula

For in vitro translations, a FF/EMCV/Ren bicistronic reporter mRNA was transcribed in vitro using SP6 RNA Polymerase (NEB, #M0207S) and co-transcriptionally capped with m^7^GpppG RNA Cap Analog (NEB, #S1404S). Nuclease-treated rabbit reticulocyte lysate (RRL) (Promega, #L4960) was programmed with 20 ng/µL mRNA and supplemented with the indicated recombinant proteins. Recombinant proteins were preincubated with lysates for 5 min at 30 °C before the addition of mRNA. After 1 h at 30 °C, reactions were placed on ice, and 10 µg/mL cycloheximide was added to stop the reaction. Luciferase activity was measured on a Berthold Lumat LB 9507 luminometer.

For [^35^S]-methionine/cysteine labeling, HEK-293T cells were seeded in a six-well plate at a density of 10^6^ cells/well in DMEM supplemented with 10% BGSS, 1% Pen-Strep, and 1% L-glutamine. Cells were transfected with the indicated pcDNA3.1 expression plasmids and 6–8 h post-transfection, washed three times with PBS, trypsinized, and seeded into a 24-well plate in technical duplicates. Twenty-four hours post-transfection, the medium was exchanged for 45 min with methionine-free, cysteine-free DMEM (Gibco, #21013-024) supplemented with 10% FBS, after which 22 µCi of ^35^S-Methionine/Cysteine Protein Labeling Mix (Perkin Elmer, #NEG772007MC) was added per well. Labelling was performed for 15 min at 37 °C/5% CO_2_ after which cells were lysed in RIPA buffer (20 mM Tris-Cl (pH 8.0), 100 mM NaCl, 1 mM EDTA, 1 mM EGTA, 1% NP-40, 0.5% sodium deoxycholate, 0.1% SDS, 0.01 mg/mL aprotinin, 0.002 mg/mL leupeptin, 2.5 µM pepstatin, and 1 mM PMSF). Lysates were spotted onto 3MM Whatman paper, and the proteins precipitated with 10% trichloroacetic acid (TCA). TCA-insoluble radiolabeled proteins were quantified by scintillation counting, and counts were normalized to the total protein amounts determined for each sample by the DC Protein Assay (Bio-Rad, #5000112). Lysates were also resolved by SDS-PAGE and transferred onto a PVDF membrane (Bio-Rad) when western blots had to be performed.

### Recombinant protein purification

Recombinant His_6_-tagged Nsp1, Nsp1 (K164A/H165A), and Nsp1–10 proteins were purified from BL21 (DE3) cells expressing pET15b-based expression vectors. Single colonies were picked, and 20 mL cultures were grown overnight. Cultures were used to inoculate 1 L of LB/amp (100 µg/mL) and induced with IPTG (0.5 mM) when the OD_600_ had reached 0.8, at which point cultures were moved to 18 °C for 16 h. Cells were pelleted by centrifugation for 20 min, resuspended in 20 mL Nsp1 sonication buffer (50 mM HEPES-KOH(pH 7.6), 500 mM KCl, 5 mM MgCl_2_, 40 mM imidazole, 10% glycerol, 1 mM PMSF, 0.01 mg/mL aprotinin, 0.002 mg/mL leupeptin, 2.5 µM pepstatin, 0.5 mM dithiothreitol), and lysed using by sonication (Heat systems ultrasonics; 10 pulses @ 1 pulse/sec). The lysate was cleared by centrifugation at 4 °C for 45 min at 48,000 × *g*. Proteins were purified on Ni-NTA agarose beads (Qiagen, #30210), washed twice with Nsp1 sonication buffer, and eluted with Nsp1 elution buffer (50 mM HEPES-KOH (pH 7.6), 500 mM KCl, 5 mM MgCl_2_, 300 mM imidazole, 10% glycerol). Eluted protein fractions were dialyzed overnight at 4 °C in Nsp1 dialysis buffer (40 mM HEPES-KOH (pH 7.6), 200 mM KCl, 4 mM MgCl_2_, 10% glycerol, 1 mM dithiothreitol).

### Direct RNA nanopore sequencing

Before sequencing, the extracted total RNA was quantified with the “Qubit RNA high sensitivity” quantification kit (Q32855; ThermoFisher Scientific), and its quality was profiled on a “High Sensitivity RNA ScreenTape” (5067–5579; Agilent). Only high-quality samples were sequenced. The total RNA was sequenced on a MinION flow-cell (FLO-MIN106; Oxford Nanopore Technologies) using the “Direct RNA sequencing” library preparation kit (SQK-RNA002; Oxford Nanopore Technologies). We followed the SQK-RNA002 library preparation protocol (version DRS_9080_v2_revM_14Aug2019) as provided by Oxford Nanopore Technologies (abbreviated as ONT) with the following modifications. The library preparation started with 2 µg of total RNA for passages 1, 14 and 30 of Exp #1 and 1 µg of total RNA for passages 1 and 29 of Exp #2, 3 µg of total RNA for passage 15 of Exp #2.

In cases where the starting material was 1 µg of total RNA, we used the following protocol. The first adaptor of the library preparation kit was ligated on the RNA in a 15 µl solution with the following components: 3 µl of NEBNext Quick Ligation Reaction Buffer (stock: 5×; B6058; NEB), 1 µg of total RNA, 0.5 µl of Recombinant RNase Inhibitor (stock: 40 Units/µl; 2313 A; Takara), 1 µl of RT Adapter (RTA; ONT); 1.5 µl of T4 DNA ligase (stock: 2 M U/mL; M0202; New England Biolabs), top up the solution to 15 µl with nuclease-free water. This solution was incubated at room temperature for 20 min and subsequently mixed with a 23 µl solution named “reverse transcription master mix” that had the following components: 9 µl of nuclease-free water, 2 µl of 10 mM dNTPs (N0447S; NEB), 8 µl of 5× SSIV RT buffer (18090010; ThermoFisher Scientific), 4 µl of 0.1 M DTT (18090010; ThermoFisher Scientific). Next, 2 µl of SuperScript IV reverse transcriptase (18090010; ThermoFisher Scientific) were added, and the whole reaction was incubated at 50 °C for 2 h and 50 min, then at 70 °C for 10 min, and then the sample was brought to 4 °C before proceeding with a 1.8× volume of “RNAClean XP” beads cleanup (A63978; Beckman Coulter) and one wash of 150 µl with 70% EtOH. The material was then eluted from the beads with 20 µl of nuclease-free water, and the second adaptor was ligated in a 40 µl solution containing the following: 20 µl of reverse-transcribed RNA, 8 µl of NEBNext Quick Ligation Reaction Buffer (stock: 5×; B6058; NEB), 6 µl of RNA Adapter (RMX; ONT), 2.5 µl of nuclease-free water, 0.5 µl of Recombinant RNase Inhibitor (stock: 40 Units/µl; 2313 A; Takara), 3 µl of T4 DNA Ligase (stock: 2 M U/mL; M0202; NEB). The reaction was incubated at room temperature for 20 min followed by 1× volume of “RNAClean XP” beads cleanup (A63978; Beckman Coulter) and two washes of 150 µl with the Wash Buffer (WSB; ONT). The material was eluted, from the beads, in 21 µl of Elution Buffer, and 1 µl of the solution was quantified with the “Qubit 1X dsDNA high sensitivity” kit (Q3323; ThermoFisher Scientific). Approximately 200–250 ngs of RNA/cDNA hybrid were recovered. After priming the MinION flow-cell as per the ONT protocol we loaded the following solution: 20 µl of prepped RNA/cDNA hybrid in Elution Buffer, 17.5 µl of nuclease-free water, 37.5 µl of RRB buffer (ONT). The duration of the sequencing run was up to 72 h or until no pores were available for sequencing.

In cases where the starting material was 2 or 3 µg of total RNA, we used the following protocol. The first adaptor of the library preparation kit was ligated on the RNA in a 30 µl solution with the following components: 6 µl of NEBNext Quick Ligation Reaction Buffer (stock: 5×; B6058; NEB), 2 or 3 µg of total RNA, 1 µl of Recombinant RNase Inhibitor (stock: 40 Units/µl; 2313 A; Takara), 1 µl of RT Adapter (RTA; ONT); 3 µl of T4 DNA ligase (stock: 2 M U/mL; M0202 NEB), top up the solution to 30 µl with nuclease-free water. The solution was incubated at room temperature for 20 min and subsequently mixed with a 46 µl solution named “reverse transcription master mix” with the following components: 18 µl of nuclease-free water, 4 µl of 10 mM dNTPs (N0447S; New England Biolabs), 16 µl of 5× SSIV RT buffer (18090010; ThermoFisher Scientific), 8 µ of 0.1 M DTT (18090010; ThermoFisher Scientific). Next, 4 µl of SuperScript IV reverse transcriptase (18090010; ThermoFisher Scientific) were added, and the whole reaction was incubated at 50 °C for 2 h and 50 min, then at 70 °C for 10 min, and the sample was brought to 4 °C before proceeding with a 1.8× volume of “RNAClean XP” beads cleanup (A63978; Beckman Coulter) and one wash of 300 µl with 70% EtOH. The material was then eluted from the beads with 40 µl of nuclease-free water, and the second adaptor was ligated in a 80 µl solution containing the following: 40 µl of reverse-transcribed RNA, 16 µl of NEBNext Quick Ligation Reaction Buffer (stock: 5×; B6058; New England Biolabs), 6 µl of RNA Adapter (RMX; ONT), 11 µl of nuclease-free water, 1 µl of Recombinant RNase Inhibitor (stock: 40 Units/µl; 2313 A; Takara), 6 µl of T4 DNA Ligase (stock: 2 M U/mL; M0202; New England Biolabs). The reaction was incubated at room temperature for 20 min followed by 1× volume of “RNAClean XP” beads cleanup (A63978; Beckman Coulter) and two washes of 150 µl with the Wash Buffer (WSB; ONT). The material was eluted, from the beads, in 38.5 µl of Elution Buffer, and 1 µl of the solution was quantified with the “Qubit 1X dsDNA high sensitivity” kit (Q33230; ThermoFisher Scientific). Approximately 400–750 ngs of RNA/cDNA hybrid were recovered. After priming the MinION flow-cell as per the ONT protocol we loaded the following solution: 37.5 µl of prepped RNA/cDNA hybrid in Elution Buffer, 37.5 µl of RRB buffer (ONT). The duration of the sequencing run was up to 72 h or until no pores were available for sequencing.

### Nanopore data analysis pipeline and analysis files

A detailed description of our nanopore analysis pipeline is presented in Supplementary Note 1. A summary statistics table for the direct RNA nanopore runs is provided as Supplementary Data 4, along with an explanatory section in Supplementary Note 2 and accompanying figure in Supplementary Fig. 13. Intermediate and final files from the nanopore analysis pipeline are provided as Supplementary Data 5 in.zip format with the description of each file given in the Supplementary Note 2.

### Statistics and reproducibility

Statistical analysis was performed using GraphPad Prism software (version 6.01; GraphPad Software Inc., La Jolla, CA, USA). Results are expressed as means ± SD. The mean comparison was carried out using two-tailed Student’s *t-*test or ANOVA. The number of biologically independent replicates performed for each experiment is indicated in the figure legends.

### Reporting summary

Further information on research design is available in the Nature Research Reporting Summary linked to this article.

## Supplementary information

Supplementary Information

Description of Additional Supplementary Files

Supplementary Data 1

Supplementary Data 2

Supplementary Data 3

Supplementary Data 4

Supplementary Data 5

Supplementary Data 6

RN-Reporting Summary

## Data Availability

All data and materials used in the analyses are available to any researcher for purposes of reproducing or extending the analyses. Uncropped and unedited gels/blots are presented in Supplementary Figs. 11 and 12. Numerical source data for all main and supplementary figures is provided in Supplementary Data 6. Plasmids used in this study (Supplementary Data 3) have been deposited at Addgene under the following accession codes: pHiC Int1K GI.50 (191822), pHiC Int1K GI.616 (191864), pHiC Int1J GI.616/EMCV-Ren (191865), pHiC Int1J GI.50/EMCV-Ren (191866), pHiC Int1K GI.616/ΔNTD-Nsp1 (191867), pHic Int1K GI.616/Δ2NTD-Nsp1 (191868), pHiC Int1K GI.616-TRS Ren (191869), phRL-polyA (191870), pcDNA3.1 3xFLAG-Nsp1 (191871), and pcDNA3.1 3xFLAG-Nasp1–10 (191872) and can be obtained under a materials transfer agreement (MTA). All other data are available from the corresponding author on reasonable request. Requests should be made to J.P. (jerry.pelletier@mcgill.ca). The fastq files of the raw sequencing data are deposited in the public repository—Sequence Read Archive database under the BioProject ID PRJNA850004^48^.

## Code availability

The pipeline used for nanopore sequence analysis is available in the Supplementary Note 1 and Supplementary Data 5.zip files.
