# Supplementary material for: Evolution of naturally arising SARS-CoV-2 defective interfering particles
Source: Commun Biol. 2022 Oct 27;5:1140. doi: 10.1038/s42003-022-04058-5 (PMC9610340; doi:10.1038/s42003-022-04058-5)
Supplement: Supplementary file 1 — Supplementary Information [file 42003_2022_4058_MOESM1_ESM.pdf]

SUPPLEMENTARY INFORMATION

Evolution of Naturally Arising SARS-CoV-2 Defective Interfering Particles

Samer Girgis[1, †] Zaikun Xu[2, †], Spyros Oikonomopoulos[3], Alla D. Fedorova[4,5] Egor P. Tchesnokov[6], Calvin J. Gordon[6], T. Martin Schmeing[1], Matthias Götte[6], Nahum Sonenberg[1,7], Pavel V. Baranov[4], Jiannis Ragoussis[3,8,9], Tom C. Hobman[2,6,10,11,‡] and Jerry Pelletier[1,7,12, ‡]

[1]Dept. Biochemistry, McGill University, Montreal, Quebec, Canada, H3G 1Y6; [2]Dept. Cell Biology, U Alberta, Edmonton, Alberta, Canada, T6G 2H7; [3]McGill Genome Centre, McGill University, Montreal, Quebec, Canada; [4]School of Biochemistry and Cell Biology, University College Cork, Cork, Ireland; [5]SFI Centre for Research Training in Genomics Data Science, University College Cork, Cork, Ireland; [6]Dept Medical Microbiology and Immunology, U Alberta, Edmonton, Alberta, Canada, T6G 2E1;[7]Rosalind and Morris Goodman Cancer Institute, Montreal, Quebec, Canada H3A 1A3; [8]Department of Human Genetics, McGill University, Montreal, Quebec, Canada; [9]Dept Bioengineering, McGill University, Montreal, Quebec, Canada; [10]Li Ka Shing Institute of Virology, U Alberta, Edmonton, Alberta, Canada T6G 2E1; [11]Women & Children's Health Research Institute, U Alberta, Edmonton, Alberta, Canada, T6G 1C9; [12]Dept Oncology, McGill University, Montreal, Quebec, Canada H3A 1G5.

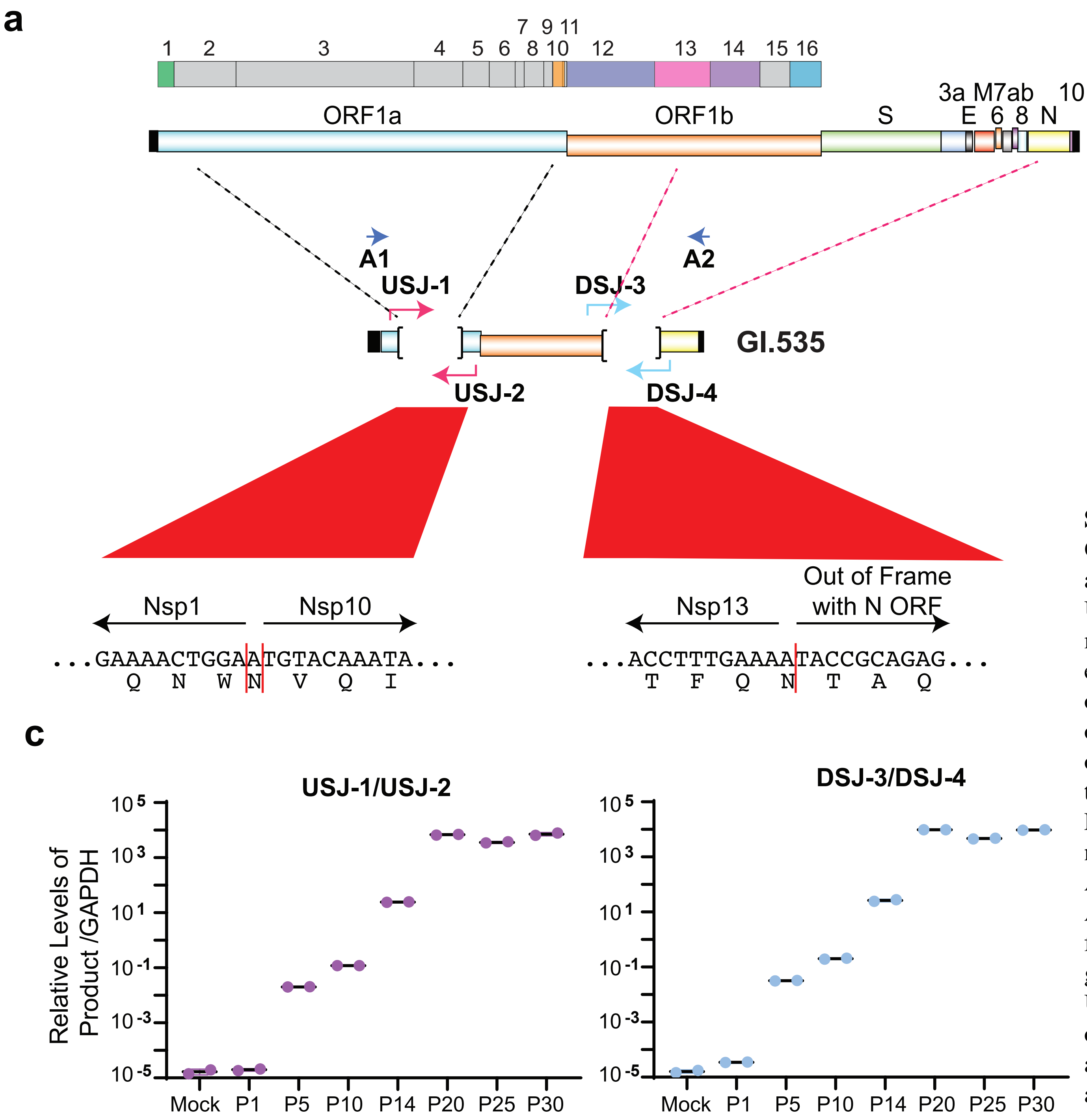

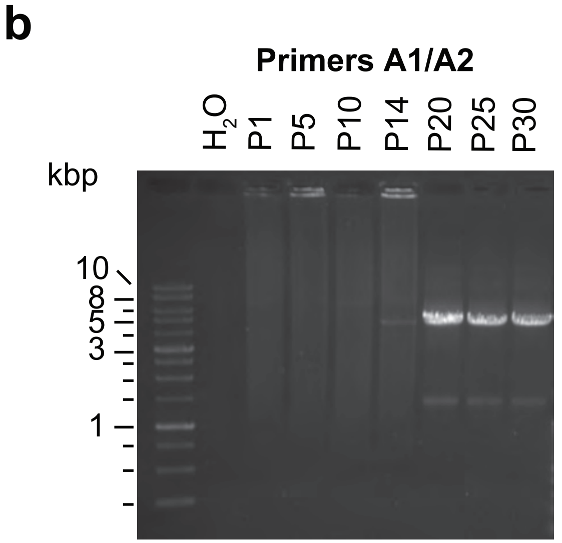

Supplementary Figure 1. Characterization of GI.535. a. Location of primers used for LR-PCR amplification (A1/A2) and for assessing presence of USJ (US-J/1/USJ-2) and DSJ (DSJ-3/DSJ-4) fragments in GI.535. Shown are the nucleotide sequences flanking the breakpoints in GI.535. Note that the origin of the A base at the 5' USJ remains ambiguous due to the presence of adenosine on both sides of the junction - therefore we cannot determine if this base originated from Nsp1 or Nsp10. **b.** End-point PCR showing emergence of most prominent DVGs at P20 with stable maintenance to P30. Amplifications were performed using primers A1/A2. Products were obtained following 30 amplification cycles and analyzed on a 0.8% agarose/TAE gel. **c.** RT-qPCR analysis of DVG breakpoints using USJ and DSJ primer pairs. RNA from infected cells of the indicated passages was reverse transcribed and cDNA used as template for qPCRs. The relative abundance of each PCR product was calculated using the ΔCt method and set relative to GAPDH. n=2.

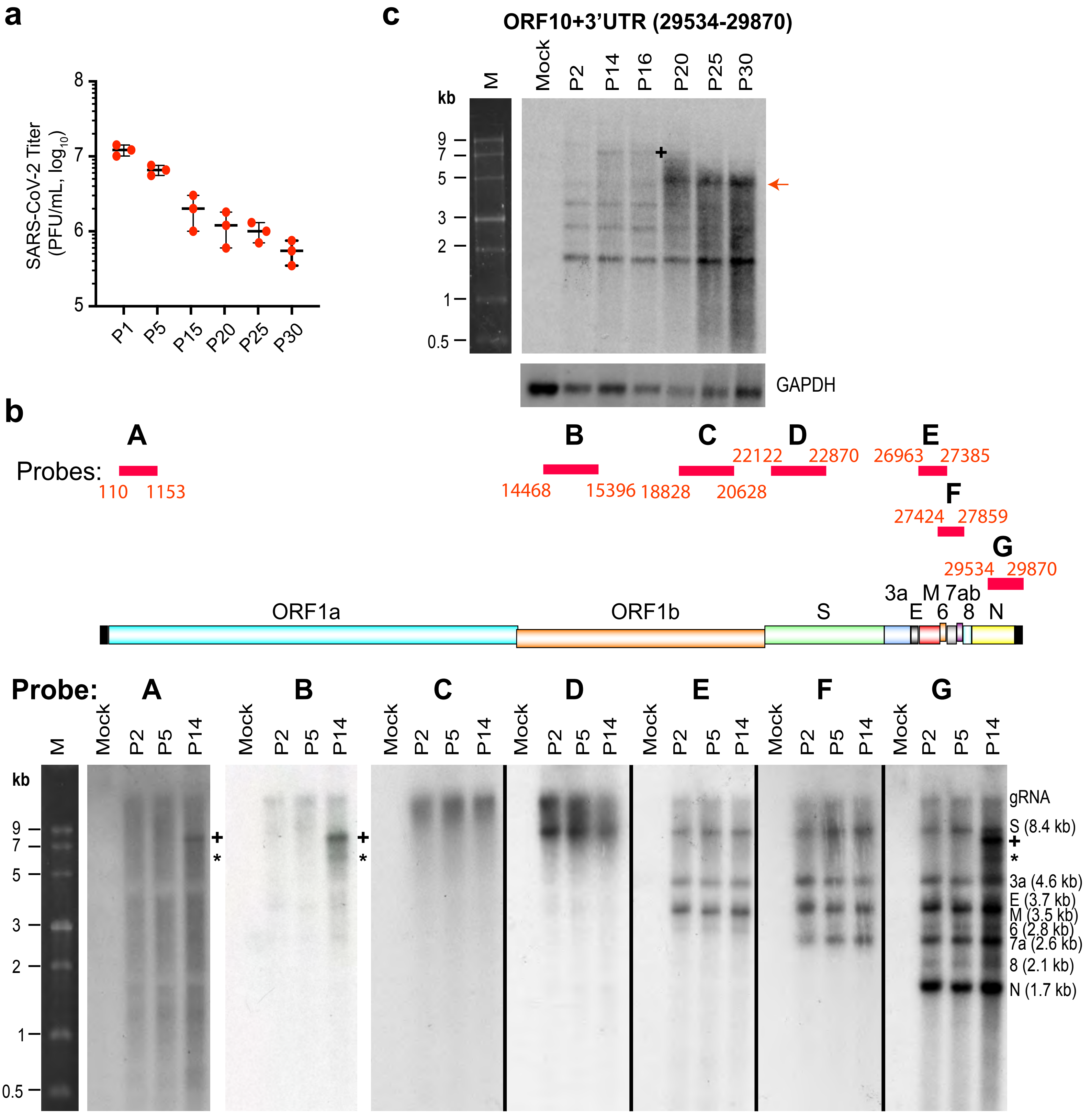

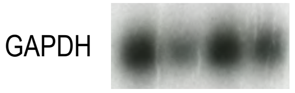

Supplementary Figure 2. Results from Exp#2 showing independent isolation of SARS-CoV-2 DVGs. a. Quantitation of virus titers obtained from the indicated passages. n=3 ± SD. **b.** Top: Schematic diagram of the SARS-CoV-2 genome illustrating ORF organization. Red boxes with nucleotide coordinates and upper-case letters denote location of 32P-labelled probes used for Northern blotting. Bottom: Northern blot analysis performed on intracellular RNA isolated from the indicated passages. RNA markers (NEB) are indicated to the left and size distribution is presented in kilobases (kb). The assignment of sgRNAs is based on predicted size. The plus sign and asterisks highlight DVGs present in P14. The same Northern blot was used in all probings shown in this panel. GAPDH was used to assess mRNA quality. Mock, uninfected cells; gRNA, genomic RNA. c. Northern blot analysis performed on RNA isolated from SARS-CoV-2 infected cells at the indicated passages. Red arrow highlights

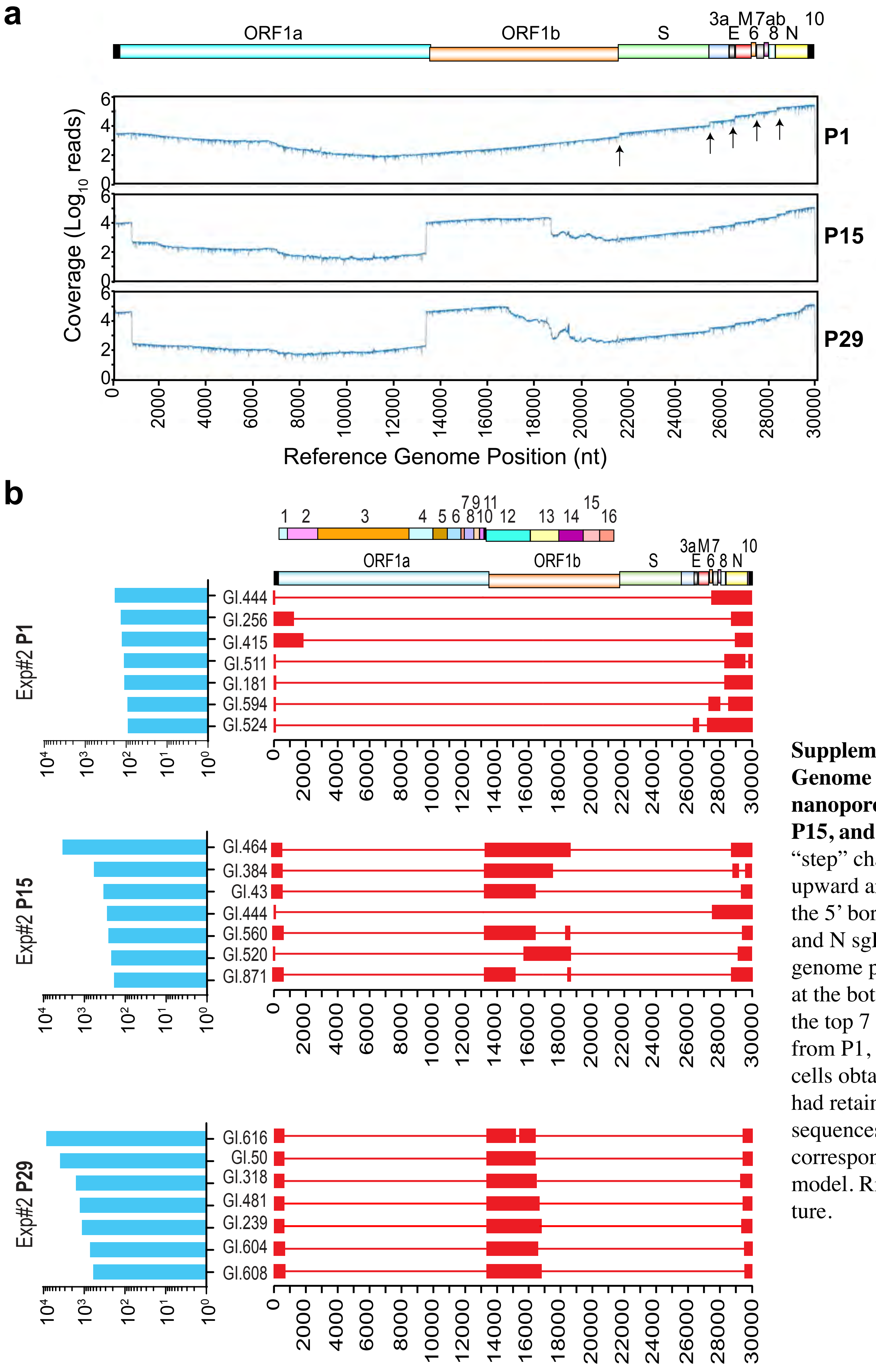

Supplementary Figure 3. Genome coverage of nanopore DRS data from P1, P15, and P29 of Exp#2. a. The "step" changes (indicated by upward arrows in P1) occur at the 5' border of the S, 3a, E, 6, and N sgRNAs. The reference genome position (nt) is shown at the bottom. **b.** Architecture of the top 7 most abundant DVGs from P1, P15, and P29 infected cells obtained in Exp#2 and that had retained 5' and 3' end sequences. Left: Read counts corresponding to the transcript model. Right: DVG architecture.

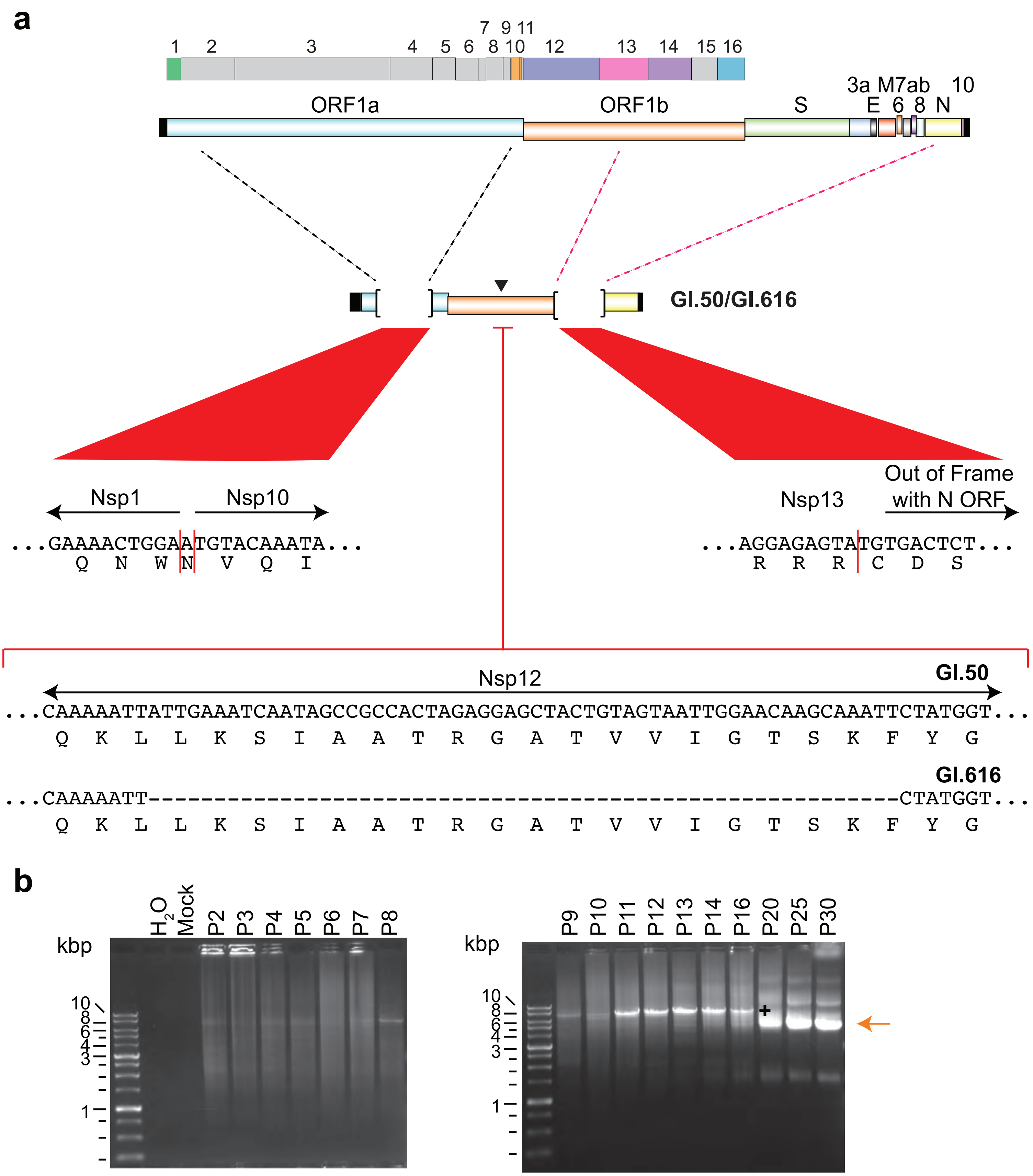

Supplementary Figure 4. Architecture of GI.616 and GI.50. a. Inverted black triangle denotes 19 amino acid deletion in GI.616. GI.50 and GI.616 harbor identical US and DS junctions. **b.** End-point PCR showing emergence of most prominent DVG from P11-P16 (indicated by a cross) and from P20-P30 (red arrow). Amplifications were performed using primers A1 and A2. Products were obtained following 30 amplification cycles and analyzed on a 0.7% agarose/TAE gel.

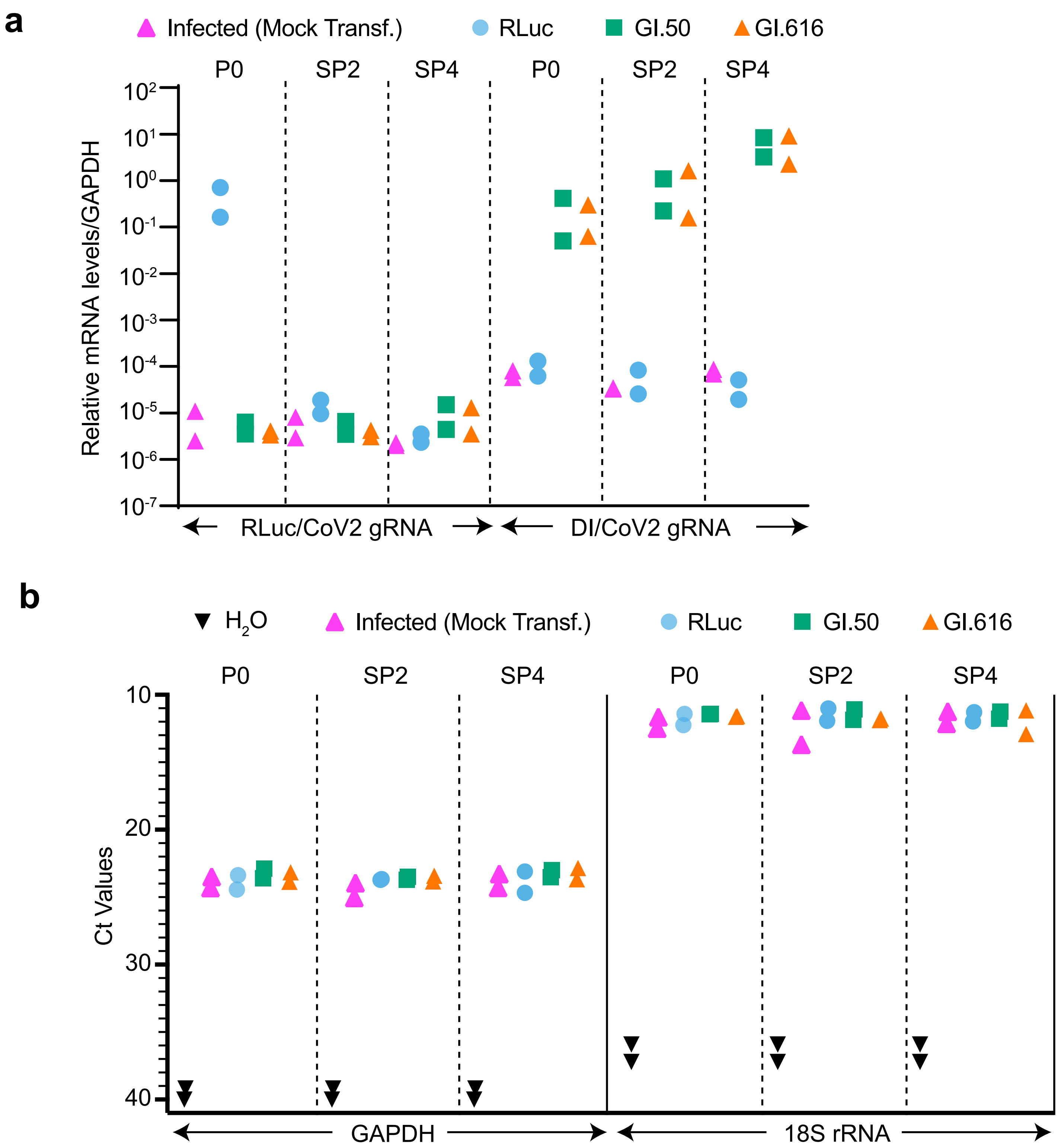

Supplementary Figure 5. Recombinant DI genomes show long term stability. a. RT-qPCR analysis of RNA from P0, SP2, and SP4 infected cells. RLuc, DI USJ and DSJ, and CoV-2 gRNA RNA levels were calculated as a fold change relative to GAPDH using the $2^{-\Delta CT}$ method. Values corresponding to the USJ and DSJ junction were averaged to yield a final DI level. Lastly, RLuc and DI levels were expressed relative to CoV-2 gRNA levels. **b.** RT-qPCR analysis of RNA from P0, SP2, and SP4 infected cells. The identity of RNAs targeted by each oligo pair is shown at the bottom. Obtained Ct values are displayed. n=2.

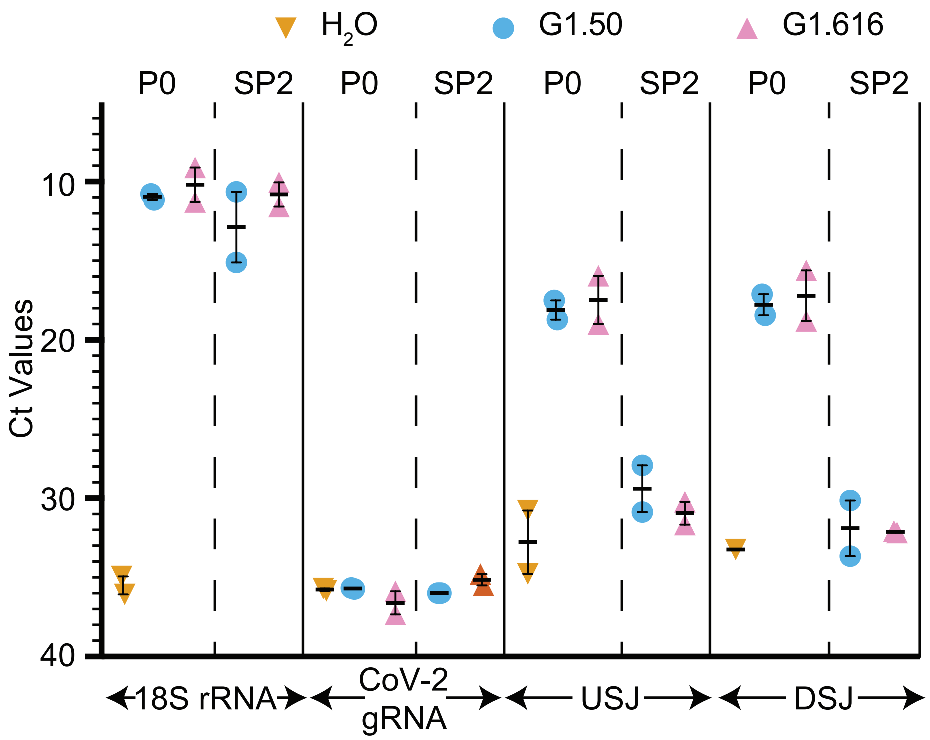

Supplementary Figure 6. Synthetic DI genomes are conditional on SARS-CoV-2 virus for replication. Vero E6 cells were transfected with GI.50 or GI.616 RNA, media collected 22 h later, clarified and applied to fresh cells for two serial passages. RT-qPCR analysis of 18S rRNA, SARS-CoV-2 gRNA, and the DI-specific USJ and DSJ sites from uninfected P0 and SP2 cells. RNA or regions targeted by each oligo pair is shown at the bottom. Obtained Ct values are displayed. n=2.

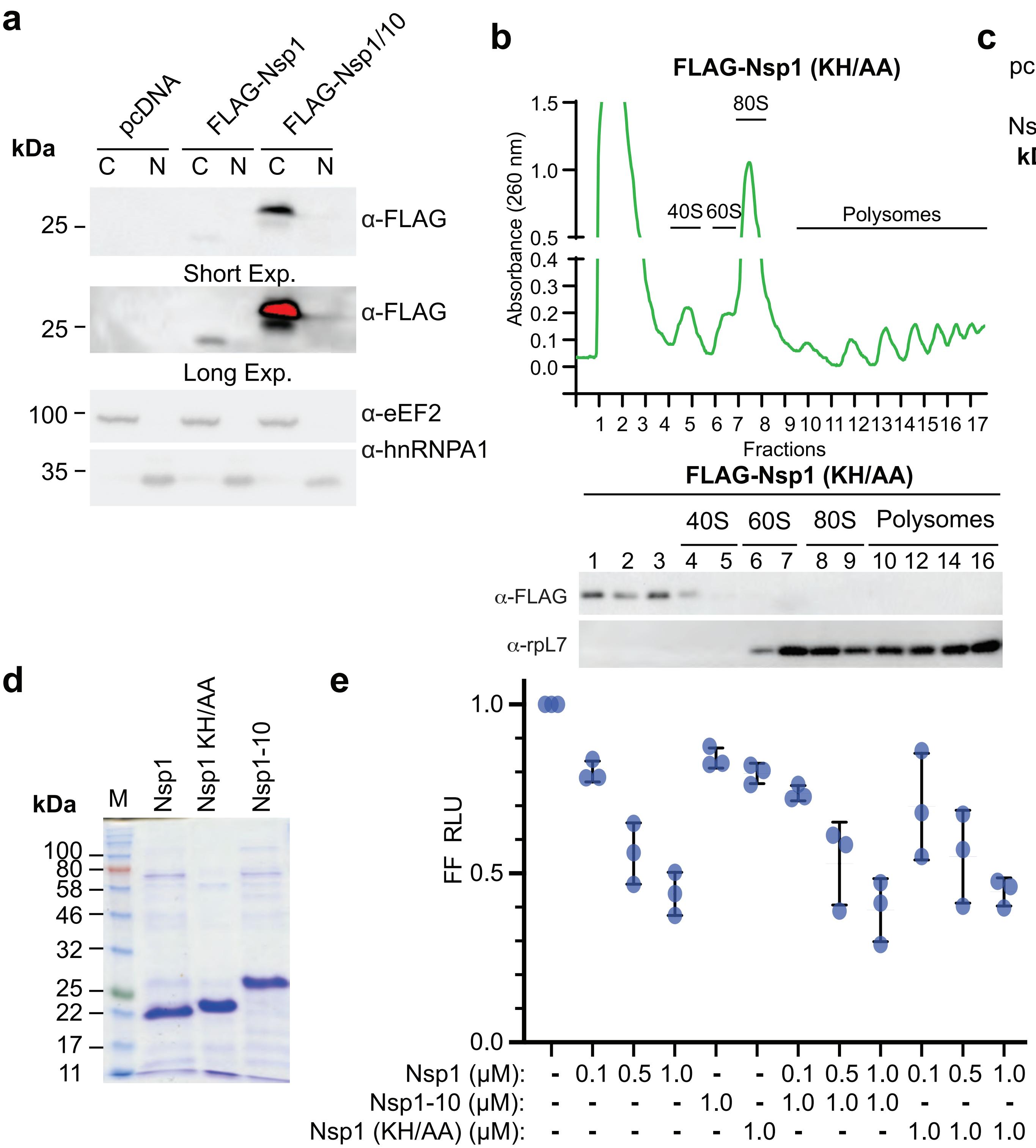

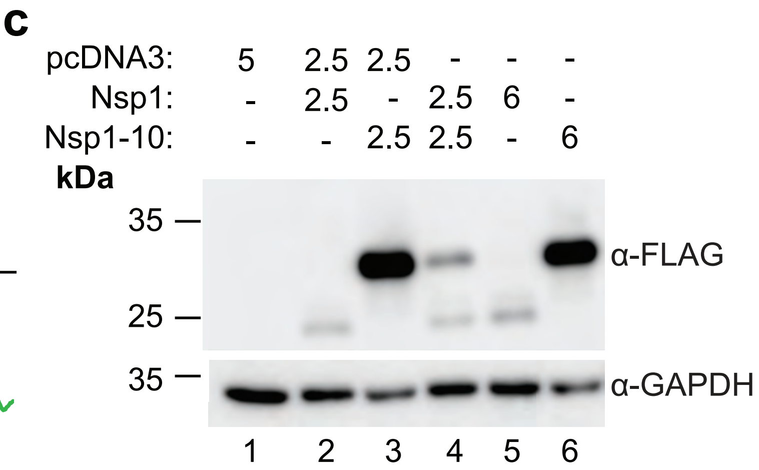

Supplementary Figure 7. Characterization of Nsp1-10. a. Nsp1-10 is predominantly a cytoplasmic protein. pcDNA-based expression vectors were transfected into 293T cells, and 48 h later cells were harvested in PBS. Following subcellular fractionation (C, cytoplasm; N, nuclear), proteins were resolved on a 10% SDS-polyacrylamide gel, transferred to immobilon PVDF membrane, and probed with antibodies indicated to the right. eEF2 and hnRNPA1 were used as loading controls for cytoplasmic and nuclear fractions, respectively. **b.** Polysome analysis of 293T cells transfected with FLAG-Nsp1 (KH/AA) expression vector (10 μg). Cytoplasmic extracts were prepared 24h post-transfections and polysomes analyzed by sucrose gradient sedimentation. Western blot analysis of protein samples obtained from individual fractions are shown below the polysome gradient. Fraction numbers are indicated to the top. Western blots were probed with antibodies shown to the right. **c.** Western blot analysis of protein samples obtained in Fig. 5e. Protein extracts were prepared from cells transfected with the indicated expression vectors, resolved on a 10% SDS-polyacrylamide gel, and transferred to immobilon PVDF membranes. Western blots were probed with antibodies shown to the right. **d.** Coomassie stain of 10% SDS-PAGE of purified recombinant protein used in in vitro translation assays. **e.** *In vitro* inhibition of translation by recombinant Nsp1, but not Nsp1-10. Relative luciferase values obtained following in vitro translations of RRL programmed with 20 μg/mL FF/HCV/Ren mRNA and supplemented with the indicated amounts of recombinant protein. RRL was pre-incubated with recombinant protein for 5 mins at 30°C before the addition of FF/HCV/Ren mRNA. Luciferase values are set relative to those obtained from extracts that received only protein storage buffer. n= 3 ± SD.

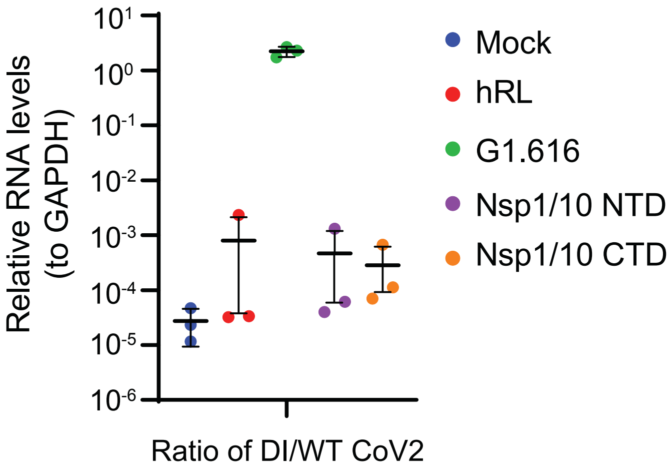

Supplementary Figure 8. RT-qPCR analysis of RNA from SP4 infected cells. For each of the transfections, the DI USJ and DSJ, and CoV-2 gRNA RNA levels were calculated as a fold change relative to GAPDH using the $2^{-\Delta CT}$ method. For Nsp1-10 Δ 2NTD, only DSJ levels were assessed due to absence of an USJ. The values corresponding to the USJ and DSJ were averaged to calculate DI levels. Finally, DI levels were expressed relative to CoV-2 gRNA levels.

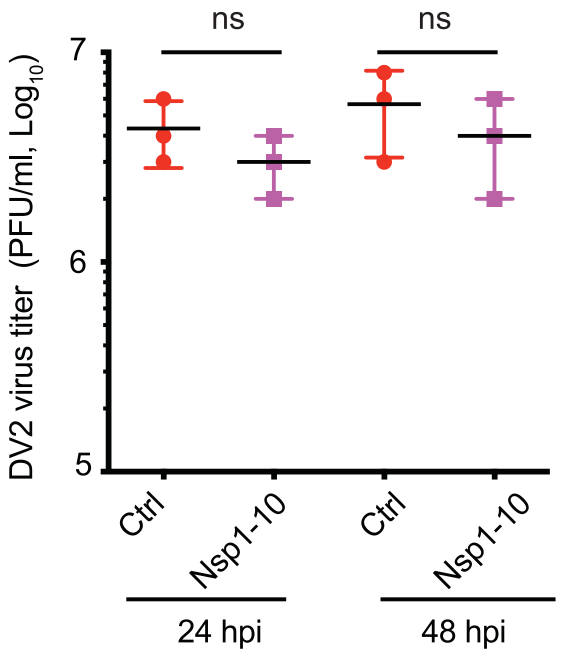

Supplementary Figure 9. Dengue type 2 virus titers obtained in 293T/ACE2/BirA (Ctrl) or 293T/ACE2/Nsp1-10 (Nsp1-10) cells. n=3 ± SD, ns – not significant.

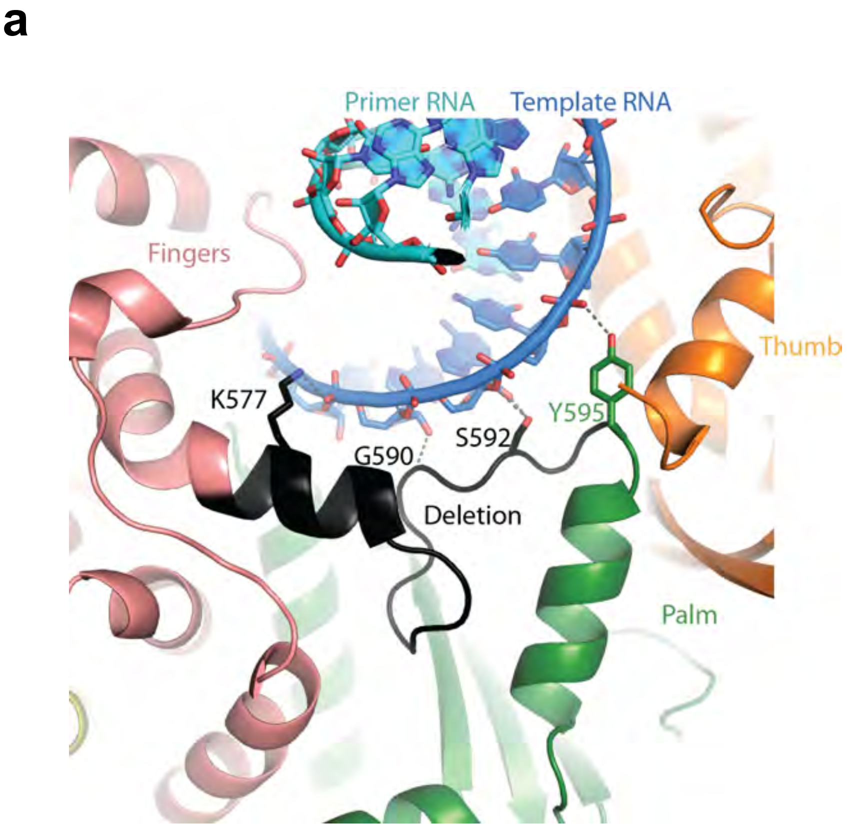

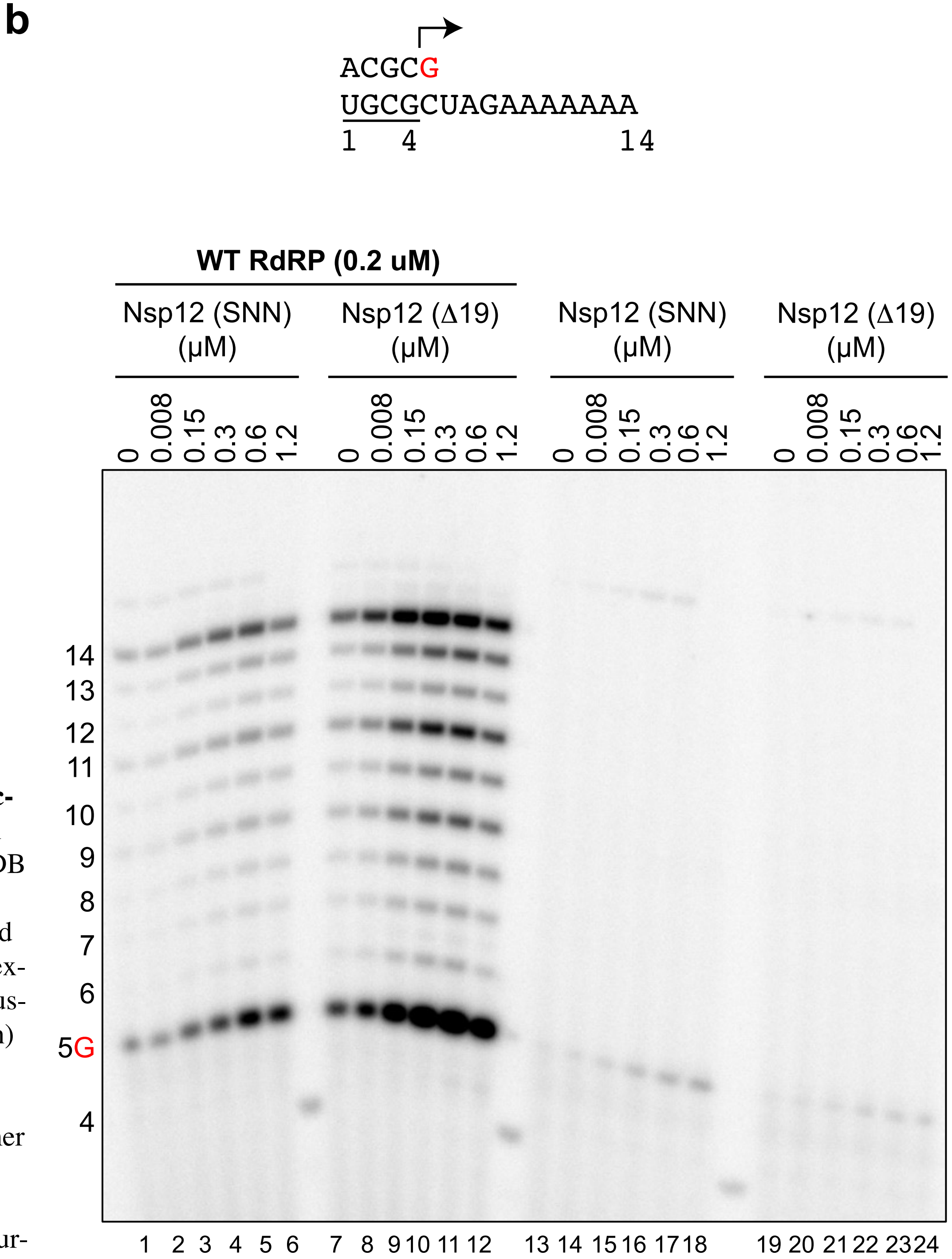

Supplementary Figure 10. Nsp12 [Δ19aa] encoded by GI.616 is functionally inactive. a. Depiction of location of the Nsp12(Δ19) deletion in the RNA-bound RNA-dependent RNA polymerase (RdRp) complex (PDB 7BV2). The deletion [575-594; shown in black] extends from the end of the fingers region (pink) to the beginning of the palm domain (green) and is predicted to impair binding to RNA. **b.** Wild-type (WT) RdRp complexes (Nsp7, Nsp8, and Nsp12) or mutant complexes containing the previously described Nsp12 (SNN, i.e. motif C mutant: SDD to SNN substitution) subunit or Nsp12 (Δ19) were expressed and purified from Baculovirus infected Sf-9 cells. These were used to assemble in vitro RNA synthesis reactions as previously described (PMID 32967965). Top: Briefly, a 4-mer primer (5'pACGC3') was extended with 0.1 μM ATP, CTP, UTP, in the presence of 0.1 μM [α-^{32}P]GTP. Bottom: Reaction products obtained in the presence or absence of WT RdRp complex were resolved on a denaturing 20% polyacrylamide gel. The Nsp12 (SNN) and Nsp12 (Δ19)RdRp complexes are significantly impaired for RNA synthesis (compare lanes 13-18 and 19-24 to lane 1).

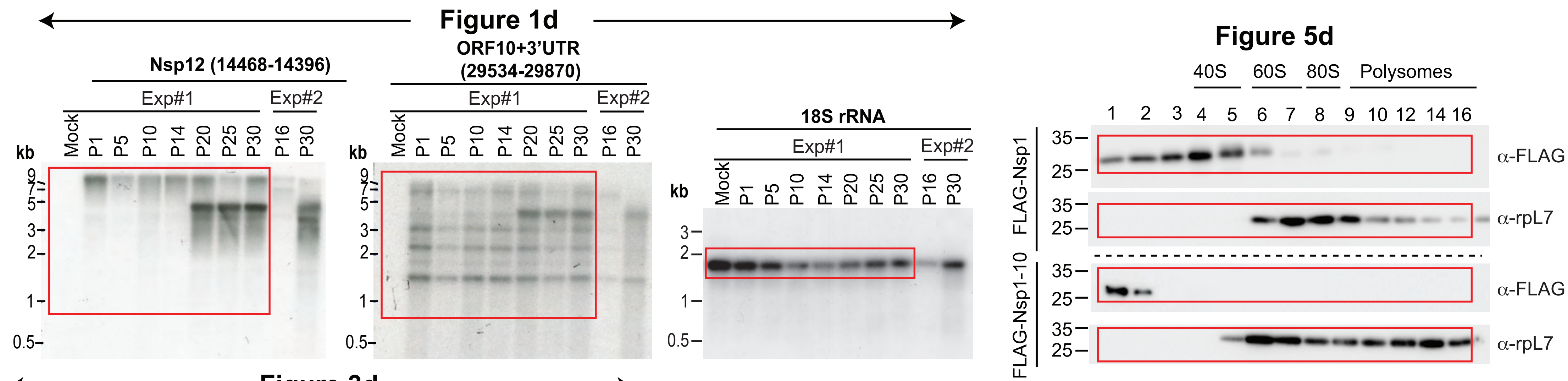

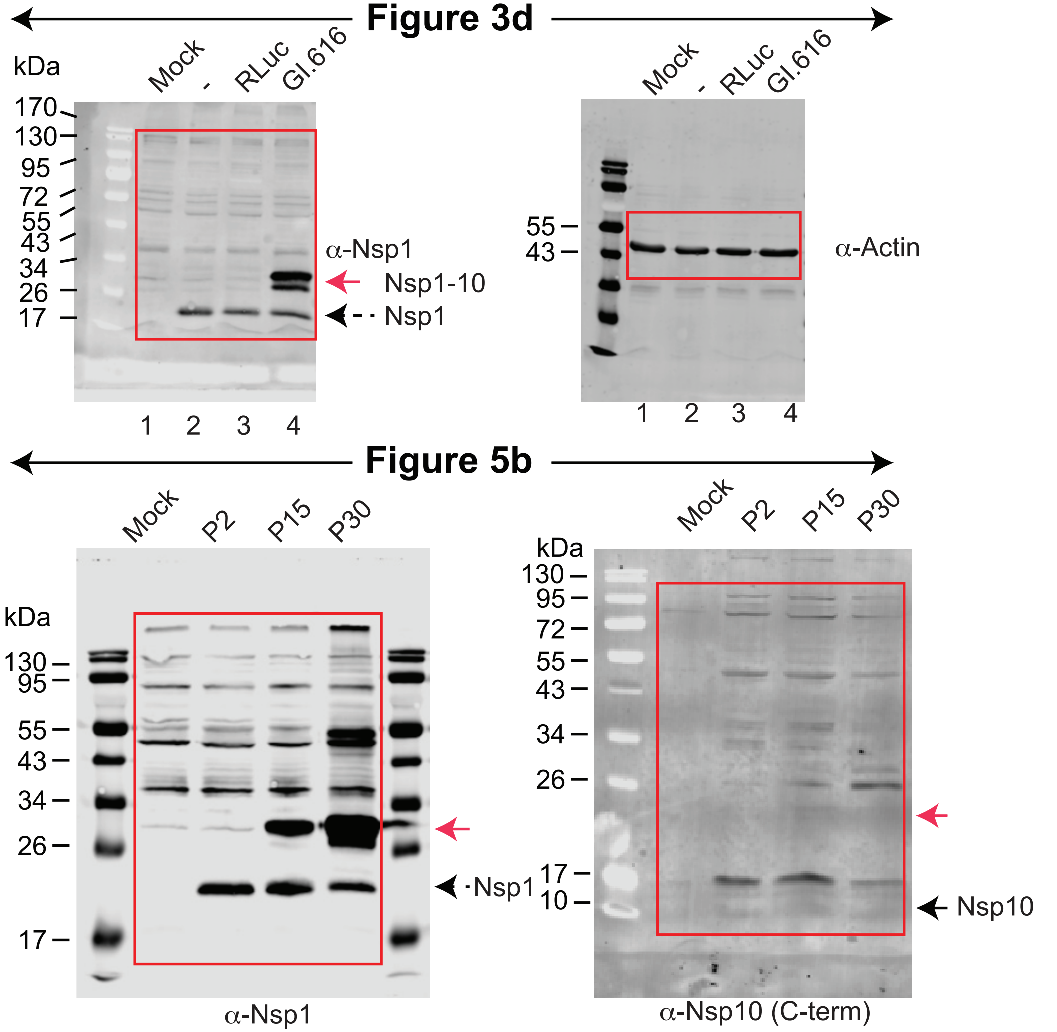

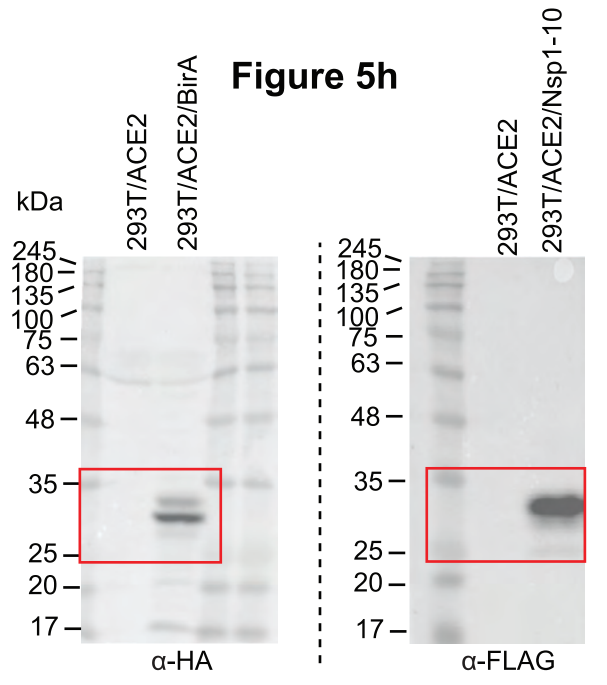

Supplementary Figure 11. Uncropped Western and Northern Blots. Portions of blots used to generate figures are indicated by red boxes. Related to Figs. 1d, 3d, 5b, 5d, and 5h.

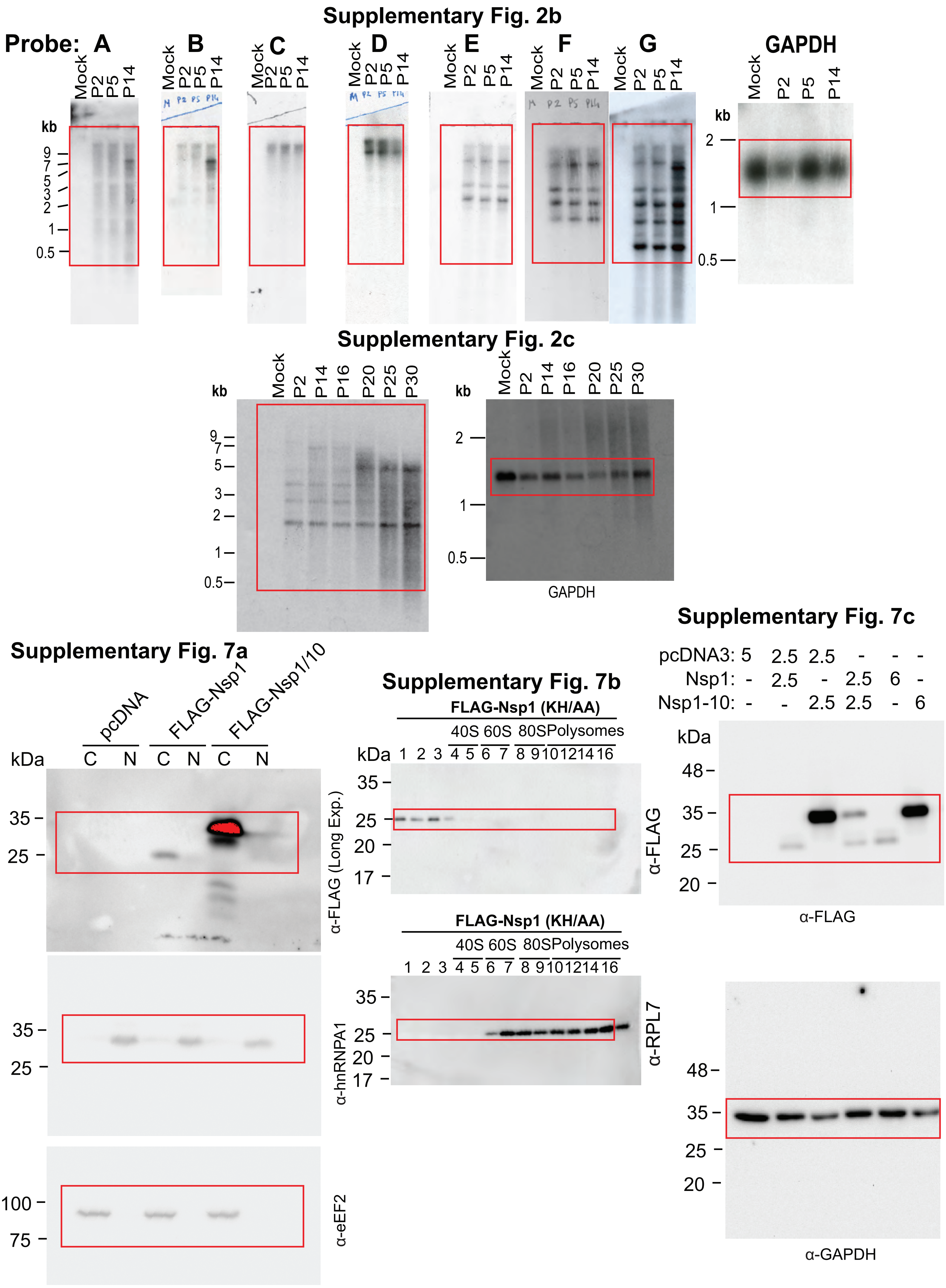

Supplementary Figure 12. Uncropped Western and Northern blots. Portions of blots used to generate figures are indicated by red boxes. Related to supplementary figs. 2b, 2c, 7a, 7b, and 7c.

SUPPLEMENTARY NOTE 1

Evolution of Naturally Arising SARS-CoV-2 Defective Interfering Particles

Samer Girgis[1, †] Zaikun Xu[2, †], Spyros Oikonomopoulos[3], Alla D. Fedorova[4,5] Egor P. Tchesnokov[6], Calvin J. Gordon[6], T. Martin Schmeing[1], Matthias Götte[6], Nahum Sonenberg[1,7], Pavel V. Baranov[4], Jiannis Ragoussis[3,8,9], Tom C. Hobman[2,6,10,11,‡] and Jerry Pelletier[1,7,12, ‡]

[1]Dept. Biochemistry, McGill University, Montreal, Quebec, Canada, H3G 1Y6; [2]Dept. Cell Biology, U Alberta, Edmonton, Alberta, Canada, T6G 2H7; [3]McGill Genome Centre, McGill University, Montreal, Quebec, Canada; [4]School of Biochemistry and Cell Biology, University College Cork, Cork, Ireland; [5]SFI Centre for Research Training in Genomics Data Science, University College Cork, Cork, Ireland; [6]Dept Medical Microbiology and Immunology, U Alberta, Edmonton, Alberta, Canada, T6G 2E1;[7]Rosalind and Morris Goodman Cancer Institute, Montreal, Quebec, Canada H3A 1A3; [8]Department of Human Genetics, McGill University, Montreal, Quebec, Canada; [9]Dept Bioengineering, McGill University, Montreal, Quebec, Canada; [10]Li Ka Shing Institute of Virology, U Alberta, Edmonton, Alberta, Canada T6G 2E1; [11]Women & Children’s Health Research Institute, U Alberta, Edmonton, Alberta, Canada, T6G 1C9; [12]Dept Oncology, McGill University, Montreal, Quebec, Canada H3A 1G5.

Supplementary Note 1 – Pipeline for Nanopore Sequence Analysis

Basecalling the raw signal of the direct RNA nanopore reads

The raw data of the direct RNA nanopore sequenced reads were "high quality" basecalled with the guppy basecaller v5.0.11 (Oxford Nanopore Technologies) using the following parameters:

--flowcell FLO-MIN106

--kit SQK-RNA002

--recursive

--records_per_fastq 4000

--fast5_out

--disable_pings

--verbose_logs

--min_qscore 7

--num_callers 3

--gpu_runners_per_device 8

--chunks_per_runner 48

--cpu_threads_per_caller 1

--chunk_size 100

--device cuda:0

Only nanopore reads with average basecalling quality of more than 7 were used in downstream analysis.

"Defective viral genomes / Viral transcripts" model reconstruction pipeline from the direct-RNA nanopore data

The FASTQ files of the "passed" nanopore reads (average high-quality basecalling value >=7) were processed through the following pipeline to be able to reconstruct the defective viral genomes and the viral transcripts. The pipeline took some elements from the analysis pipeline presented in the work of Kim et al. (Kim et al., 2020). The pipeline had the following steps that we ran sequentially:

Step 1. The nanopore reads were aligned with minimap2 (Li, 2021) (options: -k 13 -ax splice -N 32 -u n) on a file containing the following genomes:

- Chlorocebus sabaeus (Vervet-AGM; assembly number: ChlSab1.1; GCA_000409795.2). The genome was downloaded from Ensembl.
- Severe acute respiratory syndrome coronavirus 2 isolate Wuhan-Hu-1, complete genome (NC_045512.2). The genome was downloaded from NCBI.
- Human ribosomal DNA complete repeating unit (U13369.1). The genome was downloaded from NCBI.

The reads that aligned only on the NC_045512.2 genome were isolated and then realigned on the same NC_045512.2 genome with minimap2 (Li, 2021) for visualization purpose with different alignment parameters (options: -a -k 8 -w1–splice -g 30000 -G 30000 -A1 -B2 -O2,24 -E1,0 -C0 -z 400,200–no-end-flt–junc-bonus=100 -F 40000 -N 32–splice-flank=no–max-chain-skip=40 -un–junc-bed=FILE -p 0.7) .

Step 2. The selected virus specific nanopore reads from Step 1 were processed through the following three pipelines : PinFish (https://github.com/nanoporetech/pinfish) , CARNAC-LR ((Marchet et al., 2019) ; https://github.com/kamimrcht/CARNAC-LR) and RATTLE ((de la Rubia et al., 2022) ; https://github.com/comprna/RATTLE).

Step 2a

In the case of the PinFish pipeline the reads were processed as per the instructions of the manual with the following modifications. We changed the reconstruction parameters found in the parameters file (file with the ".yml" extension) as follows:

- No pychopper was performed on the reads.
  - ✓ pychopper: False
- The input genome that was used:
  - ✓ genome_fasta: NC_045512.2
  - ✓ genome_annot: The gtf file was downloaded from the UCSC genome browser site and it was the "SARS-CoV-2 Transcriptome tracks from the Kim lab" data from the SARS-CoV-2 Jan. 2020 (NC_045512.2) (wuhCor1) genome assembly.
- direct RNA reads in FASTQ format, the reads that were selected from Step 1.
- Options passed to minimap2 during indexing and required for stranded data the ones where pychopper filtered them:
  - ✓ minimap_index_opts: "-k14"
  - ✓ minimap2_opts: "-uf"
- Minimum mapping quality:
  - ✓ minimum_mapping_quality: 0
- spliced_bam2gff options:
  - ✓ spliced_bam2gff_opts: "-s"
  - ✓ spliced_bam2gff_opts_pol: "-s"
- Extra parameters:
  - ✓ minimum_cluster_size: 5
  - ✓ minimum_isoform_percent: 0.00001
  - ✓ exon_boundary_tolerance: 10
  - ✓ terminal_exon_boundary_tolerance: 30
- Extra options passed to minimap2 when mapping polished reads:

  - ✓ minimap2_opts_polished: "-uf"
- Options passed to spliced_bam2gff when converting alignments of polished reads:
  - ✓ minimap2_opts_polished: "-uf"
- Options passed for gene model creation:
  - ✓ Internal exon boundary tolerance (“collapse_internal_tol”): 5
  - ✓ Five prime boundary tolerance (“collapse_five_tol”): 30
  - ✓ Three prime boundary tolerance (“collapse_three_tol”): 30

Step 2b

For the CARNAC-LR due to the fact that the reconstruction was slow, we randomly sampled 30,000 reads (from the selected virus specific nanopore reads of Step 1) and we used them for the reconstruction process. Initially the pipeline aligned the reads against each other with minimap2 (options: -x ava-ont). The output of the read pairwise alignment was a PAF file with the following fields (the table was copied from: https://lh3.github.io/minimap2/minimap2.html):

Col	Type	Description
1	string	Query sequence name
2	int	Query sequence length
3	int	Query start coordinate (0-based)
4	int	Query end coordinate (0-based)
5	char	‘+’ if query/target on the same strand; ‘-’ if opposite
6	string	Target sequence name
7	int	Target sequence length
8	int	Target start coordinate on the original strand
9	int	Target end coordinate on the original strand
10	int	Number of matching bases in the mapping
11	int	Number bases, including gaps, in the mapping
12	int	Mapping quality (0-255 with 255 for missing)

We used the fields in the above table to create the following summary statistics for every read:

- ❖ 1st percentage *(explanation: where on the query read length, the alignment of the query sequence on the target sequence, started. The value was expressed as fraction (in percentage) of the query read length)*:

[“Query start coordinate (0-based)” field / “Query sequence length” field] * 100

- 2nd percentage *(explanation: where on the query read length, the alignment of the query sequence on the target sequence, ended. The value was expressed as fraction (in percentage) of the query read length)*:

  [“Query end coordinate (0-based)” field / “Query sequence length” field] * 100

- 3rd percentage *(explanation: where on the target read length, the alignment of the target sequence on the query sequence, started. The value was expressed as fraction (in percentage) of the target read length)*:

  [“Target start coordinate on the original strand” field / “Target sequence length” field] * 100

- 4th percentage *(explanation: where on the target read length, the alignment of the target sequence on the query sequence, ended. The value was expressed as fraction (in percentage) of the target read length)*:

  [“Target end coordinate on the original strand” field /“Target sequence length” field] * 100

Then the filtering was done as follows:

- ✓ 1st percentage <= 10 *(explanation: the alignment of the query sequence on the target sequence, started in the first 10% length of the query read length)*
- ✓ 2nd percentage >=90 *(explanation: the alignment of the query sequence on the target sequence, ended in the last 90% length of the query read length)*
- ✓ 3rd percentage <= 10 *(explanation: the alignment of the target sequence on the query sequence, started in the first 10% length of the target read length)*
- ✓ 4th percentage >=90 *(explanation: the alignment of the target sequence on the query sequence, ended in the last 90% length of the target read length)*
- ✓ and the field 5 of the PAF file (“*‘+’ if query/target on the same strand; ‘-’ if opposite”*) had the “+” sign, which practically meant that the query to target alignment happened on the same strand (neither the query nor the target sequence had to be the reverse complement of the sequenced read during the alignment)***.***

Then the selected lines from the PAF file were passed into the “paf_to CARNAC.py” script to produce a CARNAC-LR compatible object that was then passed to the CARNAC-LR script. The output was then fed into the “CARNAC_to_fasta” script. The output contained files with the individual clusters and each file had the FASTA sequence of the reads that were assigned to the specific cluster. These output files were then individually passed to the “muscle” program (v3.8.31; https://www.drive5.com/muscle/) for multiple sequence alignment. For every cluster, a consensus was built using the “msa” library in R (function: msaConcensusSequence; parameters: type=c("Biostrings", "upperlower"), thresh=c(80, 20), ignoreGaps=FALSE)). At the end, the gaps in the consensus sequences were removed and the final sequences were used as the sequence of the reconstructed models.

<u>Step 2c</u>

We also used the “RATTLE “ pipeline (https://github.com/comprna/RATTLE) with the following commands as per the manual:

rattle cluster -i virus_aligned_reads.fastq -t 31 --fastq --iso --rna
rattle cluster_summary -i virus_aligned_reads.fastq -c clusters.out --fastq
rattle extract_clusters -i virus_aligned_reads.fastq -c clusters.out -o clusters --fastq
rattle correct -i virus_aligned_reads.fastq -c clusters.out -t 31
rattle polish -i consensi.fq -t 31 –rna

where the “virus_aligned_reads.fastq” was provided as input and it was the selected virus specific nanopore reads from Step 1 and “consensi.fq” was the file with the FASTA sequence of the reconstructed models.

Step 3. The aim of this step was to take the reconstructed models from the three individual pipelines, to pool them together and to collapse similar models, that were found across multiple pipelines, into one consensus record. Models that were detected exclusively in one of the pipelines were kept as they were reported.

The FASTA files of the reconstructed “defective viral genomes / viral transcripts” models from all the above three pipelines of Step 2 were pooled together into a single file and were aligned on the NC_045512.2 genome using minimap2 (options: -k 13 -ax splice -N 32 -u n).

The SAM file of the aligned reads was then fed into the “tama_collapse.py” module from the “tama” software (https://github.com/GenomeRIK/tama) with the following parameters:

- ✓ Coverage (-c): 1
- ✓ Identity (-i): 70
- ✓ 5 prime threshold (-a): 30
- ✓ 3 prime threshold (-z): 30
- ✓ Exon/Splice junction threshold (-m): 10
- ✓ Variation coverage threshold (-vc): 1
- ✓ Capped flag (-x): capped

The output of “tama” was a “bed file” which practically had the exonic coordinates of the models (start, end) without any information for the sequence itself. To transform the exonic coordinates of the “bed file” into sequences we used the “getfasta” module from bedtools (https://bedtools.readthedocs.io/en/latest/content/tools/getfasta.html) where we provided as genome the NC_045512.2 one (options: -split -name).

Step 4. The Steps 1 to 3 were repeated separately for each dataset, from the individual viral infection passages.

Step 5. The aim of this step was to take the reconstructed models from the individual passages, to pool them together and to collapse similar models, that were found across multiple passages, into one consensus record. Models that were detected exclusively in one of the passages were

kept as they were. The logic was the same as the one presented in Step 3 but in this case, it applied across different viral passages rather than different pipelines.

The FASTA files of the reconstructed “defective viral genomes / viral transcripts” models from the all the different passages were pooled together into a single file and were aligned on the NC_045512.2 genome using minimap2 (options: -k 13 -ax splice -N 32 -u n).

The SAM file of the aligned reads was then fed into the “tama_collapse.py” module from the “tama” software with the parameters presented in Step 3.

The “bed file” output of tama was transformed into model sequences with the “getfasta” module from bedtools as presented in Step 3.

These sequences represented the reconstructed models that were present in the whole experiment independent of the viral passage number.

Step 6. The selected virus specific nanopore reads from Step 1 for the individual passages were then aligned on the complete list of reconstructed models from Step 5.

We used the FASTA file of the complete list of reconstructed models from Step 5 to create a refence index for the alignment. Then the virus specific reads from every passage were aligned on the reference index using minimap2 (options: --secondary=no -ax map-ont).

The output of the aligner in SAM format was then transformed in PAF format with the “htsbox samview“ module (https://github.com/lh3/htsbox) . The PAF format fields (see Step 2b) are then used to calculate the alignment identity of the reads as follows:

- Identity = (“Number of matching bases in the mapping” field) / ((“Query start coordinate” field) – (“Query end coordinate” field)))

We then kept the reads that only had identity value greater or equal to 0.8 as well as the filtering criteria that were already presented in step 2 namely:

- ✓ 1st percentage <= 10
- ✓ 2nd percentage >=90
- ✓ 3rd percentage <= 10
- ✓ 4th percentage >=90
- ✓ and the field 5 of the PAF file had the “+” sign.

The aligned reads that were left after the filtering, were used to calculate the abundance of the different models. The field from the PAF file with the names of the target models from the alignment (field 6 in the PAF file), was isolated and the abundance (number of records) of the individual models was calculated.

Only models with at least 50 assigned nanopore reads, in any one of the individual passages, were reported.

SUPPLEMENTARY NOTE 2

Evolution of Naturally Arising SARS-CoV-2 Defective Interfering Particles

Samer Girgis[1, †] Zaikun Xu[2, †], Spyros Oikonomopoulos[3], Alla D. Fedorova[4,5] Egor P. Tchesnokov[6], Calvin J. Gordon[6], T. Martin Schmeing[1], Matthias Götte[6], Nahum Sonenberg[1,7], Pavel V. Baranov[4], Jiannis Ragoussis[3,8,9], Tom C. Hobman[2,6,10,11,‡] and Jerry Pelletier[1,7,12, ‡]

[1]Dept. Biochemistry, McGill University, Montreal, Quebec, Canada, H3G 1Y6; [2]Dept. Cell Biology, U Alberta, Edmonton, Alberta, Canada, T6G 2H7; [3]McGill Genome Centre, McGill University, Montreal, Quebec, Canada; [4]School of Biochemistry and Cell Biology, University College Cork, Cork, Ireland; [5]SFI Centre for Research Training in Genomics Data Science, University College Cork, Cork, Ireland; [6]Dept Medical Microbiology and Immunology, U Alberta, Edmonton, Alberta, Canada, T6G 2E1;[7]Rosalind and Morris Goodman Cancer Institute, Montreal, Quebec, Canada H3A 1A3; [8]Department of Human Genetics, McGill University, Montreal, Quebec, Canada; [9]Dept Bioengineering, McGill University, Montreal, Quebec, Canada; [10]Li Ka Shing Institute of Virology, U Alberta, Edmonton, Alberta, Canada T6G 2E1; [11]Women & Children's Health Research Institute, U Alberta, Edmonton, Alberta, Canada, T6G 1C9; [12]Dept Oncology, McGill University, Montreal, Quebec, Canada H3A 1G5.

Summary statistics of the direct RNA nanopore runs

We provide Supplementary Data 4 with the summary statistics (number of reads sequenced, number of reads aligned etc) of the direct RNA nanopore runs. We also provide additional Supplementary Data 5 as a .zip folder whose content corresponds to the individual columns presented in the "summary statistics" table (to understand the contents of every file see where the "filename" is found in the corresponding columns). The columns in the table are as follows:

- **number of sequenced direct RNA nanopore reads→** number of direct RNA nanopore reads sequenced per individual passage.
- **unaligned reads→** number of direct RNA nanopore reads not aligning in either the host or the viral genome (this result is coming from "**Step 1"** of the "model reconstruction pipeline" of the previous section).
- **number of reads aligning on the host genome→** number of direct RNA nanopore reads aligning on the host genome (this result is coming from "**Step 1"** of the "model reconstruction pipeline" of the previous section).
- **number of reads aligning on the viral genome→** number of direct RNA nanopore reads aligning on the viral genome (this result is coming from "**Step 1"** of the "model reconstruction pipeline" of the previous section).
- **% of unaligned reads→** unaligned reads as a percentage number.
- **% of reads aligning on the host genome→** reads aligning on the host genome as a percentage number.
- **% of reads aligning on the viral genome→** reads aligning on the viral genome as a percentage number.

✓ ***We note here the following. The files corresponding to the four bullet points below are intermediate files of the pipeline. These files are not presented (used) in the manuscript and are only provided for the interested reader who would like to see the output of the intermediate steps of our pipeline. These files can be found in the folders "Pipeline_intermediate_files_experiment1" and "Pipeline_intermediate_files_experiment2" in the provided Supplementary Data 5.***

- **Number of RNA models from CARNAC-LR→** Reconstructed models for the indicated sample from the CARNAC-LR pipeline. One file is provided ("CARNACLR_RNA_models_reconstruction_experiment_*_passage_*.fasta"). This fasta file contains the sequence of the models. For each record the name is presented as follows, for example ">cluster28.fasta:2150" with "cluster28.fasta" the name of the model and "2150" the number of reads used to build it. The consensus is built from the sequence of the sequenced reads (this result is coming from "**Step 2b"** of the "model reconstruction pipeline" of the previous section).
- **Number of RNA models from PinFish→**Reconstructed models for the indicated sample from the PinFish pipeline. One file is provided ("PINFISH_RNA_models_reconstruction_experiment_*_passage_*.fasta"). This

fasta file contains the sequence of the models. For each record the name is presented as follows, for example “77c5b67e-4df4-4cb3-9ed1-aa5d2c0ab984|7” with “77c5b67e-4df4-4cb3-9ed1-aa5d2c0ab984” the name of the model and “7” the number of reads used to build it. The consensus is built from the sequence of the sequenced reads (this result is coming from “**Step 2a”** of the “model reconstruction pipeline” of the previous section).

- **Number of RNA models from RATTLE→**Reconstructed models for the indicated sample from the RATTLE pipeline. One file is provided (“RATTLE_RNA_models_reconstruction_experiment_*_passage_*.fasta”). This fasta file contains the sequence of the models. For each record the name is presented as follows, for example “>cluster_0 reads=2 total_reads=413713” with “cluster_0” the name of the model and “413713” the number of reads used to build it. The consensus is built from the sequence of the sequenced reads (this result is coming from “**Step 2c”** of the “model reconstruction pipeline” of the previous section).
- **Number of collapsed RNA models from PinFish & CARNAC-LR & RATTLE→** Consensus reconstructed models for the same **passage** across the different reconstructed pipelines models. The models from the “CARNAC-LR”,” PINFISH” and ”RATTLE” pipelines were used as an input into the “tama_collapse” module and common models between the different pipelines were collapsed into one record (not common models were left as is) for the indicated passage of the indicated experiment (the black brackets and the directional black arrows show which files were used to produce these set of files). The main output of the “tama_collapse” as presented in their manual (https://github.com/GenomeRIK/tama/wiki/Tama-Collapse) is a bed12 file with the coordinates of the reconstructed models, the “collapsed_RNA_models_reconstruction_experiment_*_passage_*.bed12” file we provide here. No fasta file with a consensus sequence per model is provided by default from ”tama_collapse”**.** Nevertheless we provide a fasta file for the collapsed models (“collapsed_RNA_models_reconstruction_experiment_*_passage_*.fasta”) that was created by using the bed12 file to extract the sequence of the models from the SARS-CoV-2 “NC_045512.2” genome. If the reader wants consensus sequences from the sequence of the nanopore reads, he needs to use the fasta files from the individual assemblers provided above. The “*collapsed_RNA_models_reconstruction_trans_read_experiment_*_passage_*. bed” file provided for each passage is the “prefix_trans_read.bed” from the “tama_collapse” module (https://github.com/GenomeRIK/tama/wiki/Tama-Collapse). The 4th column is of interest as it contains the relationship between original models and collapsed RNA models. The 1st subfield (before the first “;”) in the 4th column shows the RNA model ID from the collapsed list of RNA models (we call them “Intermediate collapsed IDs”) and the 2nd subfield (after the first ”;”) in the 4th column shows the RNA model ID from the outputs of either the “CARNAC-LR” or ” PINFISH” or ”RATTLE” pipelines (we call them “pipeline IDs”). Each line has an association between an “Intermediate collapsed ID” and a “pipeline ID”. “Pipelines IDs” associated with the same “Intermediate collapsed ID”

means that all these “Pipelines IDs” were collapsed into the same “Intermediate collapsed ID” representation. All these three files that we provide here, are coming from “**Step 3”** of the “model reconstruction pipeline” of the previous section.

✓ ***We note here the following. The files corresponding to the bullet point below are the ones presented (used) in the manuscript. The general reader need only download these files. These files can be found in the folders “Files_used_for_the_analysis_of_the_manuscript_experiment1” and “Files_used_for_the_analysis_of_the_manuscript_experiment2” the in provided Supplementary Data 5.***

- **Number of collapsed RNA models across all passages of experiment 1 (or experiment 2) →** Consensus reconstructed models for the same **experiment** across the models from the different passages of the same experiment. The models (fasta files) from the “tama_collapse” output of the different passages were used as an input into a new round of collapsing, using again the “tama_collapse” module (the black brackets and the directional black arrows show which files were used to produce these set of files). As before, common models between the different passages of the same experiment were collapsed into one record (not common models were left as is). Again, the main output of the “tama_collapse” is a bed12 file with the coordinates of the reconstructed models. We use this bed12 file to extract the sequence of the models from the SARS-CoV-2 “NC_045512.2” genome. The files provided are the following ones (these files are coming from “**Step 5”** of the “model reconstruction pipeline” of the previous section):
    - “experiment_*_collapsed_RNA_models_reconstruction.bed12” : Genomic coordinates (“NC_045512.2” genome) of the final RNA models (we call them “Final collapsed IDs”) from either the experiment 1 or 2 .
    - “experiment_*_collapsed_RNA_models_reconstruction.fasta”:Fasta sequence of the final RNA models from either the experiment 1 or 2 .
    - “experiment_*_collapsed_RNA_models_reconstruction_trans_read.bed”: This file is the “prefix_trans_read.bed” from the Tama-Collapse module (https://github.com/GenomeRIK/tama/wiki/Tama-Collapse). The 4th column is of interest as it contains the relationship between the “Intermediate collapsed IDs” and the “Final collapsed IDs”. The 1st subfield (before the first “;”) in the 4th column is the record of a “Final collapsed ID” and the 2nd subfield (after the first ”;”) in the 4th column is the record of an “Intermediate collapsed ID”. Each line has an association between a “Final collapsed ID” and an “Intermediate collapsed ID”. “Intermediate collapsed IDs” associated with the same “Final collapsed ID” means that all these “Intermediate collapsed IDs” were collapsed into the same “Final collapsed ID” representation.

We are also providing the following files per each passage of a given experiment (these files are coming from "**Step 6"** of the "model reconstruction pipeline" of the previous section):

- "abundance_of_RNA_models_experiment_*_passage_*": For each experiment (experiment 1 or experiment 2) we take the "Final collapsed ID" models and we are realigning on their fasta sequence the individual nanopore reads for each one of the passages in order to find the abundance of a given RNA model in the passage of interest (the name of the file indicates to which passage the abundance corresponds to). The file has 2 columns. The first column is the number of nanopore reads that are aligning on the model name of the second column.
- "RNA_MODELS_WITH_COVERAGE_experiment_*_passage_*.pdf": This file contains the coverage of the nanopore reads for a given model. For every pair of pictures, the left picture indicates the alignment coordinates of the different segments of the individual RNA models and the right picture the coverage. A detailed description of these files is presented in the "Supplementary Figure 13".

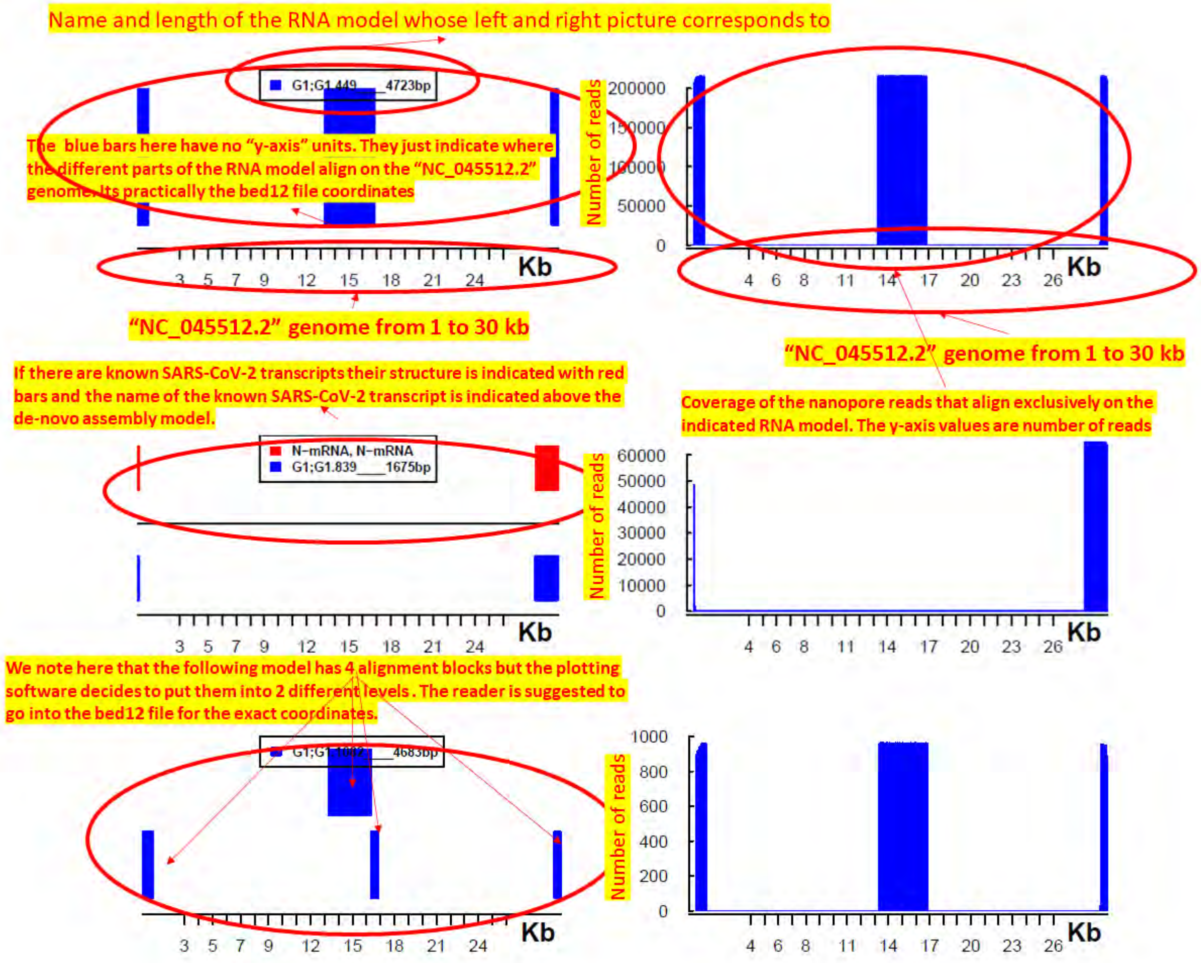

Supplementary Figure 13. Detailed description of the supplementary files named "RNA_MODELS_WITH_COVERAGE_experiment_*_passage_*.pdf" found in Supplementary Data 5.

SUPPLEMENTARY REFERENCES

de la Rubia, I., Srivastava, A., Xue, W., Indi, J.A., Carbonell-Sala, S., Lagarde, J., Albà, M.M., and Eyras, E. (2022). RATTLE: Reference-free reconstruction and quantification of transcriptomes from Nanopore sequencing. bioRxiv, 2020.2002.2008.939942.

Kim, D., Lee, J.Y., Yang, J.S., Kim, J.W., Kim, V.N., and Chang, H. (2020). The Architecture of SARS-CoV-2 Transcriptome. Cell *181*, 914-921 e910.

Li, H. (2021). New strategies to improve minimap2 alignment accuracy. Bioinformatics.

Marchet, C., Lecompte, L., Silva, C.D., Cruaud, C., Aury, J.M., Nicolas, J., and Peterlongo, P. (2019). De novo clustering of long reads by gene from transcriptomics data. Nucleic Acids Res *47*, e2.
