# Supplementary material for: Evolution of naturally arising SARS-CoV-2 defective interfering particles
Source: Commun Biol. 2022 Oct 27;5:1140. doi: 10.1038/s42003-022-04058-5 (PMC9610340; doi:10.1038/s42003-022-04058-5)
Supplement: Supplementary file 2 — Description of Additional Supplementary Files [file 42003_2022_4058_MOESM2_ESM.pdf]

Description of Additional Supplementary Files

File name: Supplementary Data 1
Description: Nucleotide sequence of transcript models corresponding to the indicated DVGs.

File name: Supplementary Data 2
Description: List of Primers Used in the Current Study.

File name: Supplementary Data 3
Description: Nucleotide sequence of constructs used in this study

File name: Supplementary Data 4
Description: Summary statistics table for the direct RNA nanopore runs

File name: Supplementary Data 5
Description: Intermediate and final files from the nanopore analysis pipeline

File name: Supplementary Data 6
Description: Numeric Source Data for Figures 1c, 2a, 3b, 5a, 5b, 6, 7b, 7e, 8, 9
