# Supplementary material for: Evolution of naturally arising SARS-CoV-2 defective interfering particles
Source: Commun Biol. 2022 Oct 27;5:1140. doi: 10.1038/s42003-022-04058-5 (PMC9610340; doi:10.1038/s42003-022-04058-5)
Supplement: Supplementary file 4 — Supplementary Data 2 [file 42003_2022_4058_MOESM4_ESM.pdf]

**Supplementary Table 2. List of Primers Used in the Current Study.**

| Primer Name | Sequence (5' – 3') | Comments |
|---|---|---|
| **SARS-CoV-2 3' End primer** | CTCCTAAGAAGCTATTAAAATCAC | Targets SARS-CoV-2 End. Targets nts 29841-29864 (NC 045512.2) |
| **USJ-1 qPCR primer** | CGAGCTTGGCACTGATCCTT | For amplification of GI.535, GI.50, GI.616 US junctions |
| **USJ-2 qPCR primer** | AACCCACAGGGTCATTAGCAC | For amplification of GI.535, GI.50, GI.616 US junctions |
| **DSJ-1 qPCR primer** | GGACTTCCTTGGAATGTAGTGC | For amplification of GI.464 DSJ |
| **DSJ-2 qPCR primer** | GTATTCAAGGCTCCCTCAGTTGC | For amplification of GI.464 DSJ |
| **DSJ-3 qPCR primer** | CCTAGACCACCACTTAACCG | For amplification of GI.535, GI.50, GI.616 DS junctions |
| **DSJ-4 qPCR Primer** | TCATCCAAATCTGCAGCAGG | For amplification of GI.535, GI.50, GI.616 DS junctions |
| **GAPDH-f qPCR primer** | GGTATCGTGGAAGGACTCAT | Used in RT-qPCRs for GAPDH quantification |
| **GAPDH-r qPCR primer** | GCAGGGATGATGTTCTGGAG | Used in RT-qPCRs for GAPDH quantification |
| **gRNA3000-f qPCR primer** | AACAAGACGGCAGTGAGGAC | Used in RT-qPCRs for SARS-CoV-2 gRNA quantification. Targets nts: 3234-3253 (NC_045512.2) |
| **gRNA3000-r qPCR primer** | GCTTCTTCCACAATGTCTGCA | Used in RT-qPCRs for SARS-CoV-2 gRNA quantification. Targets nts: 3391-3411 (NC_045512.2) |
| **18S rRNA-f qPCR Primer** | GGCCCTGTAATTGGAATGAGTC | Used for quantification of 18S rRNA |
| **18S rRNA-r qPCR Primer** | CCAAGATCCAACTACGAGCTT | Used for quantification of 18S rRNA |
| **Renilla-f qPCR** | TTAGACGGCCTACCCTCTCC | Used for quantification of Renilla mRNA |
| **Renilla-r qPCR** | CCCTCGACAATAGCGTTGGA | Used for quantification of Renilla mRNA |
| **A1 Long-range PCR primer** | ATTACTGTCGTTGACAGGACACGAGTAA | Used in long-range amplification of GI.50 and GI.616. Targets nts: 143-170 (NC_045512.2) |
| **A2 Long-range PCR primer** | ATCACATGGGGATAGCACTACTAAA | Used in long-range amplification of GI.50 and GI.616. Targets nts: 29821-29845 (NC_045512.2) |
| **NarI-f** | AGTCCACAAGCACGGCGCCGATCTAAAGTCA | Used to clone pcDNA3.1_3xFLAG-Nsp1/10 |

| | | |
|---|---|---|
| **NarI/BamHI-r** | GATTGTTGTCAATGGCGCCGGATCCCTACTGAAGCATGGGTTCGCGGA | Used to clone pcDNA3.1_3xFLAG-Nsp1/10 |
| **Junction B1 Long-range PCR primer** | CAAGAAAACTGGAATGTACAAATACC | Used for long-range PCR of GI.464 and GI.384 |
| **Junction B2 Long-range PCR primer** | GCCGTCTTTGTTATACTTCATAGATG | Used for long-range PCR of GI.464 |
| **Junction C1 Long-range PCR primer** | ATGTTGTTCCTTGGGACAACGCCGAC | Used for long-range PCR GI.384 |
| **IFNL1-f qPCR Primer** | CGCCTTGGAAGAGTCACTCA | Used for quantification of IFNL1 mRNA |
| **IFNL1-f qPCR Primer** | GAAGCCTTAGGTCCCAATTC | Used for quantification of IFNL1 mRNA |
| **TNF-f qPCR Primer** | TGCACTTTGGAGTGATCGGC | Used for quantification of TNF mRNA |
| **TNF-r qPCR Primer** | CTTGTCACTTGGGGTTCGAGA | Used for quantification of TNF mRNA |
| **CCL5-f qPCR Primer** | CGTGCCCACATCAAGGAGTAT | Used for quantification of CCL5 mRNA |
| **CCL5-r qPCR Primer** | CACTTGGCGATTCTTTCGGG | Used for quantification of CCL5 mRNA |
| **CCL2-f qPCR Primer** | ACCTCCAGCATGAAAGTCTC | Used for quantification of CCL2 mRNA |
| **CCL2-r qPCR Primer** | AAGTGACGGGGGCATTGAT | Used for quantification of CCL2 mRNA |
| **ISG20-f qPCR Primer** | TGAGGGAGAGATCACCGATT | Used for quantification of ISG20 mRNA |
| **ISG20-r qPCR Primer** | TAGCGGCTCATGTCCTCTTT | Used for quantification of ISG20 mRNA |

| | | |
|---|---|---|
| **ISG54-f qPCR Primer** | GGTCTTTTCAGCGTTTATTGGG | Used for quantification of ISG54 mRNA |
| **ISG54-r qPCR Primer** | TGCCGTAGGCTGCTCTCCA | Used for quantification of ISG54 mRNA |
| **CXCL10-f qPCR Primer** | GTGGCATTCAAGGAATACCTC | Used for quantification of CXCL10 mRNA |
| **CXCL10-r qPCR Primer** | GCCTTAGATTCTGGATTCAGCA | Used for quantification of CXCL10 mRNA |
| **TLR7-f qPCR Primer** | TTGGCACCTCTCATGCTCTG | Used for quantification of TLR7 mRNA |
| **TLR7-r qPCR Primer** | GTGTCCACACTGGAAACATCATT | Used for quantification of TLR7 mRNA |
| **TLR8-f qPCR Primer** | TTTCTCTTCTCGGCCACCTC | Used for quantification of TLR8 mRNA |
| **TLR8-r qPCR Primer** | TCAGCATTGACGACTGAAGGA | Used for quantification of TLR8 mRNA |
| **RIG-1-f qPCR Primer** | GGCTGCCACGCTTTTTCTC | Used for quantification of RIG-1 mRNA |
| **RIG-1-r qPCR Primer** | CAATGGCTTCATAAAGTCCAGAAT | Used for quantification of RIG-1 mRNA |
| **IKBKE-f qPCR Primer** | GGAAGAGCCTTTGCCTGACT | Used for quantification of IKBKE mRNA |
| **IKBKE-r qPCR Primer** | GGTTGTGCTCTGCATCTCCT | Used for quantification of IKBKE mRNA |
| **IFIT1-f qPCR Primer** | ACAGCTGCCTAATTCACAGC | Used for quantification of IFIT1 mRNA |
| **IFIT1-r qPCR Primer** | GGCCTTTCAGGTGTTTCACA | Used for quantification of IFIT1 mRNA |
| **IFIH1 (MDA5)-f qPCR Primer** | ACGGAGGAGGAACTGTTGAC | Used for quantification of IFIH1 mRNA |

| | | |
|---|---|---|
| **IFIH1 (MDA5)-r qPCR Primer** | CCAGAAATGCAGAGAACCAG | Used for quantification of IFIH1 mRNA |
