# Supplementary material for: Evolution of naturally arising SARS-CoV-2 defective interfering particles
Source: Commun Biol. 2022 Oct 27;5:1140. doi: 10.1038/s42003-022-04058-5 (PMC9610340; doi:10.1038/s42003-022-04058-5)
Supplement: Supplementary file 6 — Supplementary Data 4 [file 42003_2022_4058_MOESM6_ESM.pdf]

The files indicated below are intermediate files of the pipeline. <u>These files are not presented (used) in the manuscript</u> and are only provided for the interested reader that wants to see the output of the intermediate steps of our pipeline. These files can be found in the folders "Pipeline_intermediate_files_experiment1" and "Pipeline_intermediate_files_experiment2" in the provided supplementary files.

<u>The files indicated below are the ones presented (used) in the manuscript</u>. **The general reader should download only these files.** These files can be found in the folders "Files_used_for_the_analysis_of_the_manuscript_experiment1" and "Files_used_for_the_analysis_of_the_manuscript_experiment2" in the provided supplementary files.

Sample name	number of sequenced direct RNA nanopore reads	unaligned reads	number of reads aligning on the host genome	number of reads aligning on the viral genome	% of unaligned reads	% of reads aligning on the host genome	% of reads aligning on the viral genome		Number of RNA models from CARNAC-LR	Number of RNA models from PinFish	Number of RNA models from RATTLE	Number of collapsed RNA models from PinFish & CARNAC-LR & RATTLE
experiment 1 passage 1	758,728	5,676	100,434	650,087	0.75	13.24	85.68	experiment 1 passage 1	1,547	1,338	163	1,368
experiment 1 passage 14	666,857	5,881	53,470	604,542	0.88	8.02	90.66	experiment 1 passage 14	4,091	1,071	232	1,541
experiment 1 passage 30	1,517,899	12,339	100,725	1,401,106	0.81	6.64	92.31	experiment 1 passage 30	1,549	2,588	285	2,488
experiment 2 passage 1	476,925	3,692	167,780	302,662	0.77	35.18	63.46	experiment 2 passage 1	1,651	492	90	638
experiment 2 passage 15	342,812	4,979	175,873	155,402	1.45	51.30	45.33	experiment 2 passage 15	2,372	503	102	725
experiment 2 passage 29	368,716	4,024	150,553	208,949	1.09	40.83	56.67	experiment 2 passage 29	2,175	1,884	150	2,322

	filenames:			
experiment 1 passage 1	CARNACLR_RNA_models_reconstruction_experiment_1_passage_1.fasta	PINFISH_RNA_models_reconstruction_experiment_1_passage_1.fasta	RATTLE_RNA_models_reconstruction_experiment_1_passage_1.fasta	collapsed_RNA_models_reconstruction_experiment_1_passage_1.fasta<br>collapsed_RNA_models_reconstruction_experiment_1_passage_1.bed12<br>collapsed_RNA_models_reconstruction_trans_read_experiment_1_passage_1.bed
experiment 1 passage 14	CARNACLR_RNA_models_reconstruction_experiment_1_passage_14.fasta	PINFISH_RNA_models_reconstruction_experiment_1_passage_14.fasta	RATTLE_RNA_models_reconstruction_experiment_1_passage_14.fasta	collapsed_RNA_models_reconstruction_experiment_1_passage_14.fasta<br>collapsed_RNA_models_reconstruction_experiment_1_passage_14.bed12<br>collapsed_RNA_models_reconstruction_trans_read_experiment_1_passage_14.bed
experiment 1 passage 30	CARNACLR_RNA_models_reconstruction_experiment_1_passage_30.fasta	PINFISH_RNA_models_reconstruction_experiment_1_passage_30.fasta	RATTLE_RNA_models_reconstruction_experiment_1_passage_30.fasta	collapsed_RNA_models_reconstruction_experiment_1_passage_30.fasta<br>collapsed_RNA_models_reconstruction_experiment_1_passage_30.bed12<br>collapsed_RNA_models_reconstruction_trans_read_experiment_1_passage_30.bed
experiment 2 passage 1	CARNACLR_RNA_models_reconstruction_experiment_2_passage_1.fasta	PINFISH_RNA_models_reconstruction_experiment_2_passage_1.fasta	RATTLE_RNA_models_reconstruction_experiment_2_passage_1.fasta	collapsed_RNA_models_reconstruction_experiment_2_passage_1.fasta<br>collapsed_RNA_models_reconstruction_experiment_2_passage_1.bed12<br>collapsed_RNA_models_reconstruction_trans_read_experiment_2_passage_1.bed
experiment 2 passage 15	CARNACLR_RNA_models_reconstruction_experiment_2_passage_15.fasta	PINFISH_RNA_models_reconstruction_experiment_2_passage_15.fasta	RATTLE_RNA_models_reconstruction_experiment_2_passage_15.fasta	collapsed_RNA_models_reconstruction_experiment_2_passage_15.fasta<br>collapsed_RNA_models_reconstruction_experiment_2_passage_15.bed12<br>collapsed_RNA_models_reconstruction_trans_read_experiment_2_passage_15.bed
experiment 2 passage 29	CARNACLR_RNA_models_reconstruction_experiment_2_passage_29.fasta	PINFISH_RNA_models_reconstruction_experiment_2_passage_29.fasta	RATTLE_RNA_models_reconstruction_experiment_2_passage_29.fasta	collapsed_RNA_models_reconstruction_experiment_2_passage_29.fasta<br>collapsed_RNA_models_reconstruction_experiment_2_passage_29.bed12<br>collapsed_RNA_models_reconstruction_trans_read_experiment_2_passage_29.bed

<u>Number of collapsed RNA models across all passages of experiment 1</u>

4,420

experiment_1_collapsed_RNA_models_reconstruction.fasta

experiment_1_collapsed_RNA_models_reconstruction.bed12

experiment_1_collapsed_RNA_models_reconstruction_trans_read.bed

abundance_of_RNA_models_experiment_1_passage_1

abundance_of_RNA_models_experiment_1_passage_14

abundance_of_RNA_models_experiment_1_passage_30

RNA_MODELS_WITH_COVERAGE_experiment_1_passage_1.pdf

RNA_MODELS_WITH_COVERAGE_experiment_1_passage_14.pdf

RNA_MODELS_WITH_COVERAGE_experiment_1_passage_30.pdf

<u>Number of colapsed RNA models across all passages of experiment 2</u>

3,430

experiment_2_collapsed_RNA_models_reconstruction.fasta

experiment_2_collapsed_RNA_models_reconstruction.bed12

experiment_2_collapsed_RNA_models_reconstruction_trans_read.bed

abundance_of_RNA_models_experiment_2_passage_1

abundance_of_RNA_models_experiment_2_passage_15

abundance_of_RNA_models_experiment_2_passage_29

RNA_MODELS_WITH_COVERAGE_experiment_2_passage_1.pdf

RNA_MODELS_WITH_COVERAGE_experiment_2_passage_15.pdf

RNA_MODELS_WITH_COVERAGE_experiment_2_passage_29.pdf
