# Supplementary figures and images for: Evolution of naturally arising SARS-CoV-2 defective interfering particles
Source: Commun Biol. 2022 Oct 27;5:1140. doi: 10.1038/s42003-022-04058-5 (PMC9610340; doi:10.1038/s42003-022-04058-5)

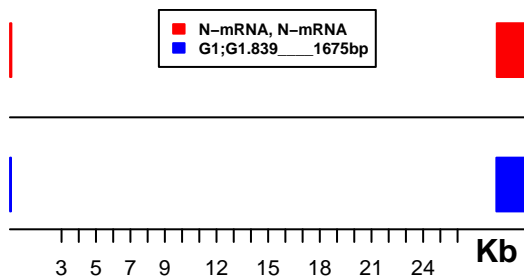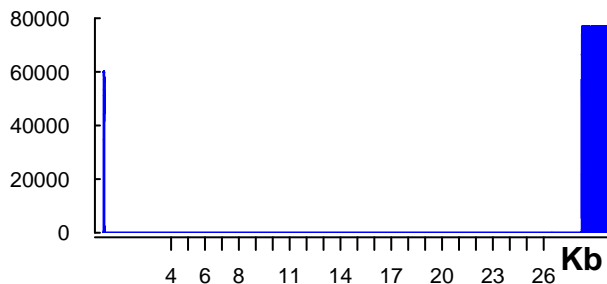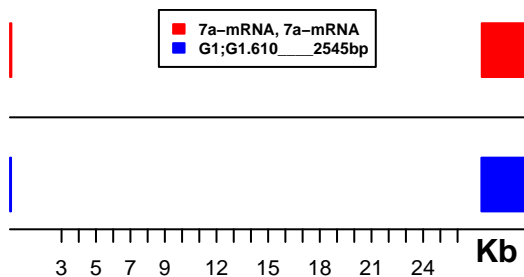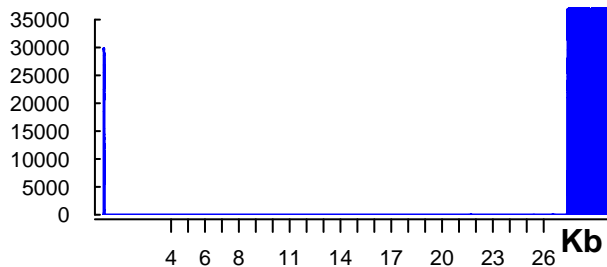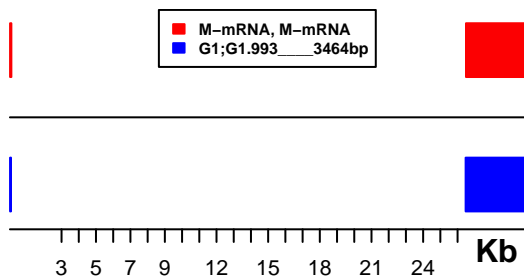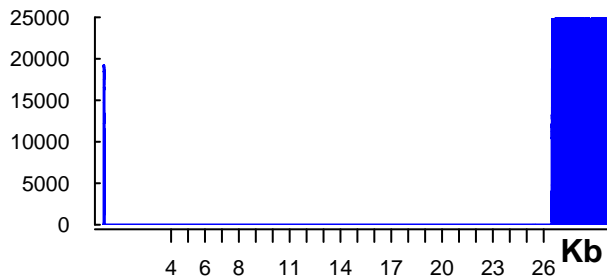

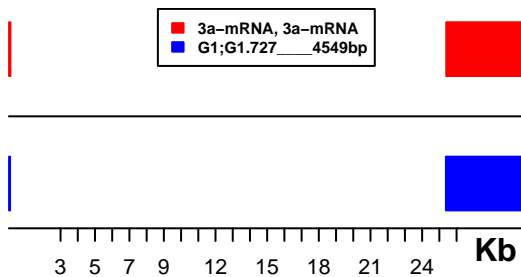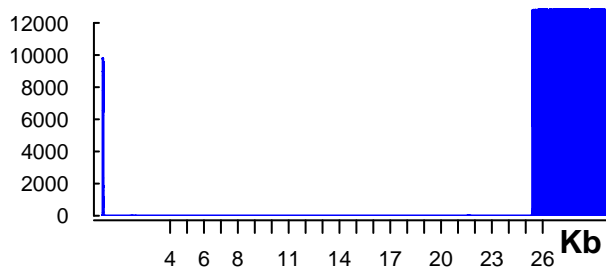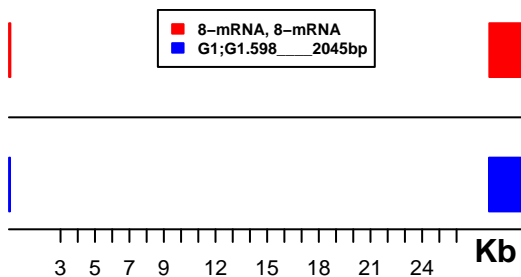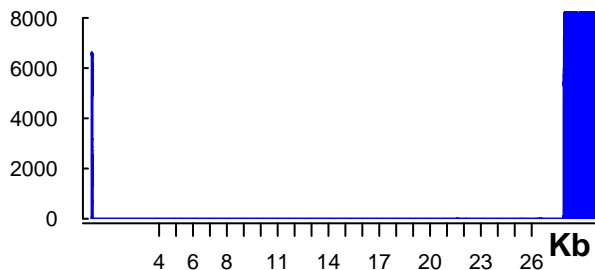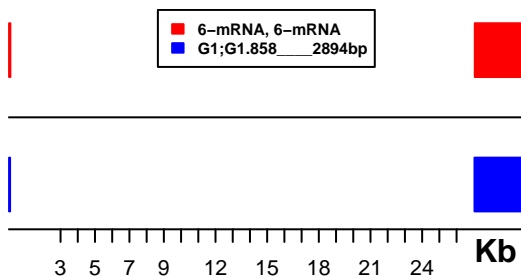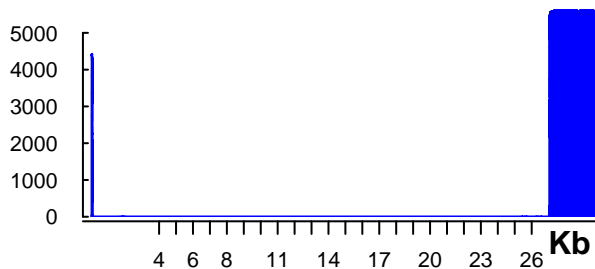

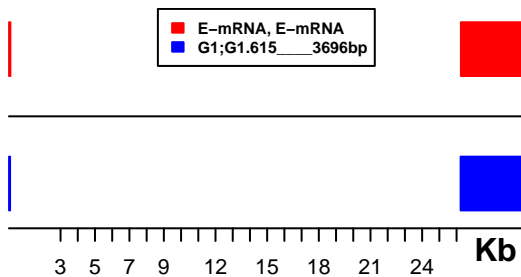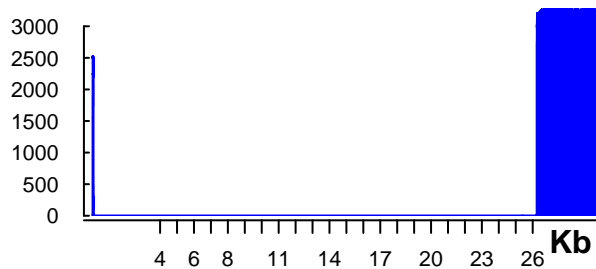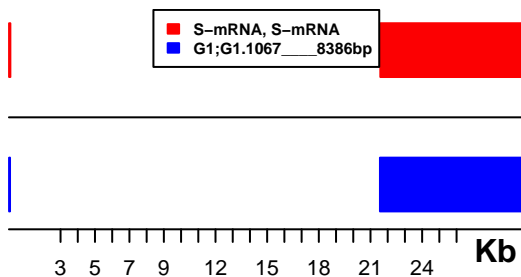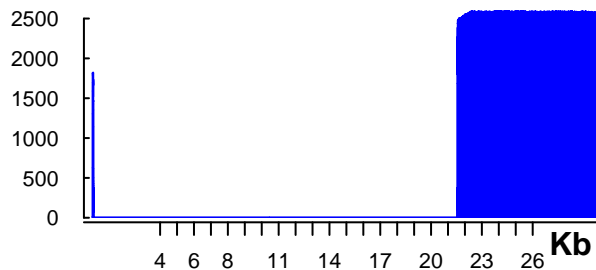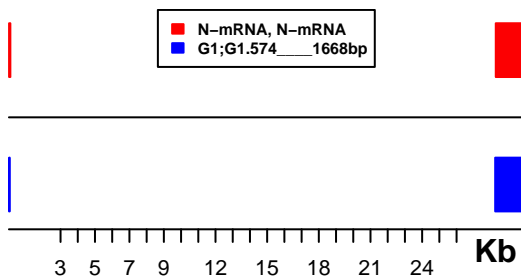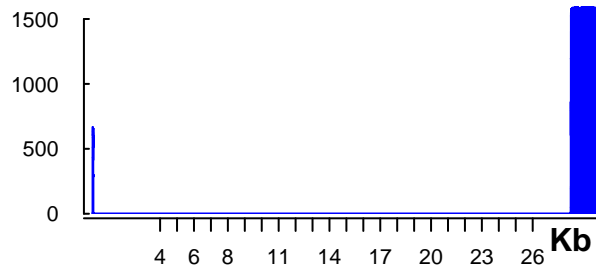

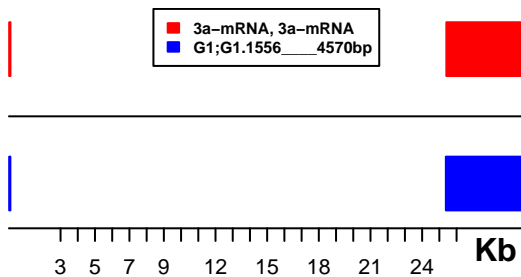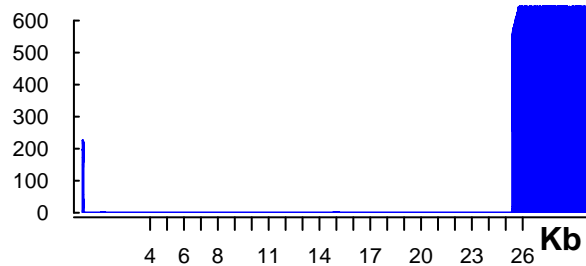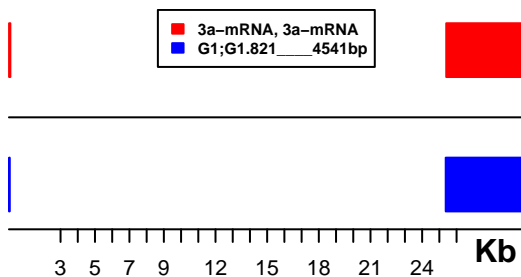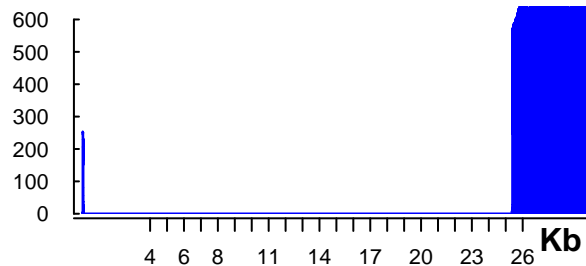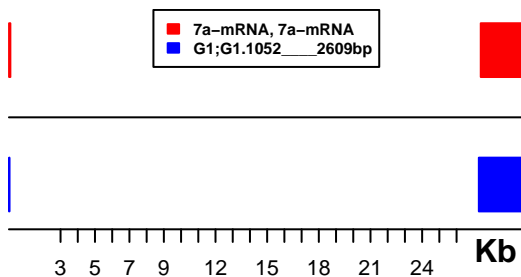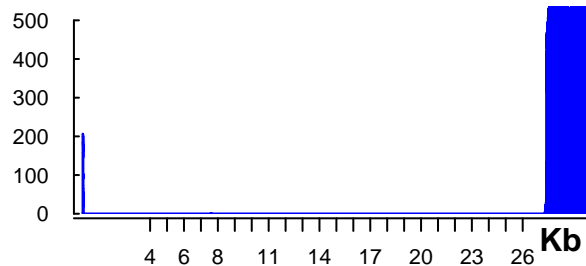

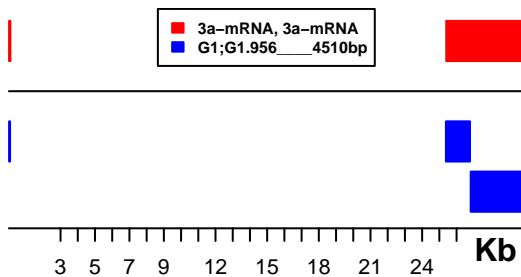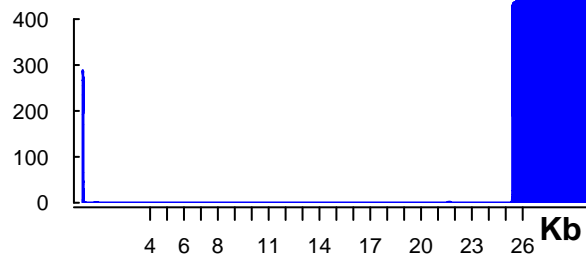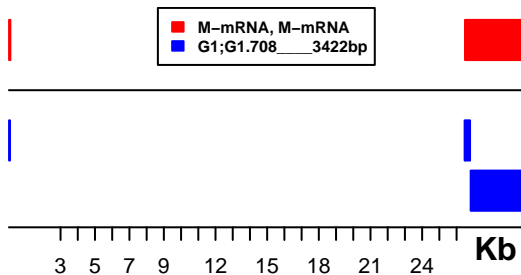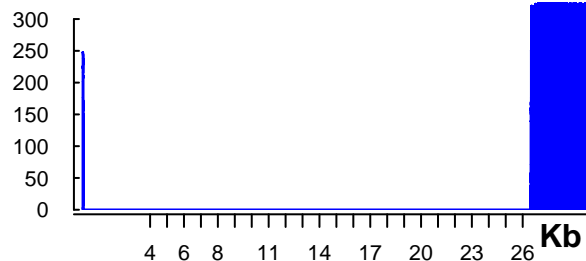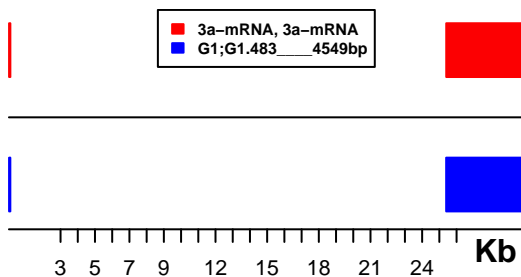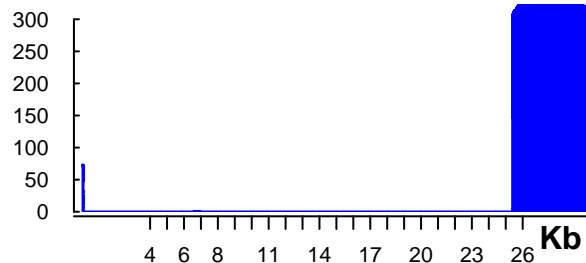

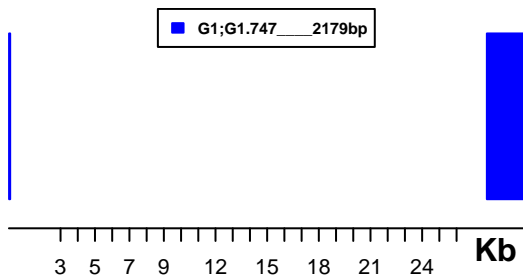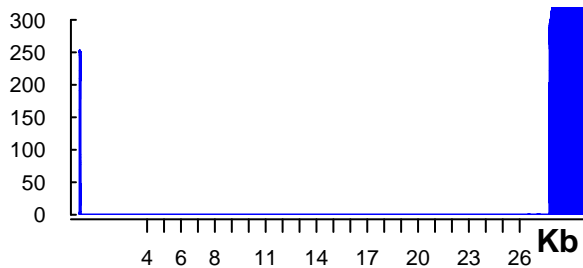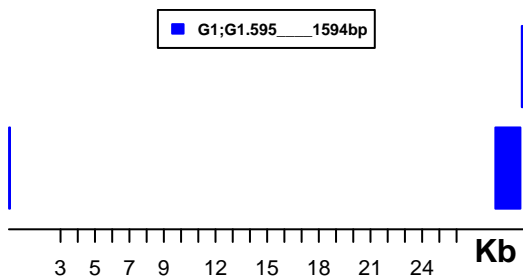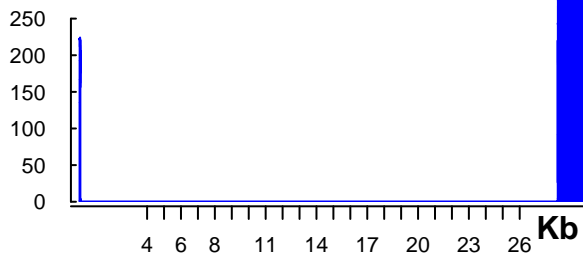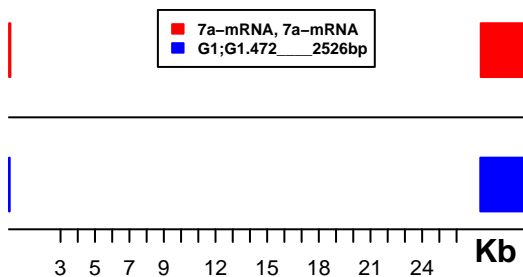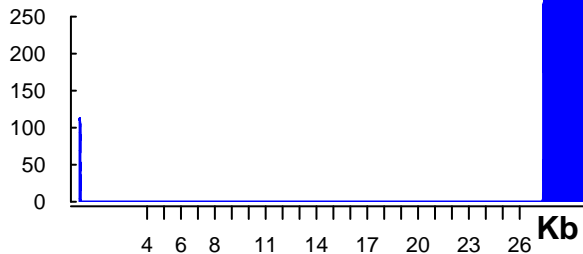

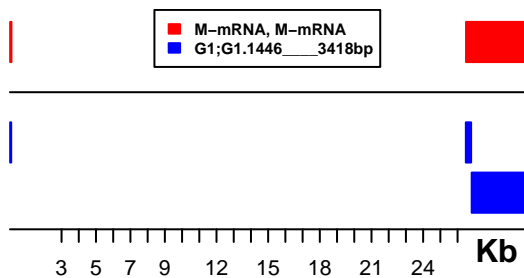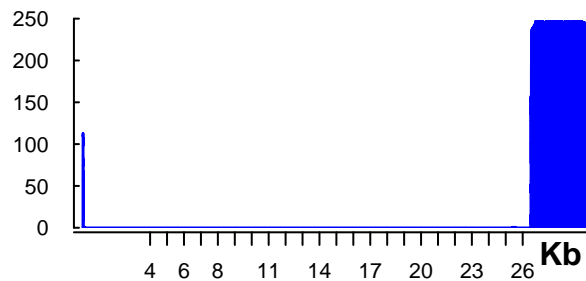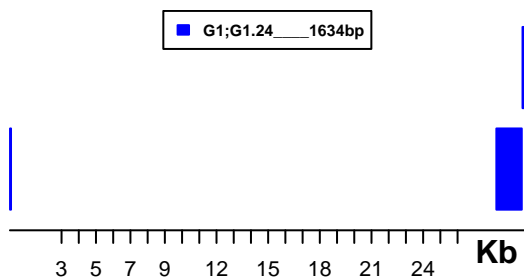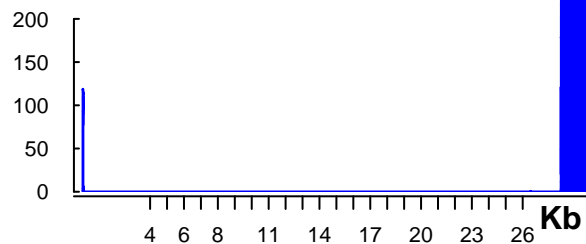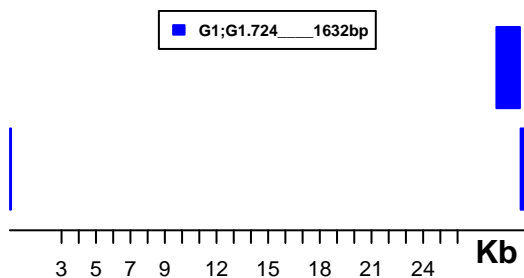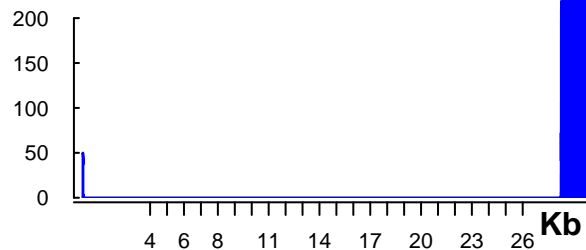

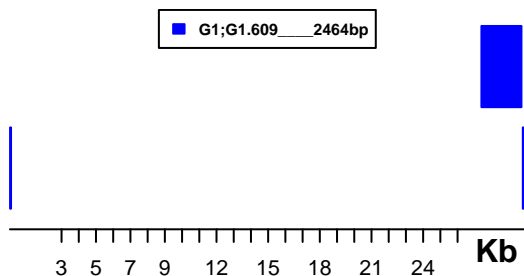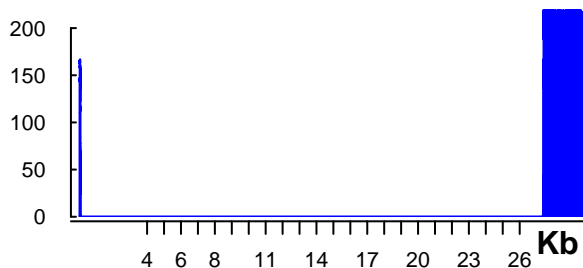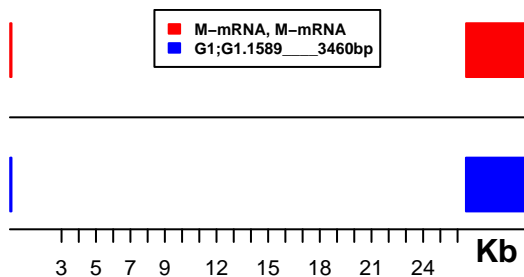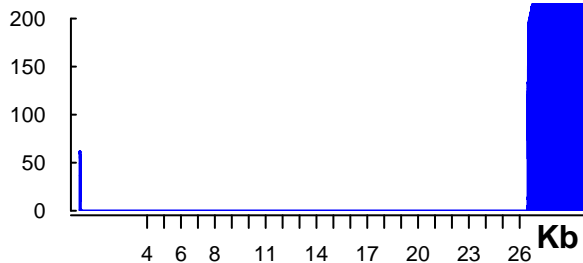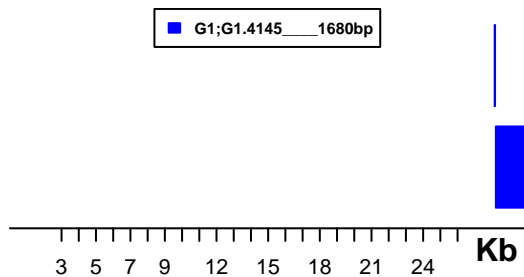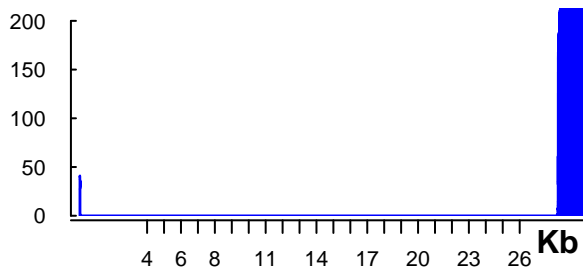

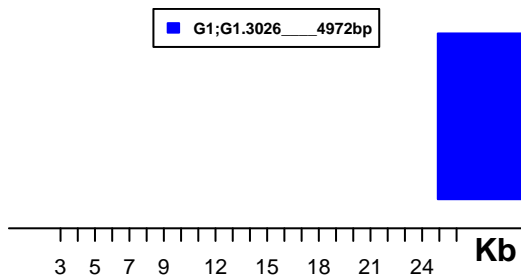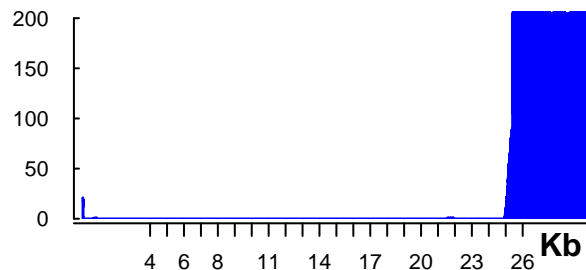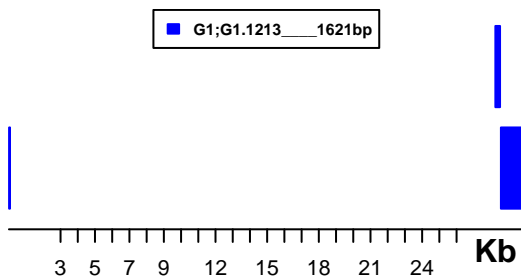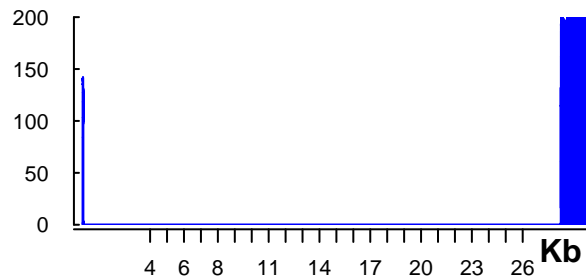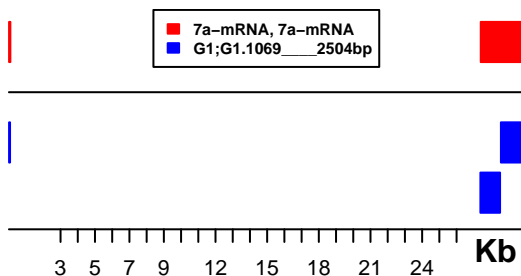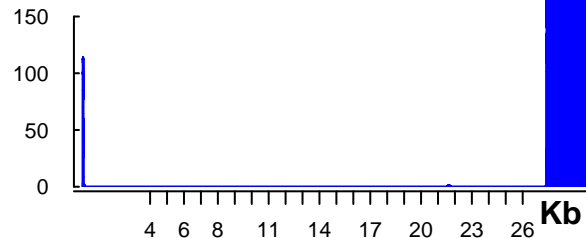

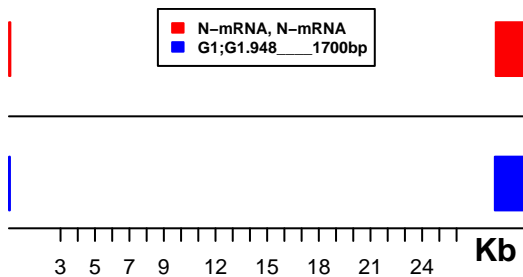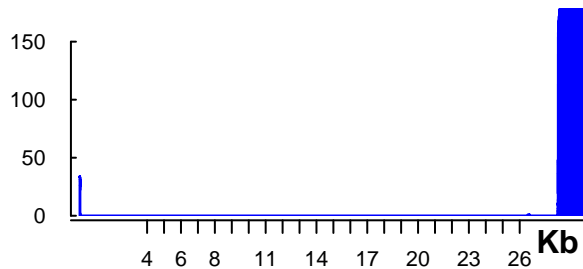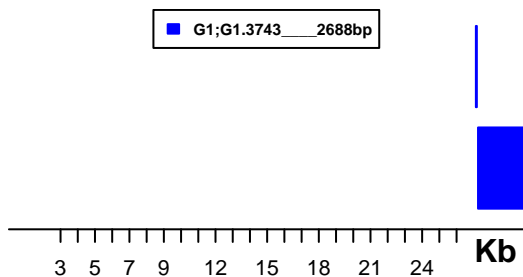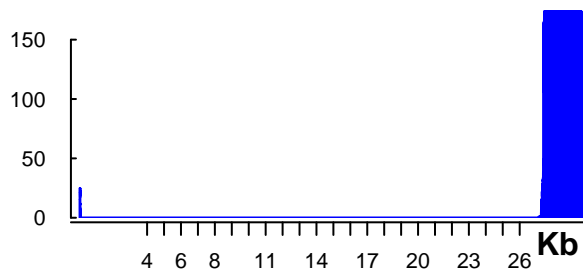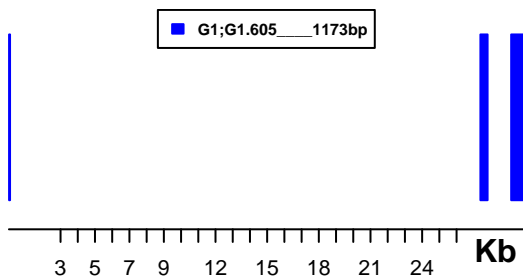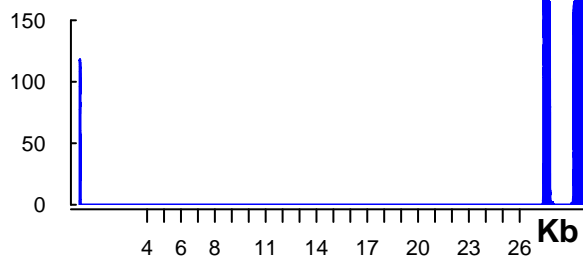

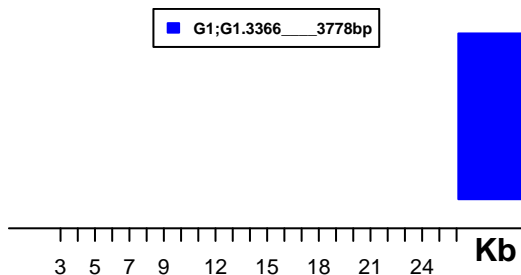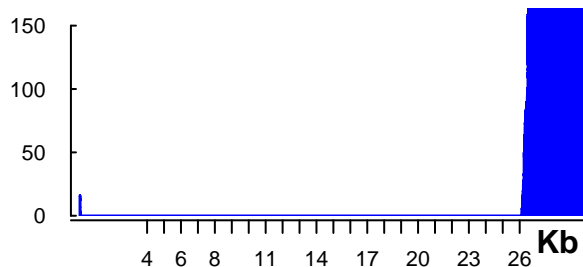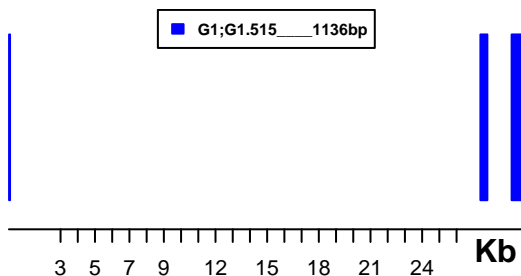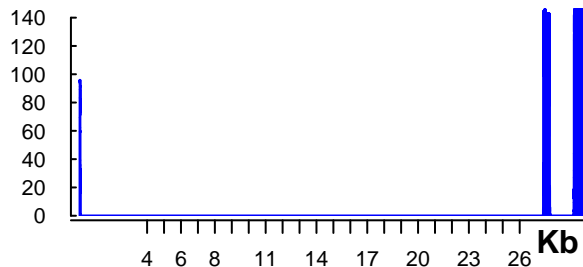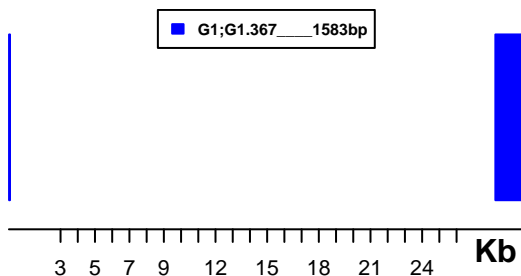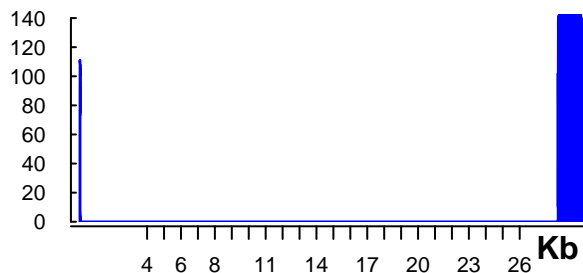

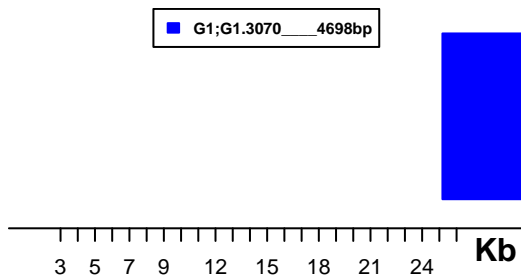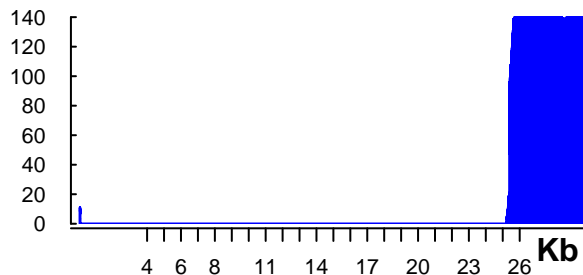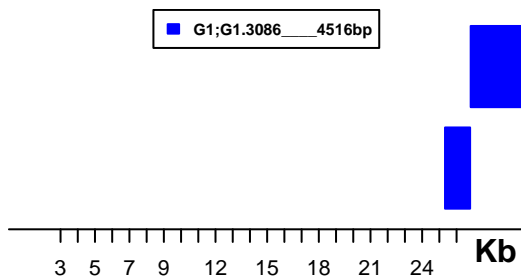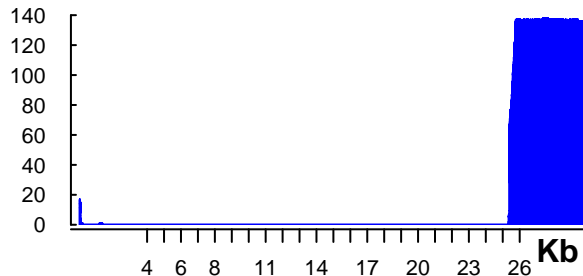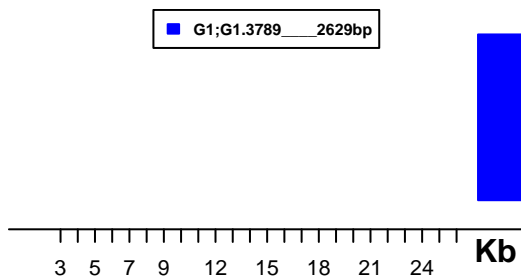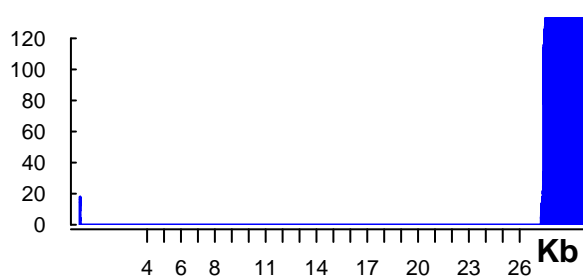

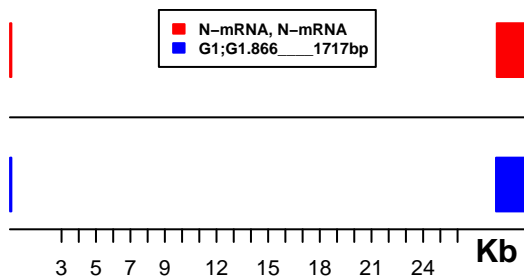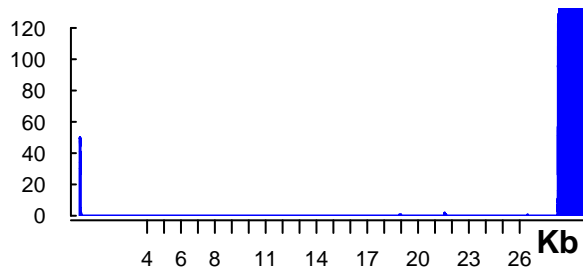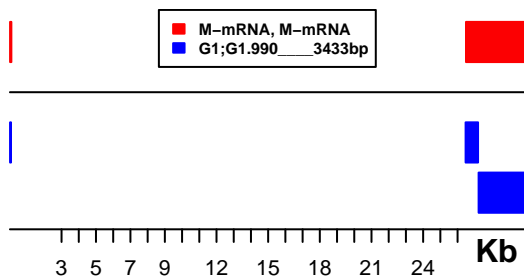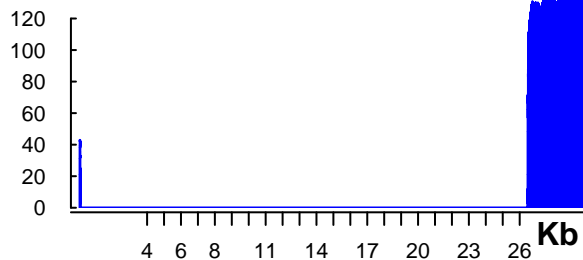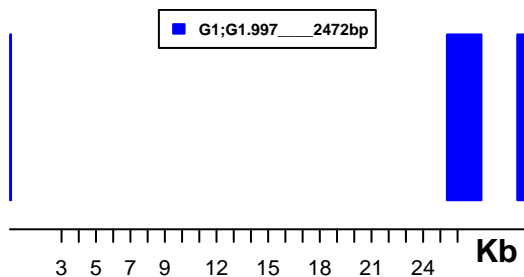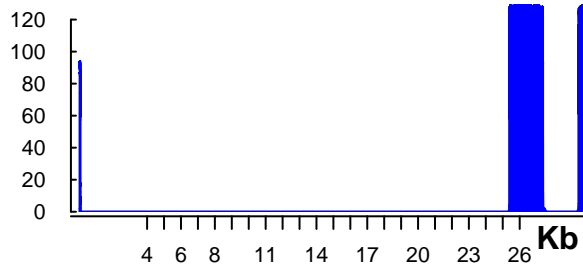

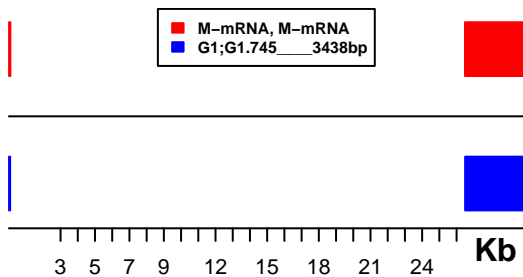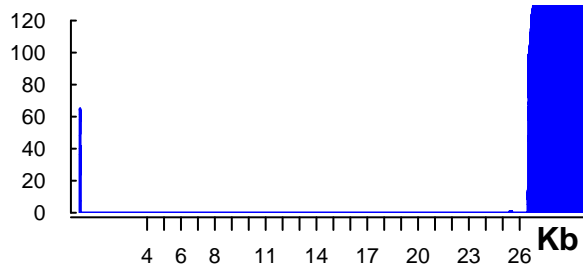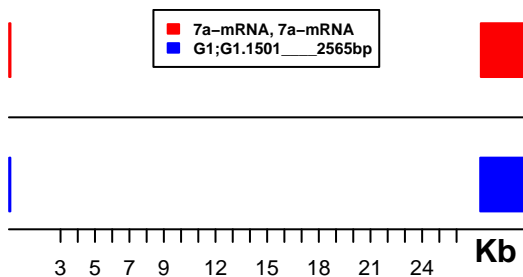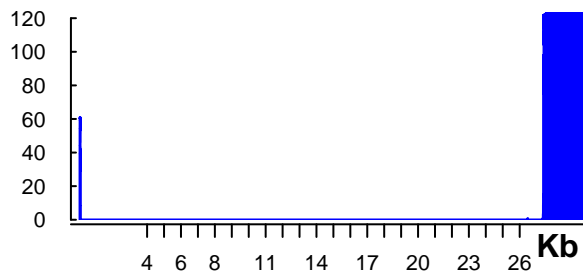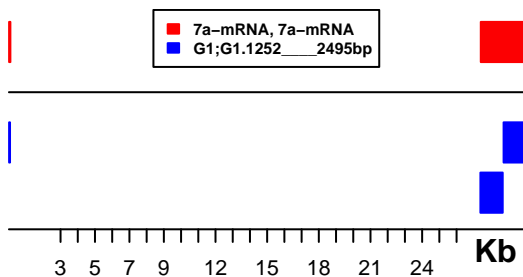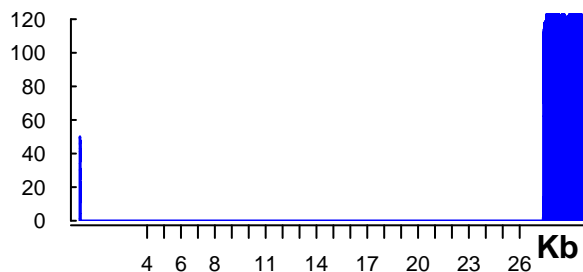

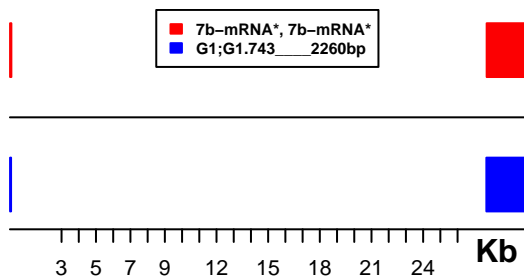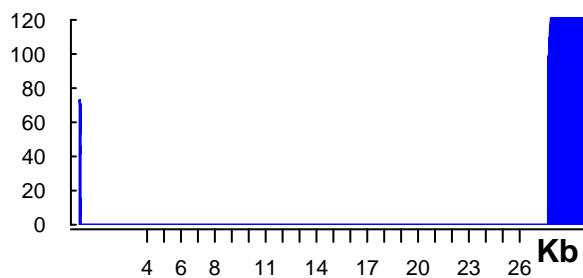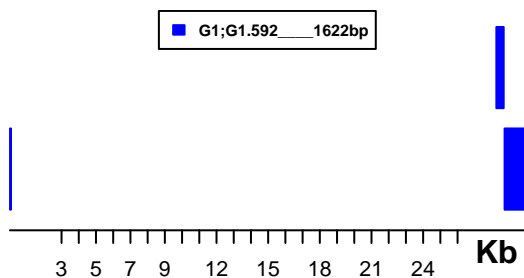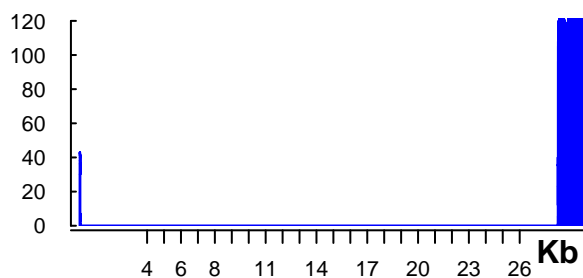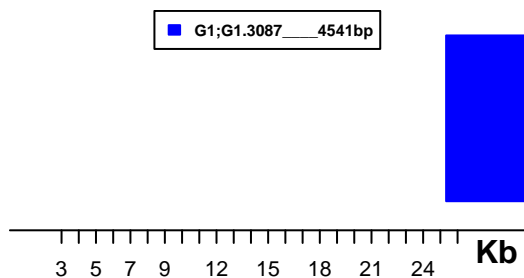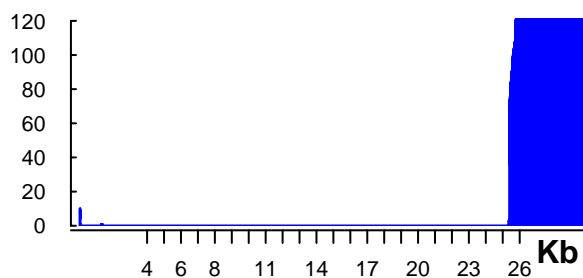

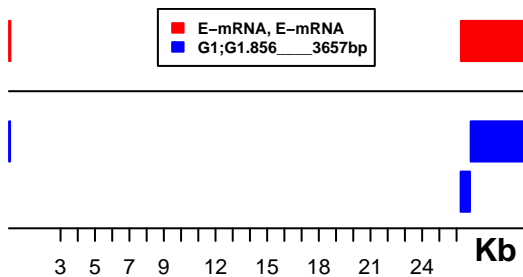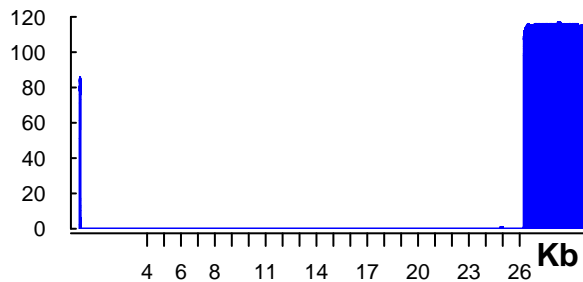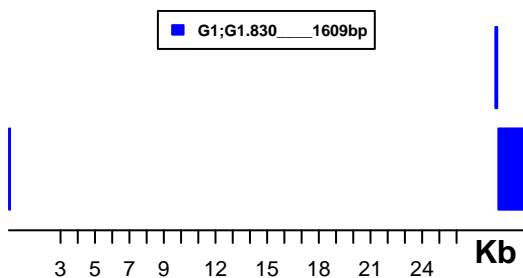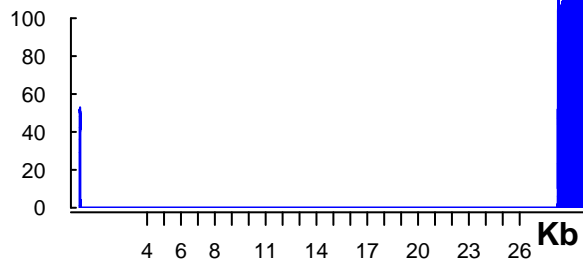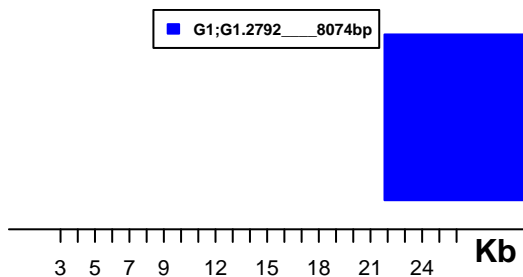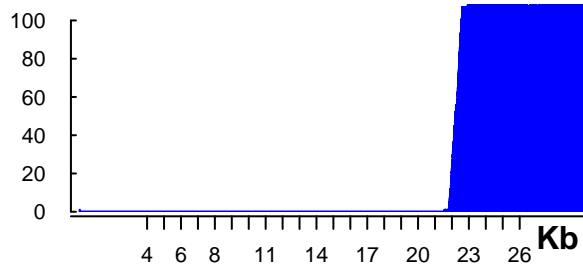

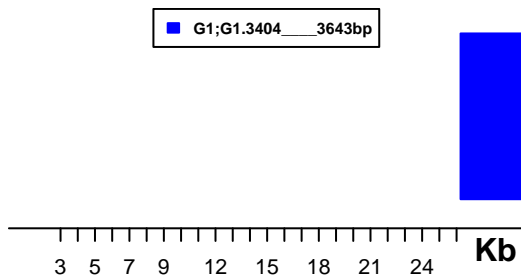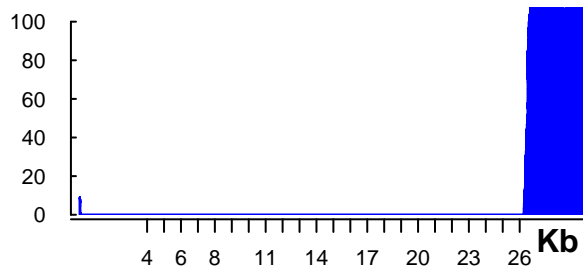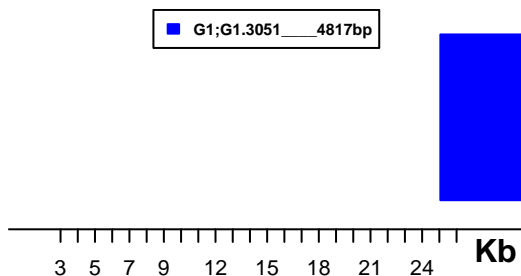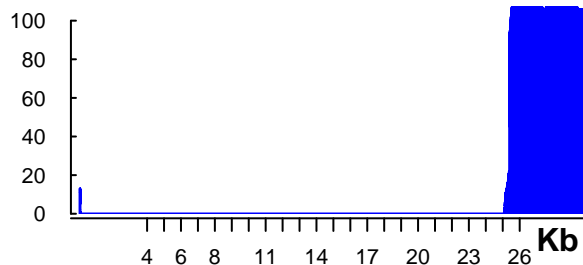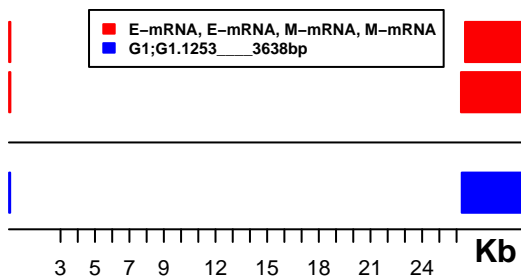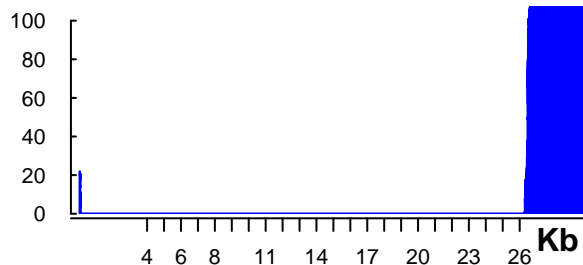

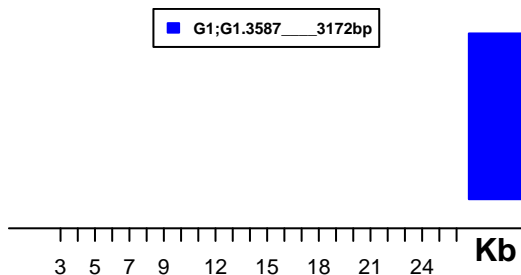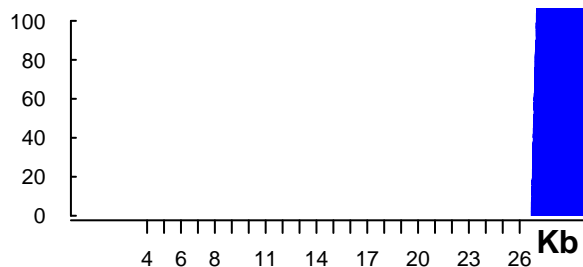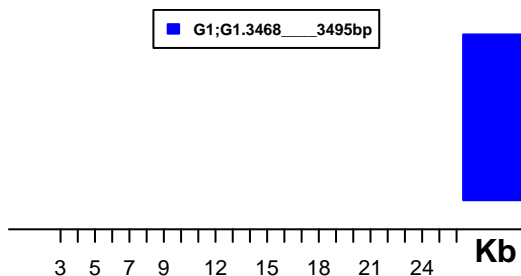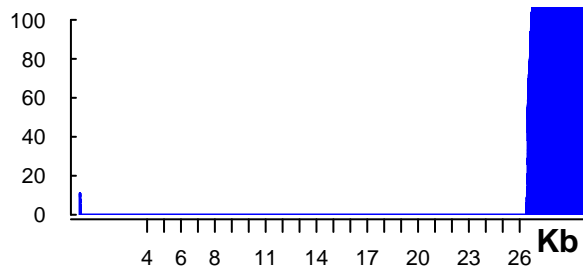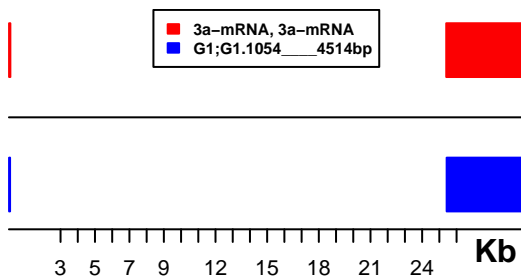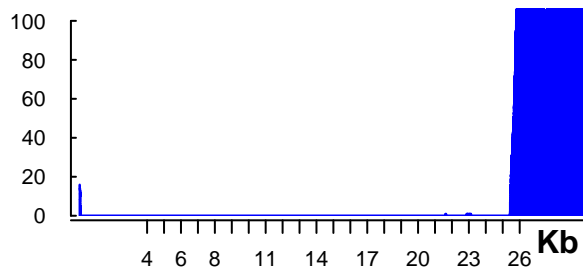

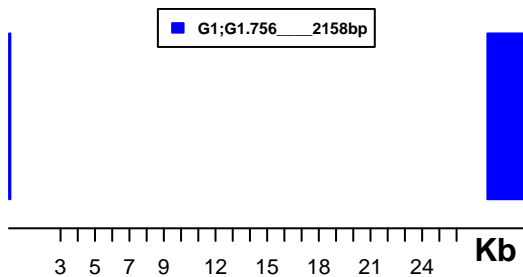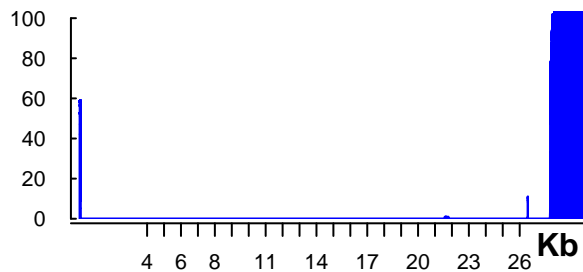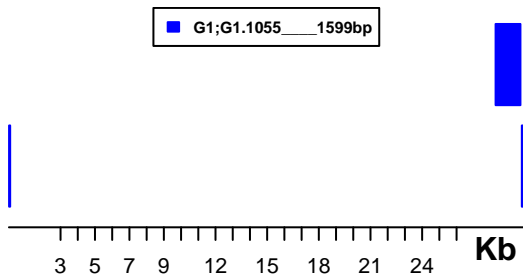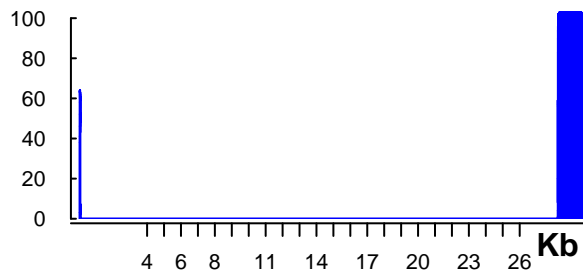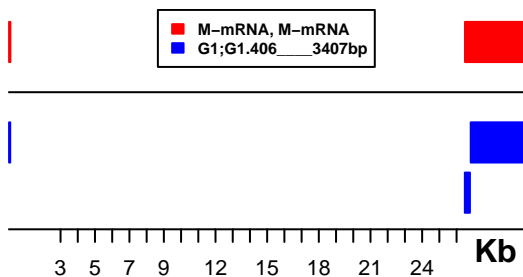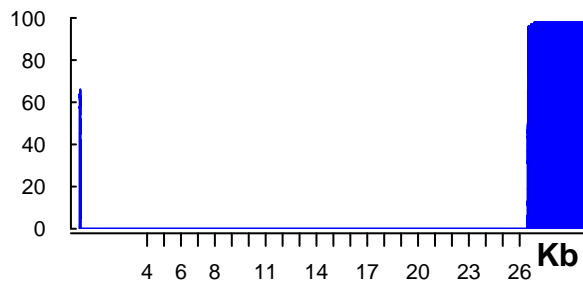

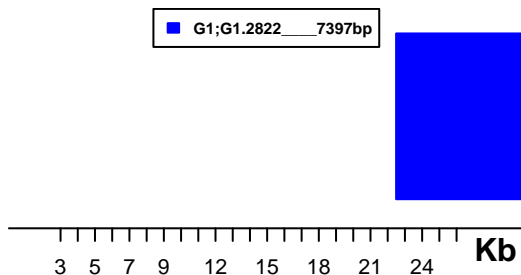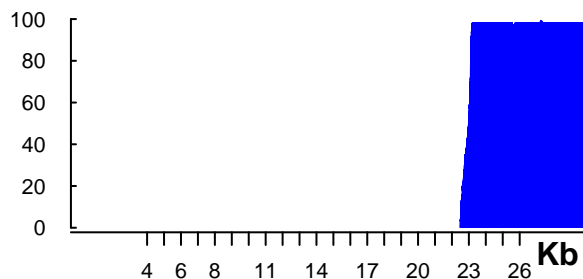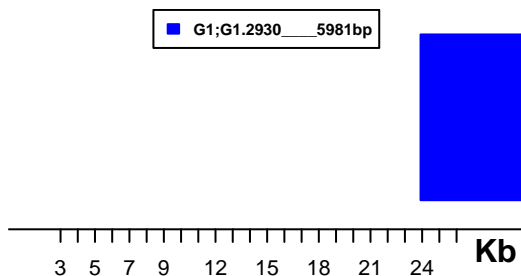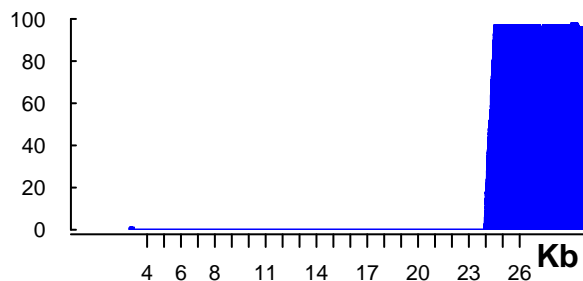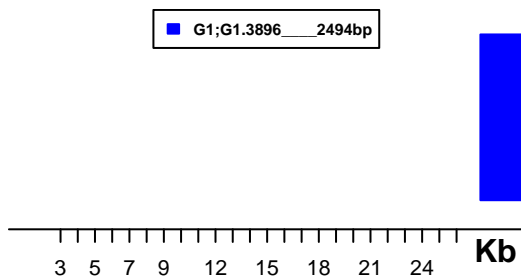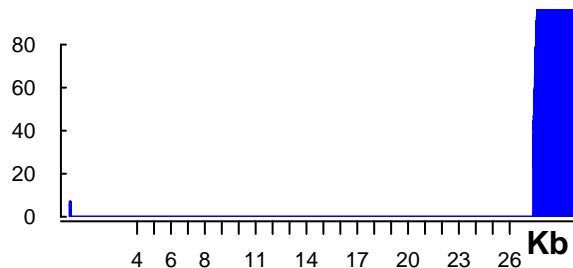

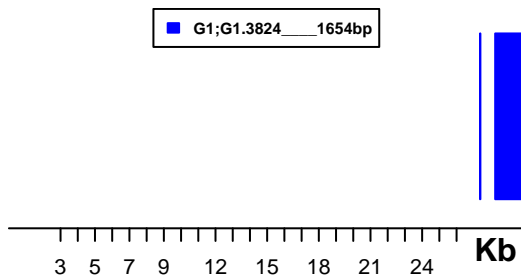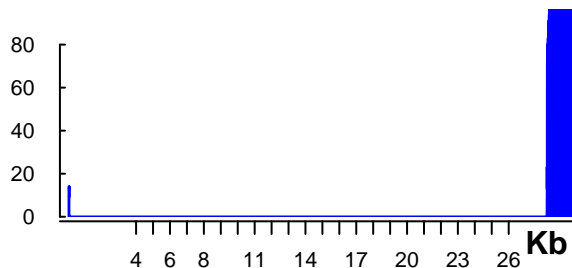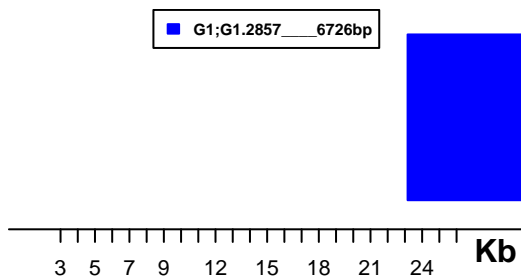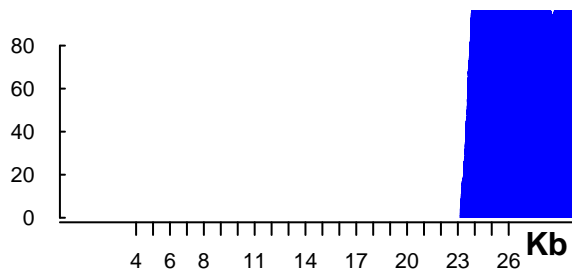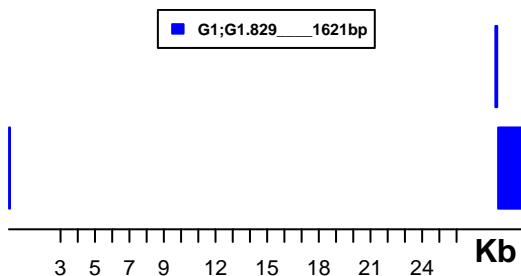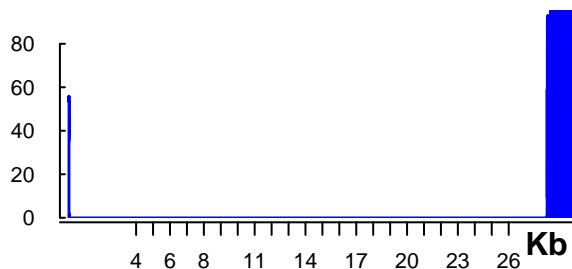

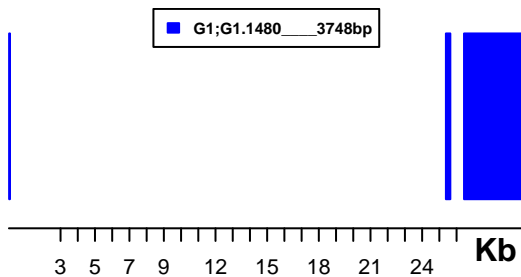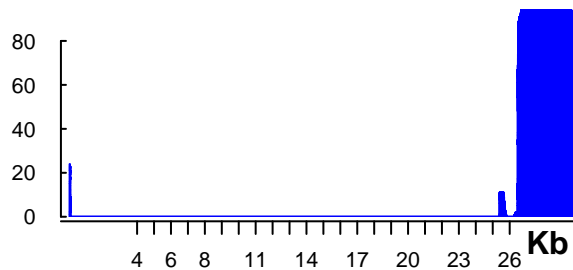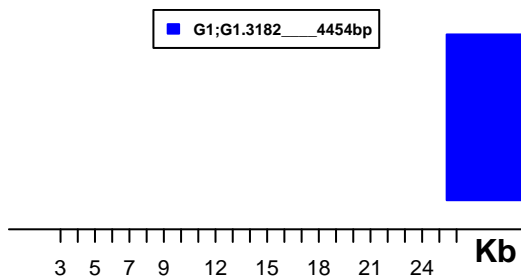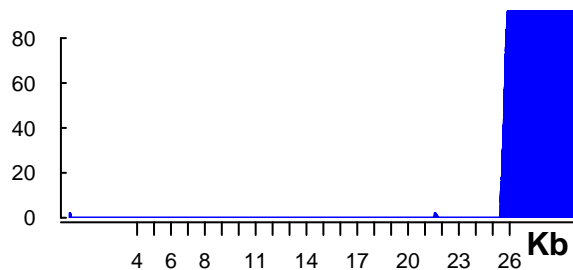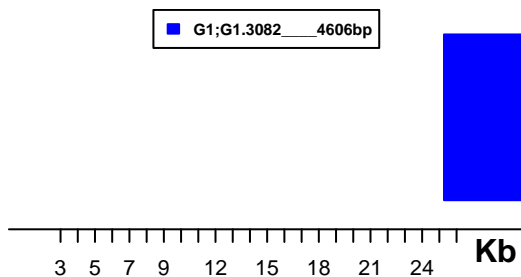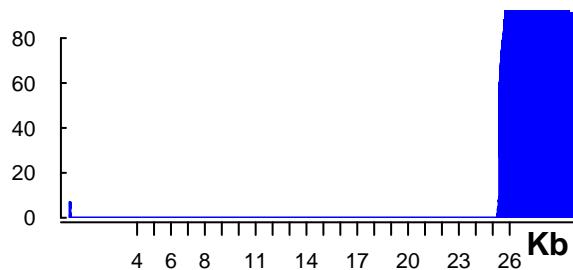

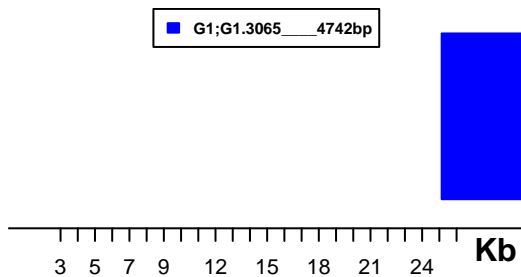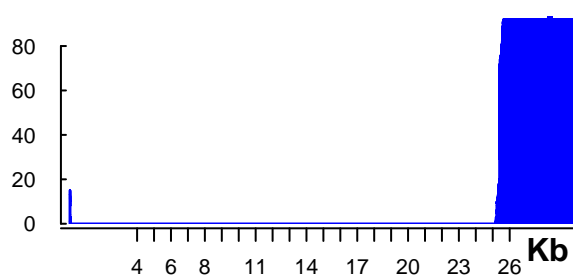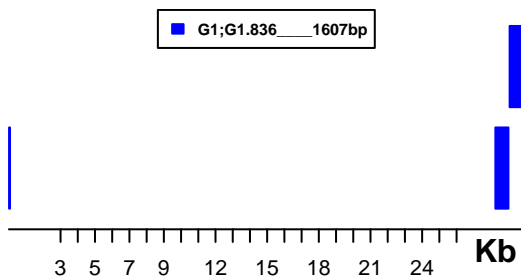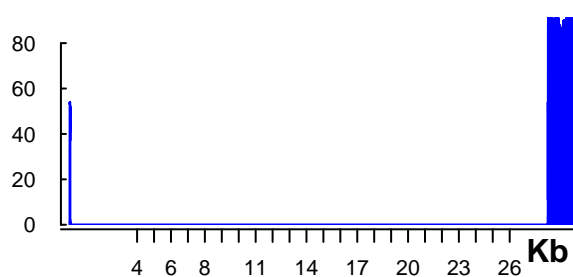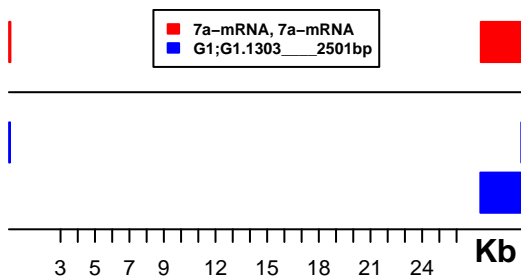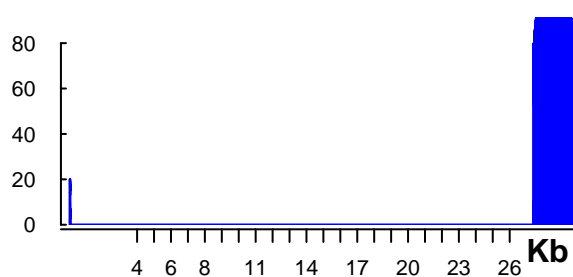

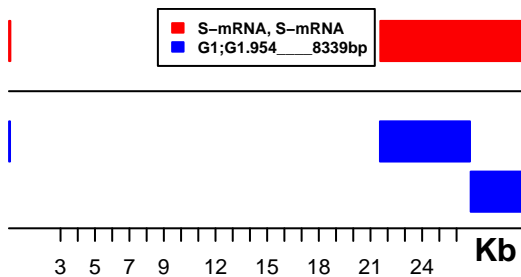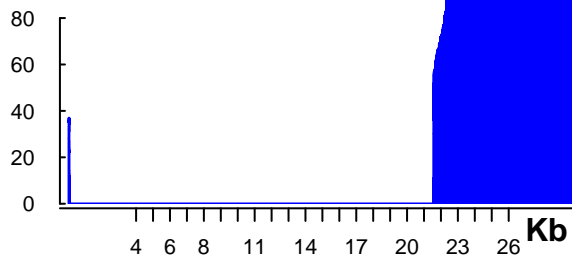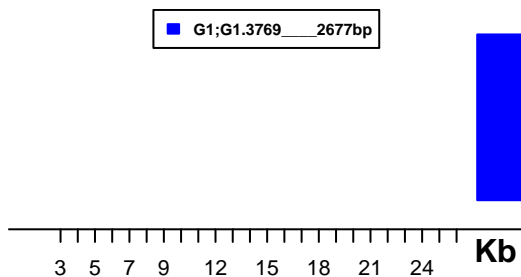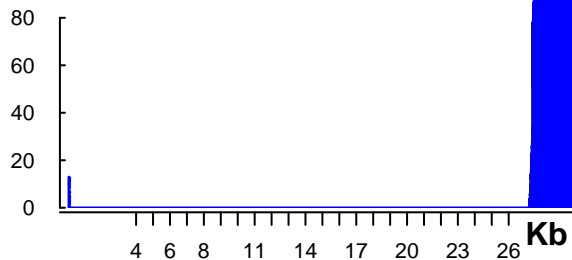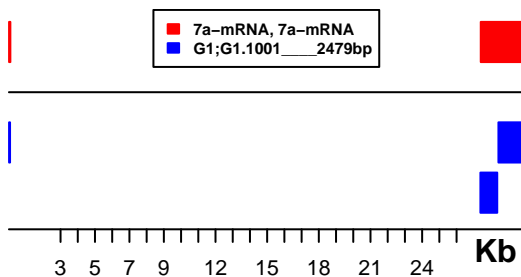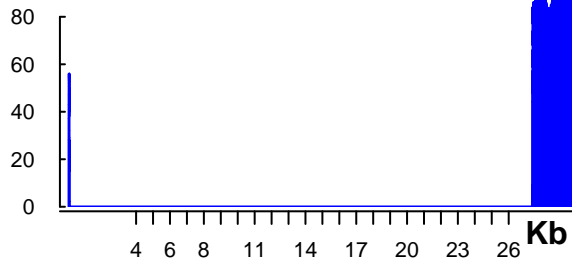

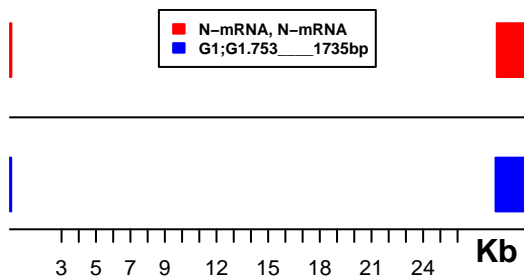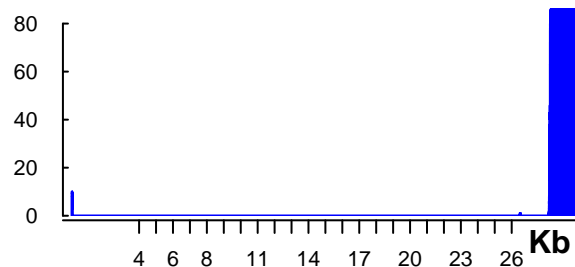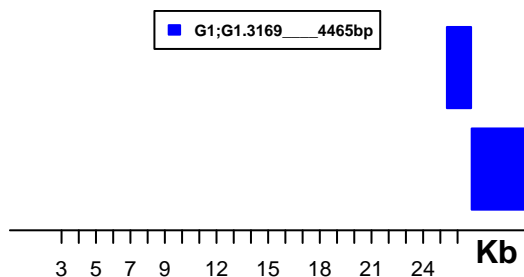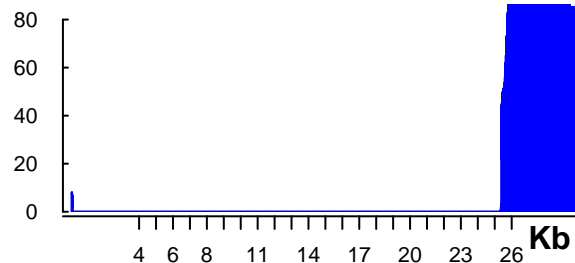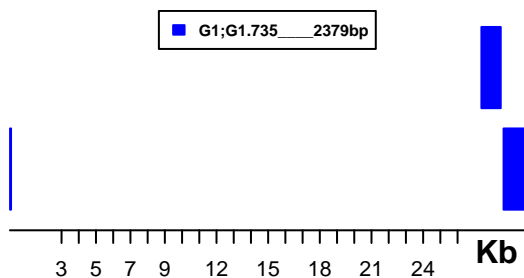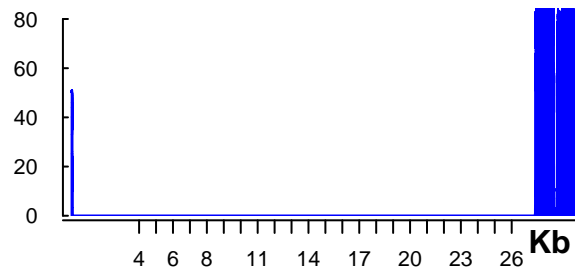

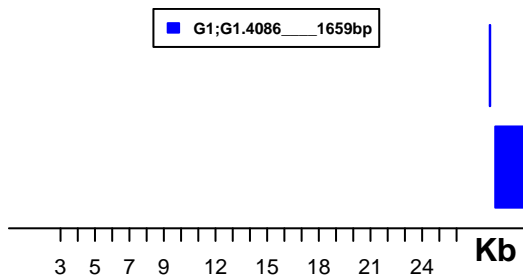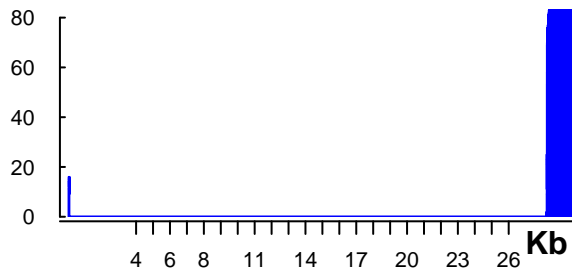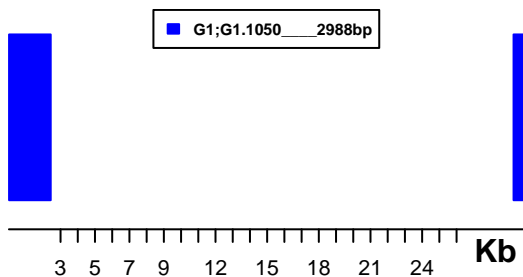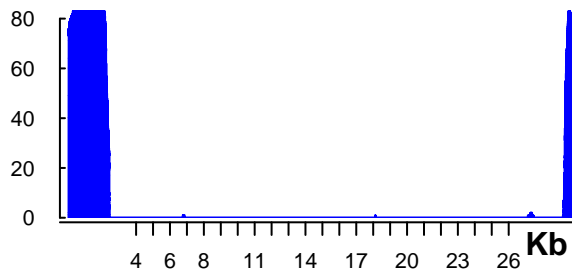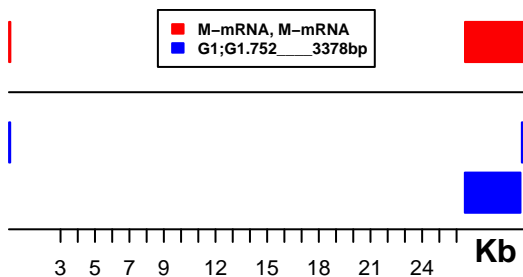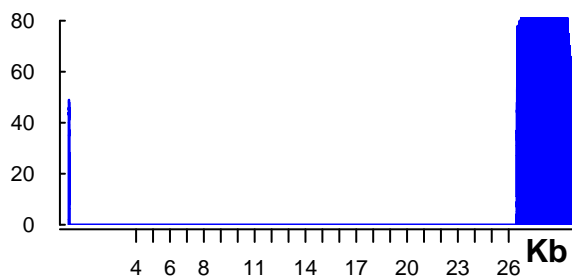

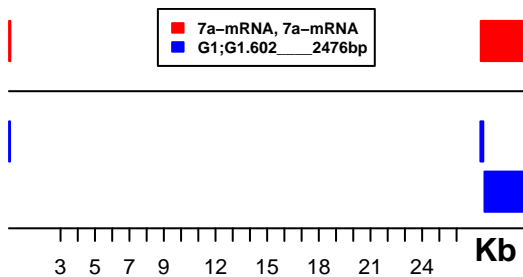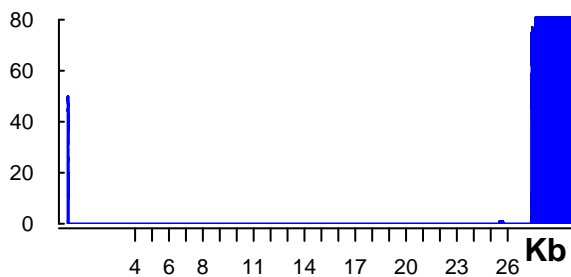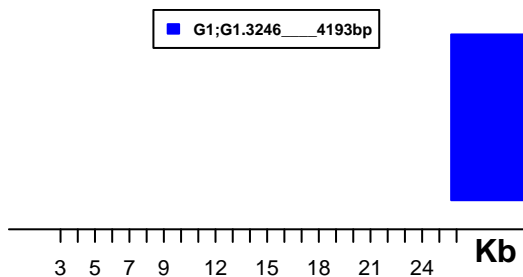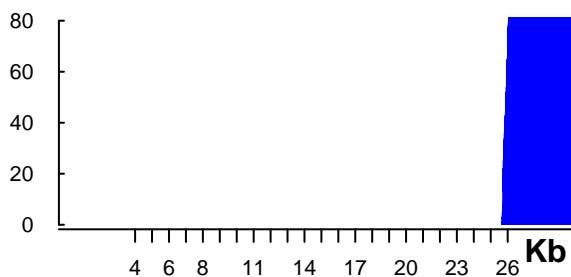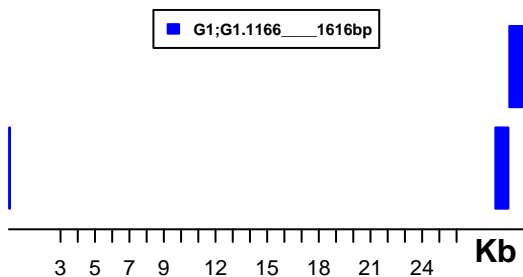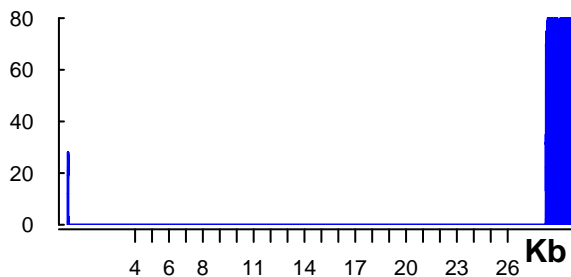

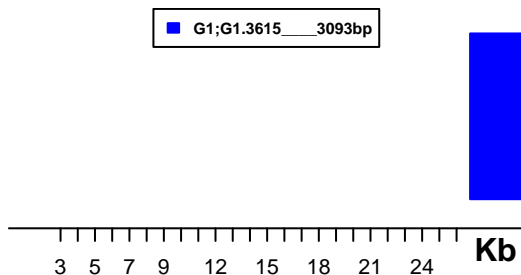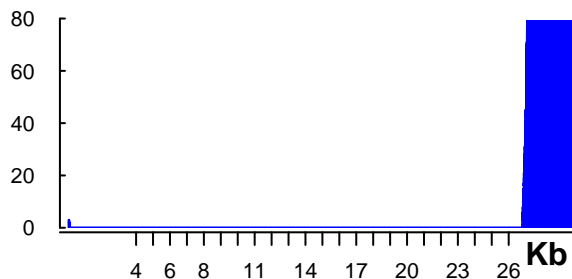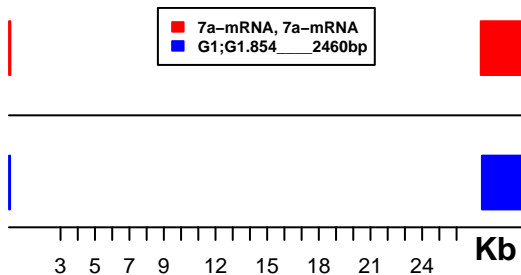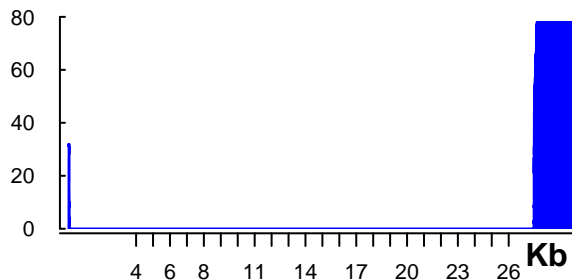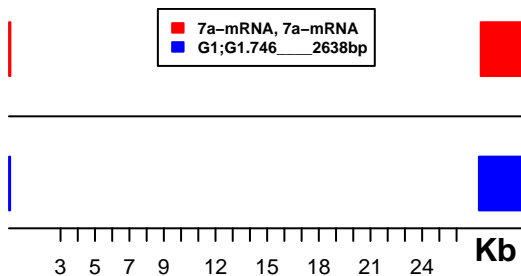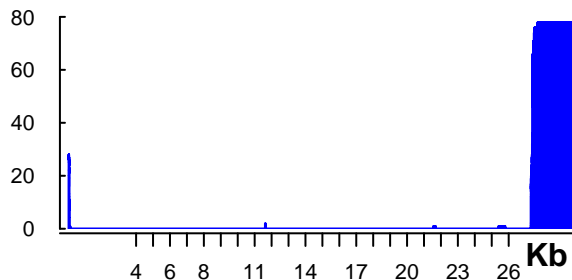

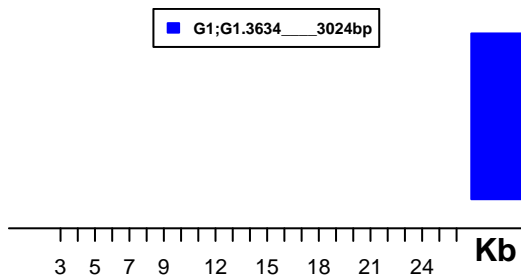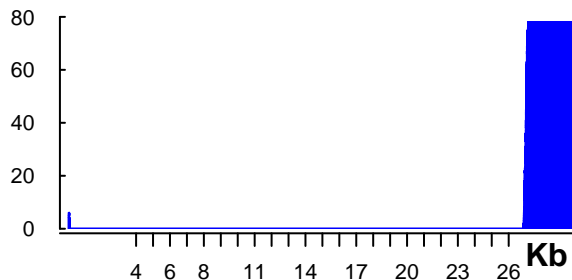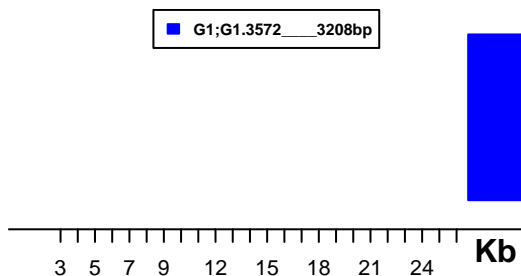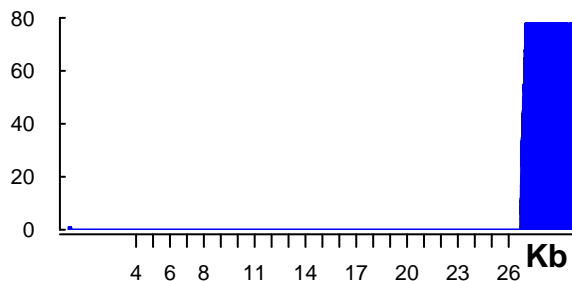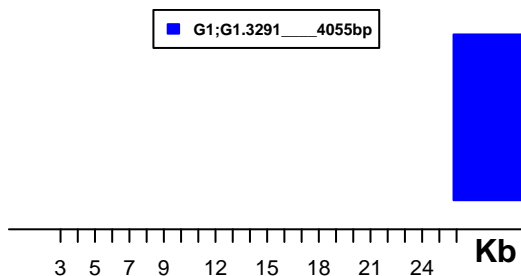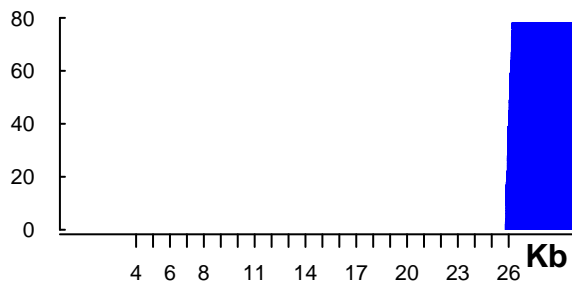

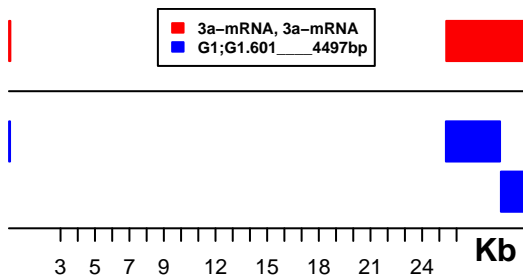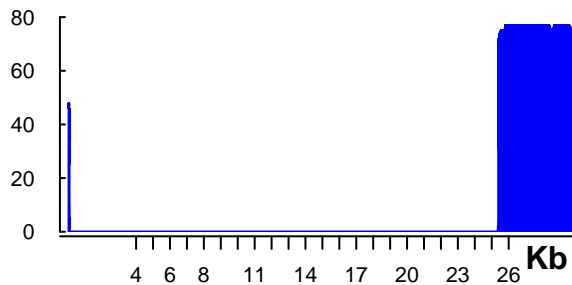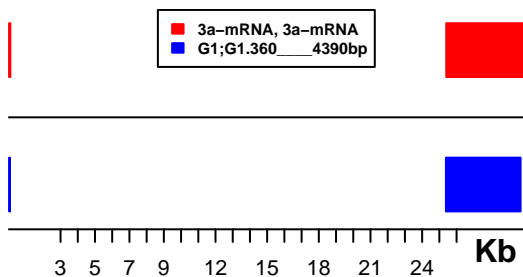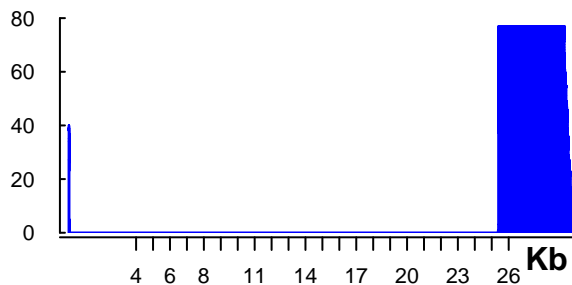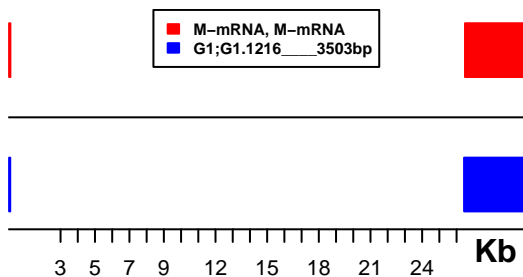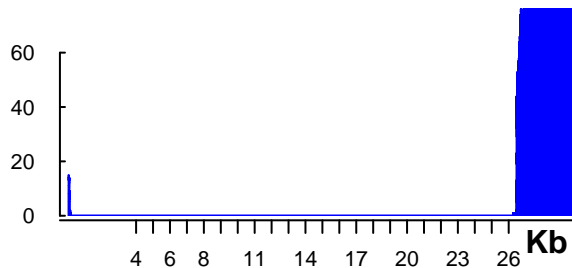

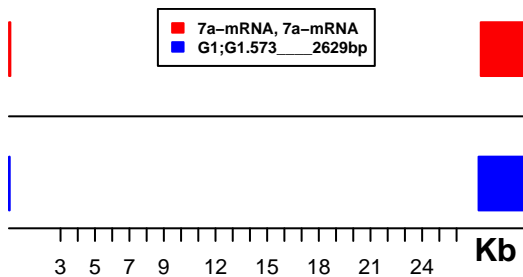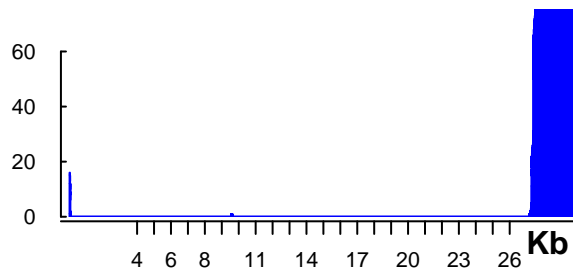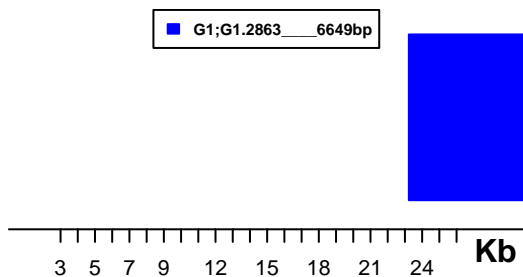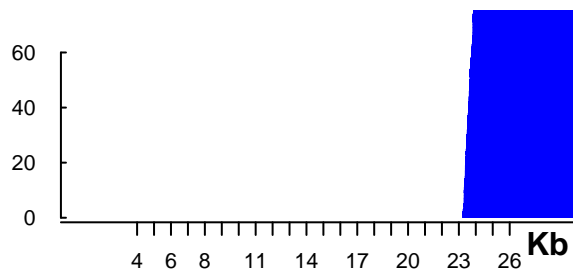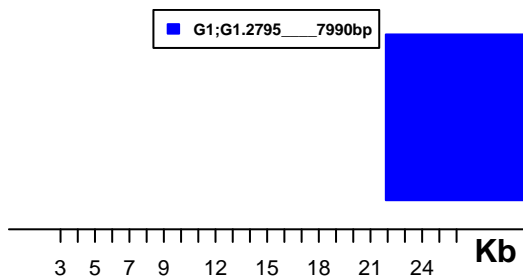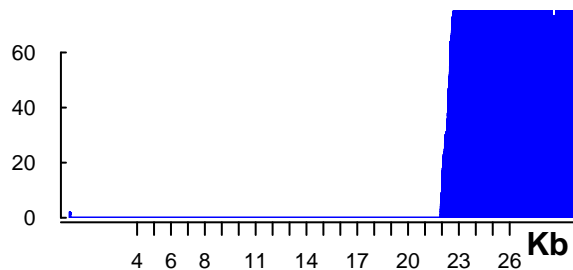

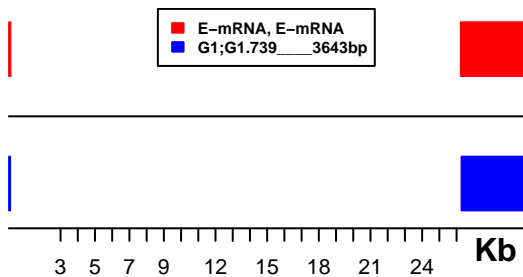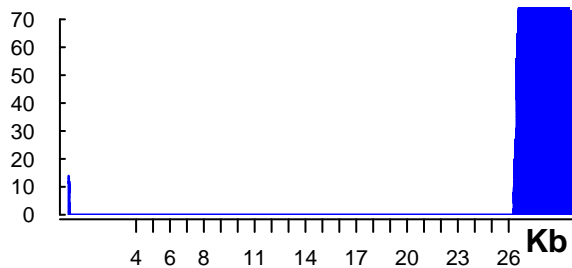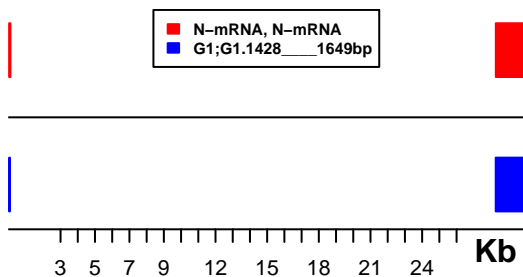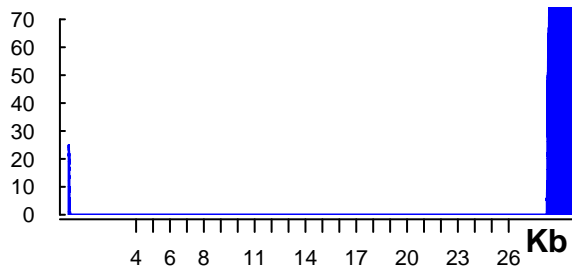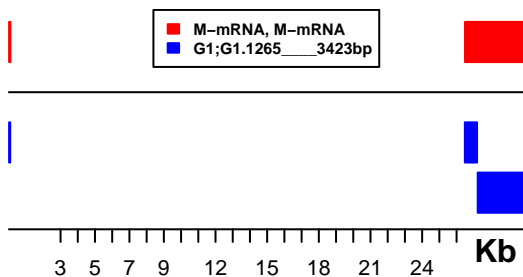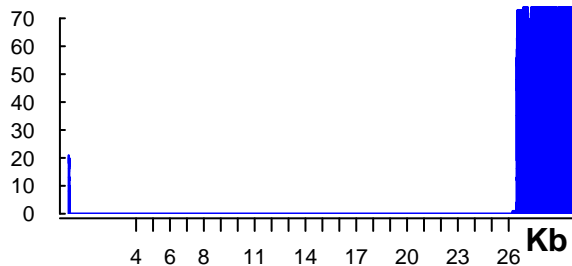

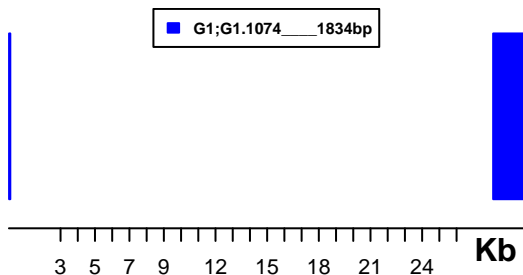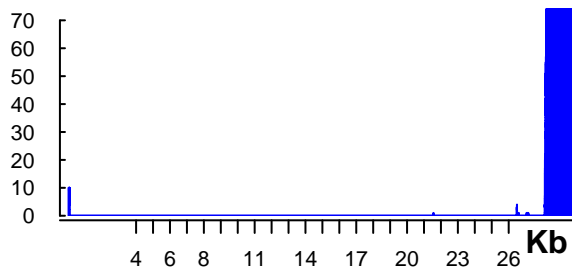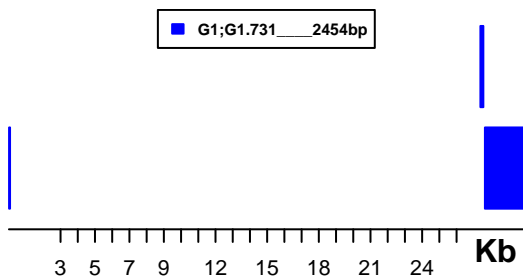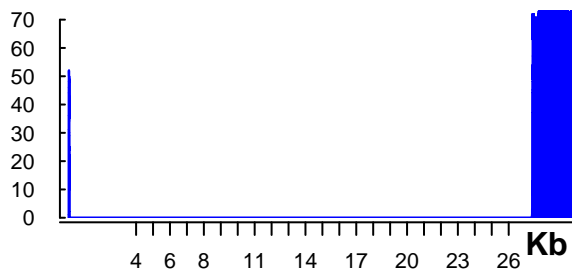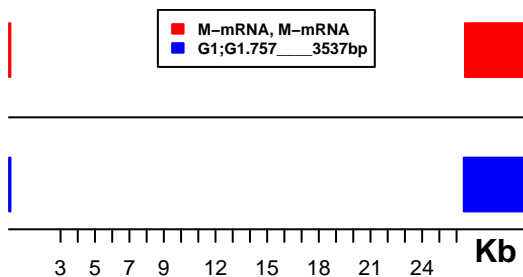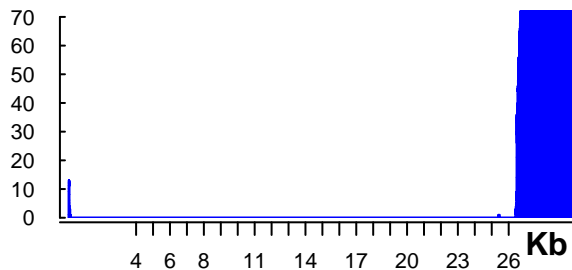

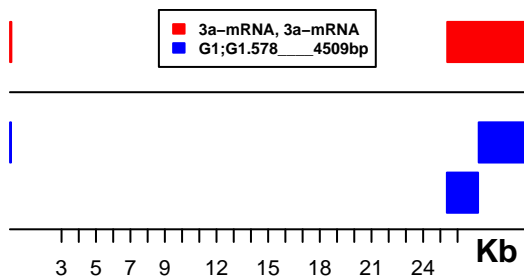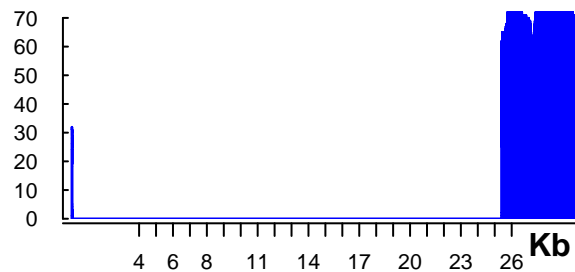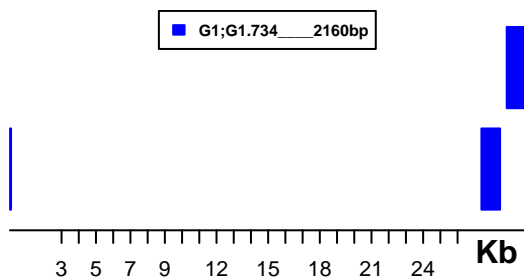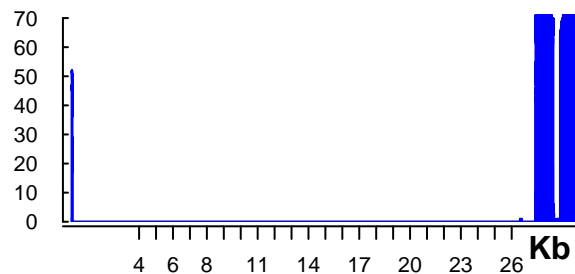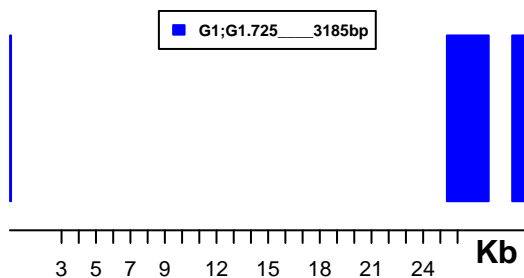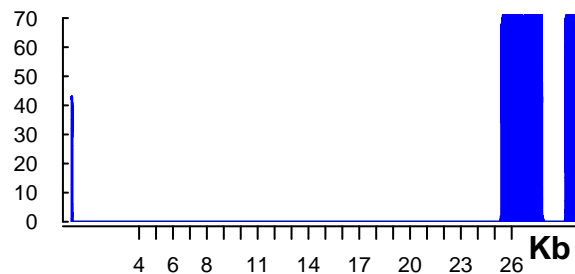

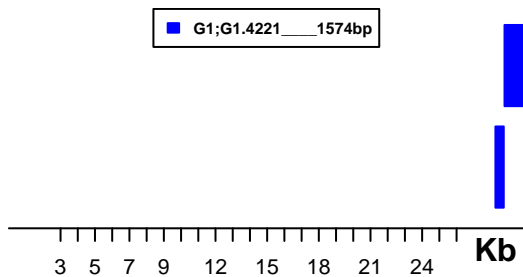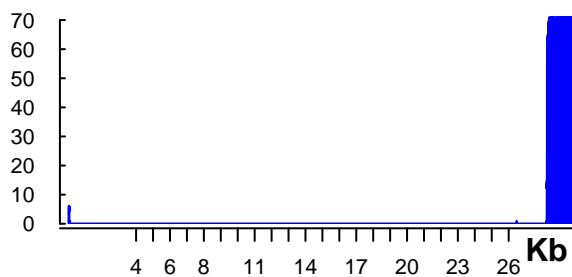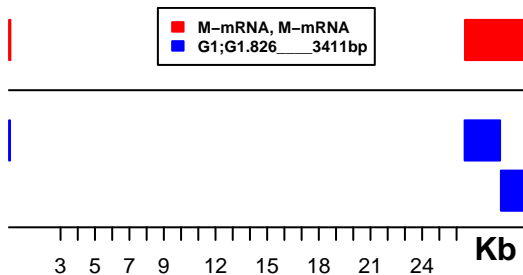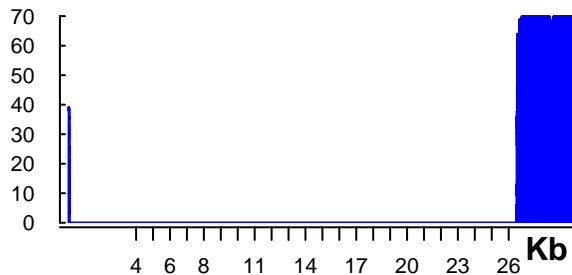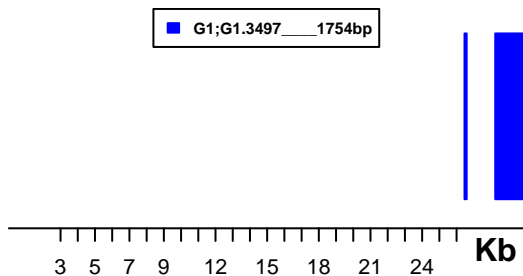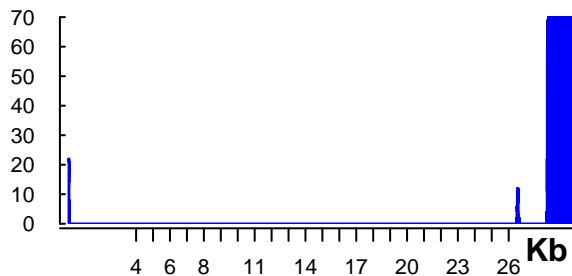

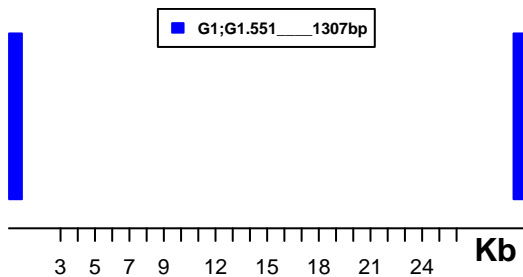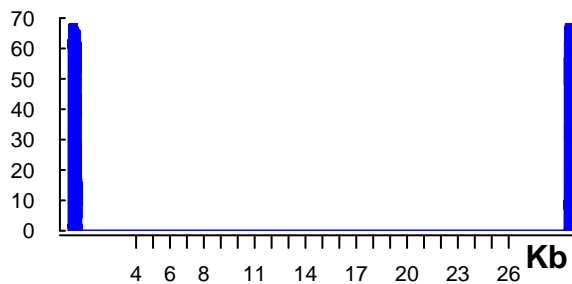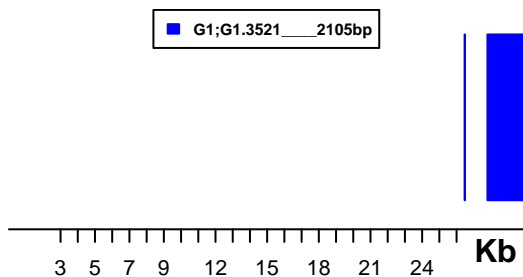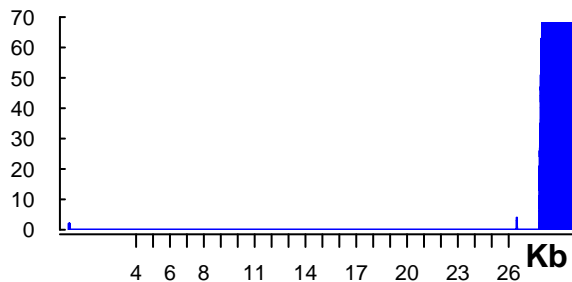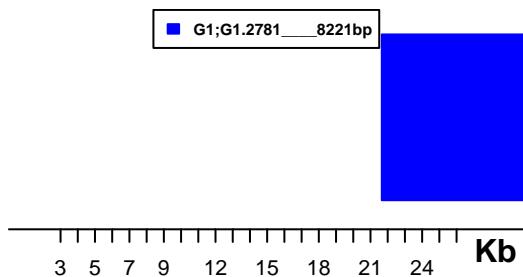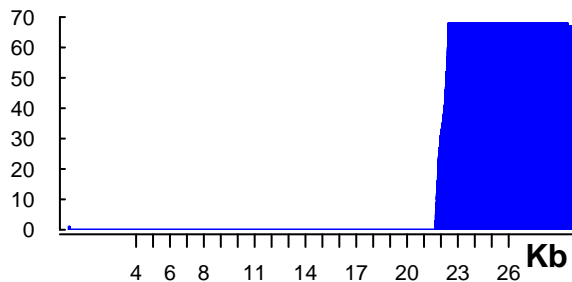

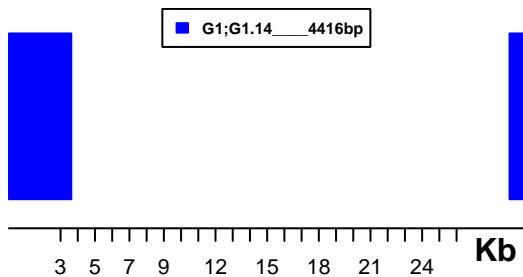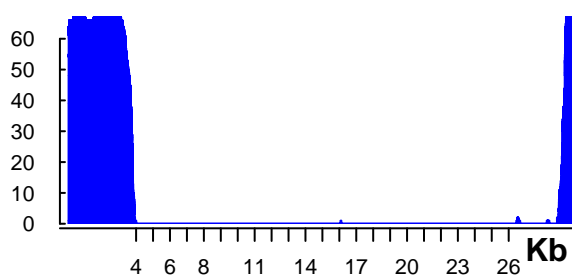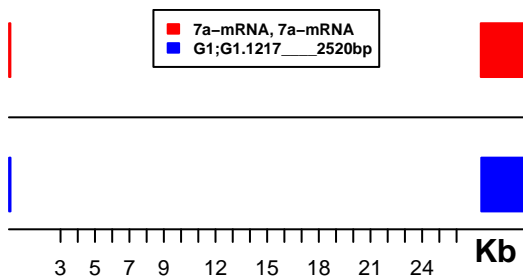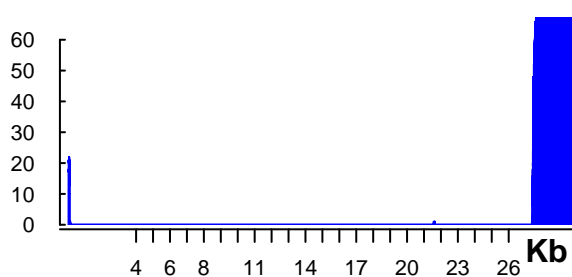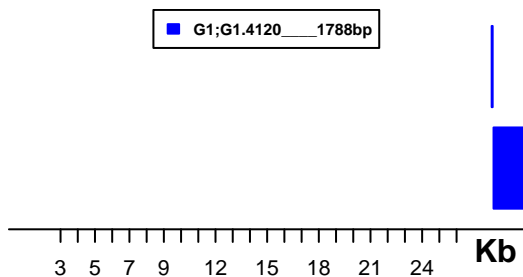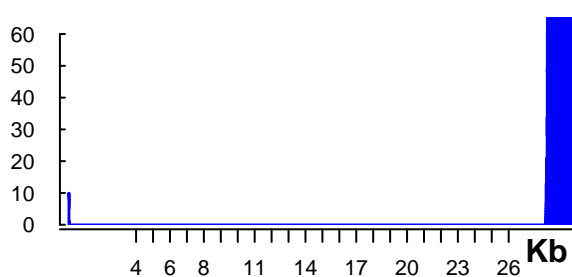

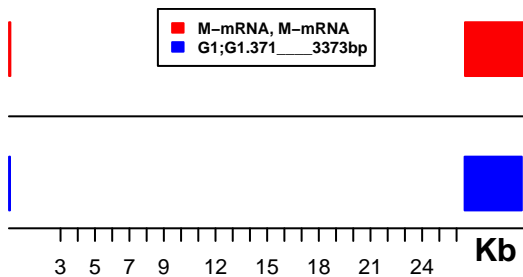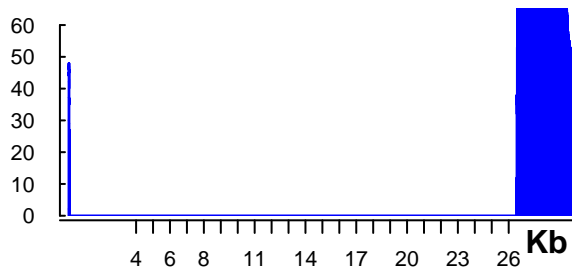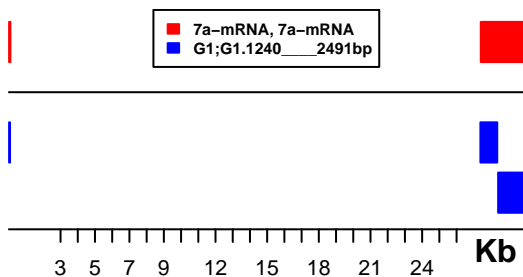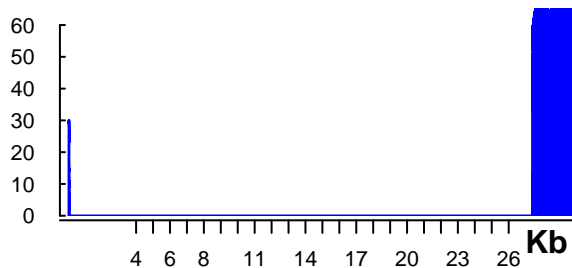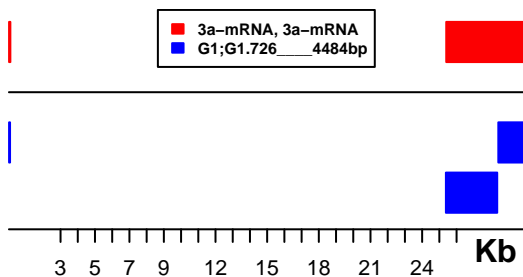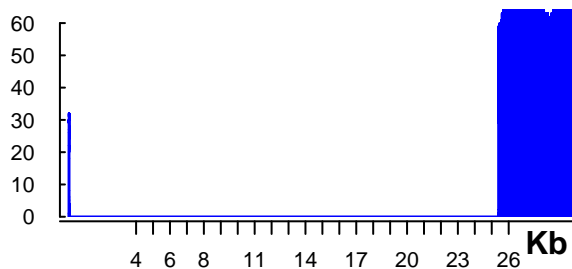

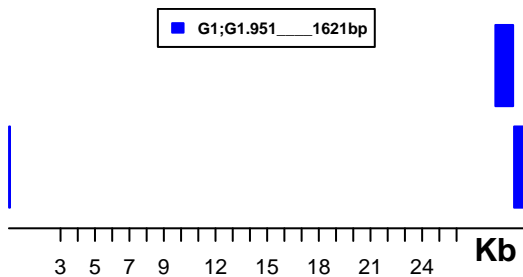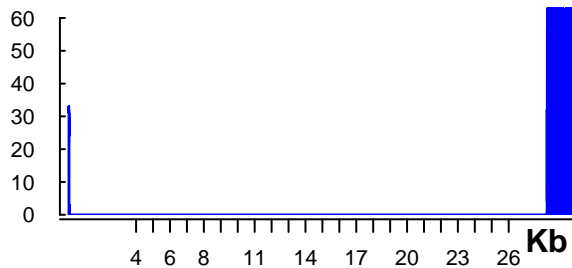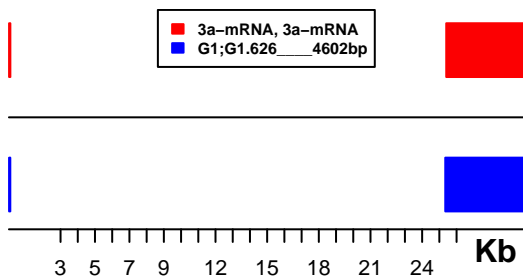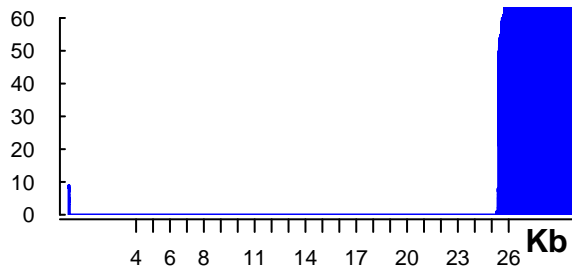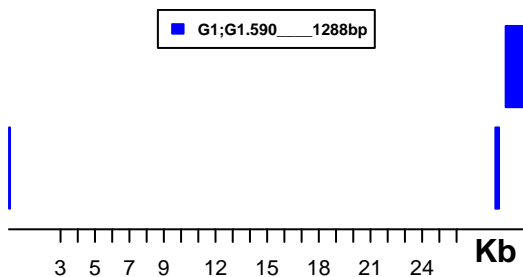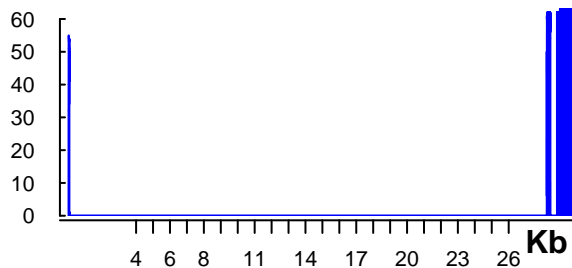

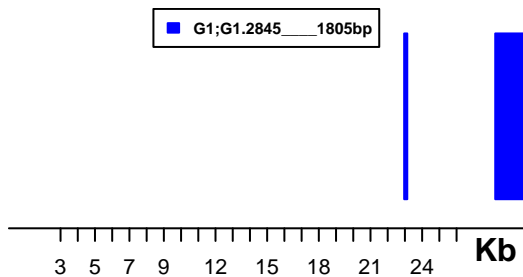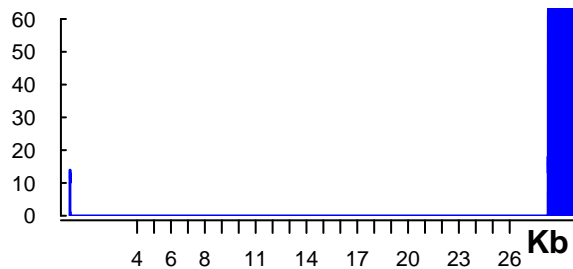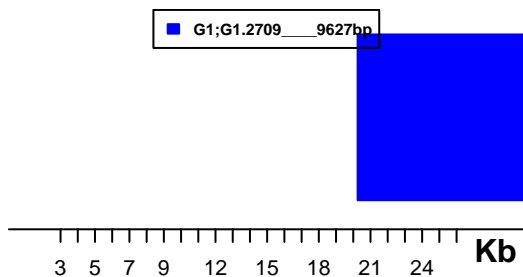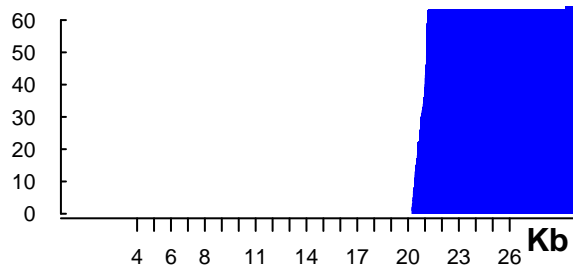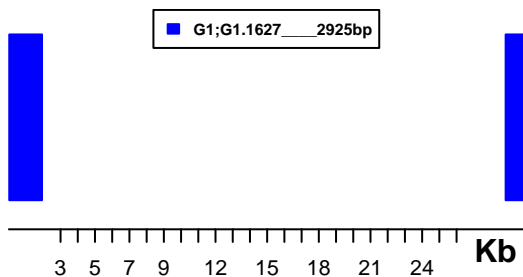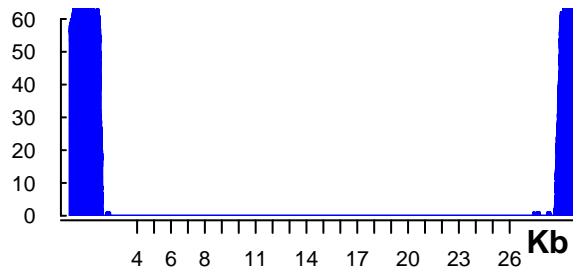

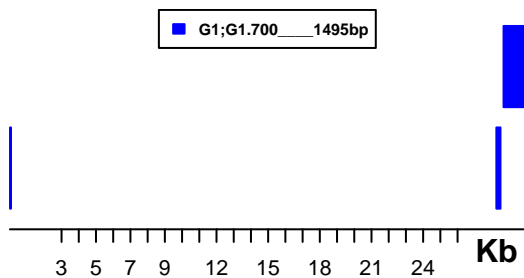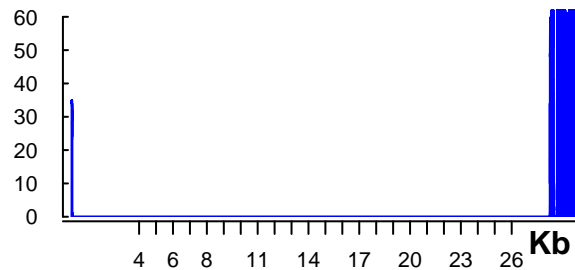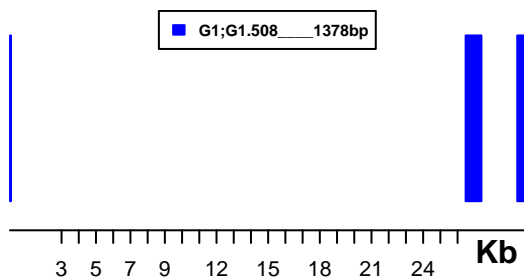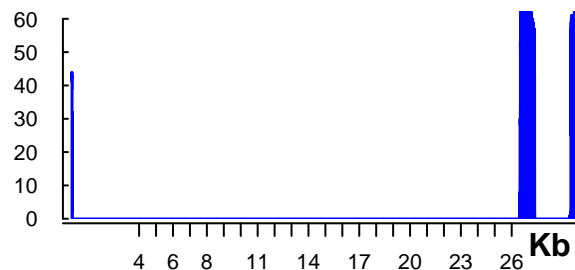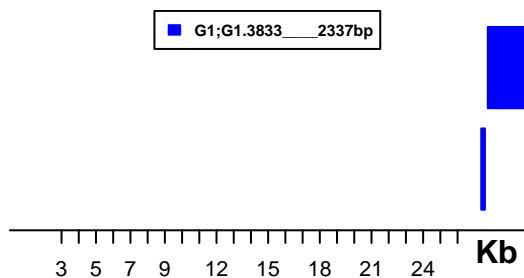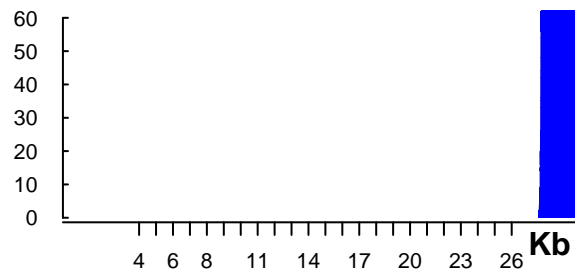

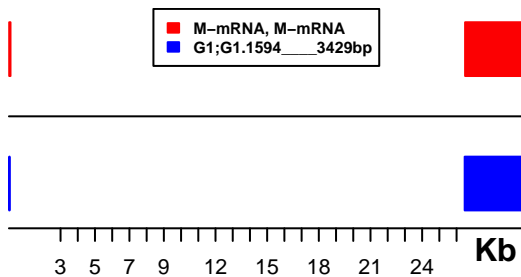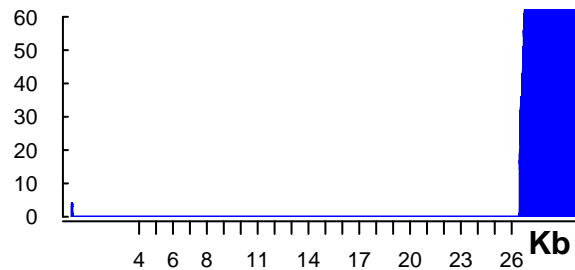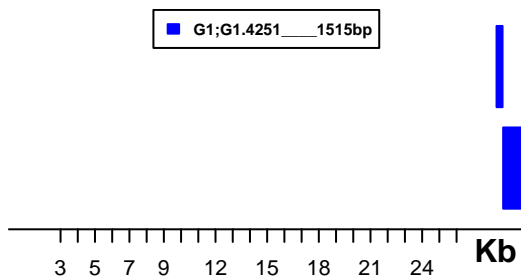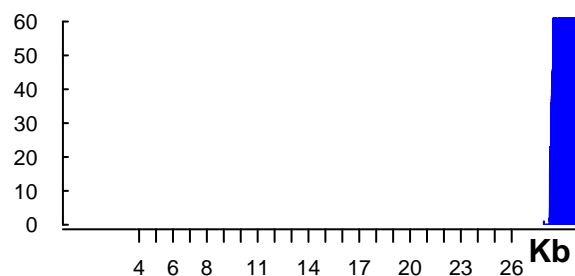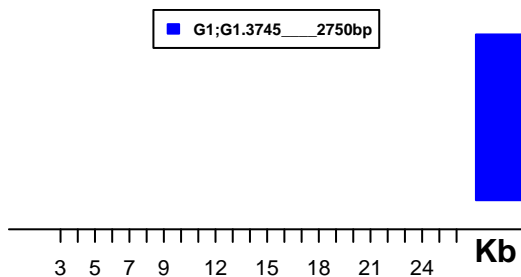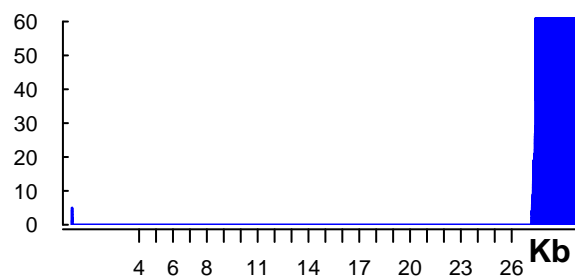

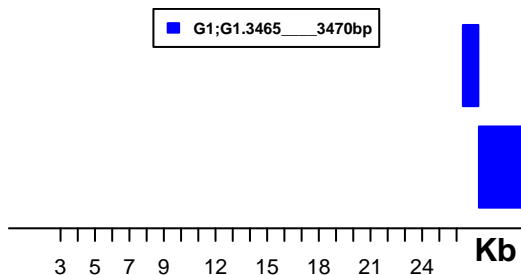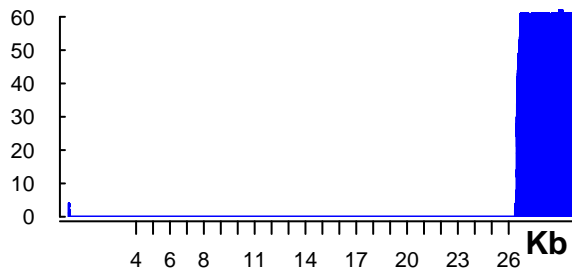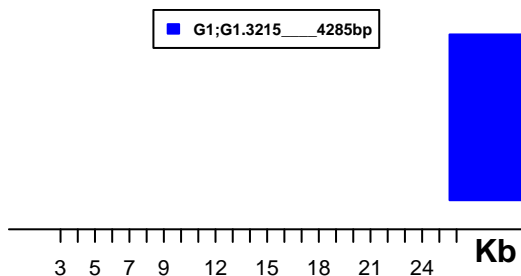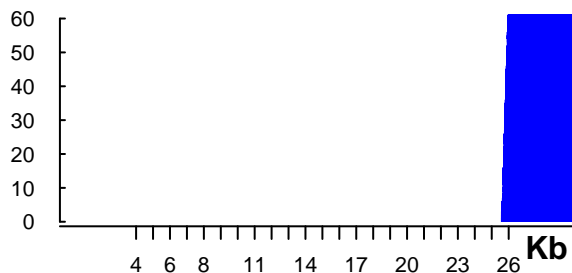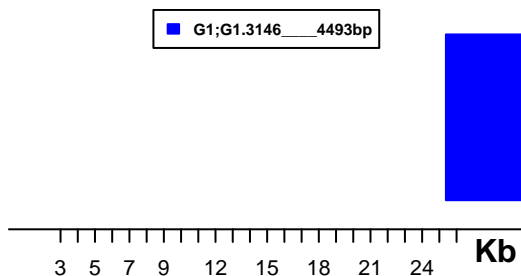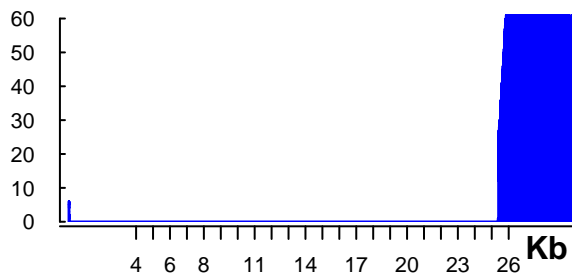

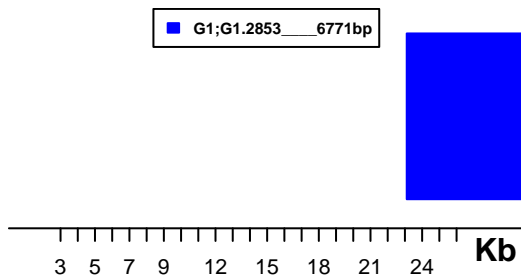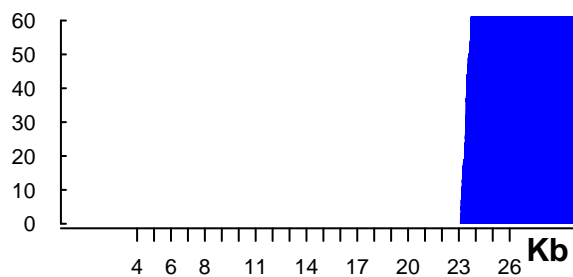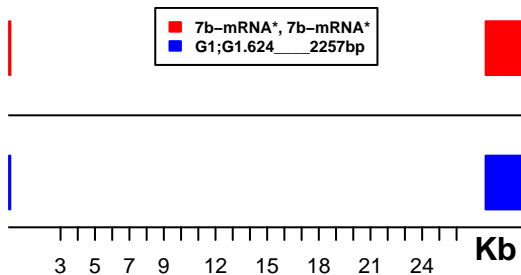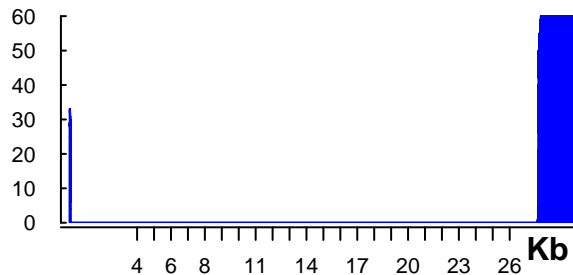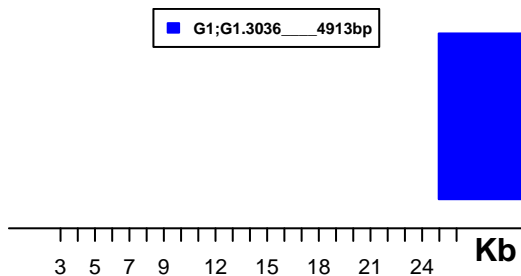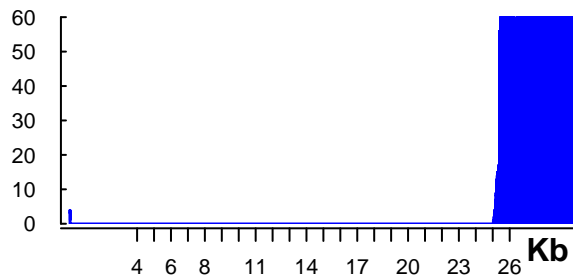

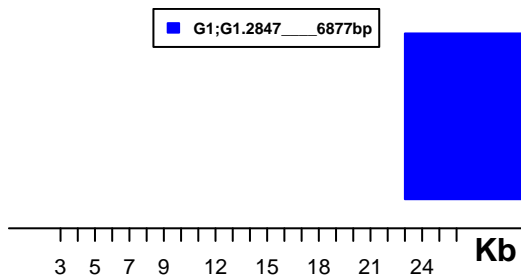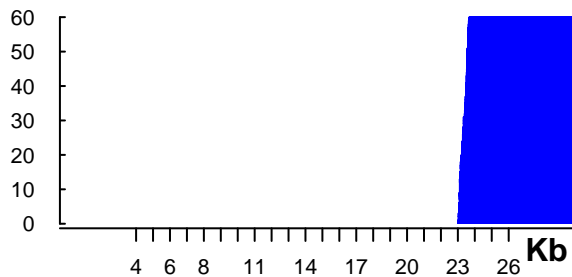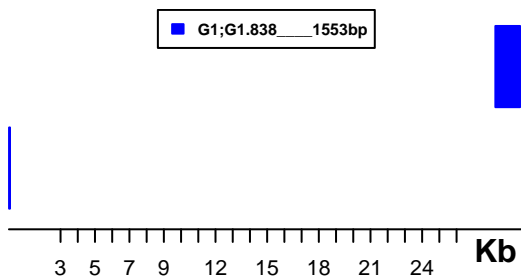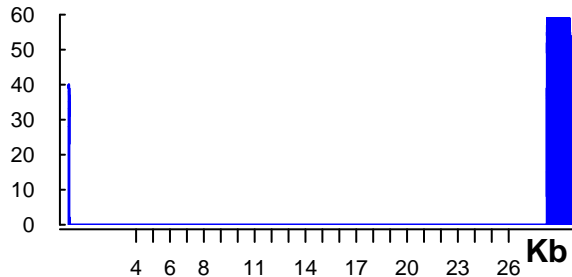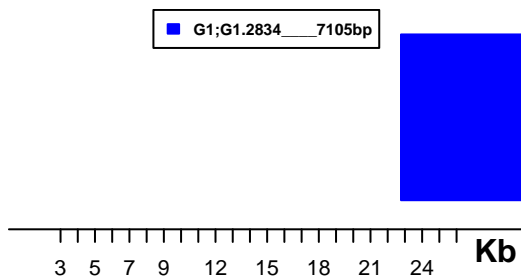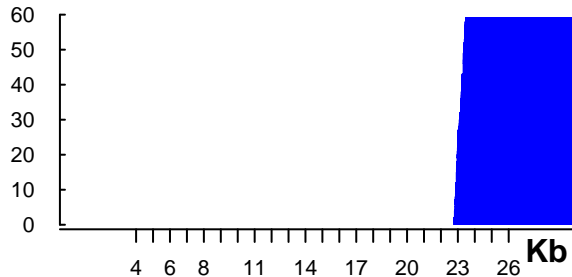

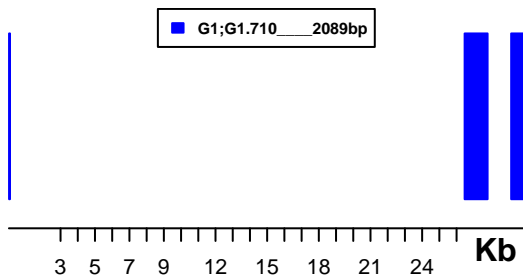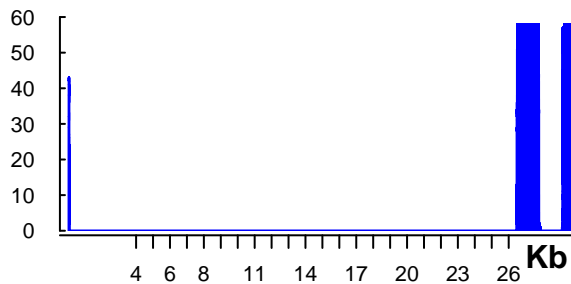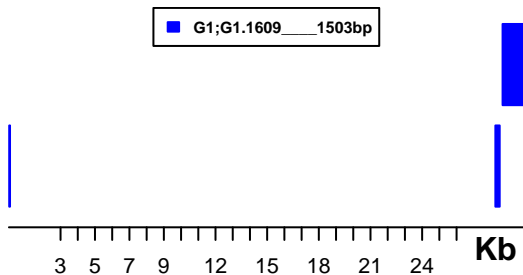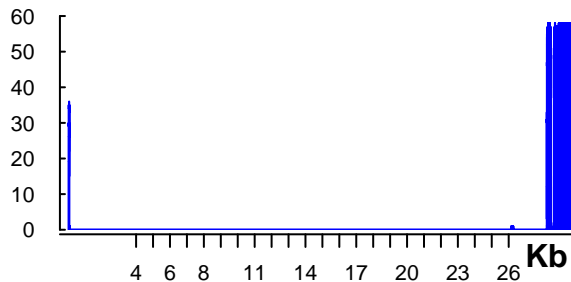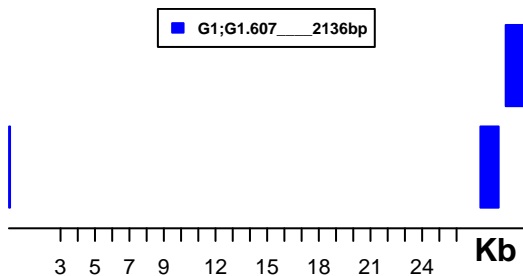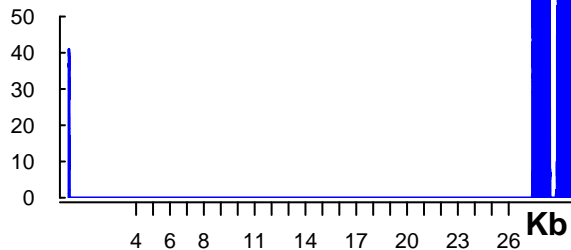

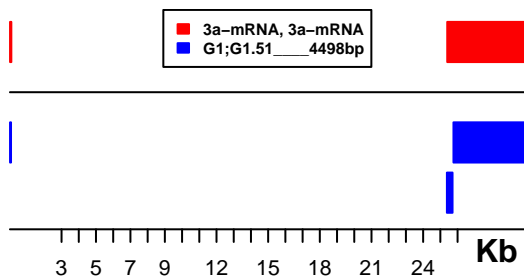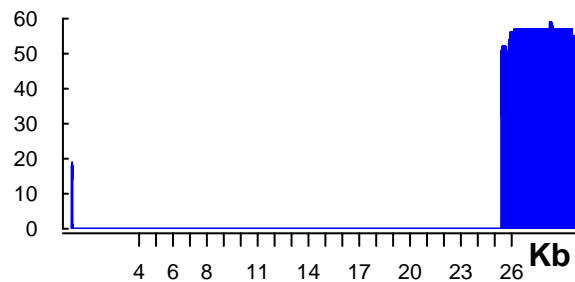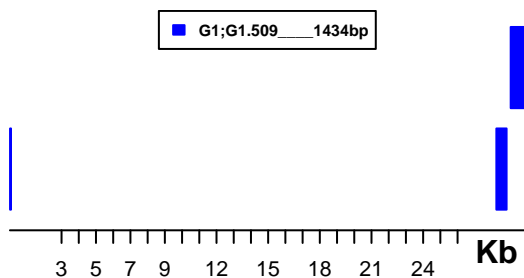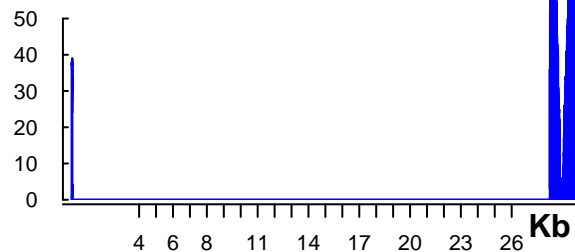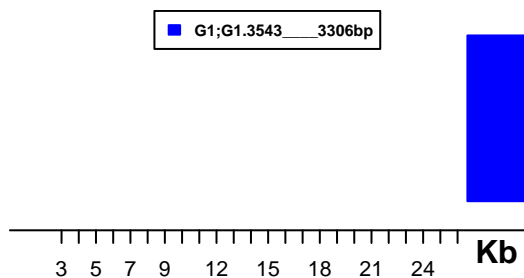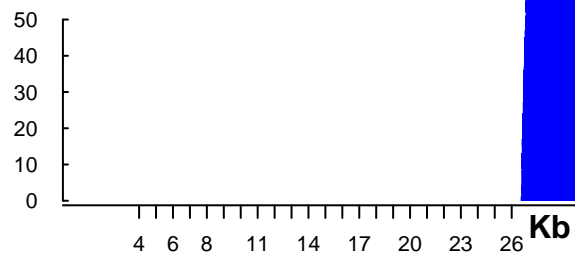

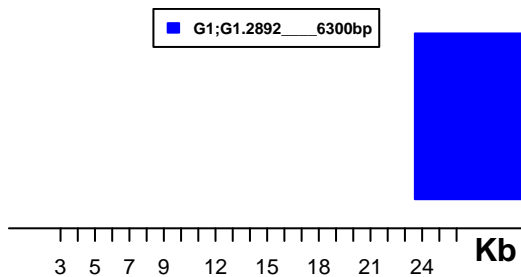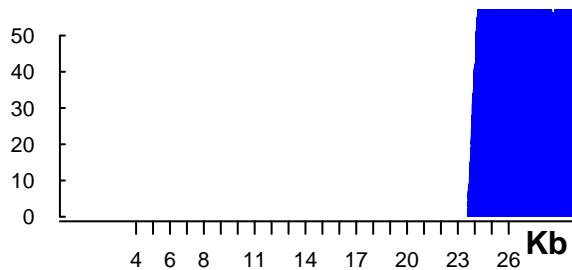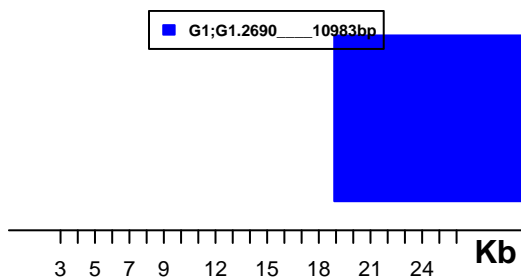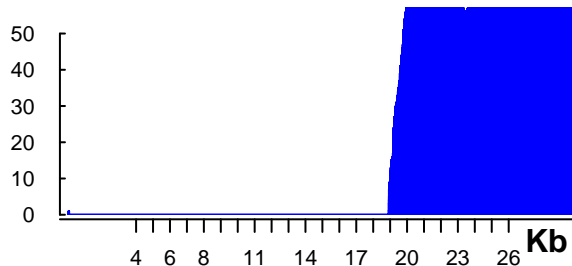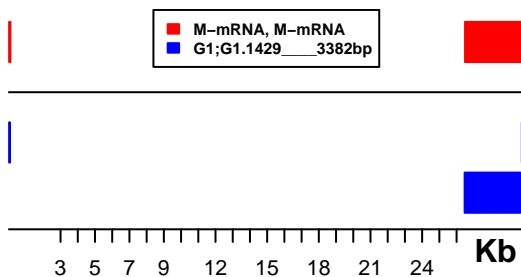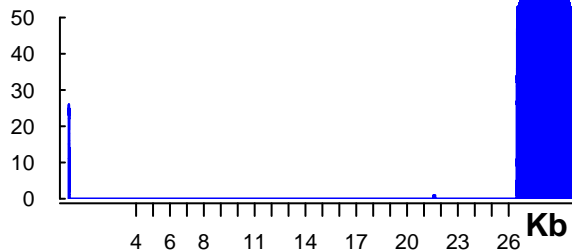

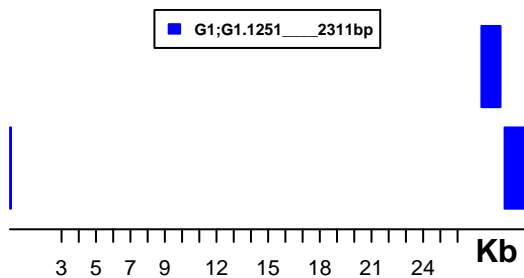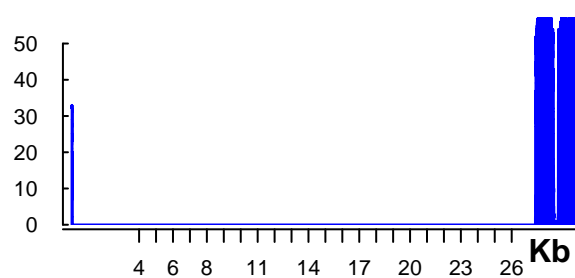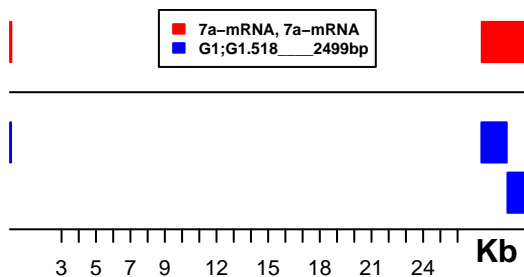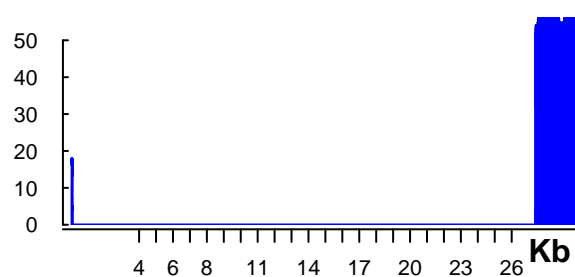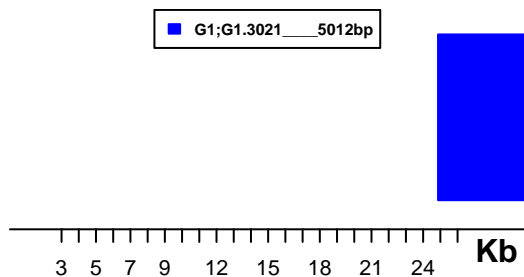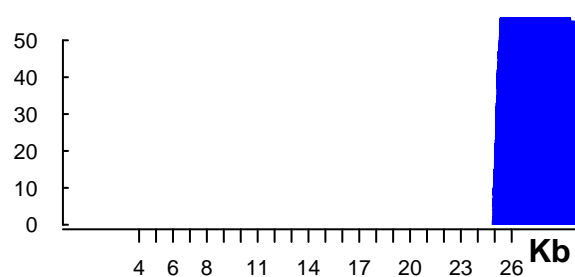

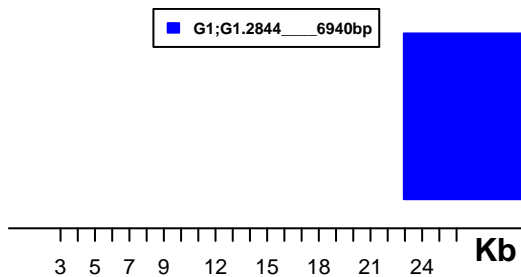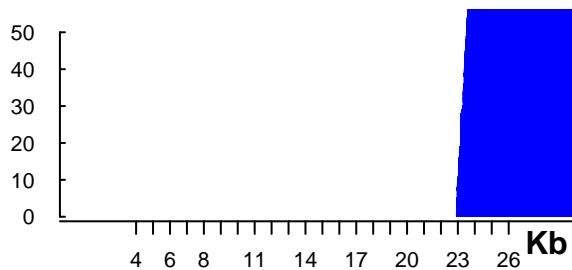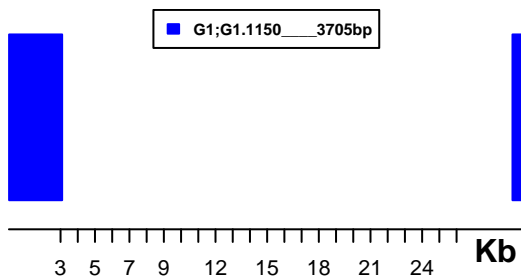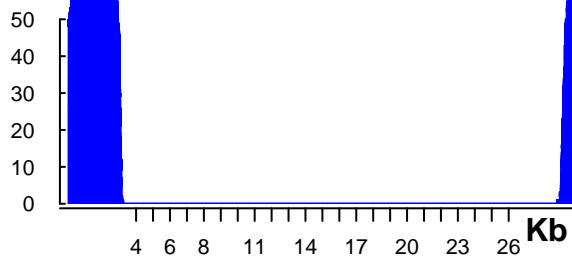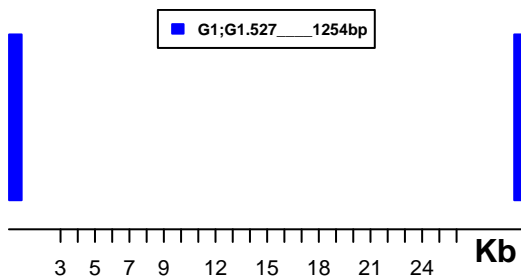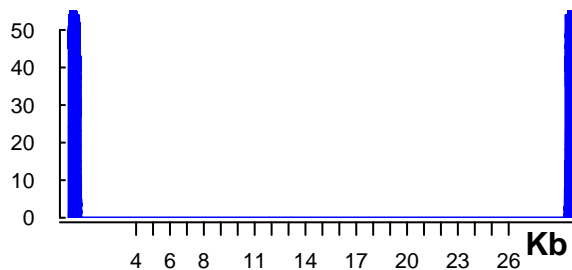

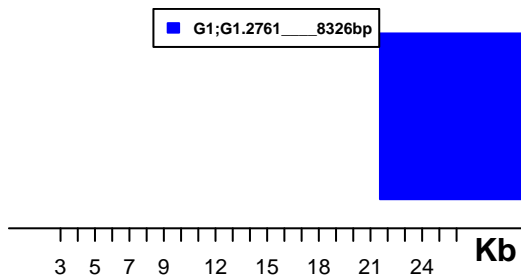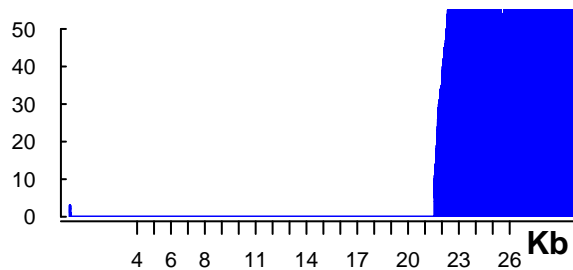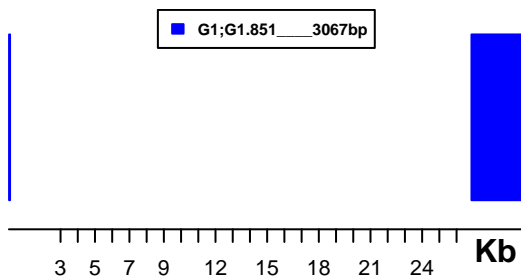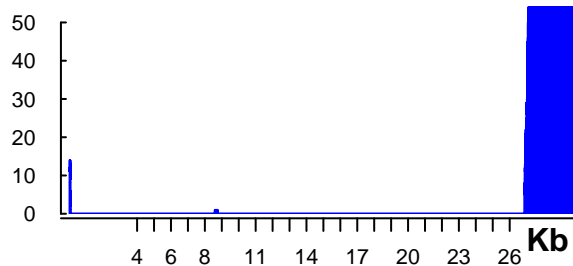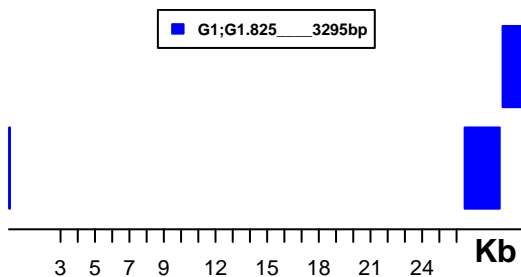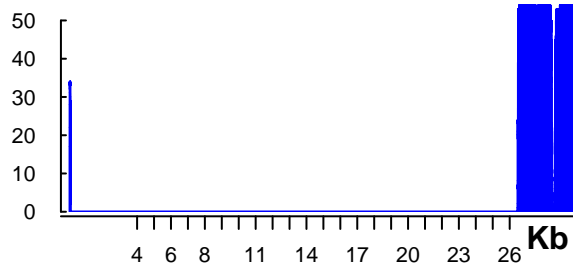

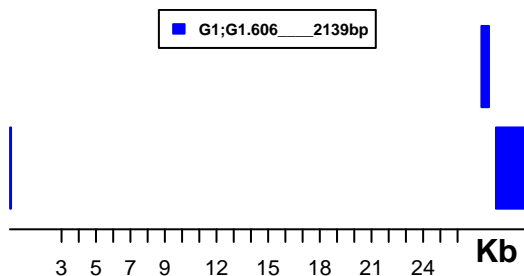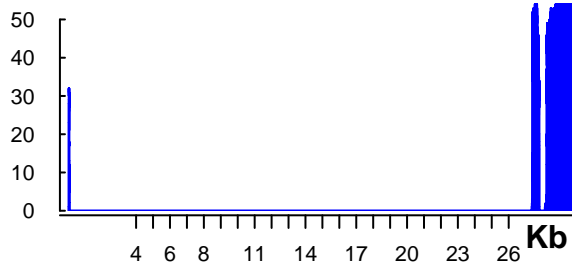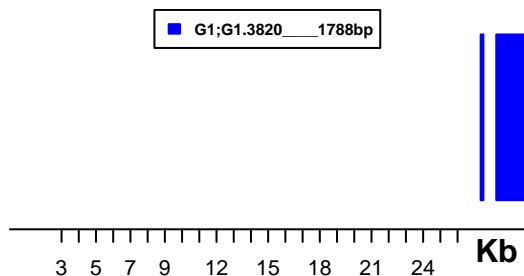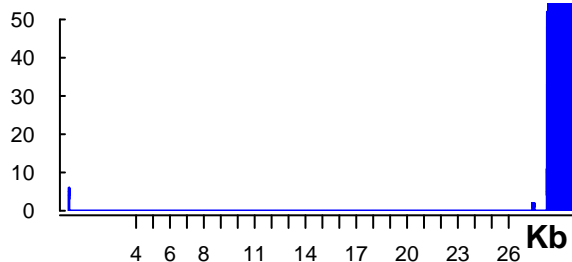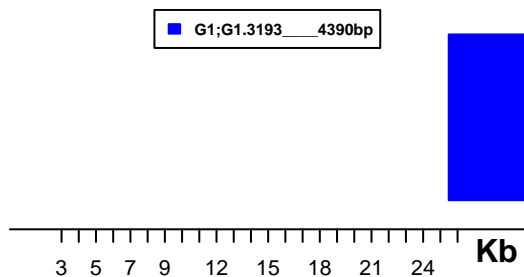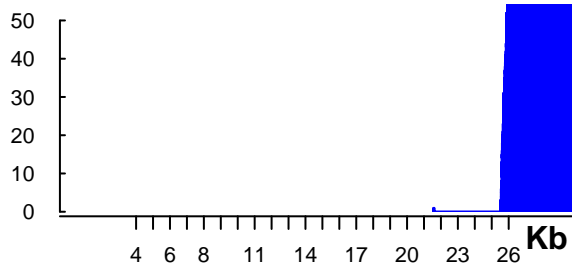

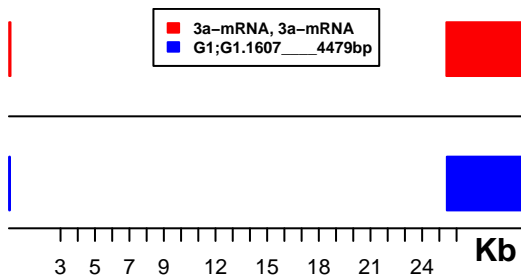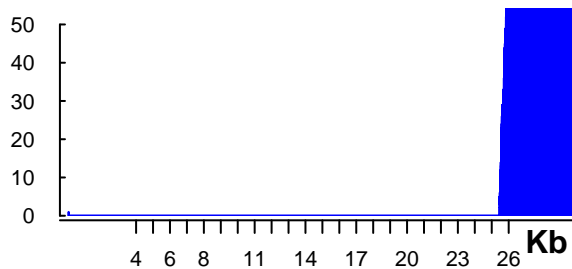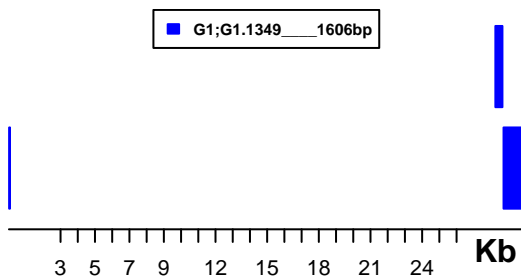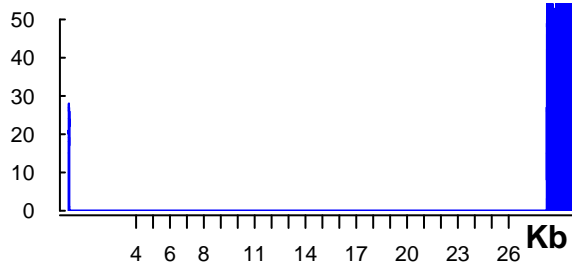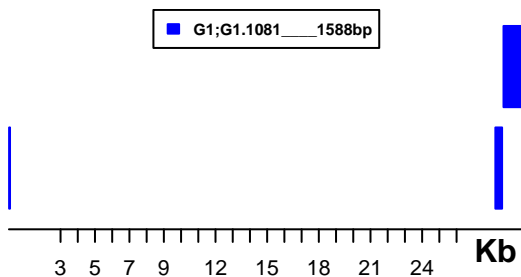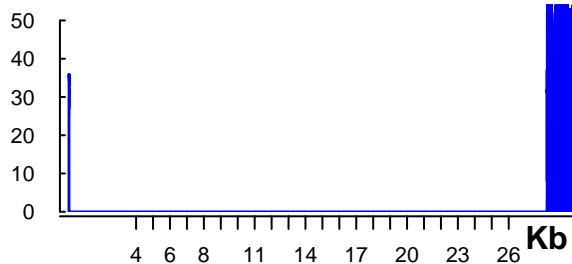

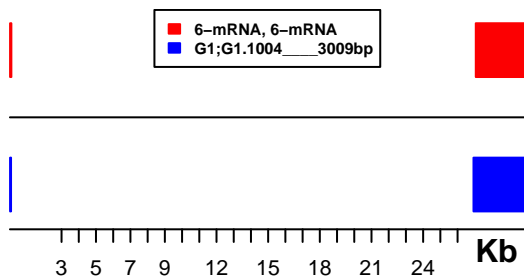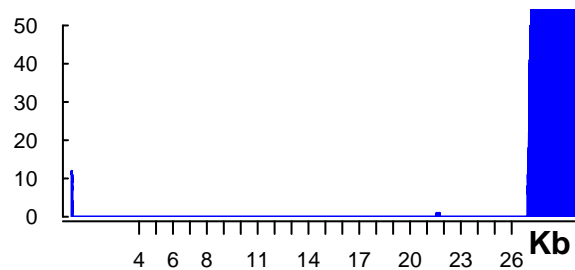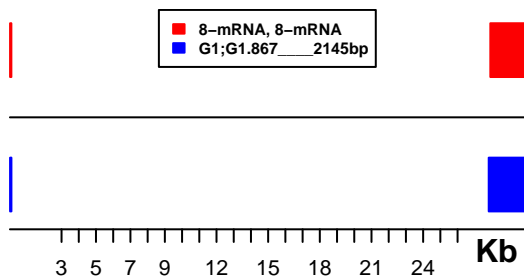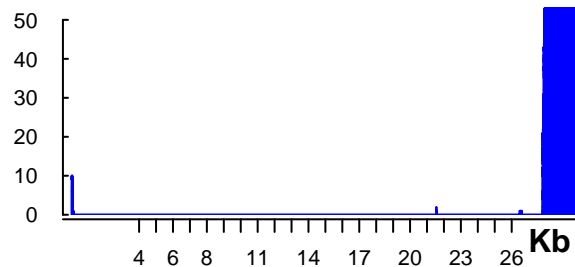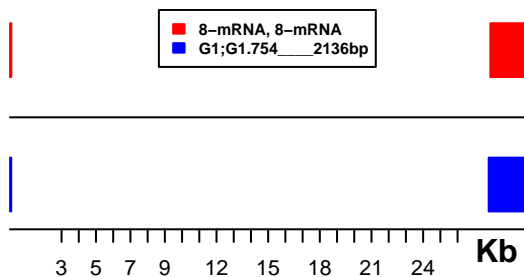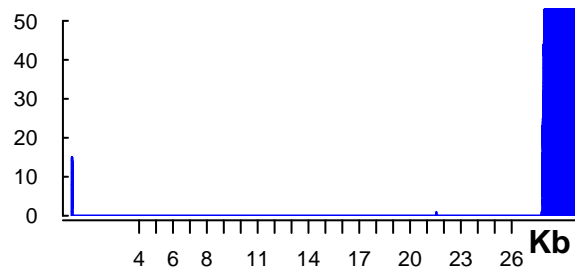

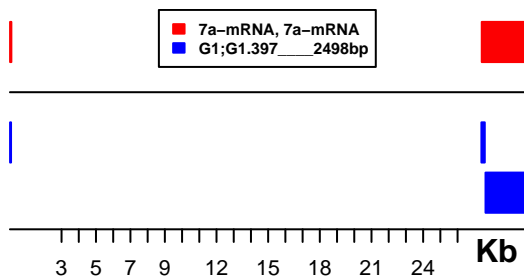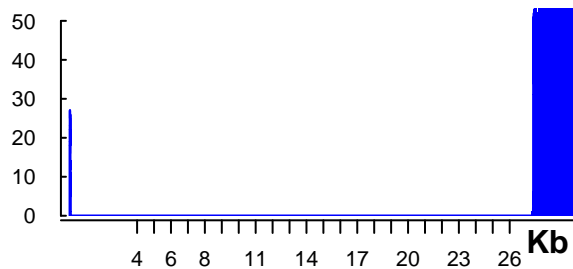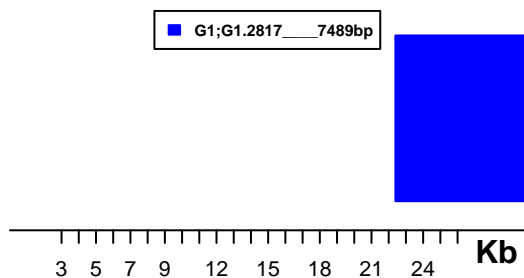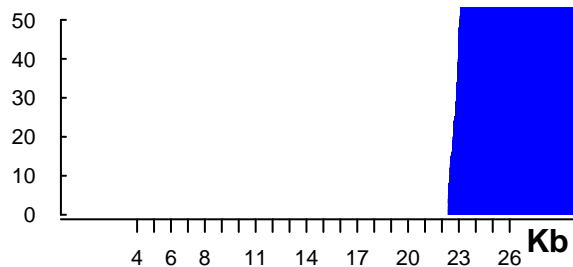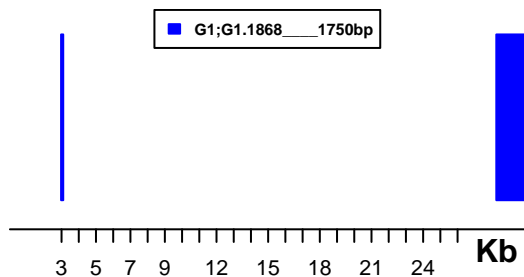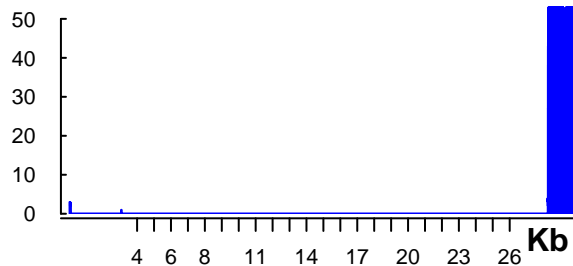

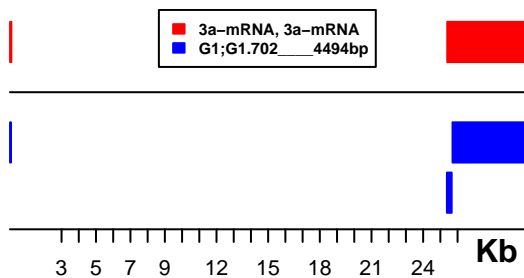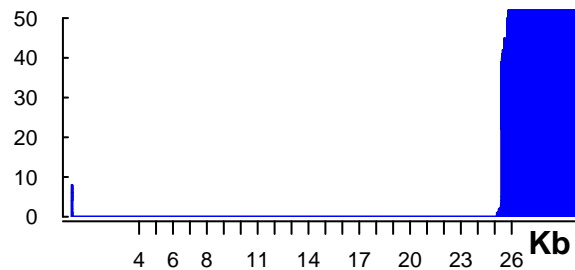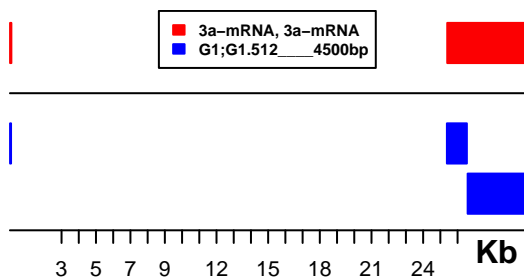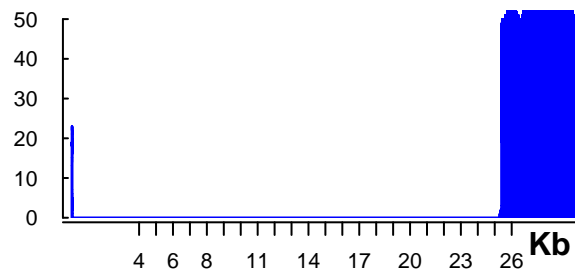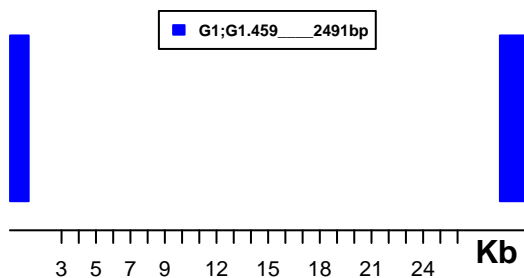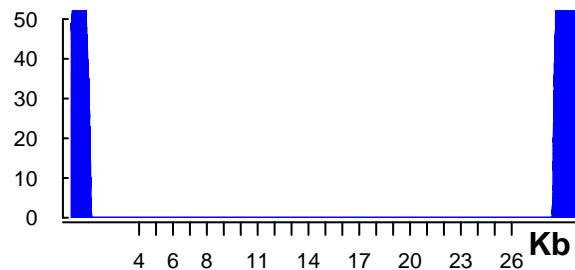

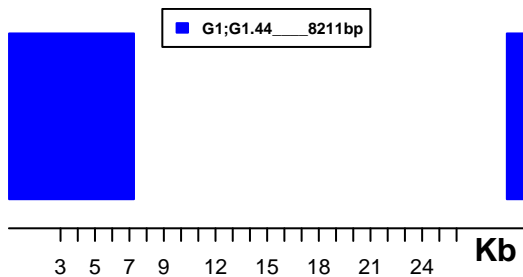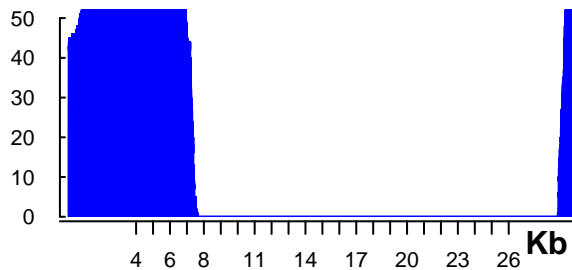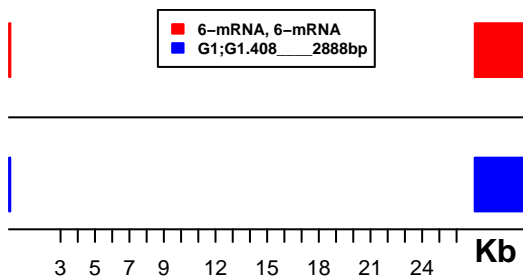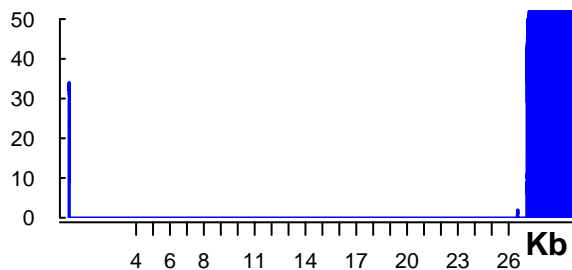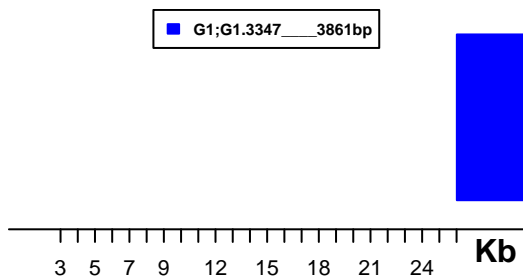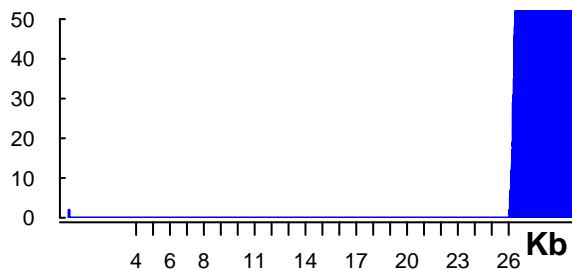

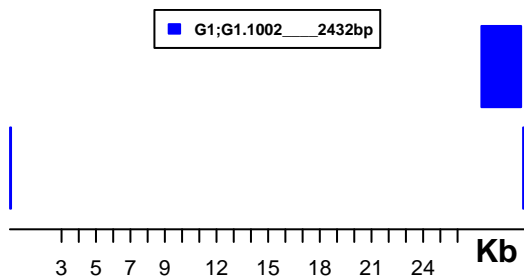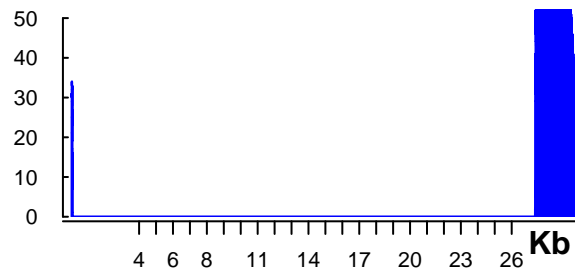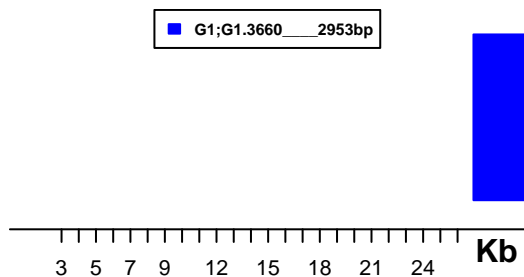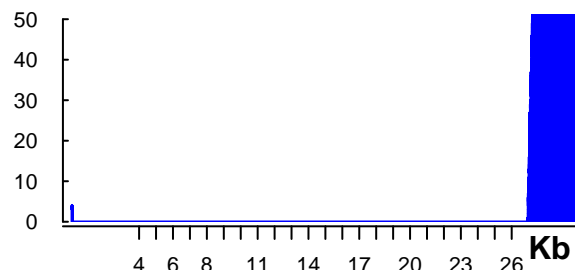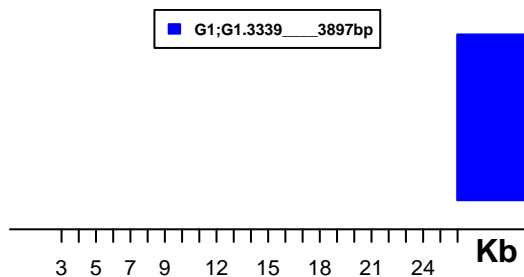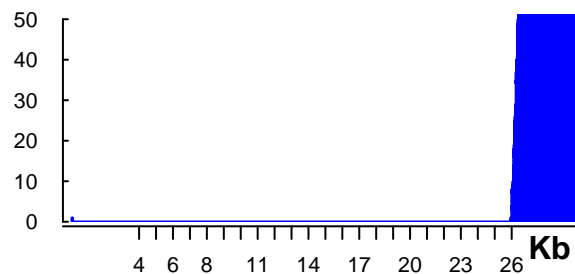

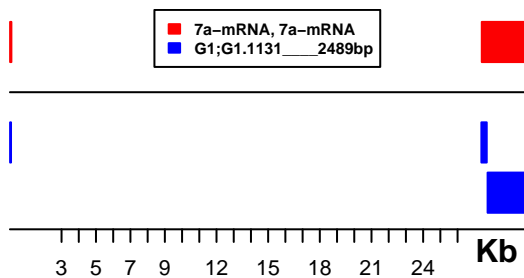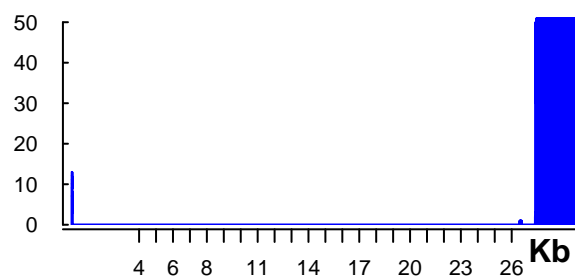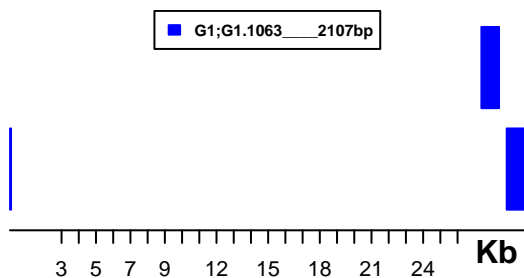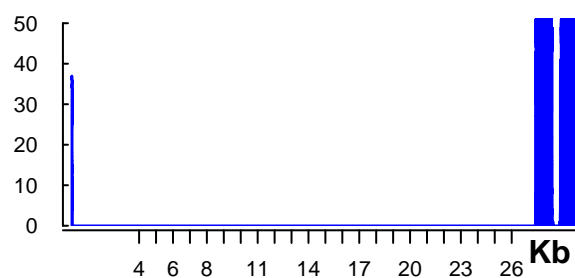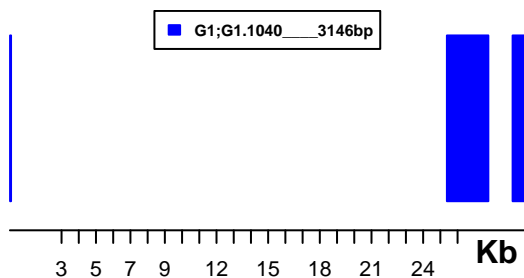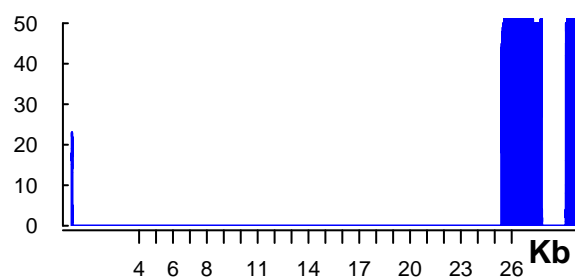

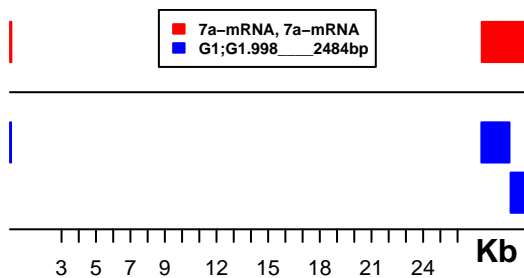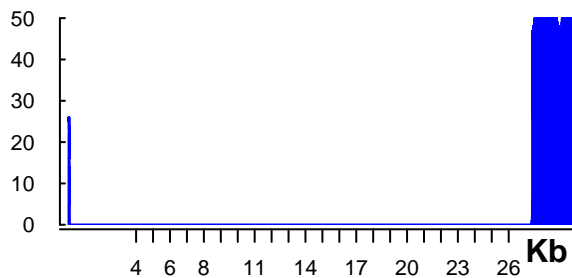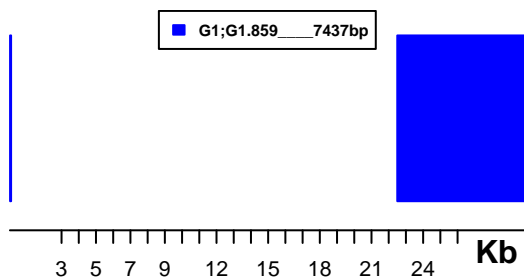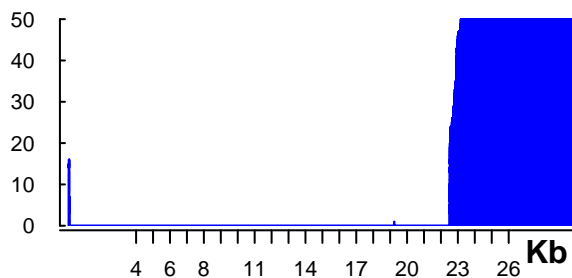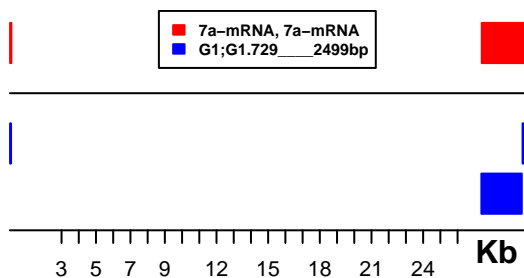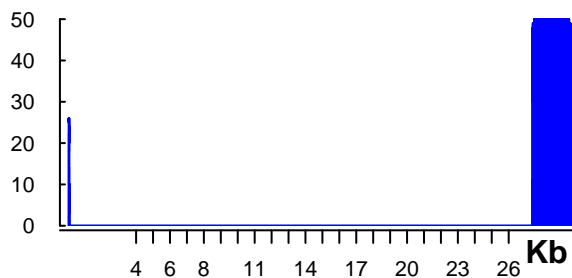

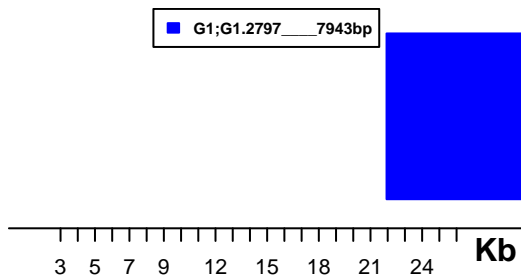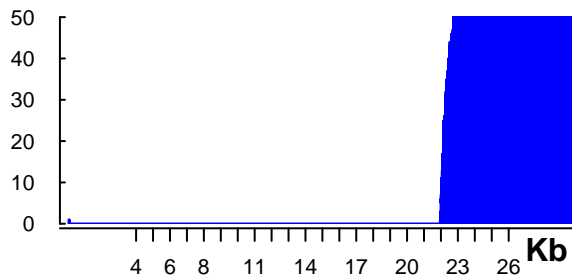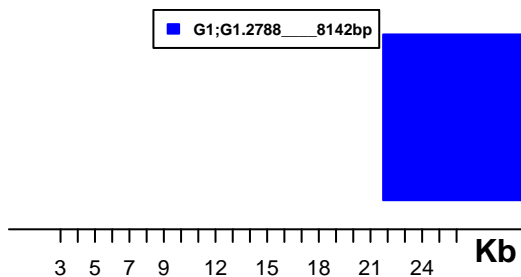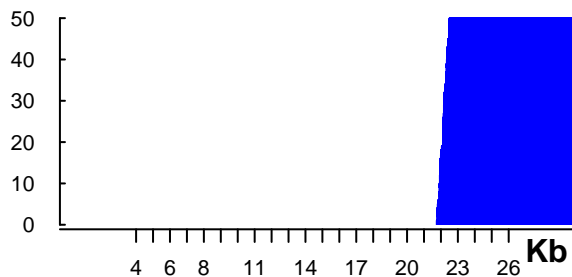

Supplement: Supplementary file 7 — Supplementary Data 5 [file 42003_2022_4058_MOESM7_ESM.zip › experiment1/Files_used_for_the_analysis_of_the_manuscript_experiment1/RNA_MODELS_WITH_COVERAGE_experiment_1_passage_1.pdf]

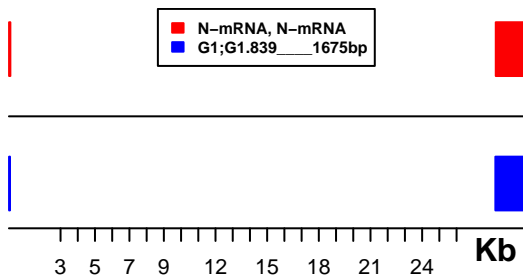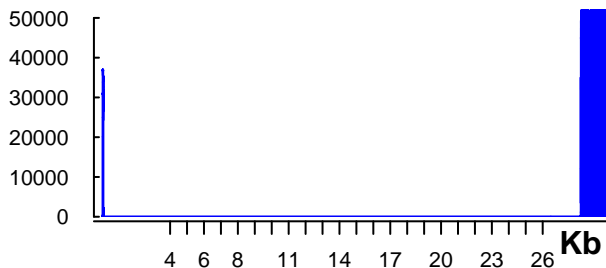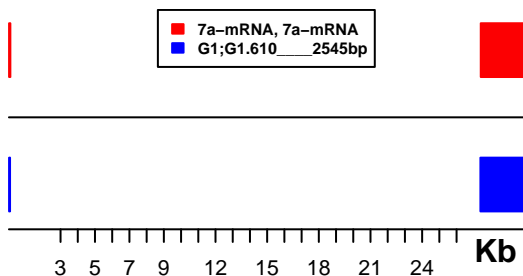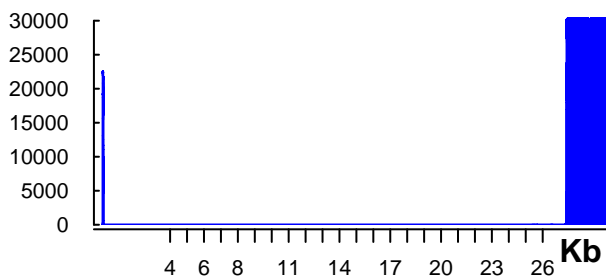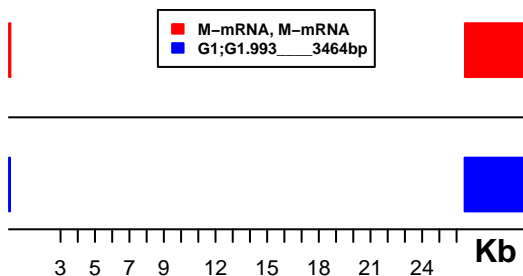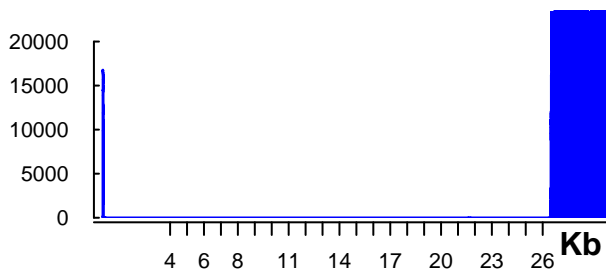

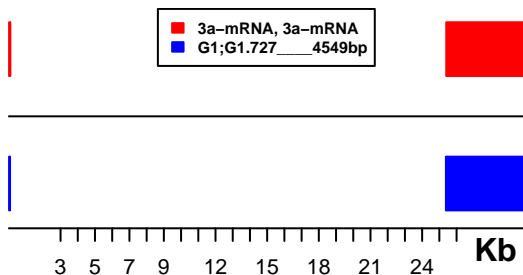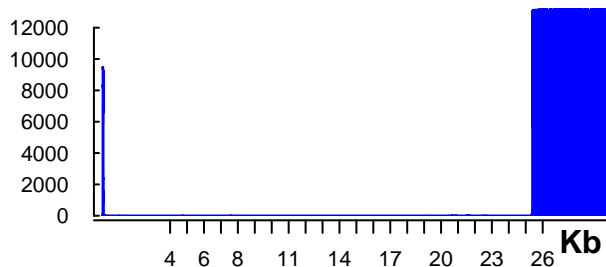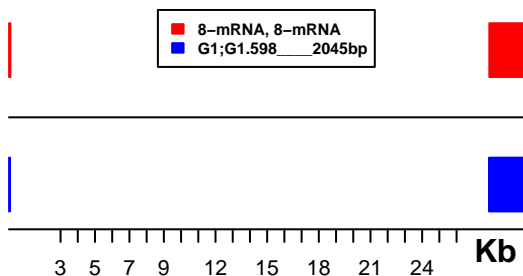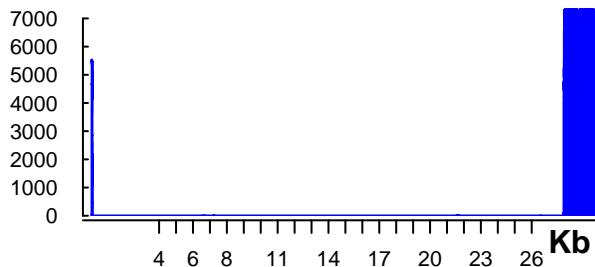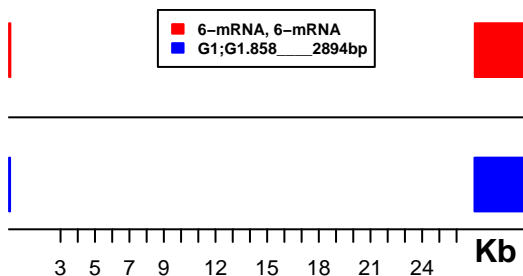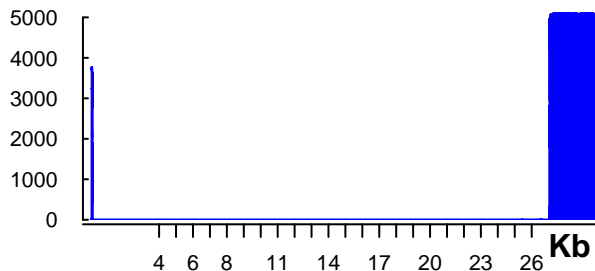

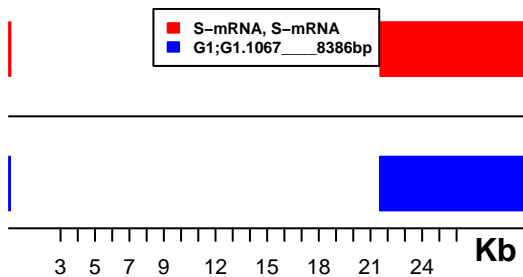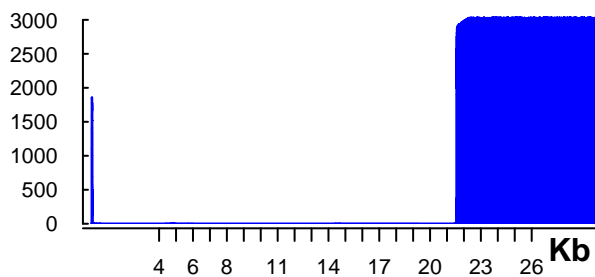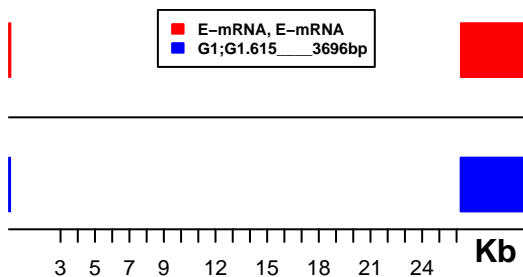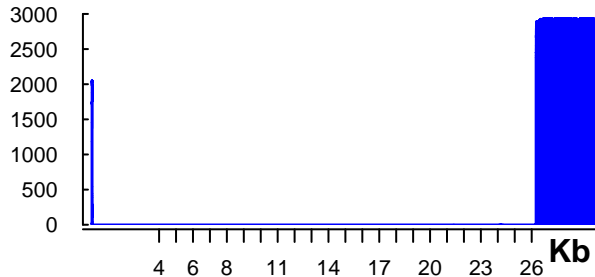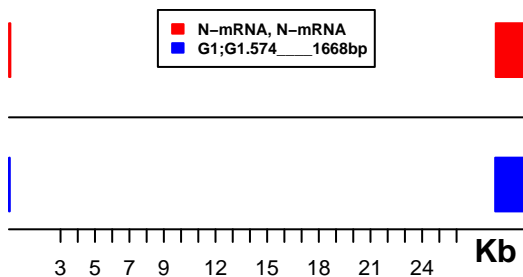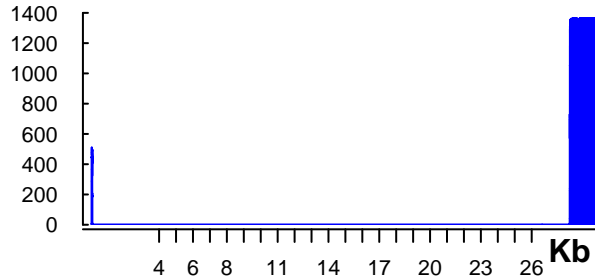

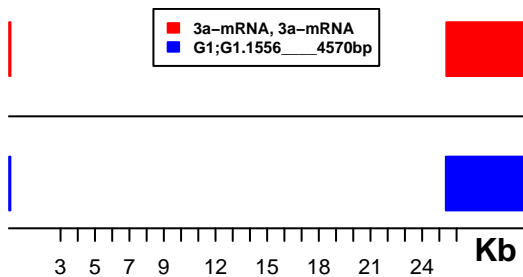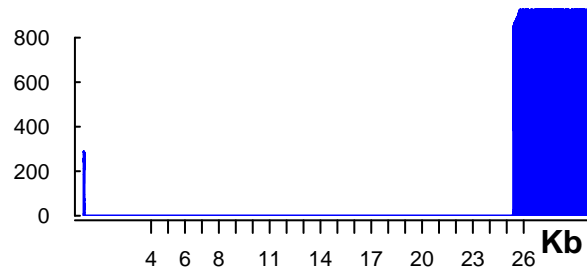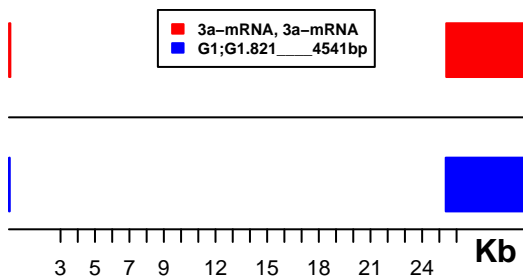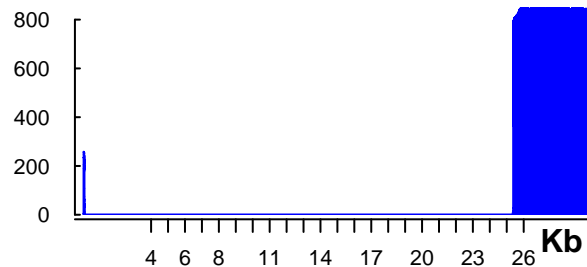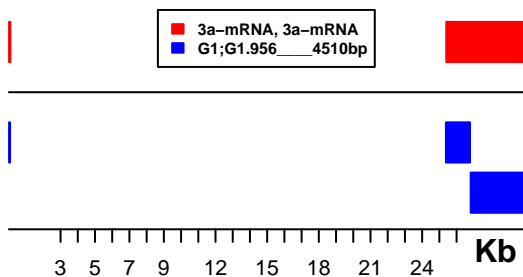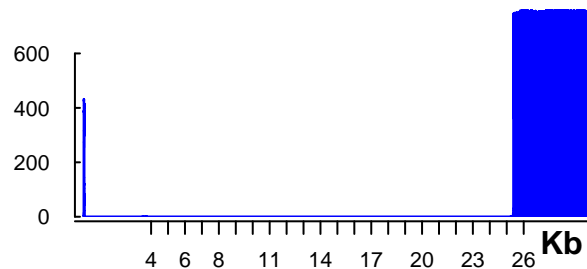

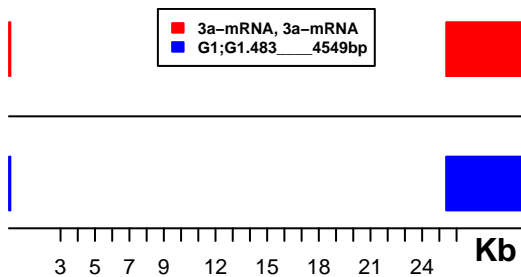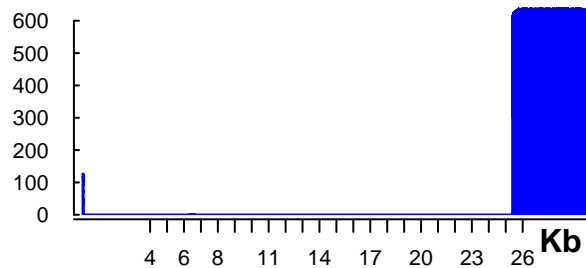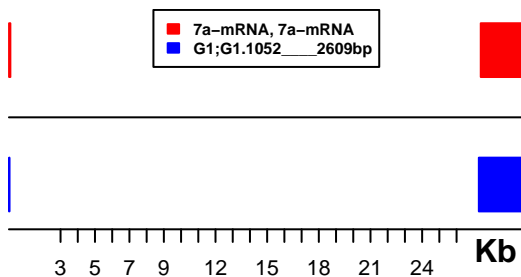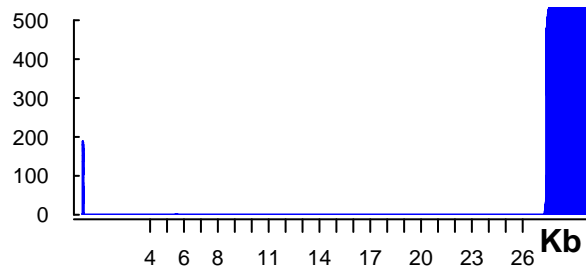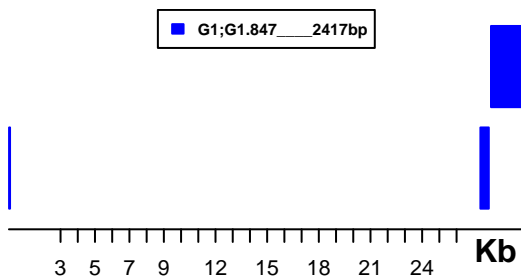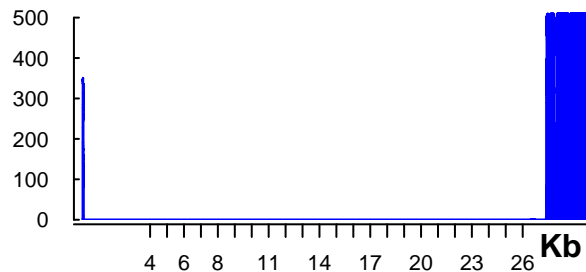

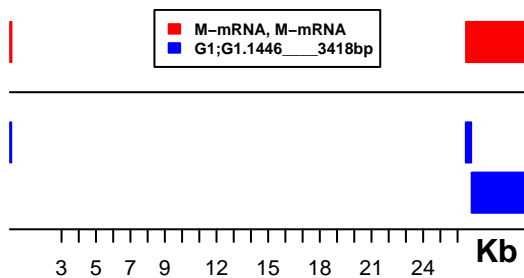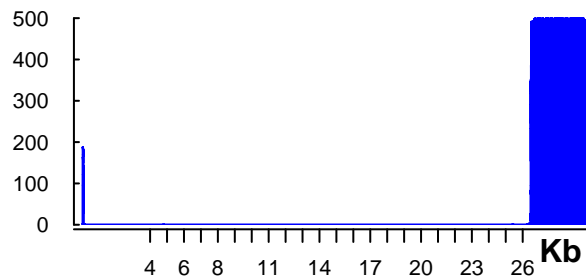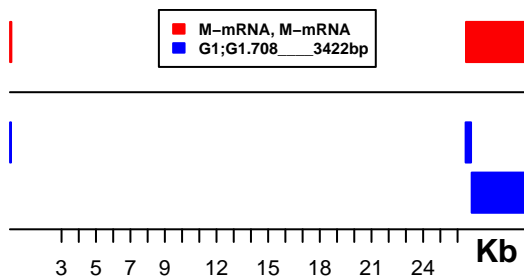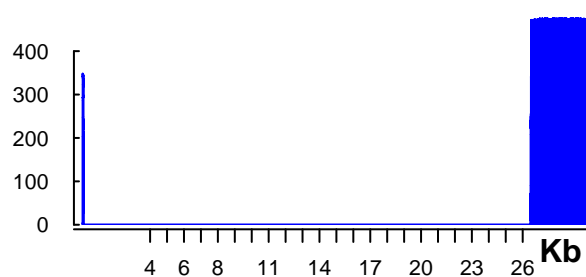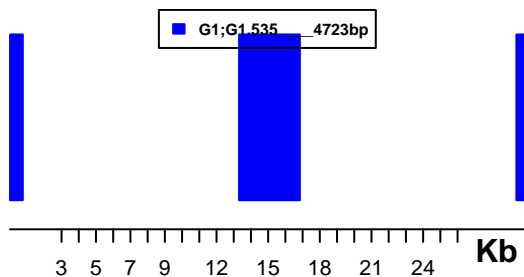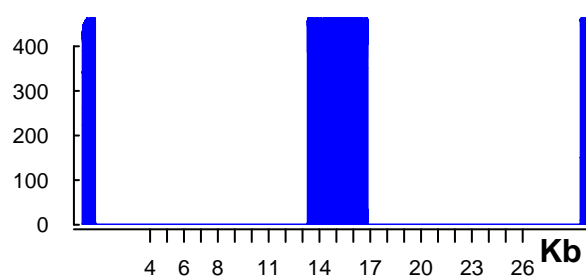

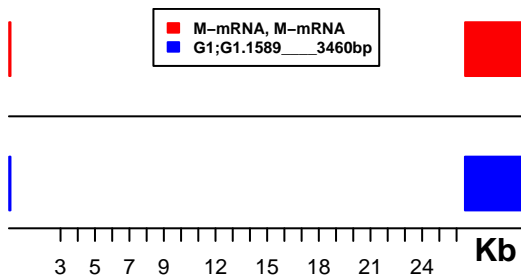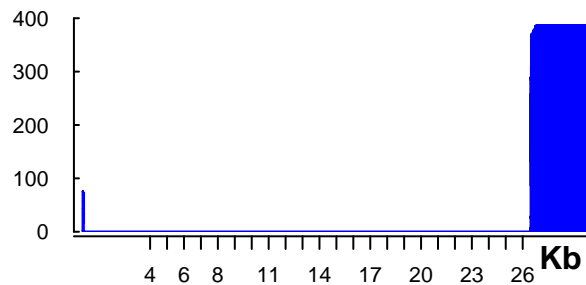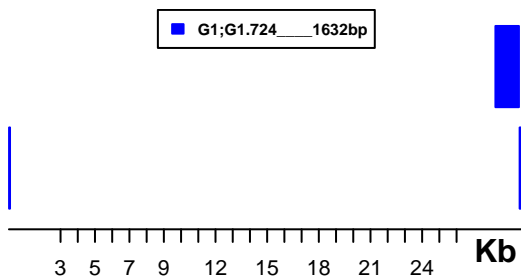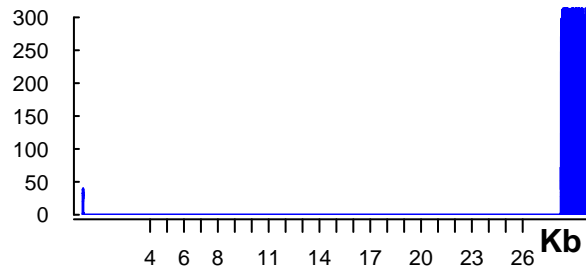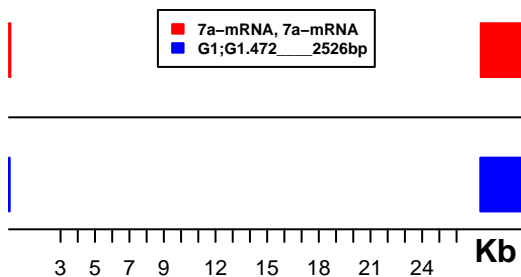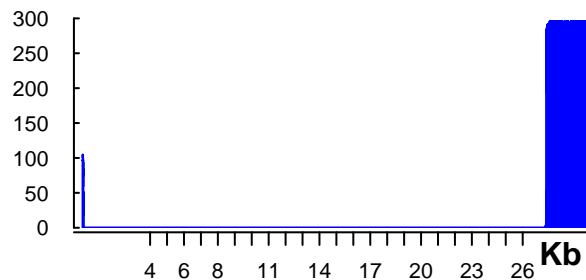

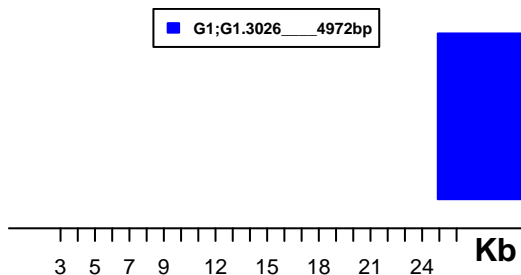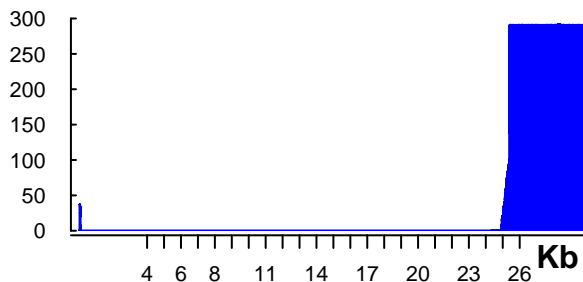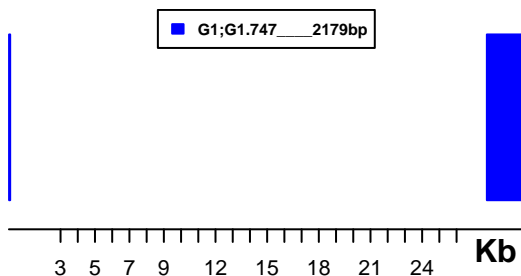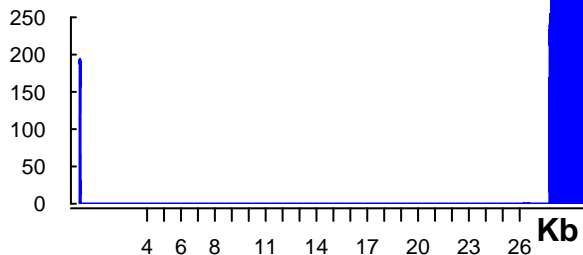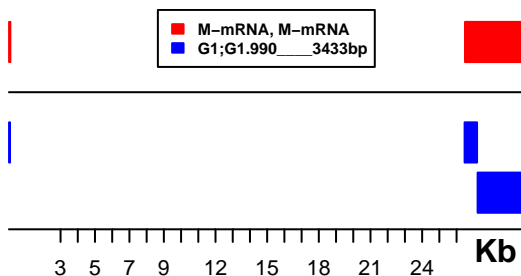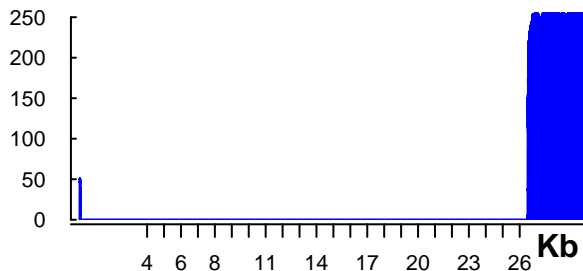

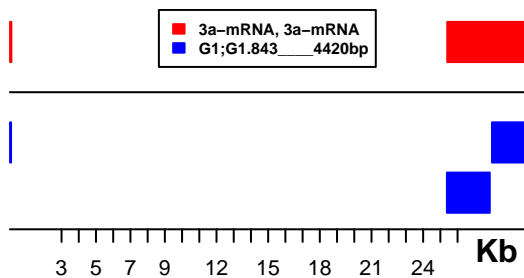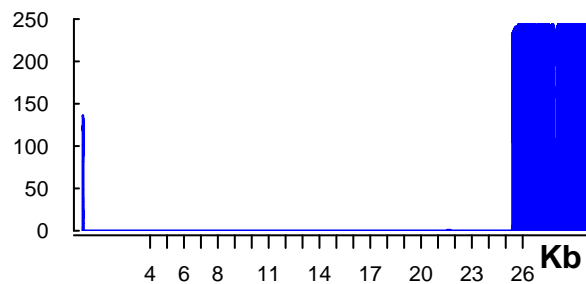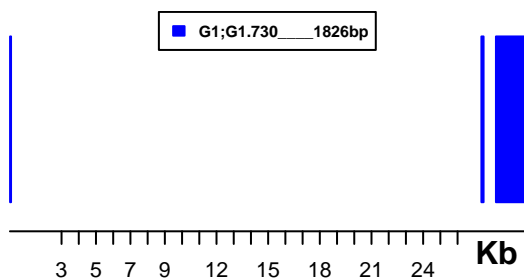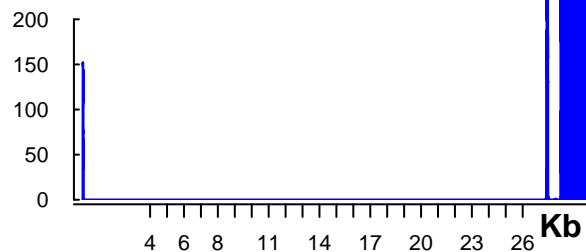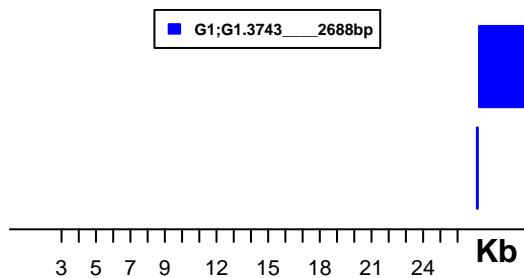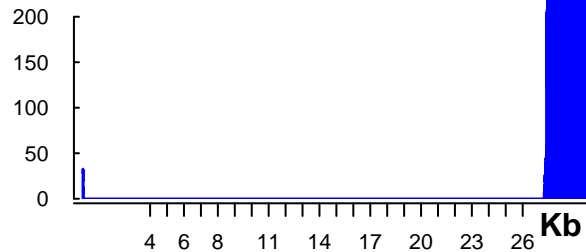

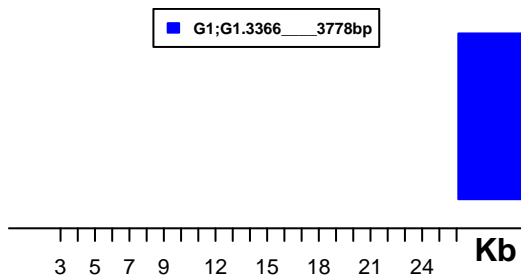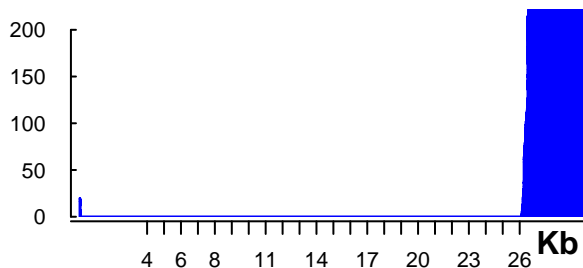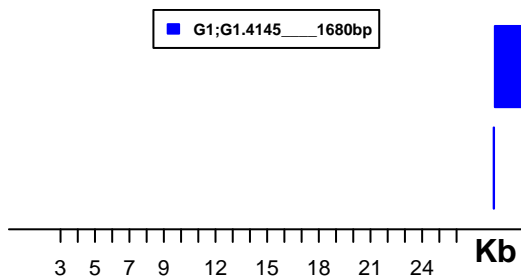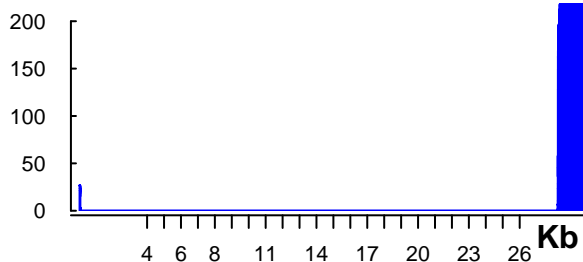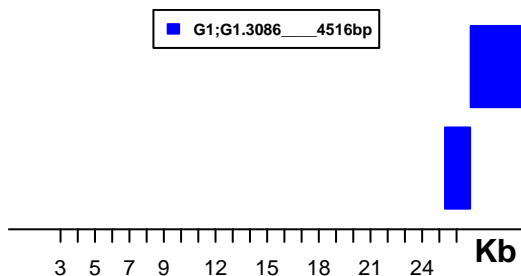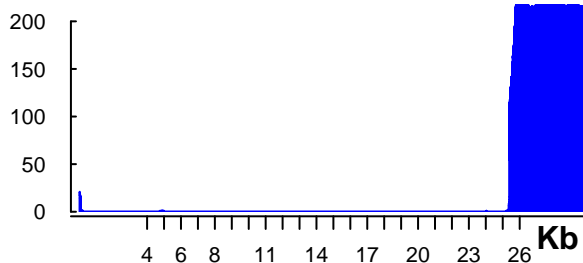

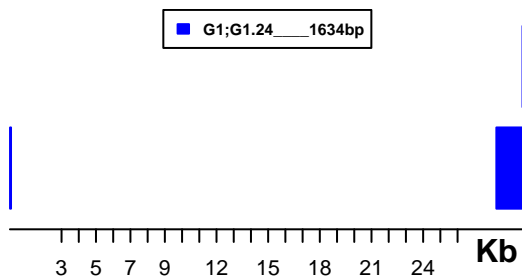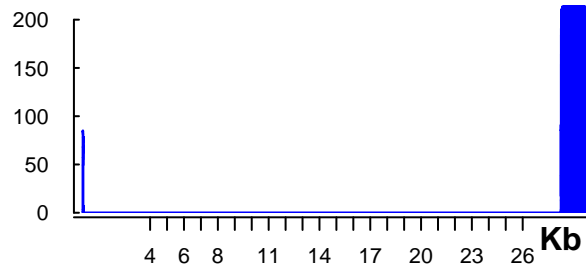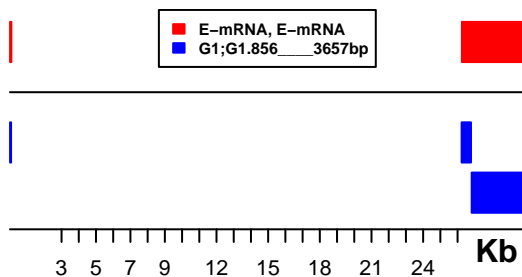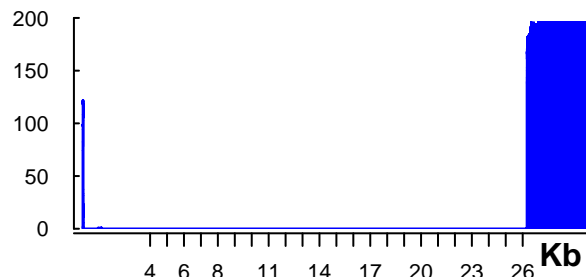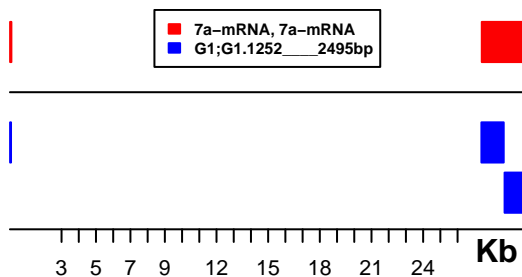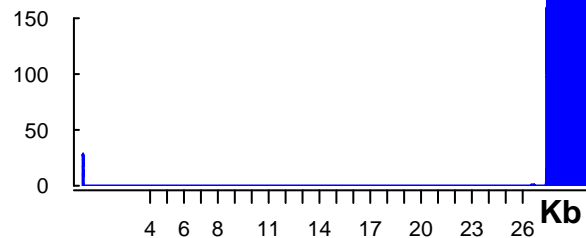

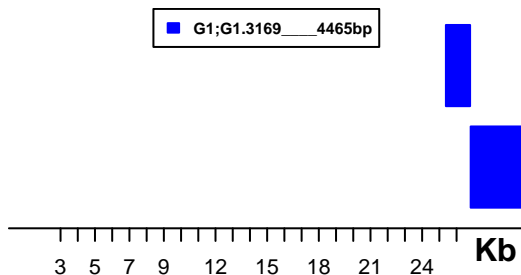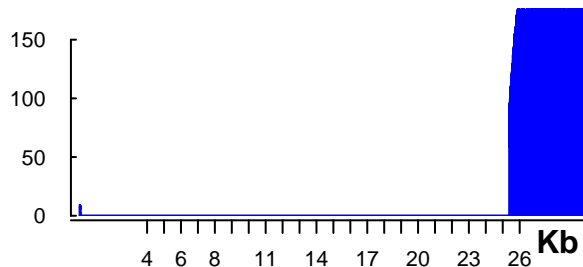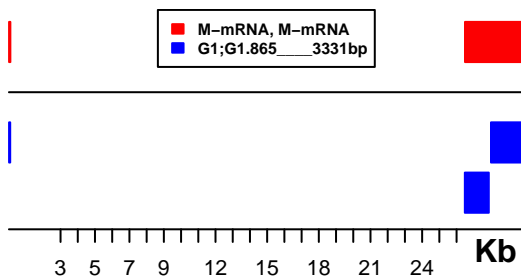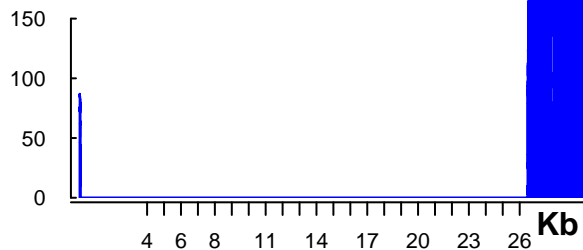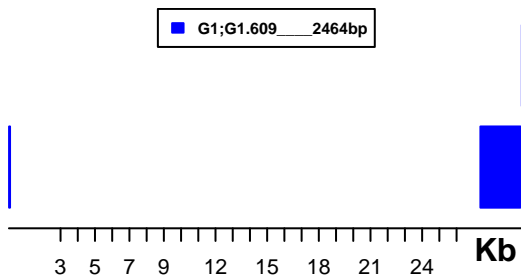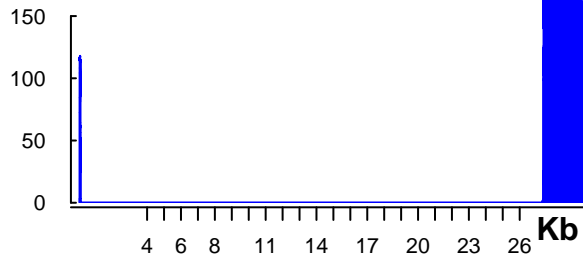

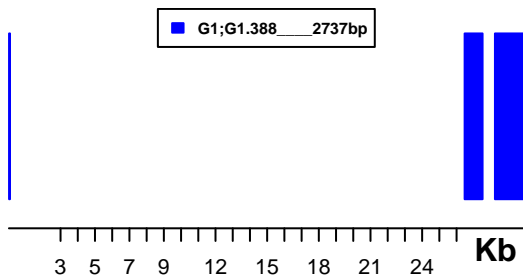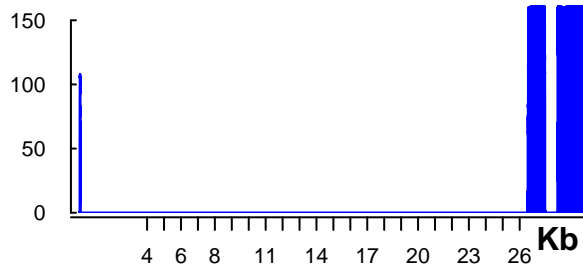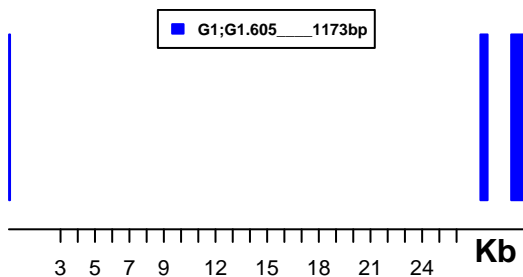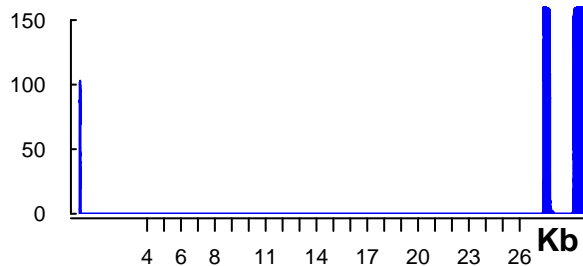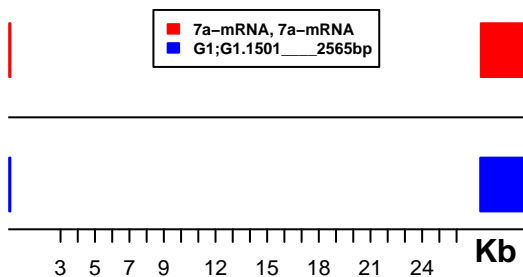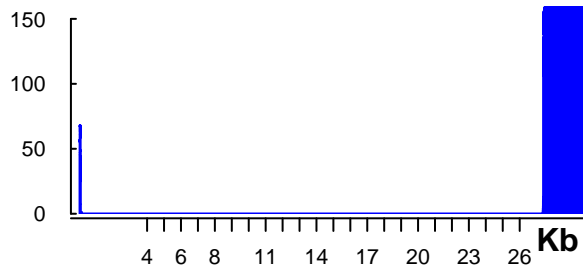

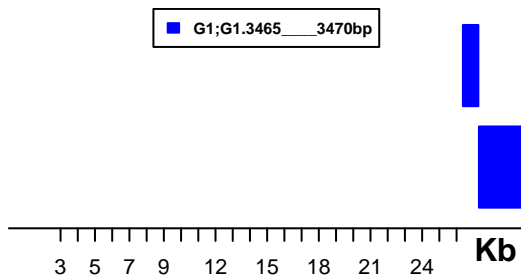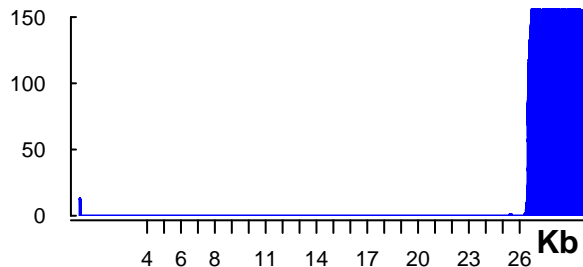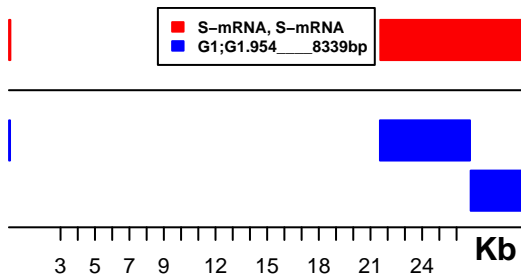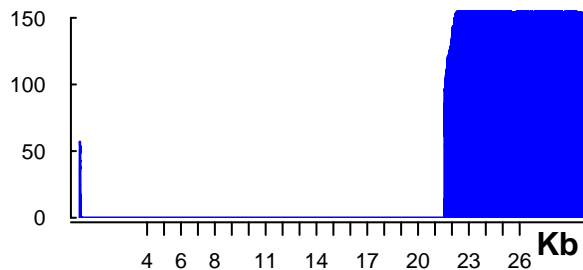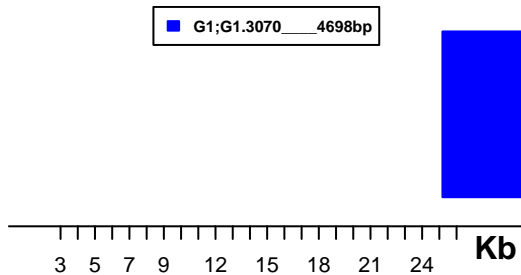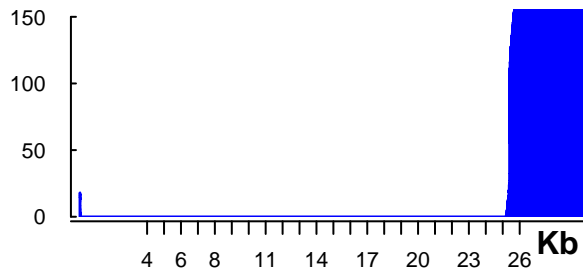

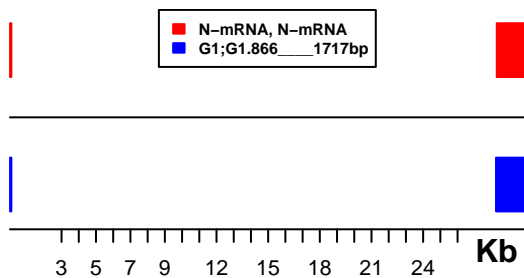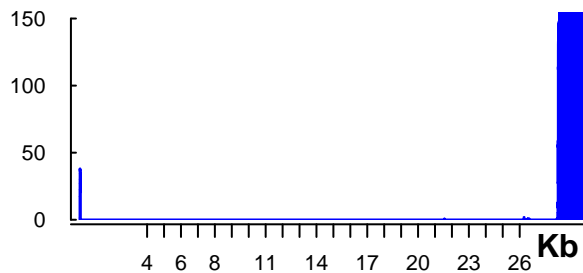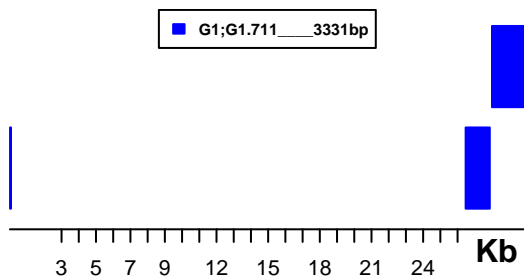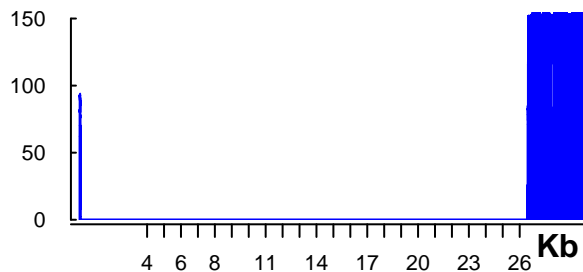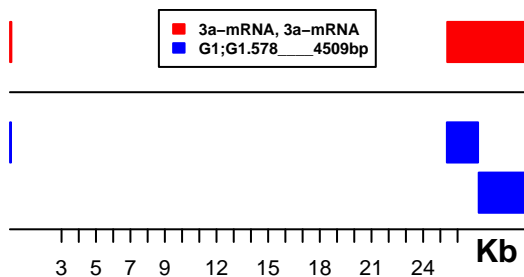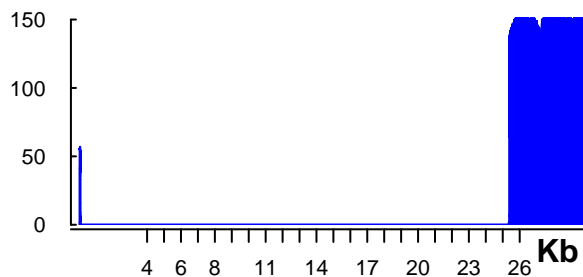

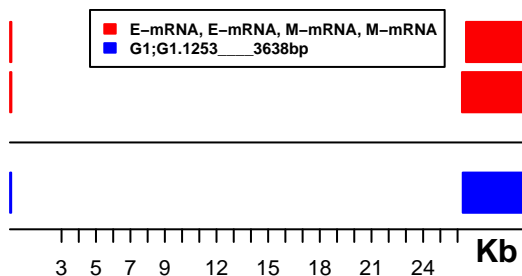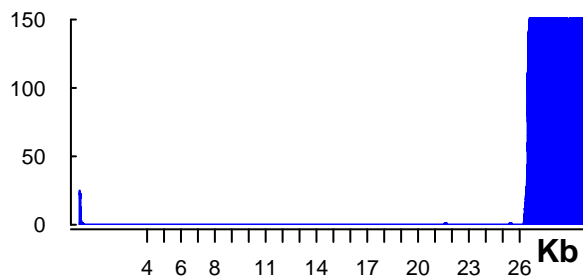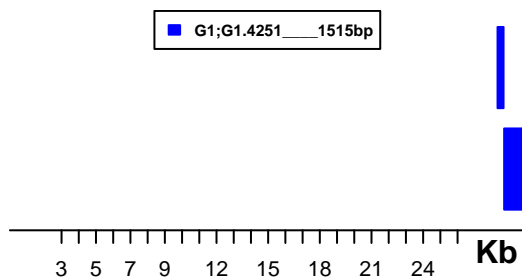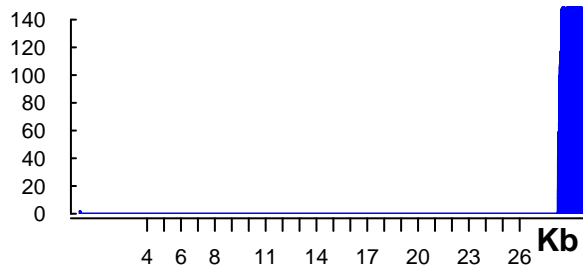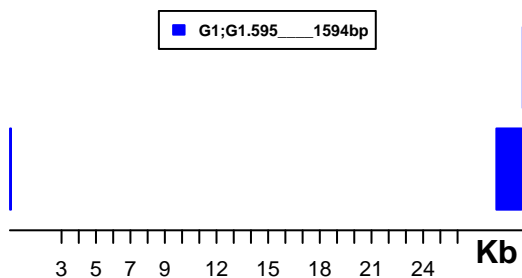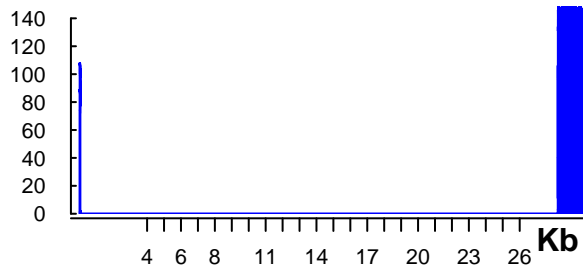

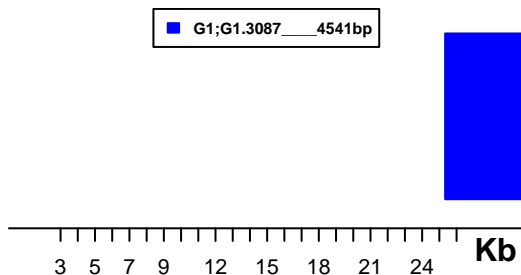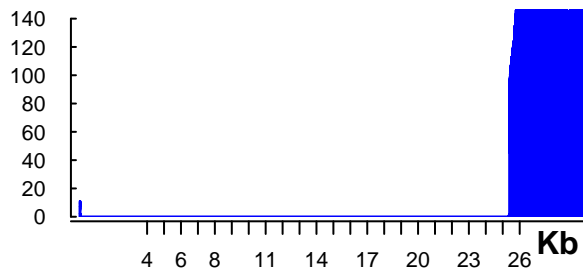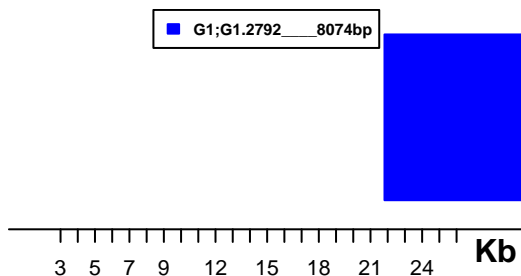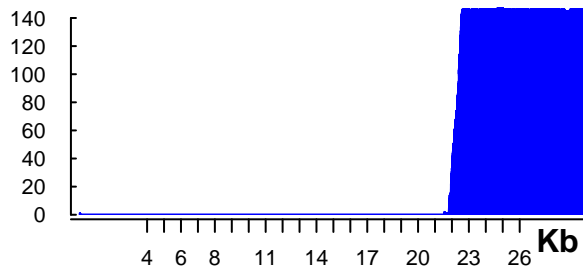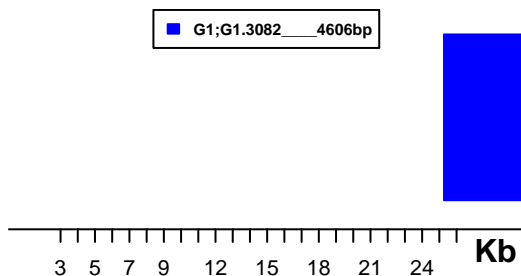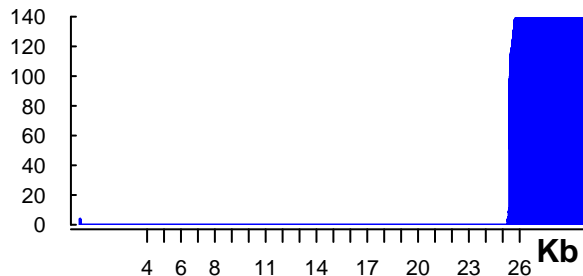

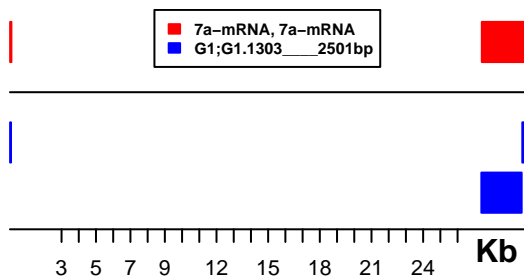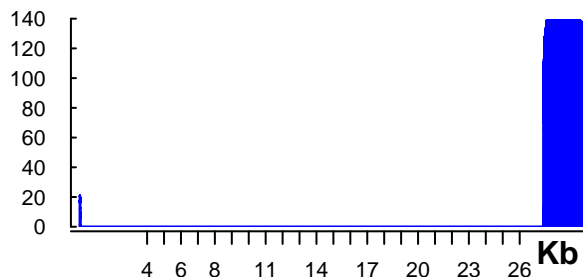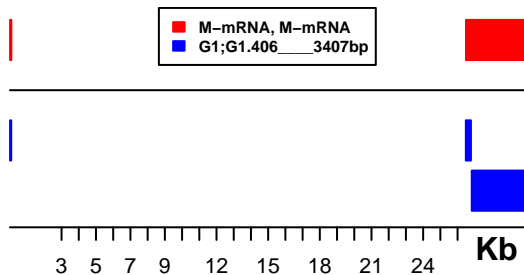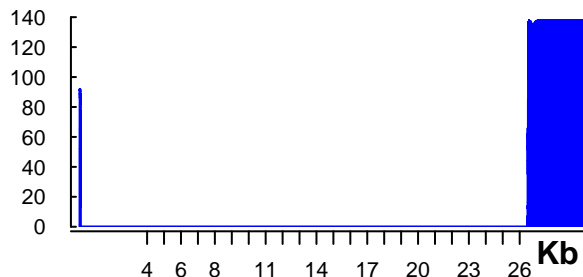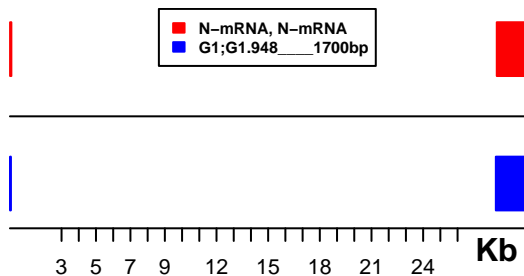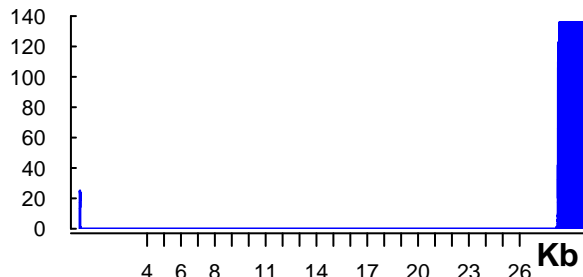

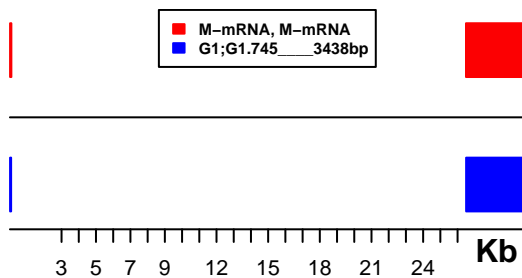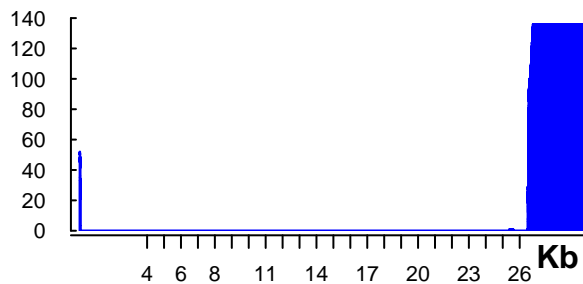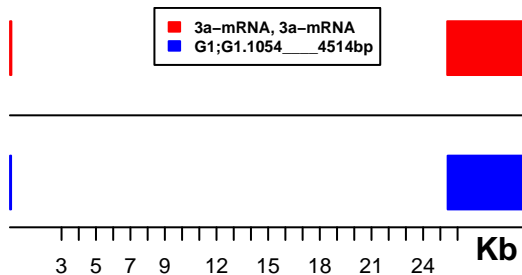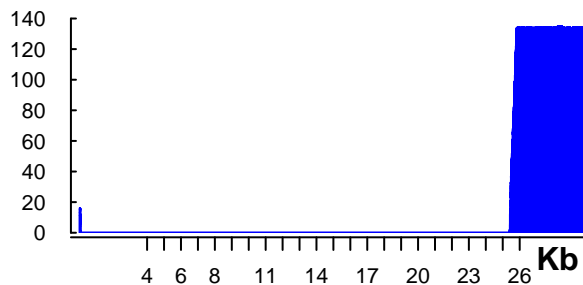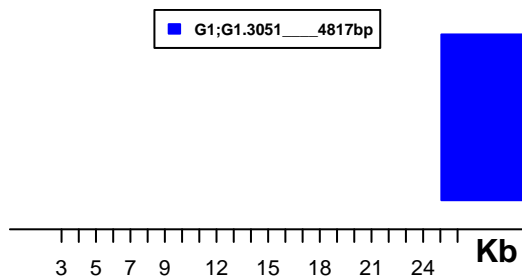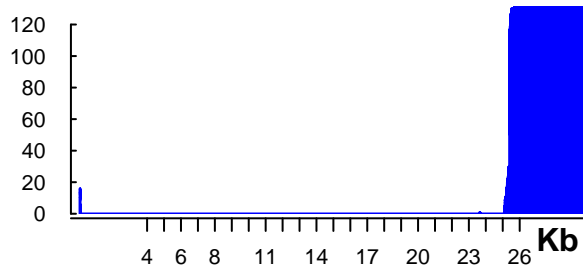

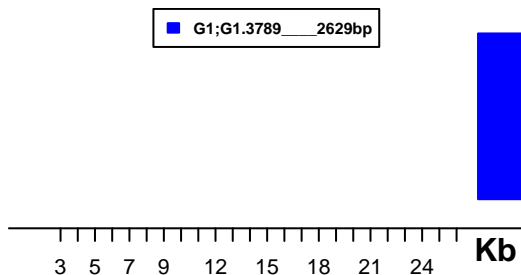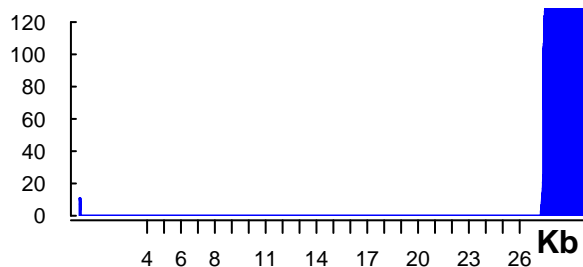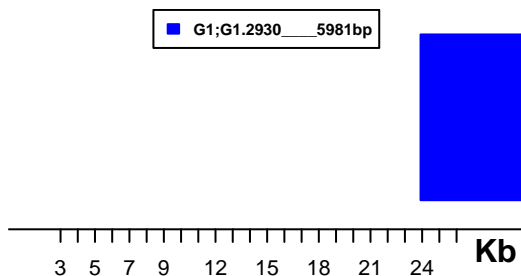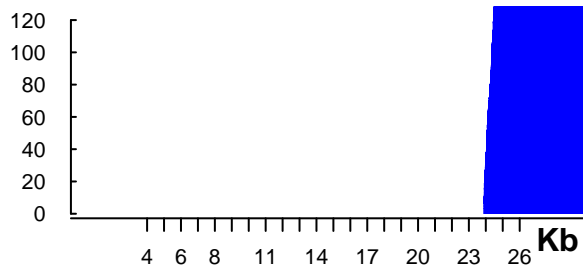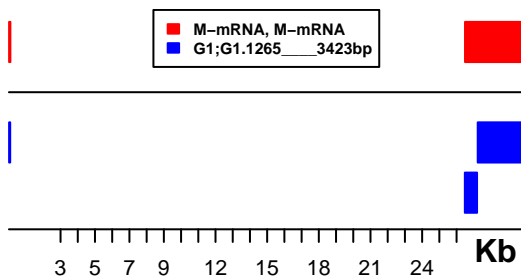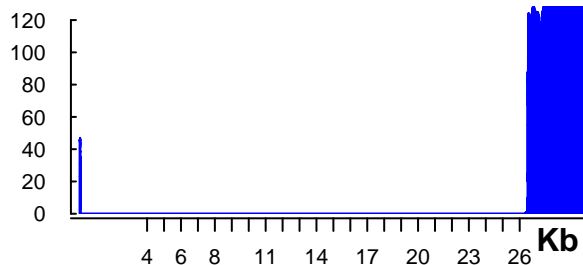

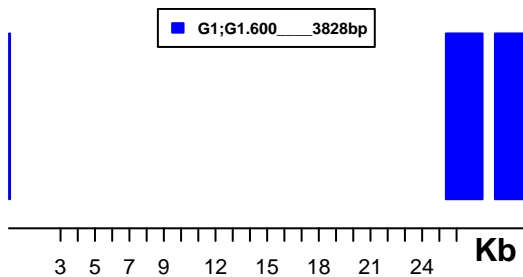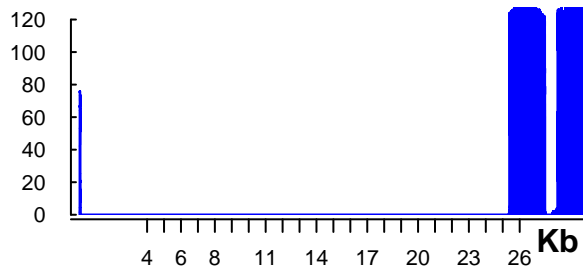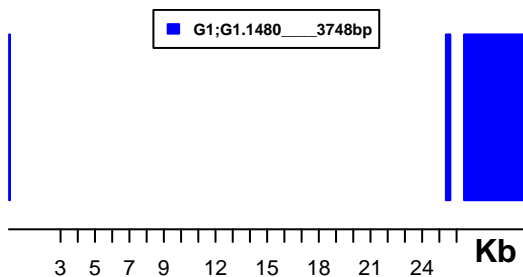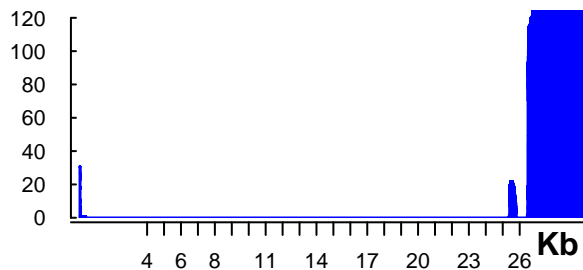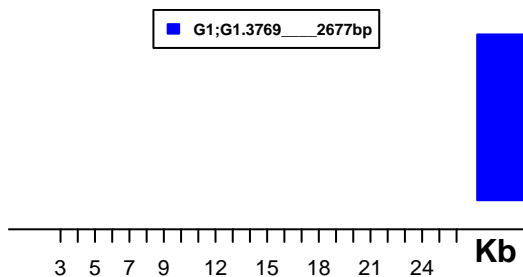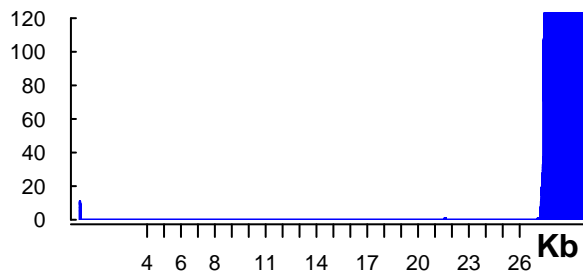

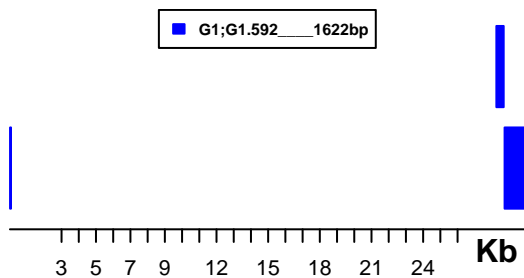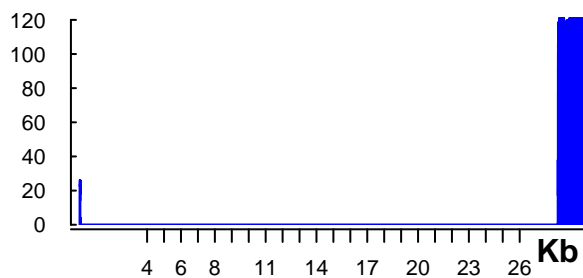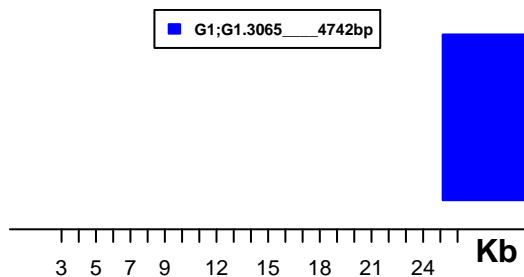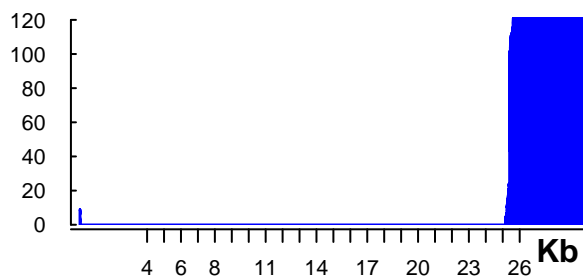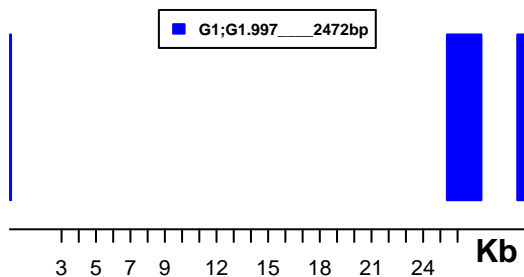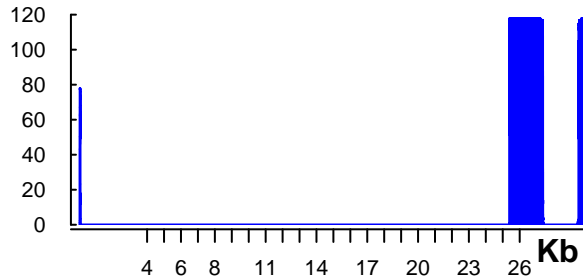

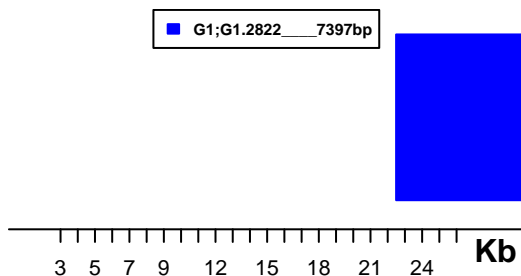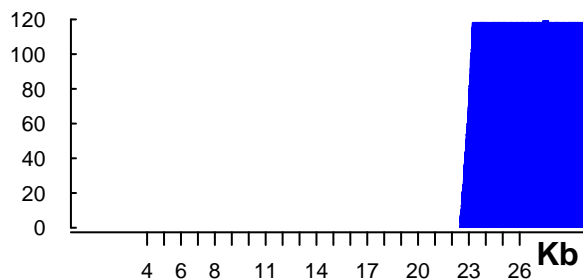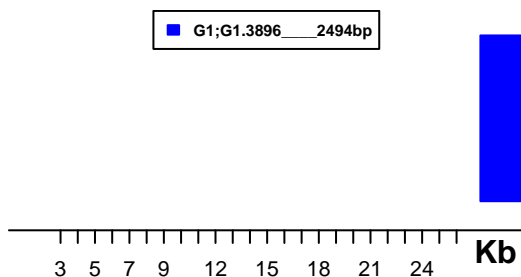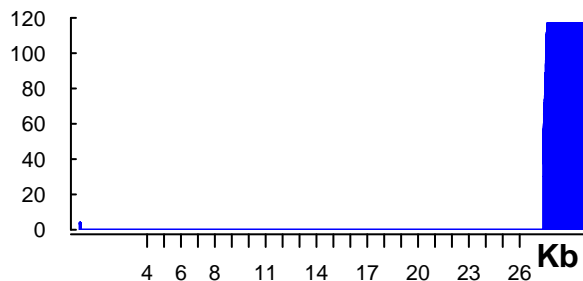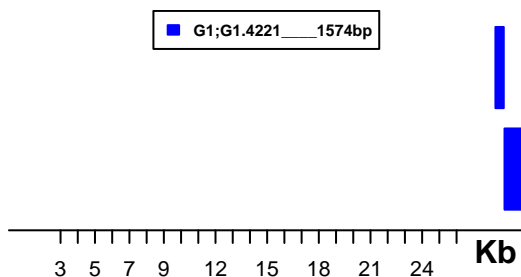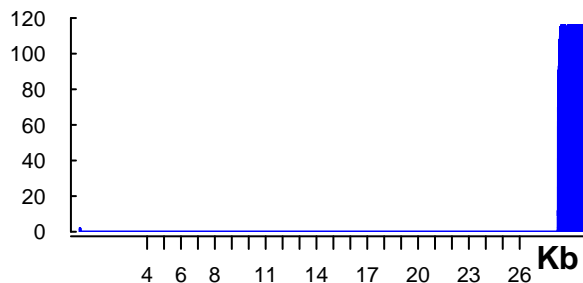

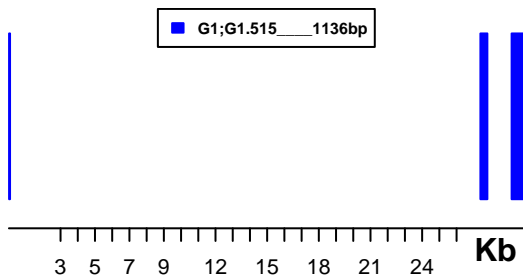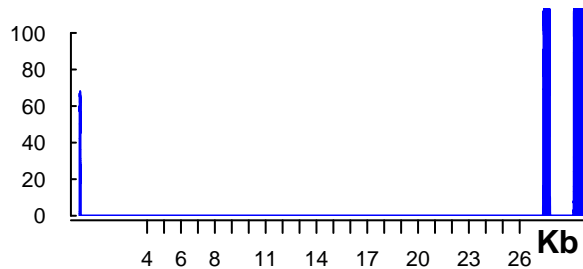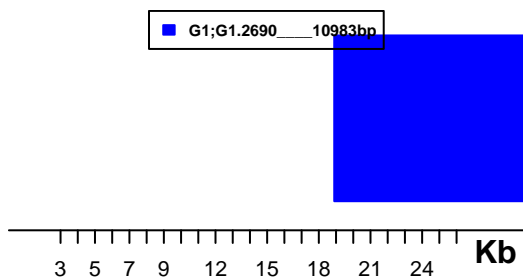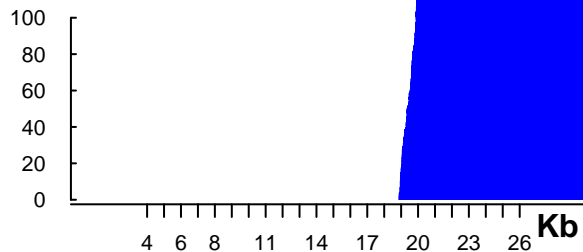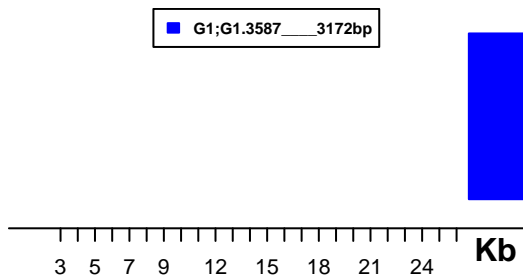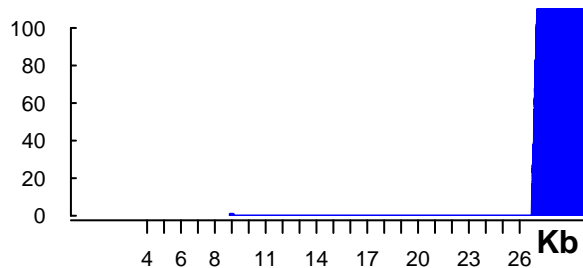

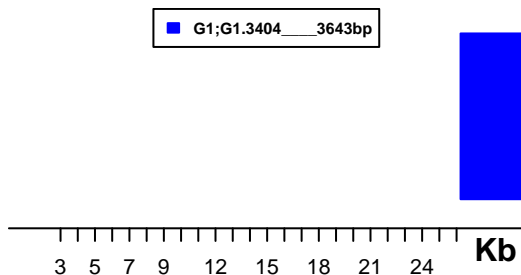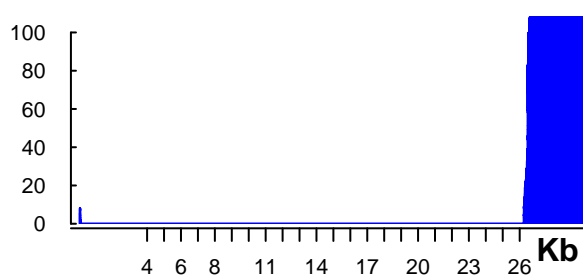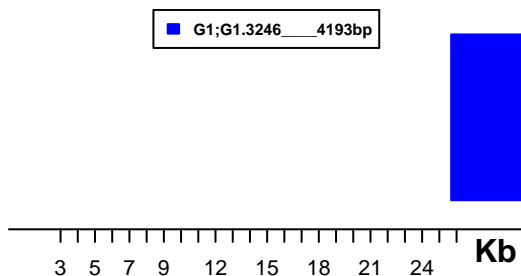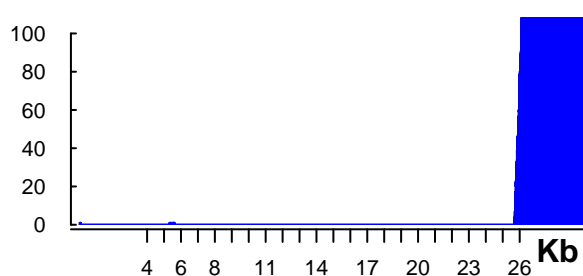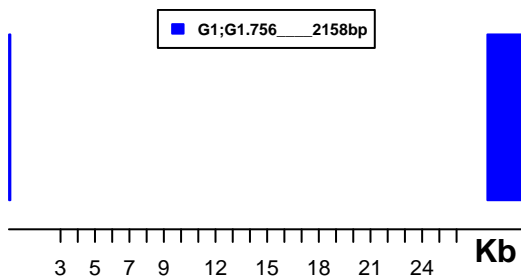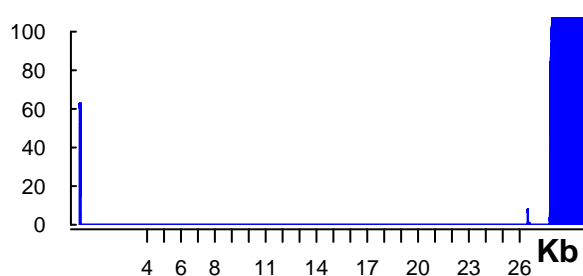

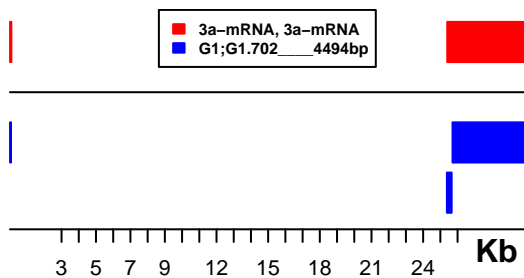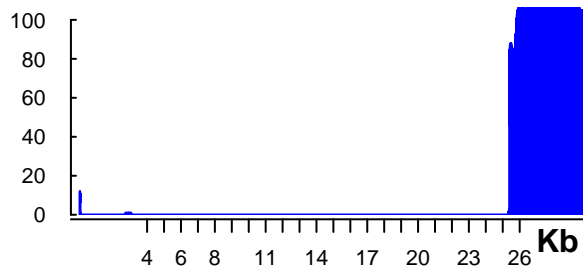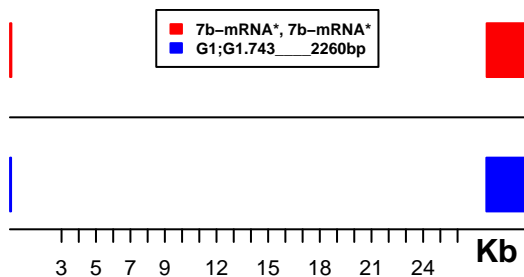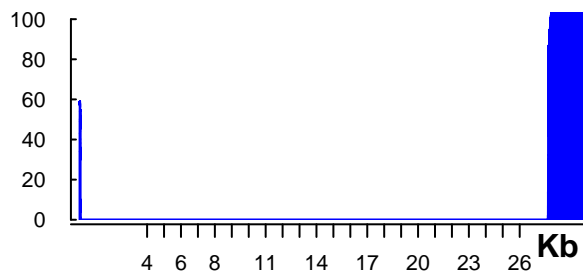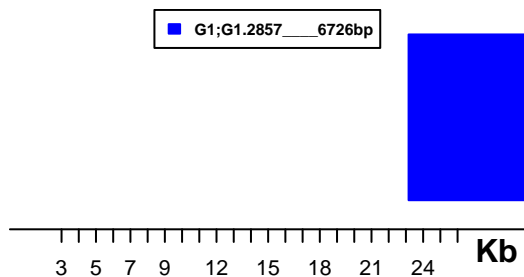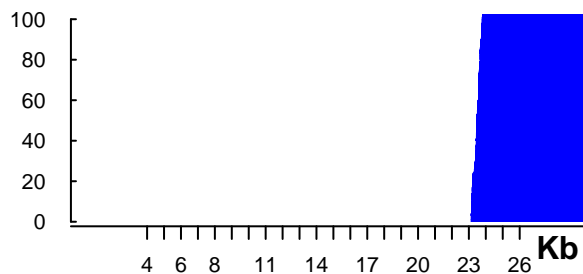

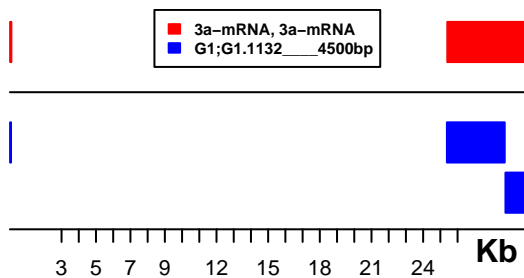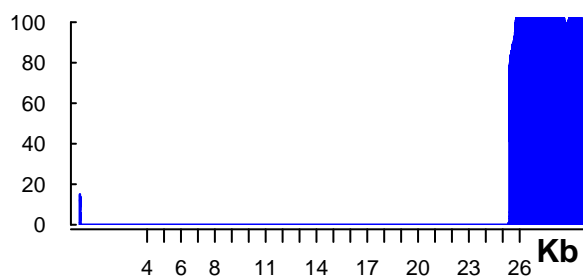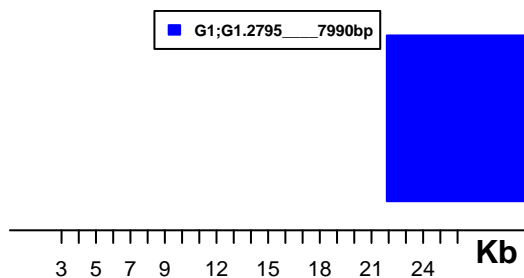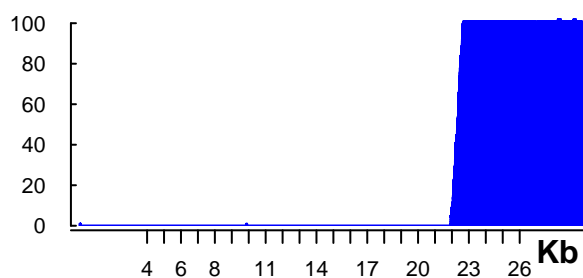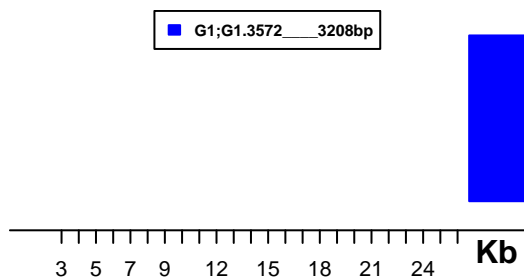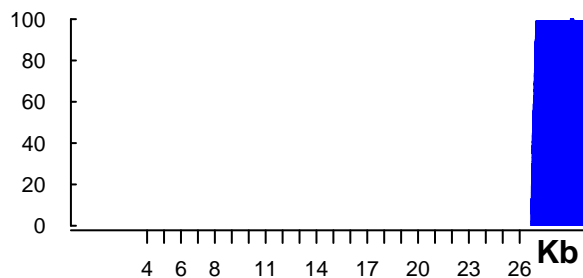

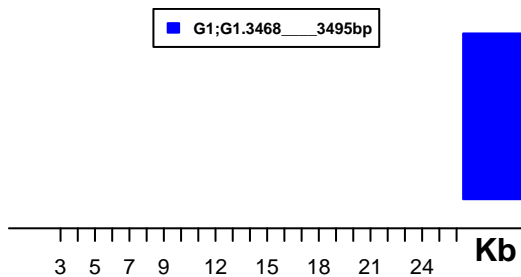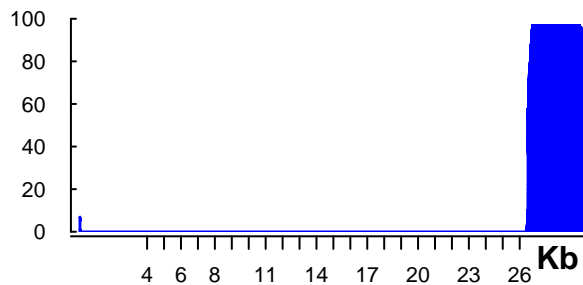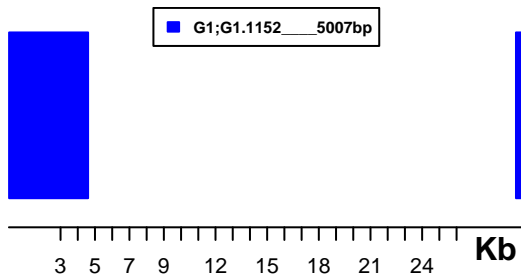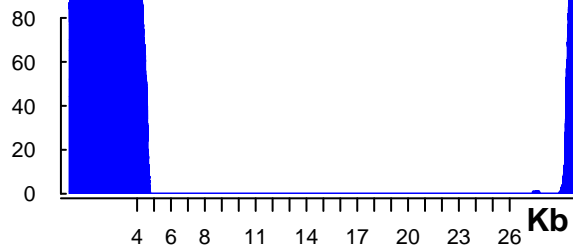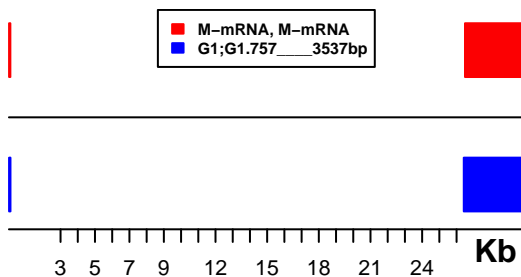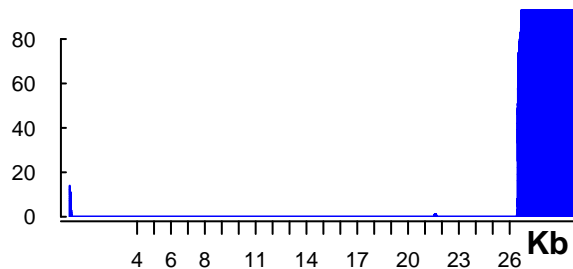

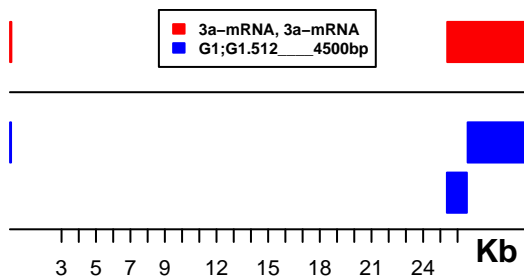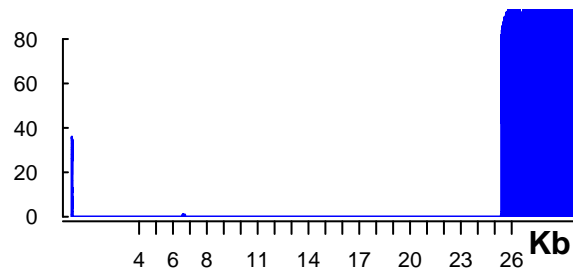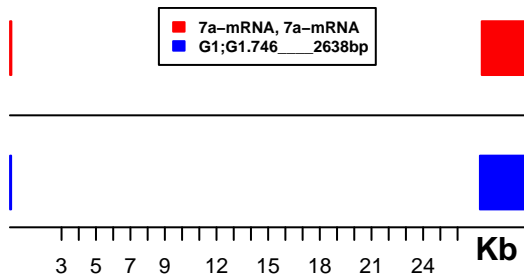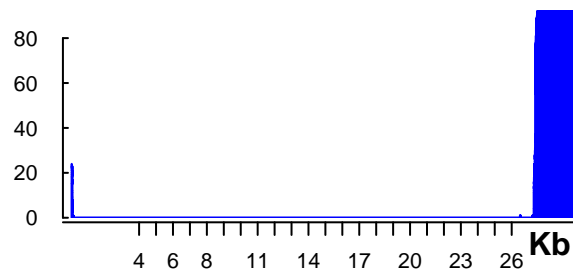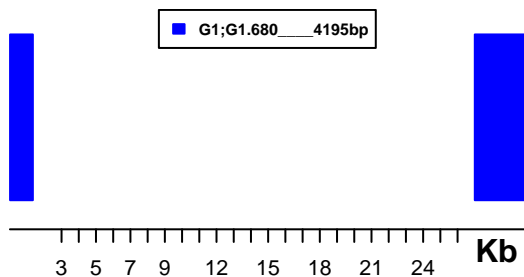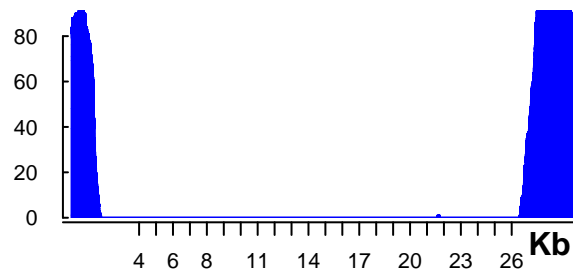

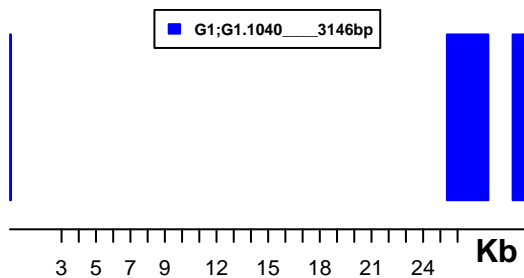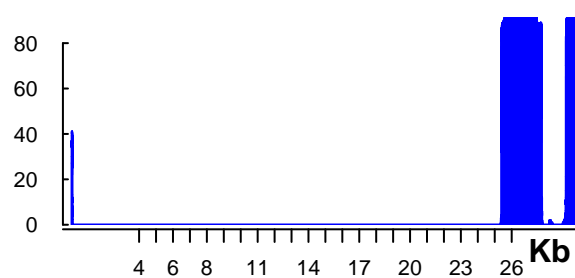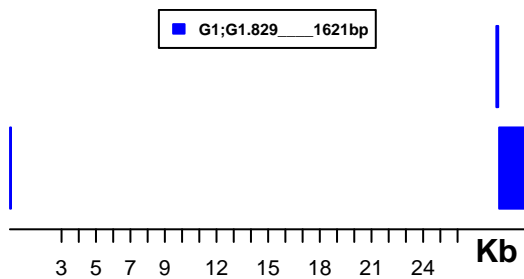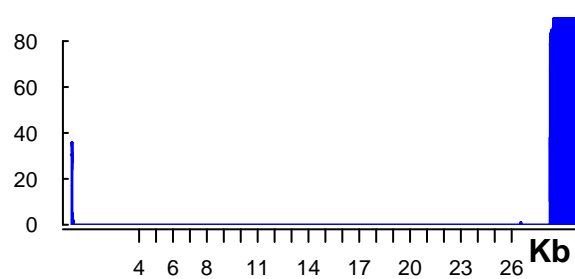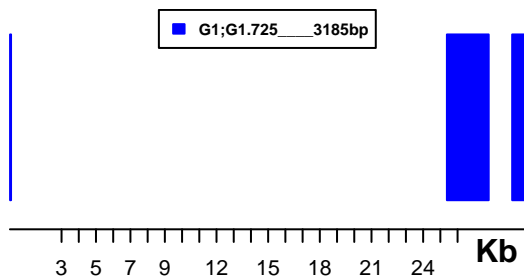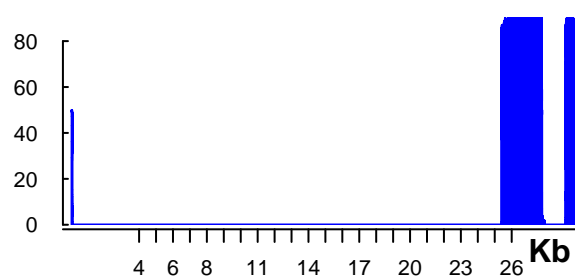

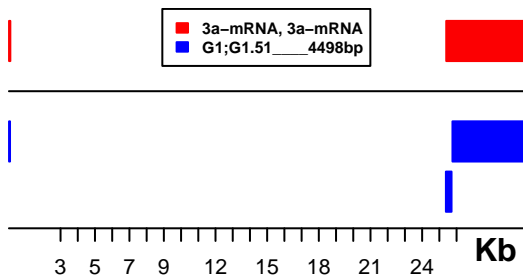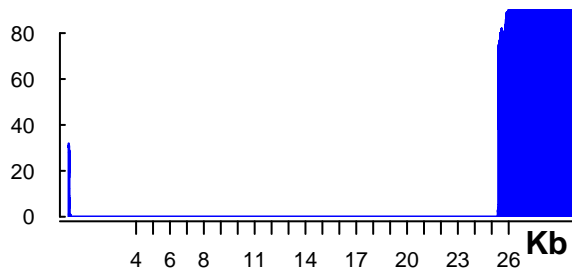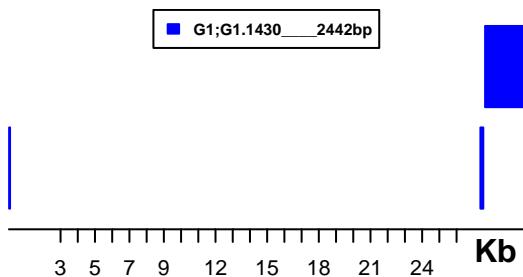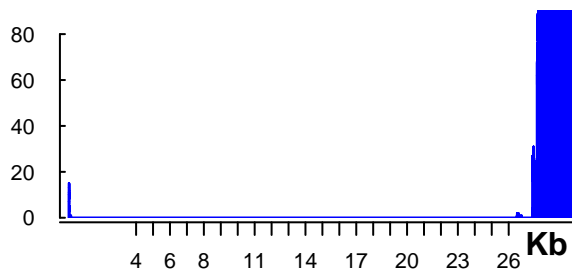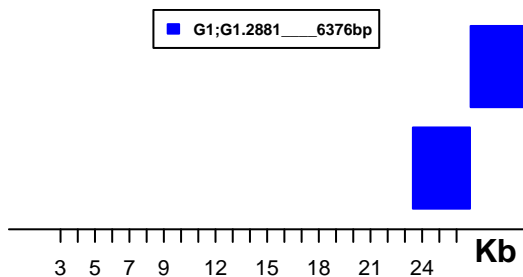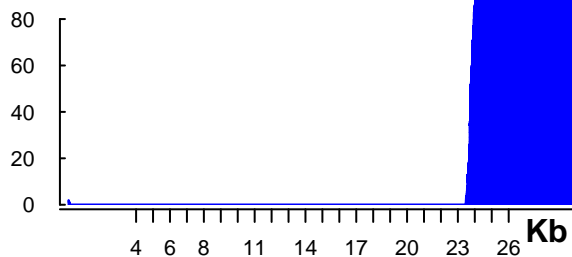

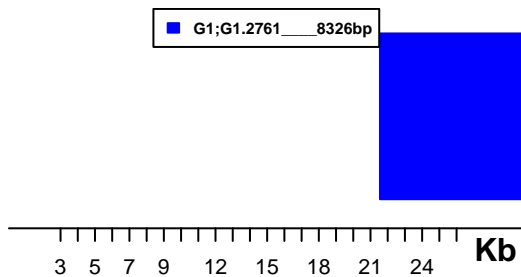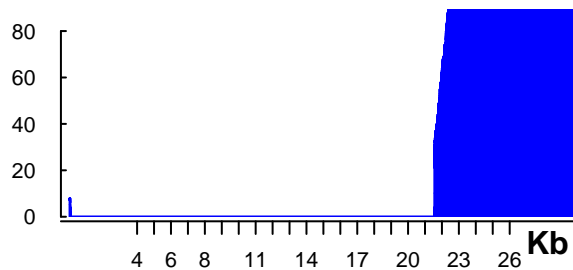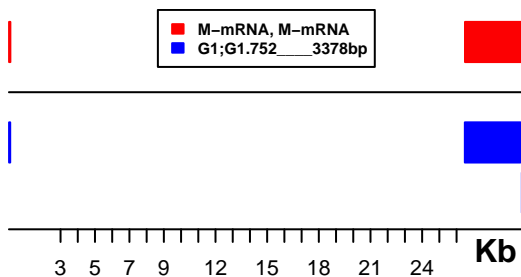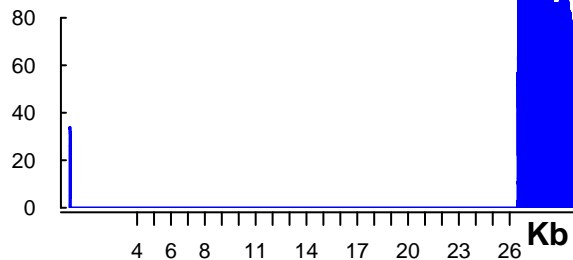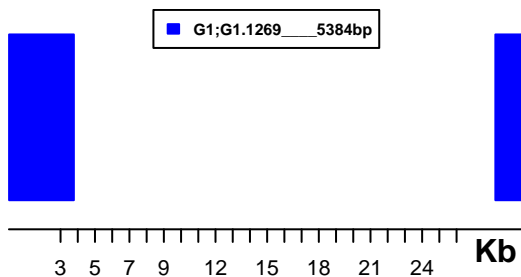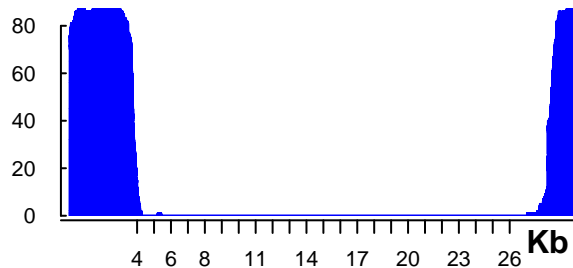

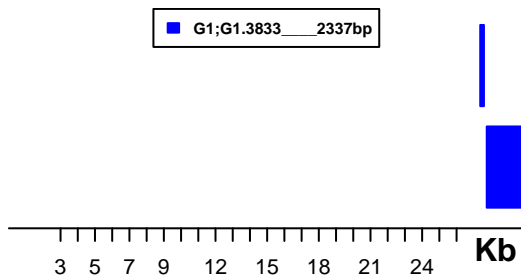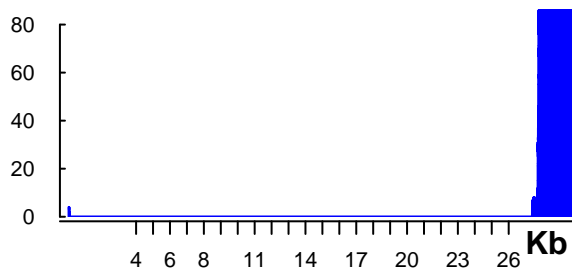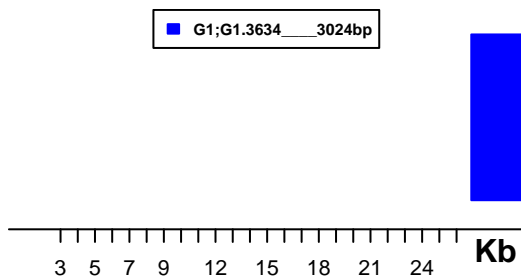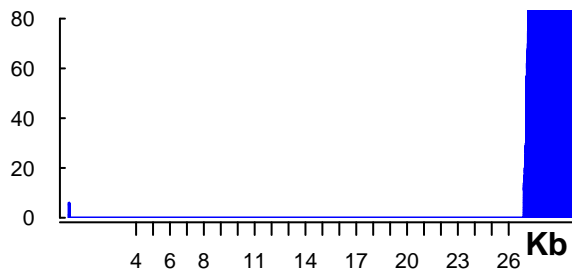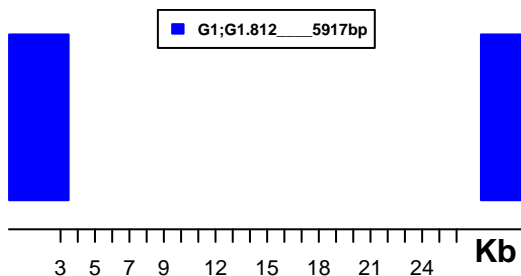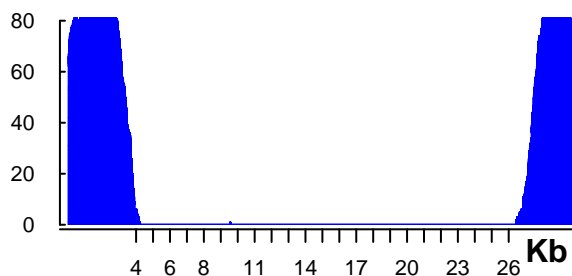

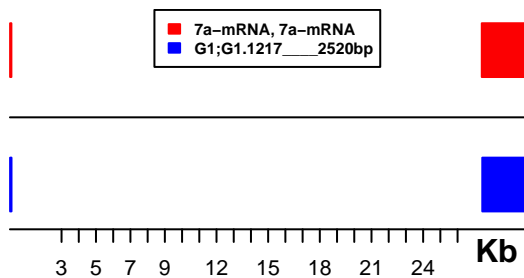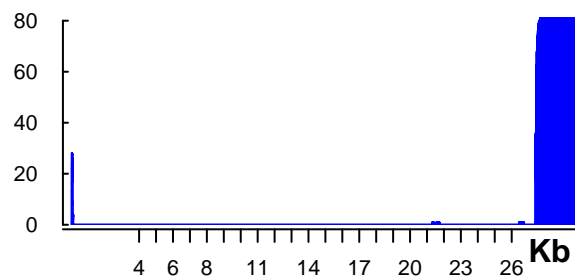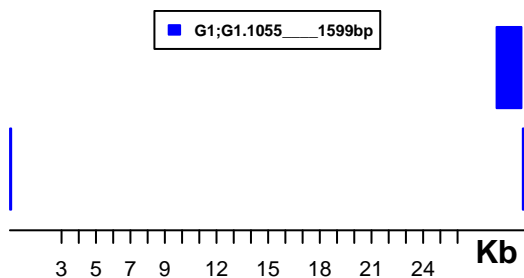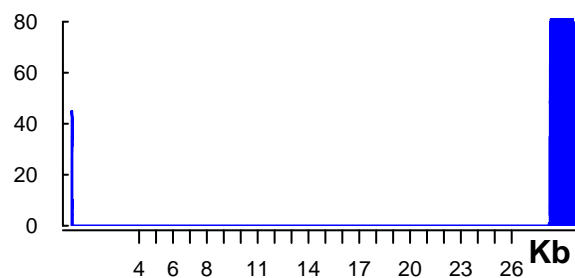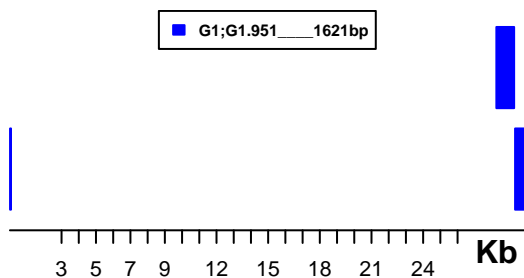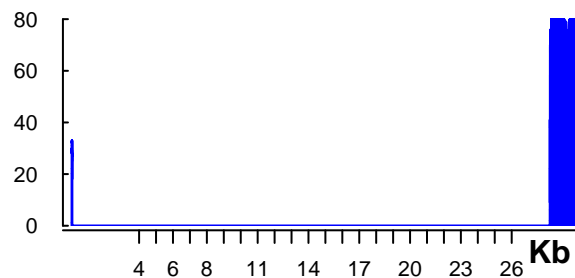

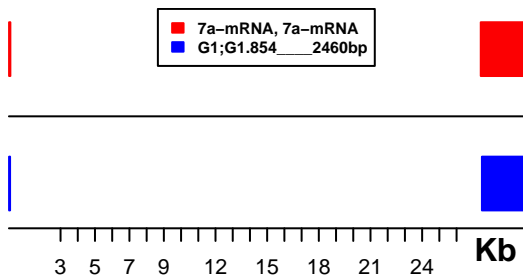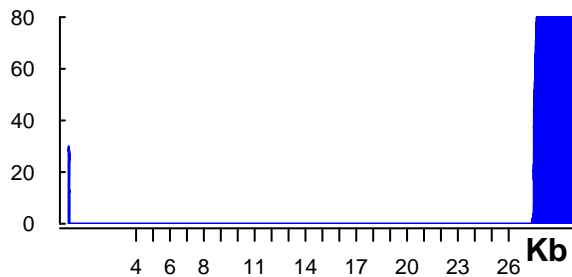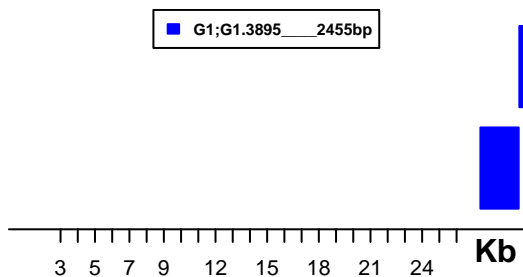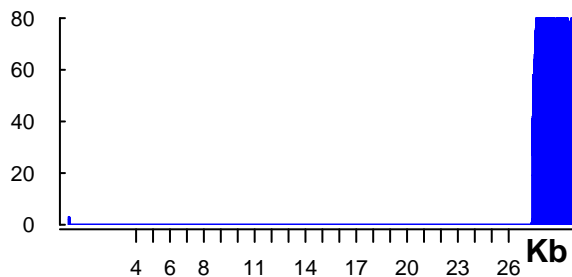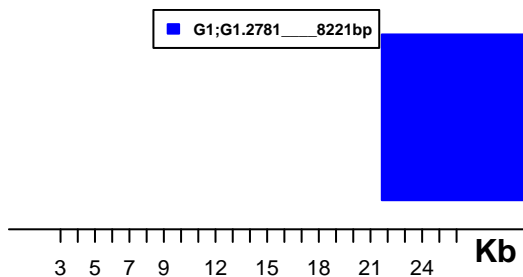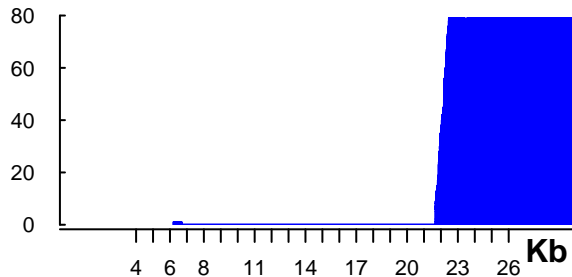

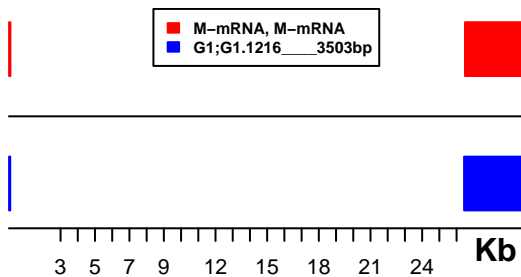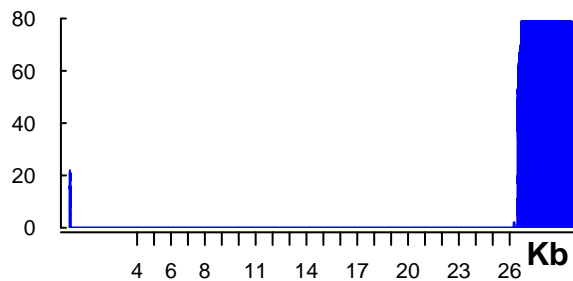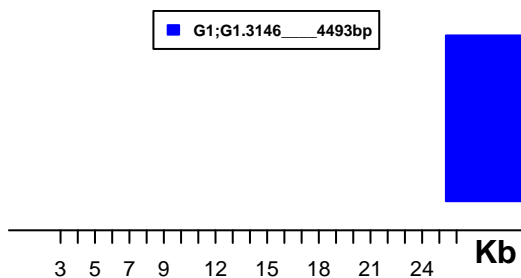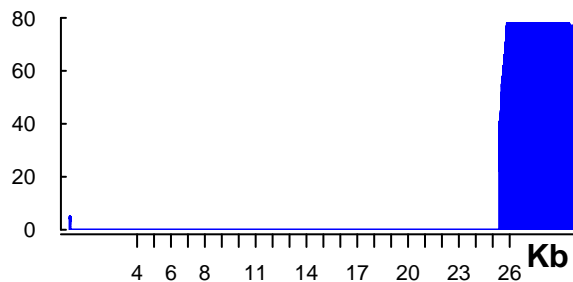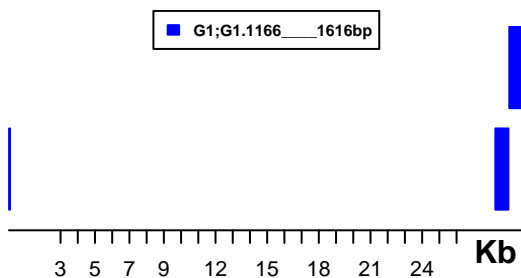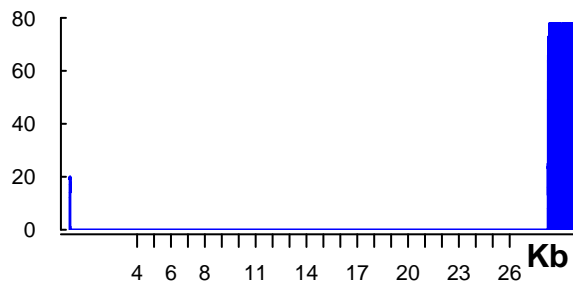

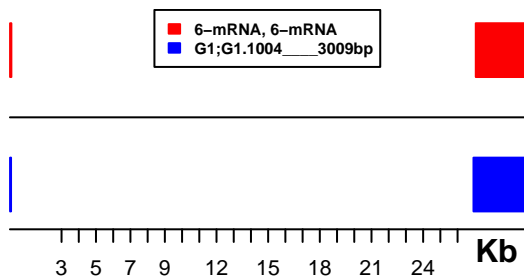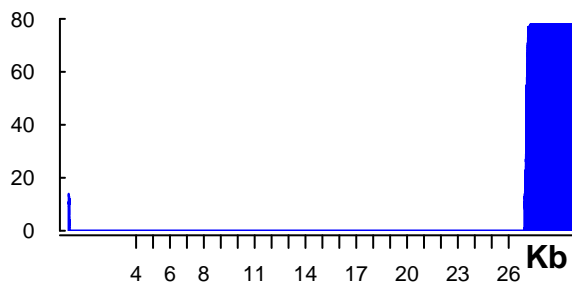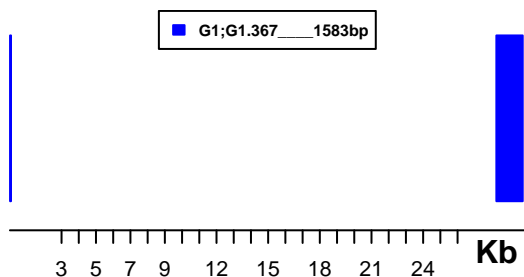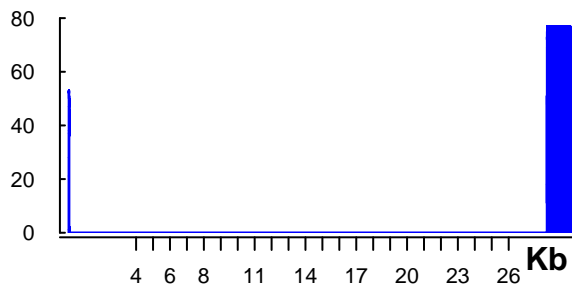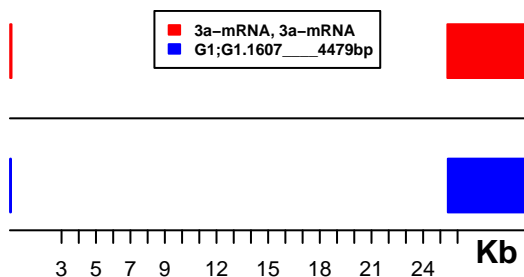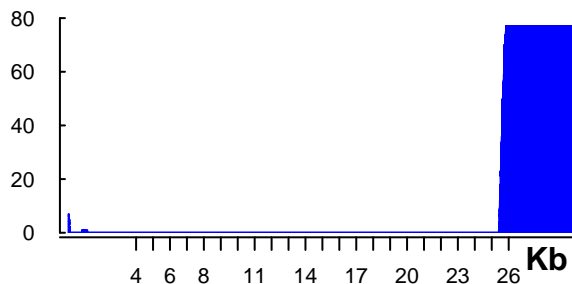

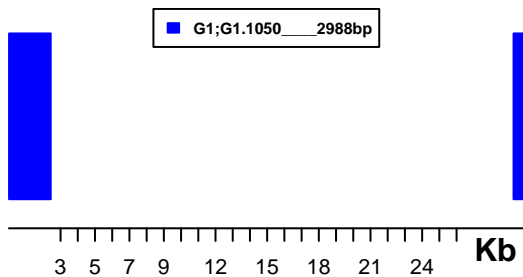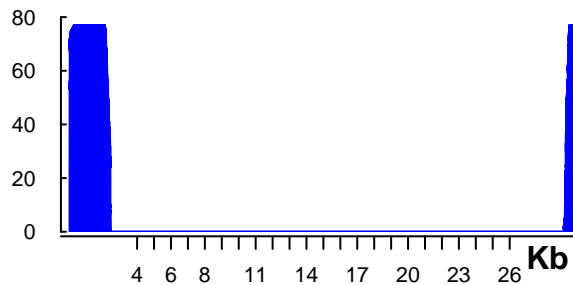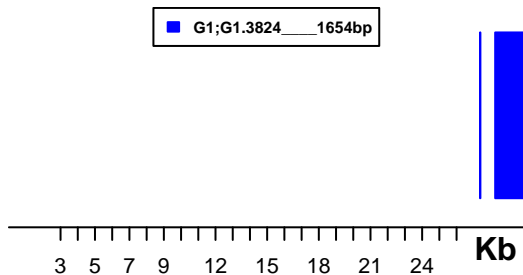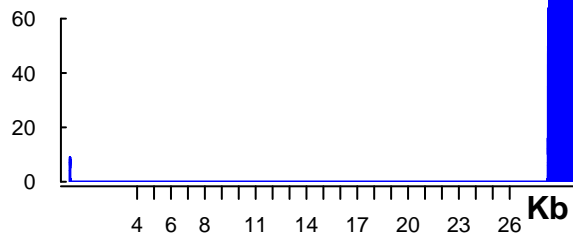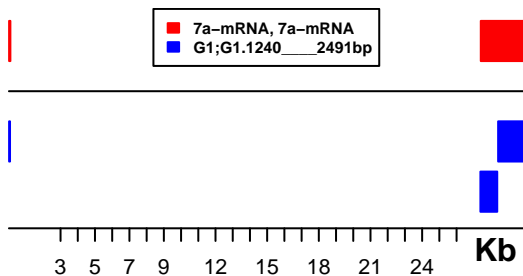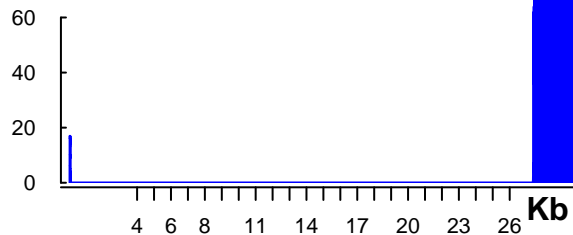

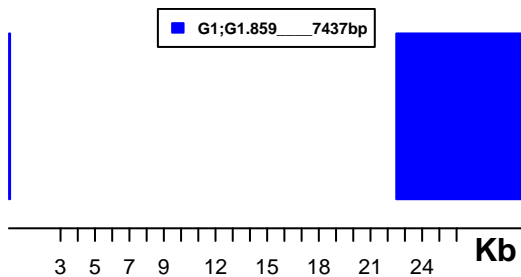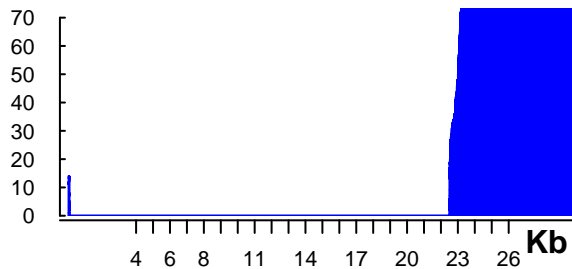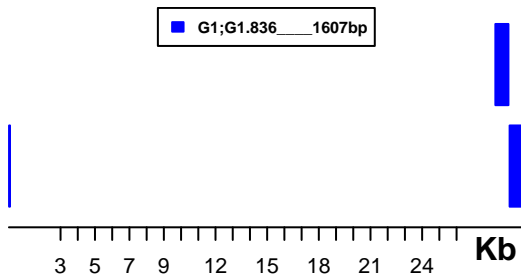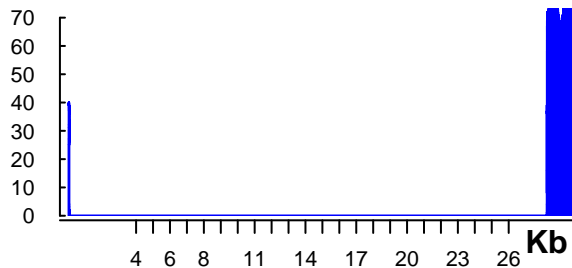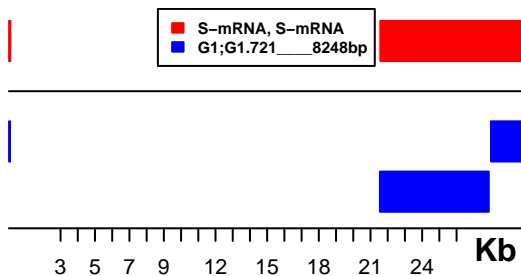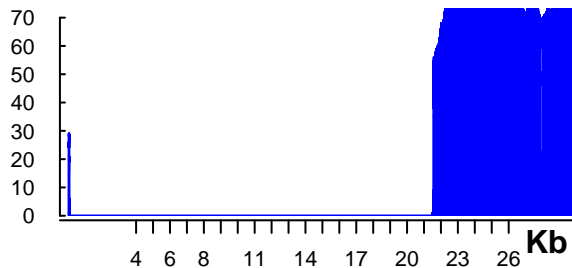

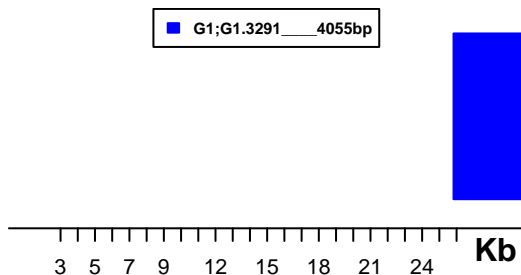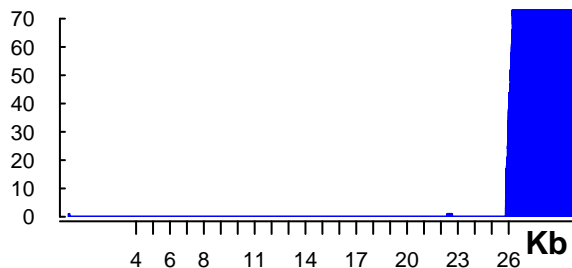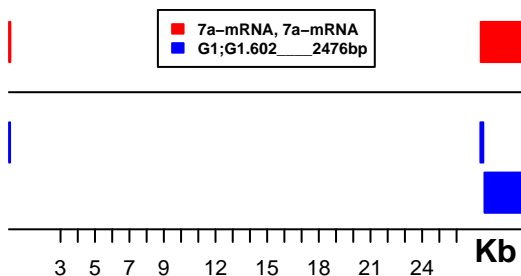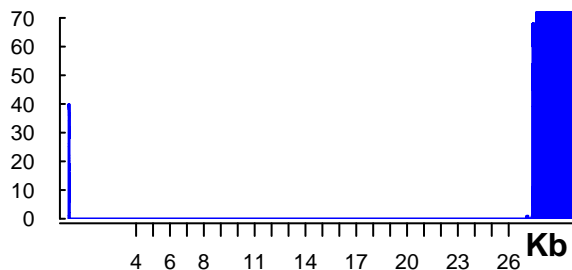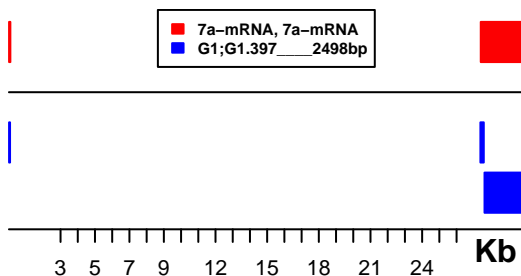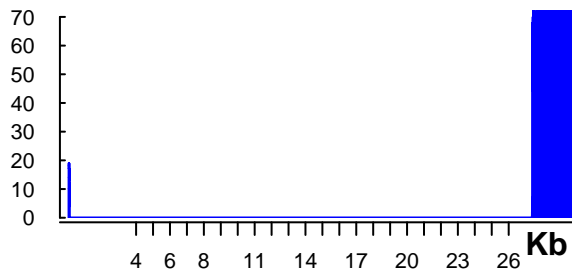

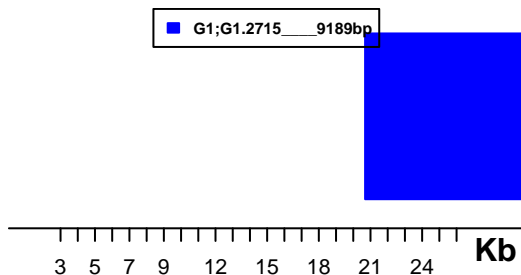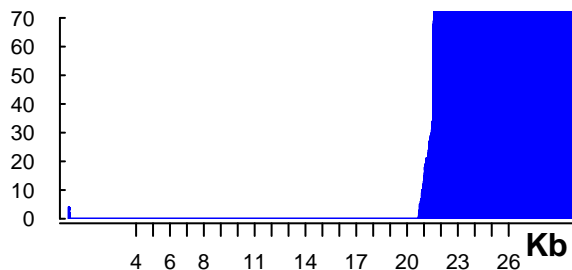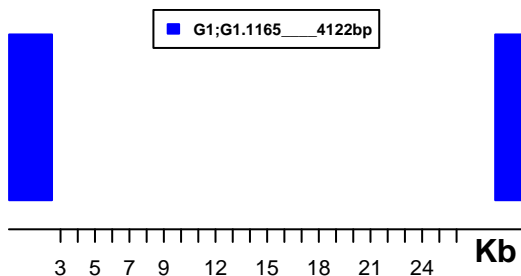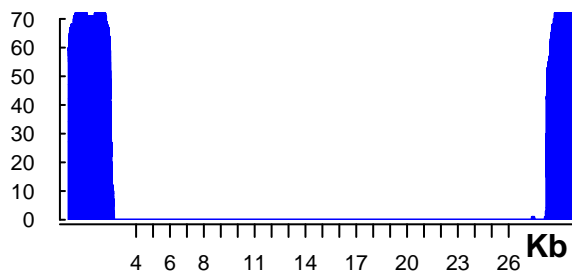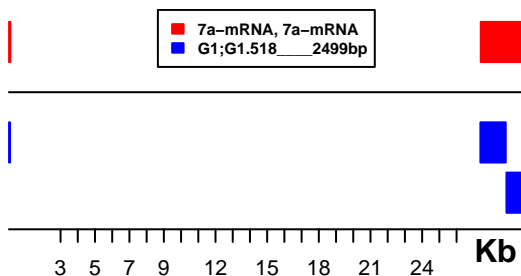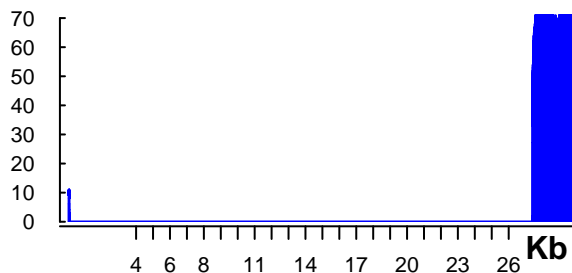

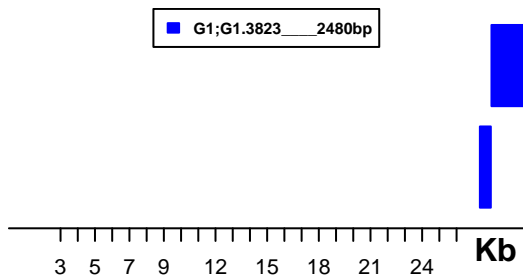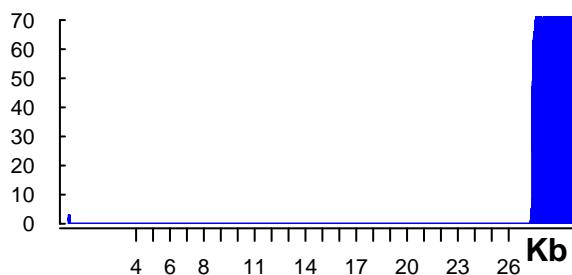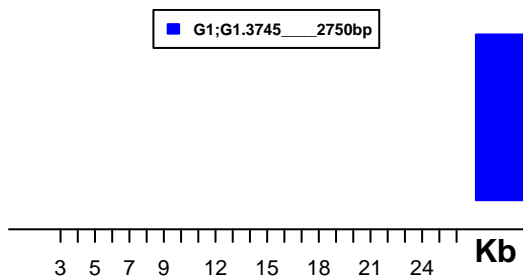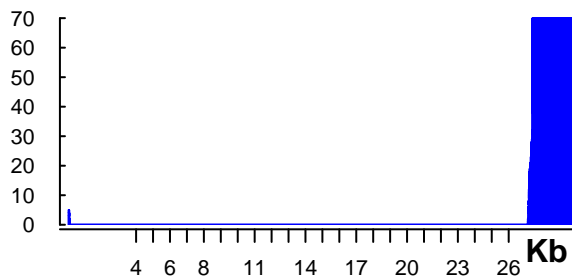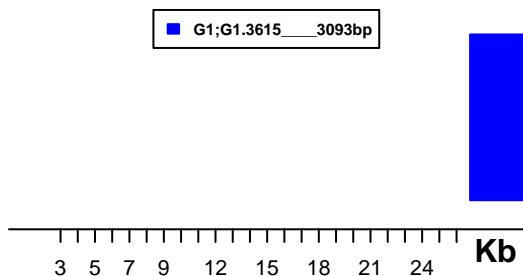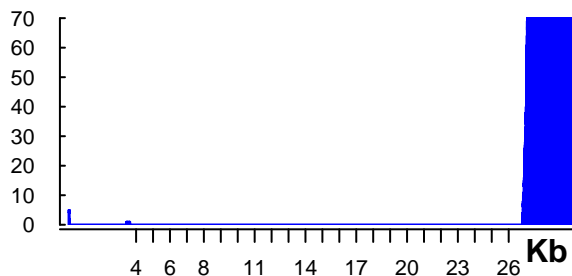

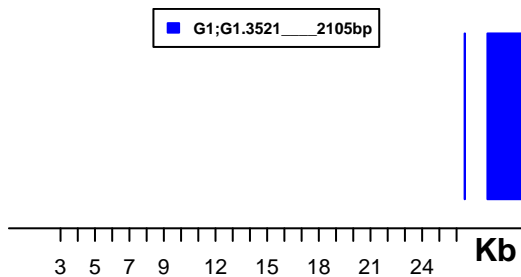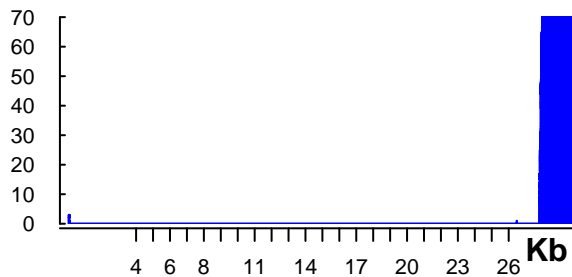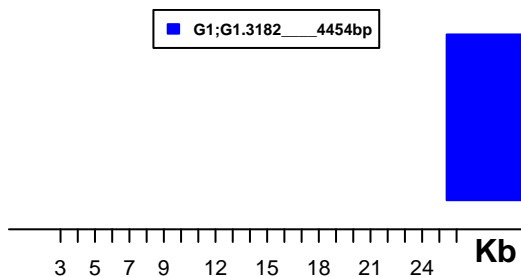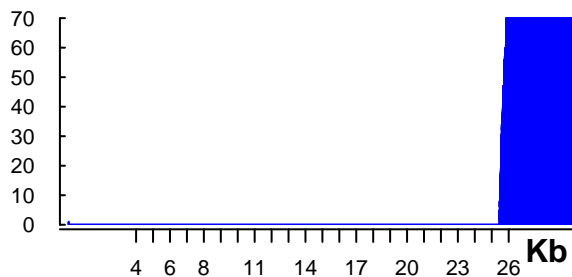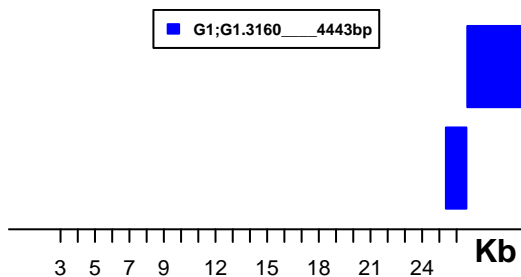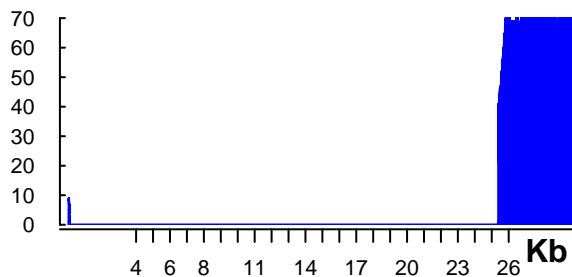

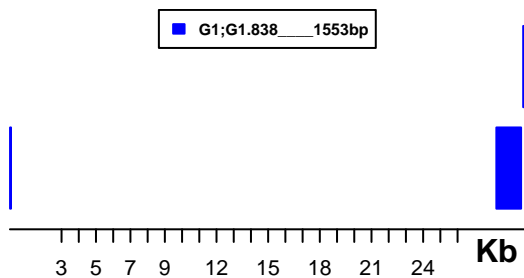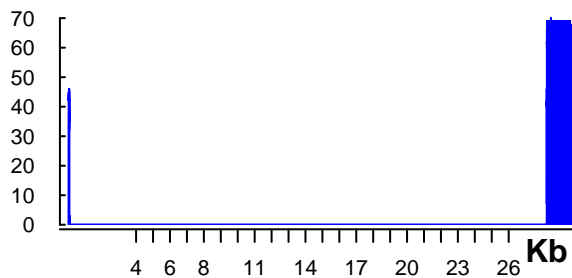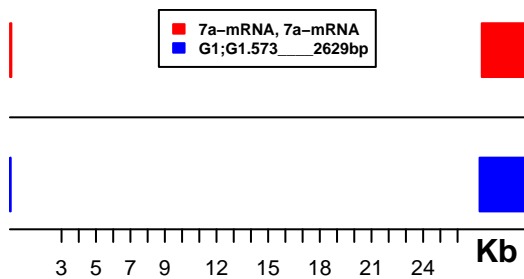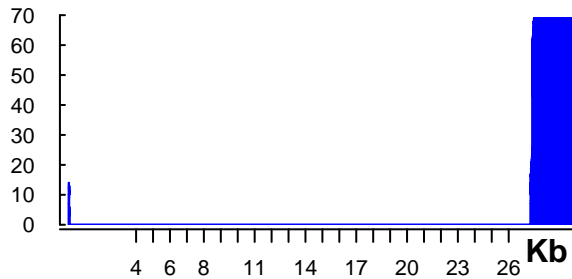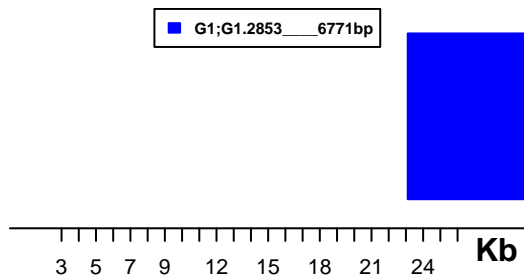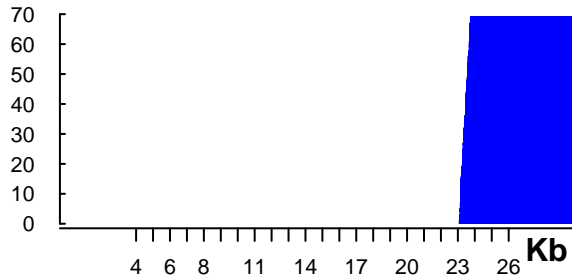

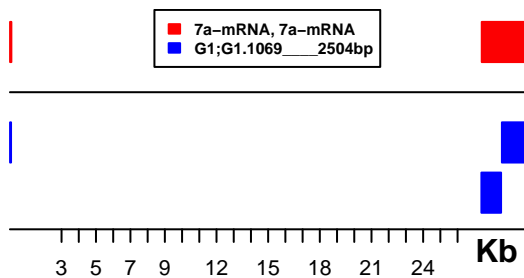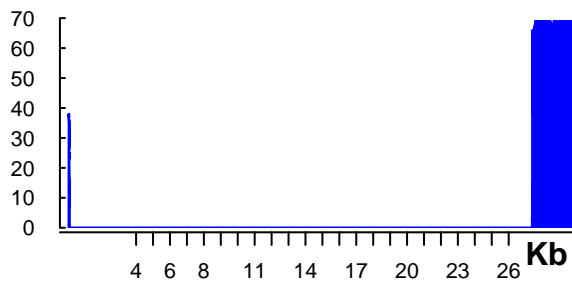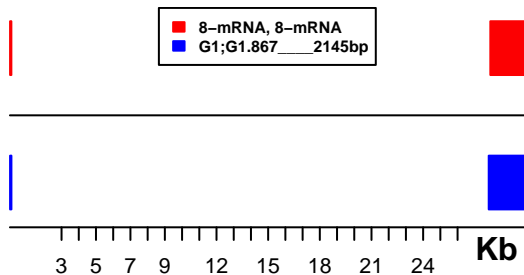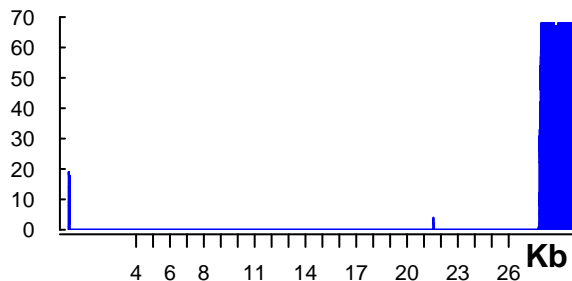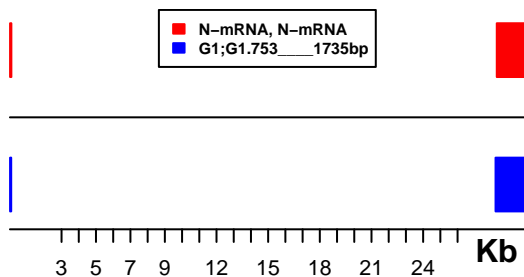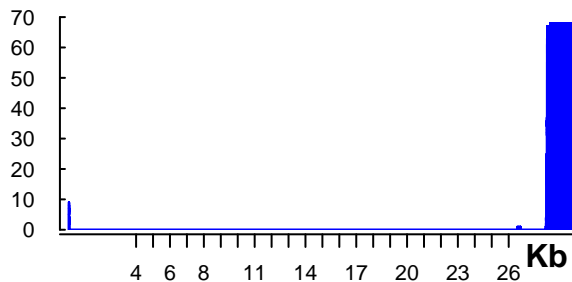

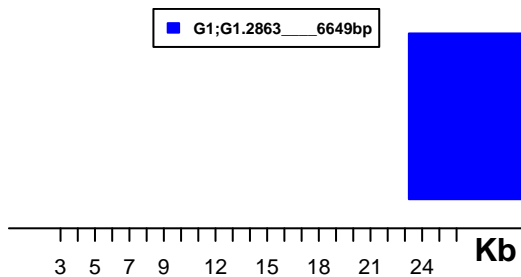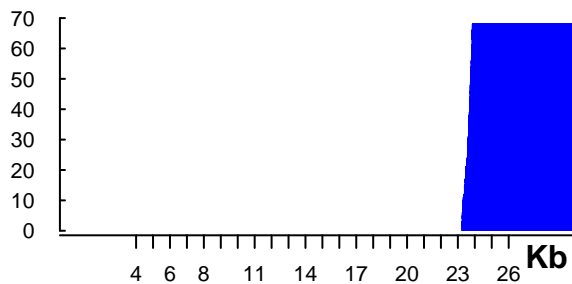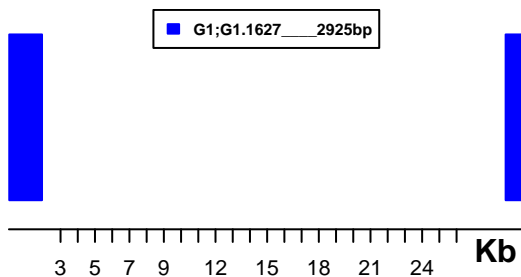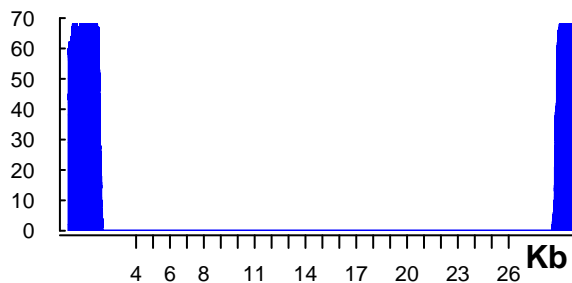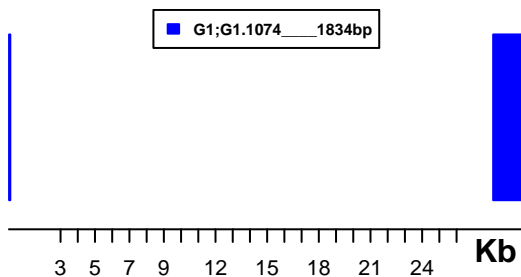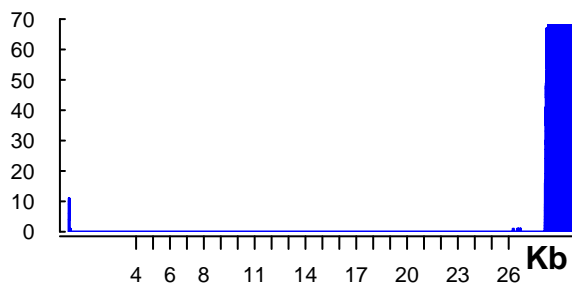

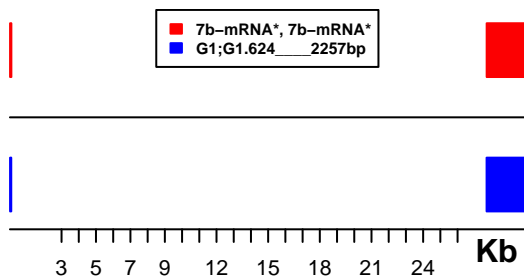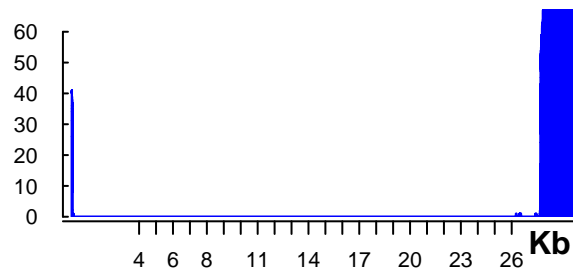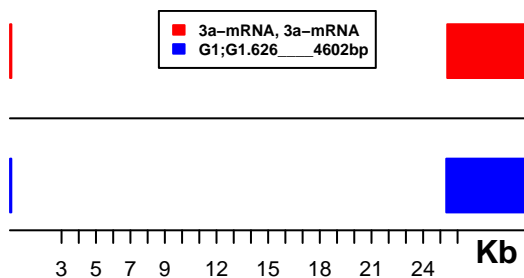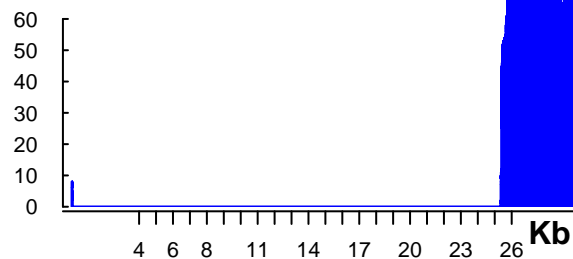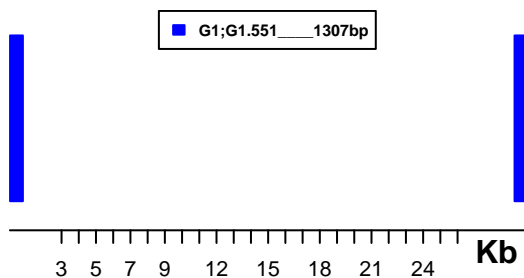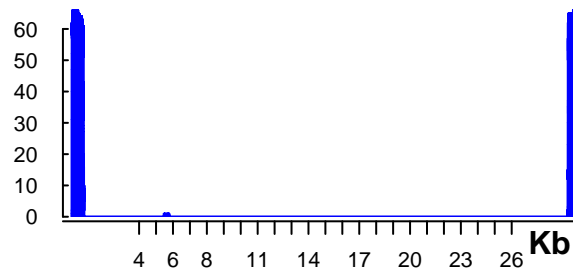

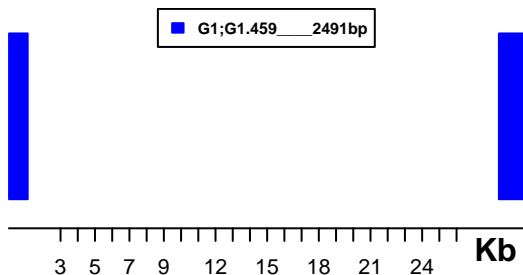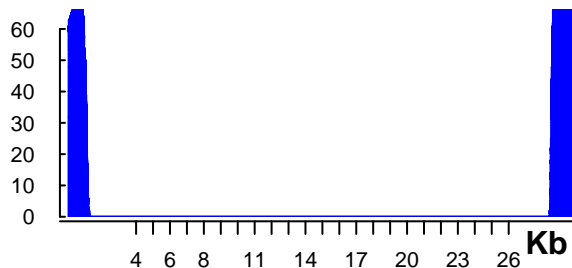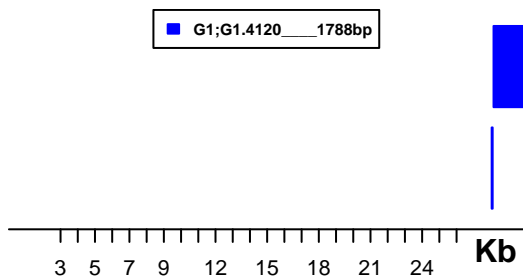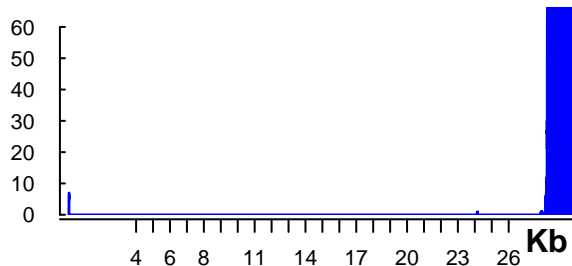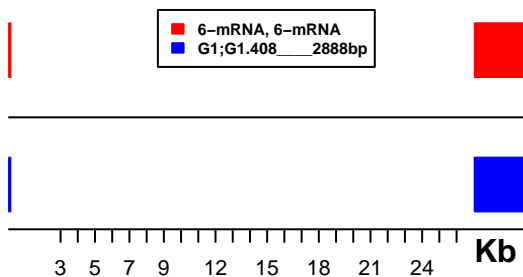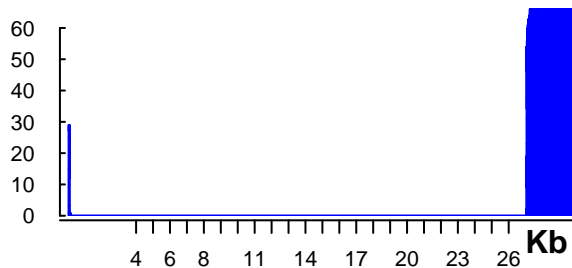

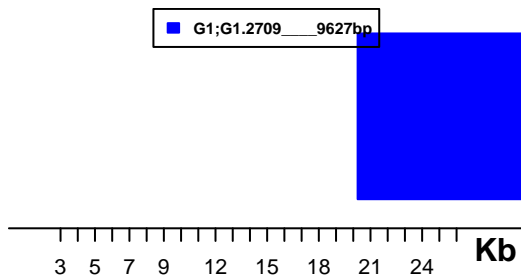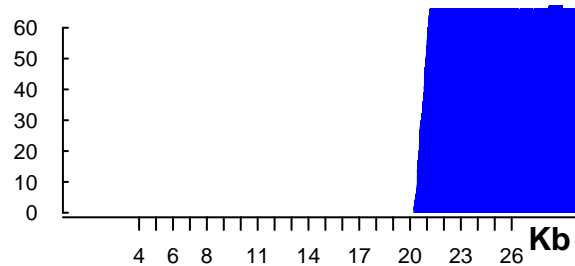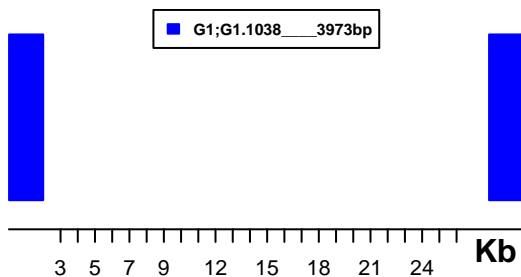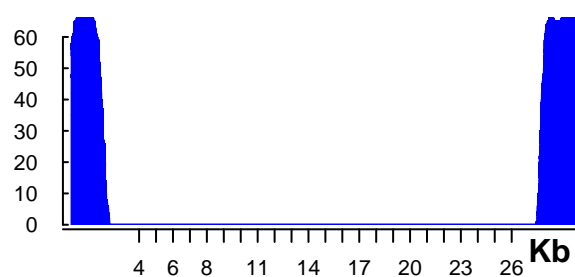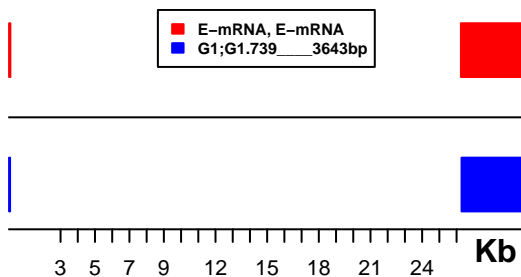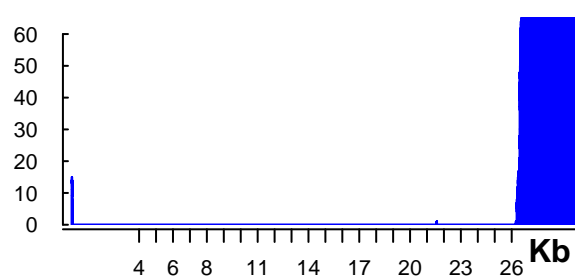

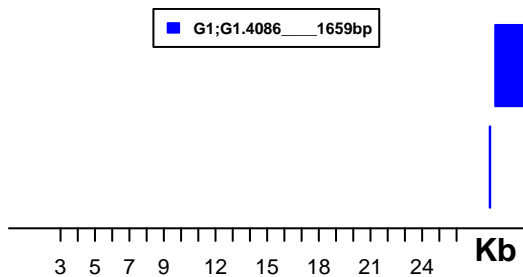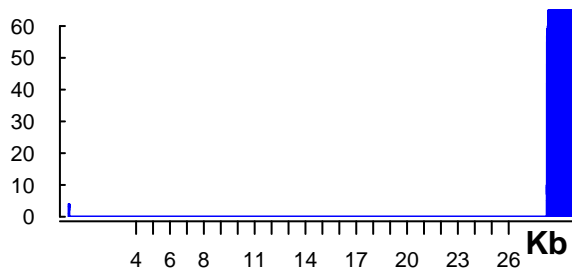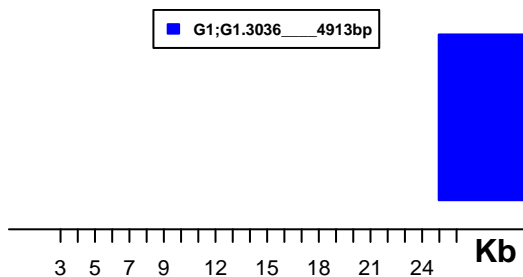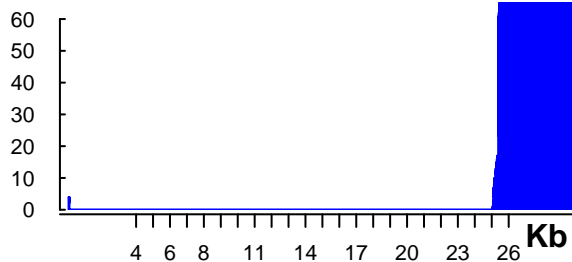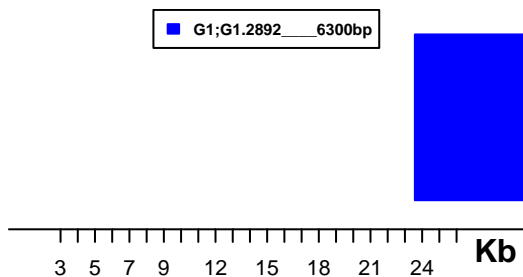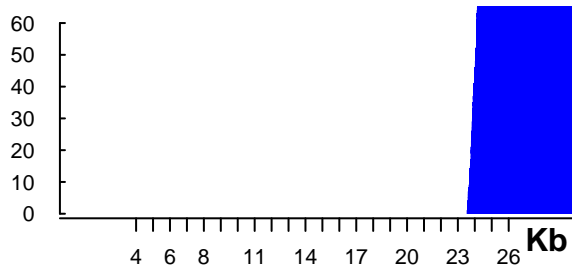

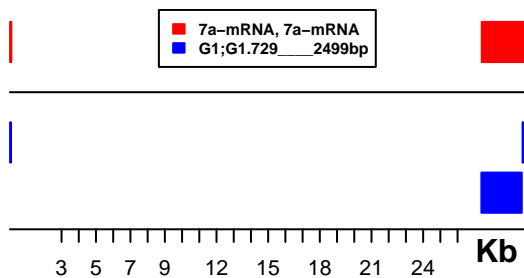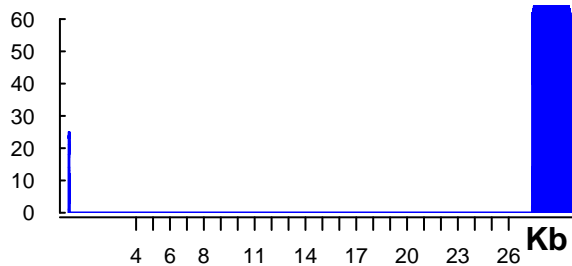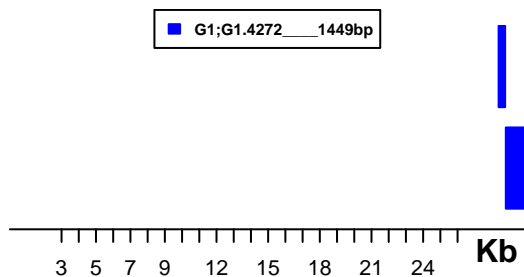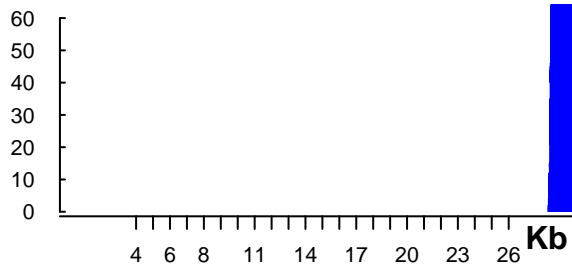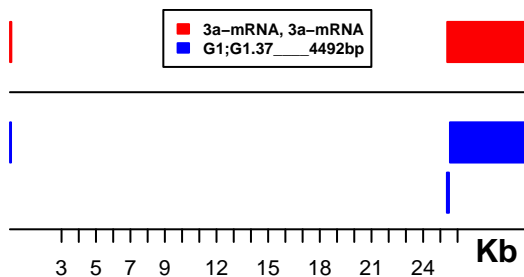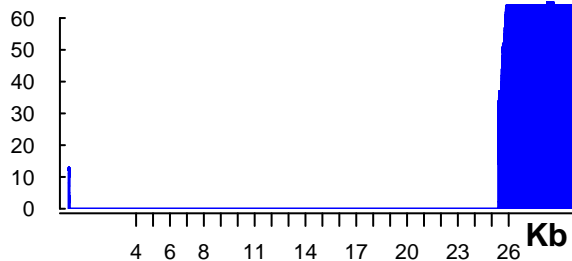

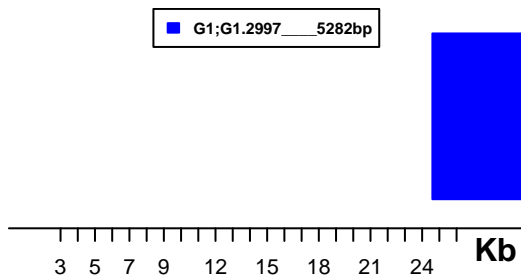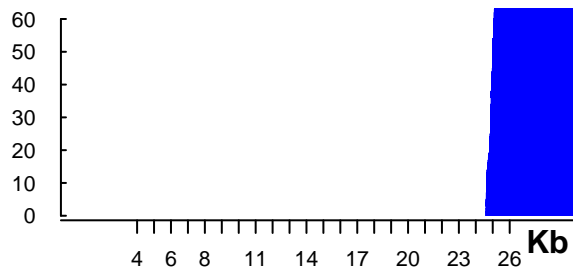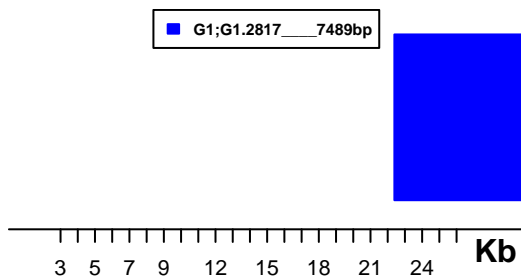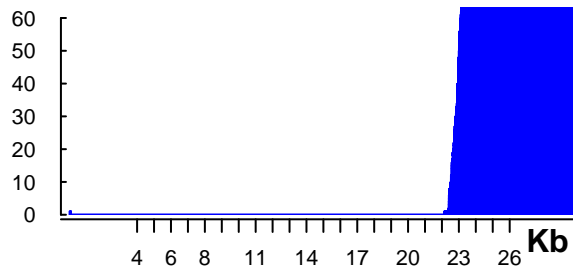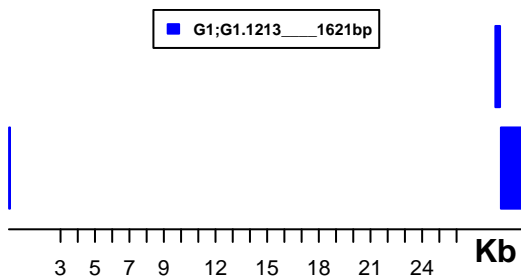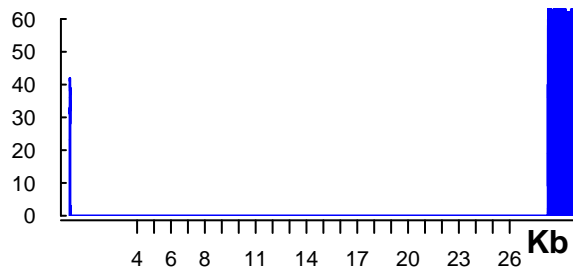

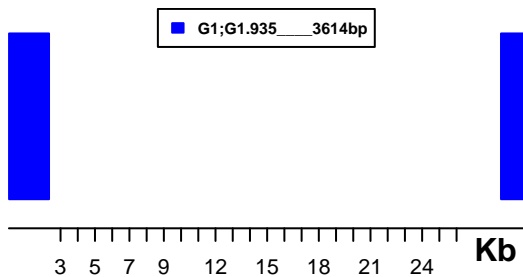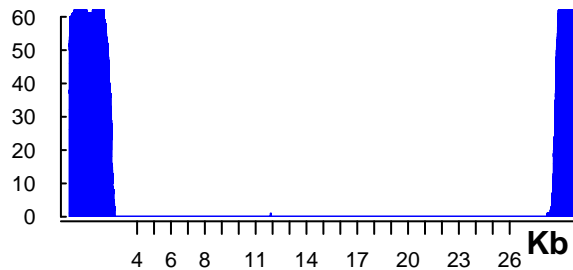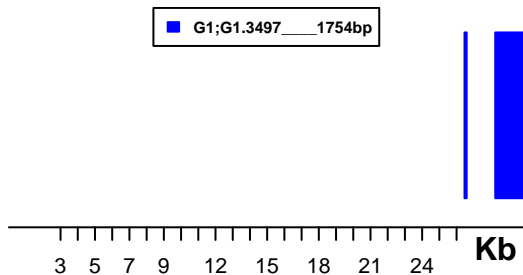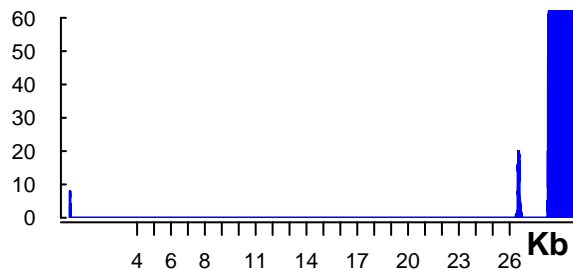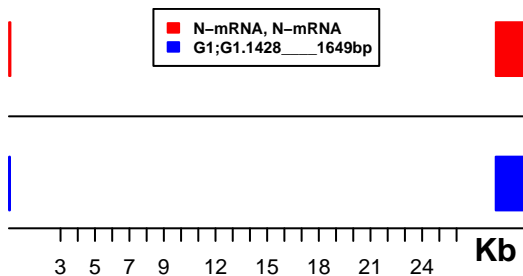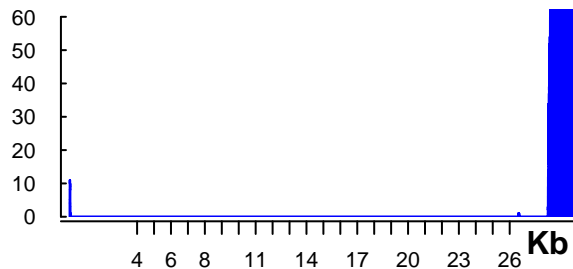

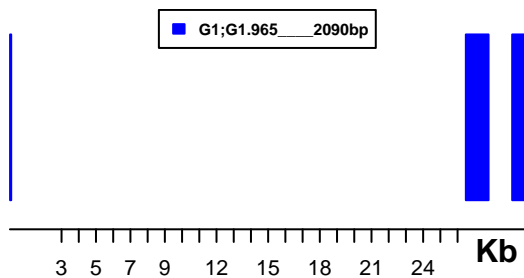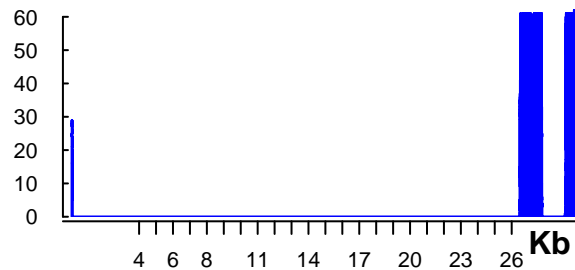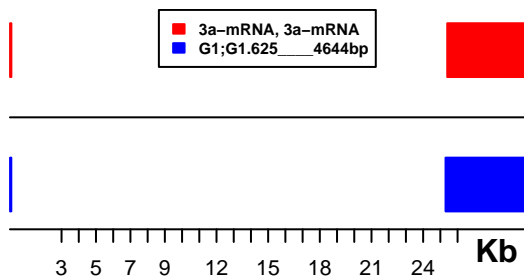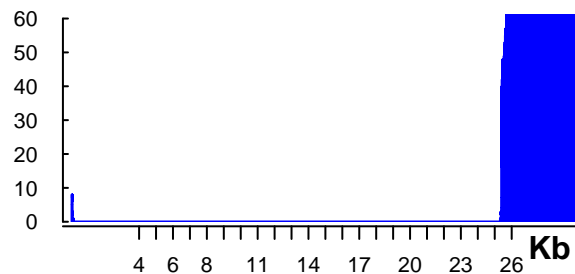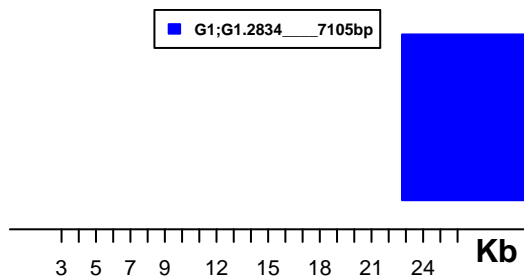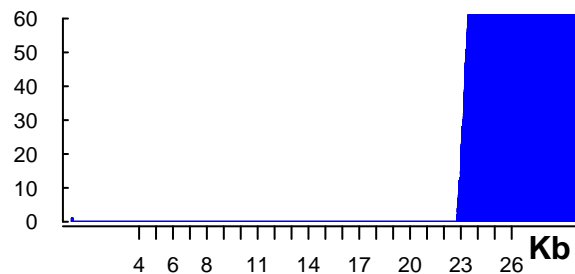

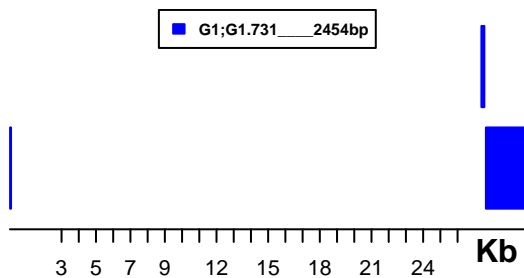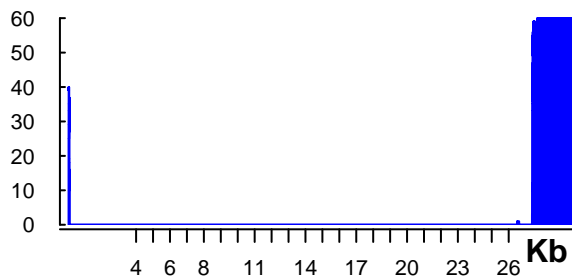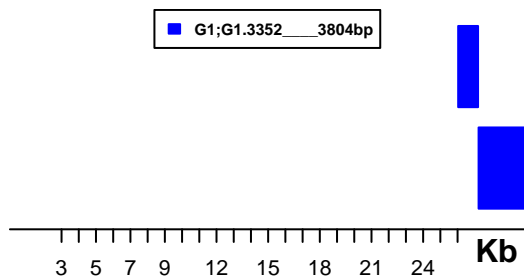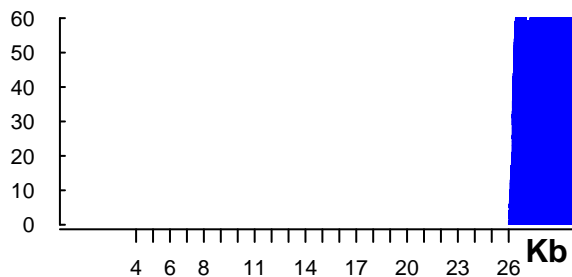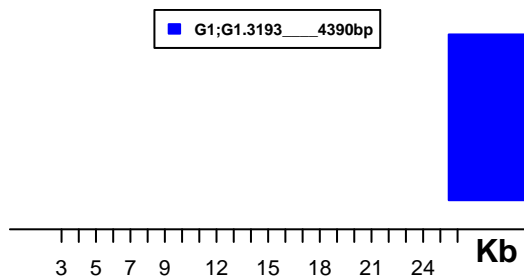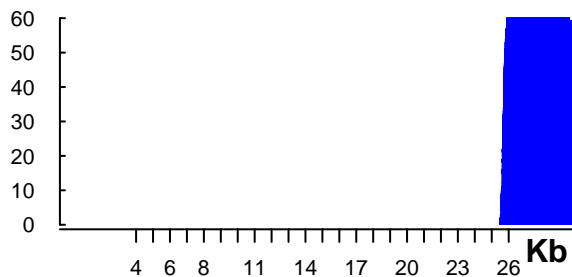

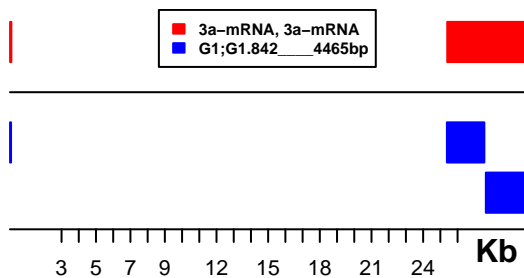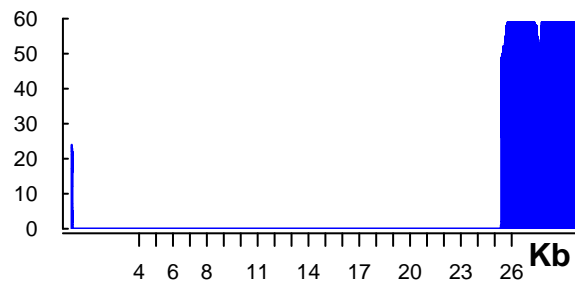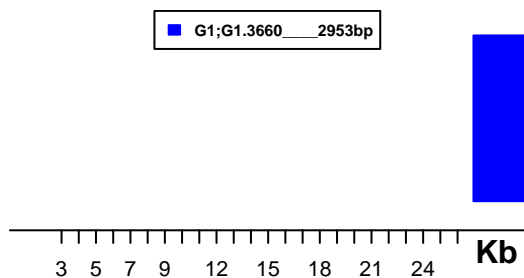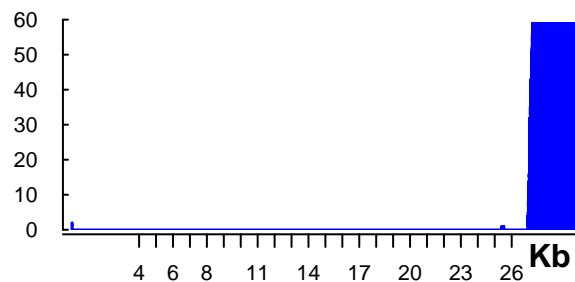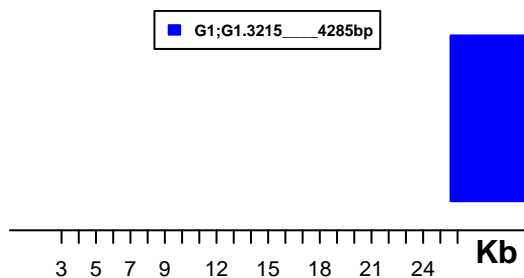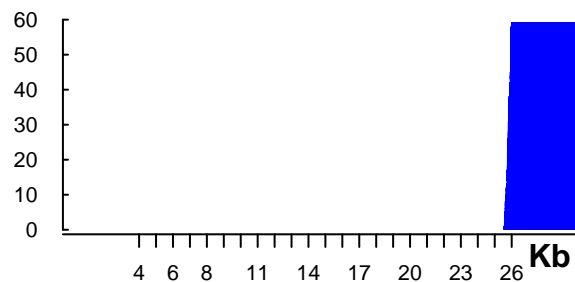

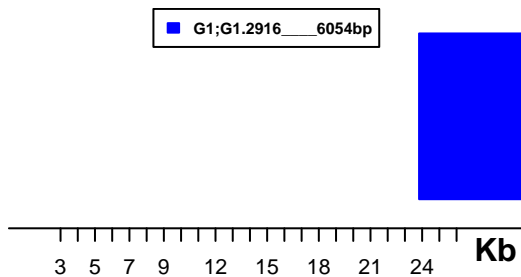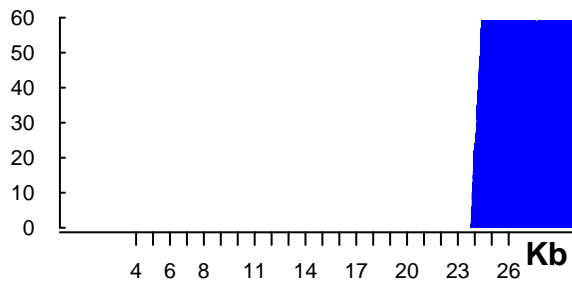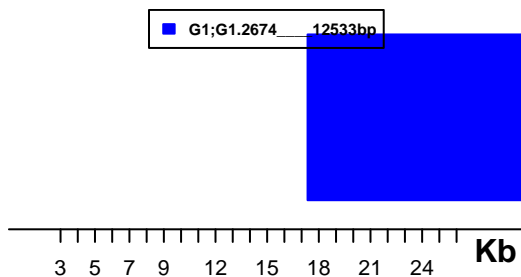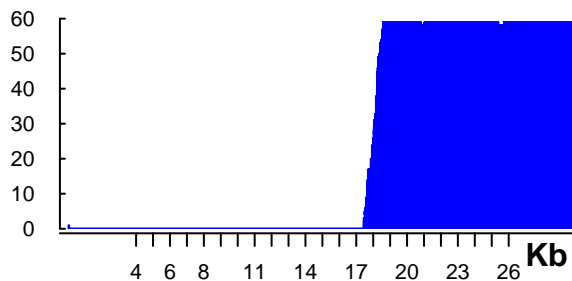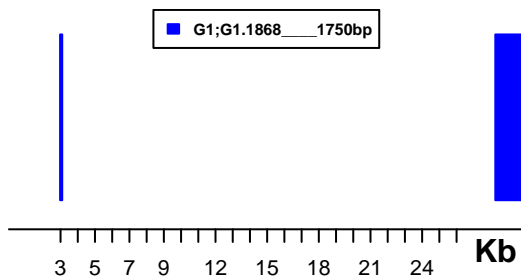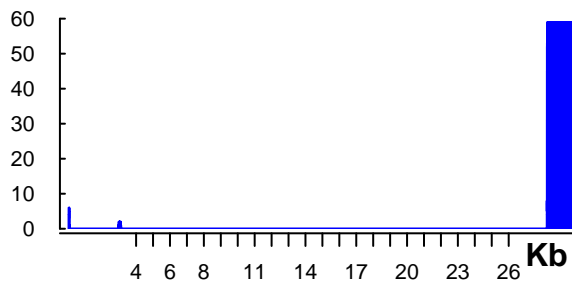

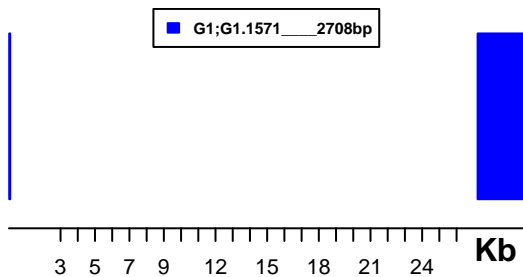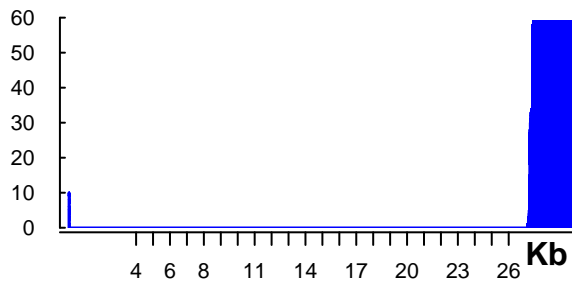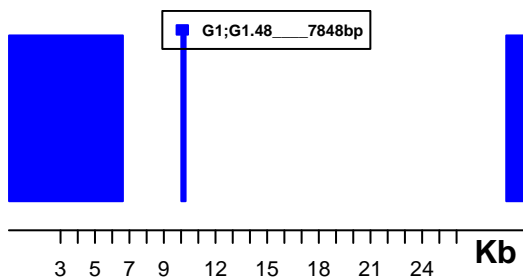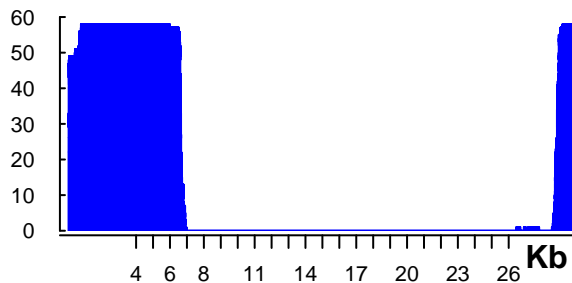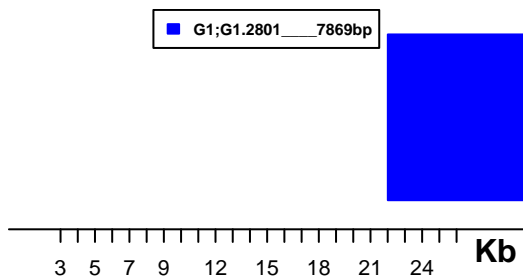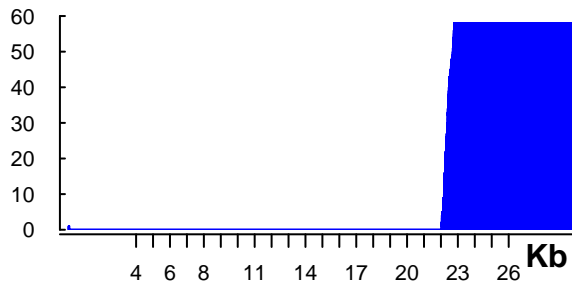

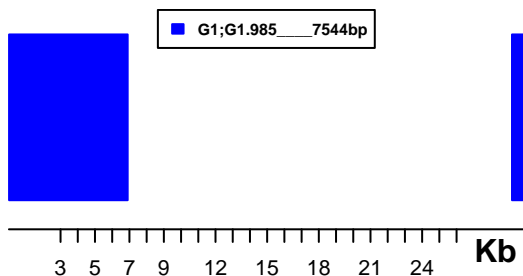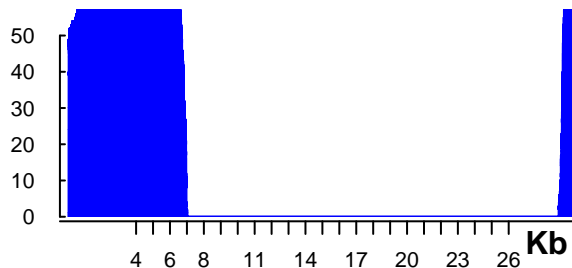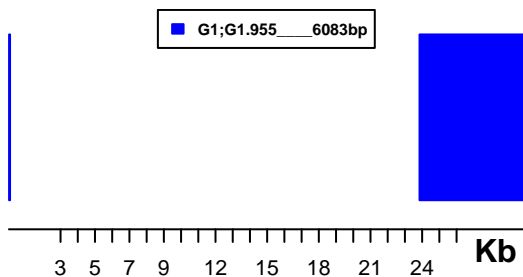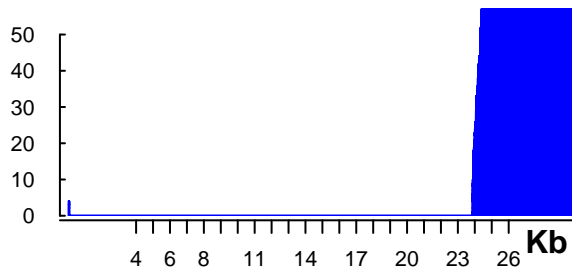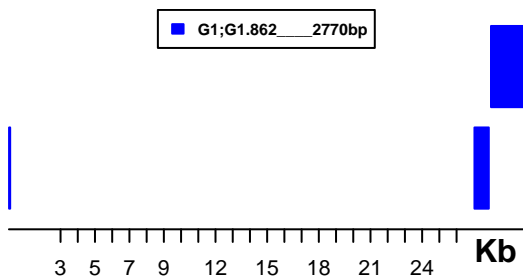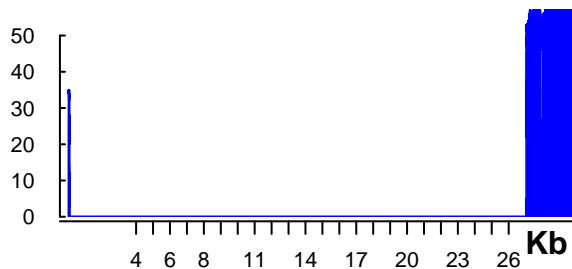

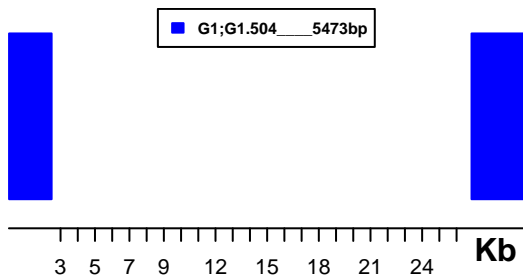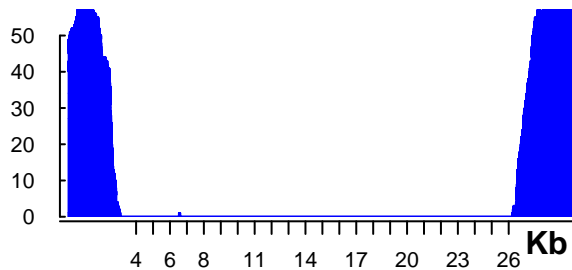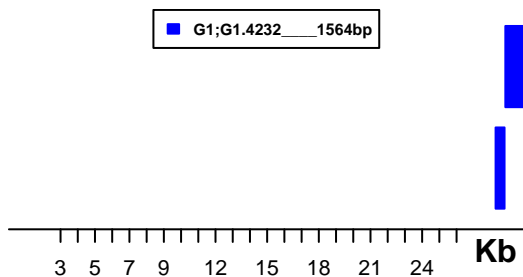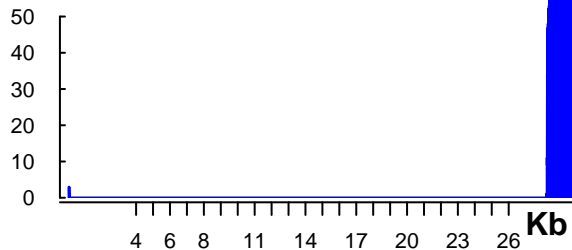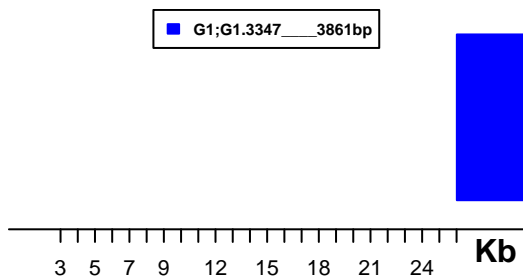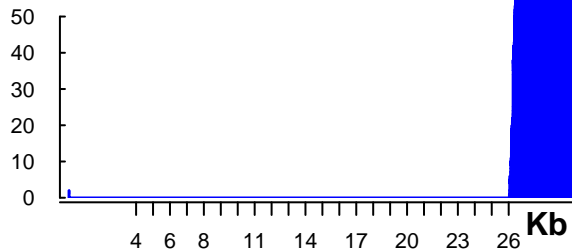

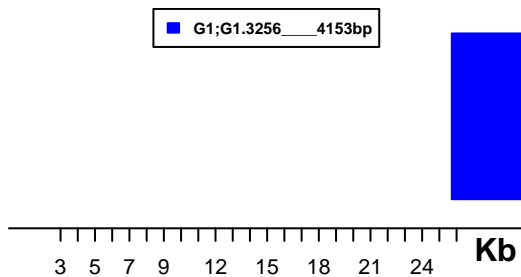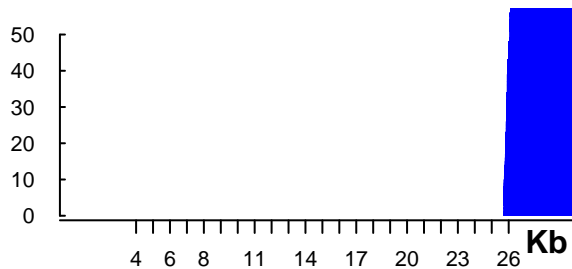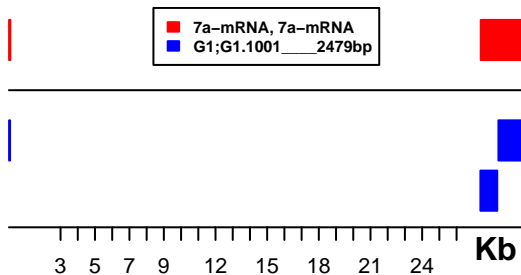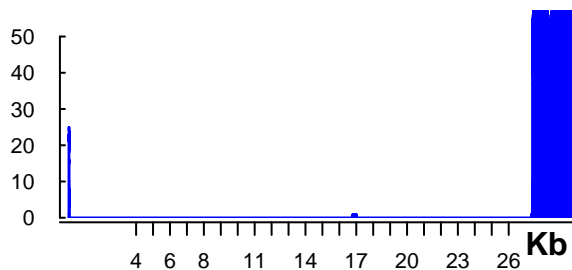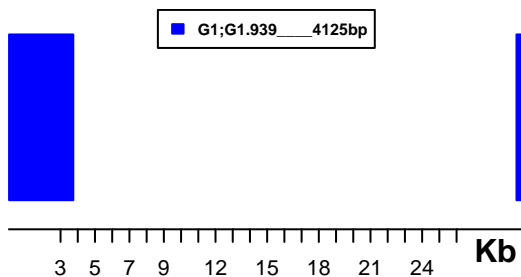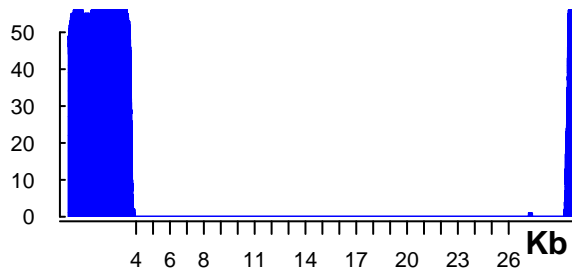

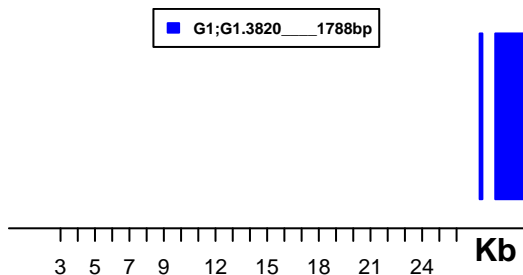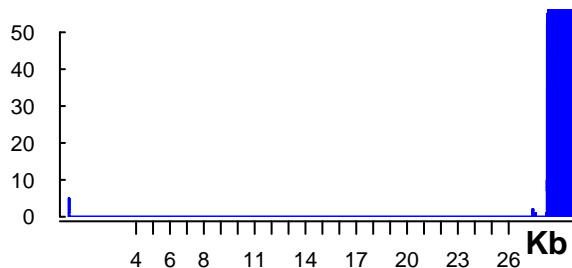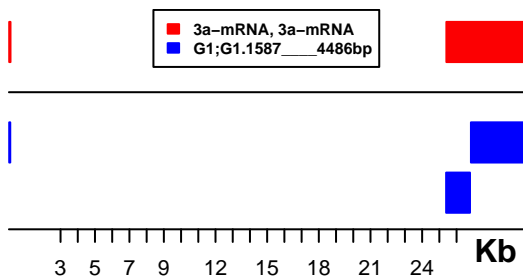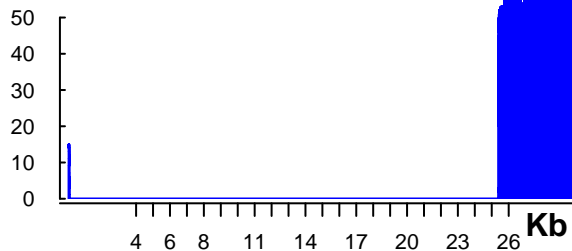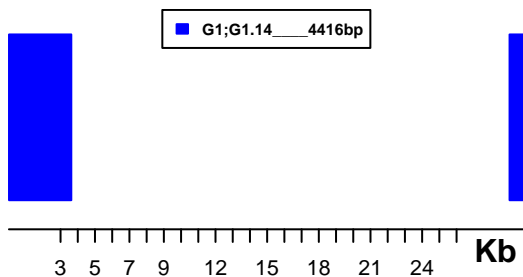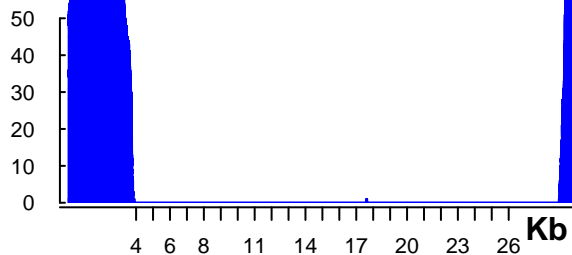

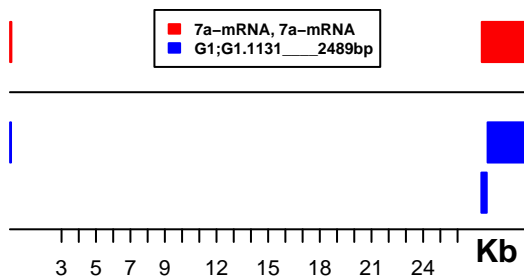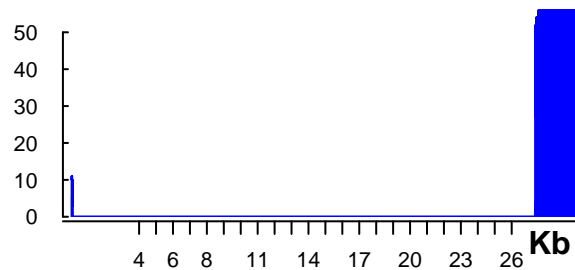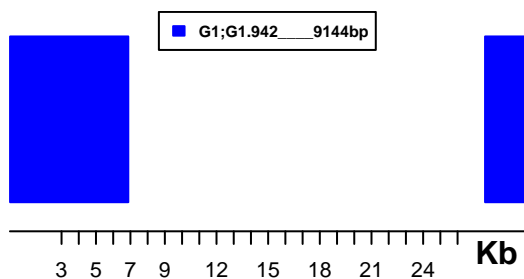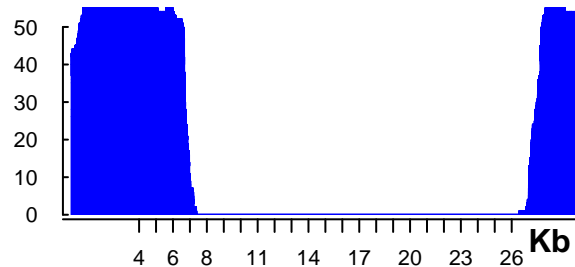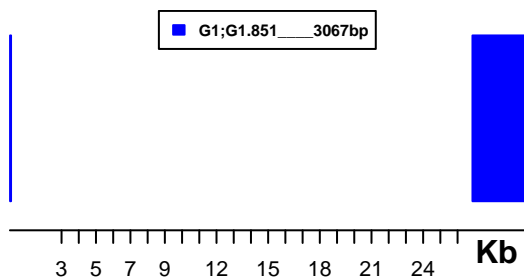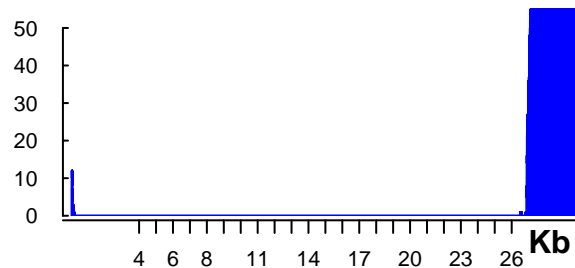

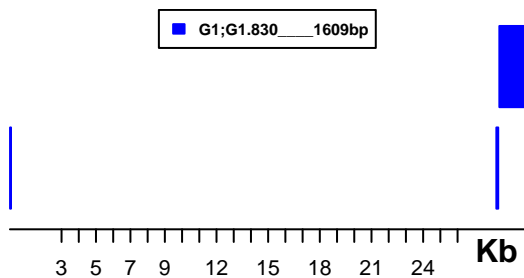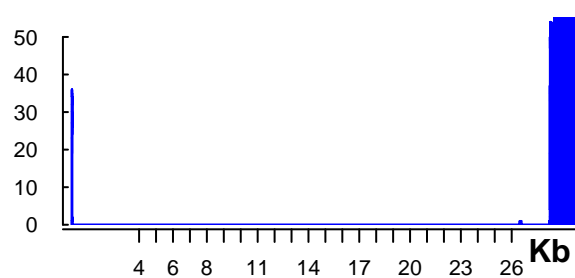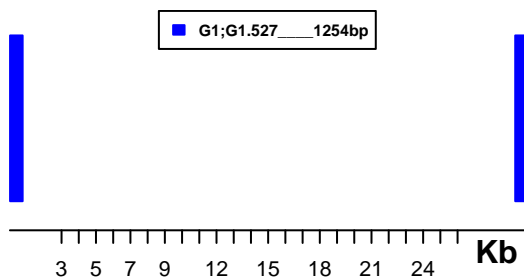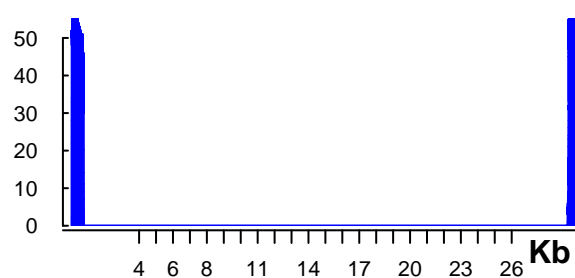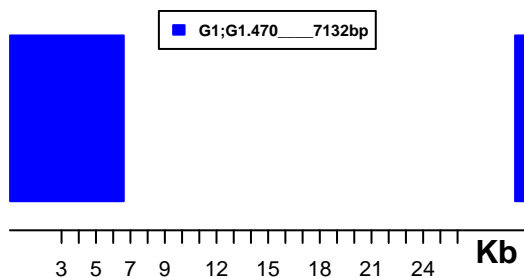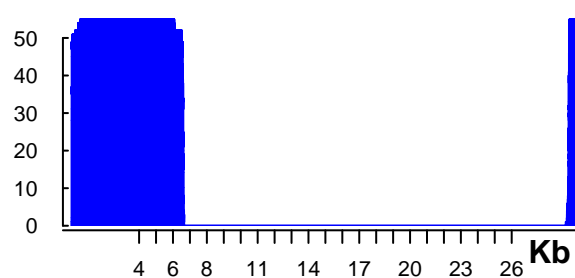

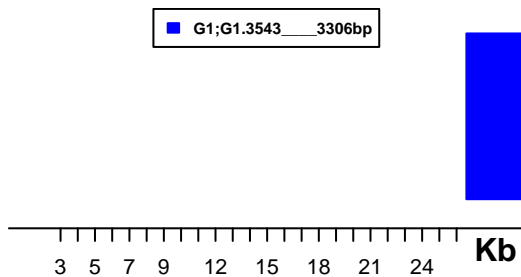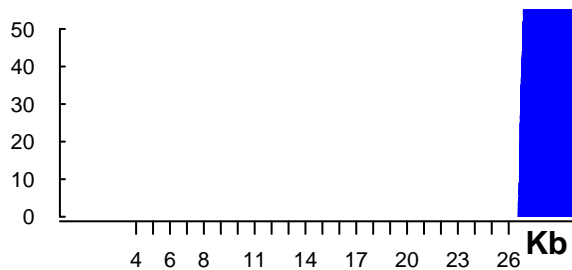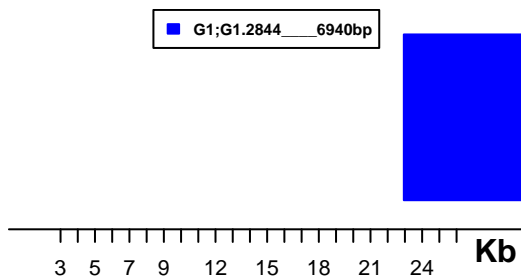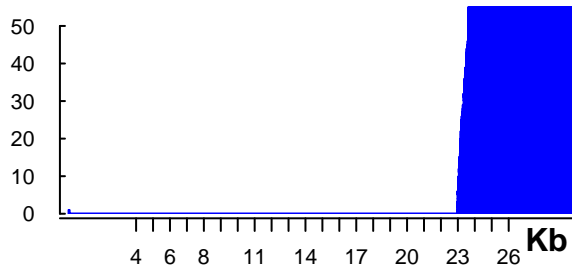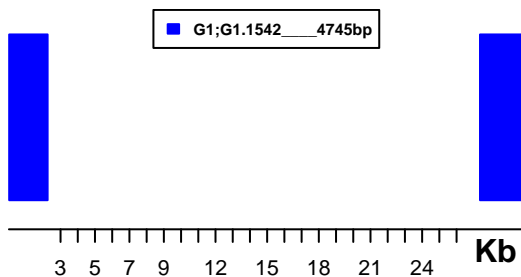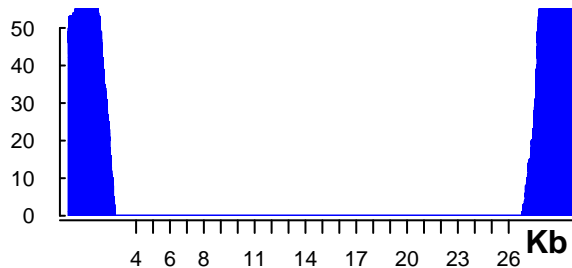

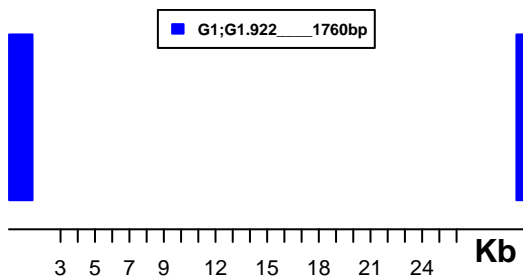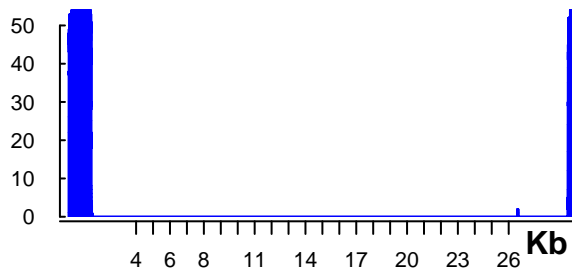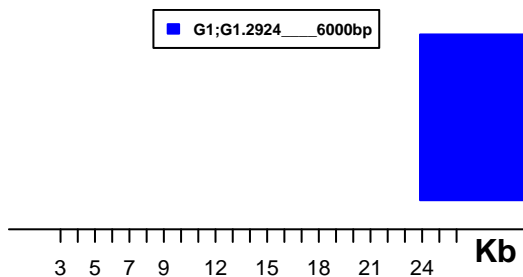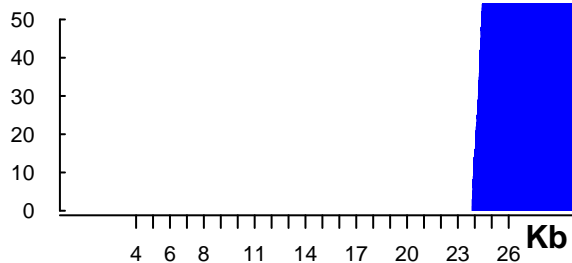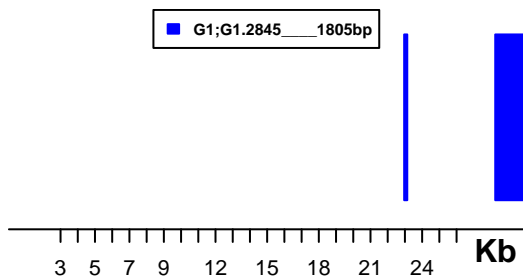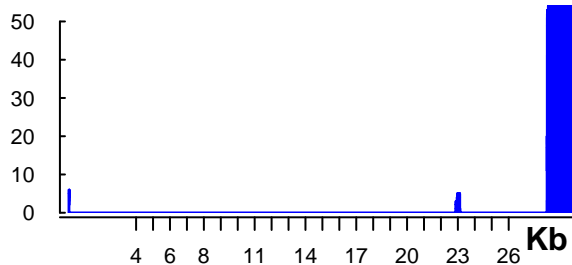

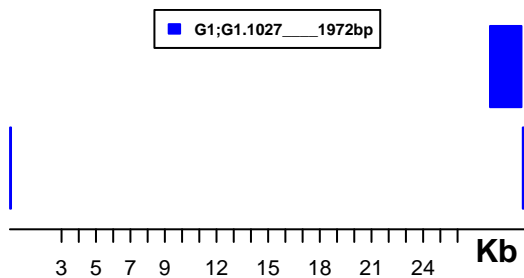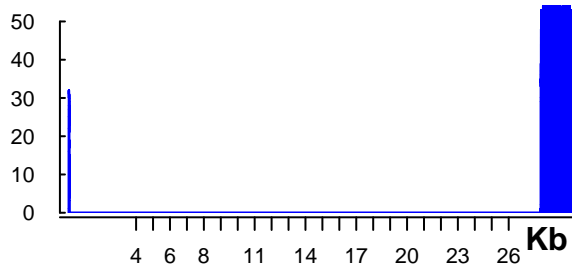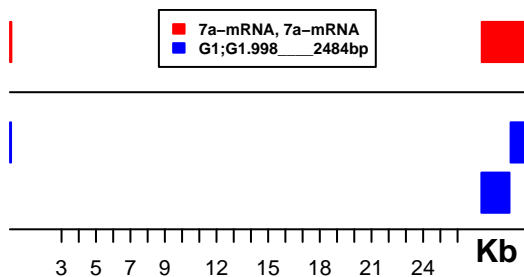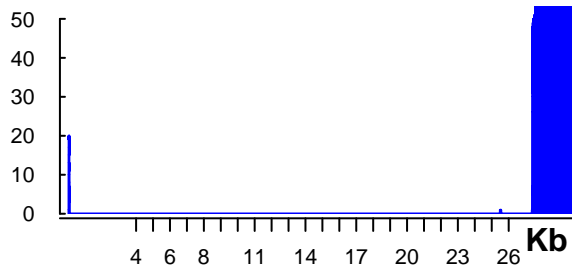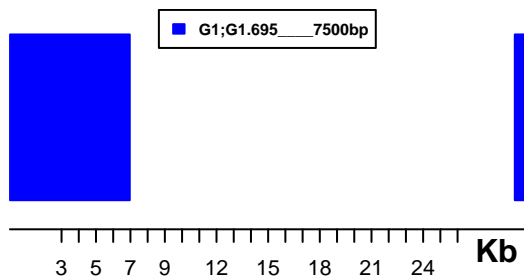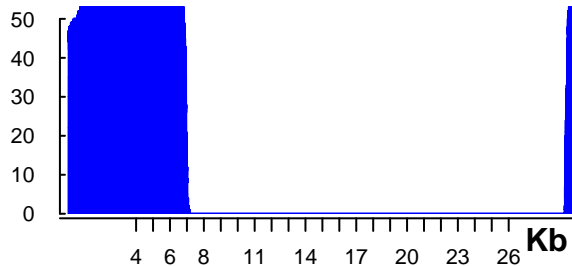

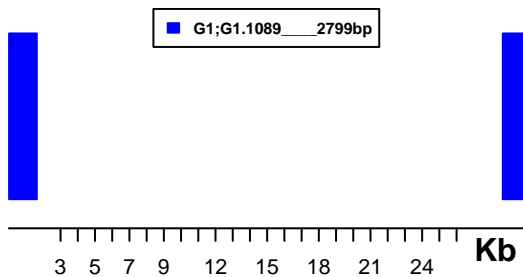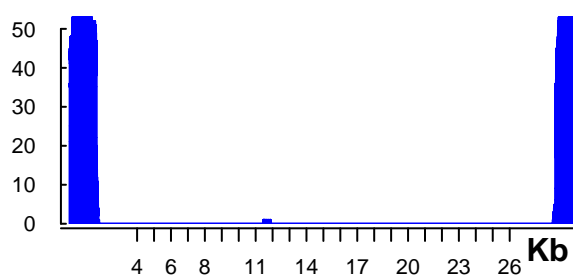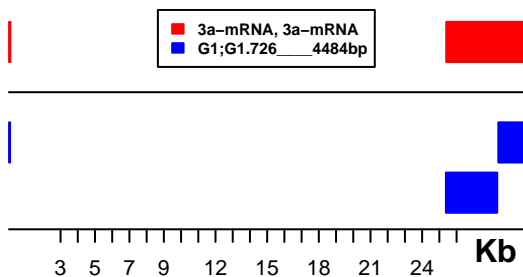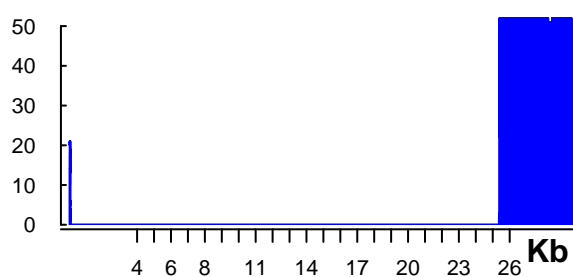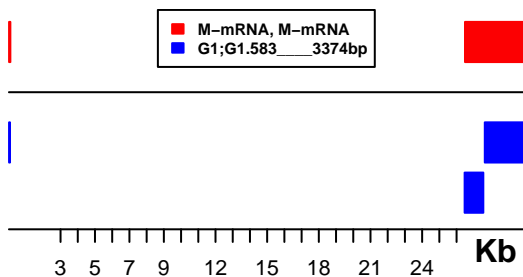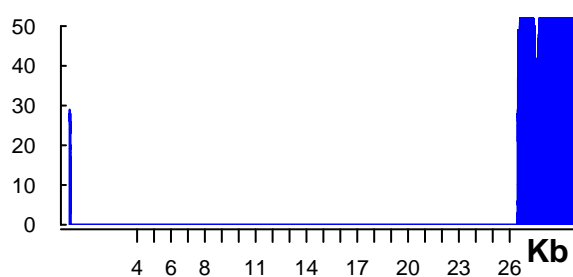

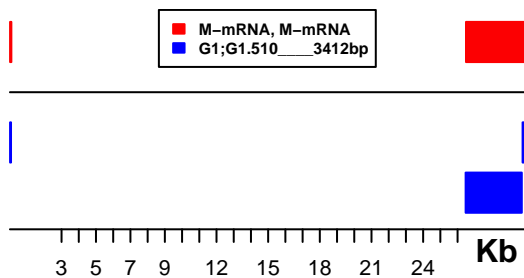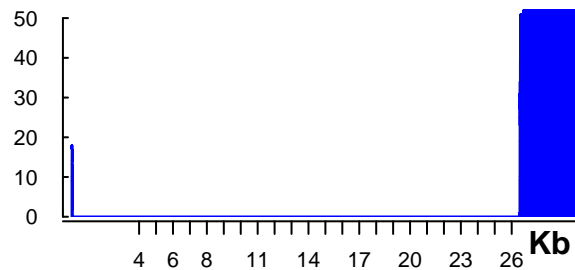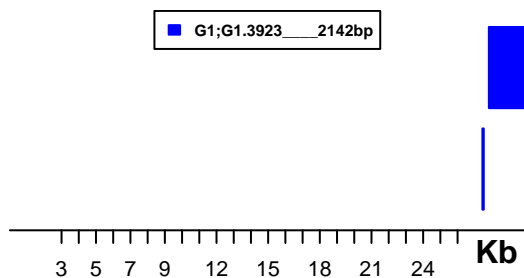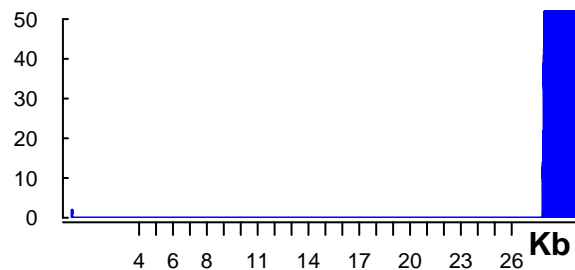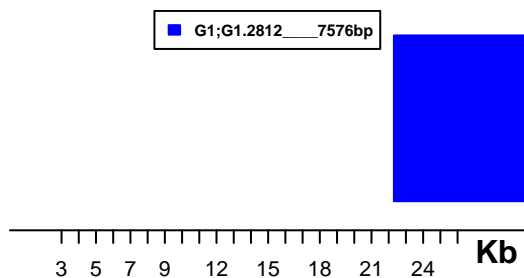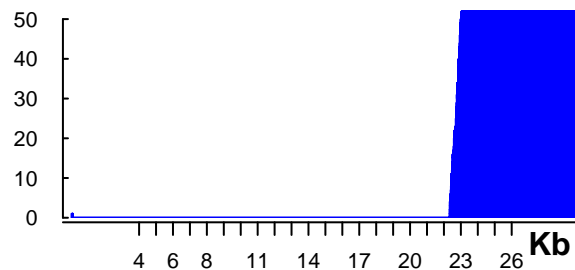

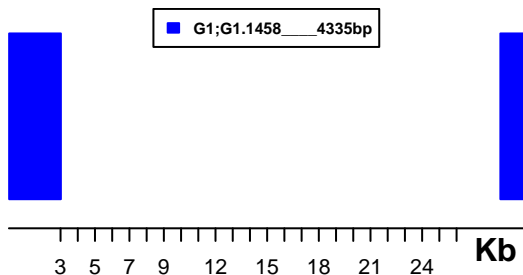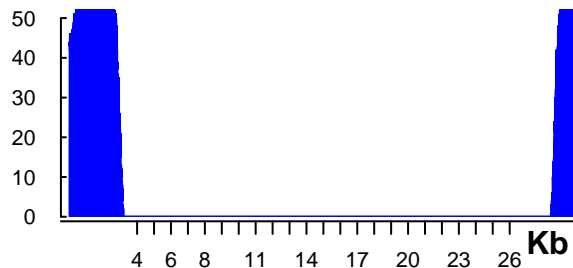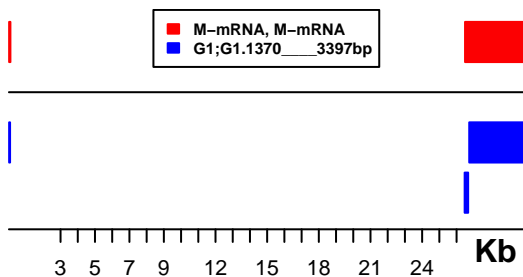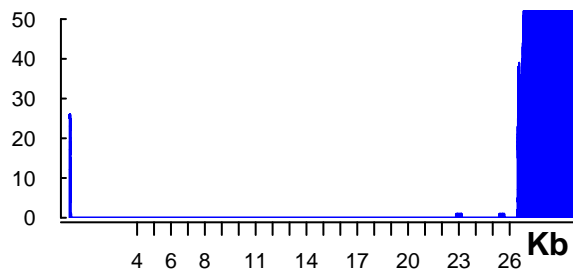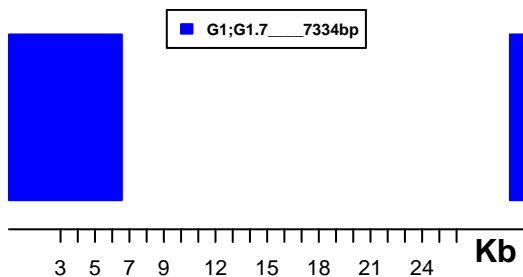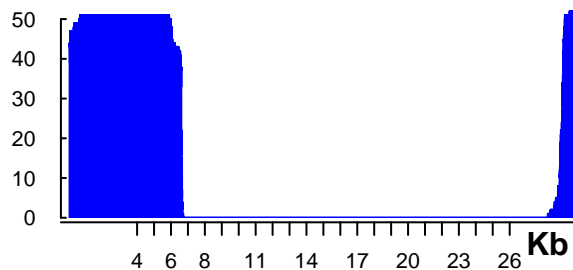

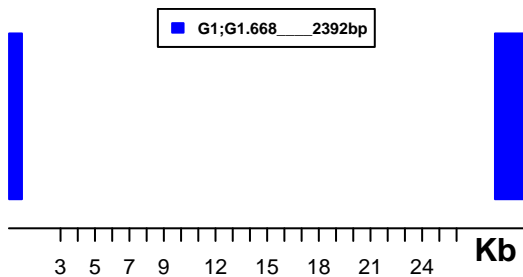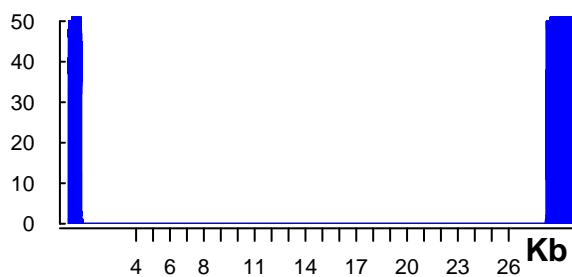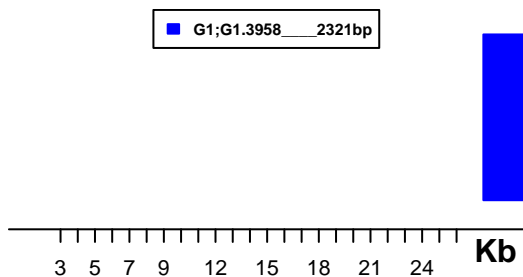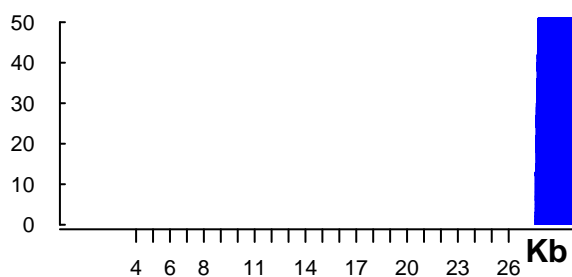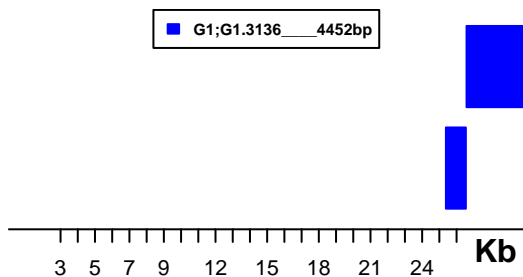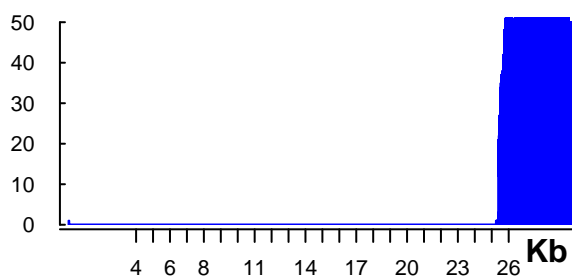

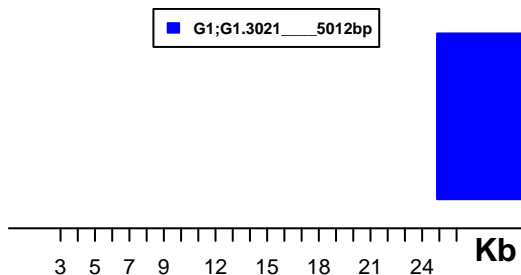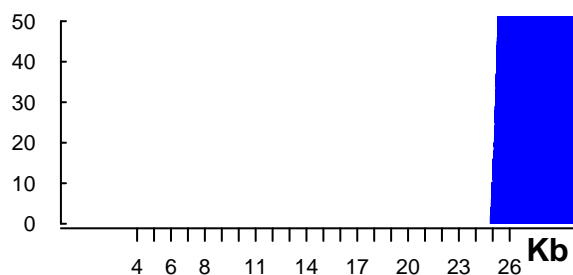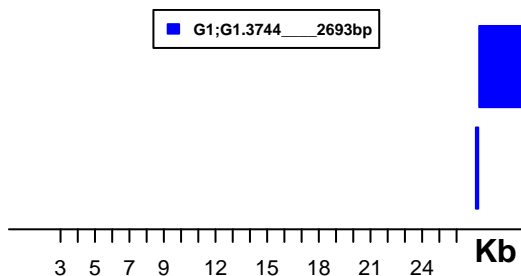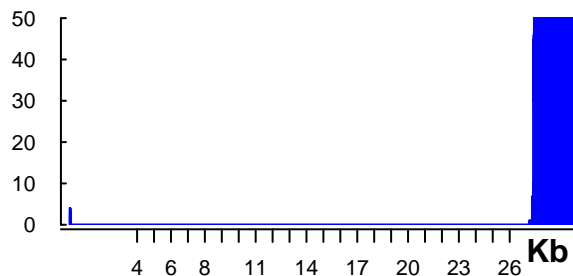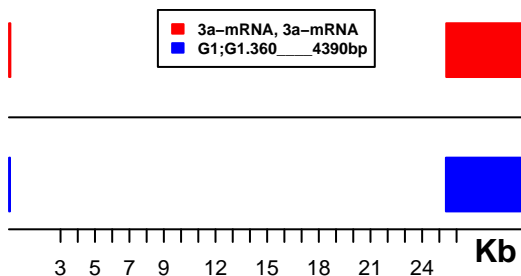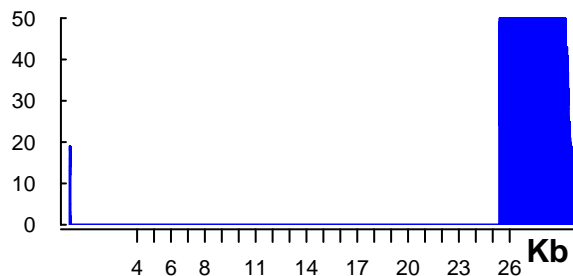

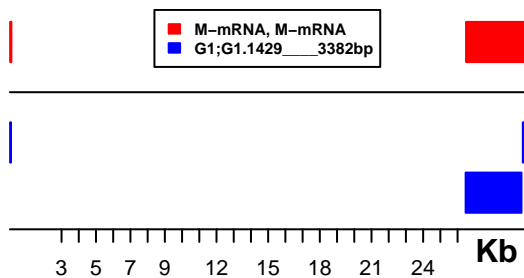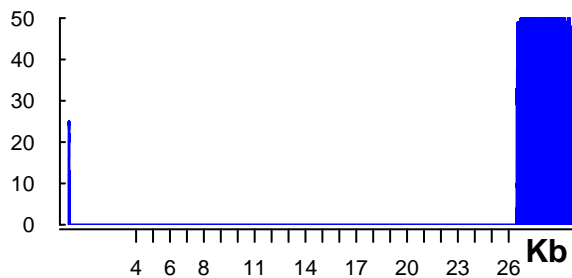

Supplement: Supplementary file 7 — Supplementary Data 5 [file 42003_2022_4058_MOESM7_ESM.zip › experiment1/Files_used_for_the_analysis_of_the_manuscript_experiment1/RNA_MODELS_WITH_COVERAGE_experiment_1_passage_14.pdf]

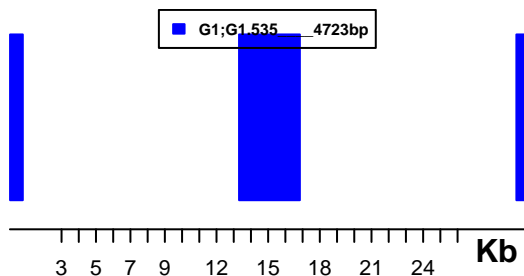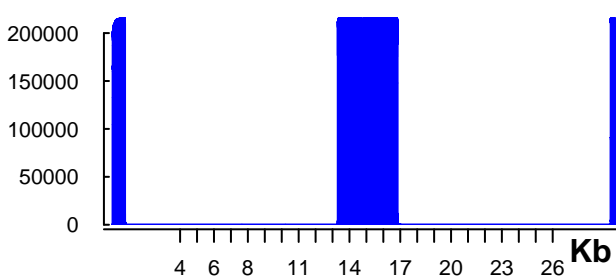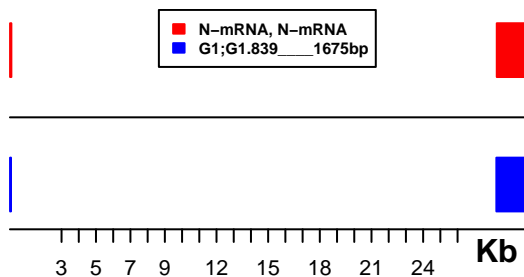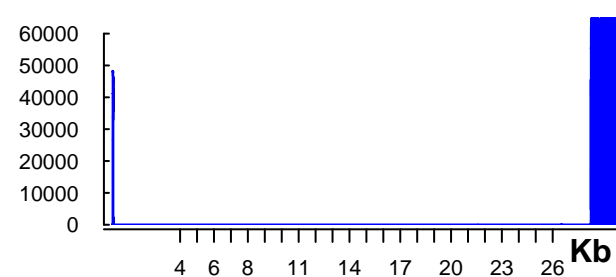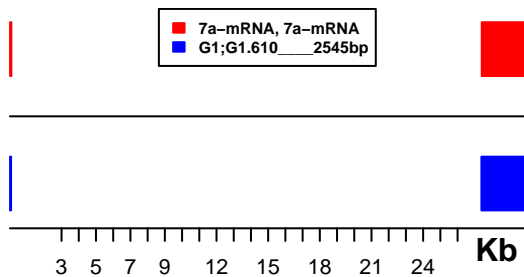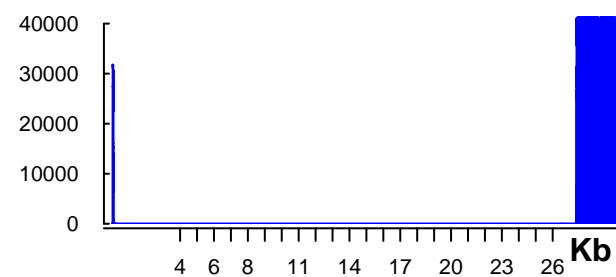

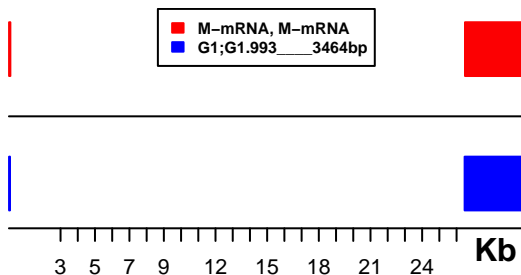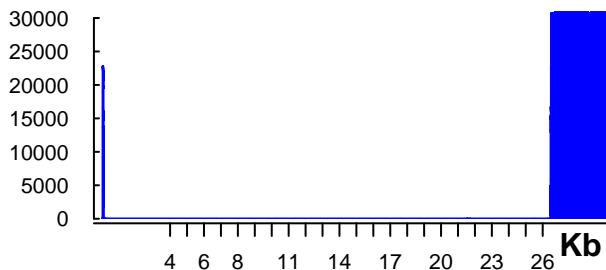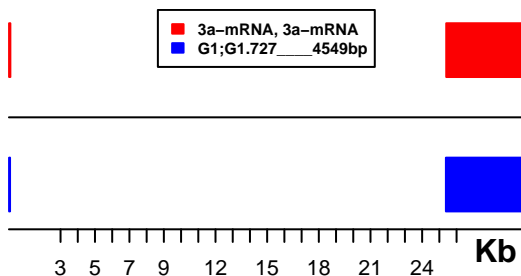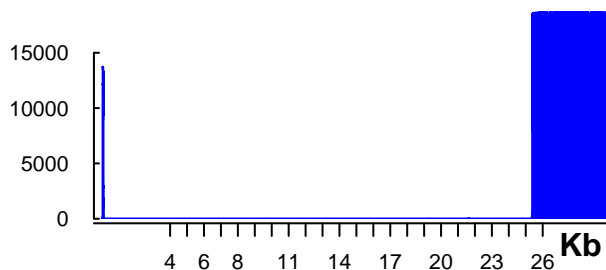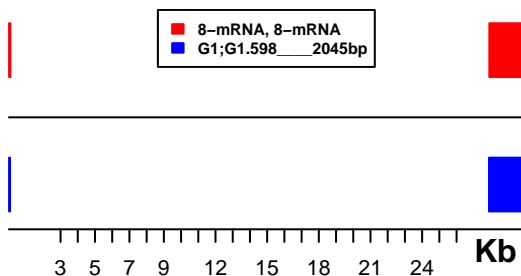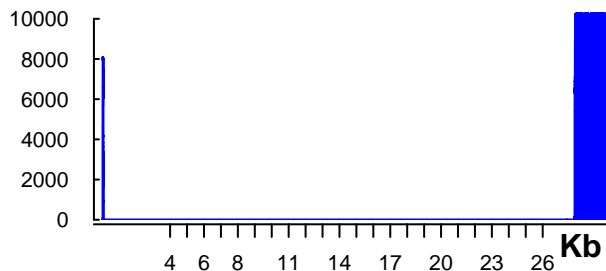

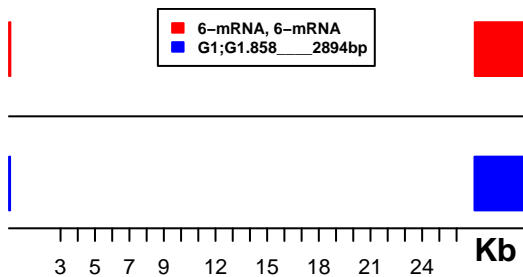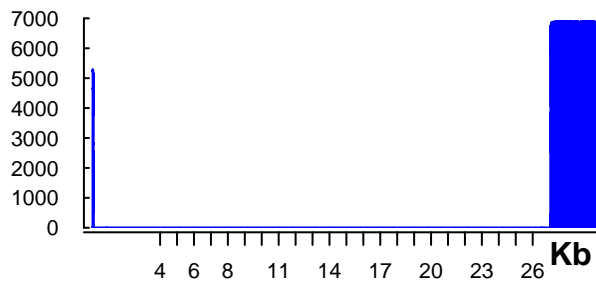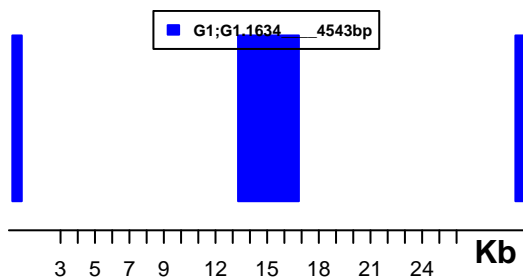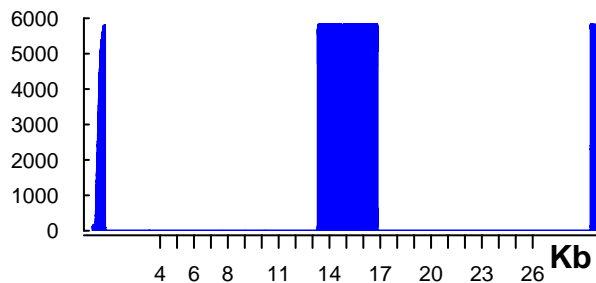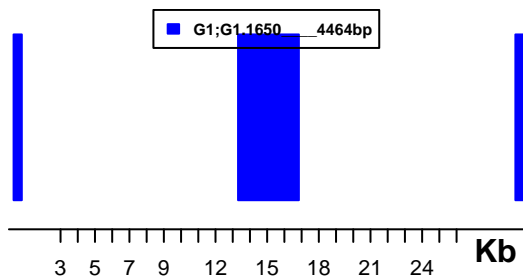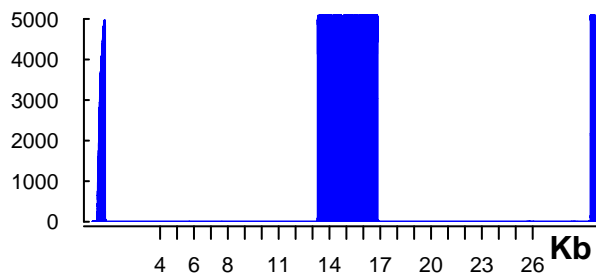

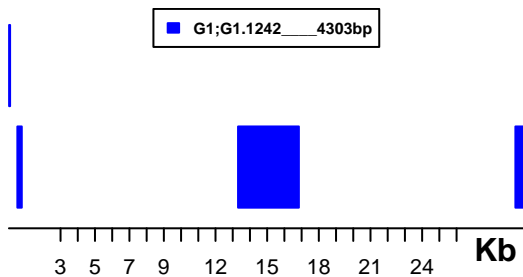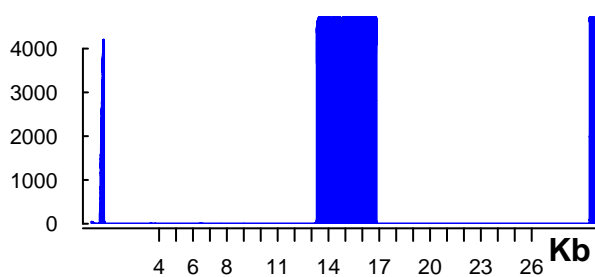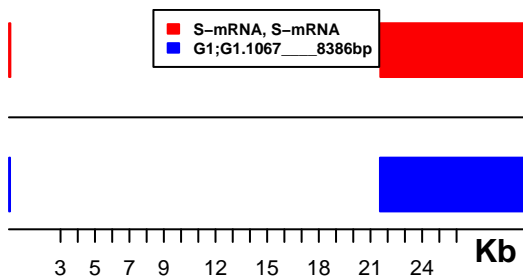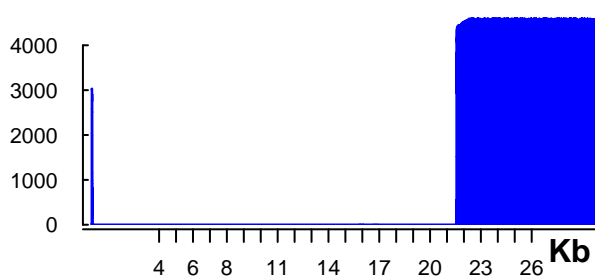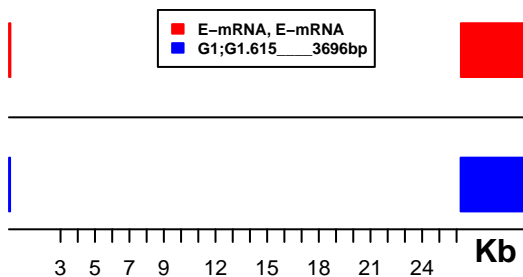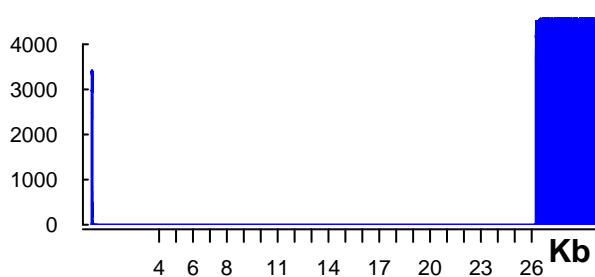

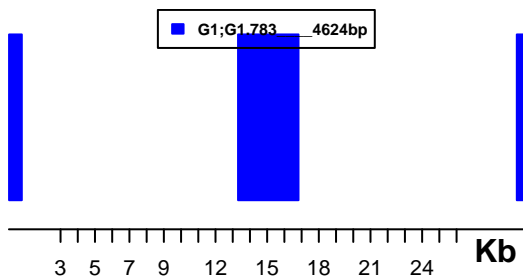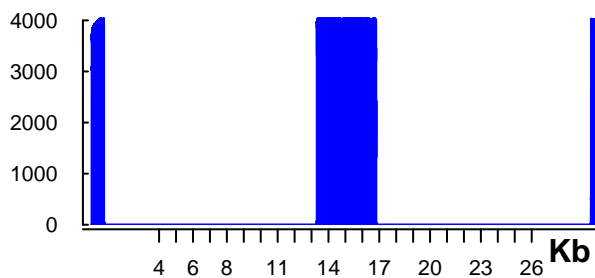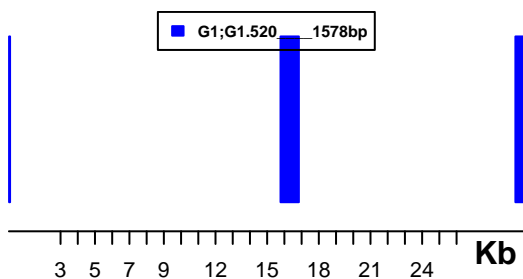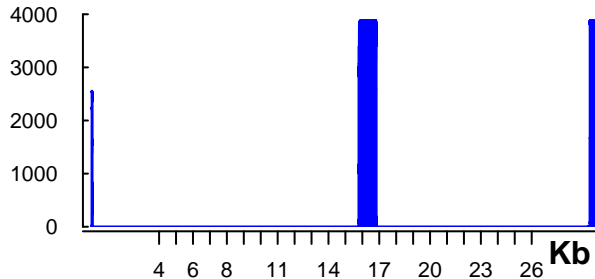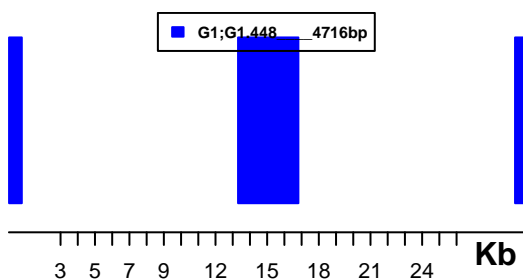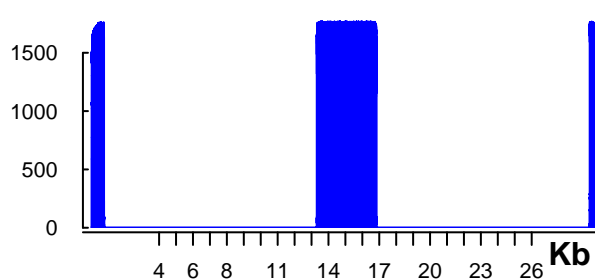

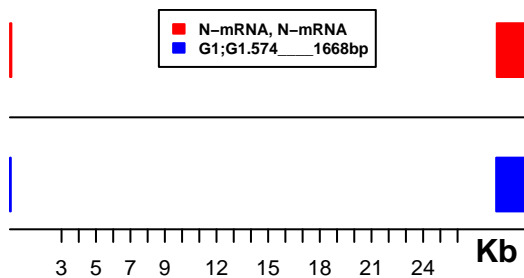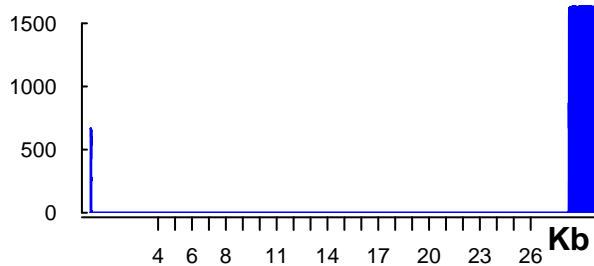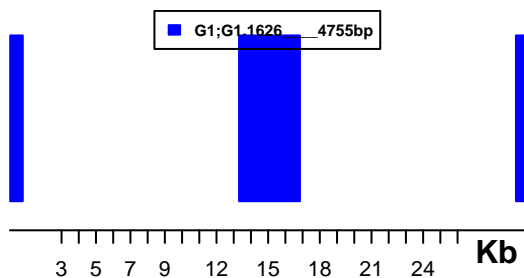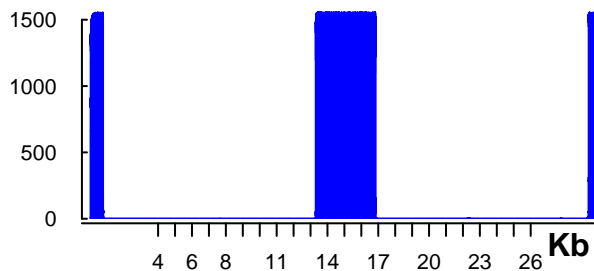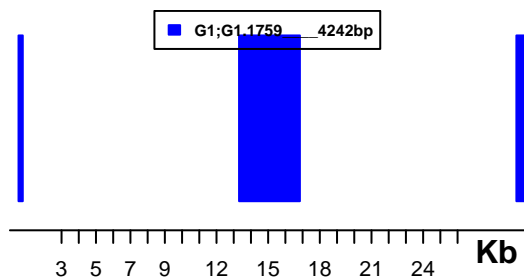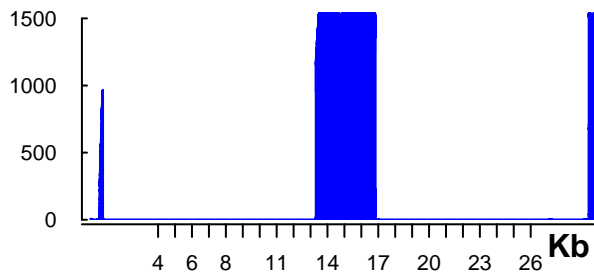

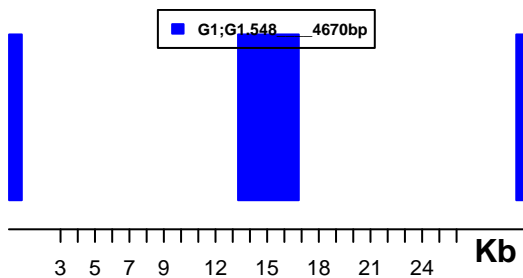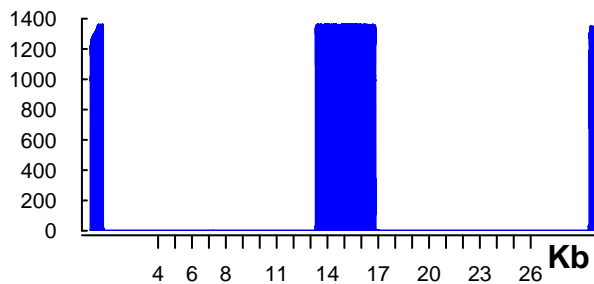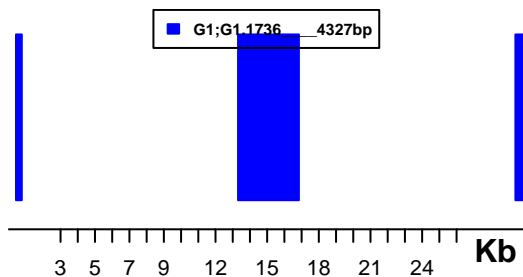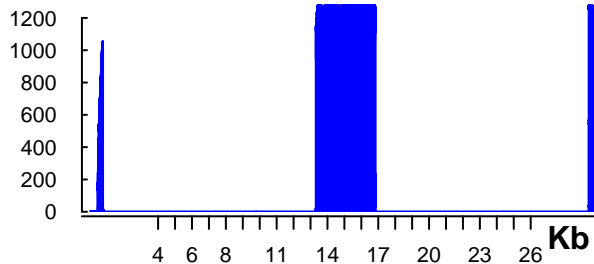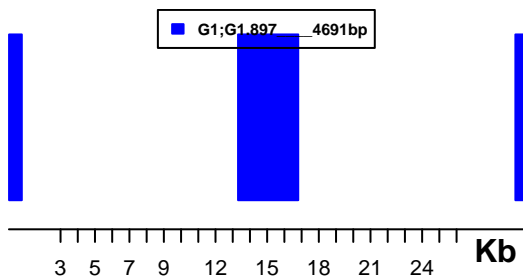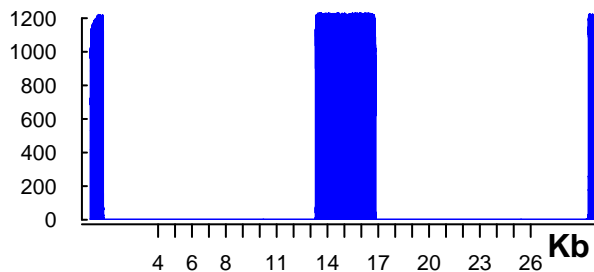

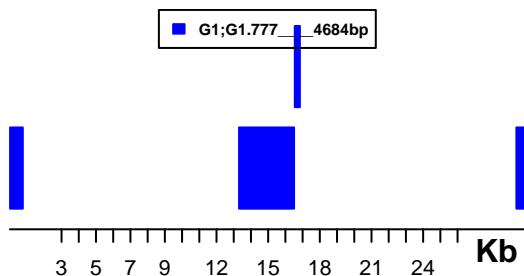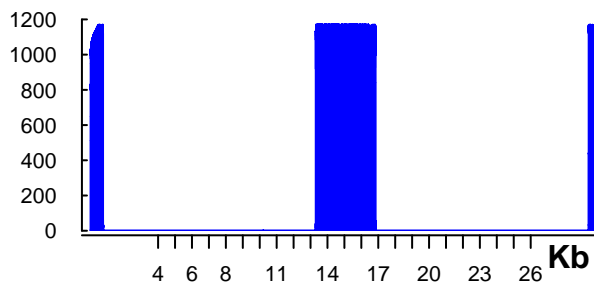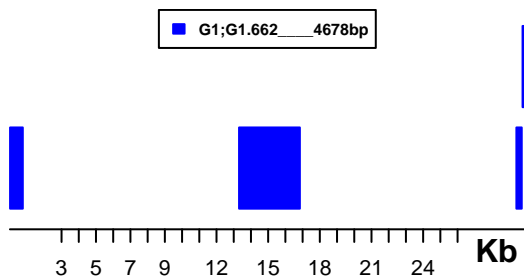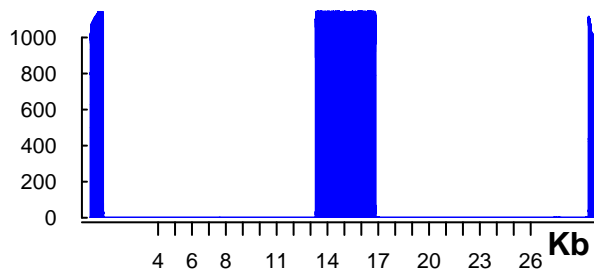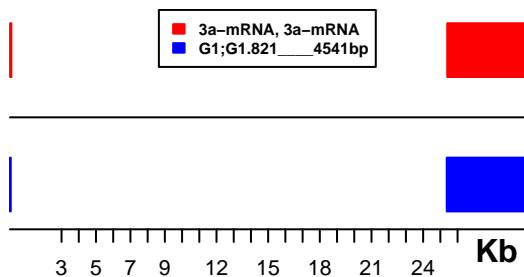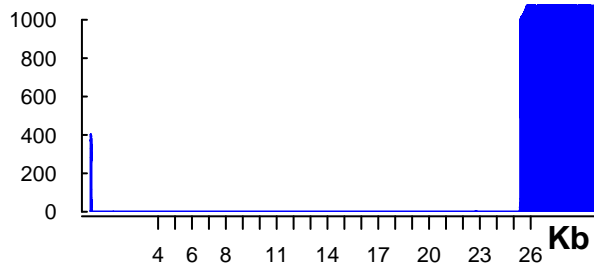

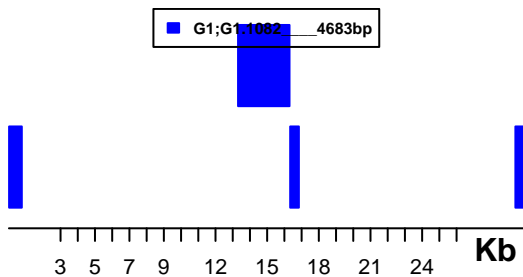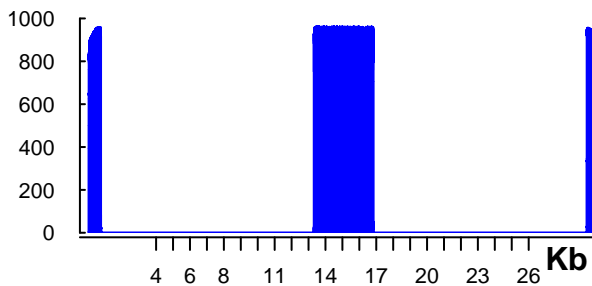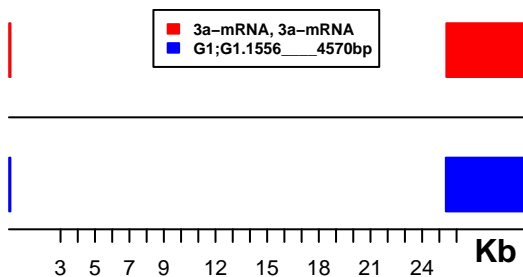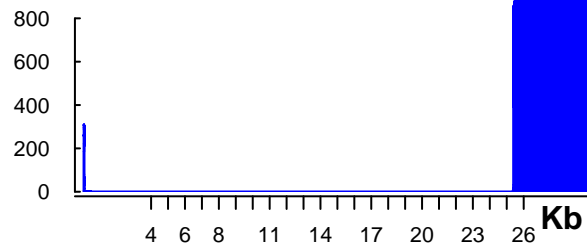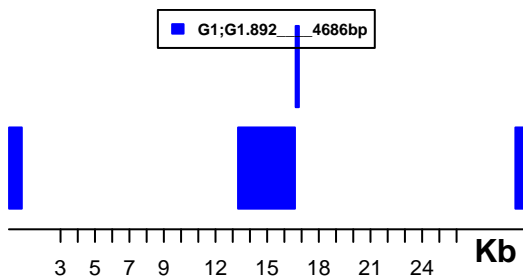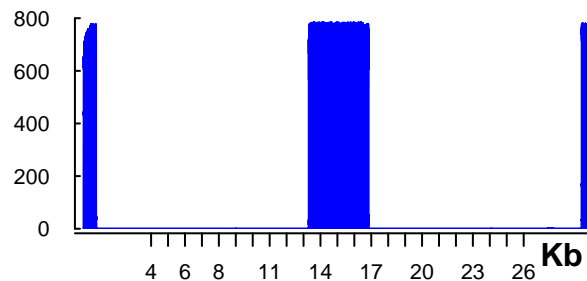

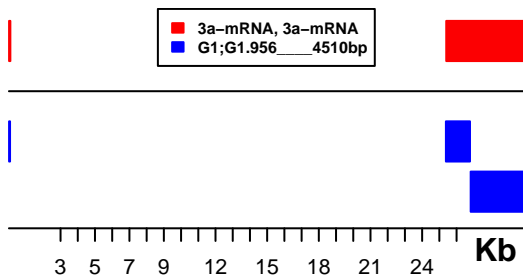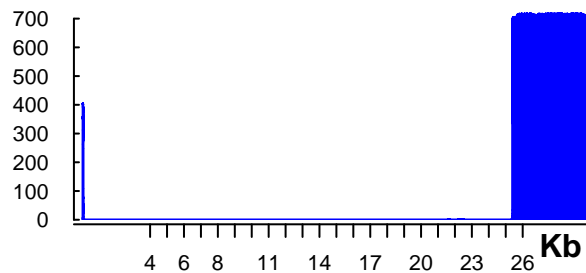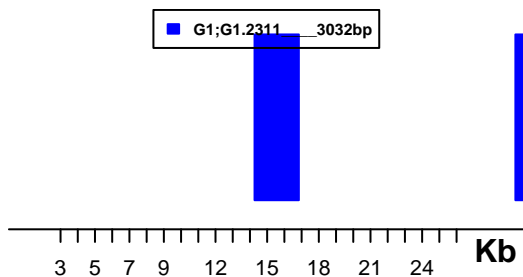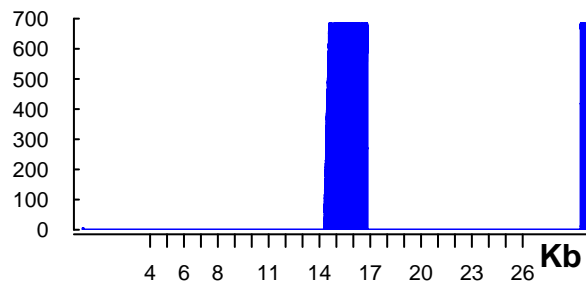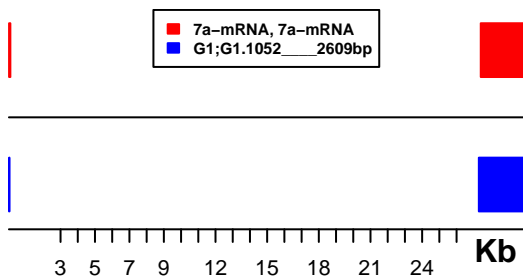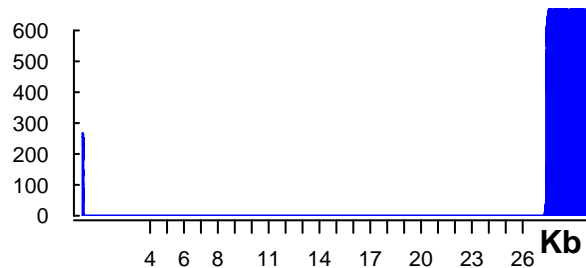

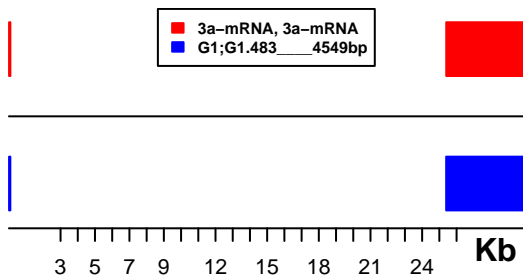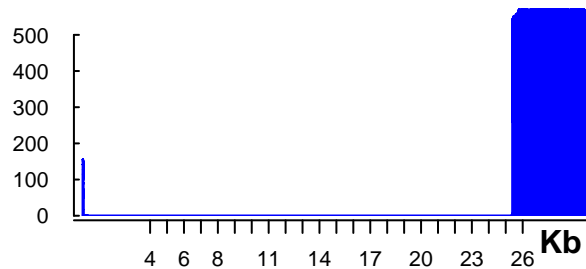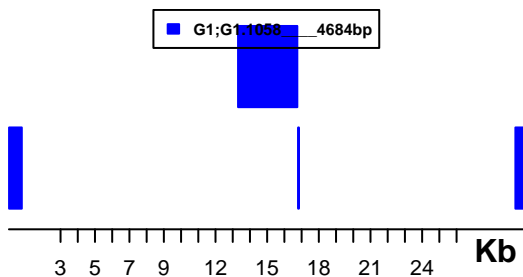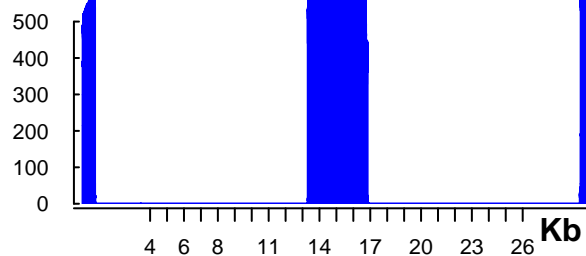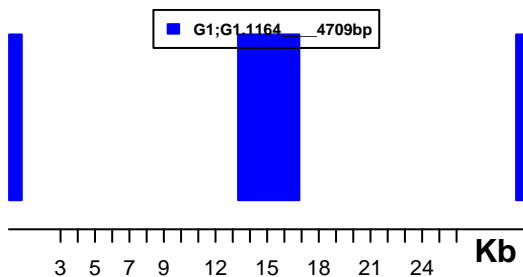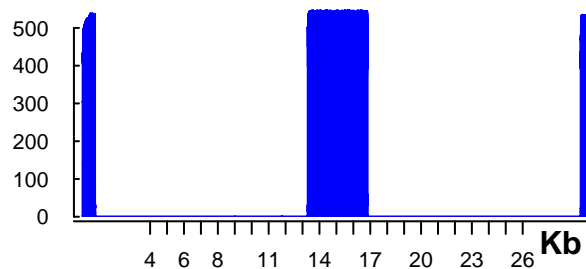

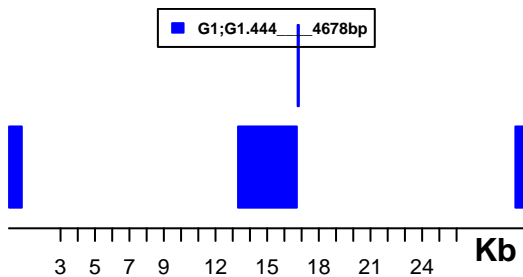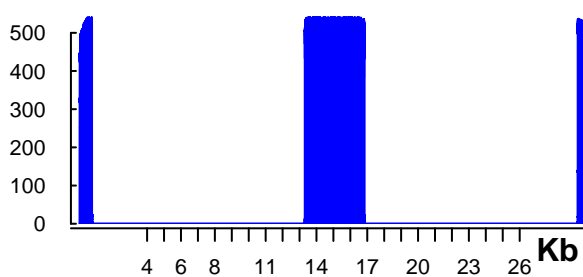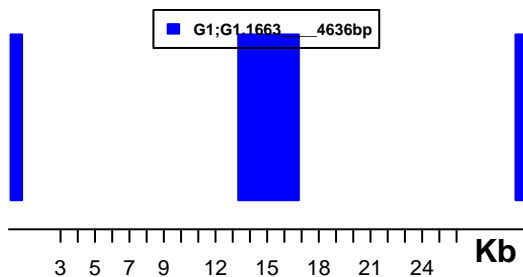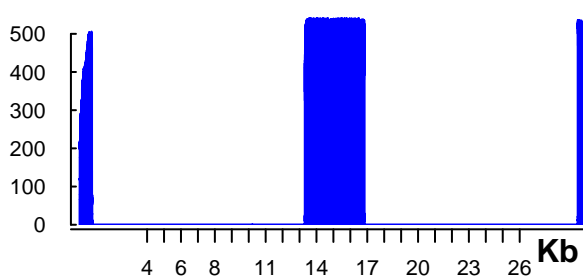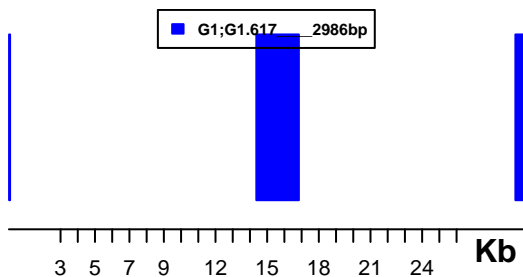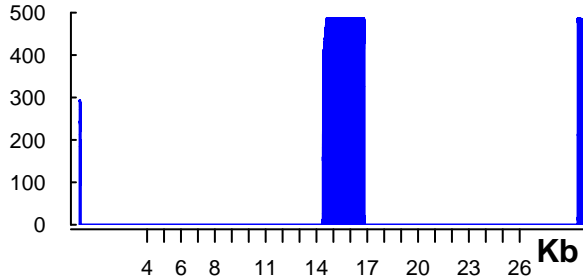

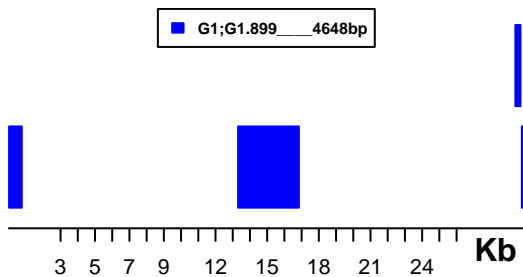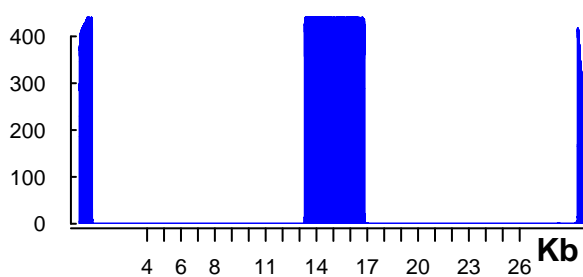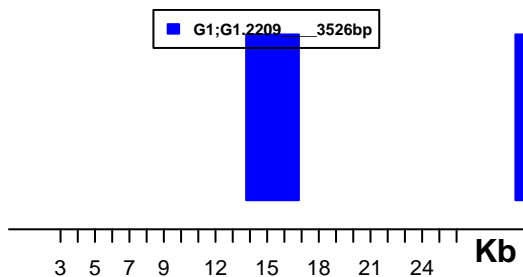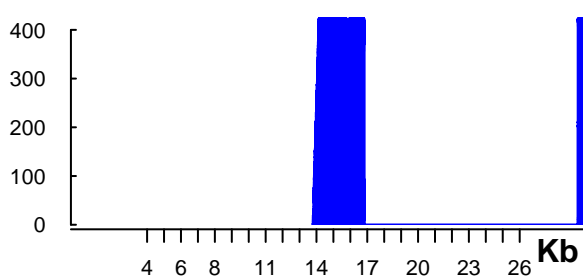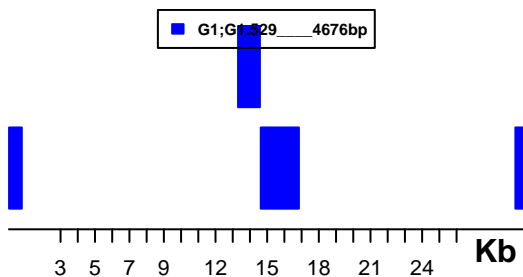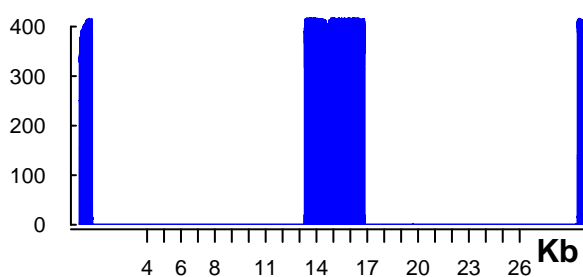

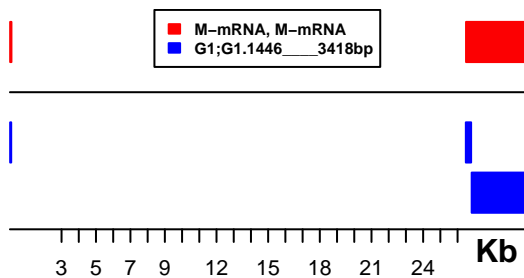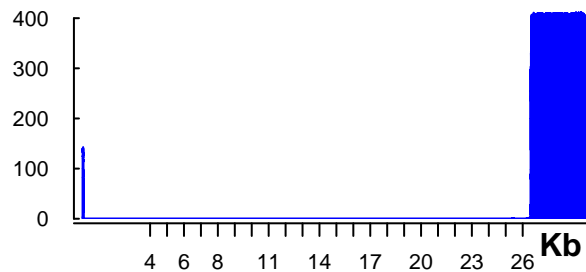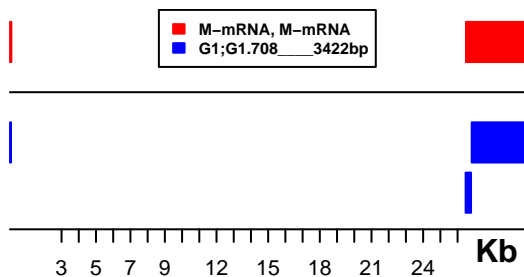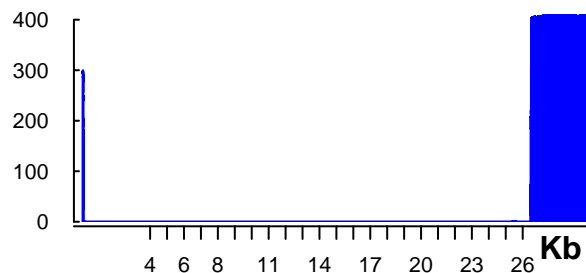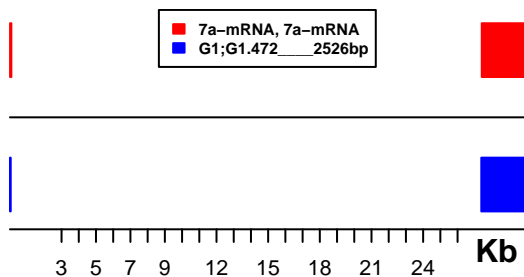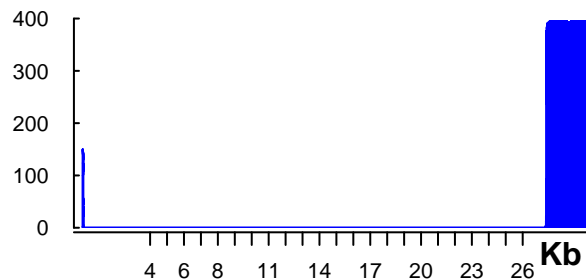

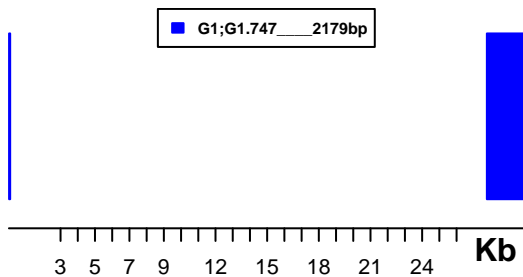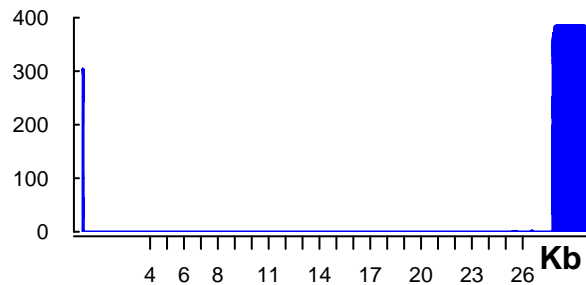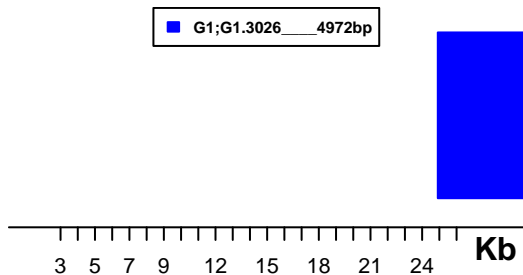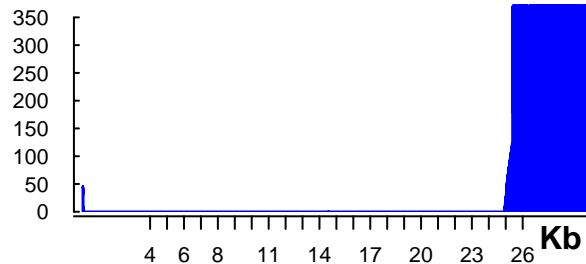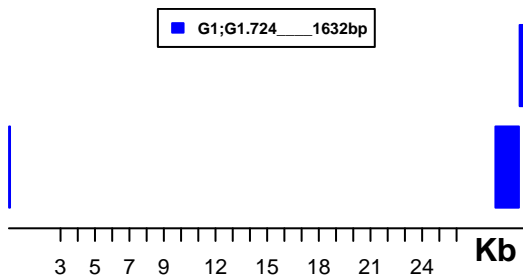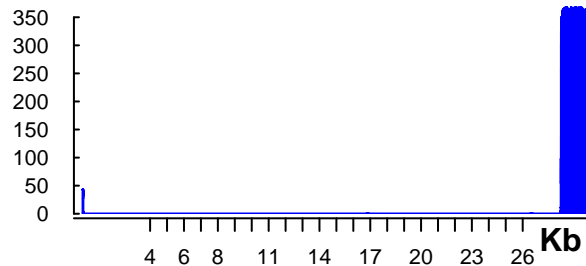

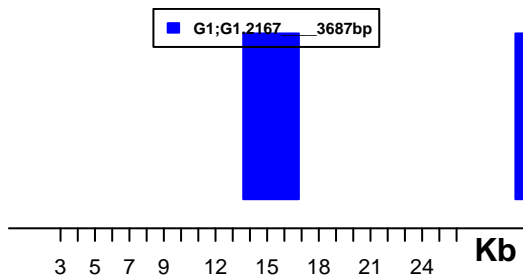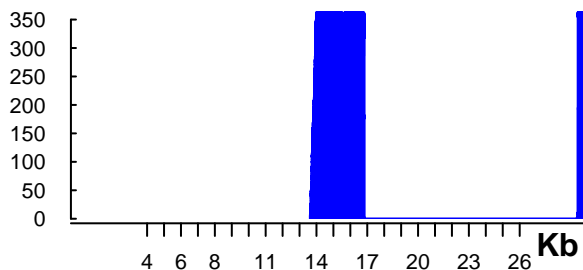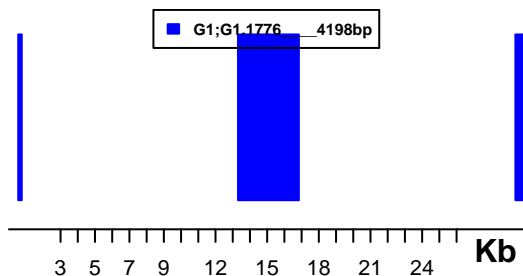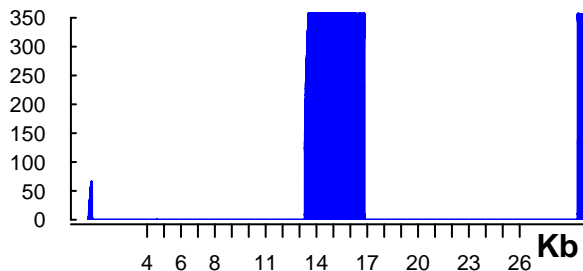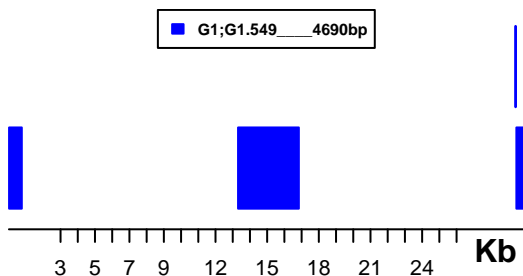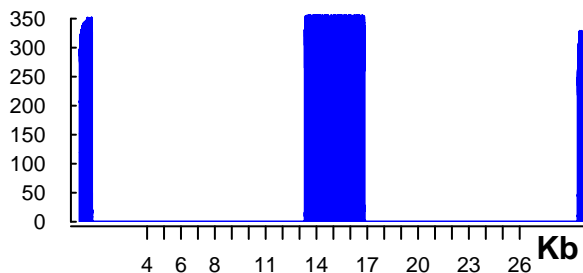

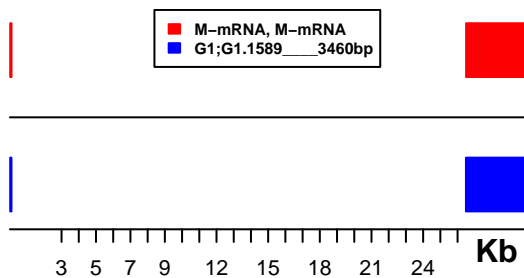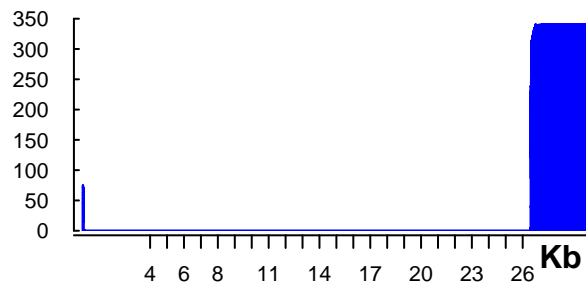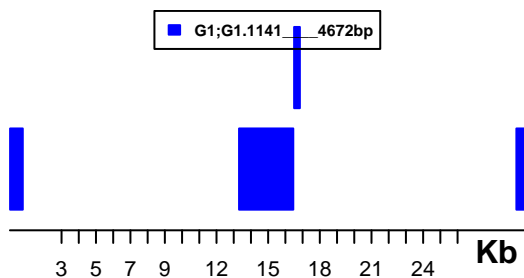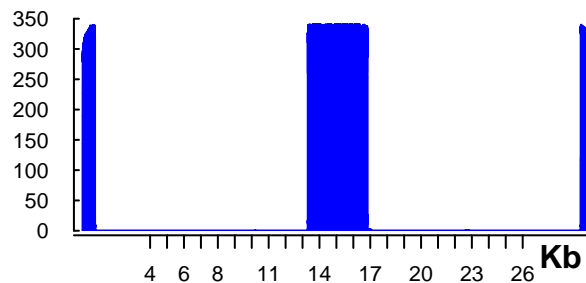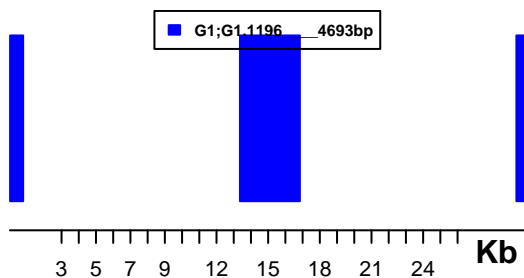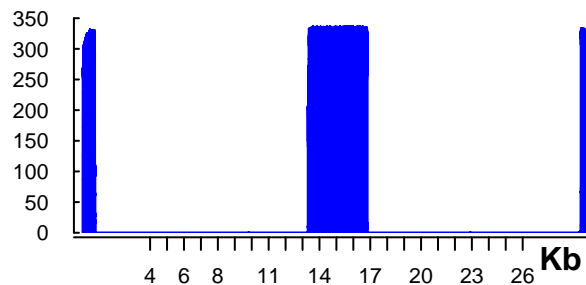

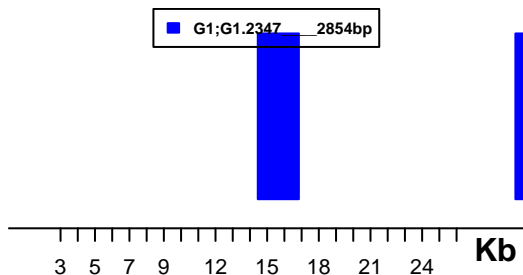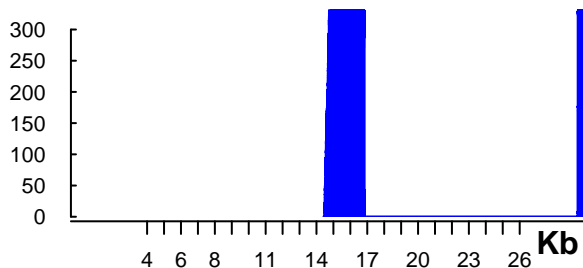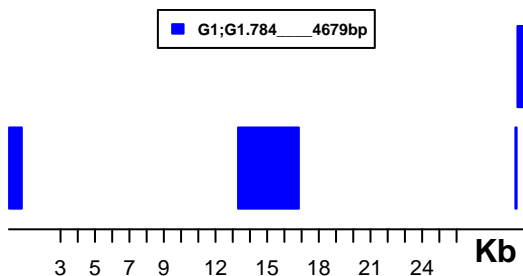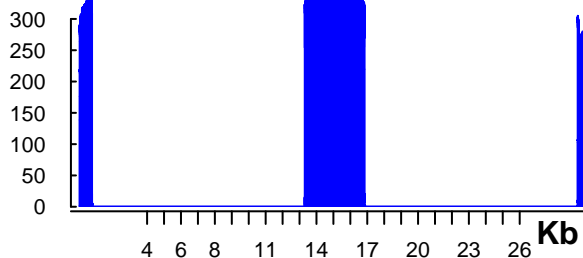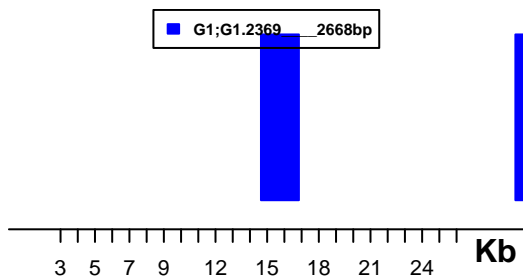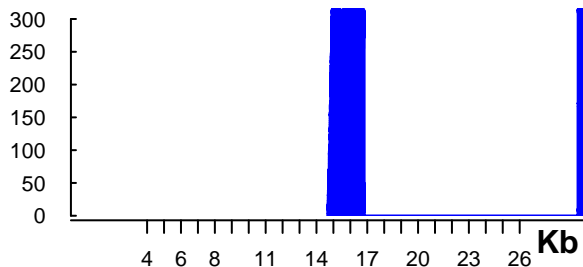

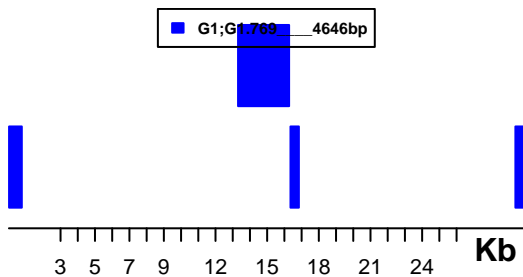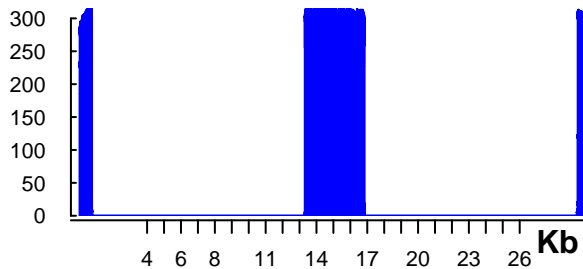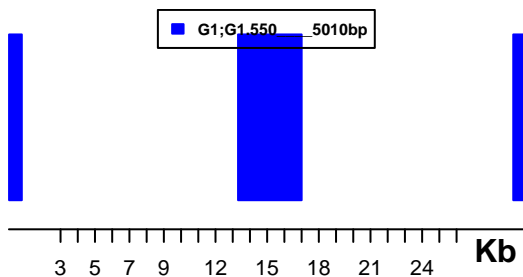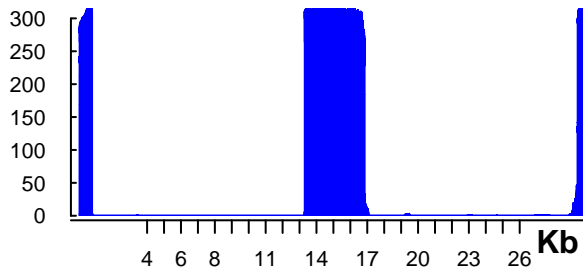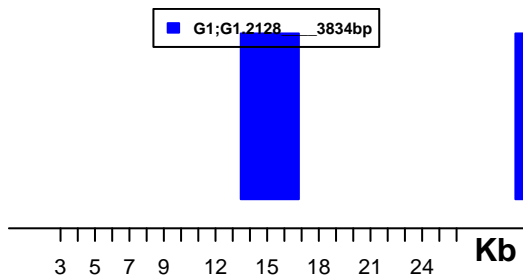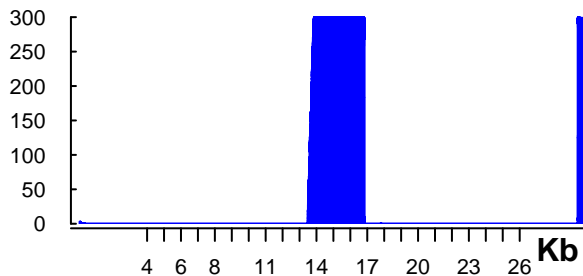

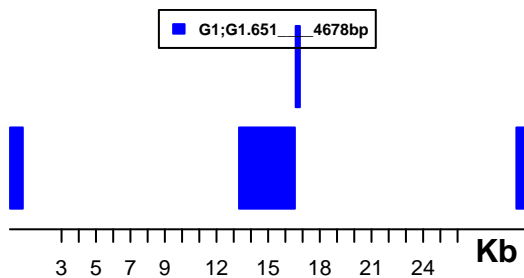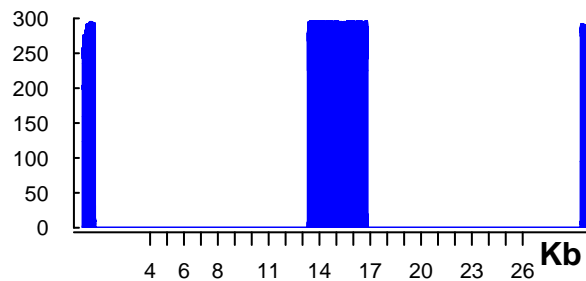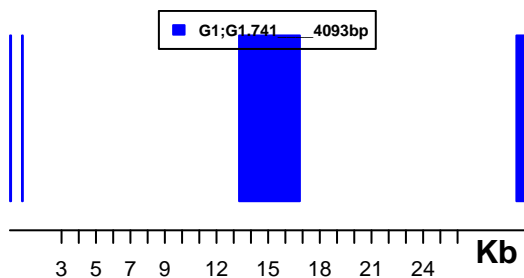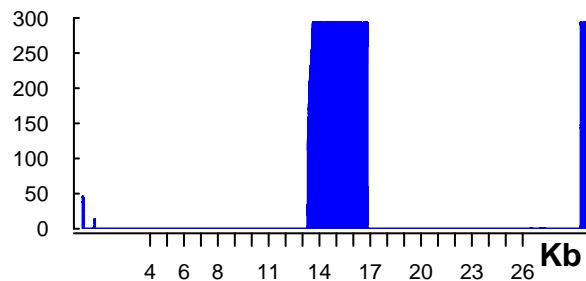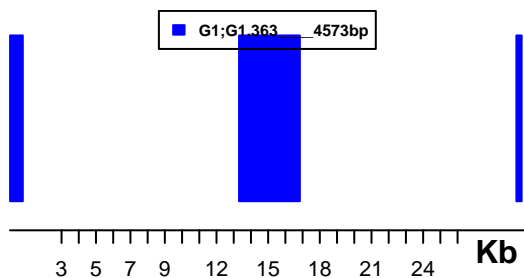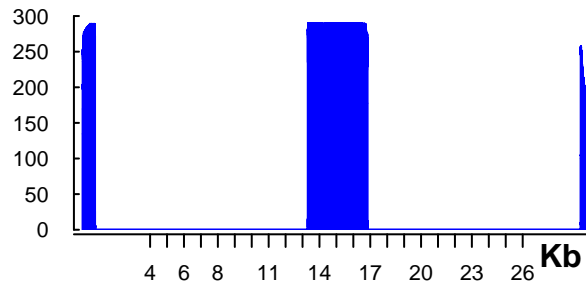

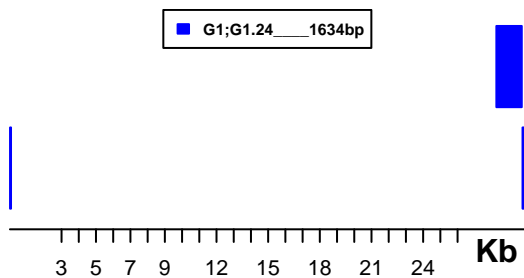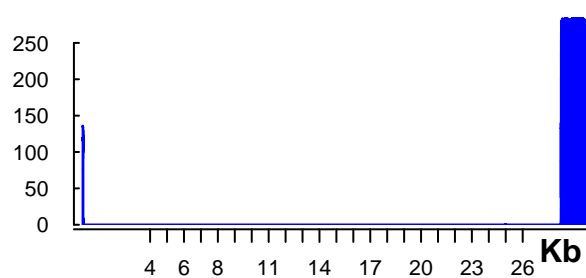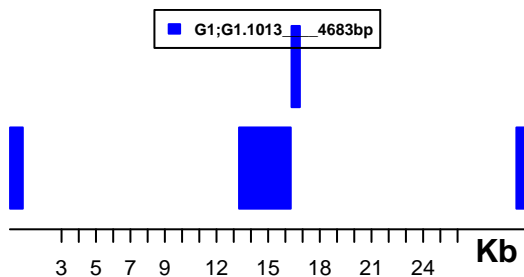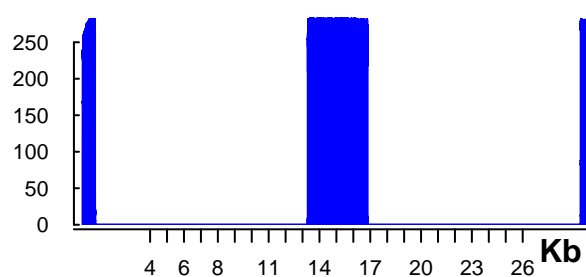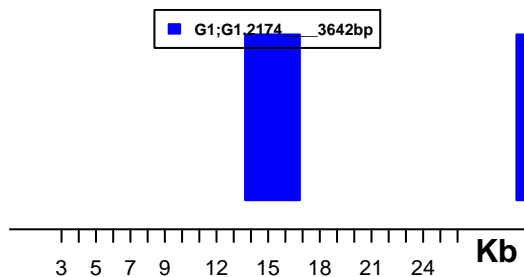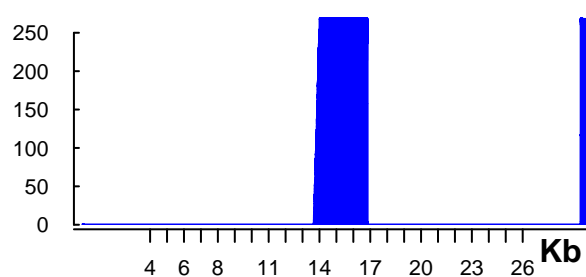

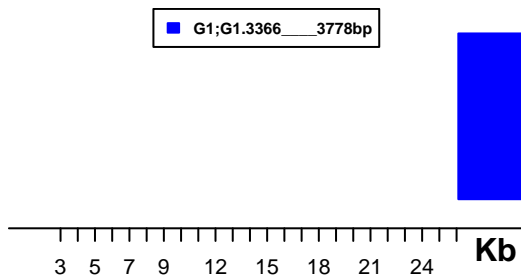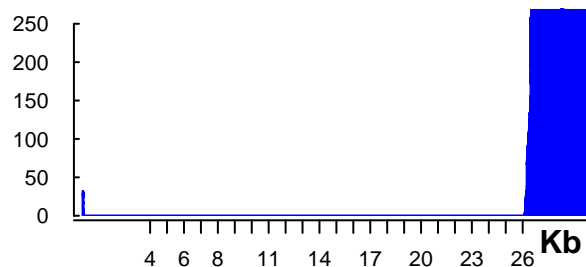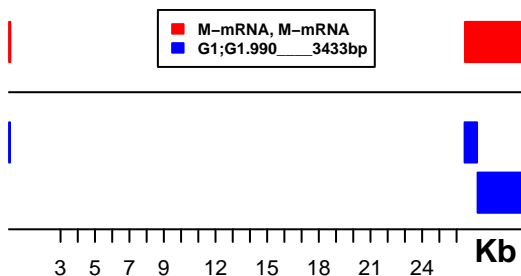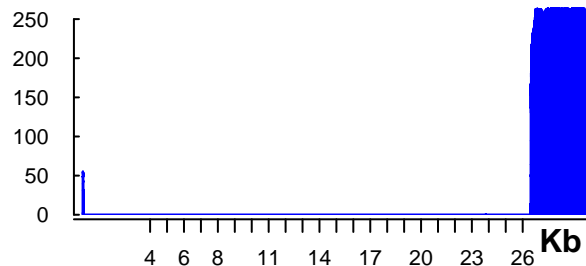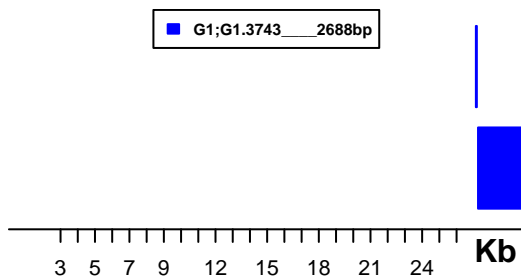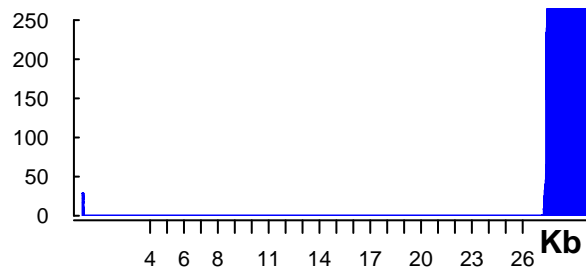

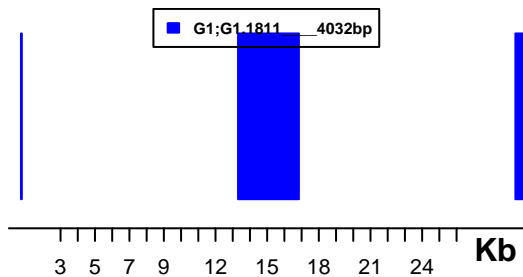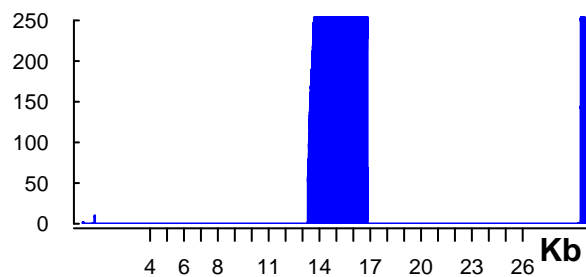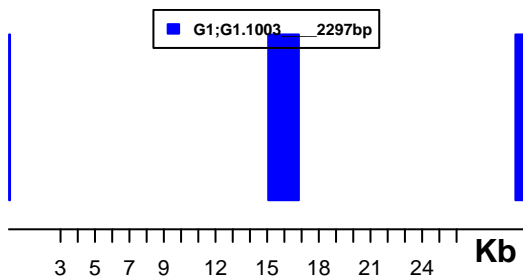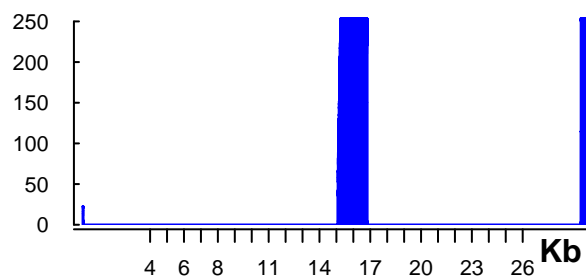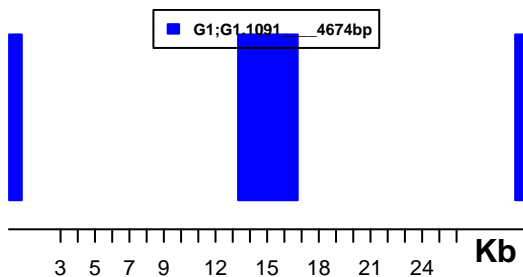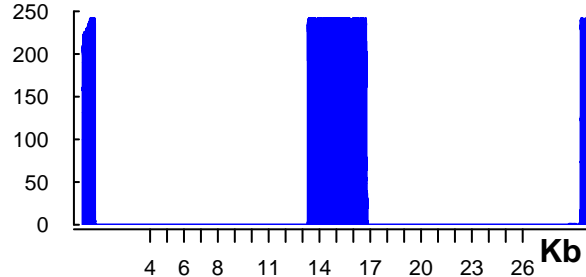

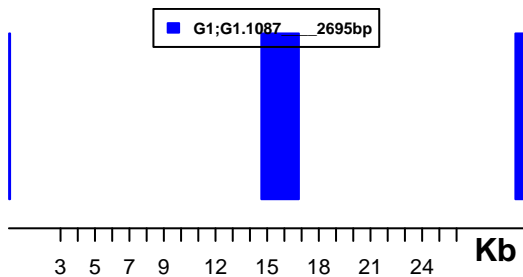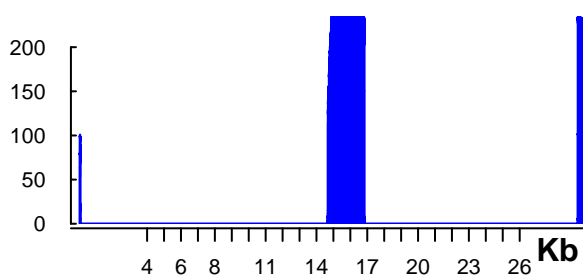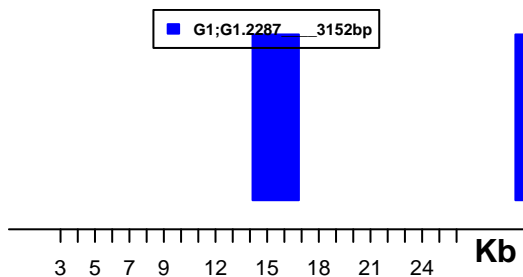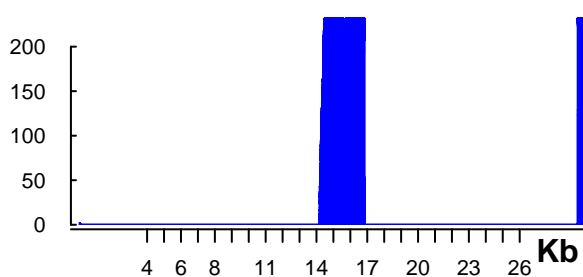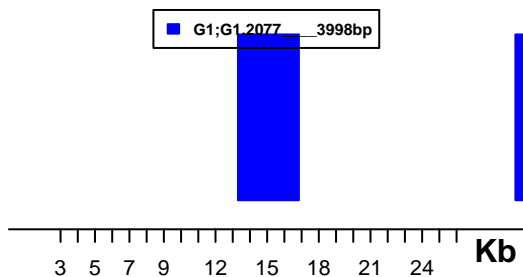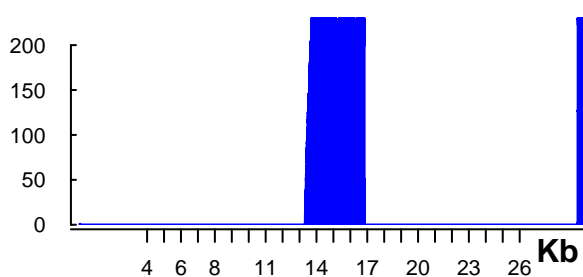

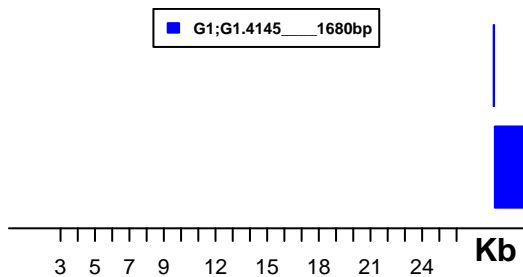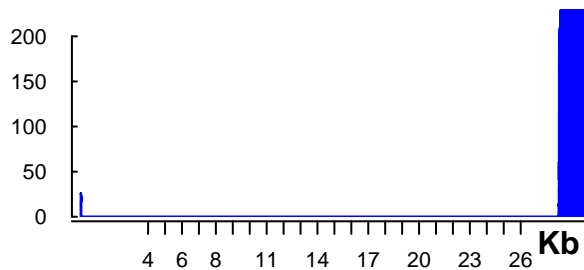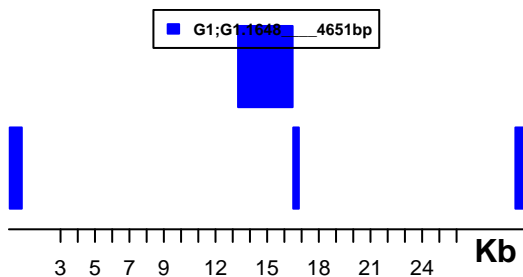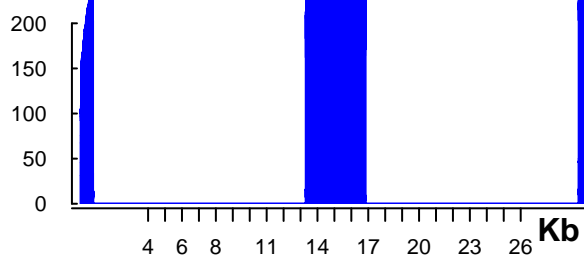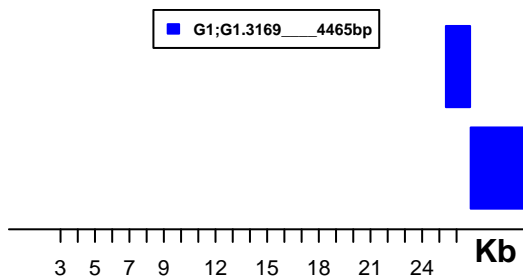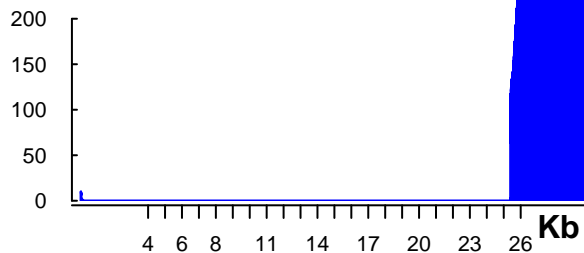

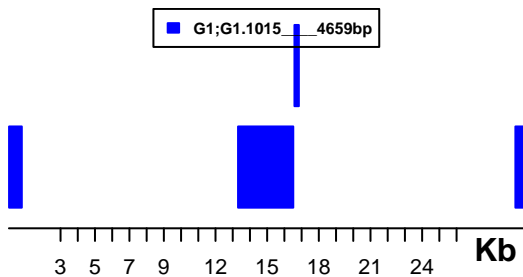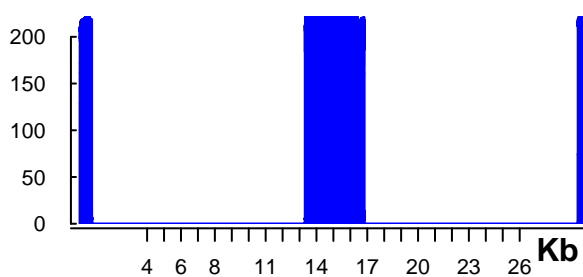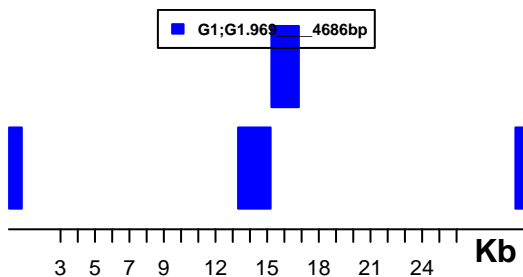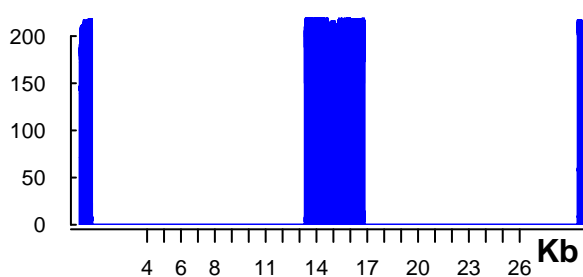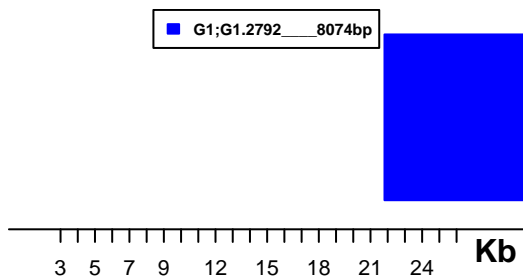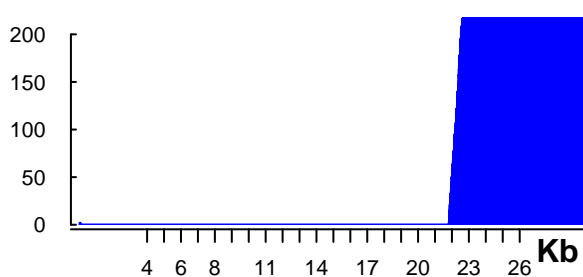

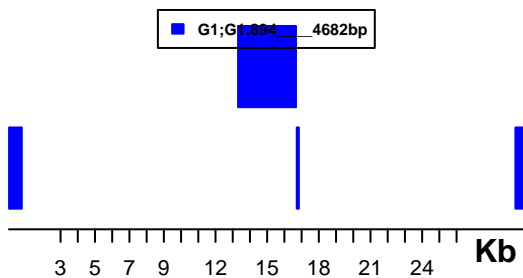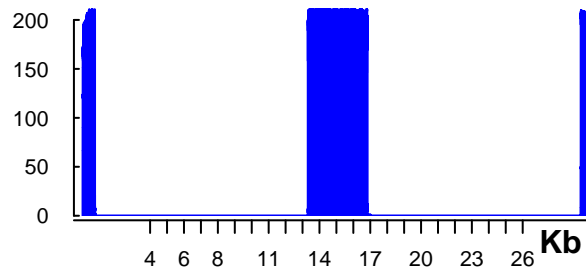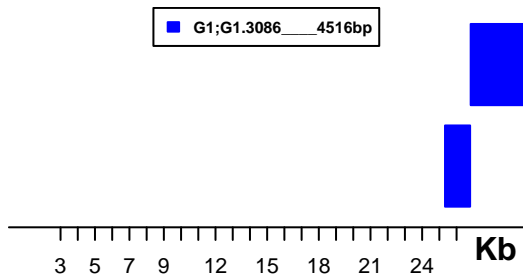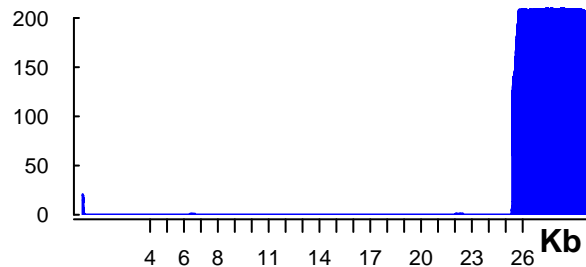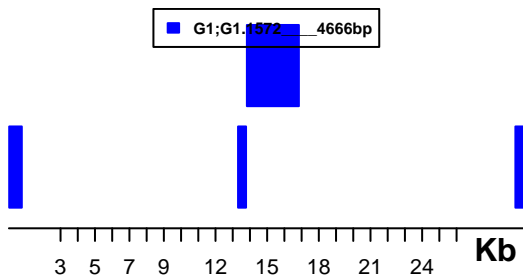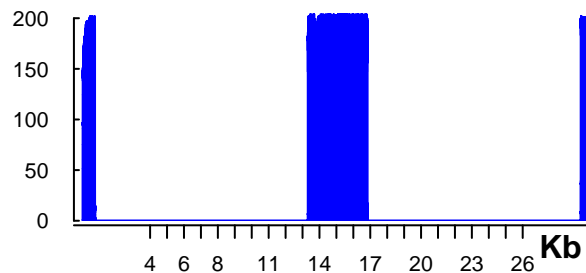

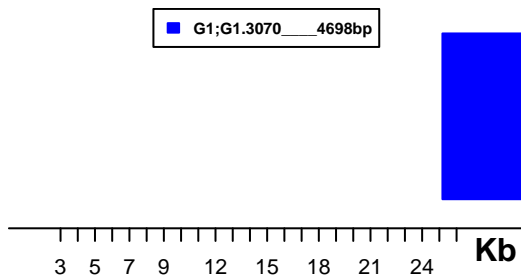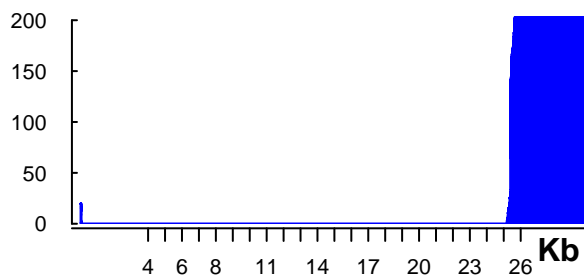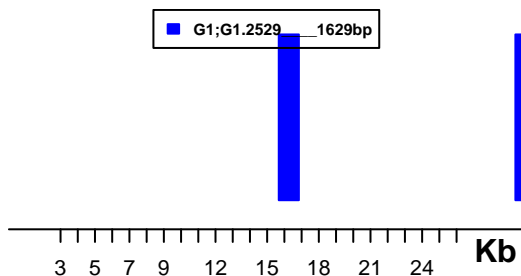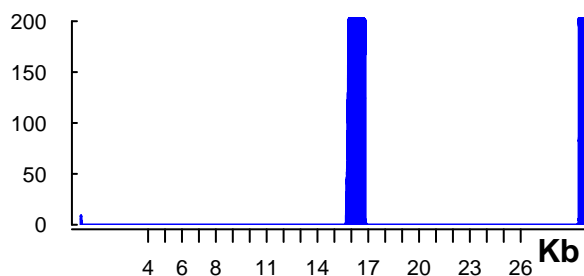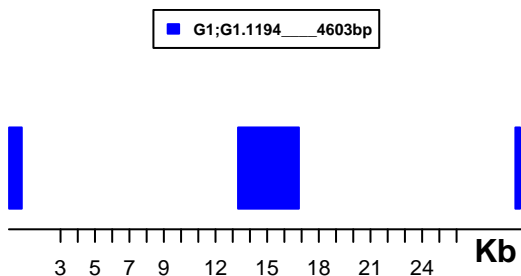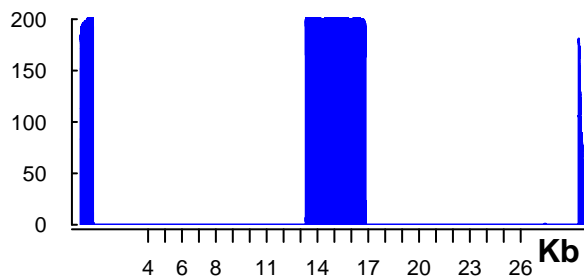

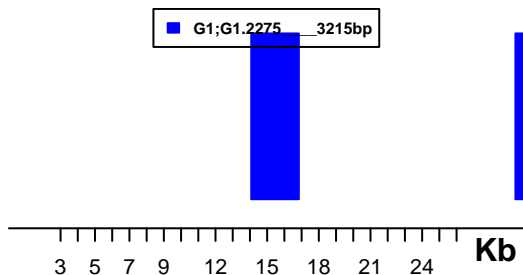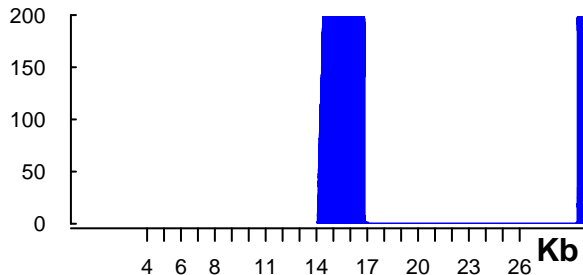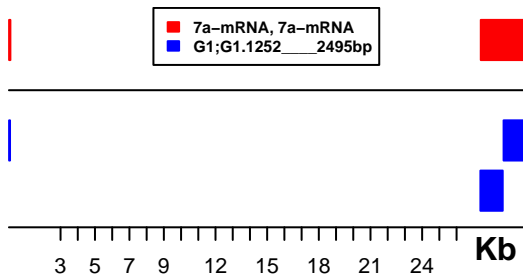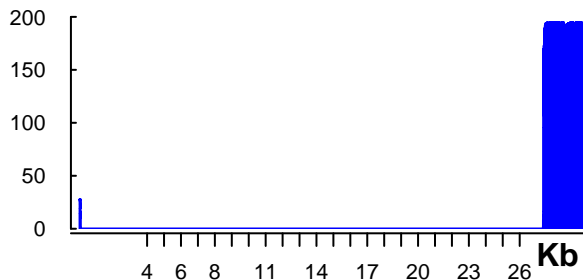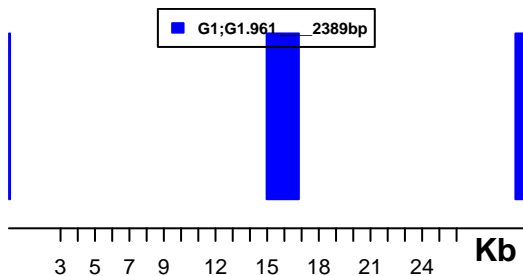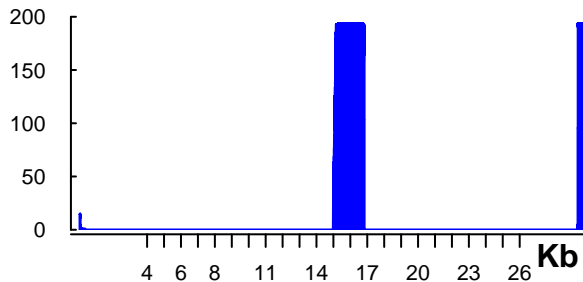

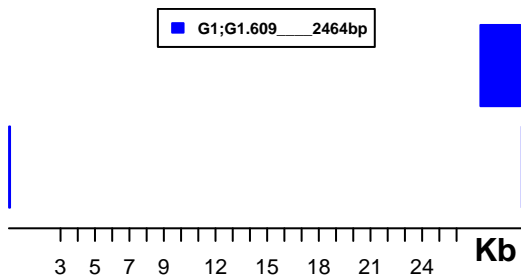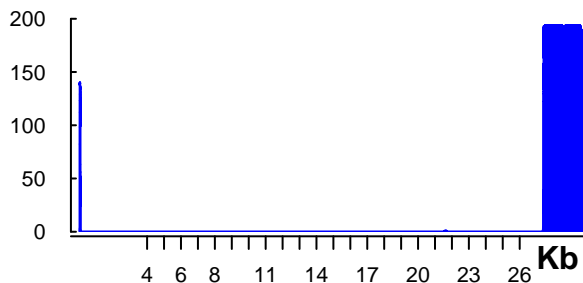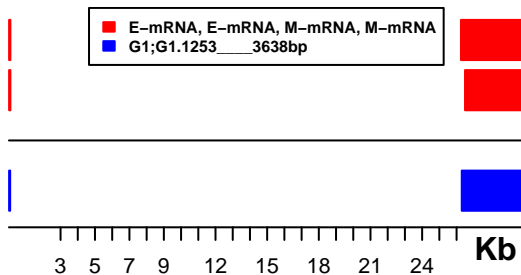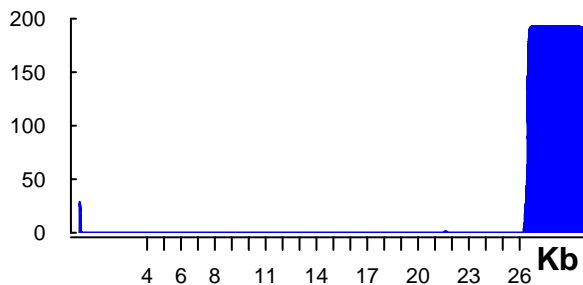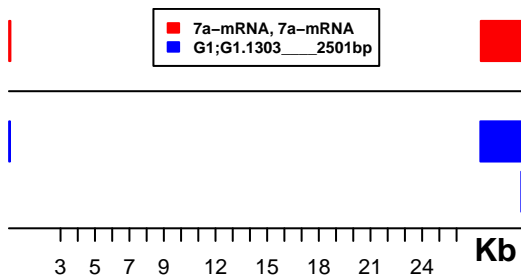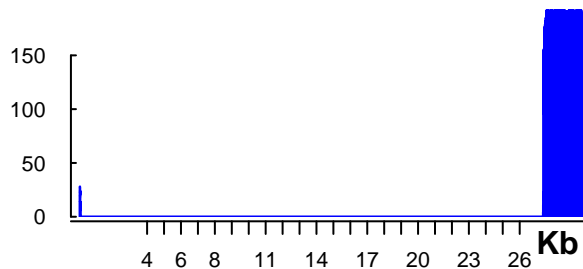

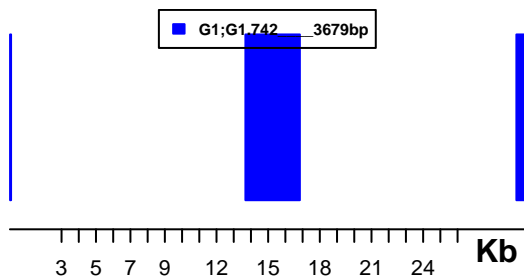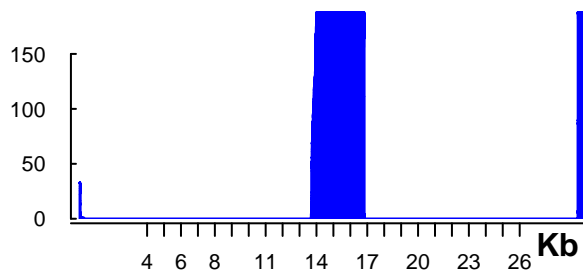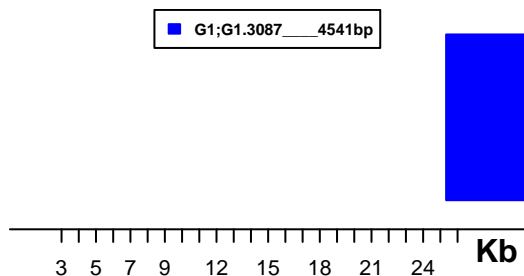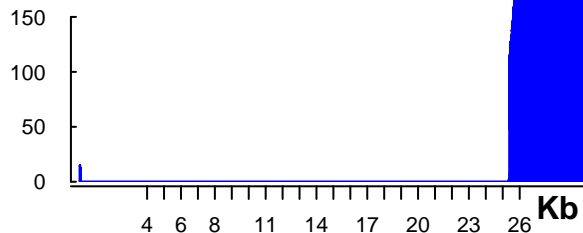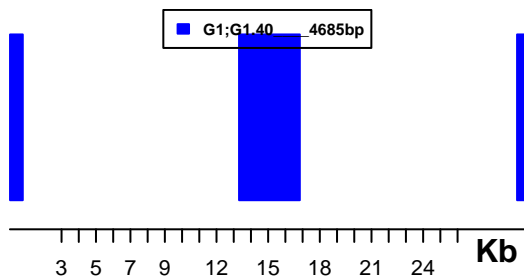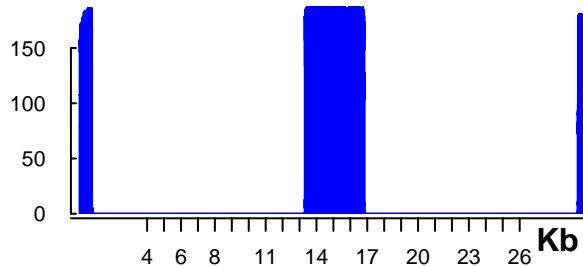

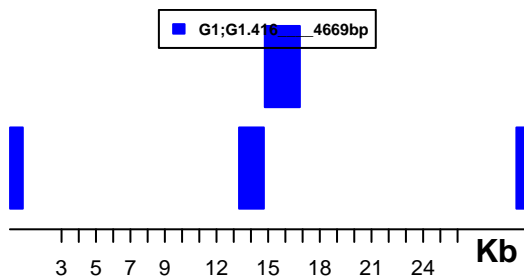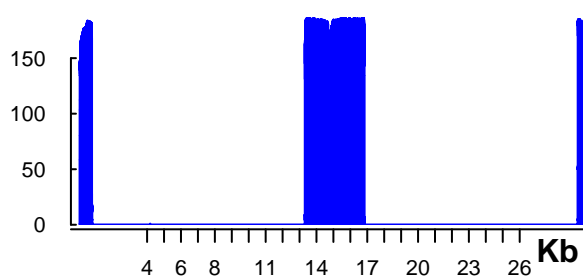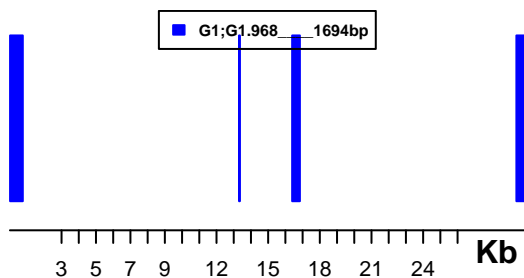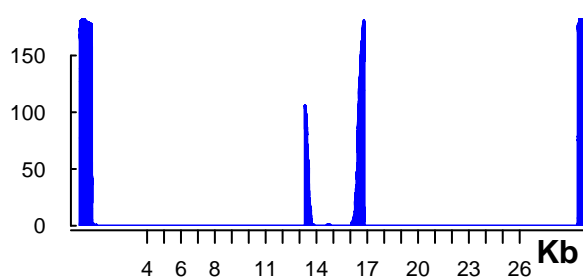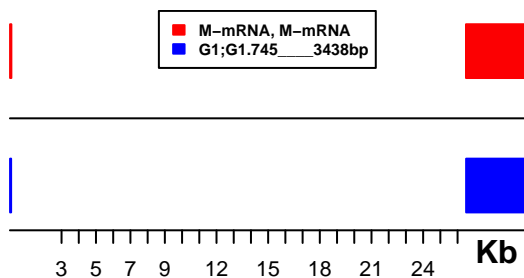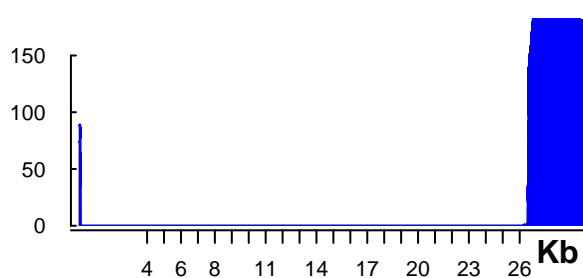

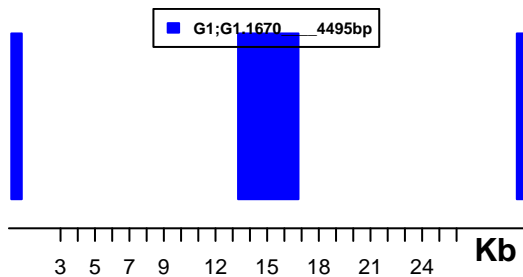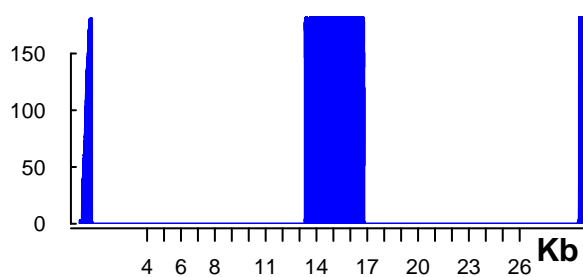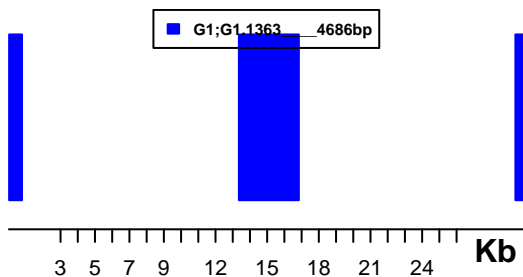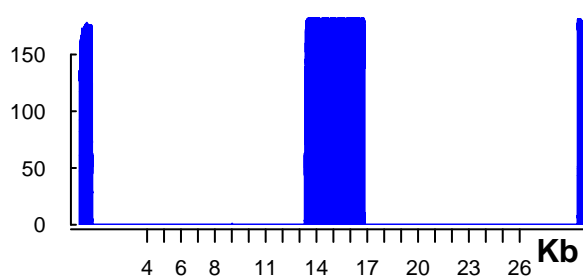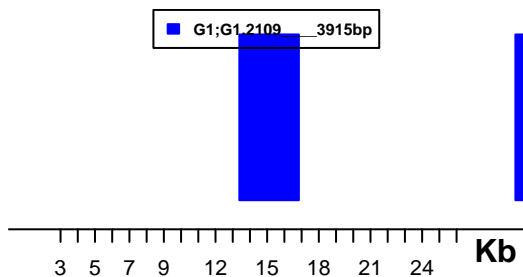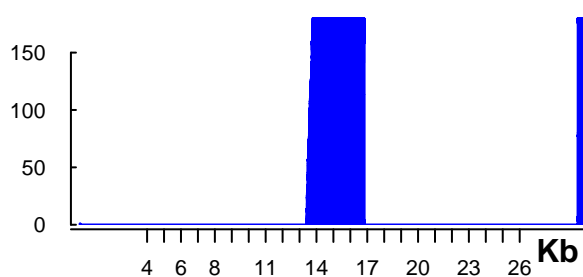

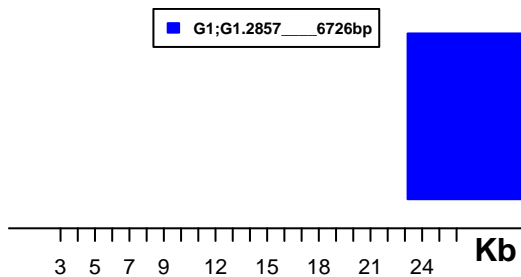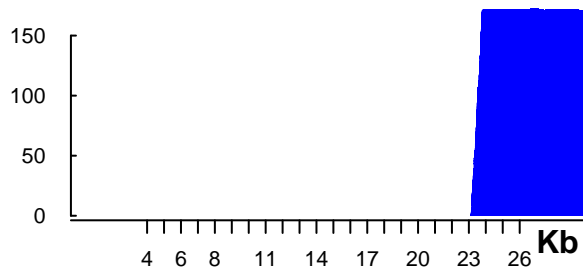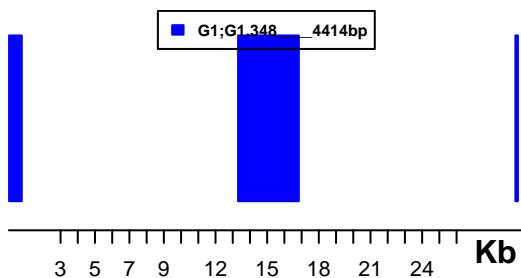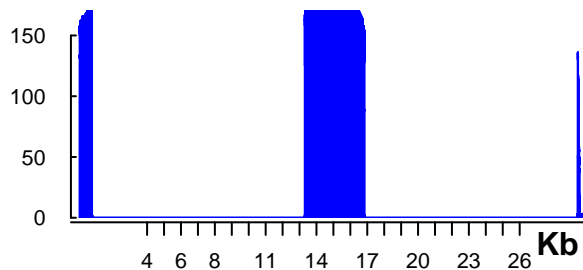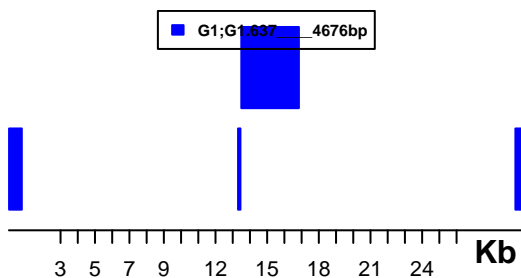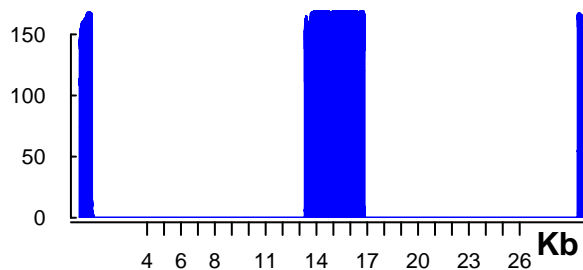

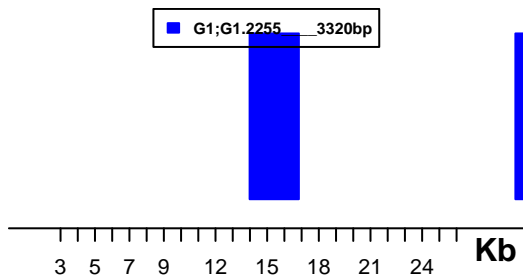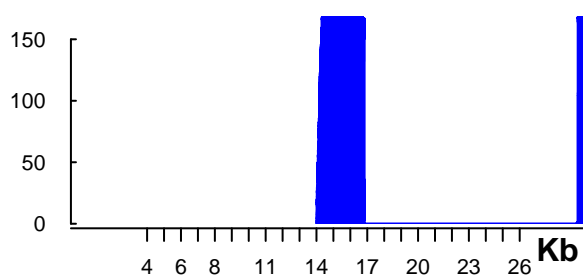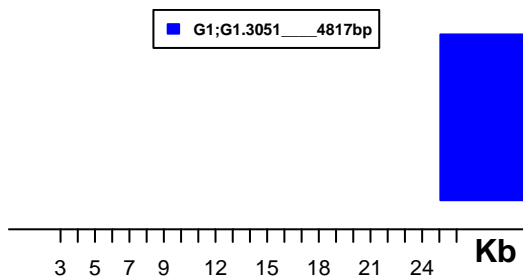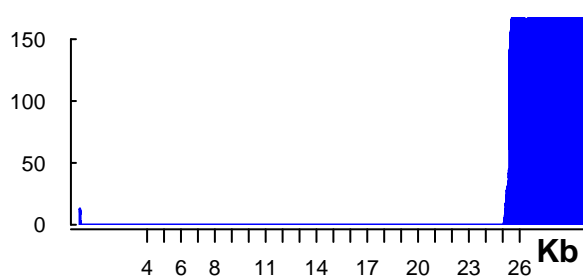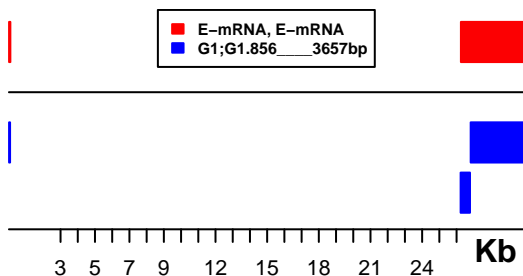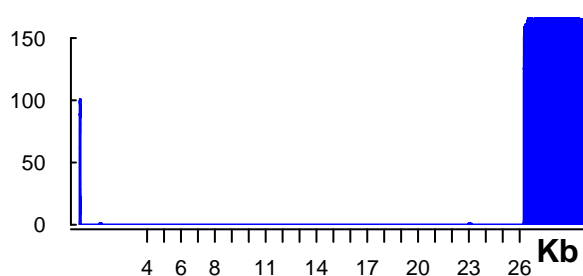

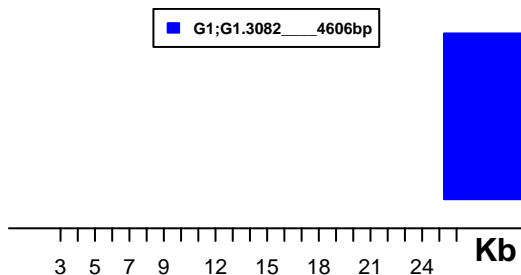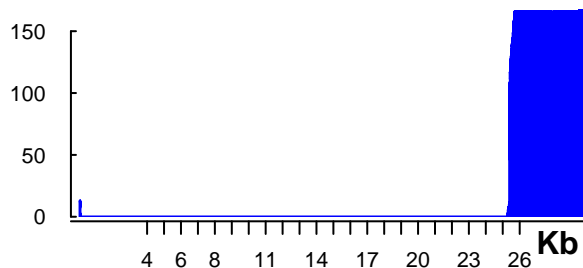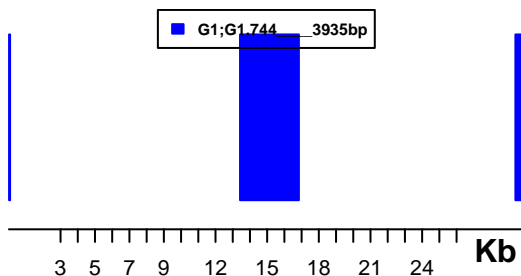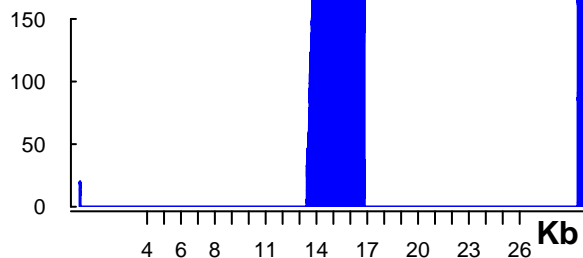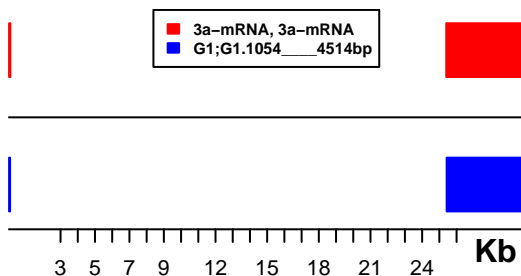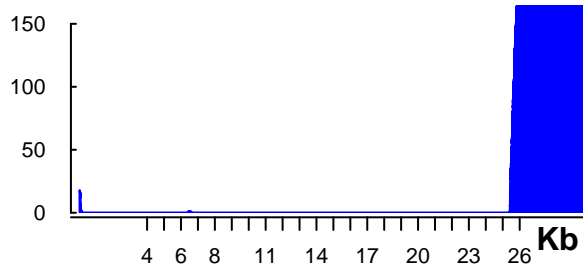

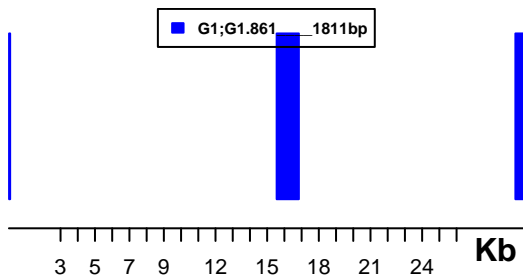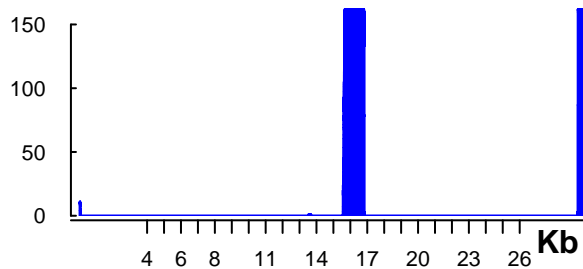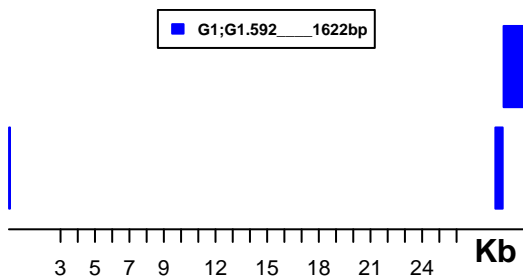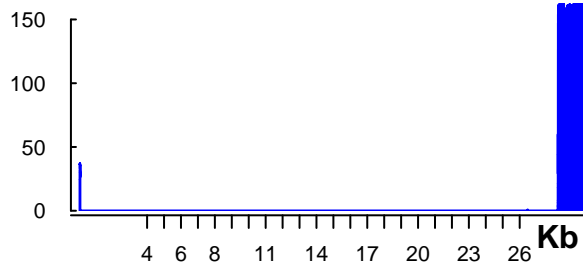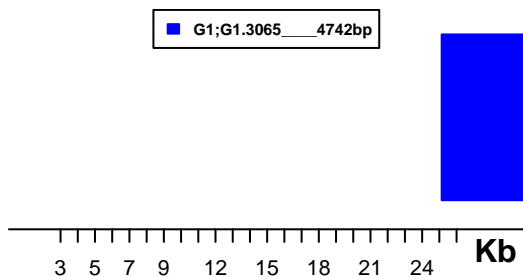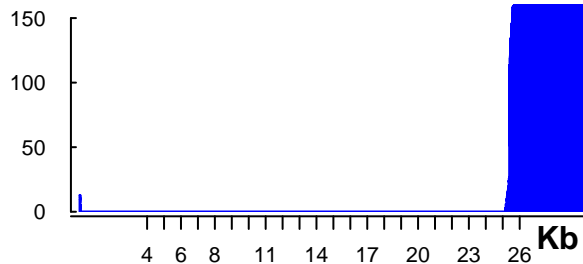

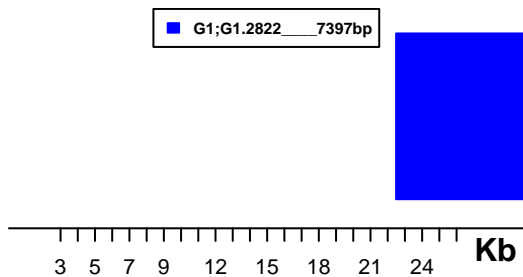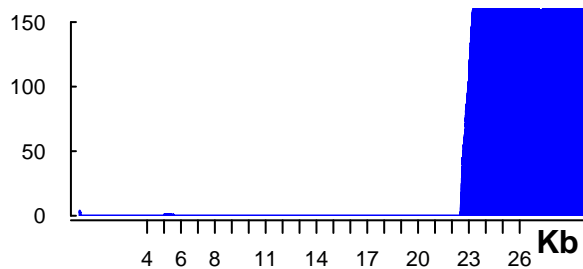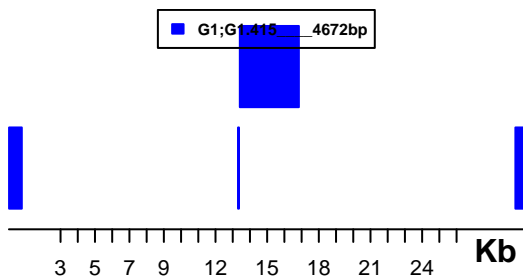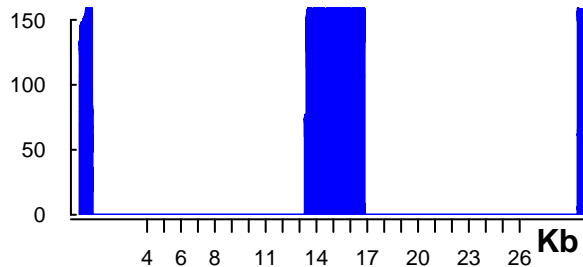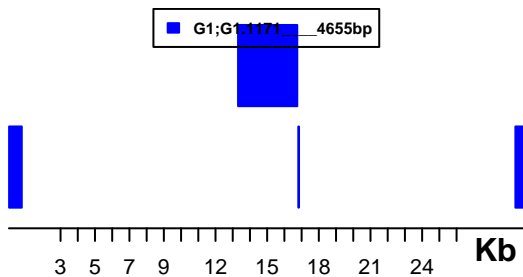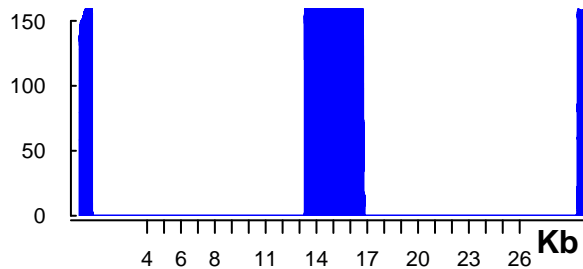

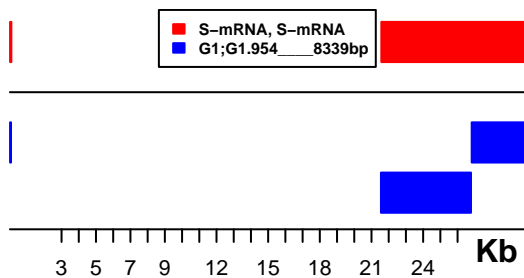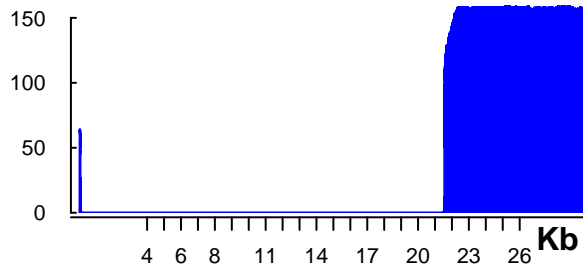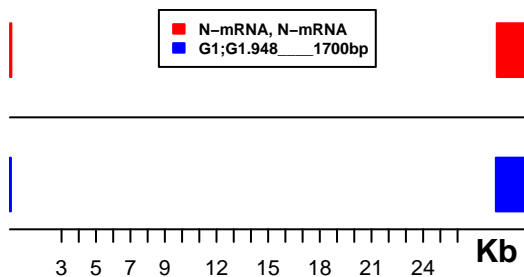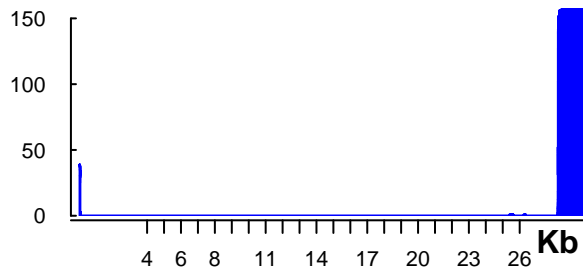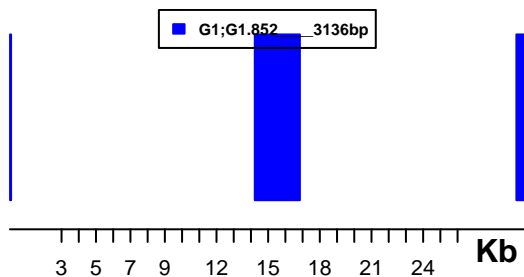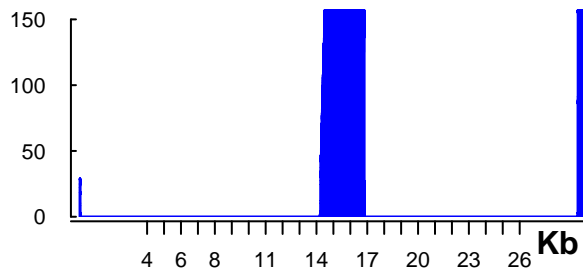

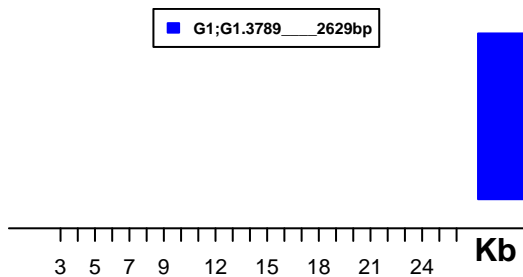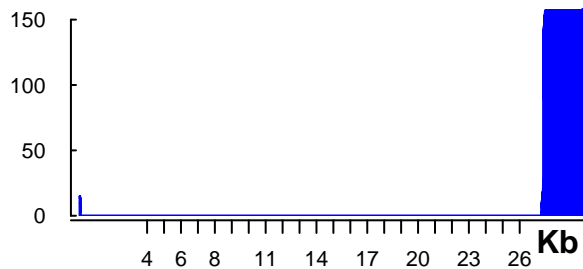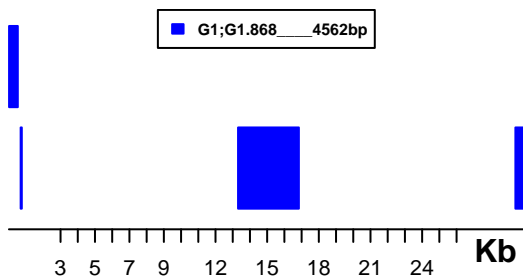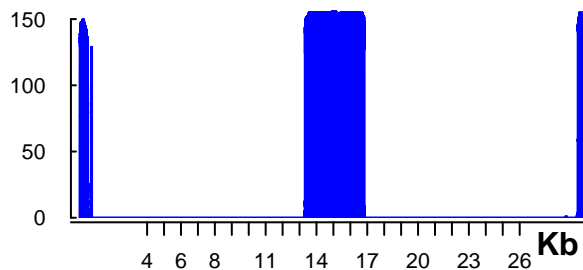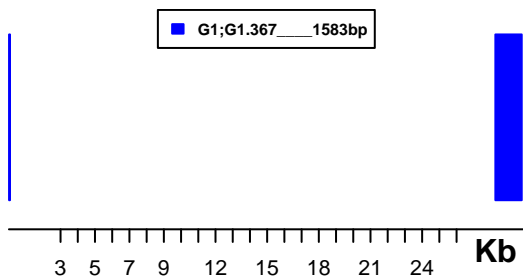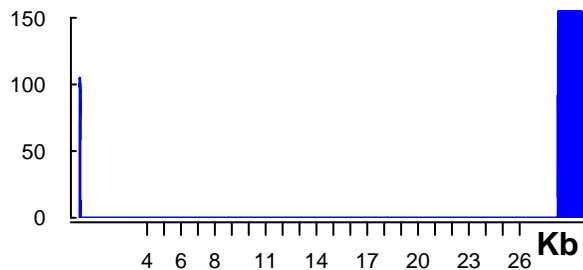

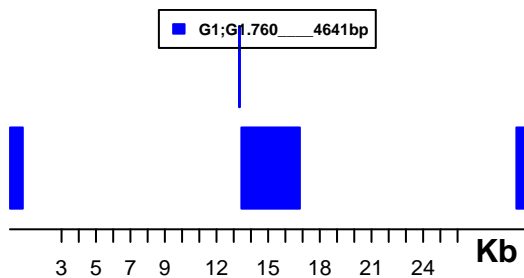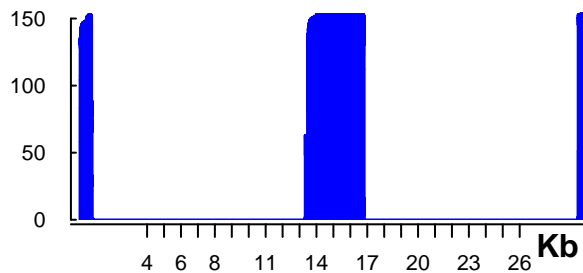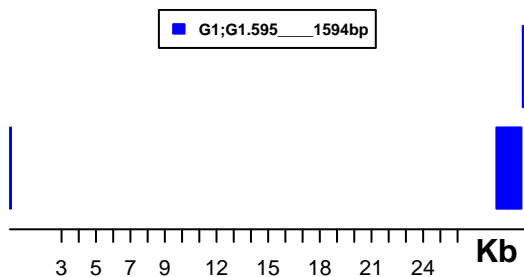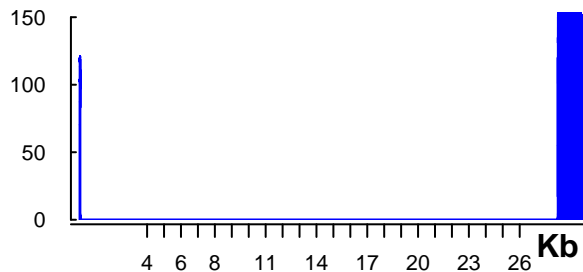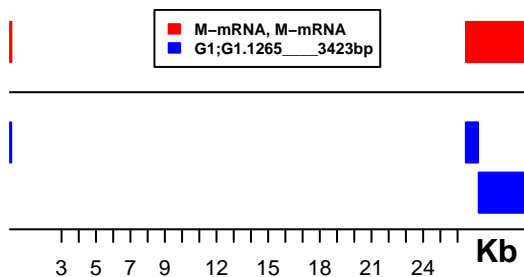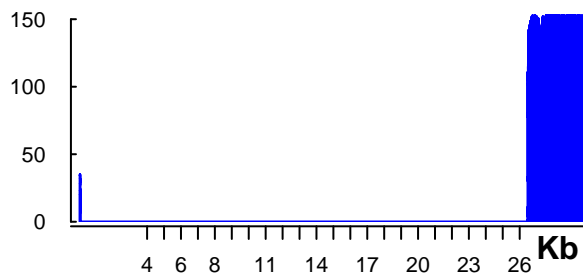

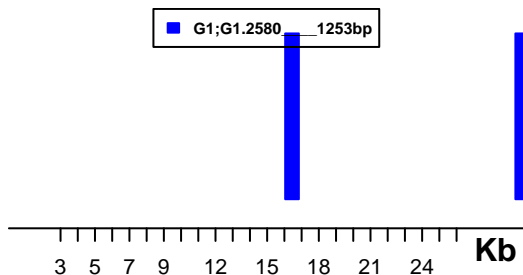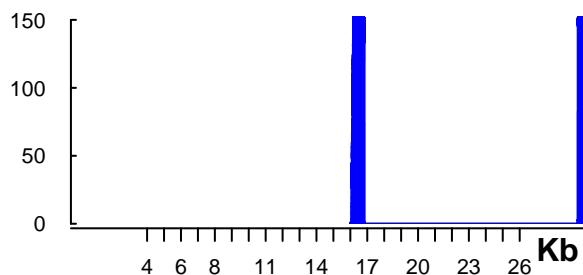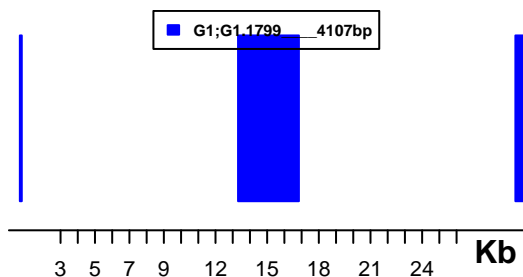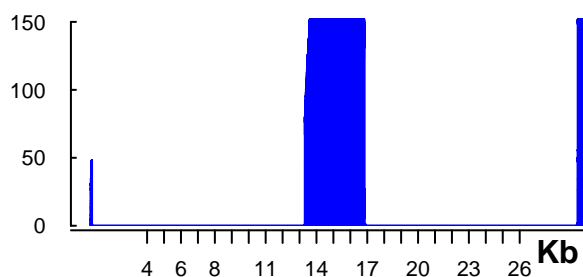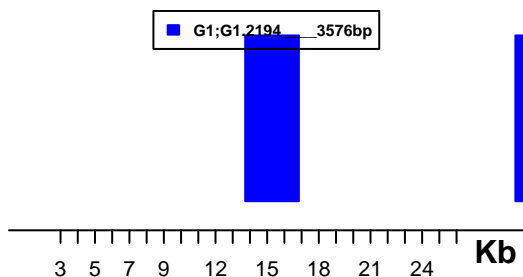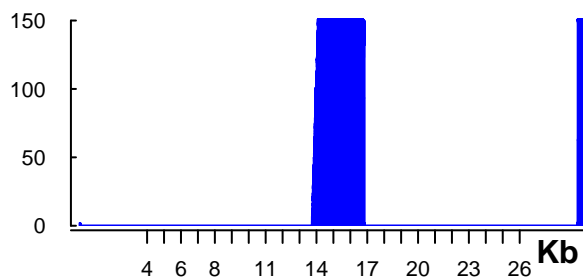

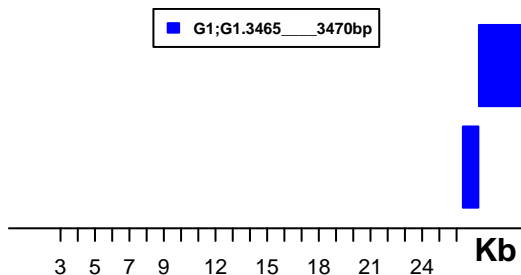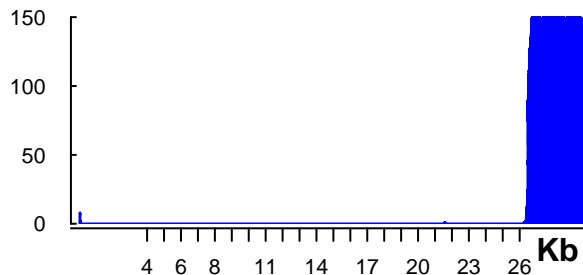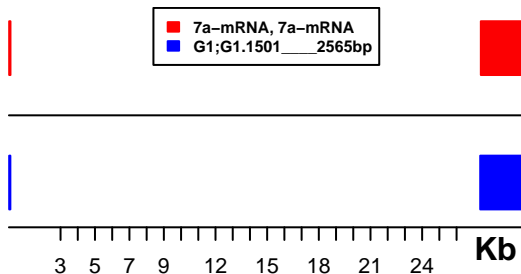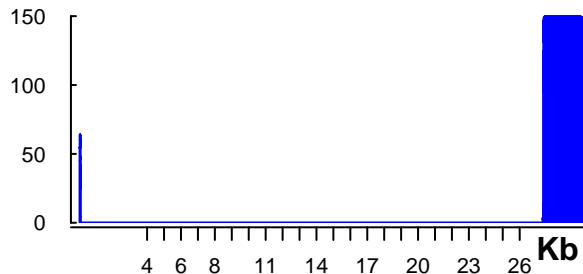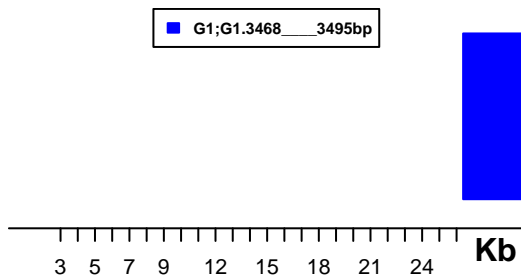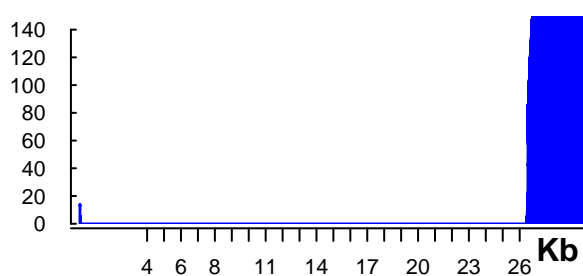

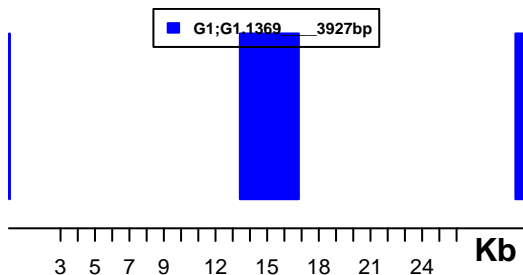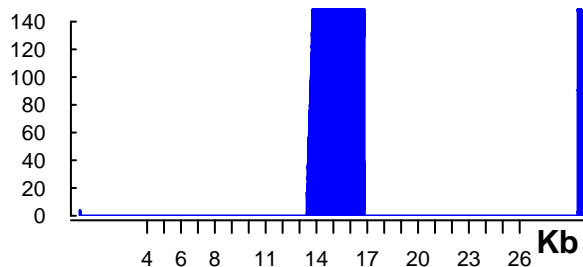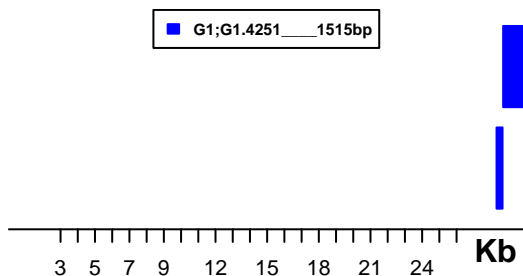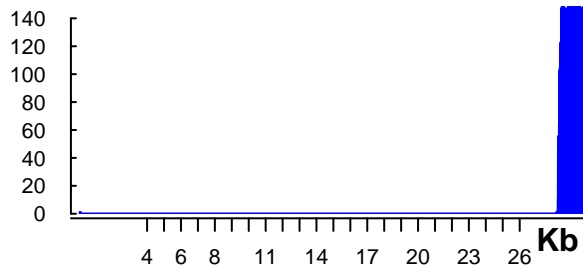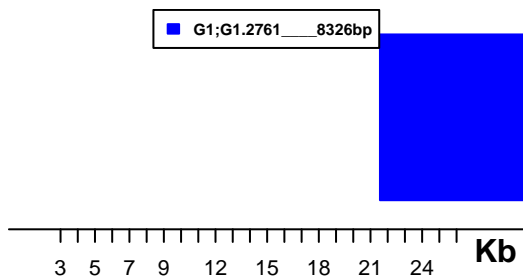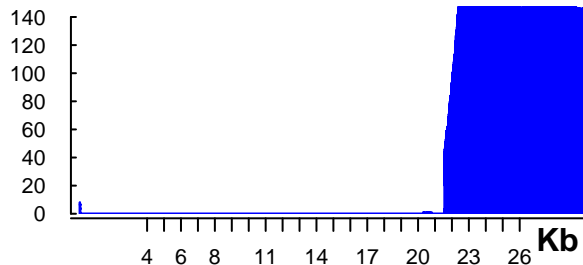

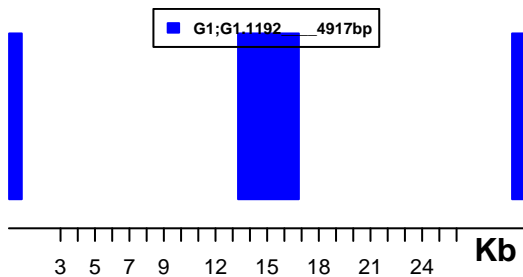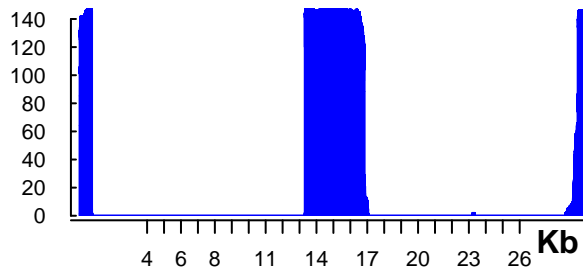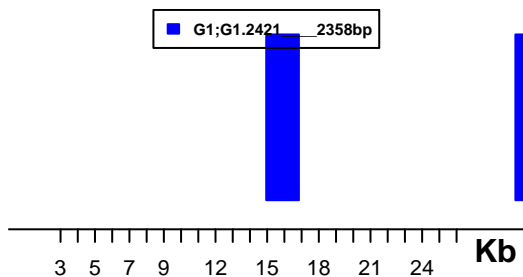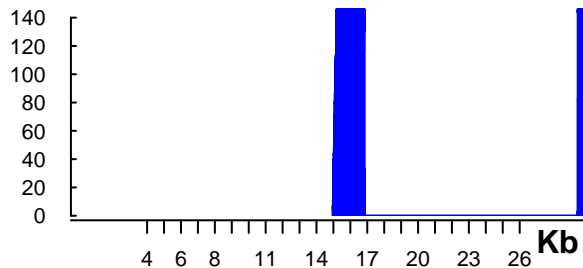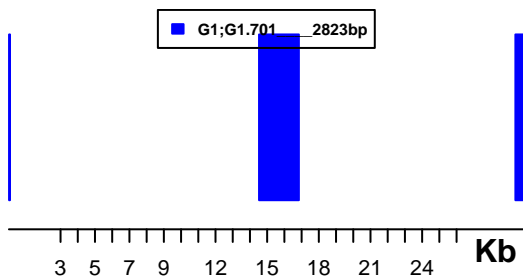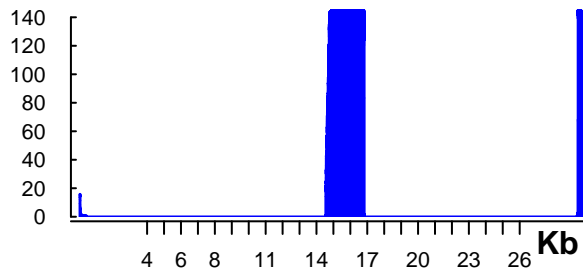

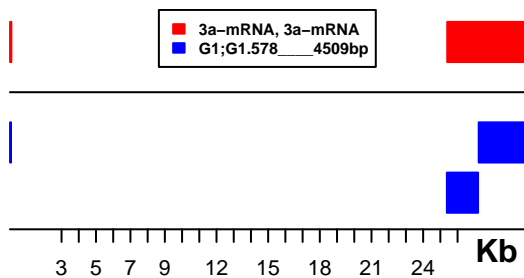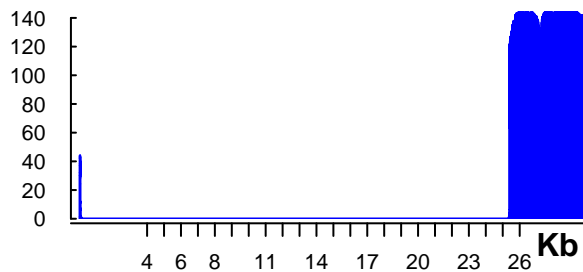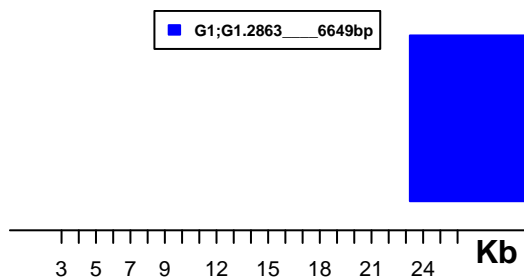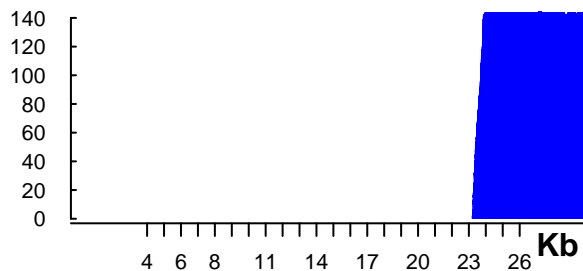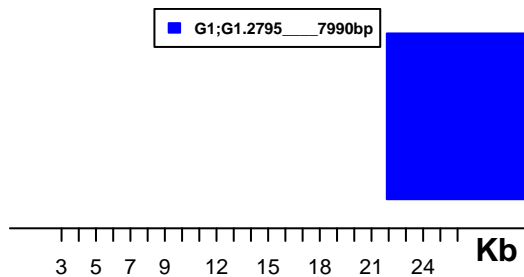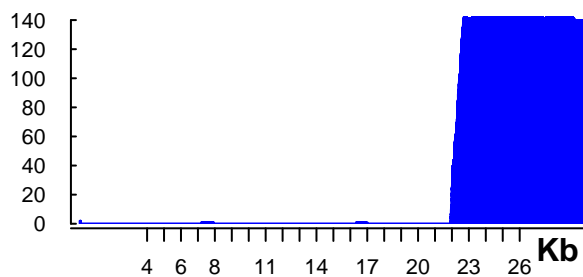

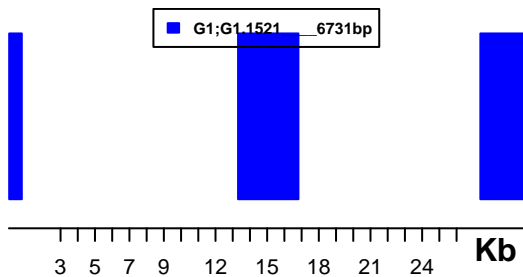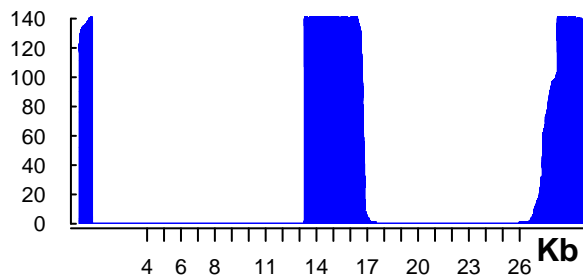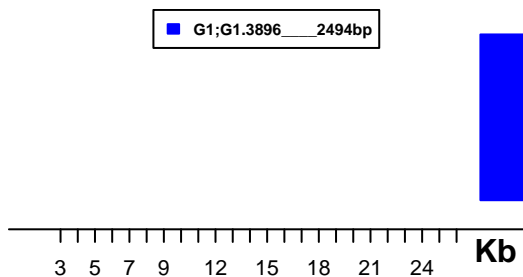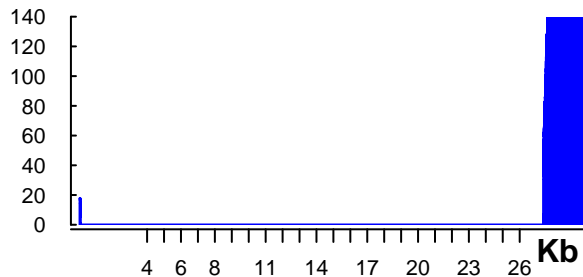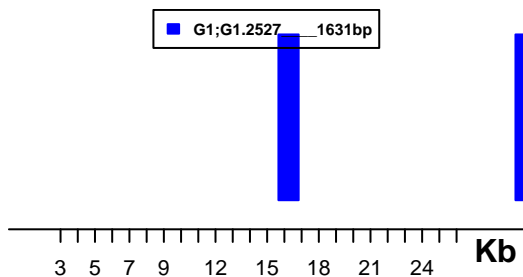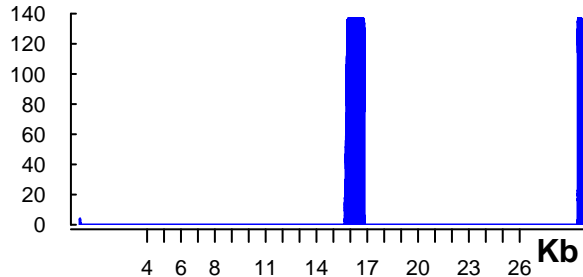

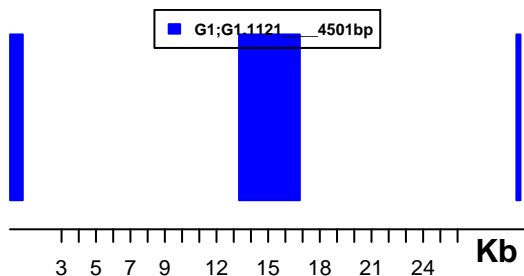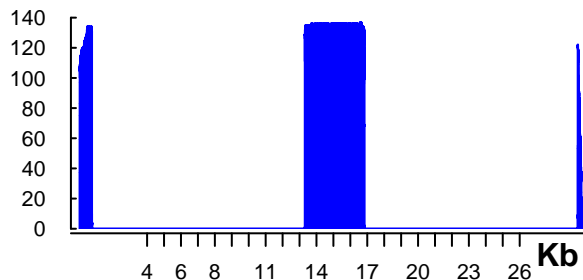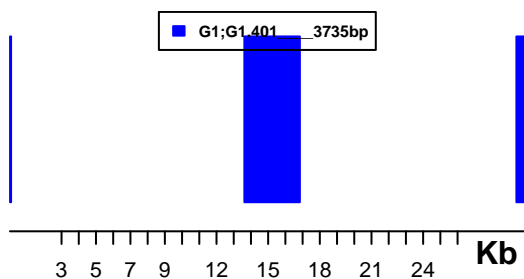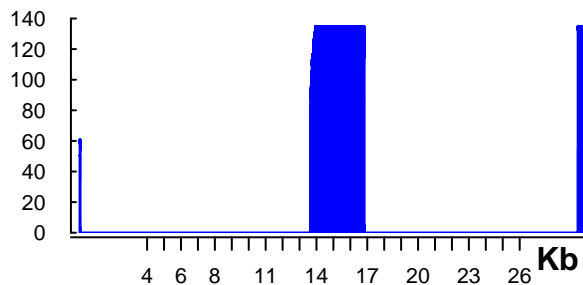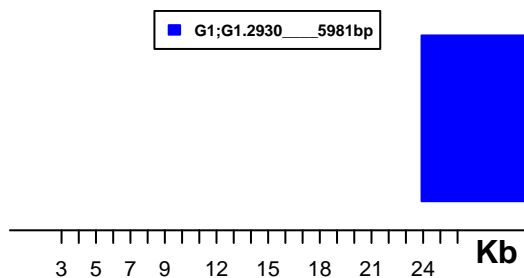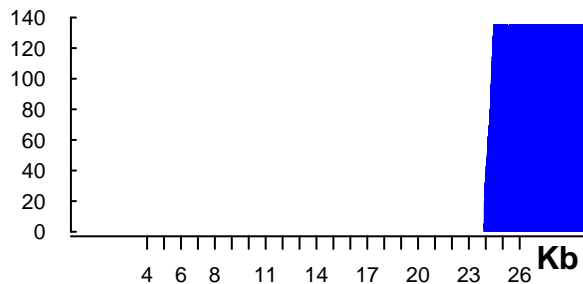

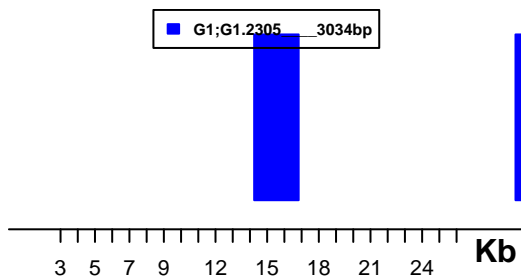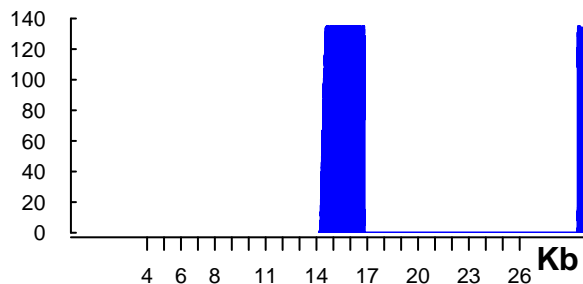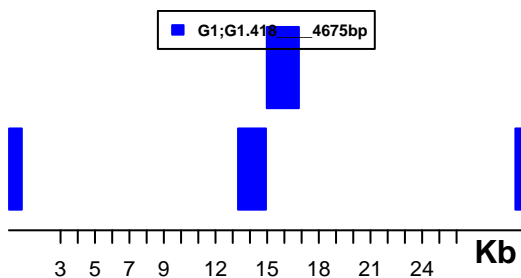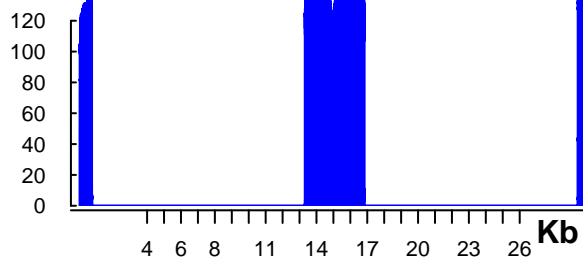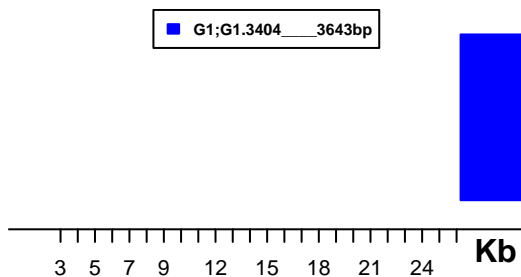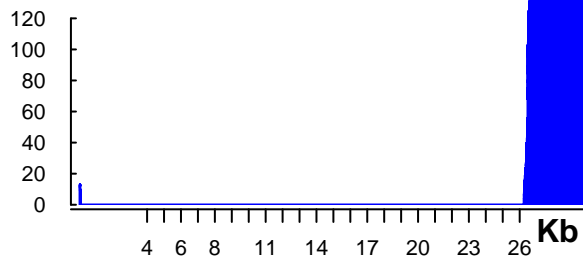

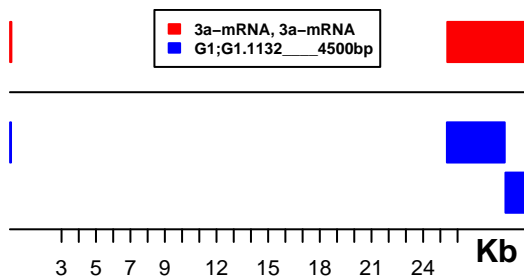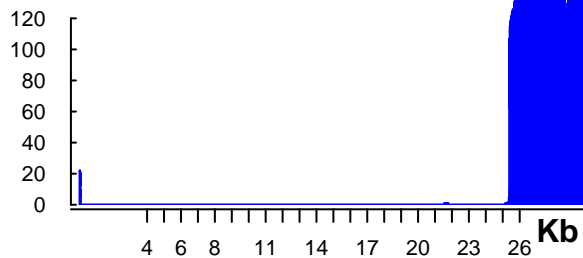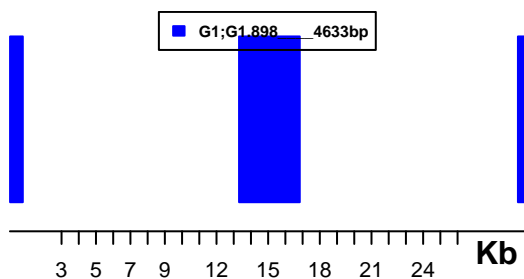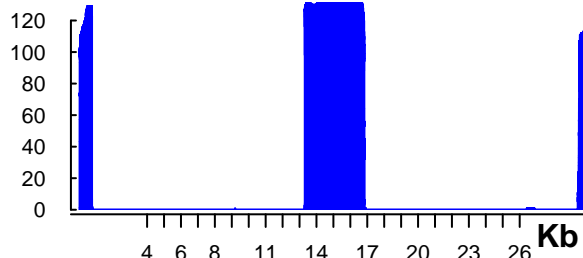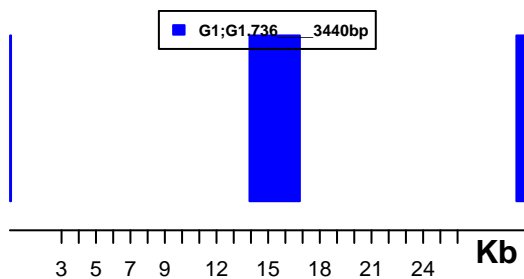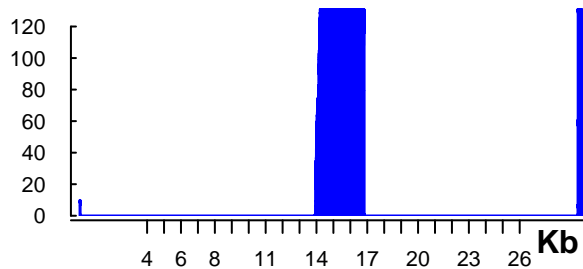

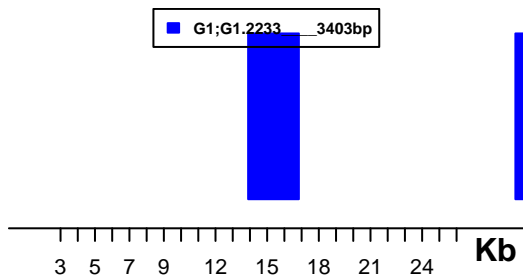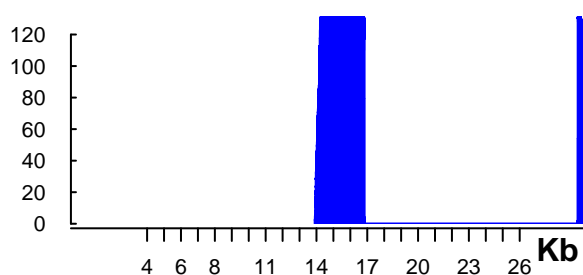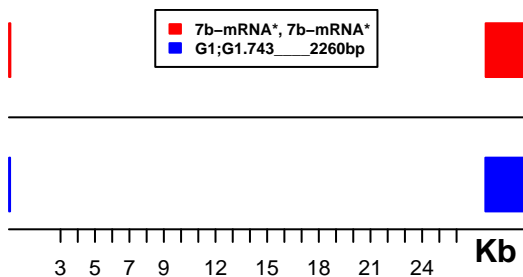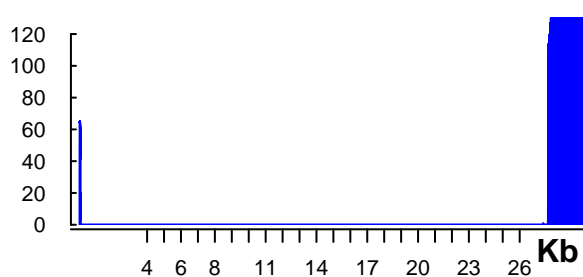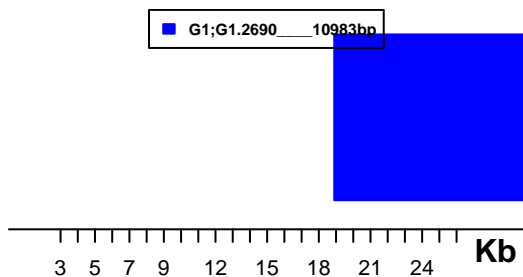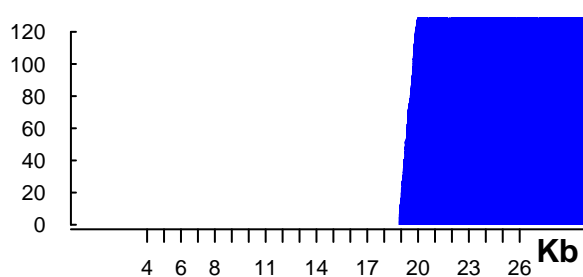

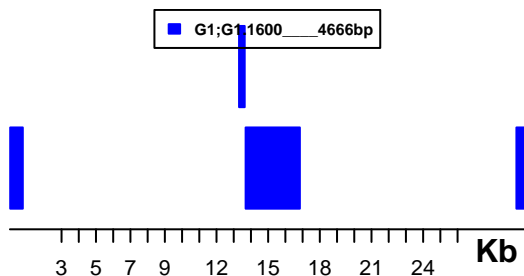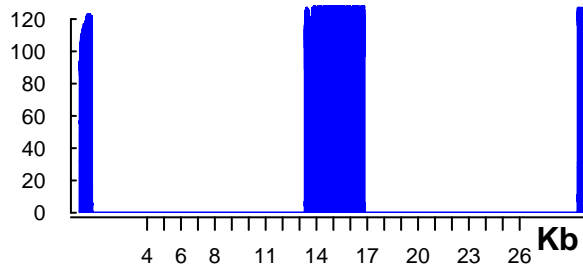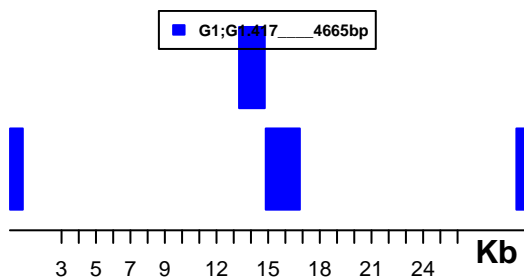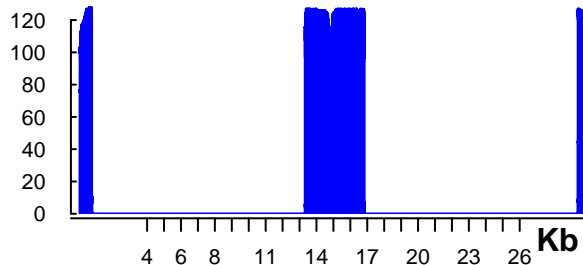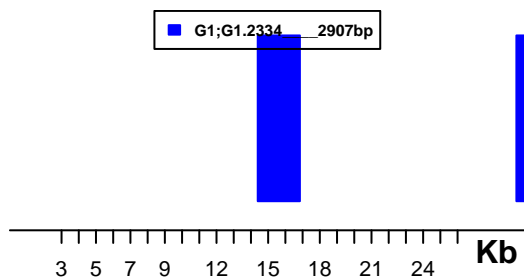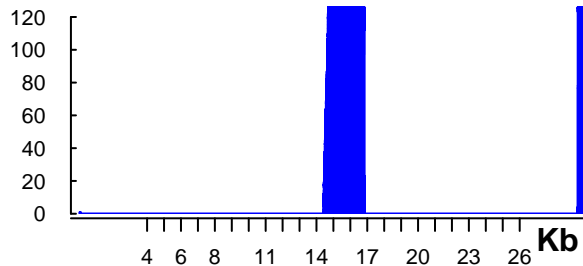

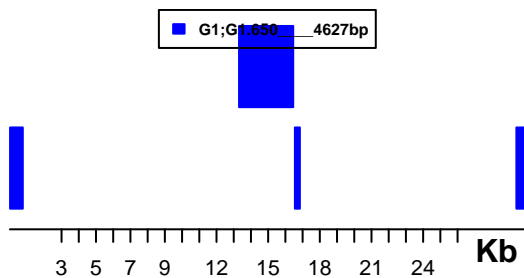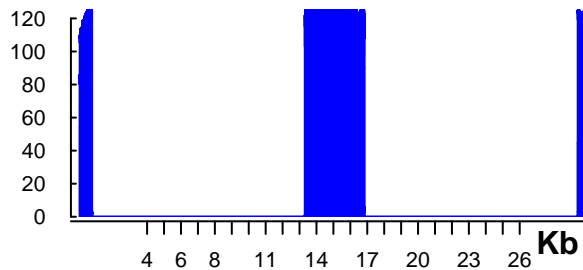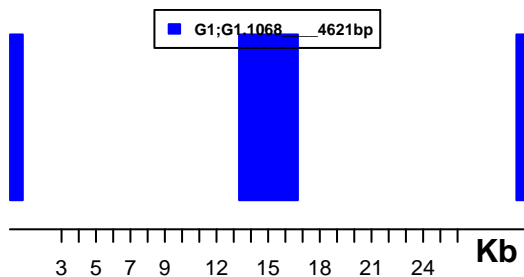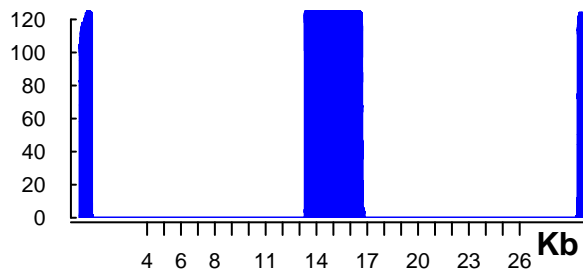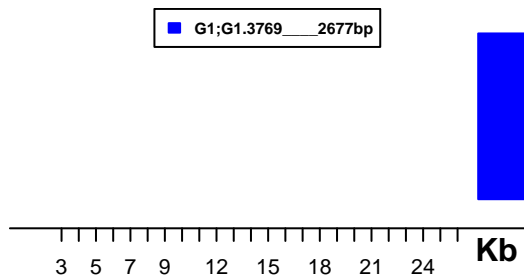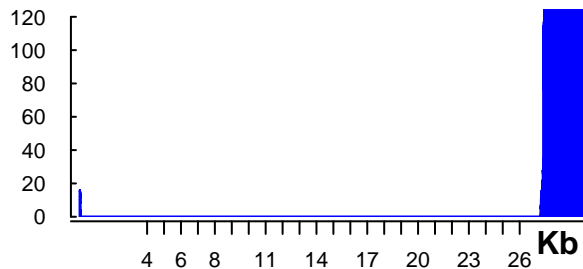

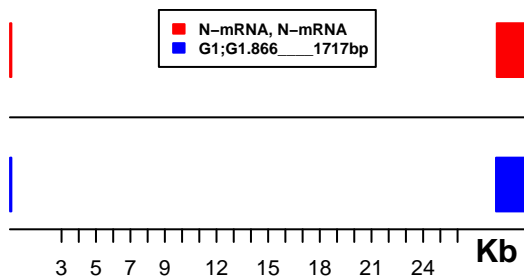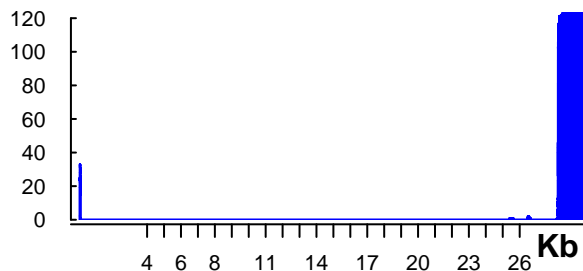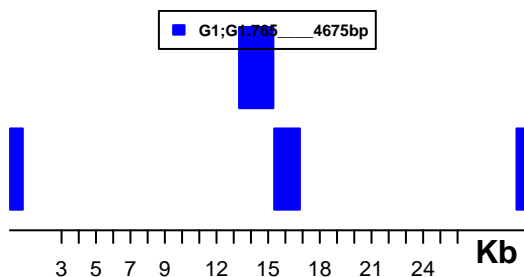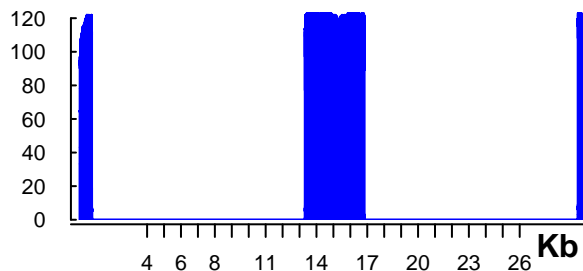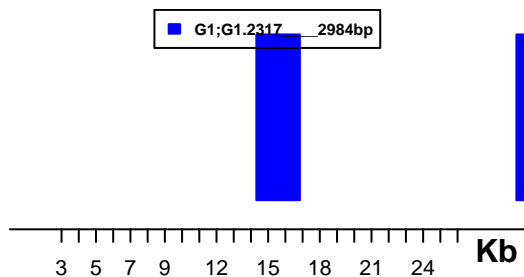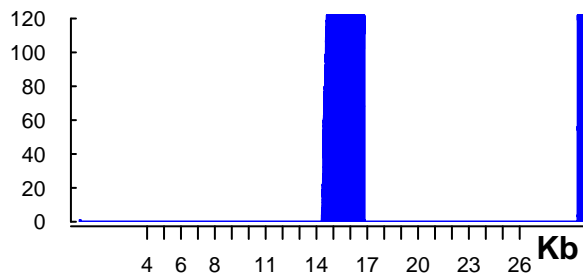

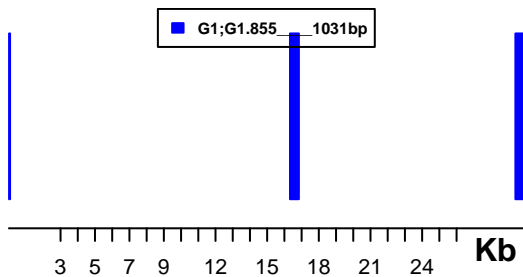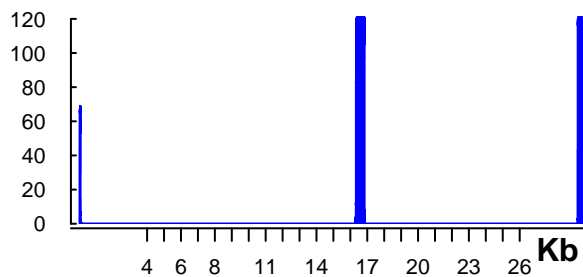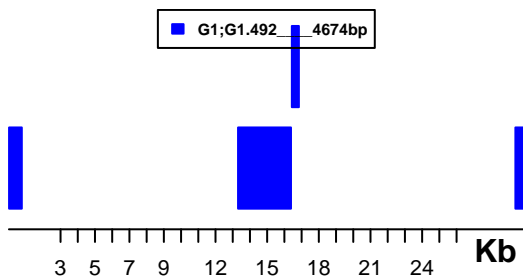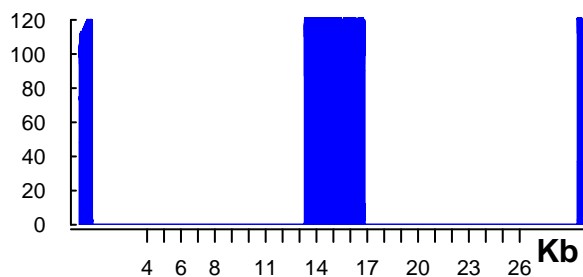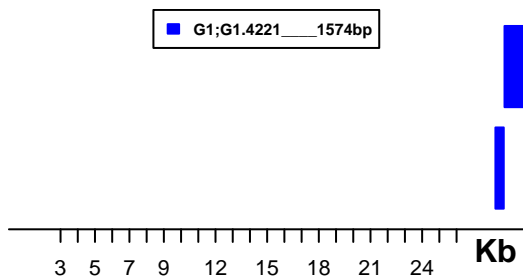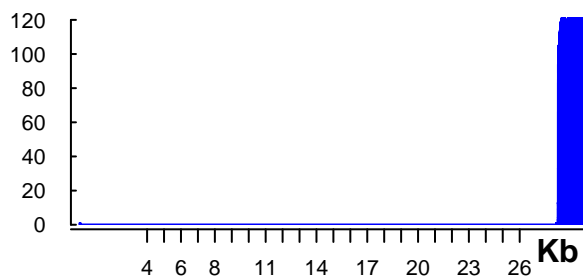

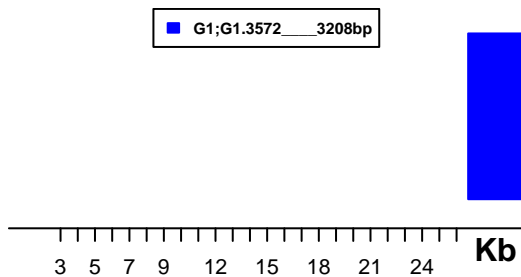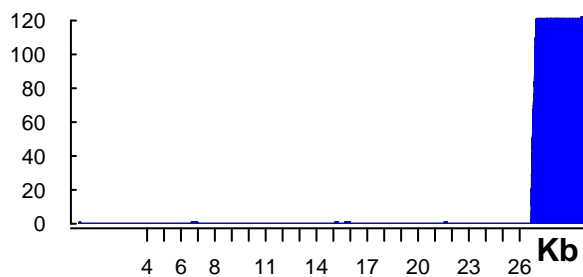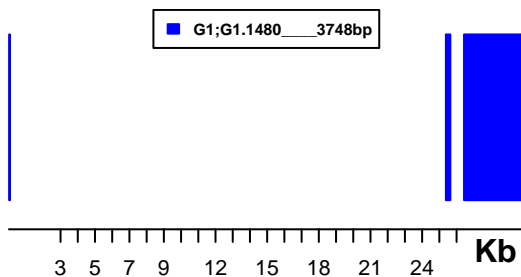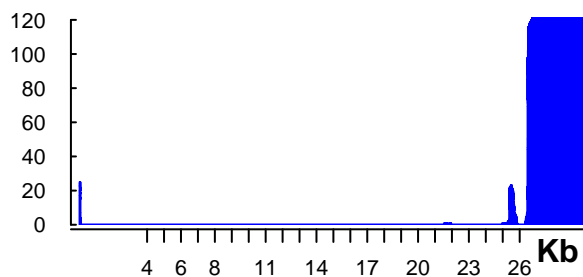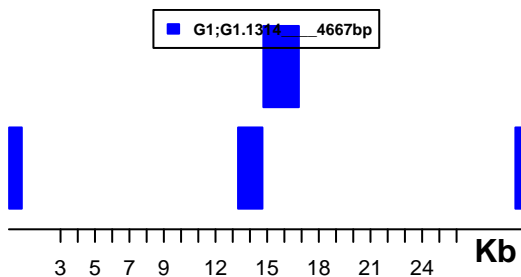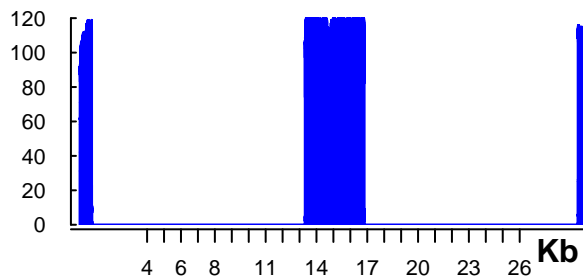

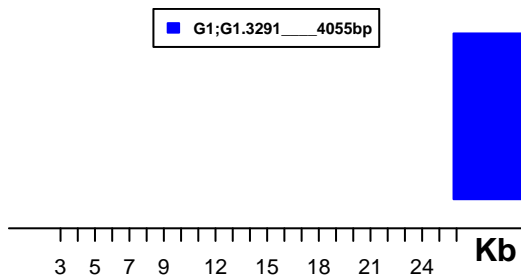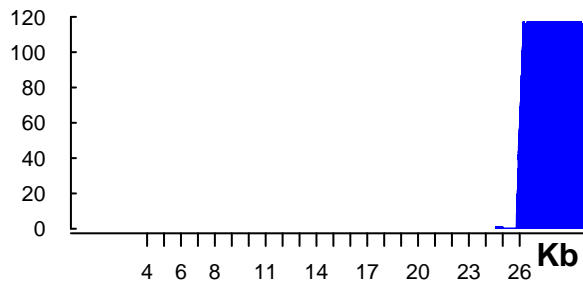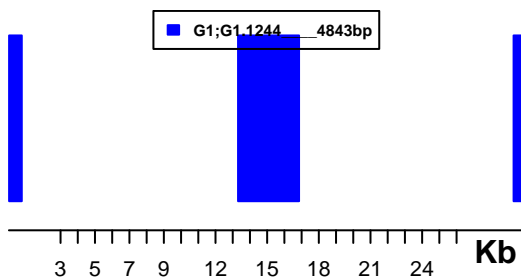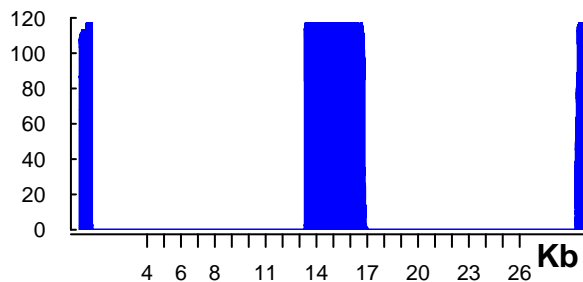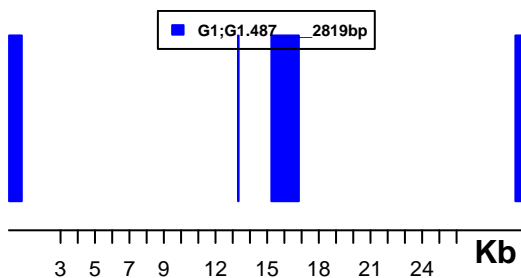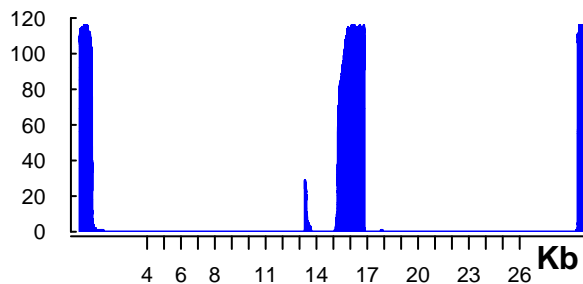

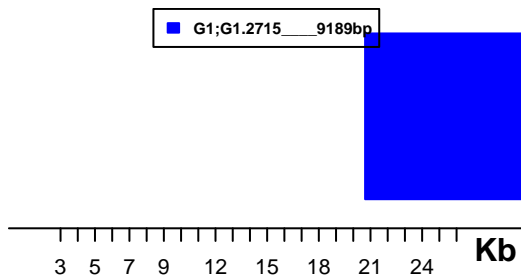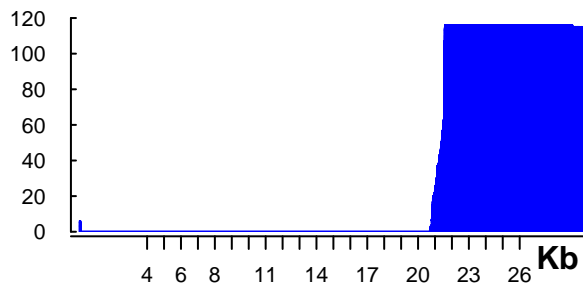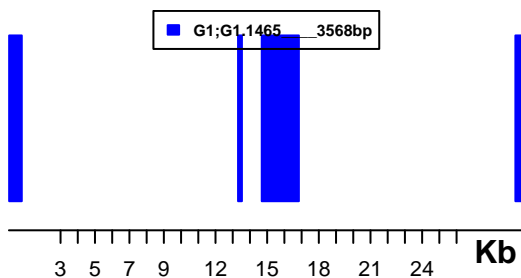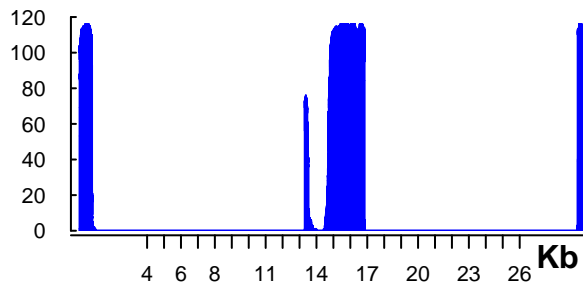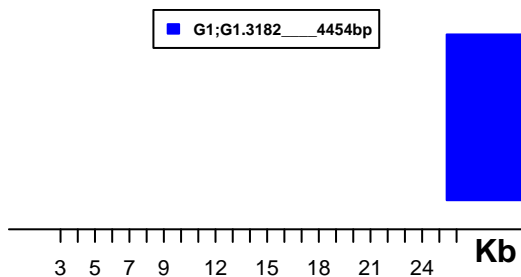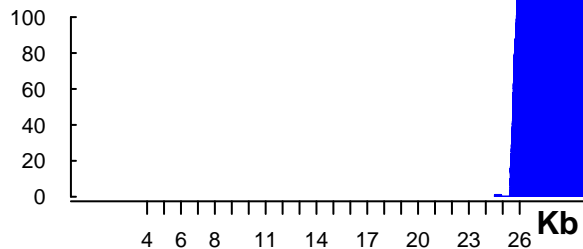

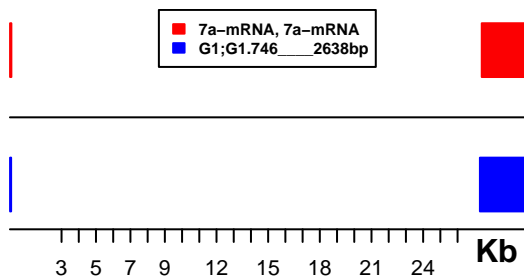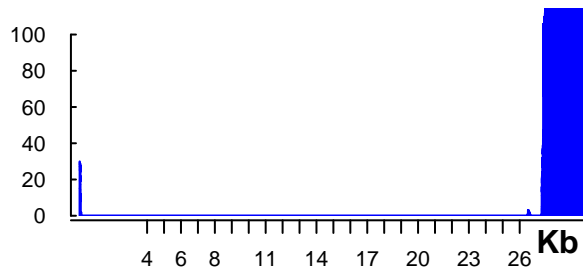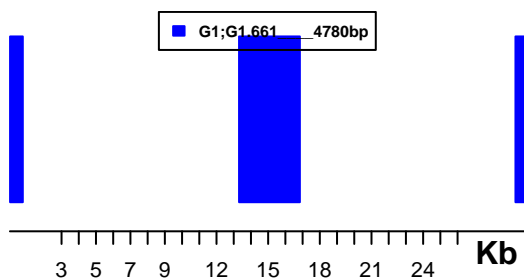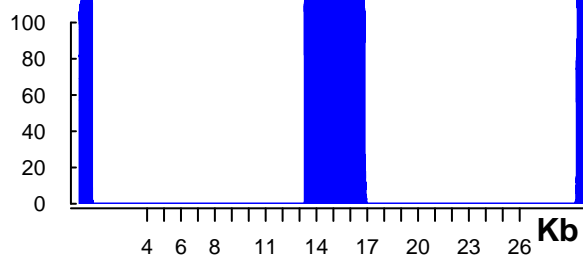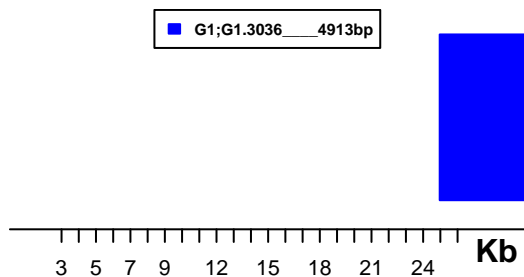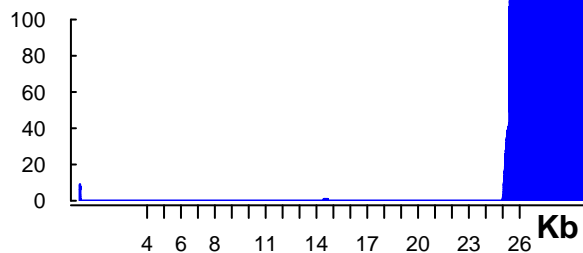

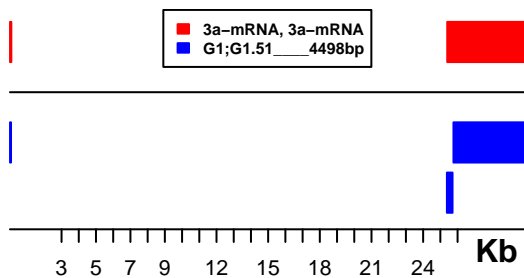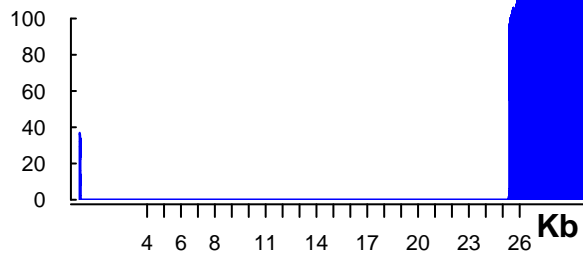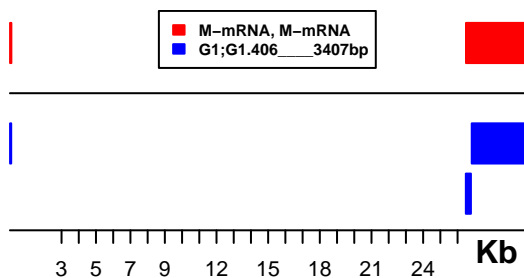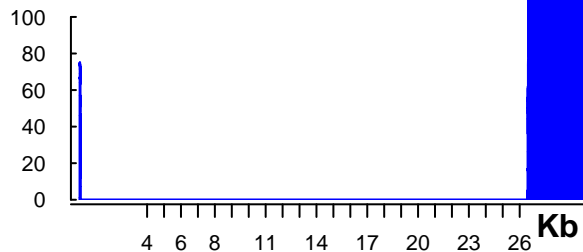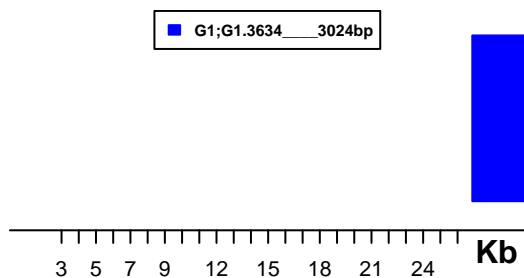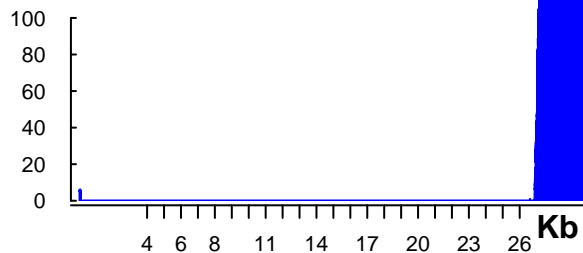

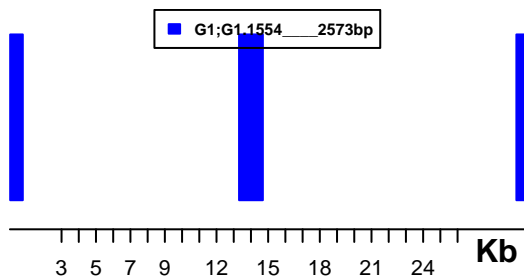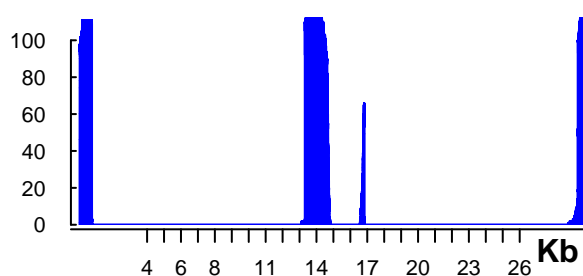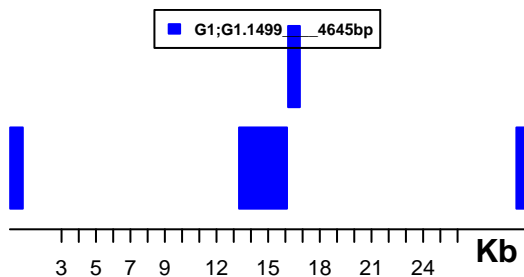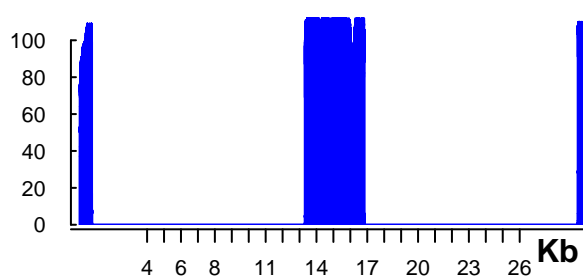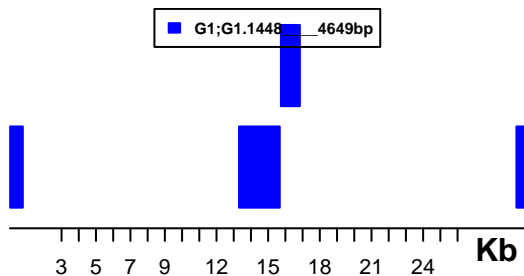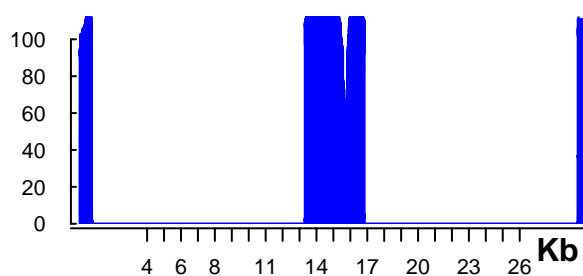

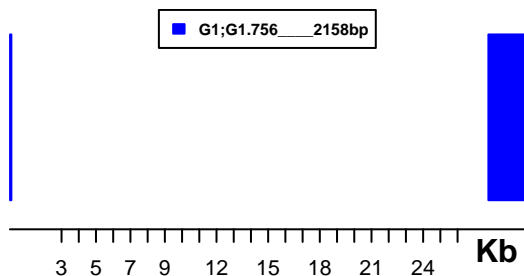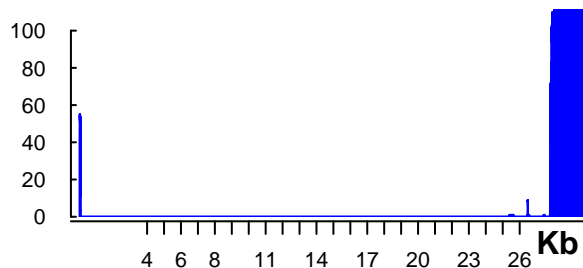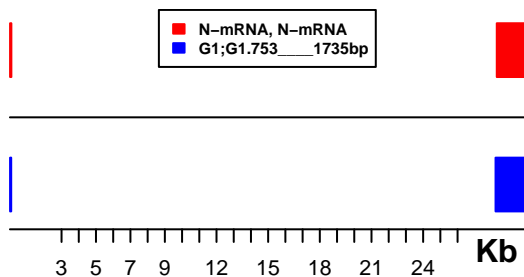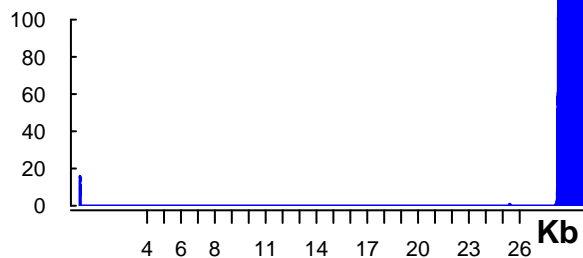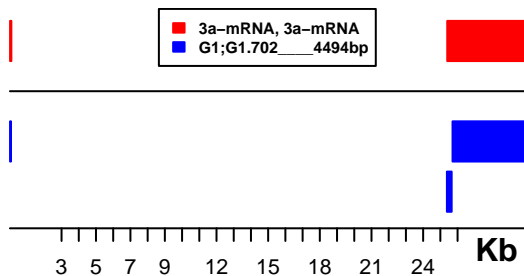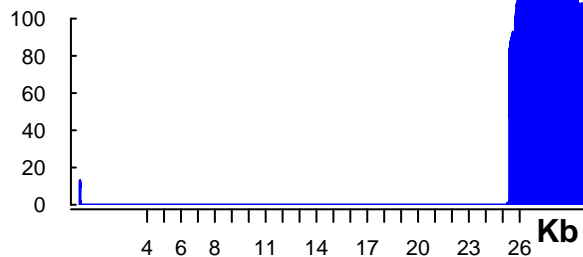

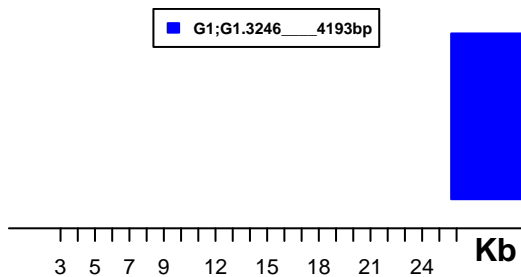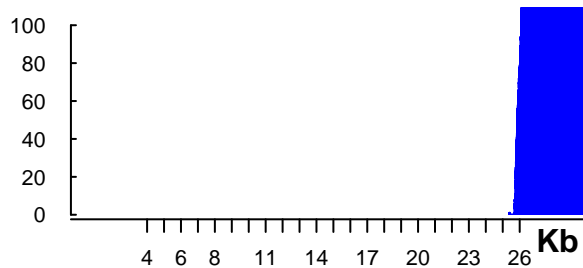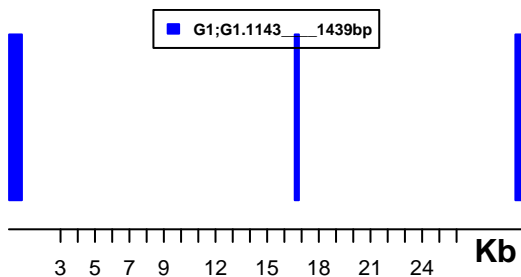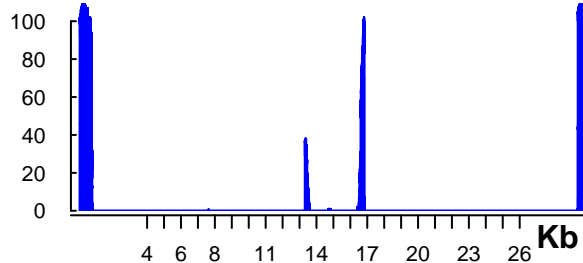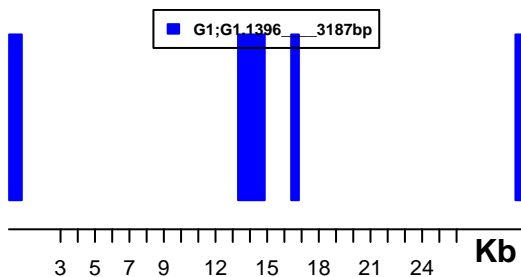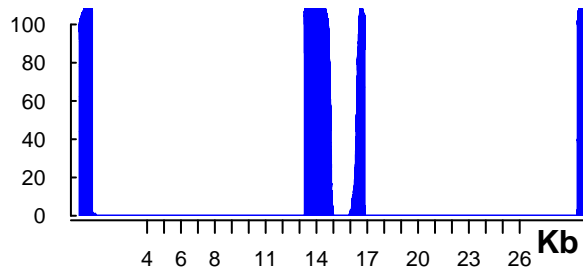

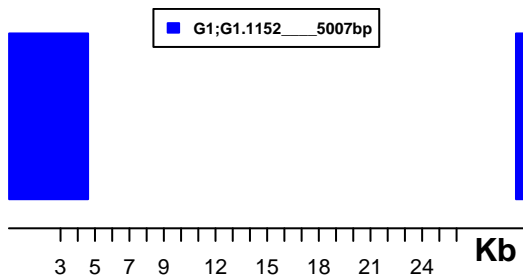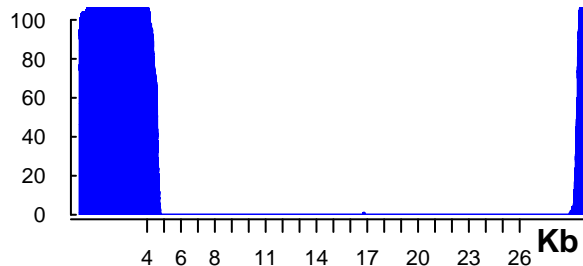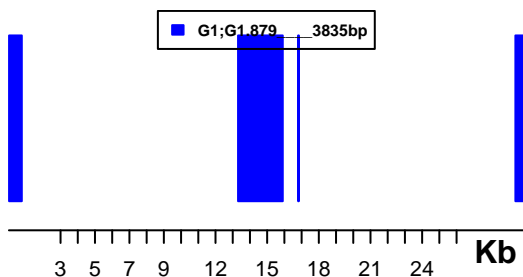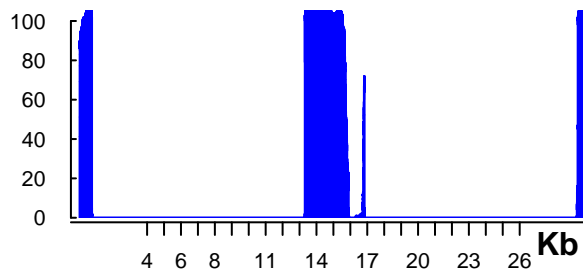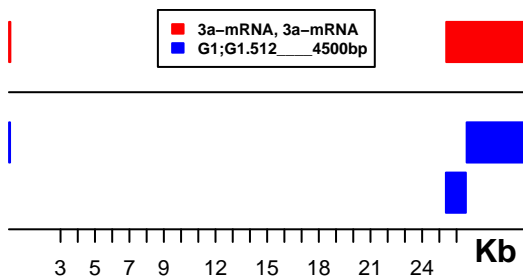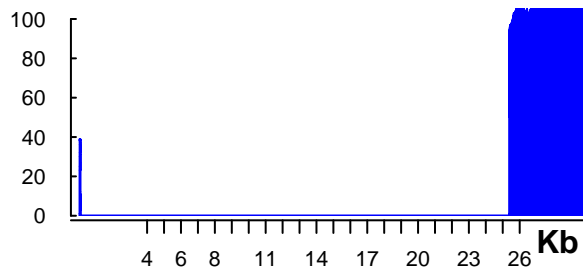

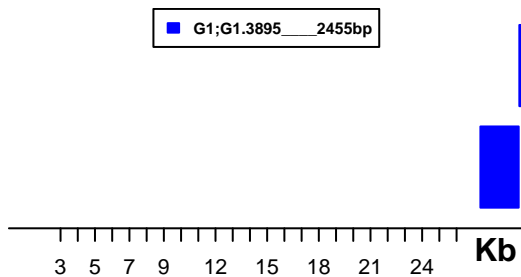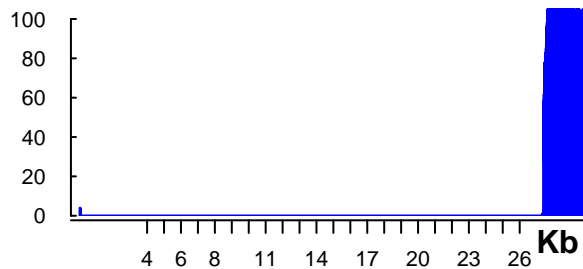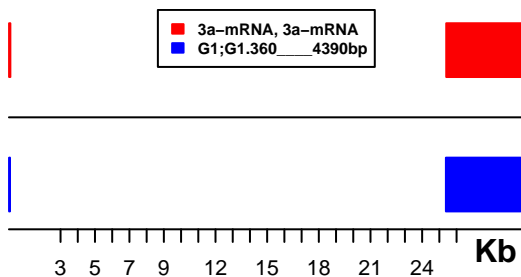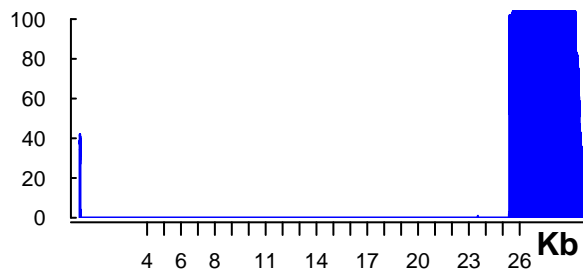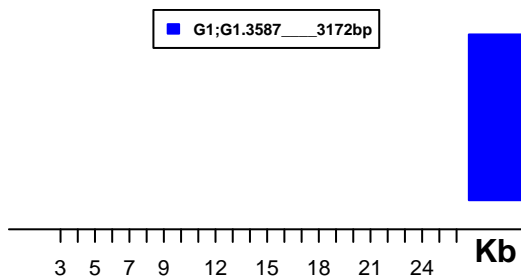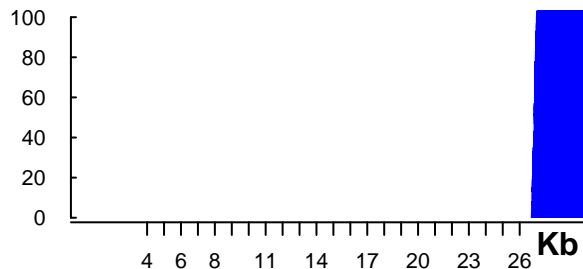

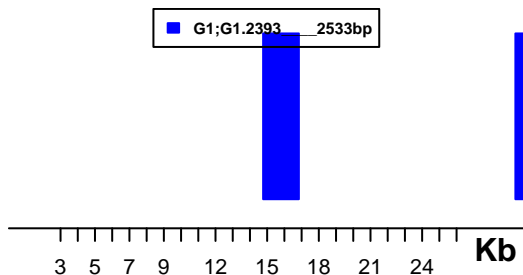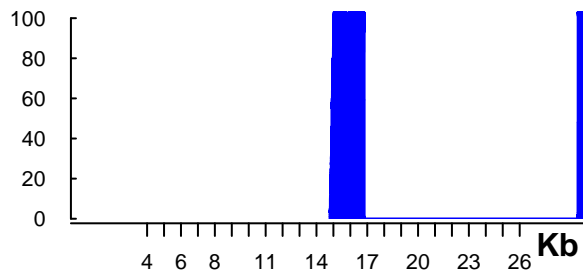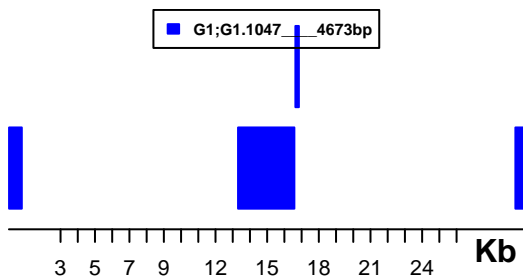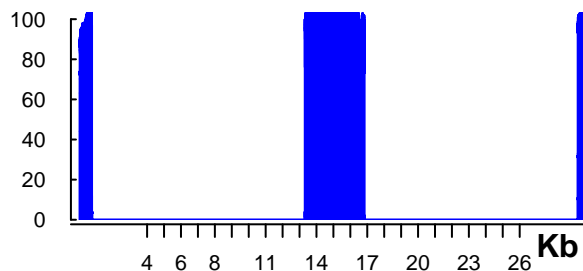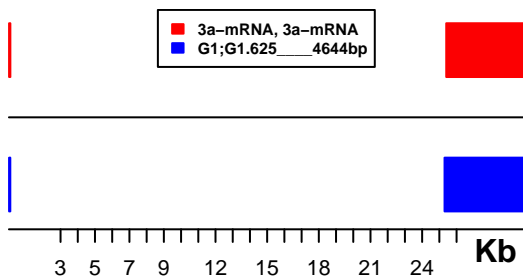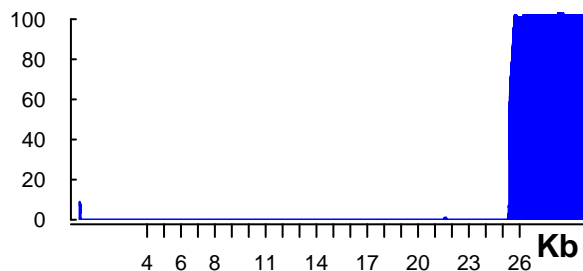

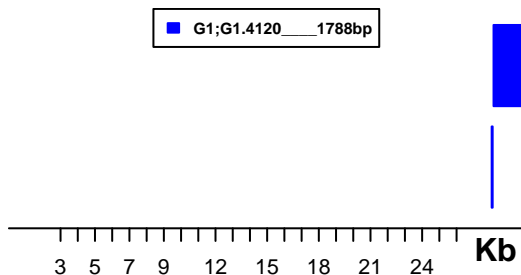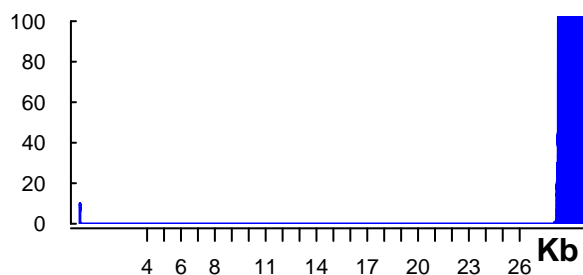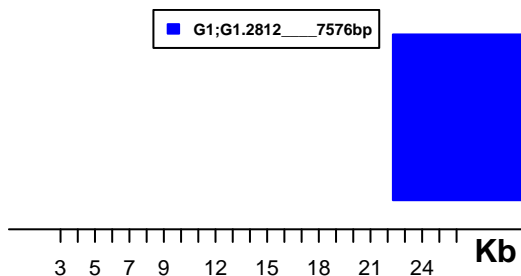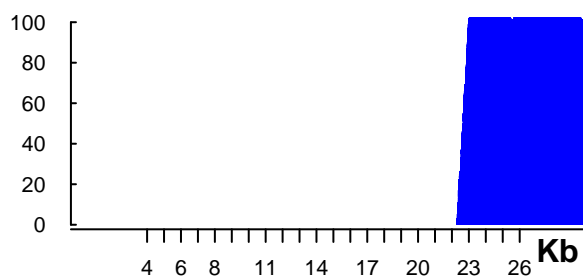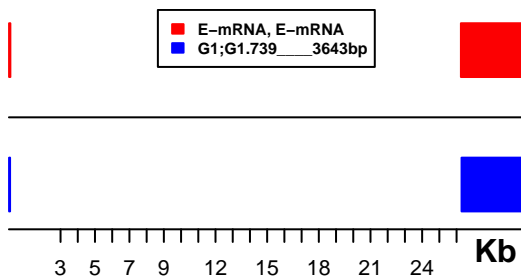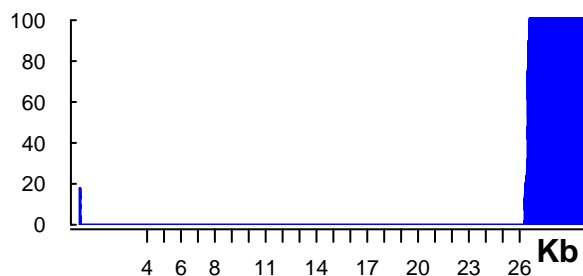

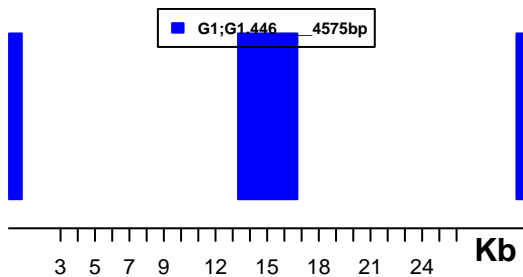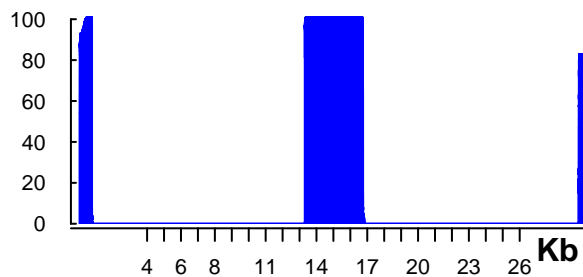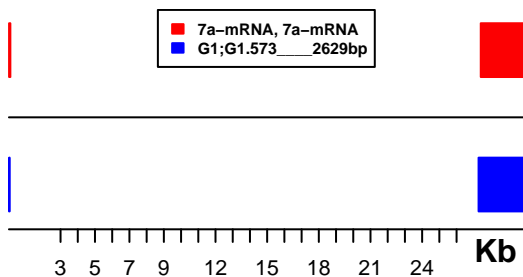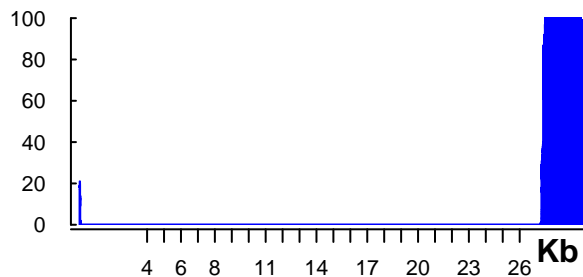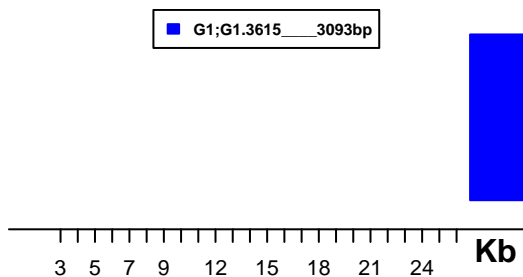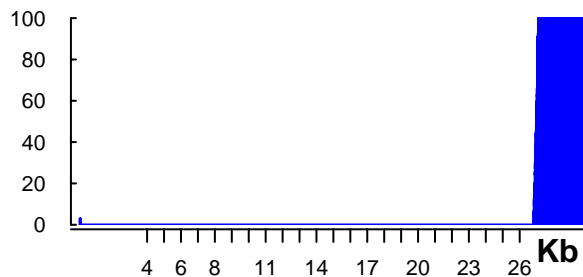

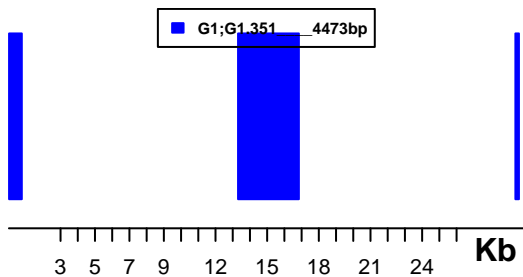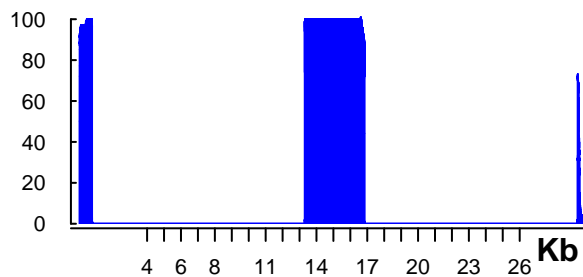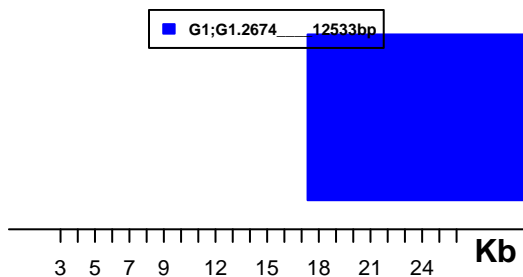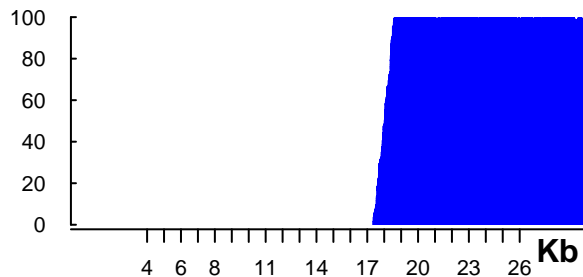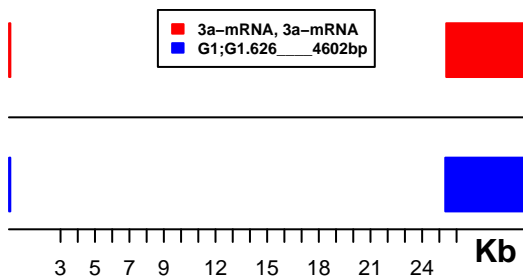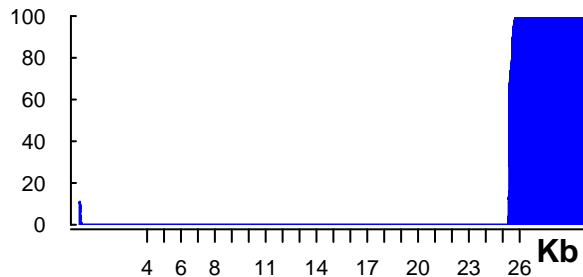

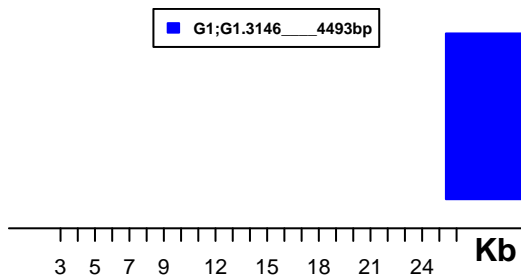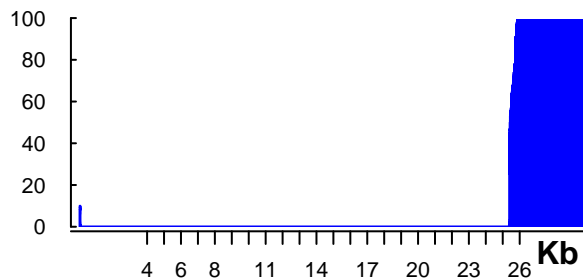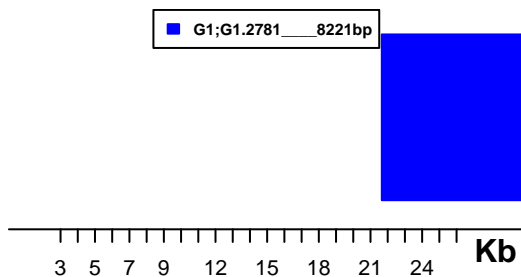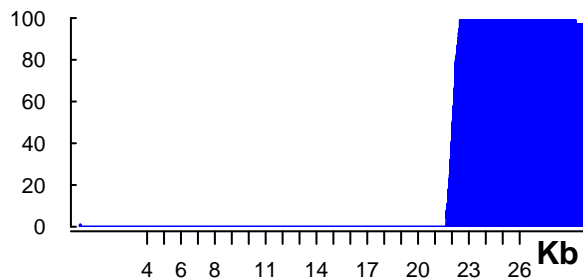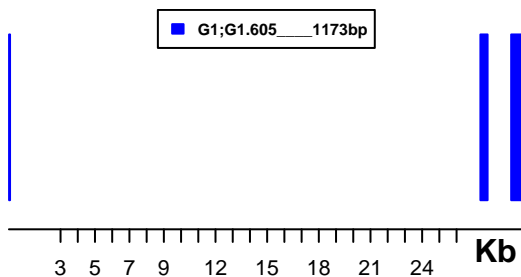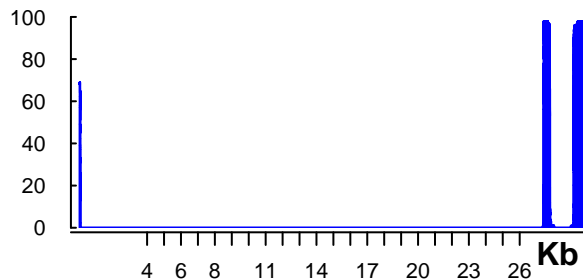

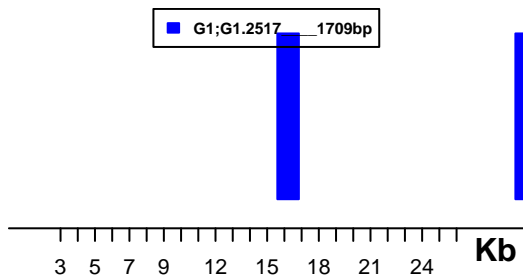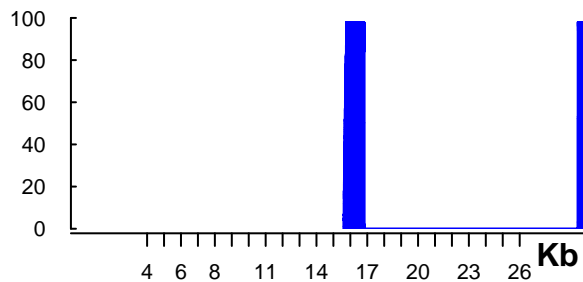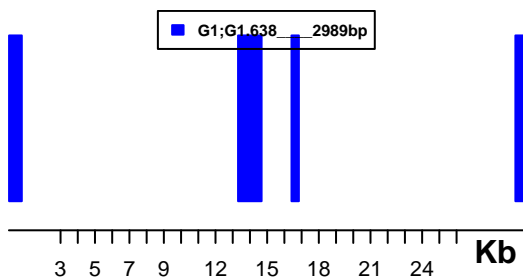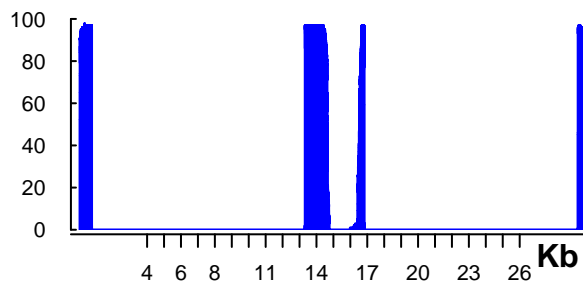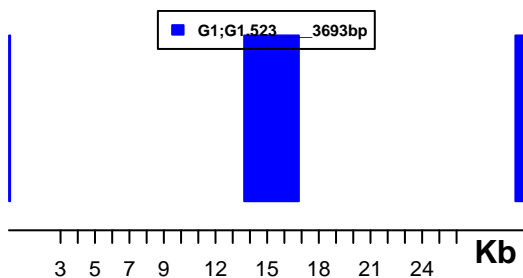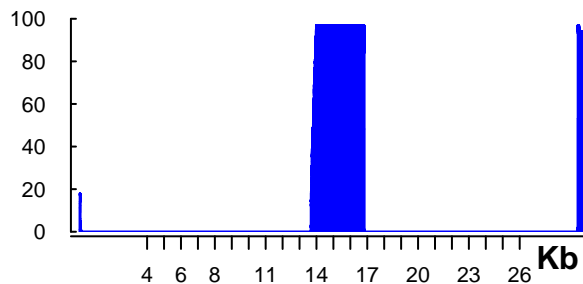

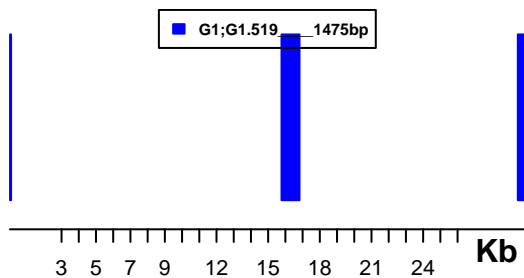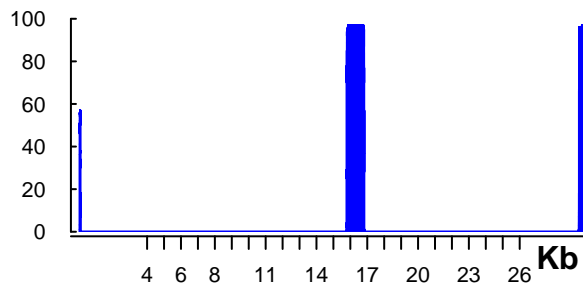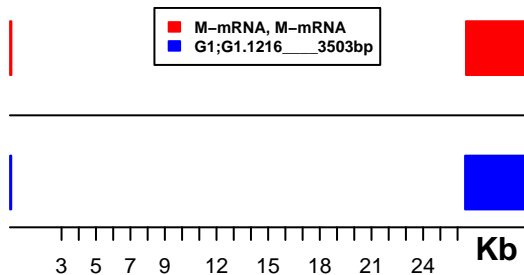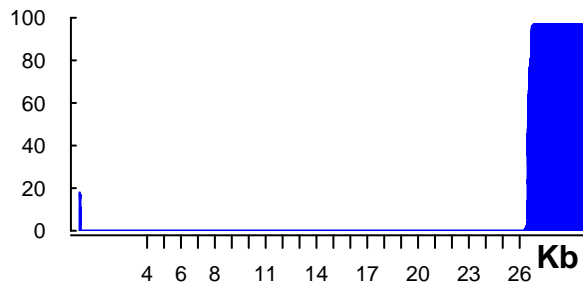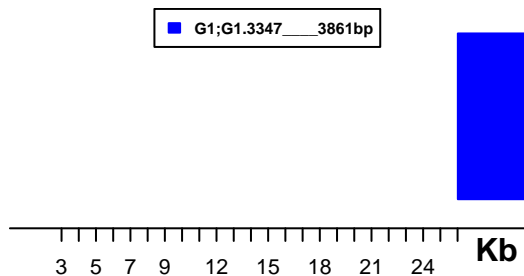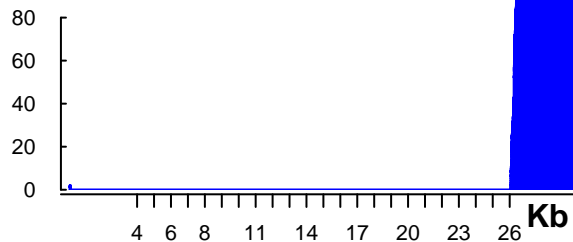

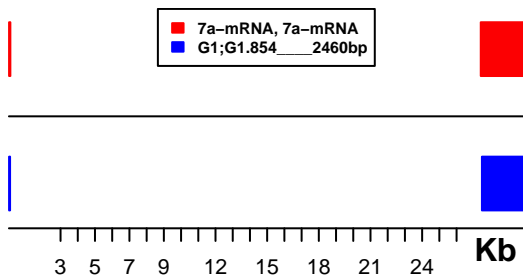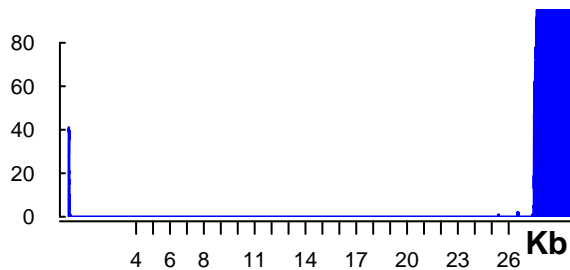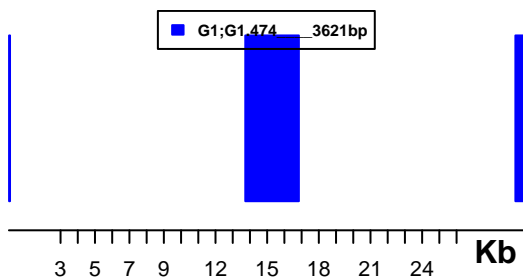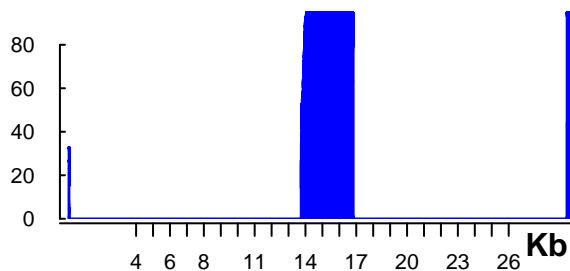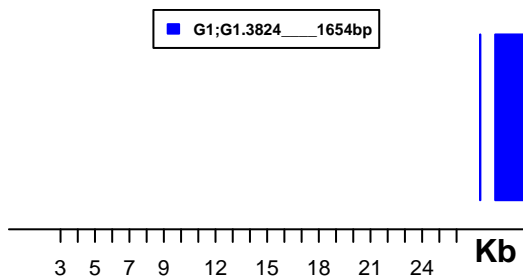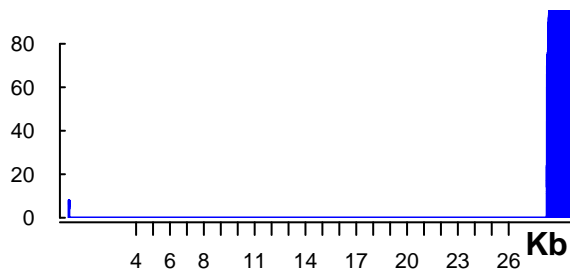

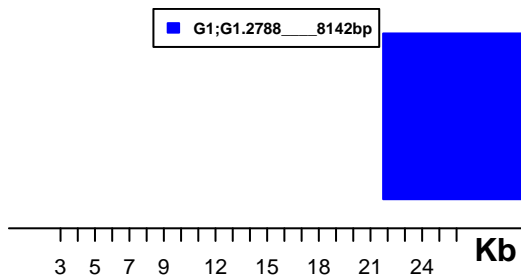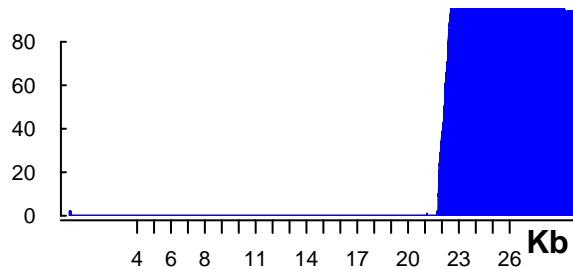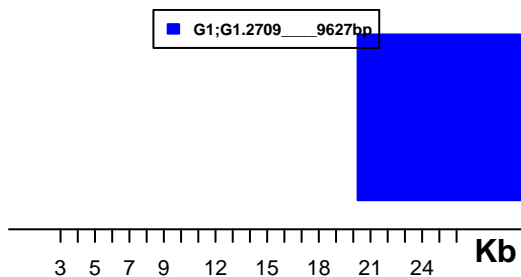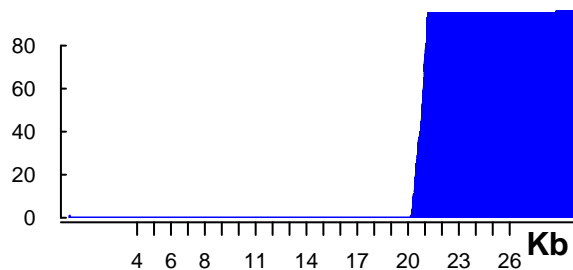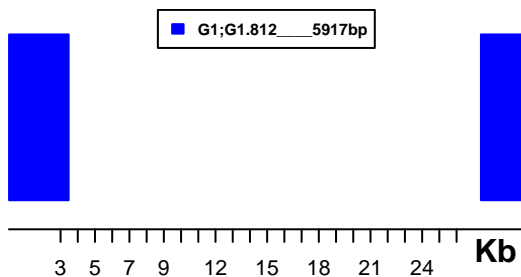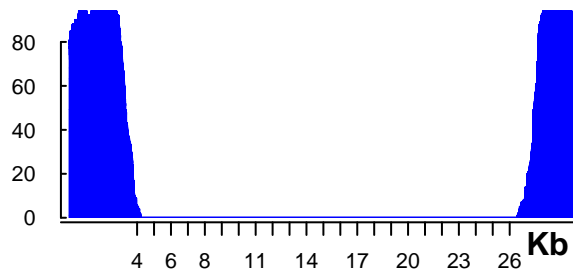

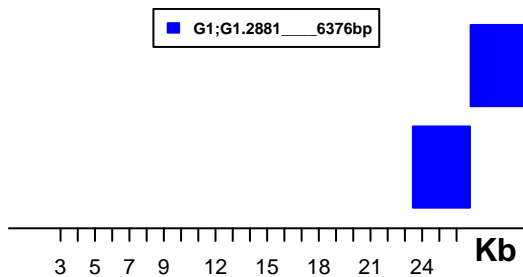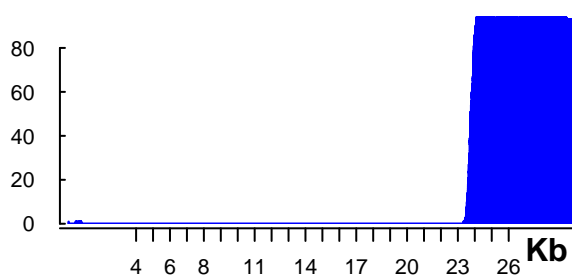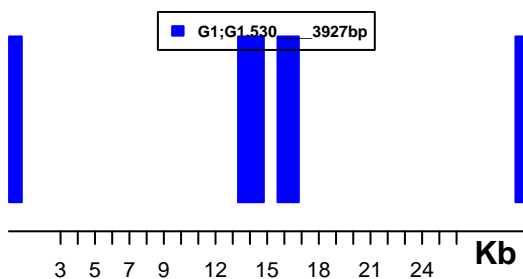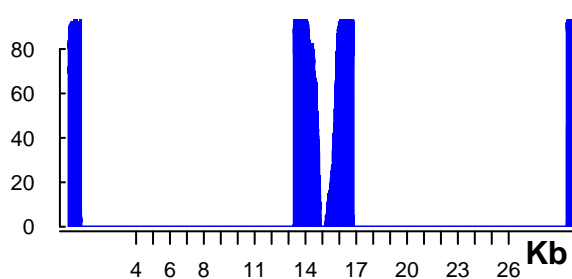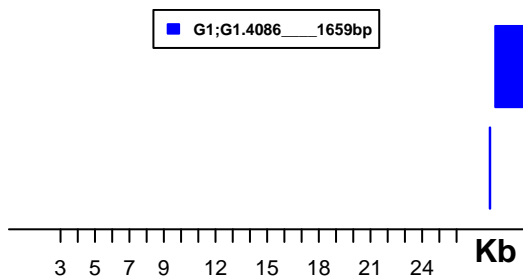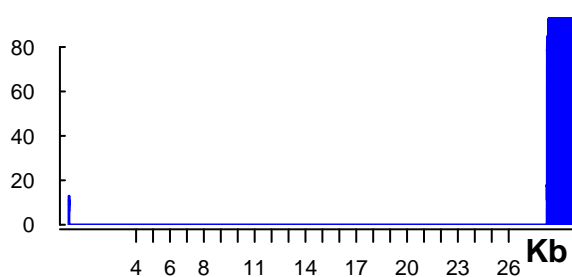

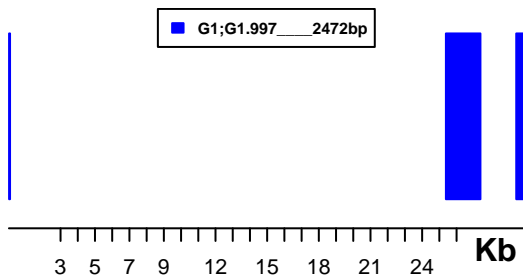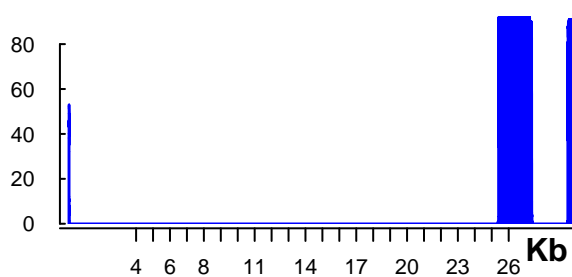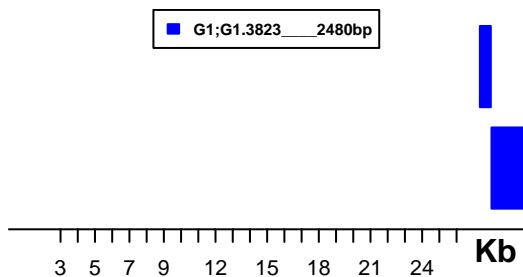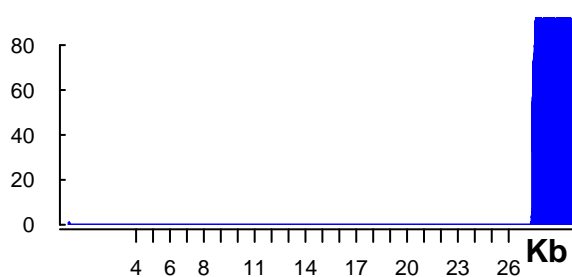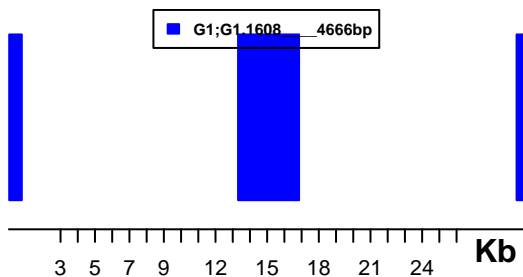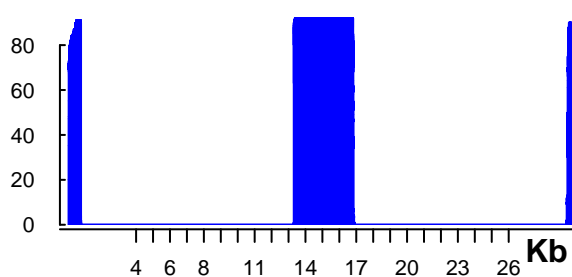

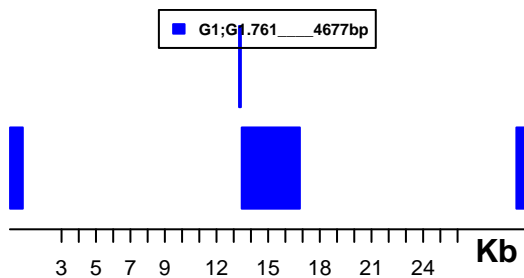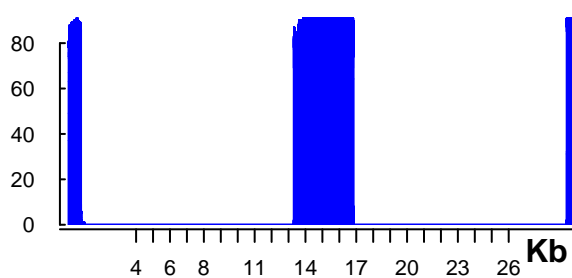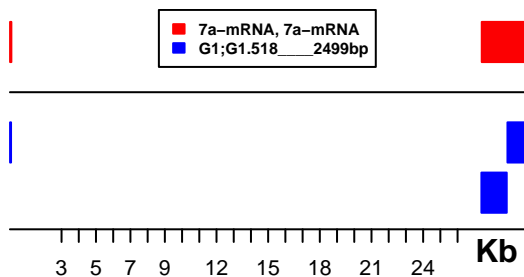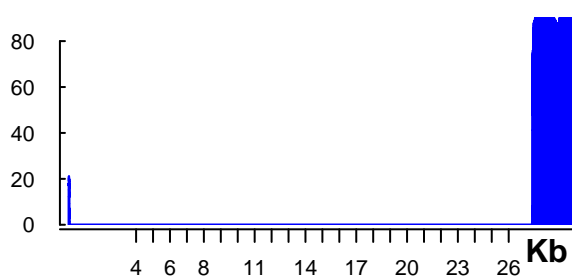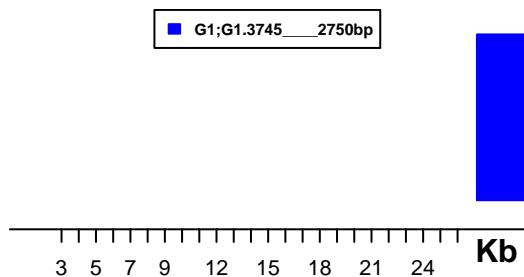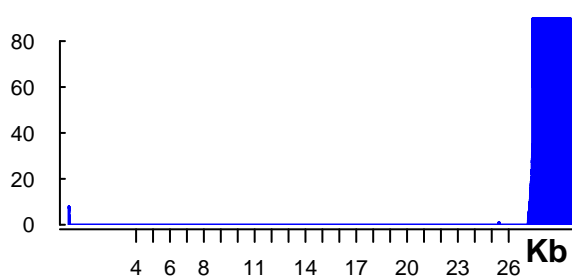

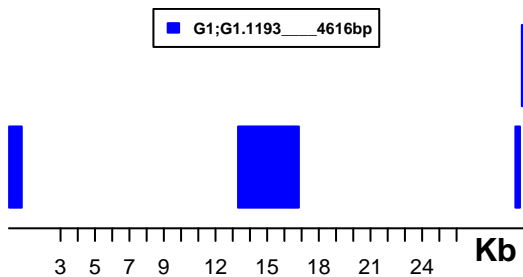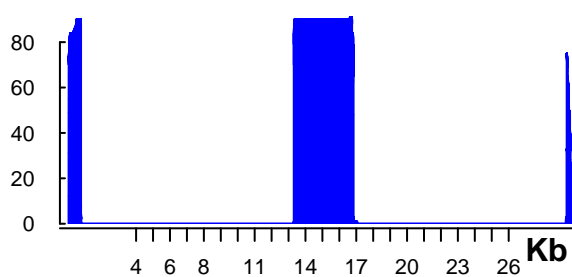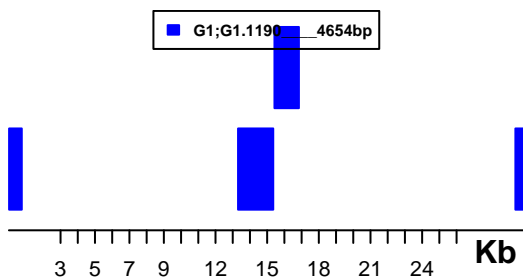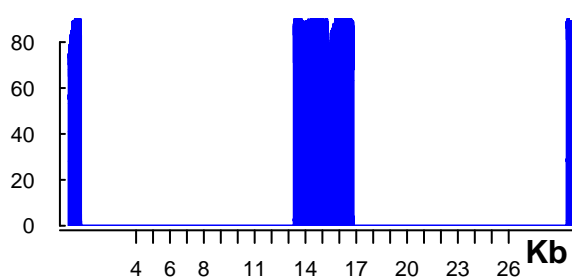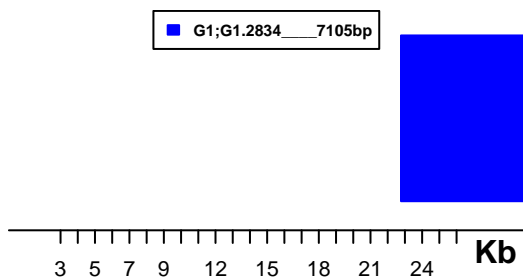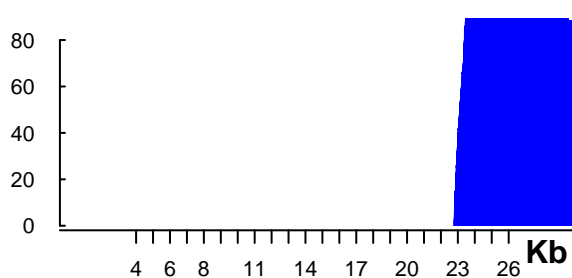

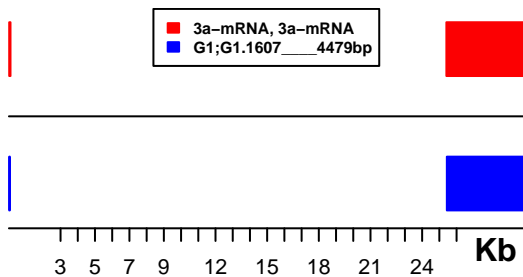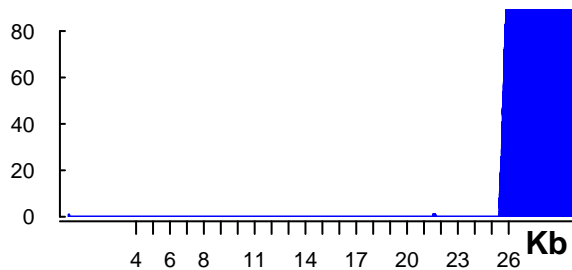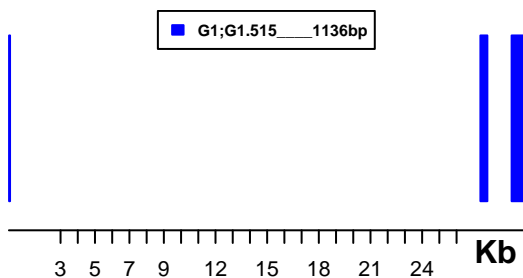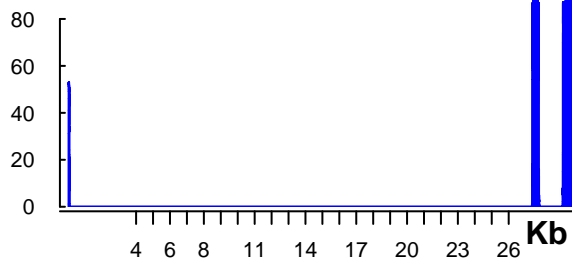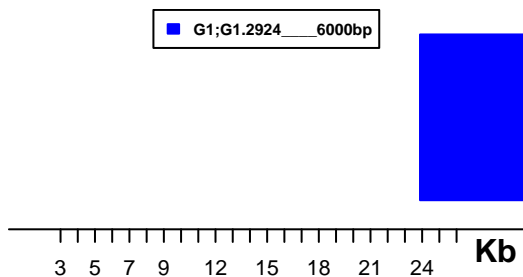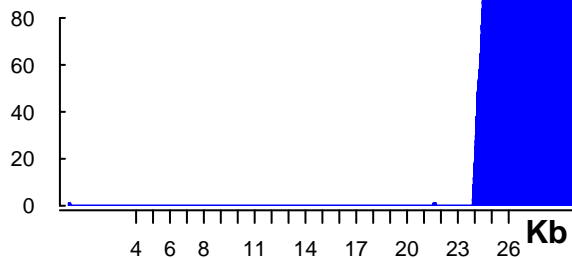

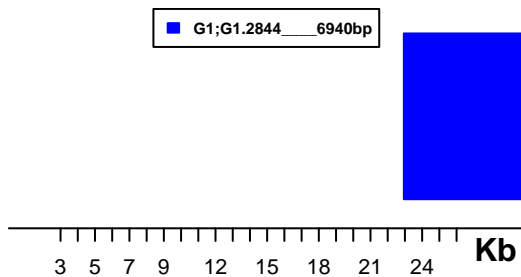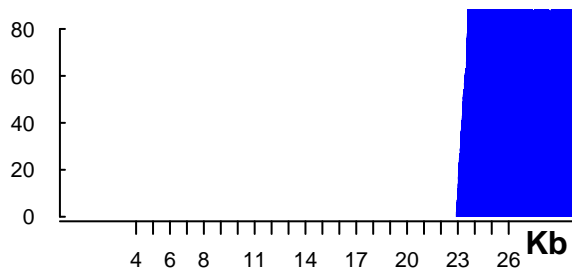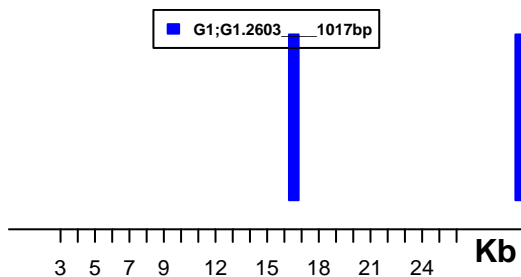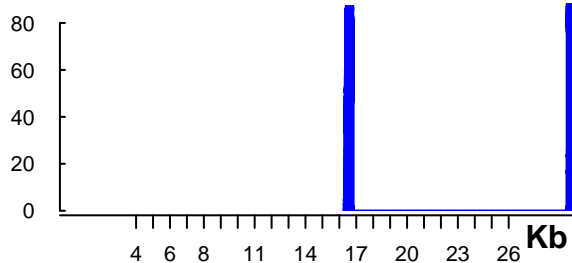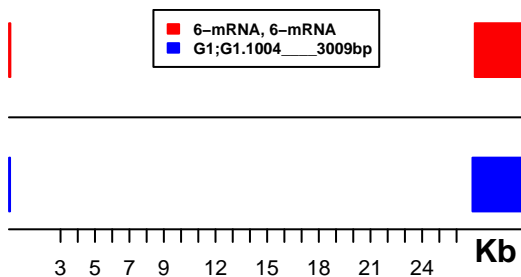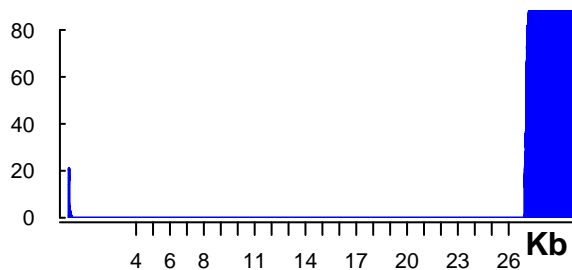

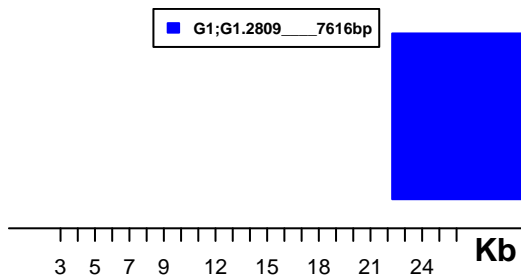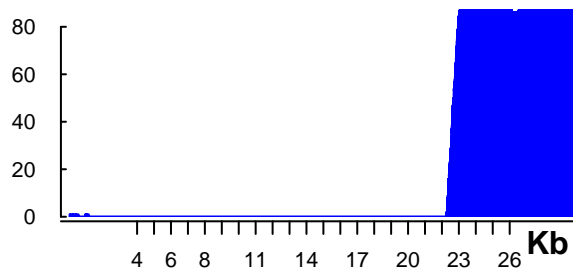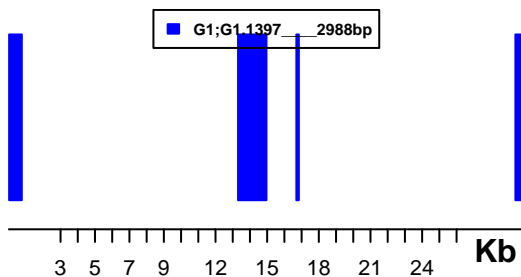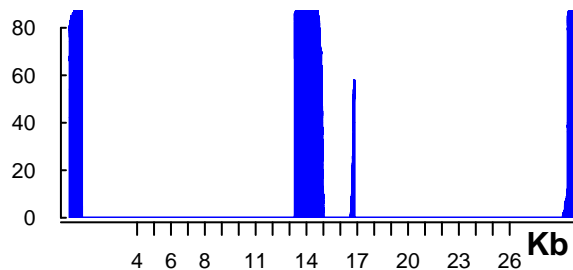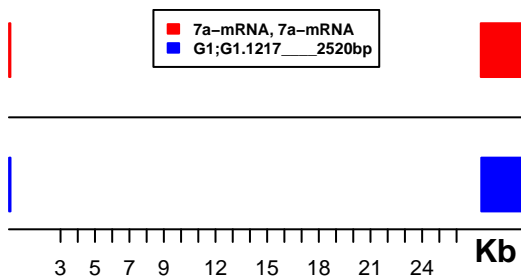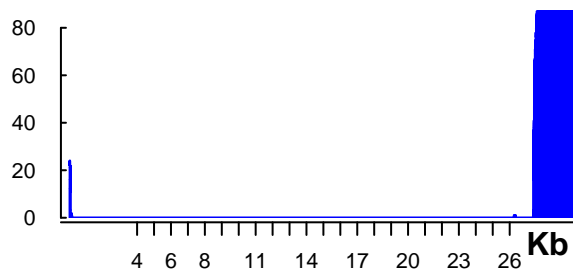

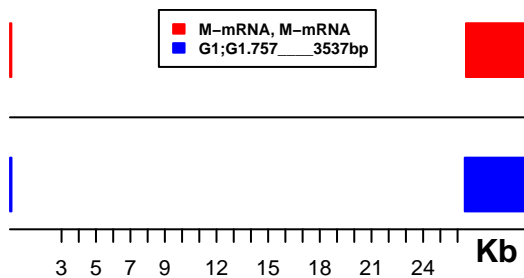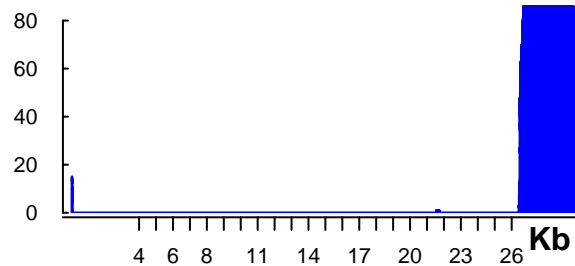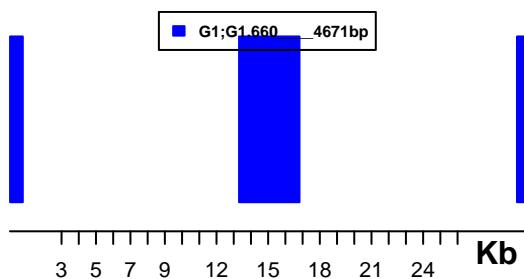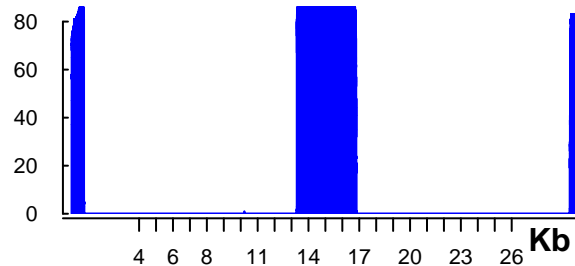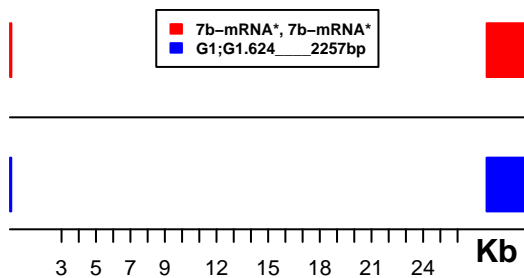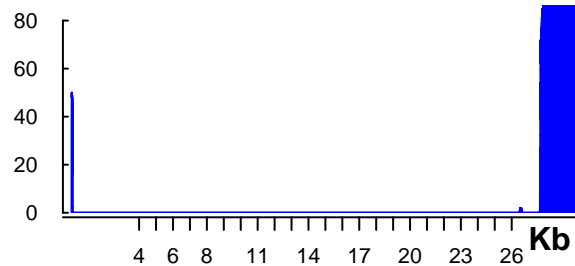

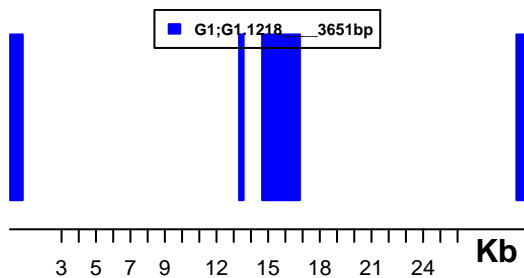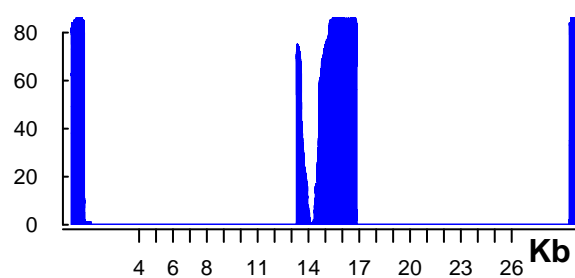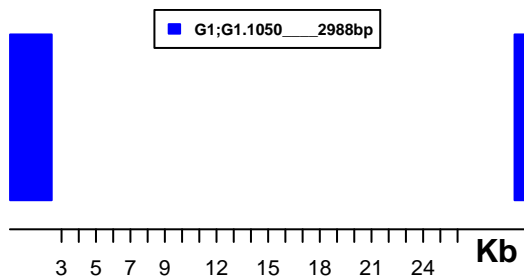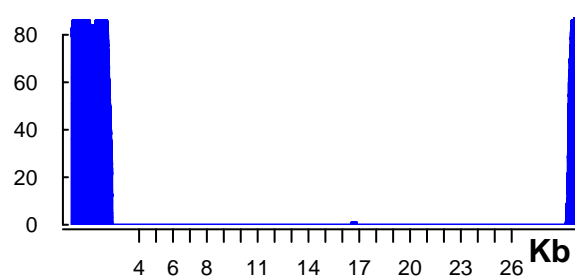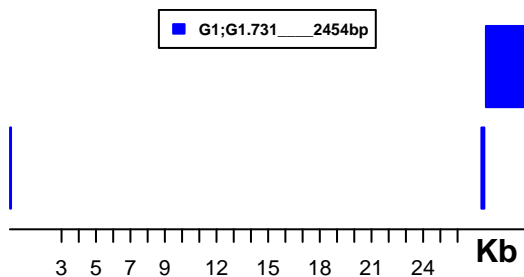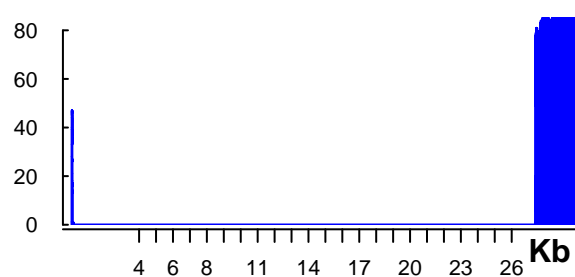

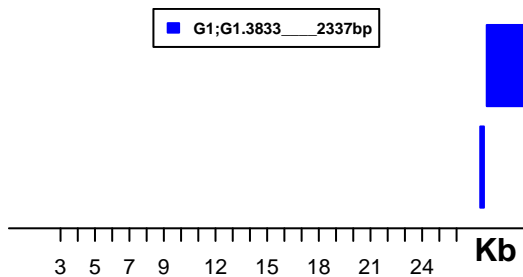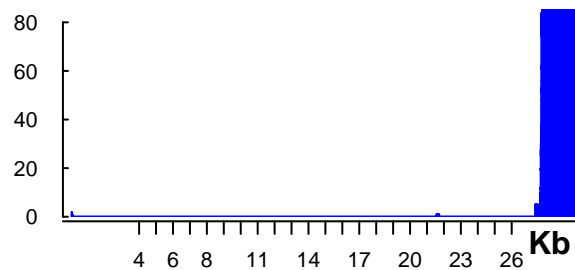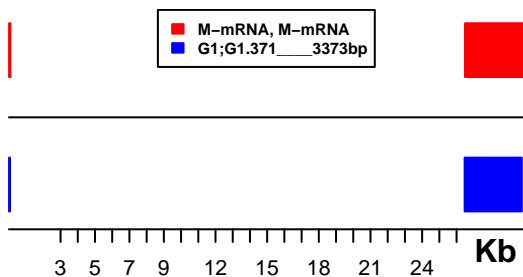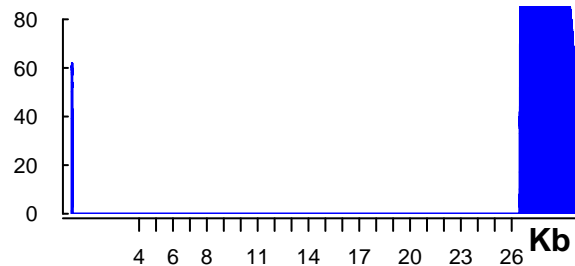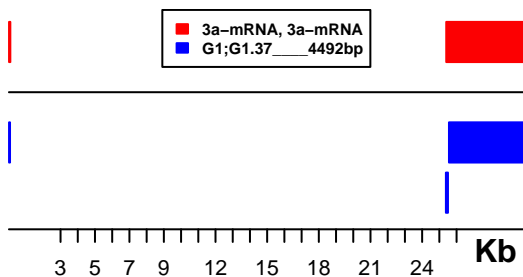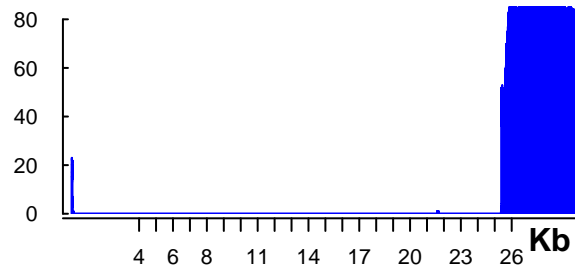

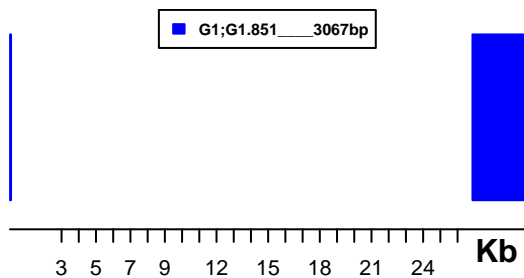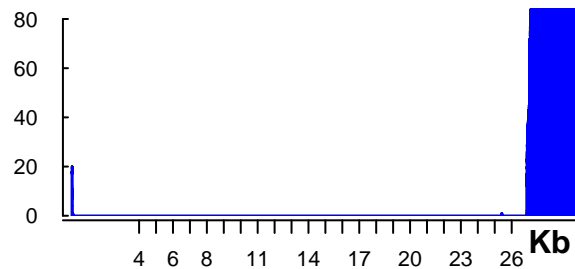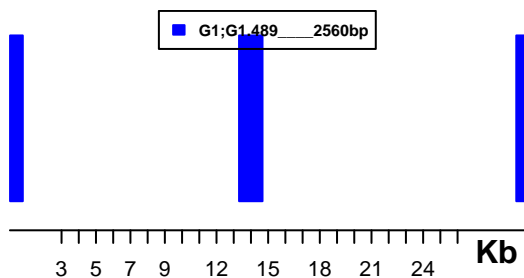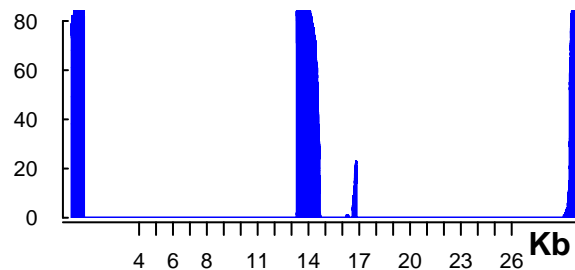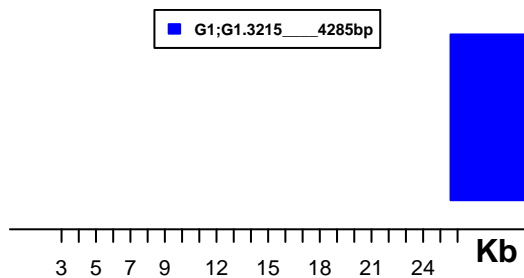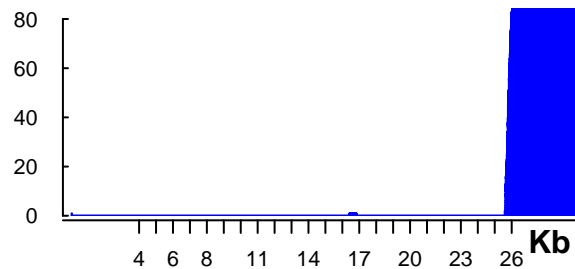

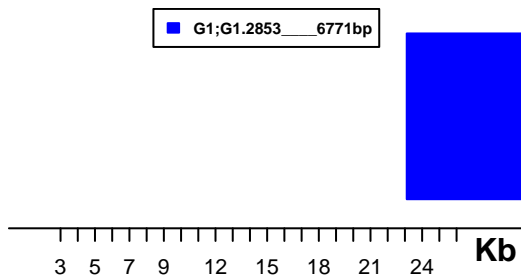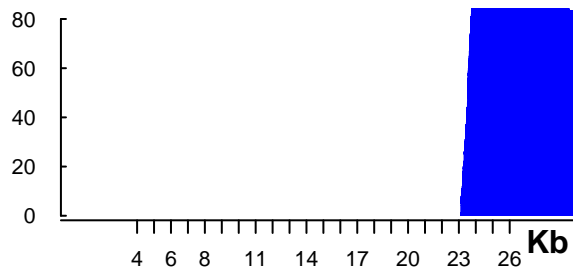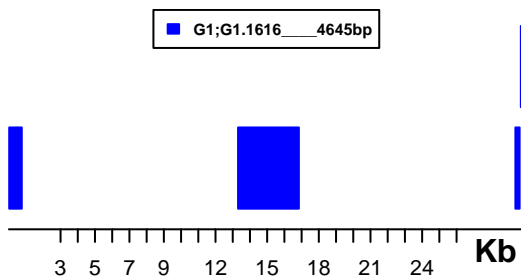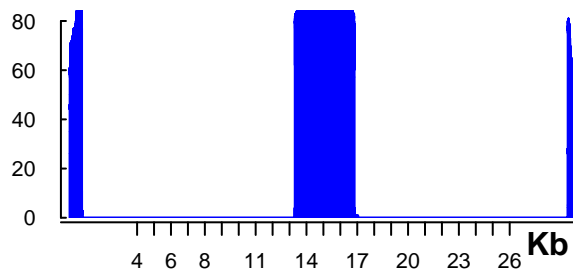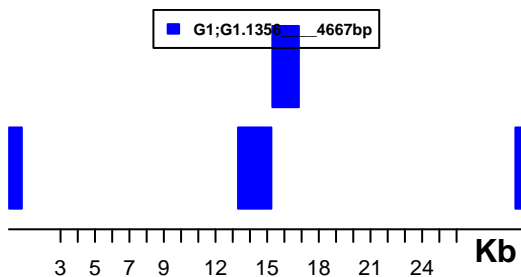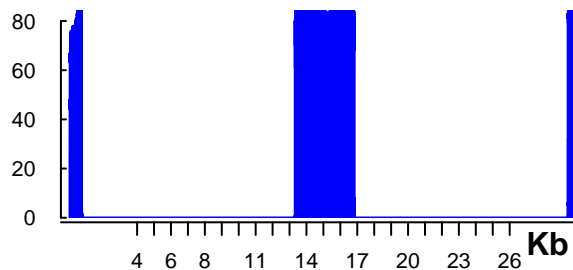

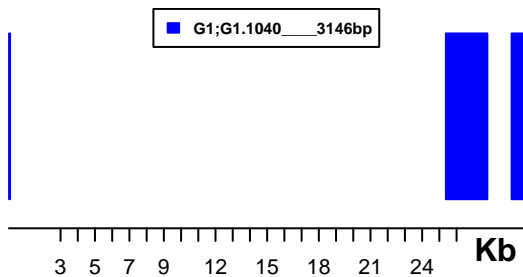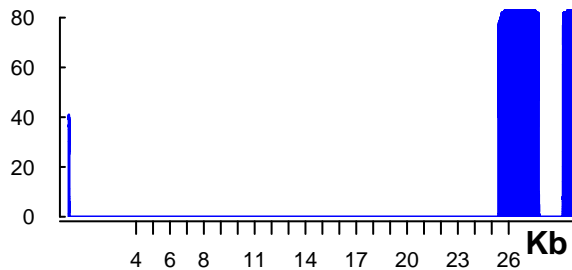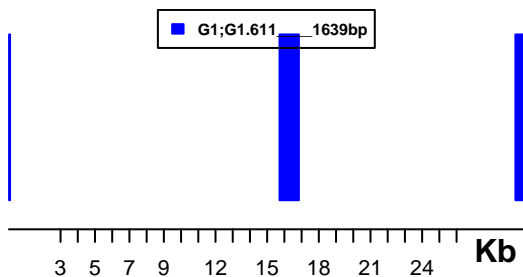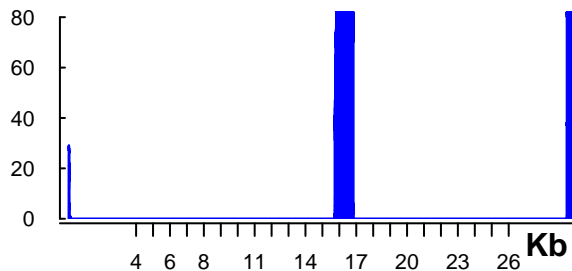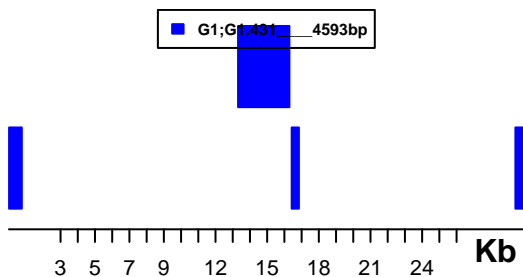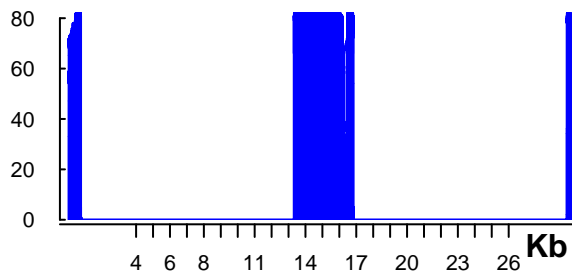

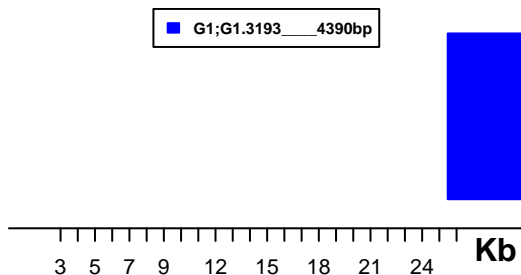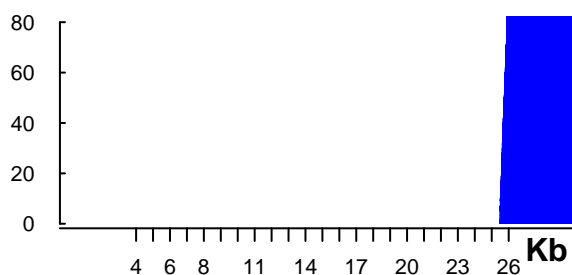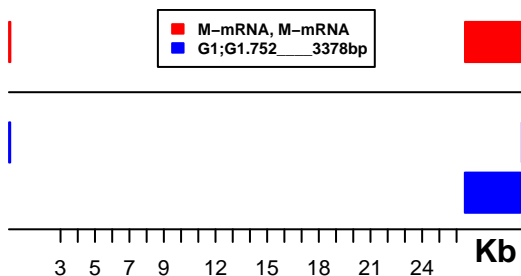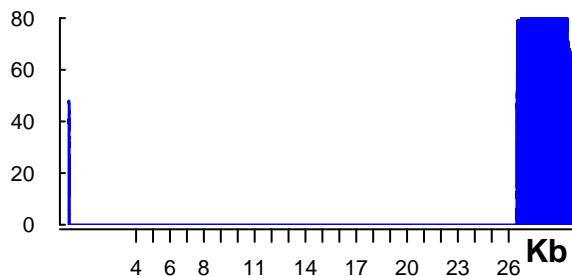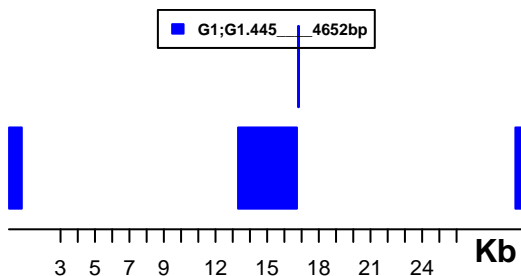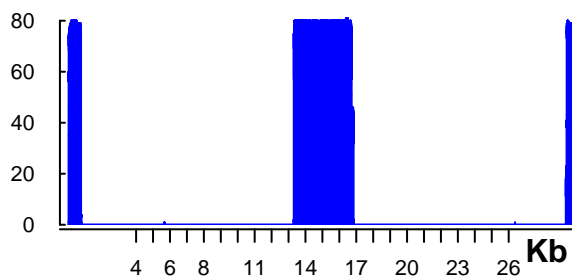

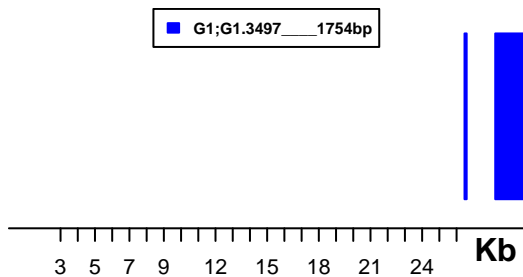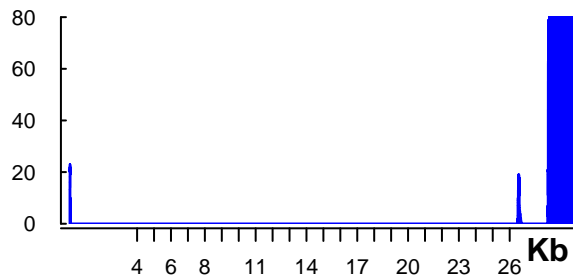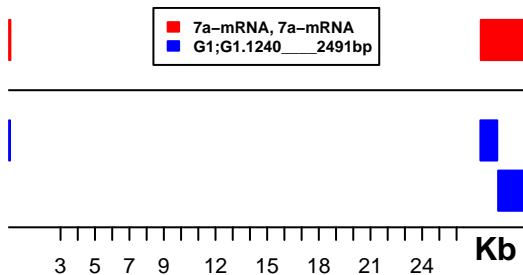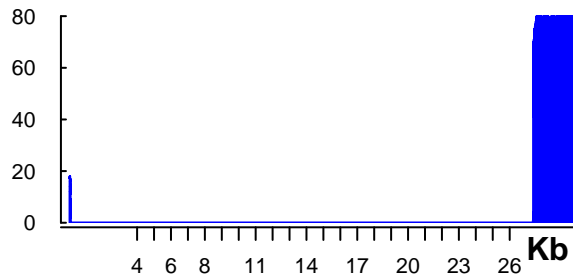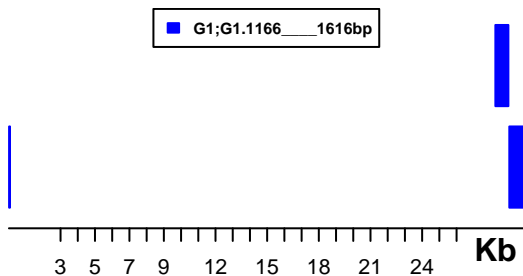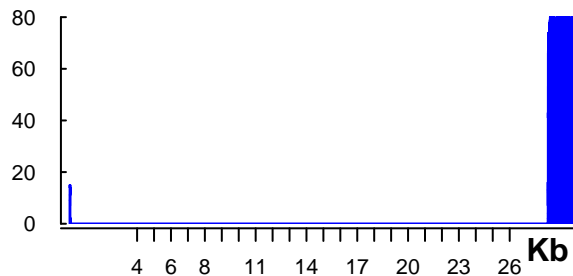

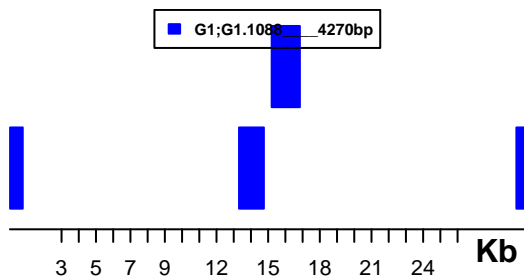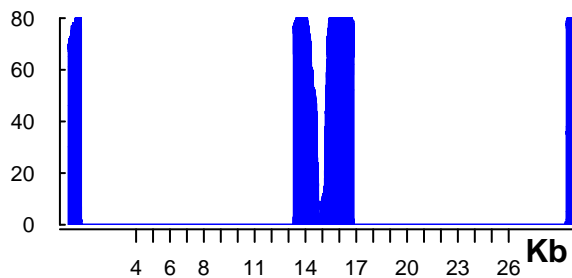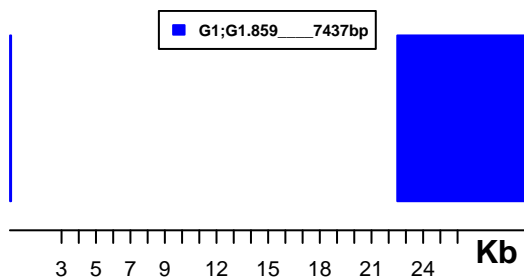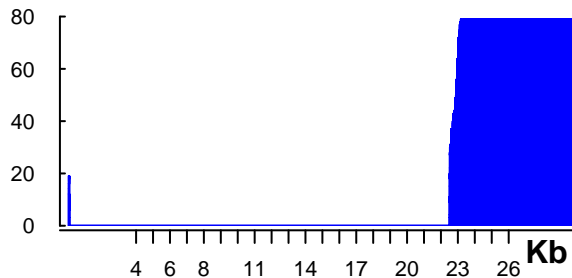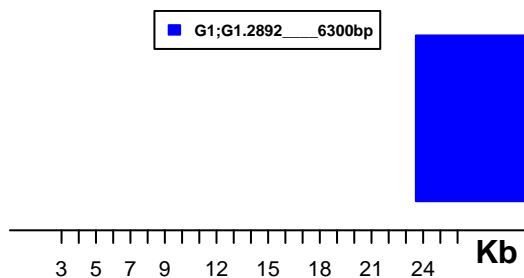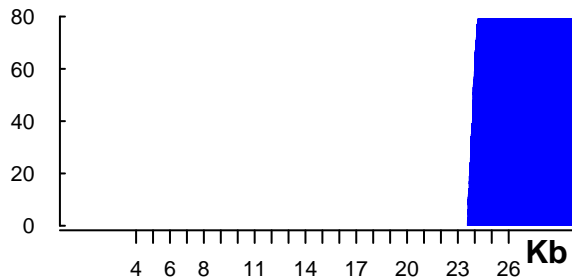

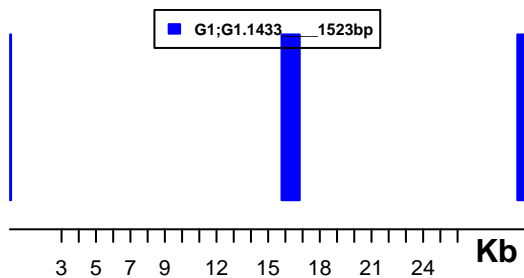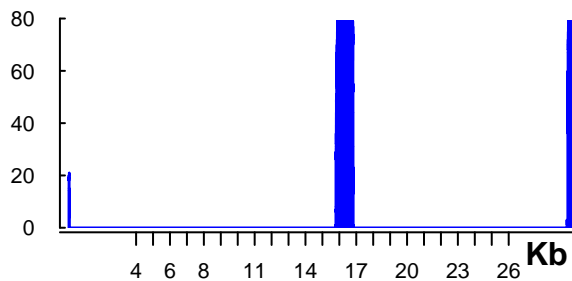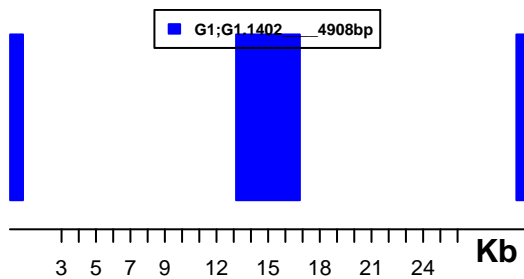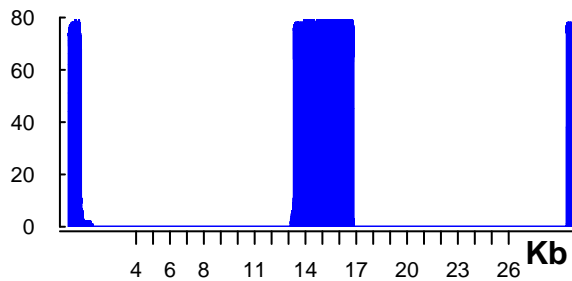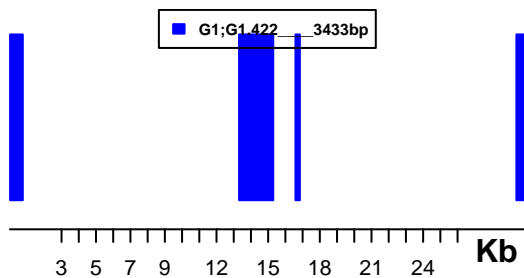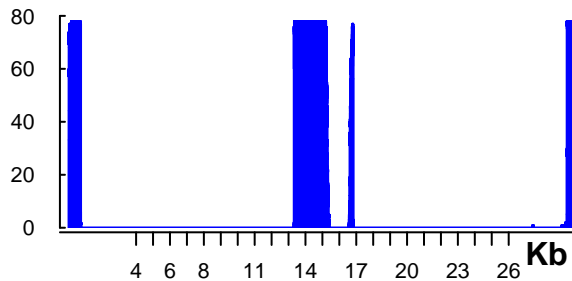

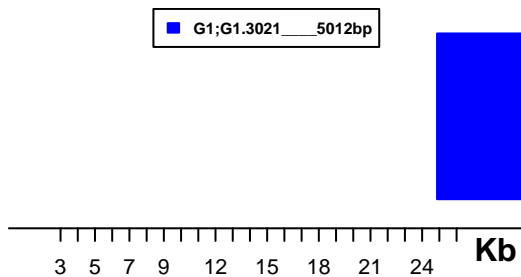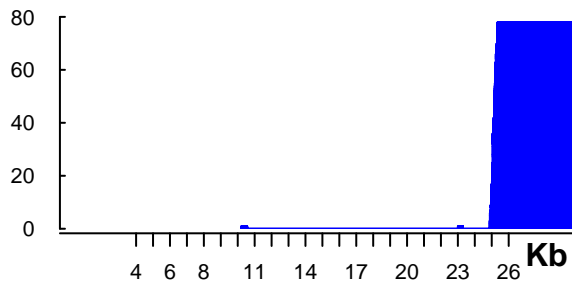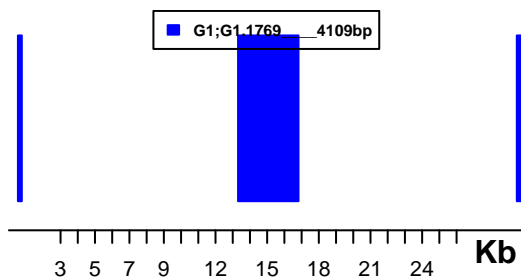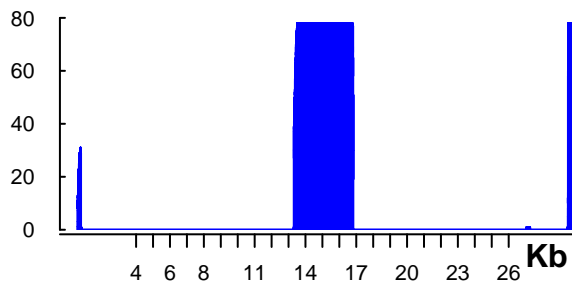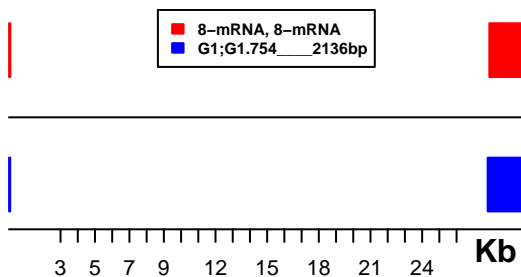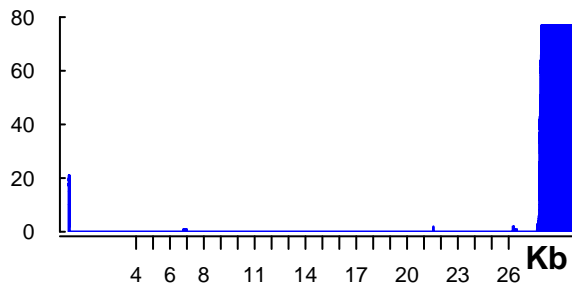

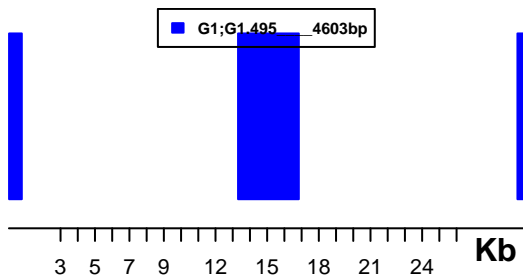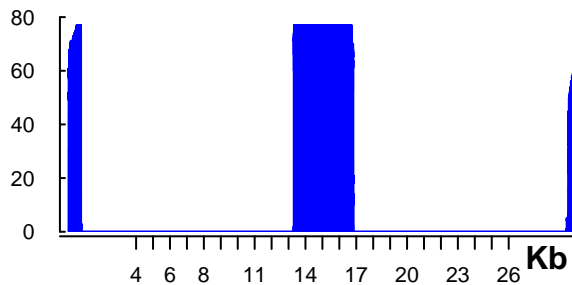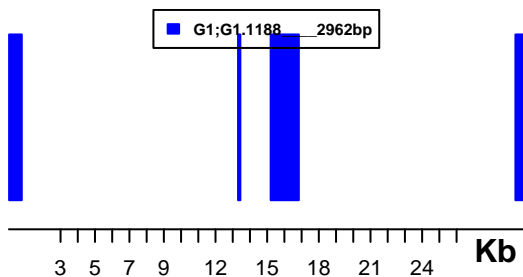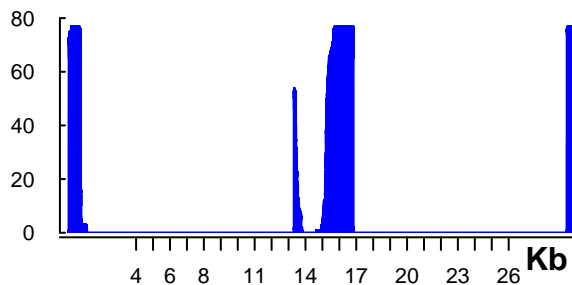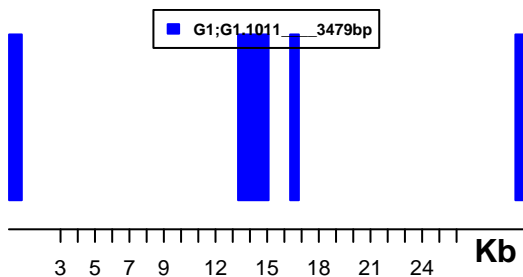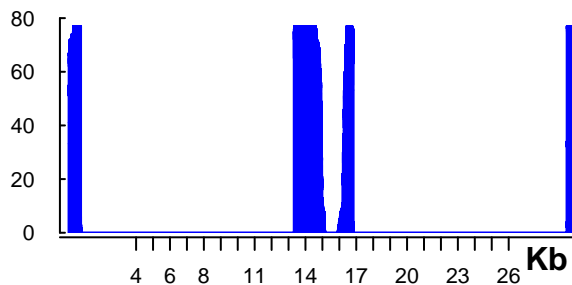

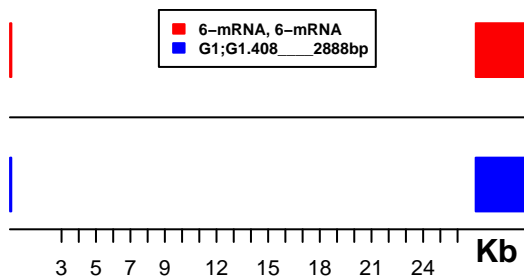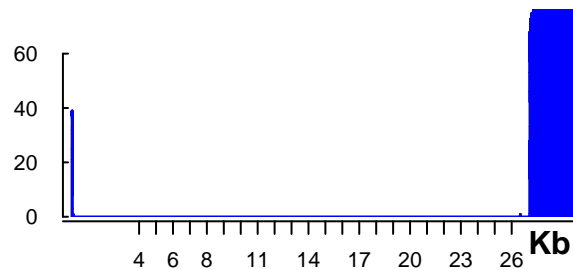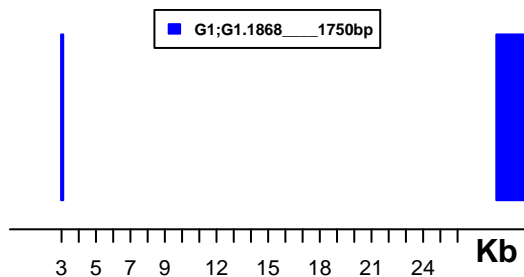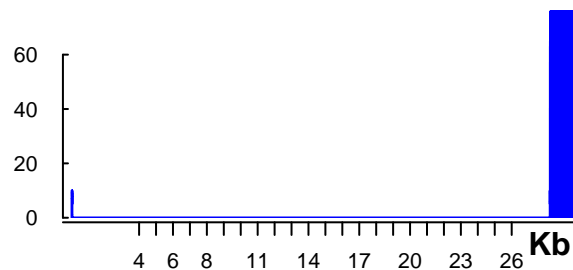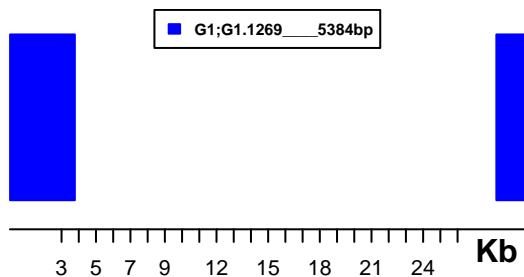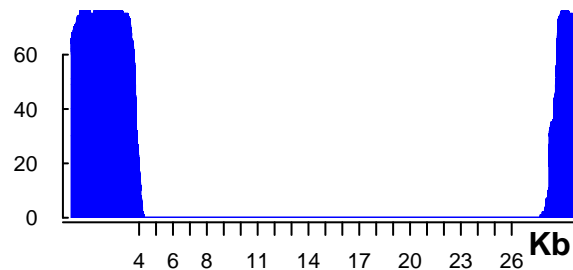

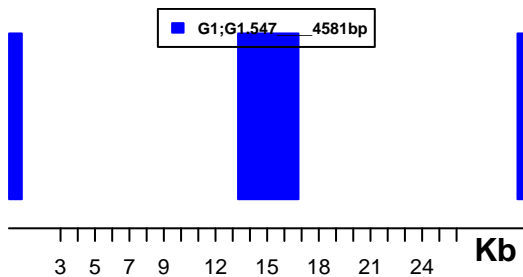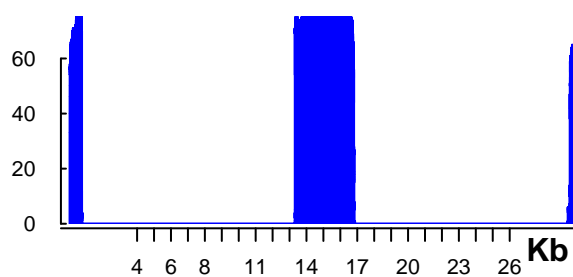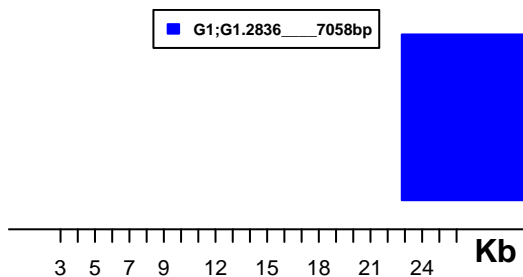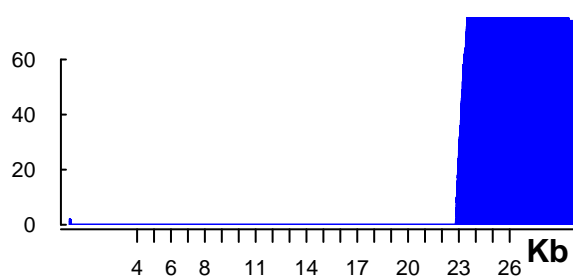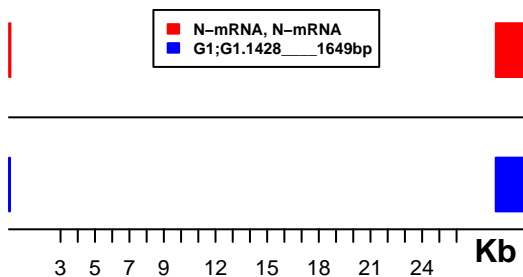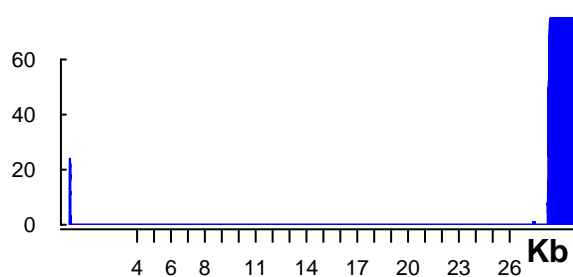

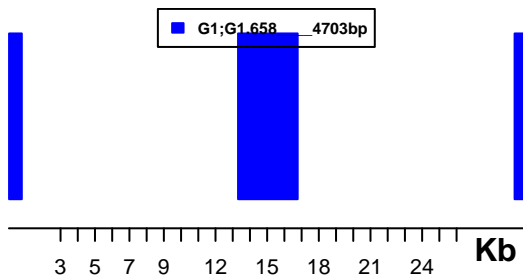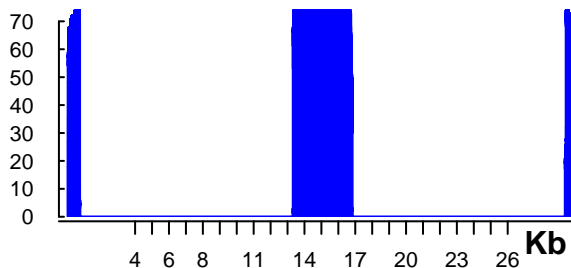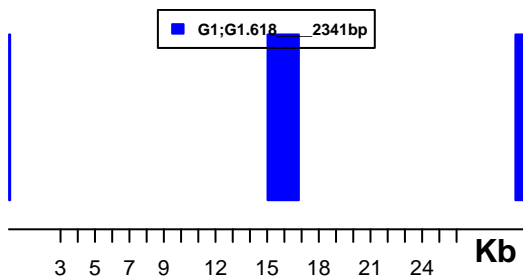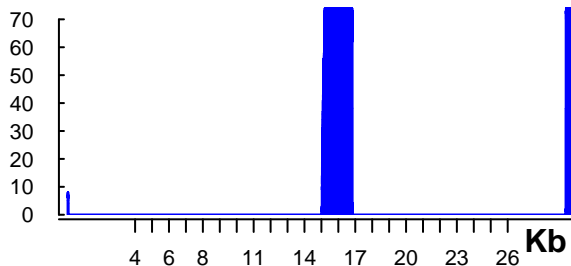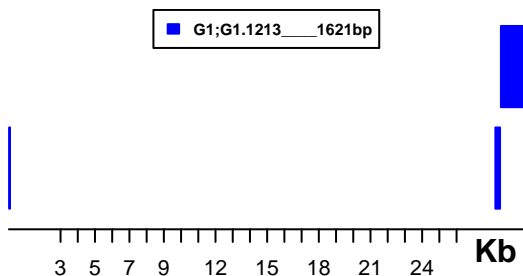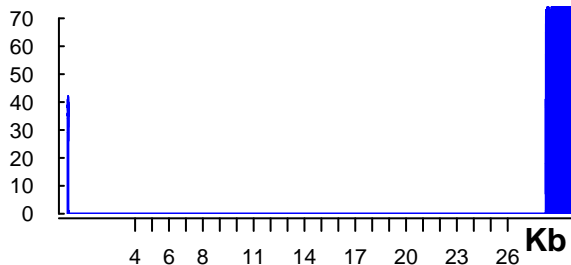

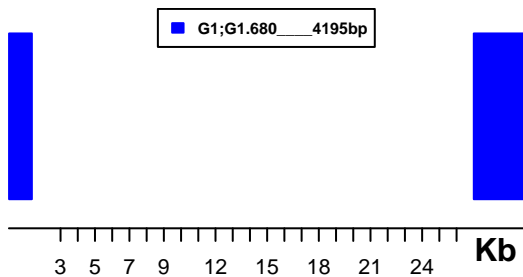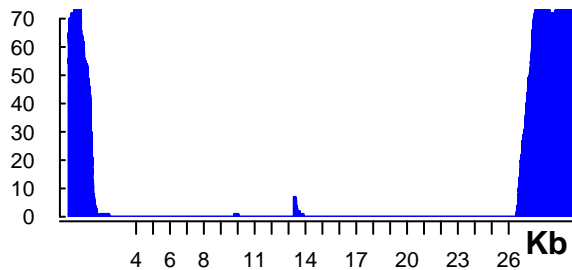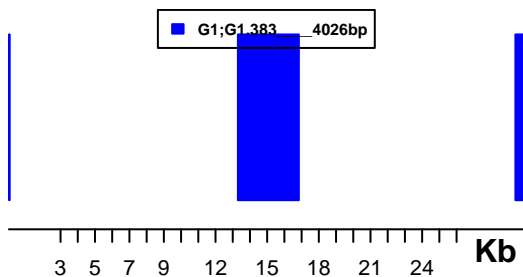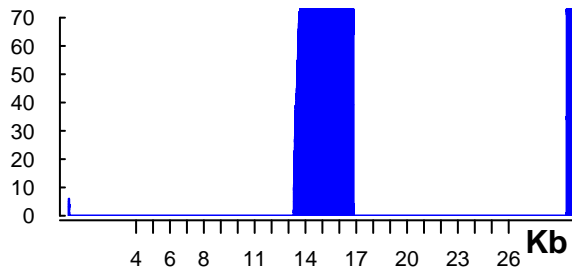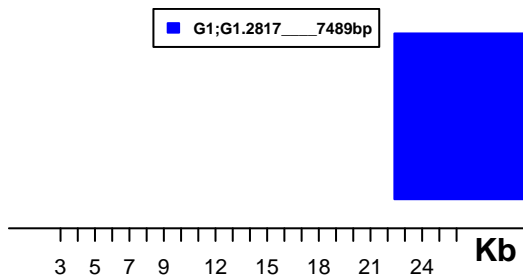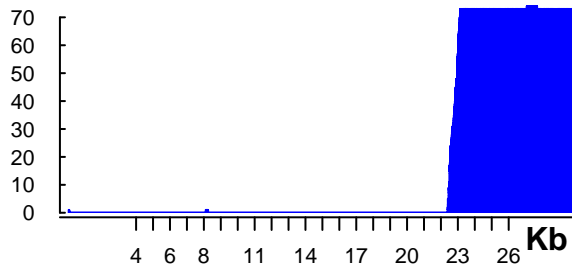

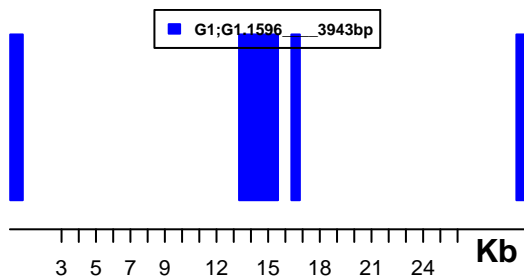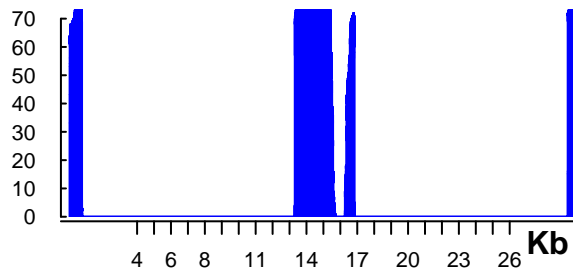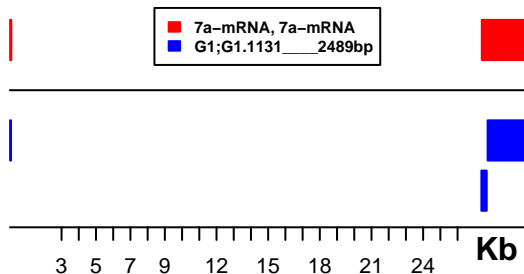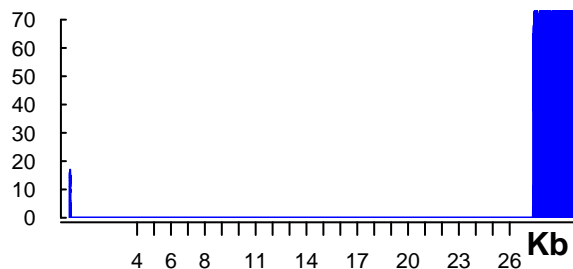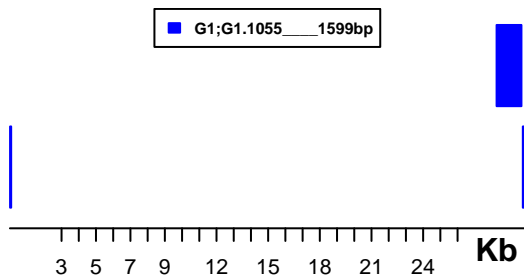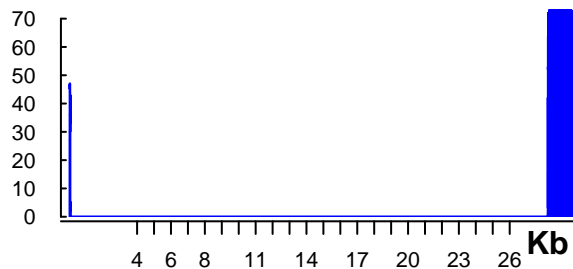

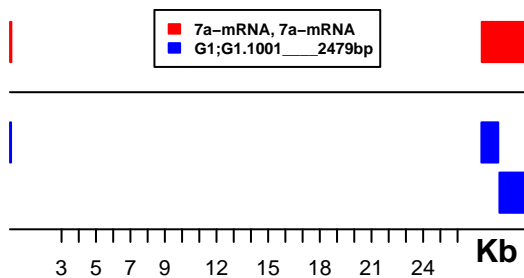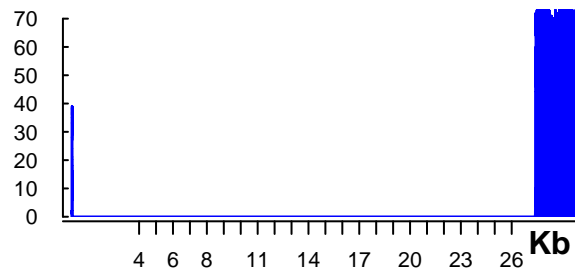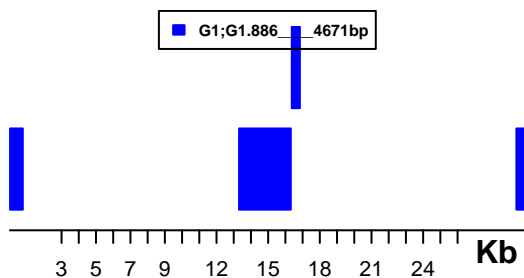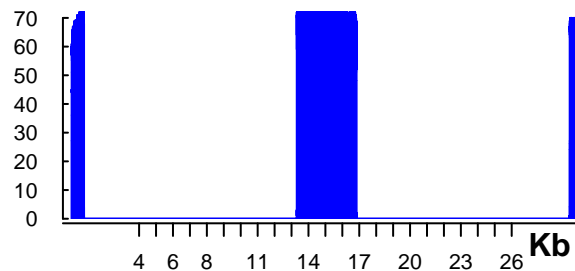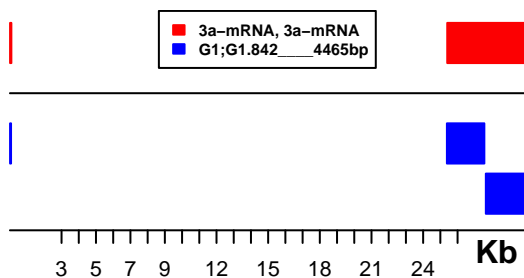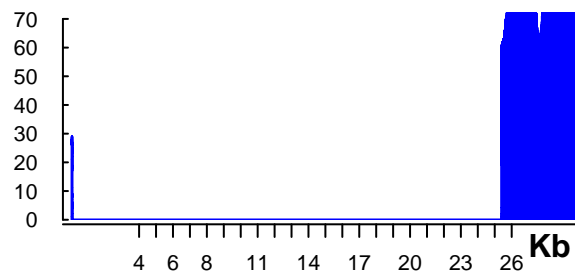

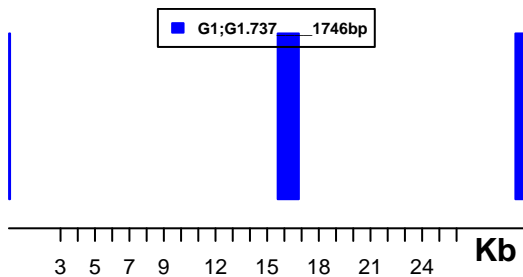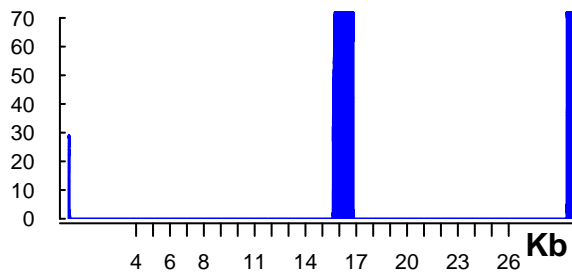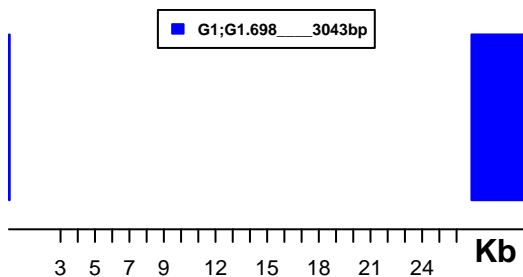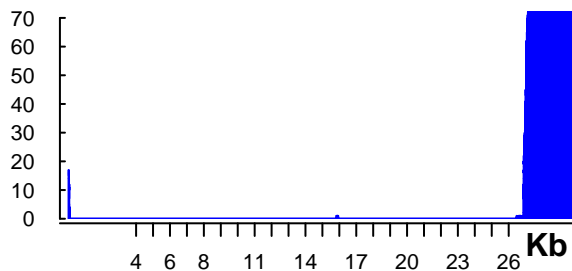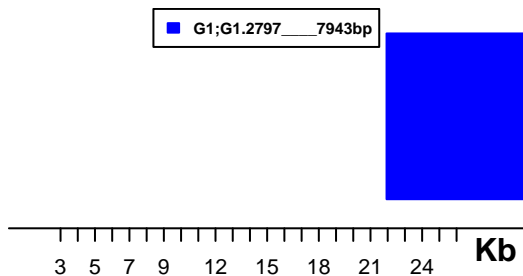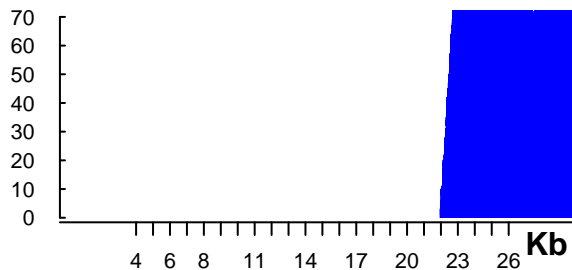

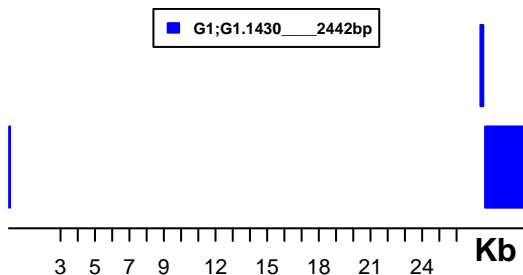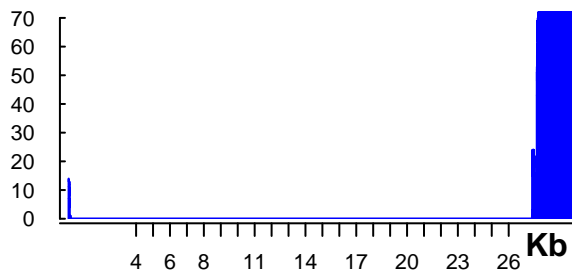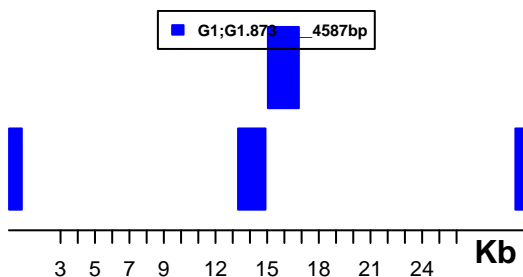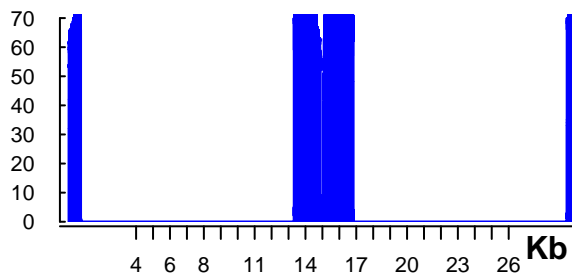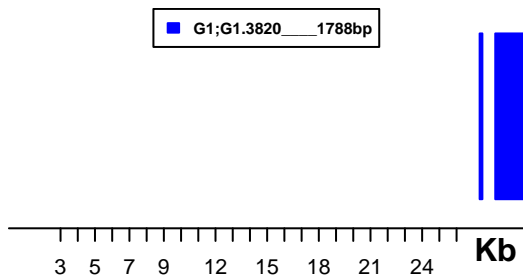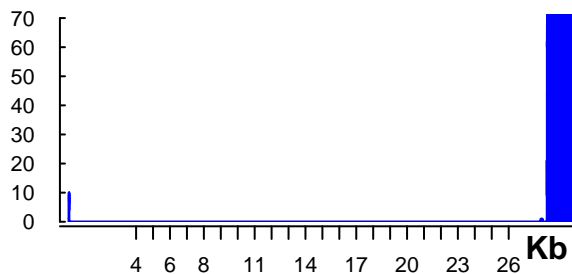

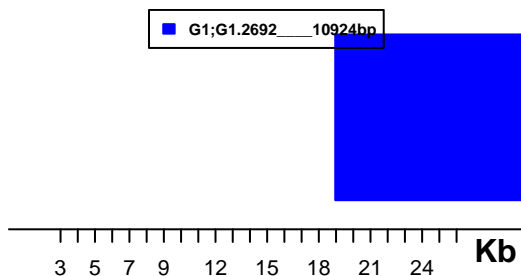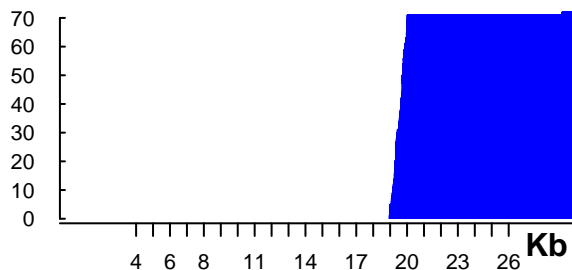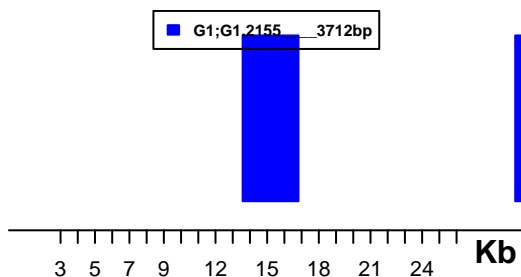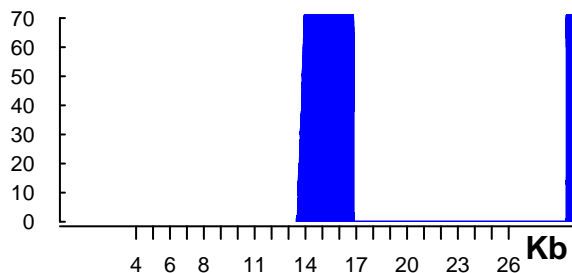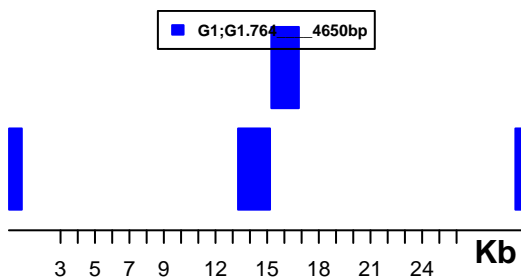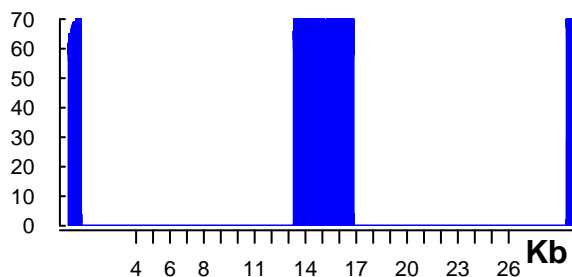

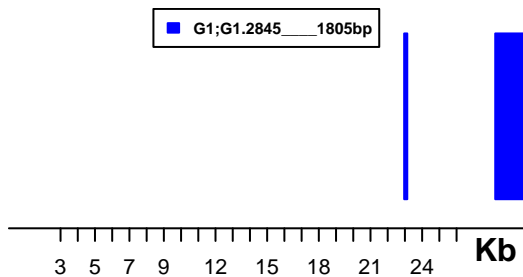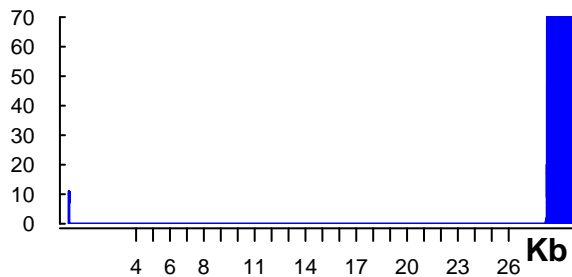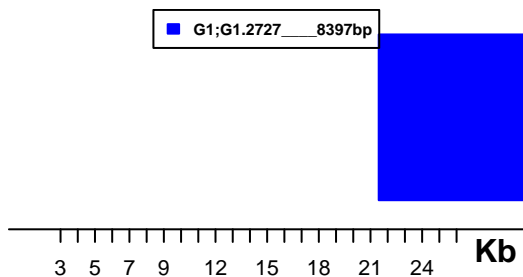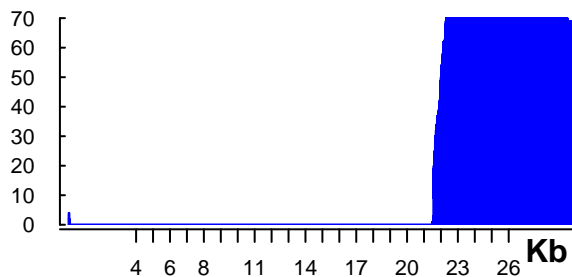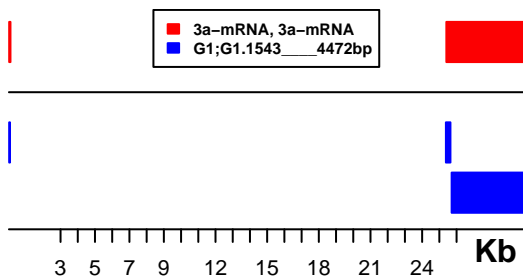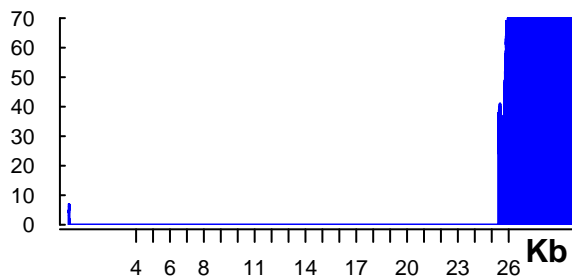

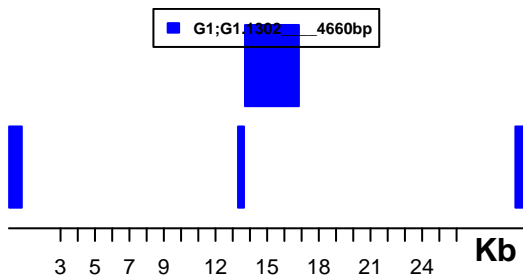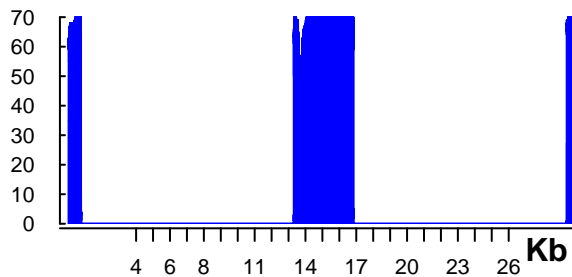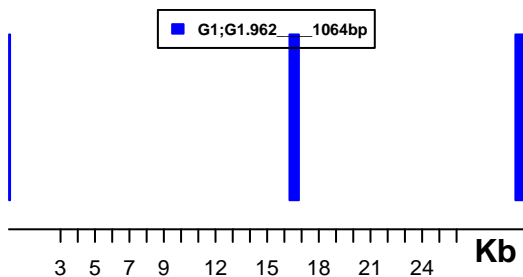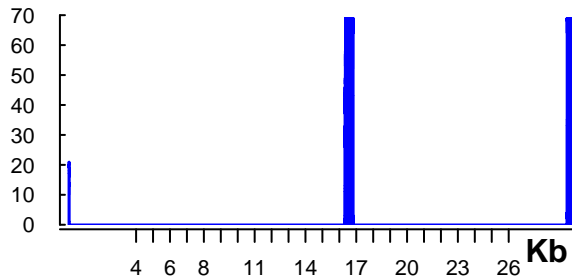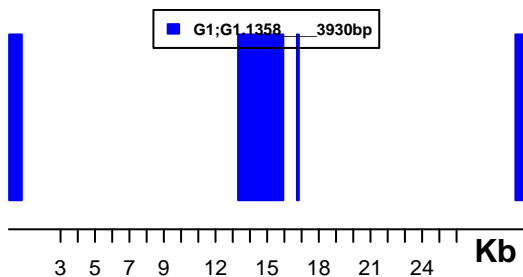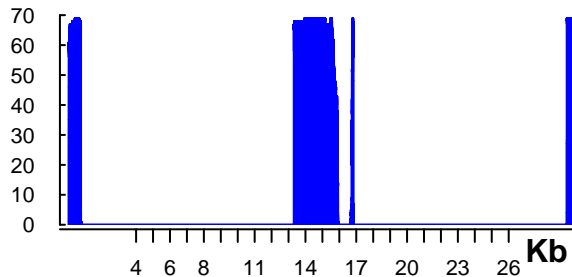

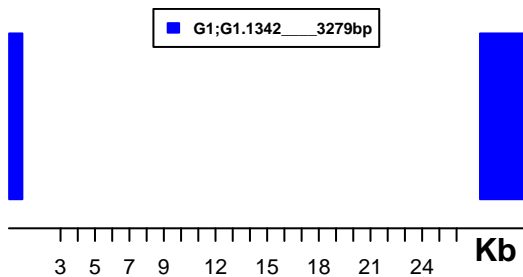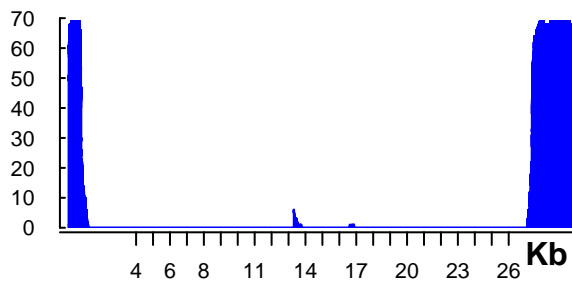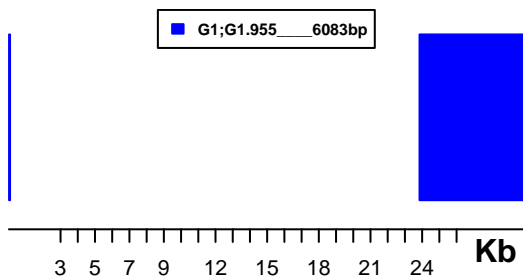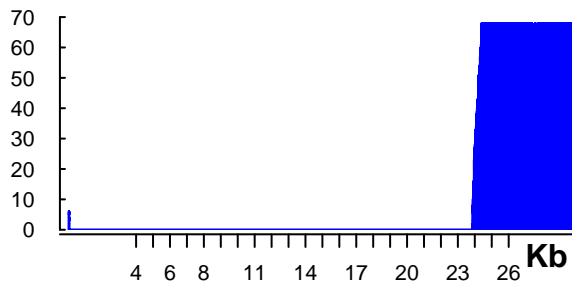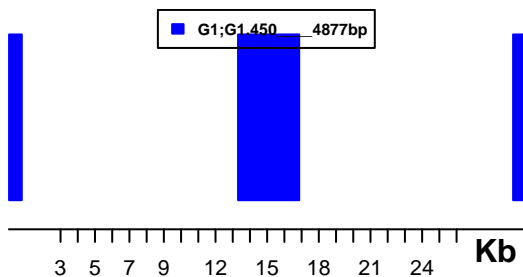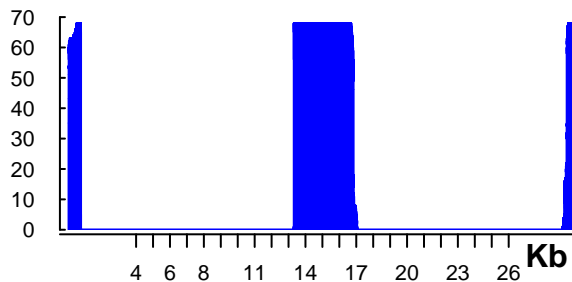

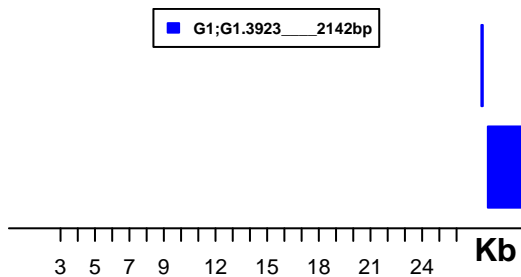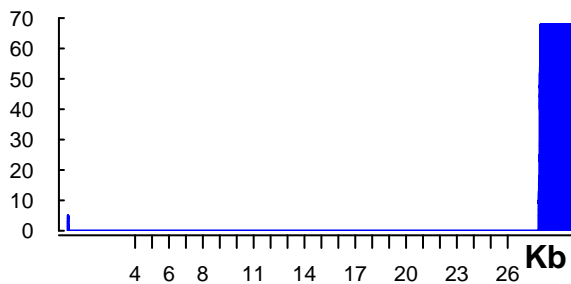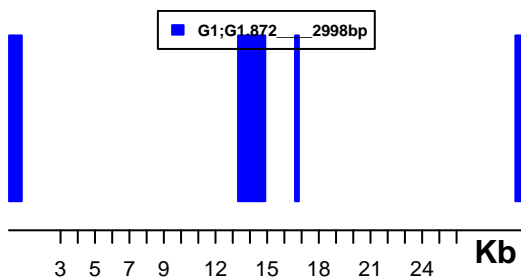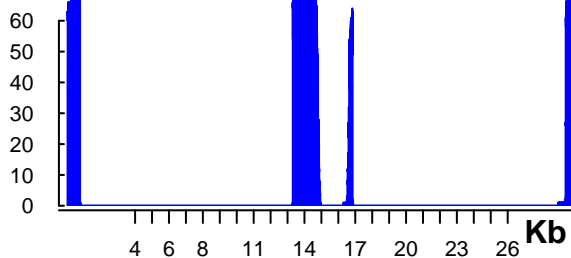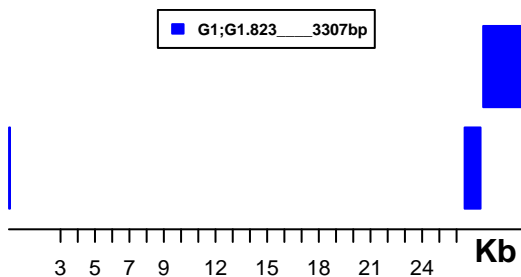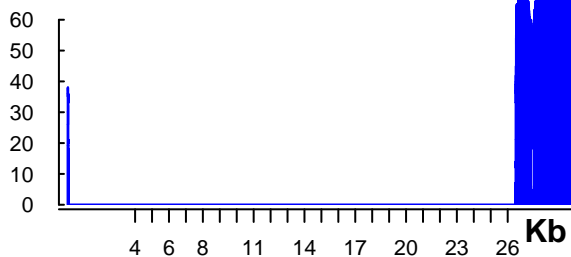

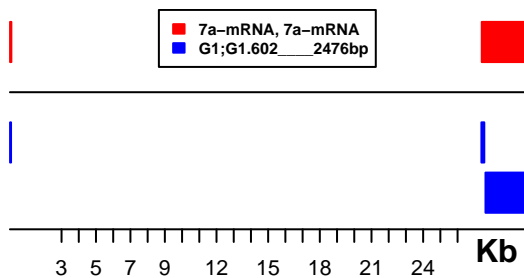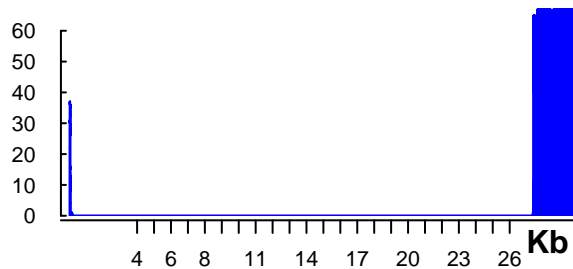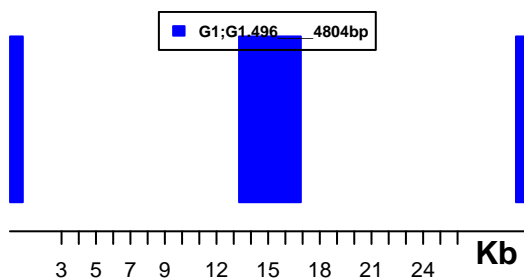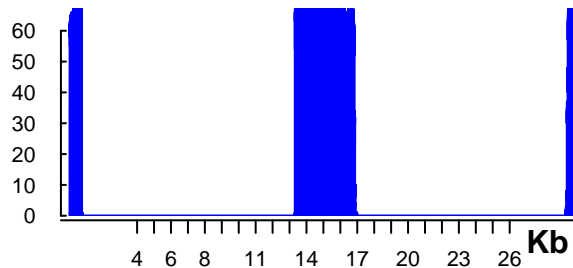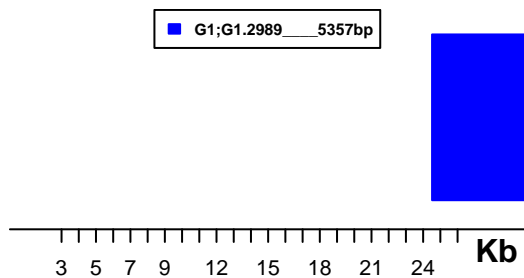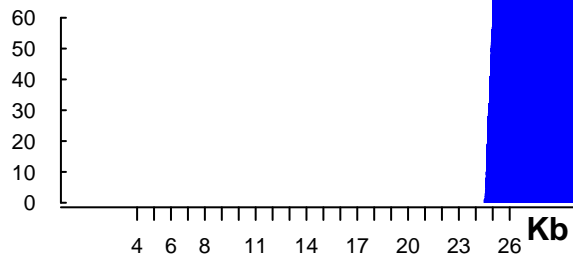

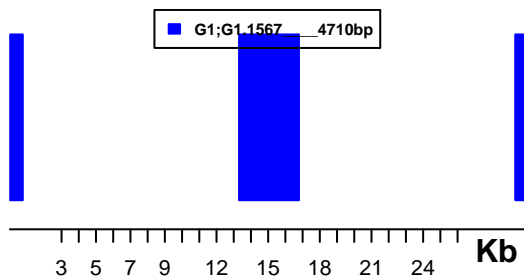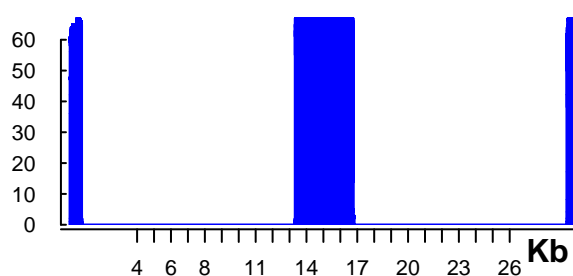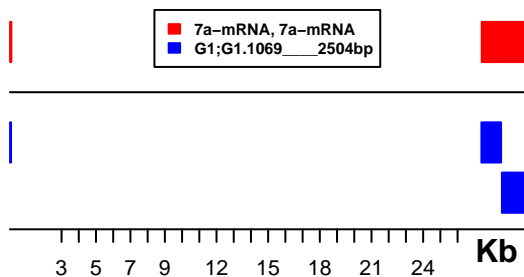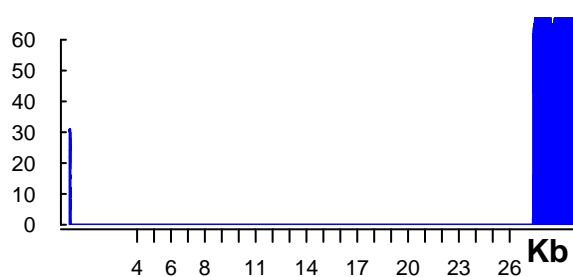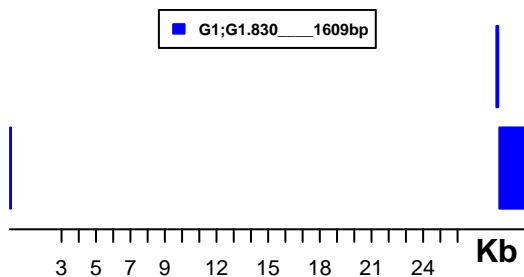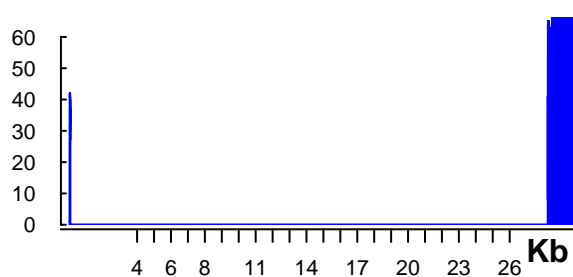

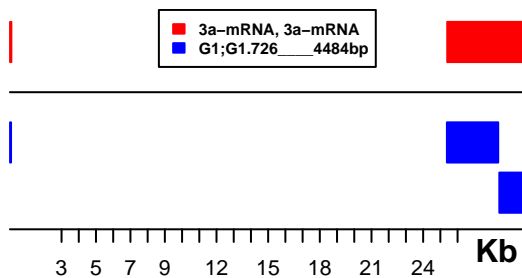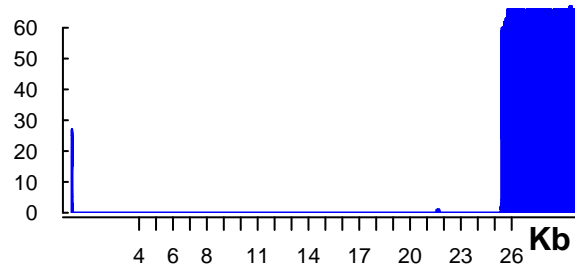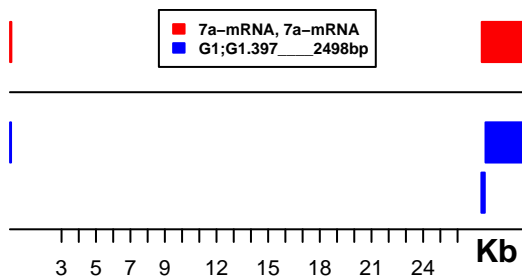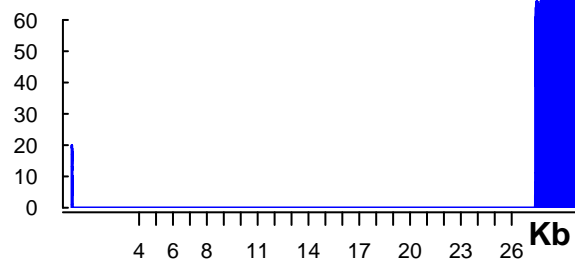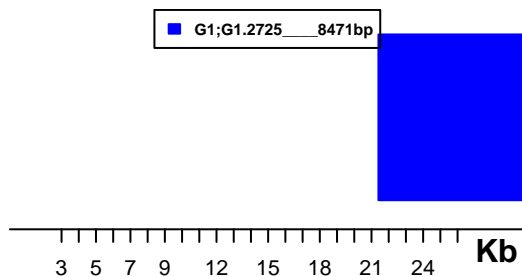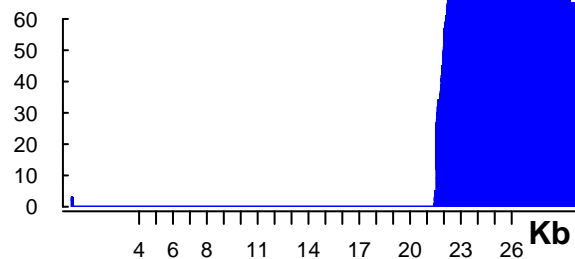

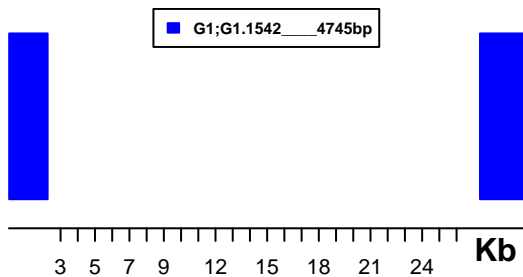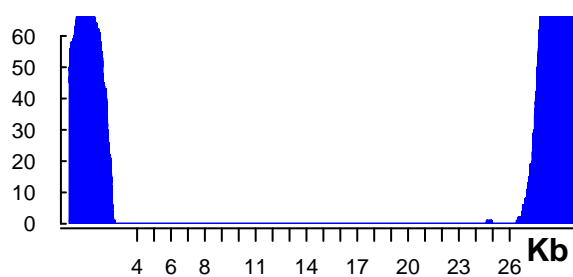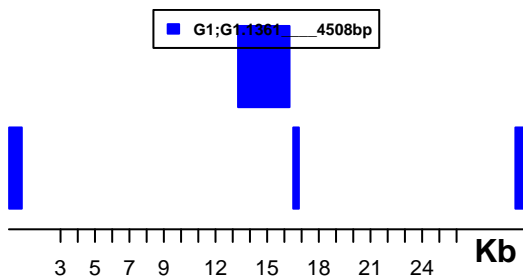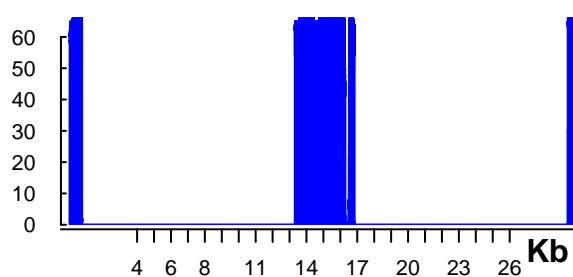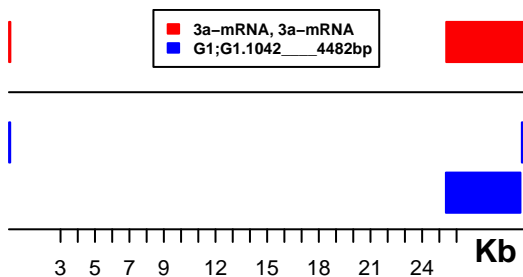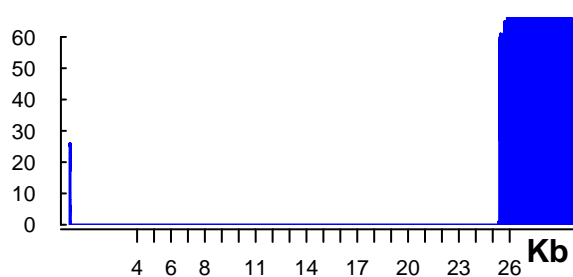

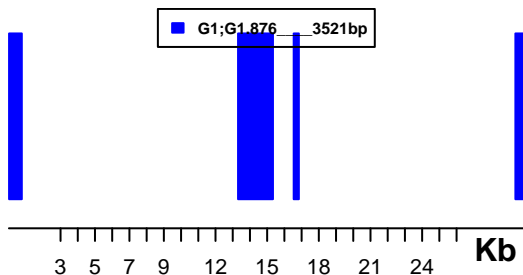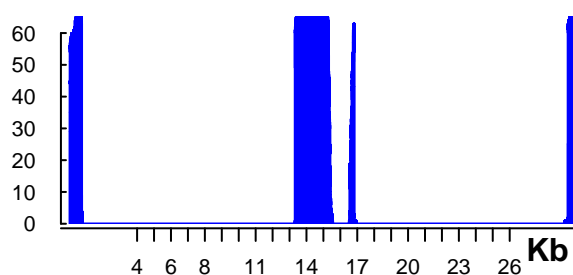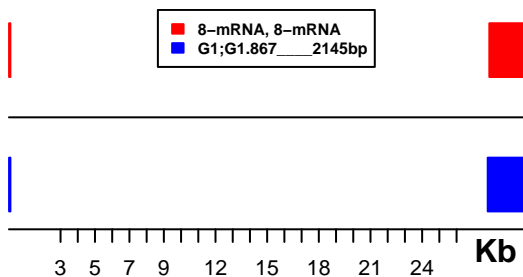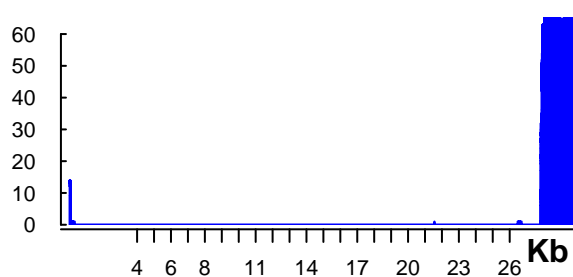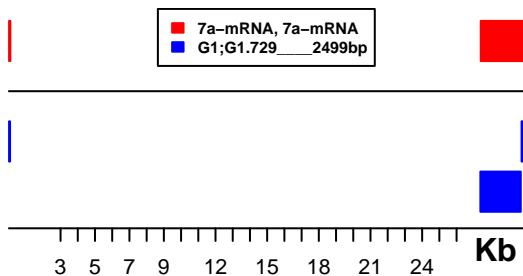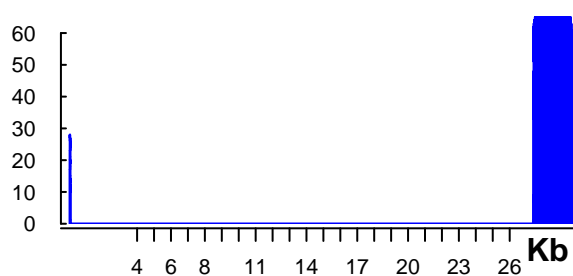

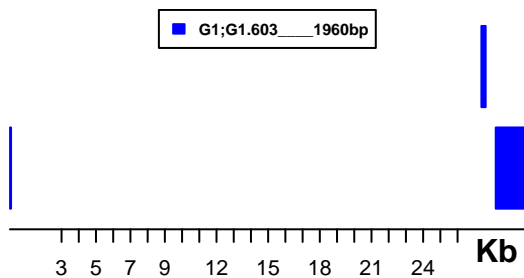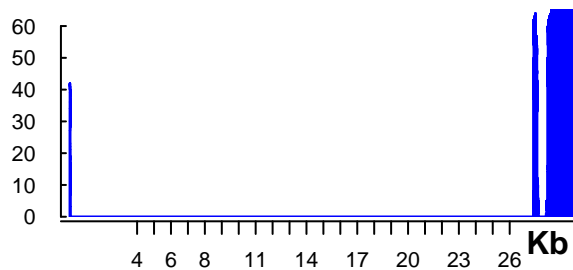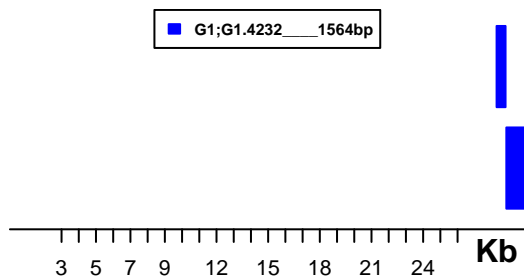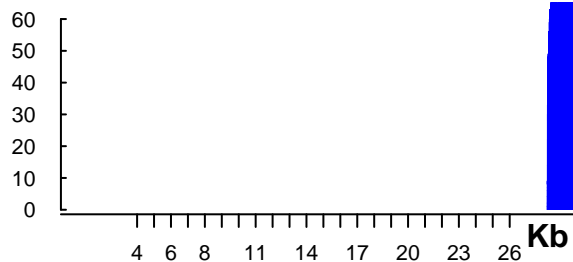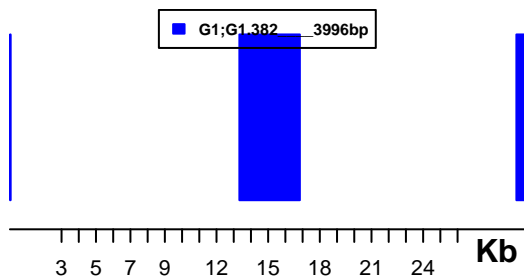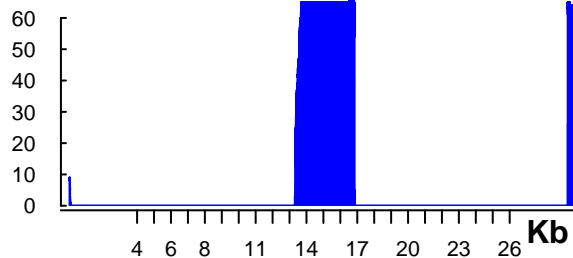

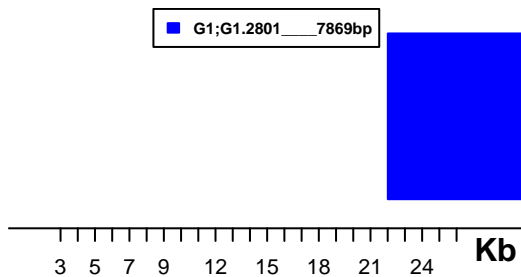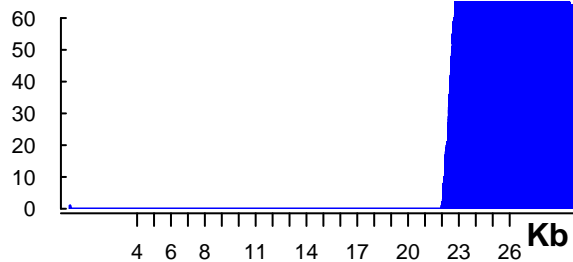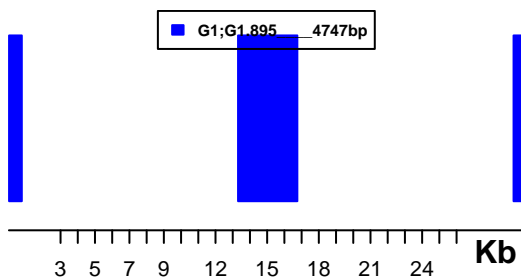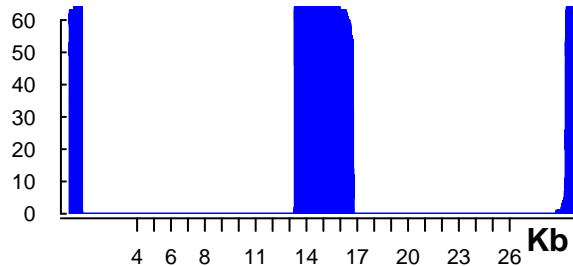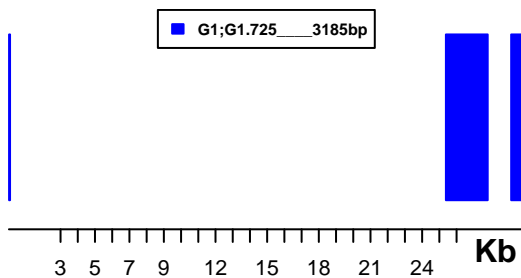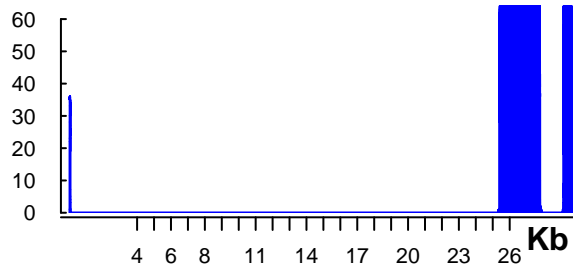

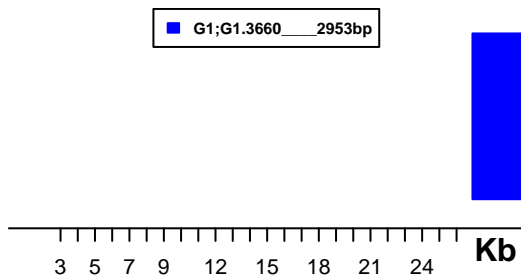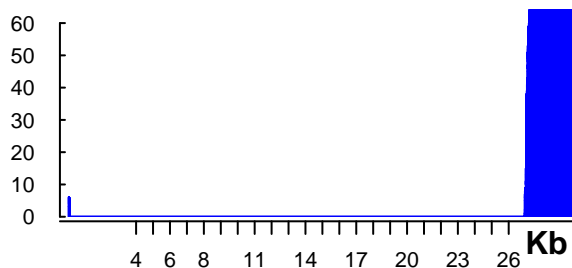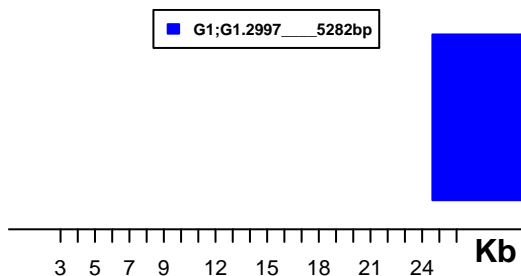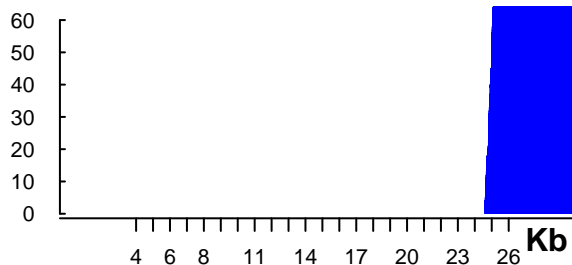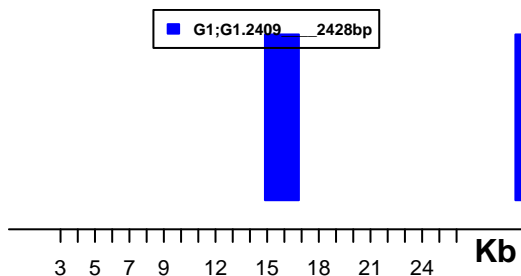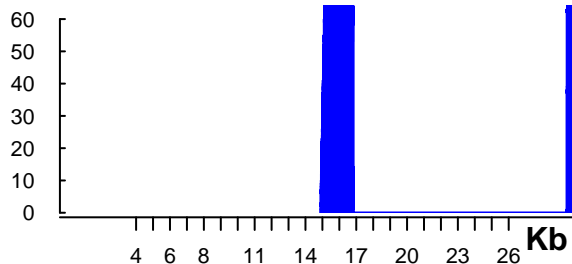

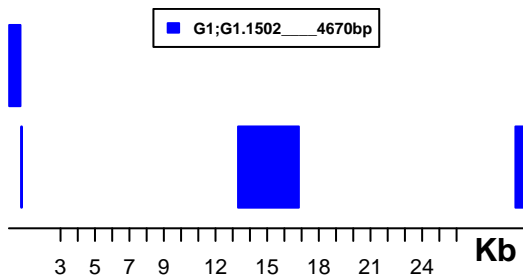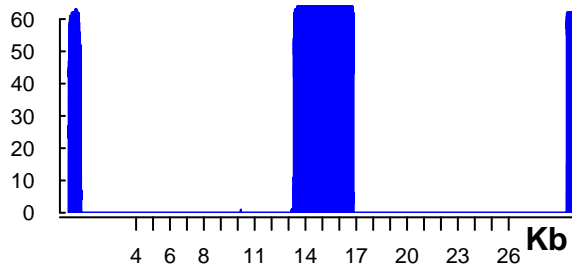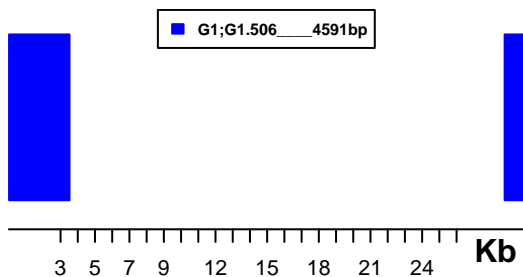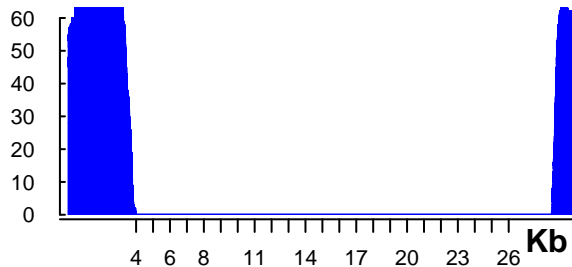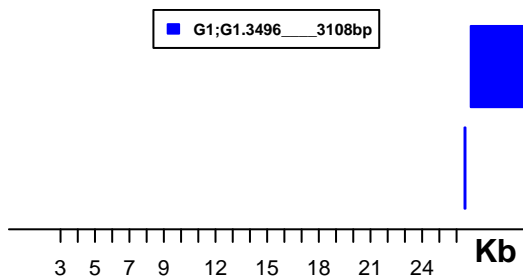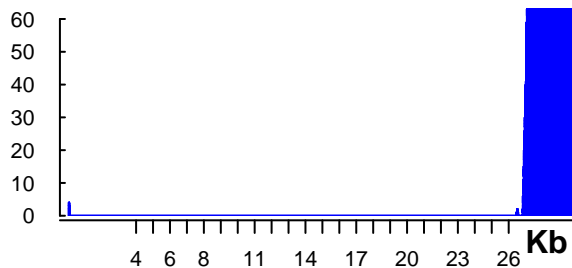

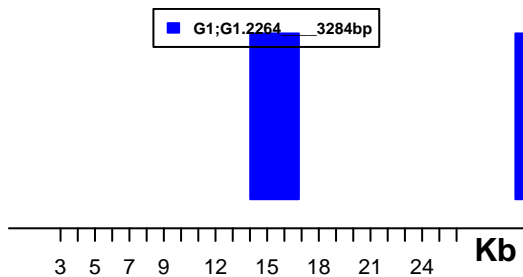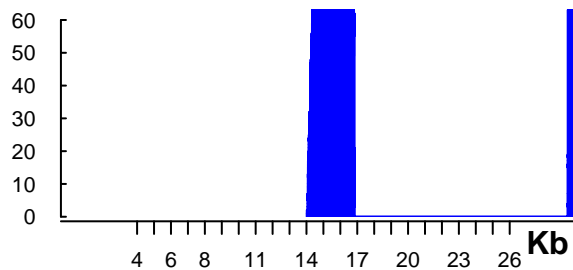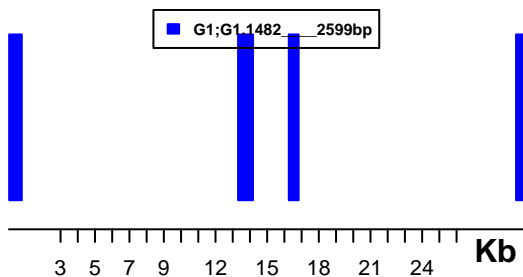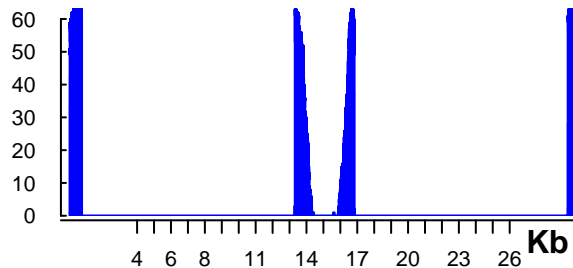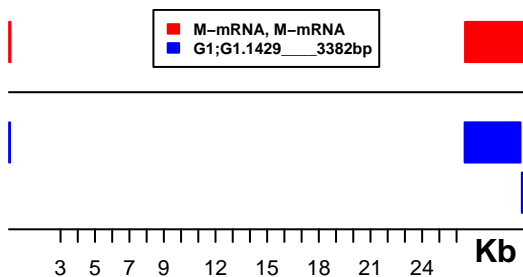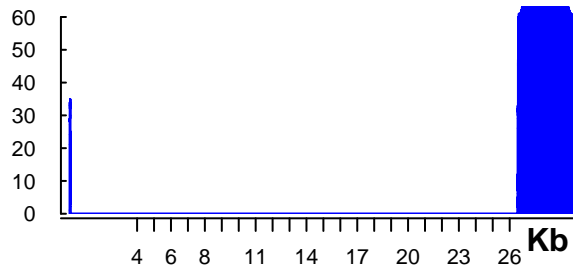

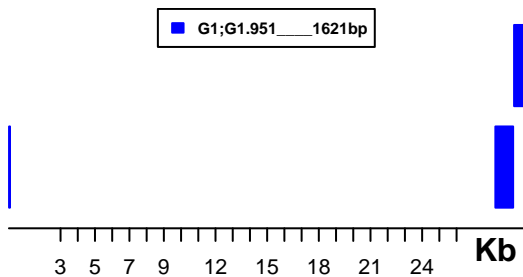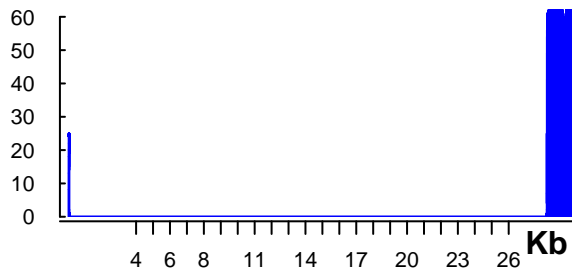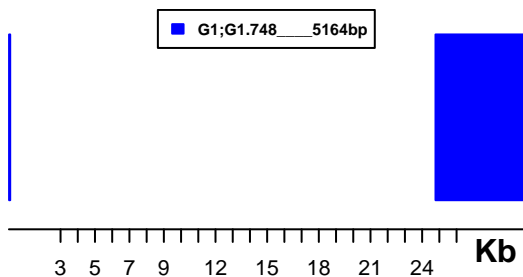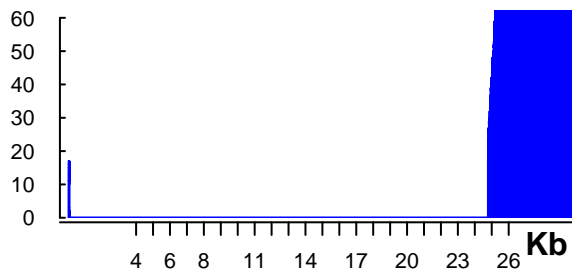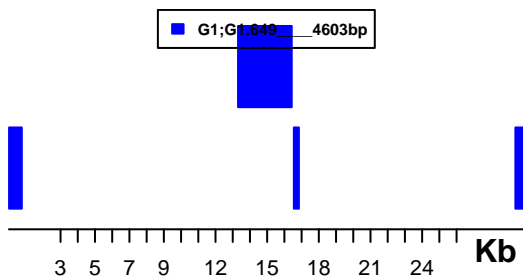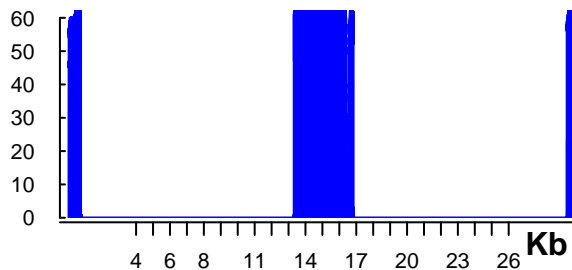

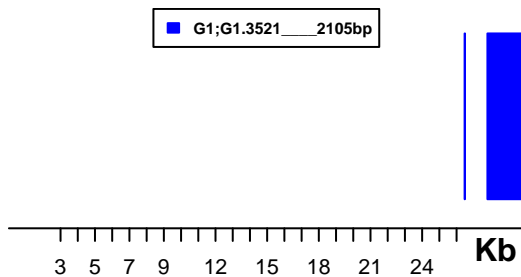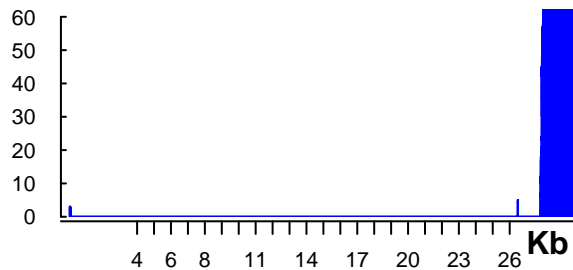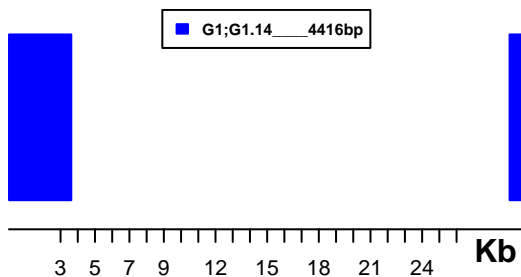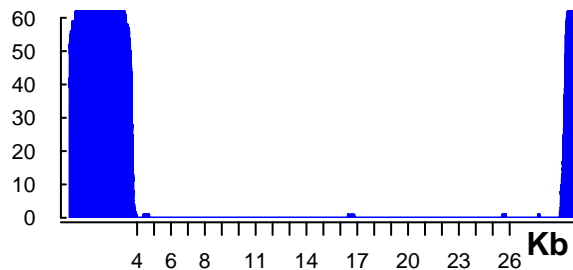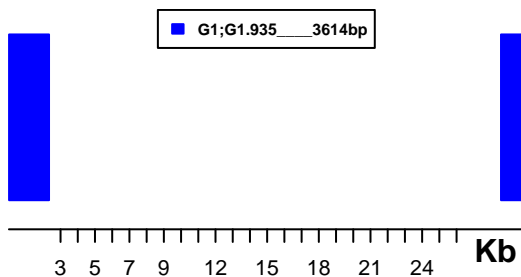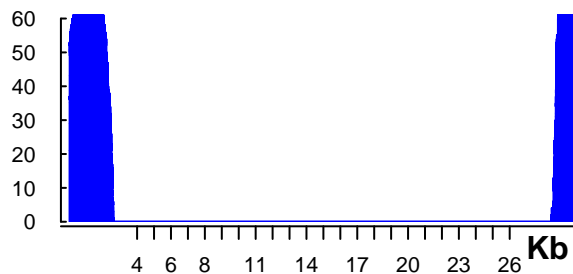

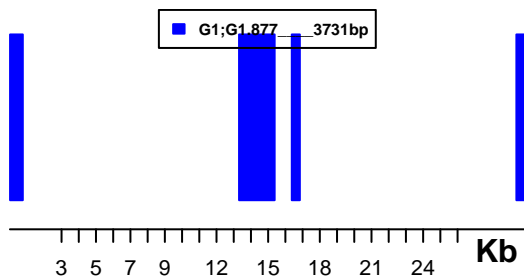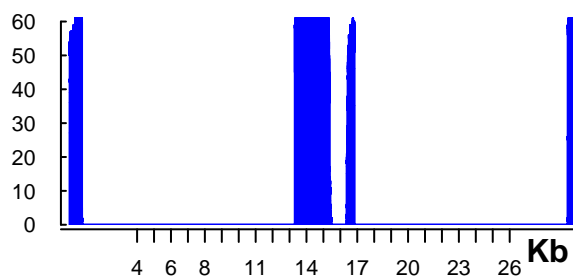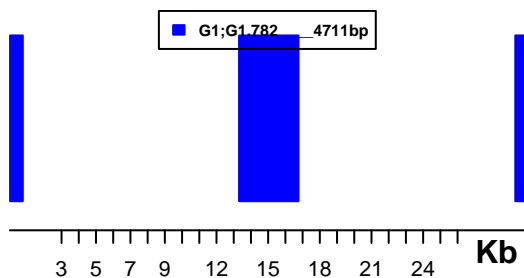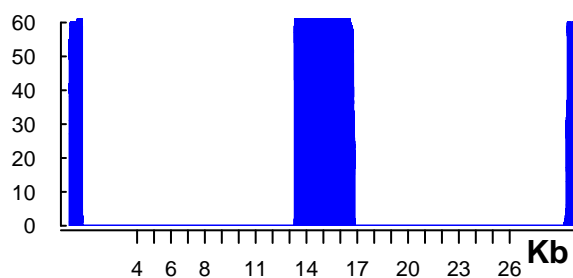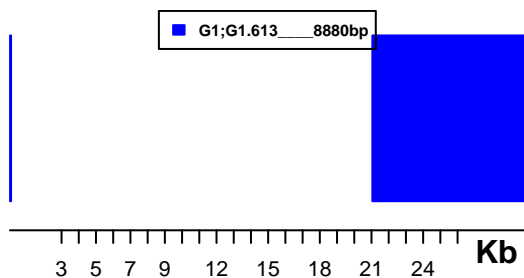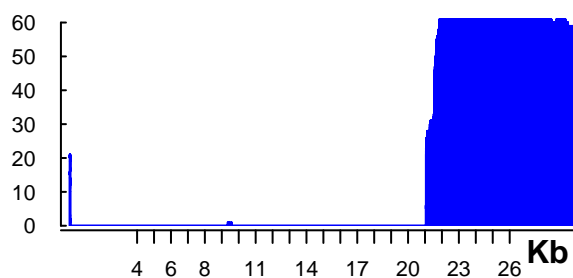

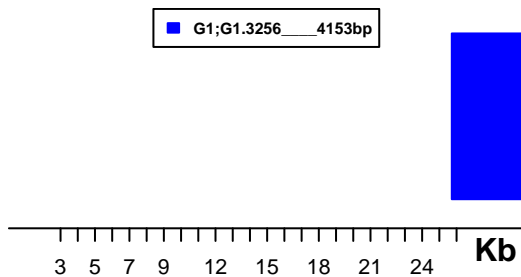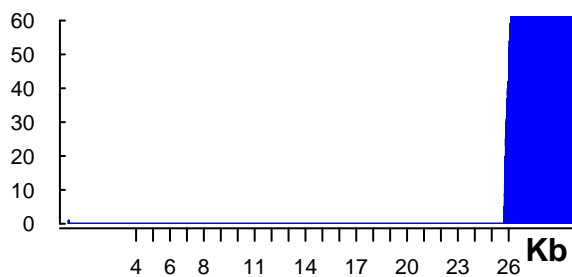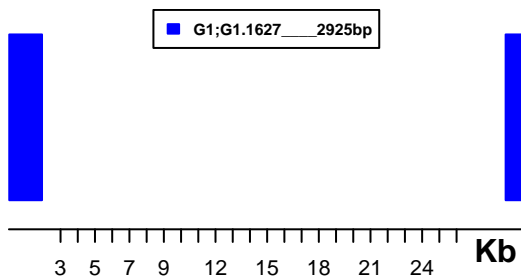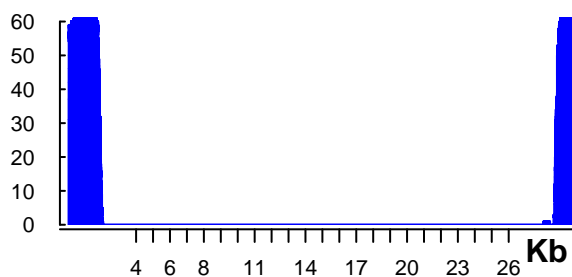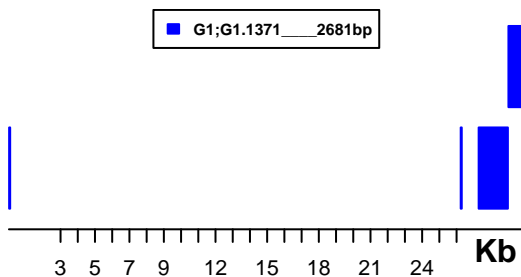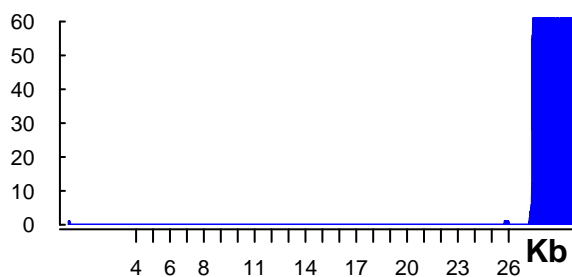

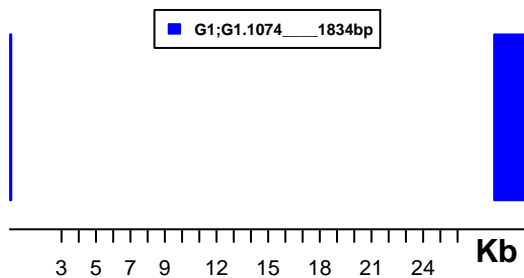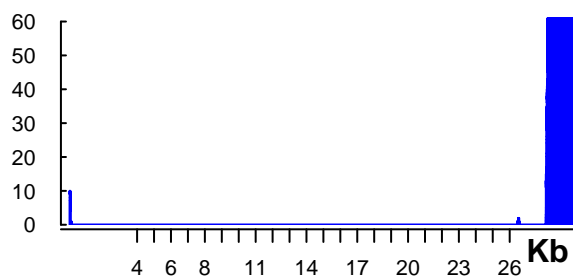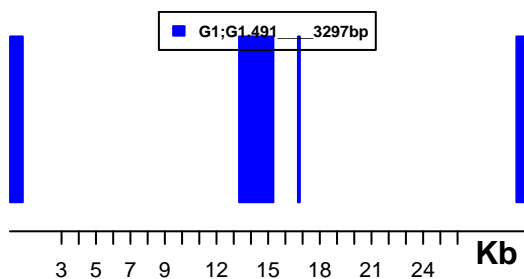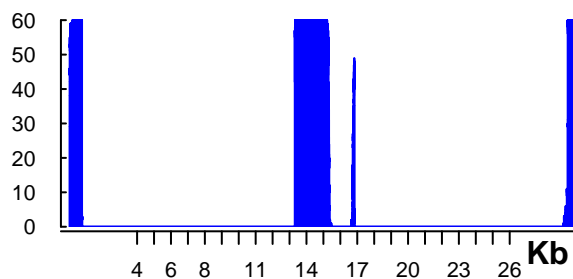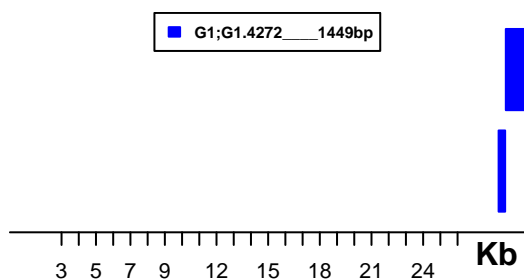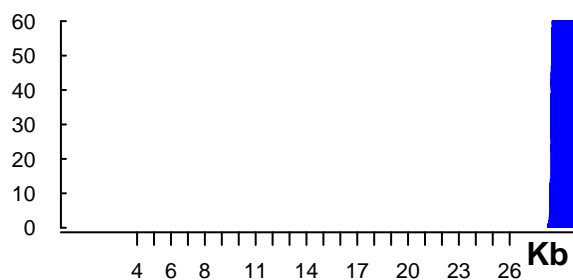

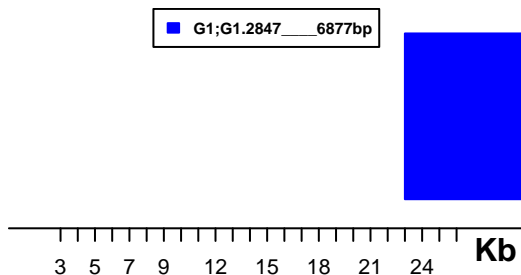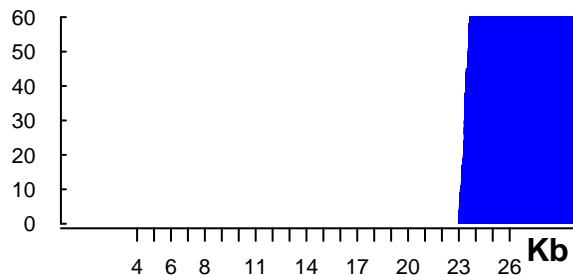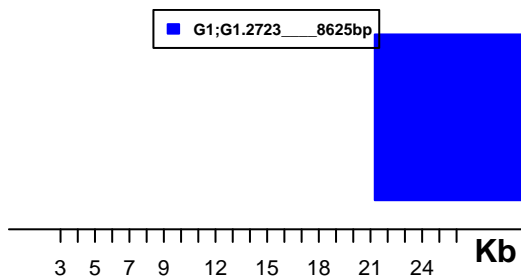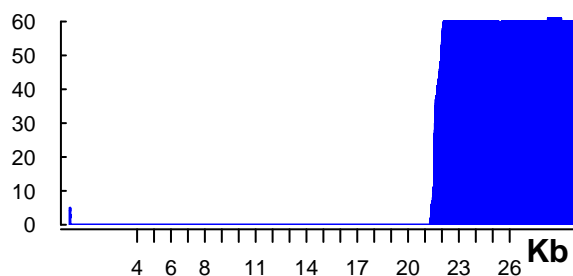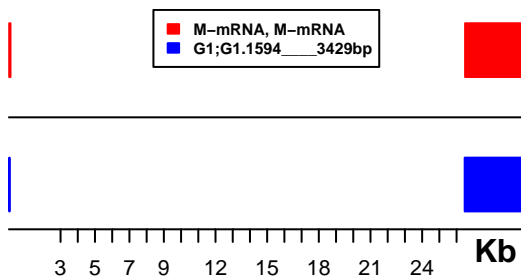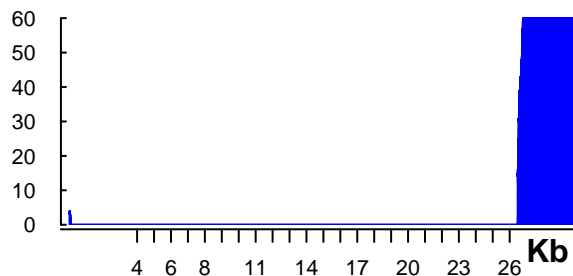

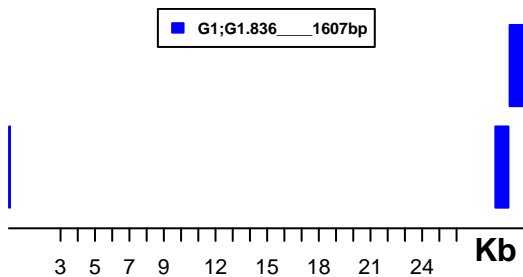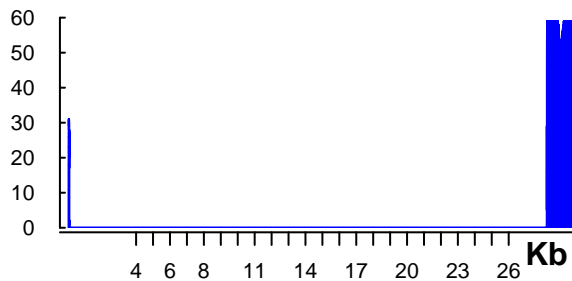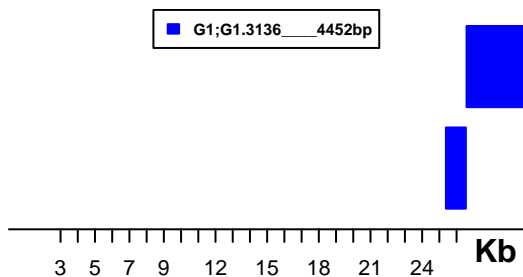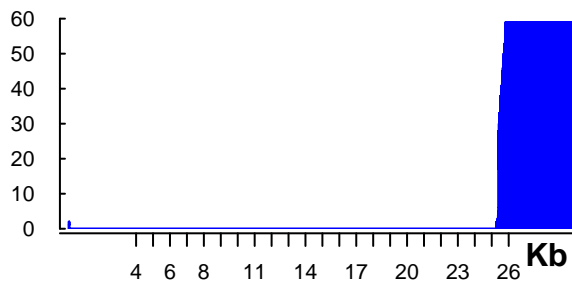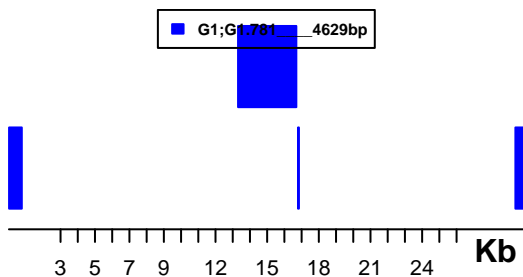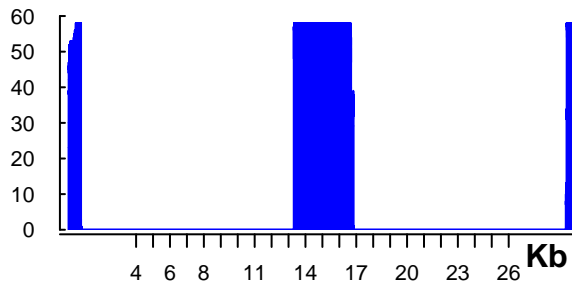

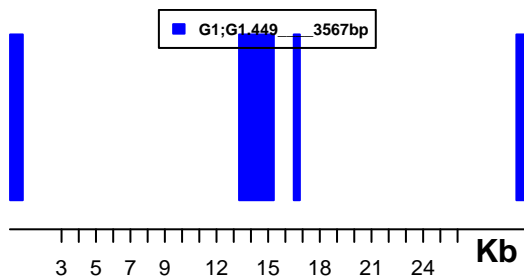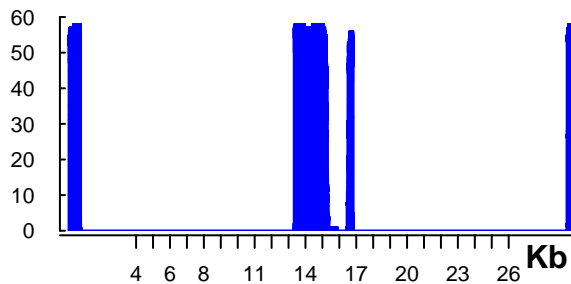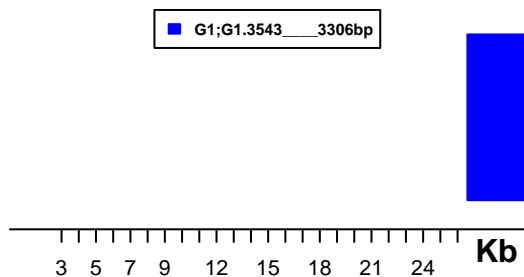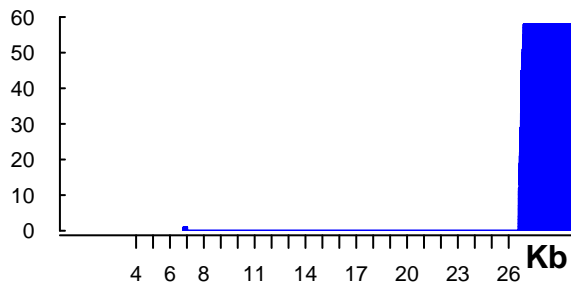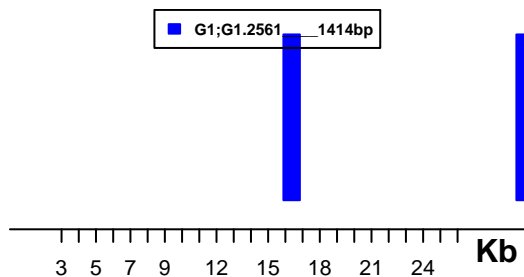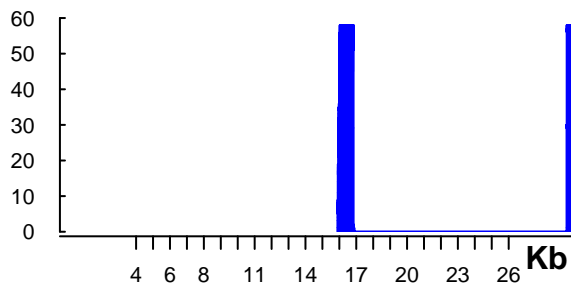

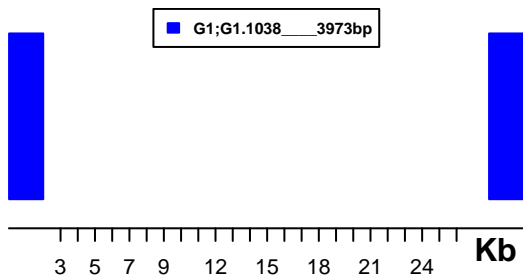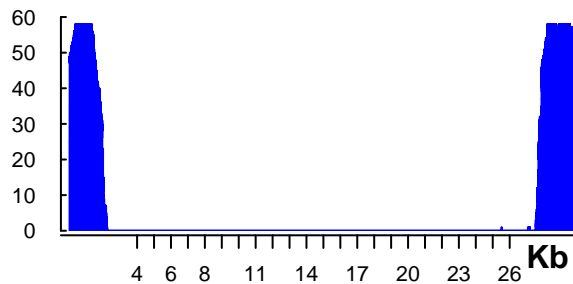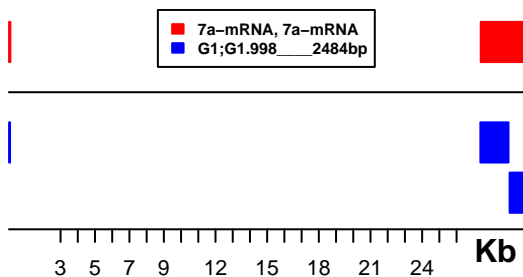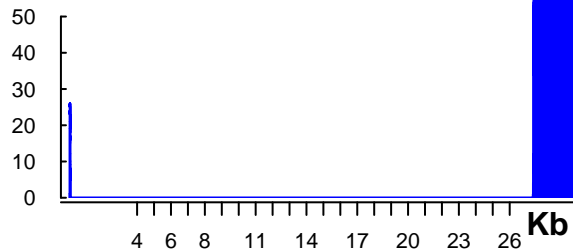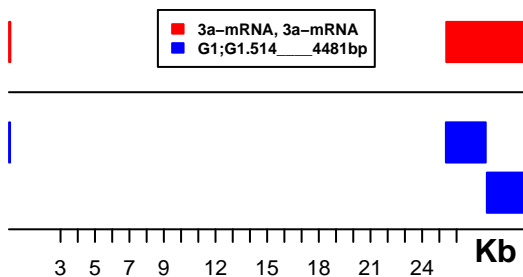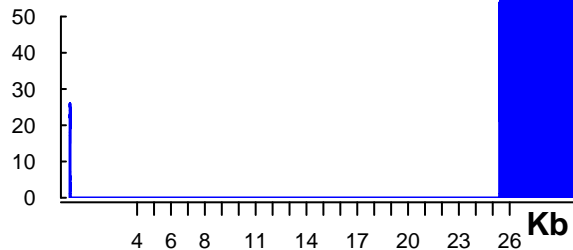

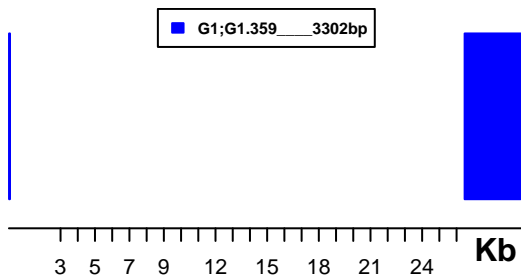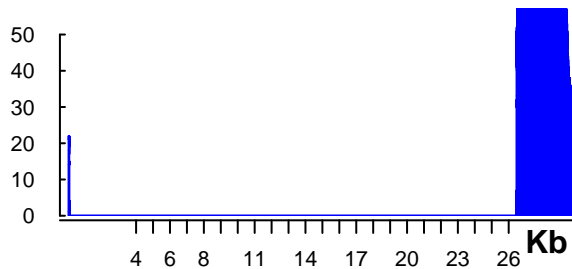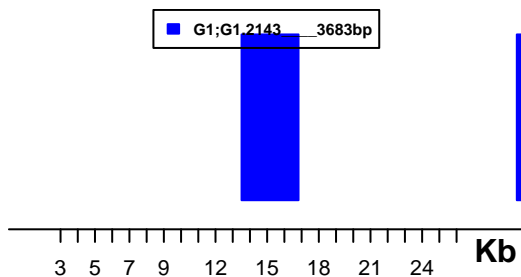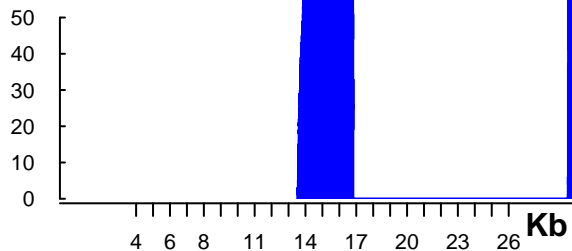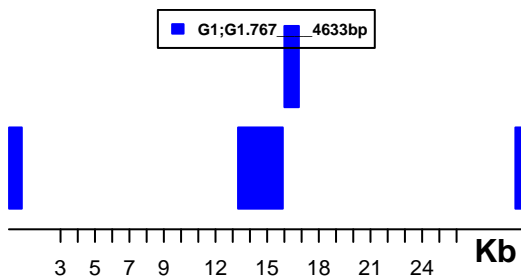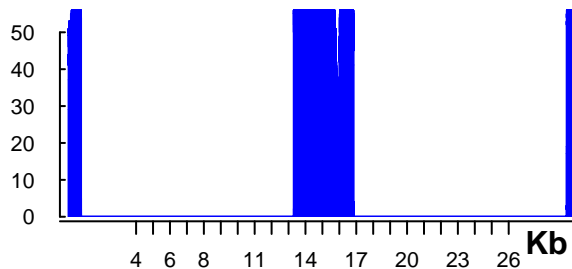

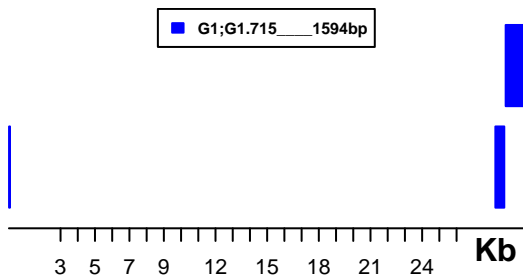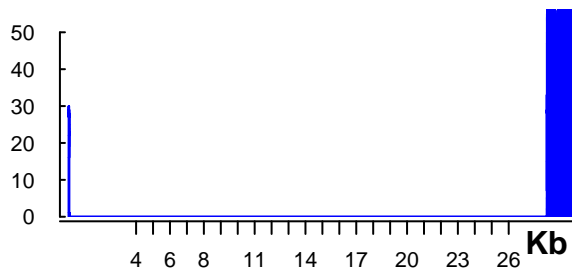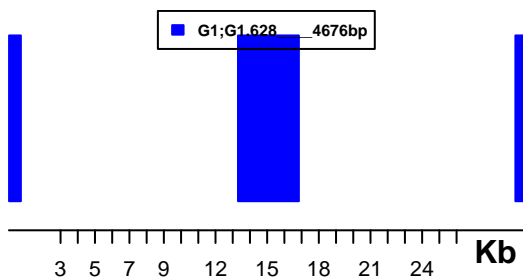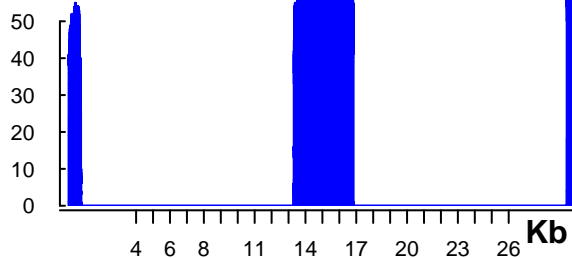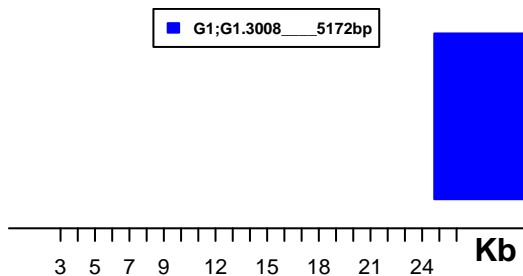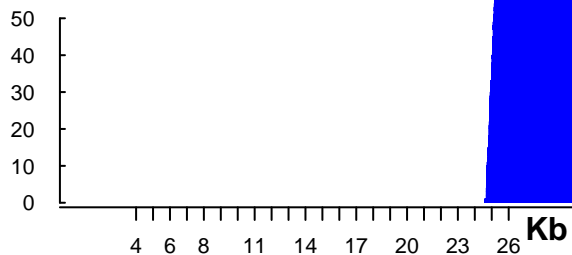

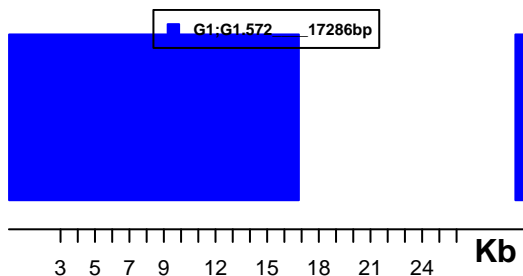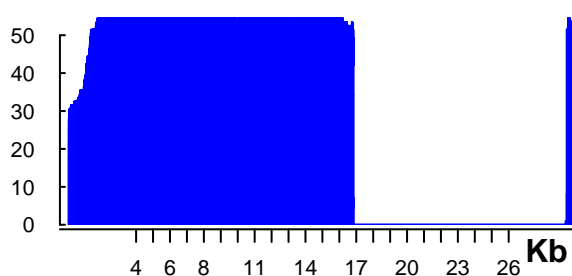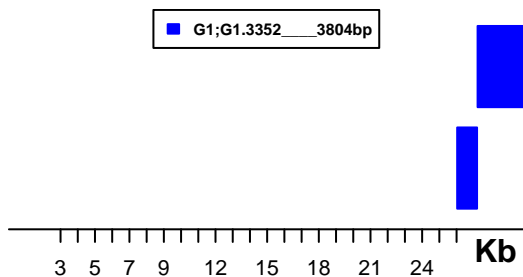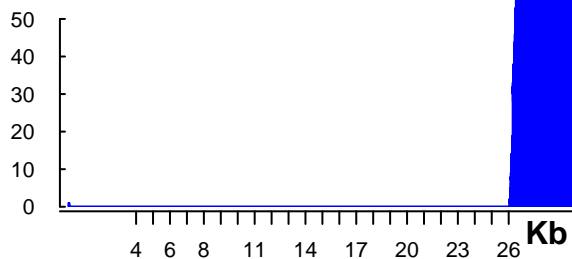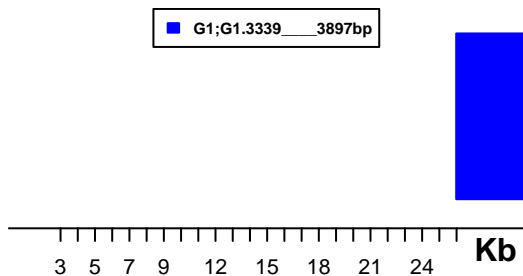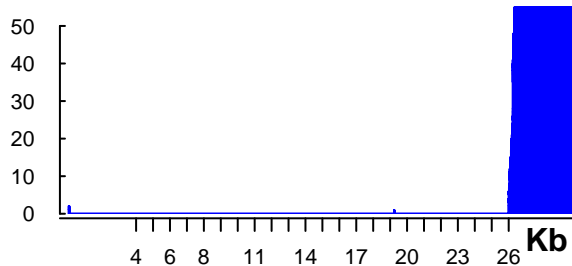

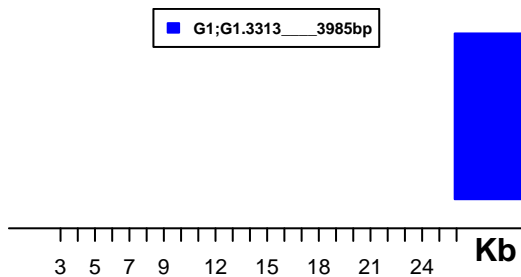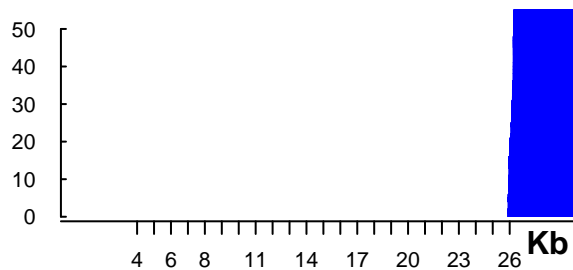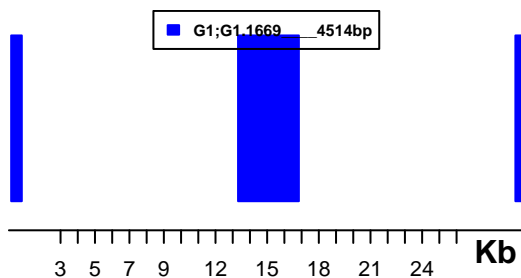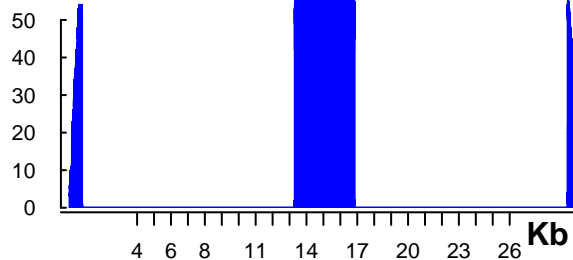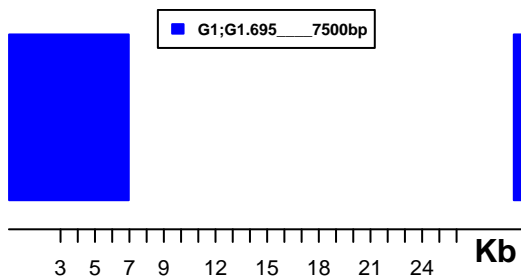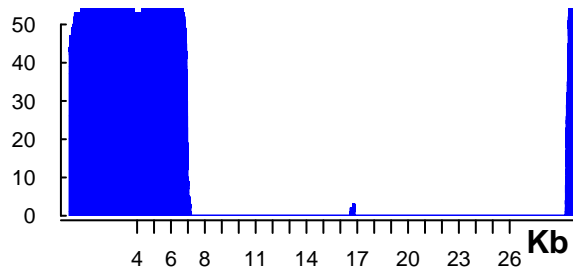

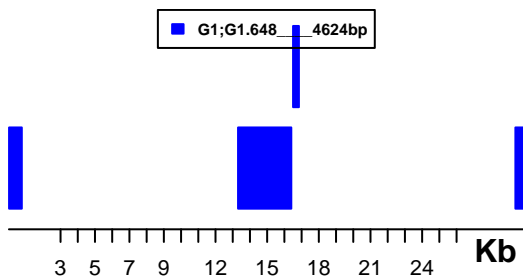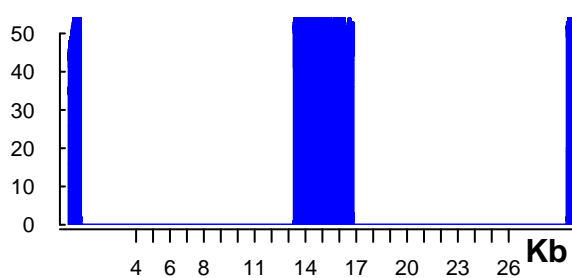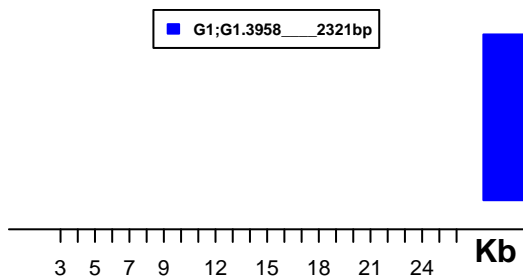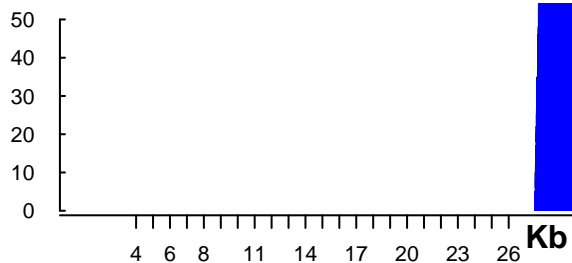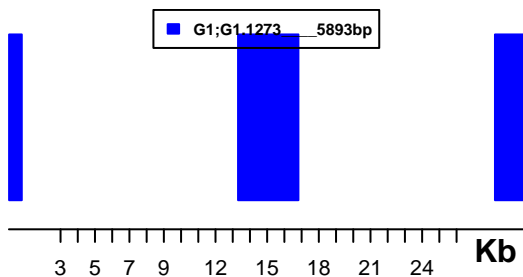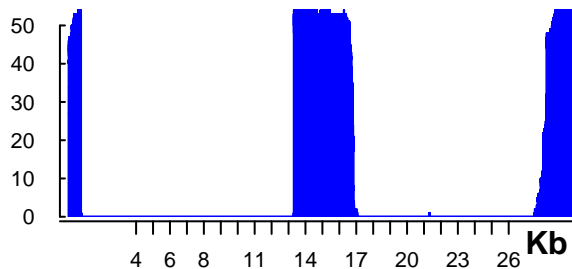

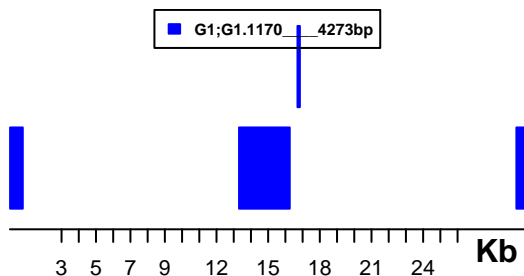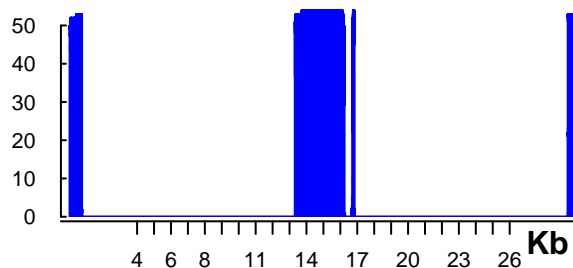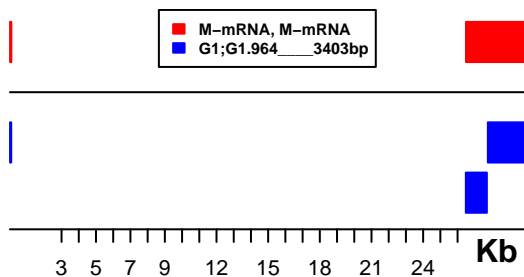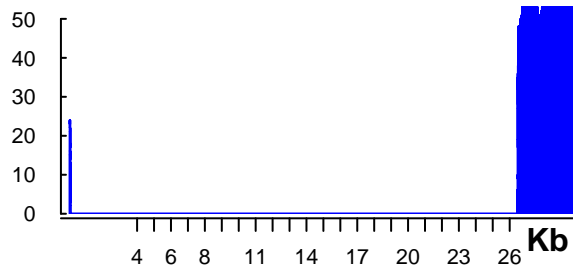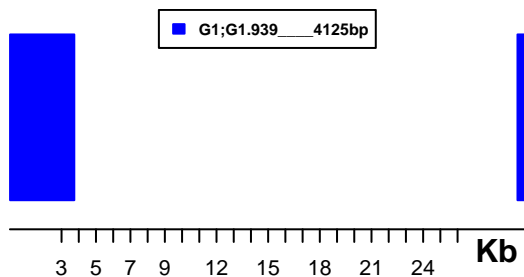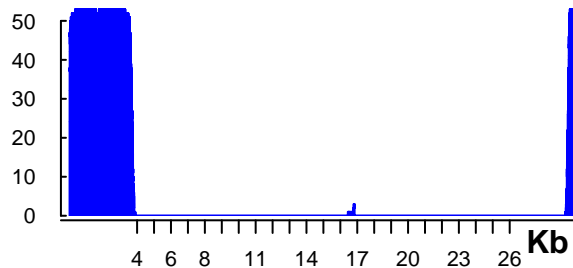

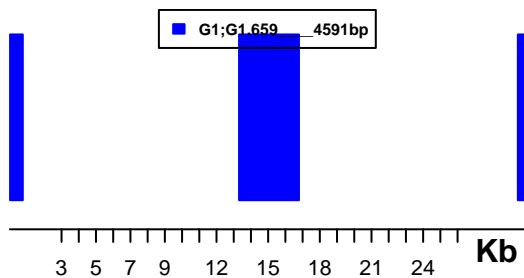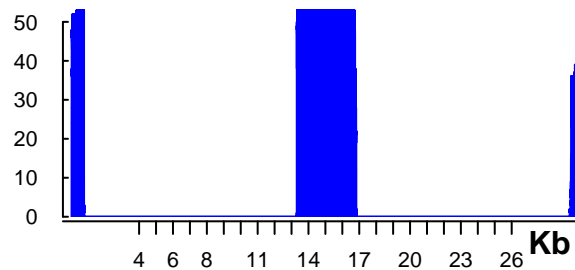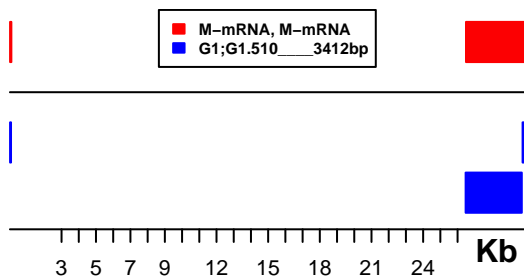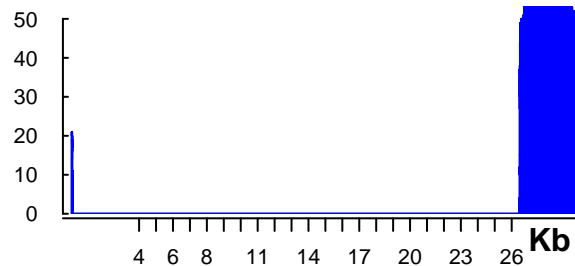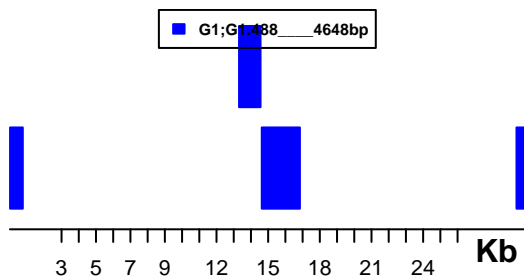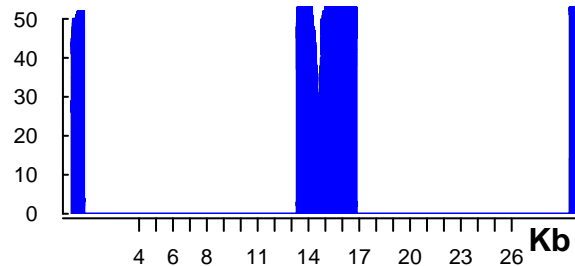

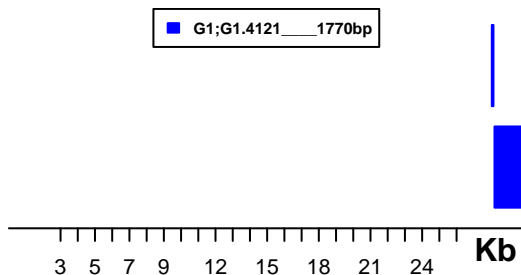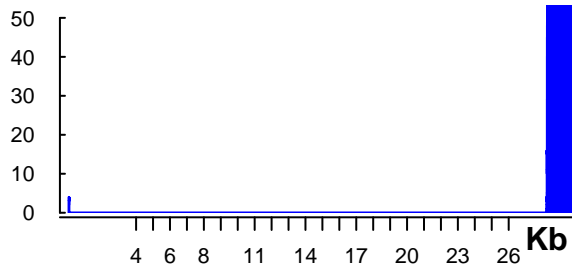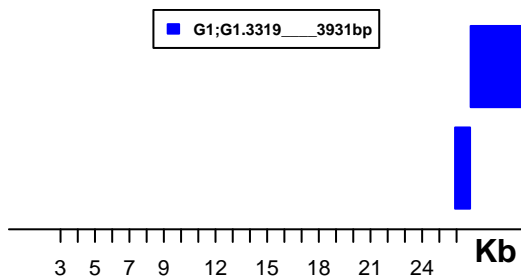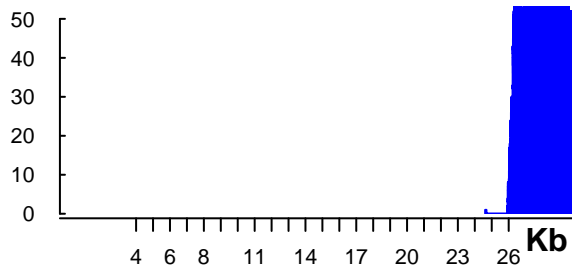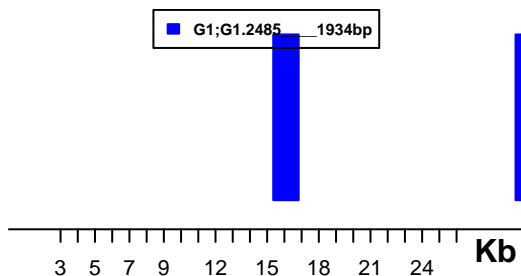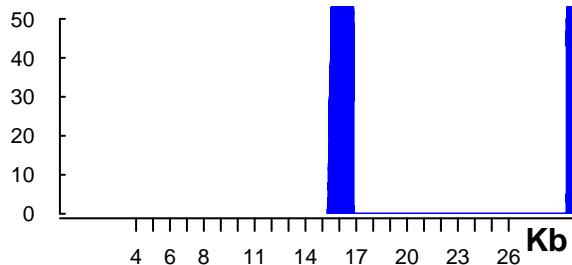

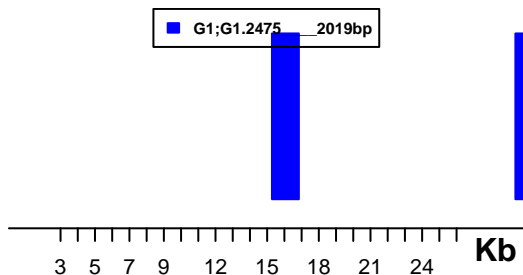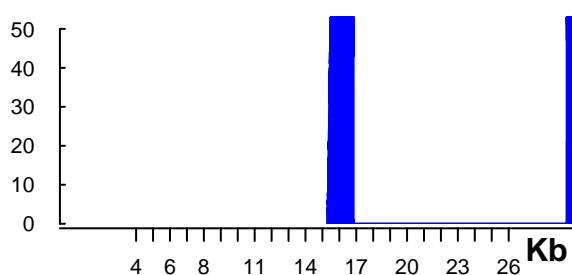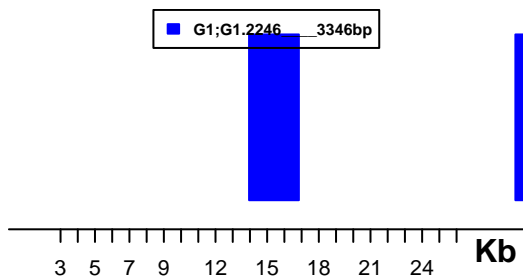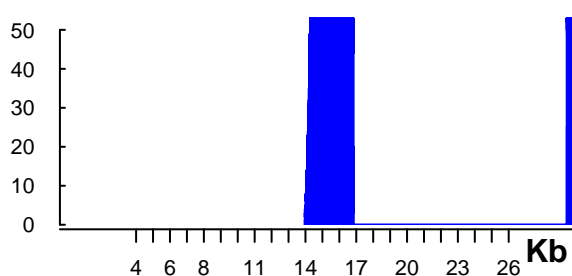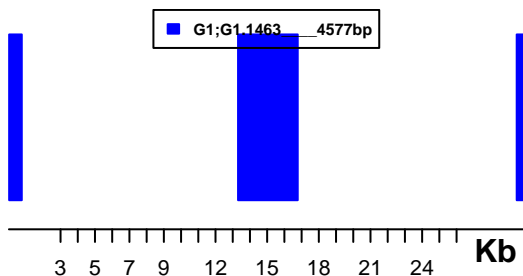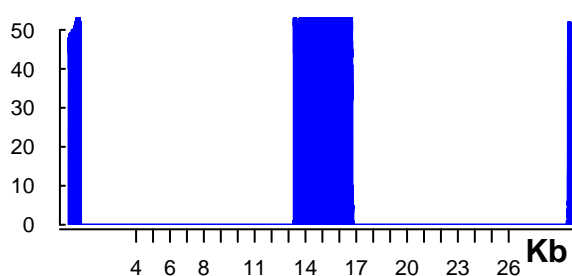

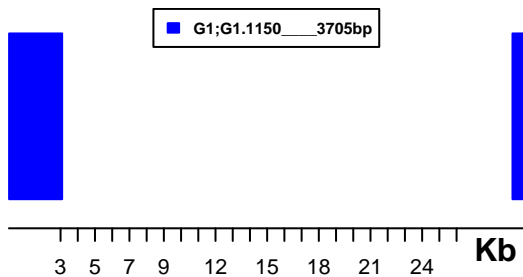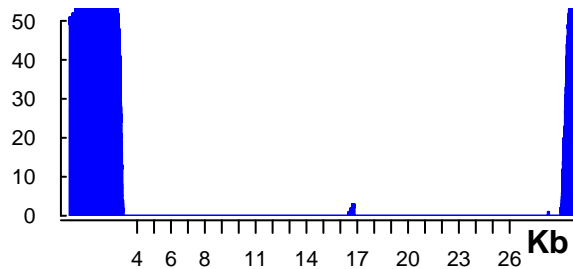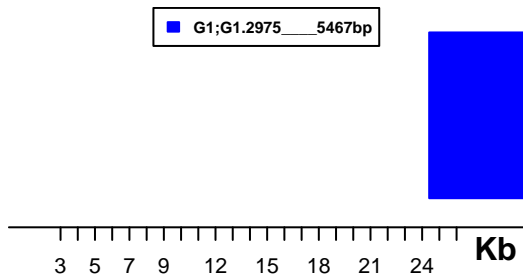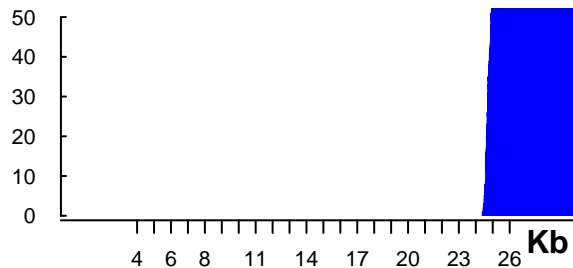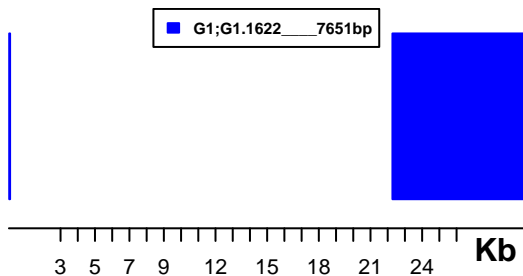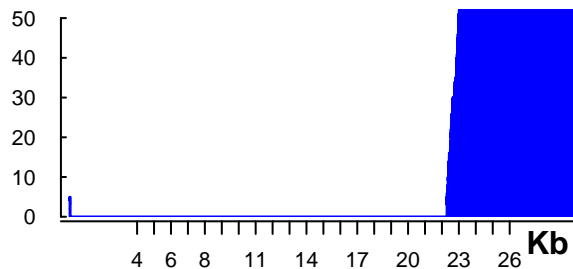

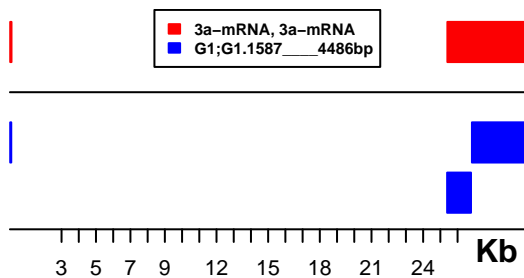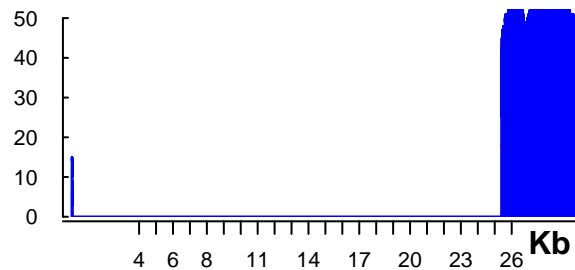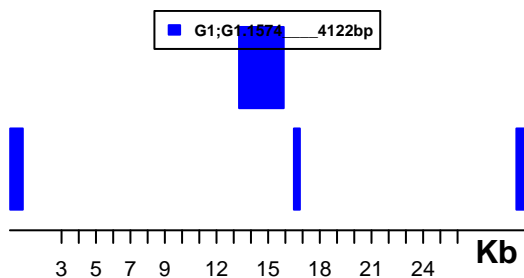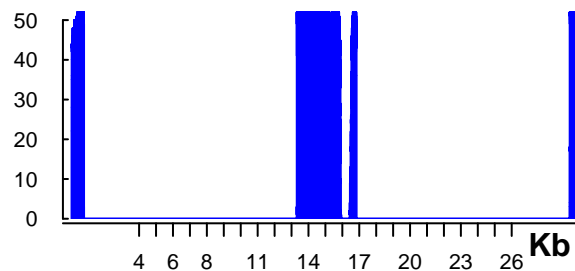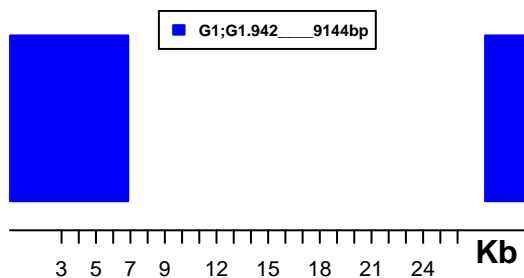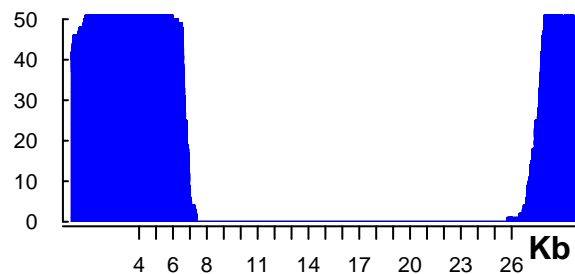

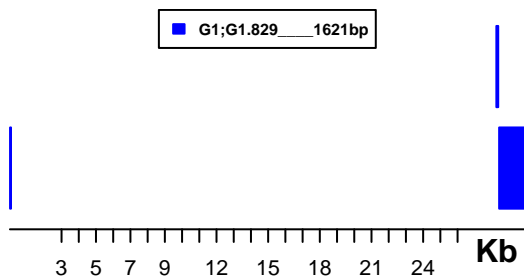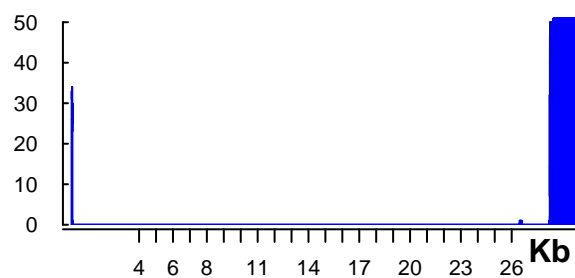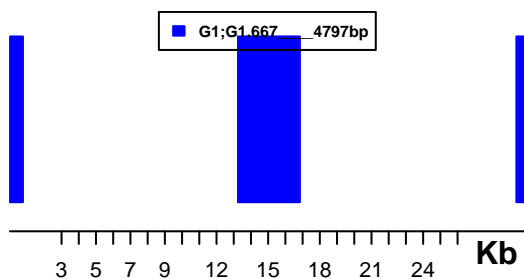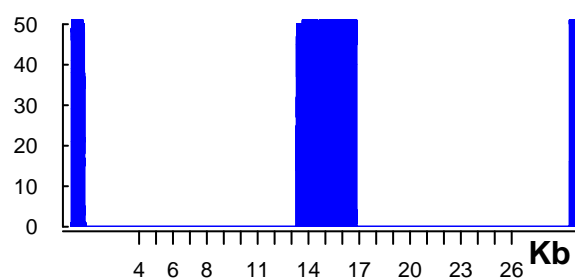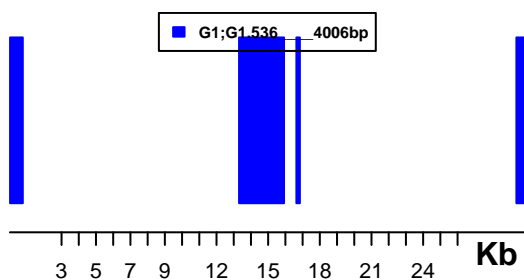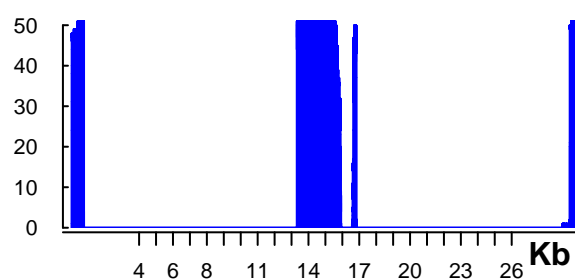

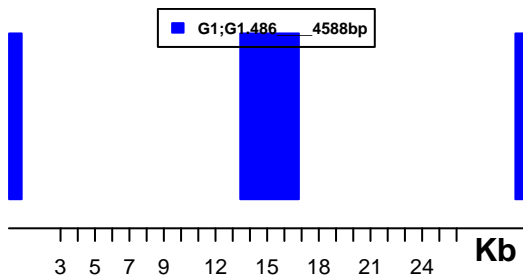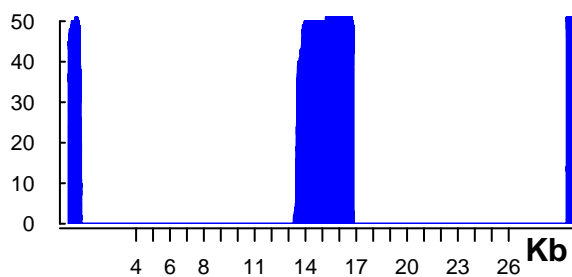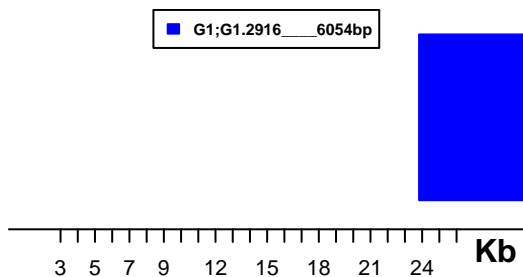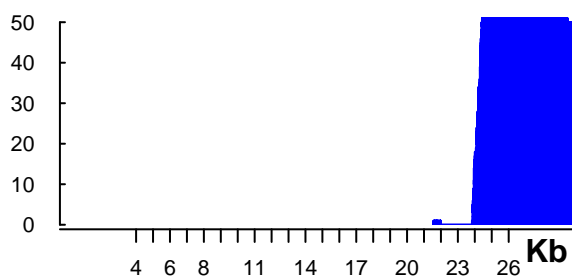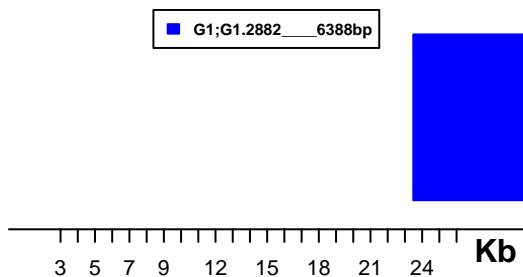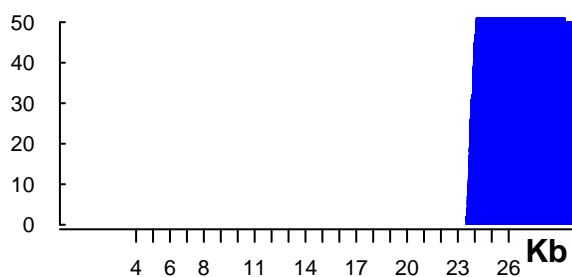

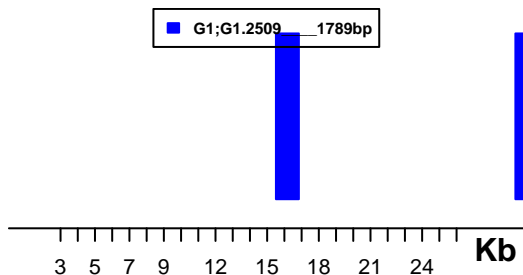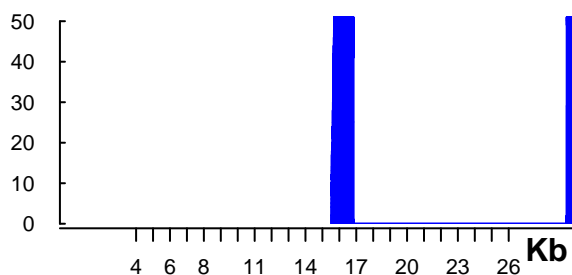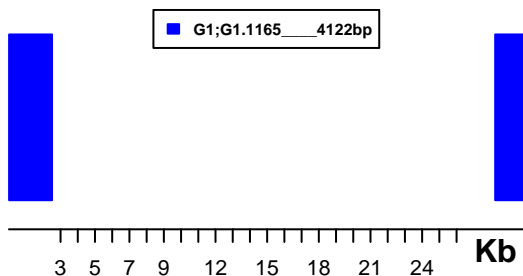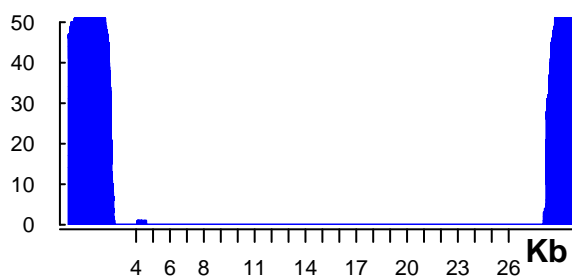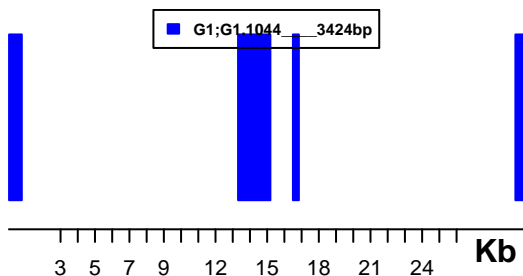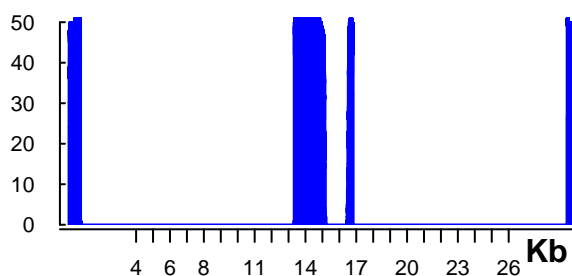

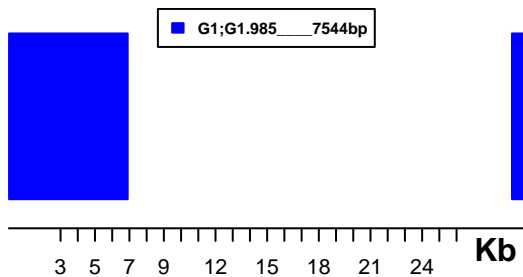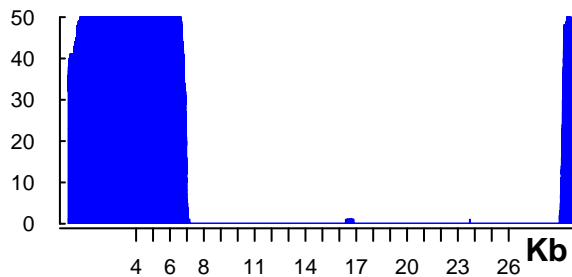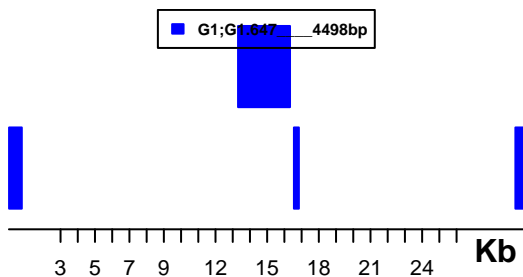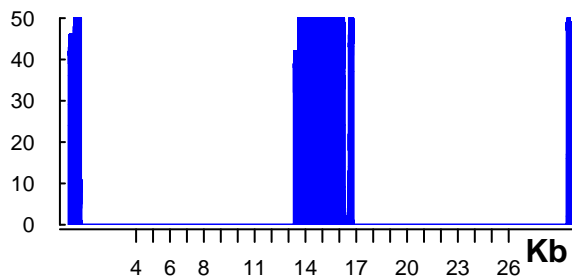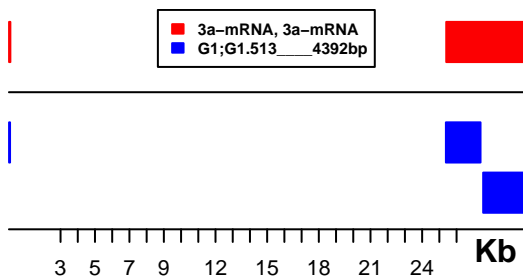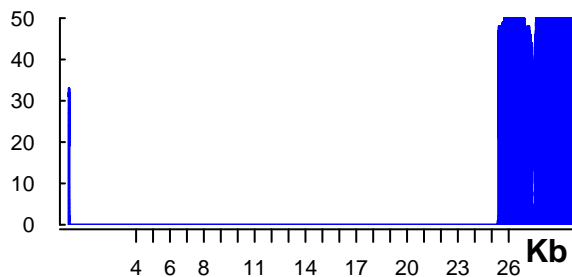

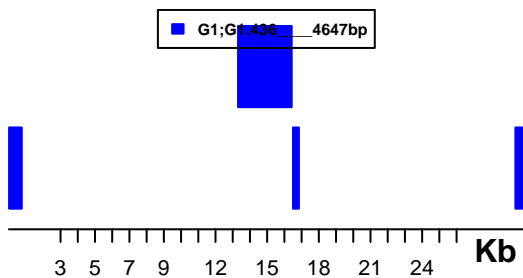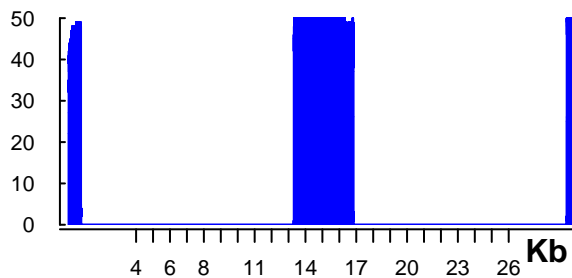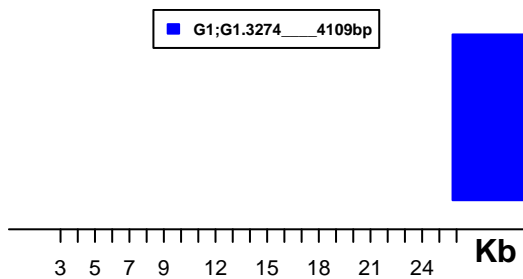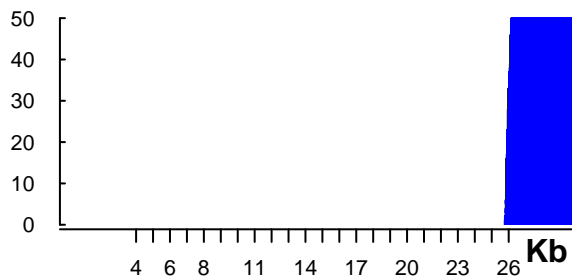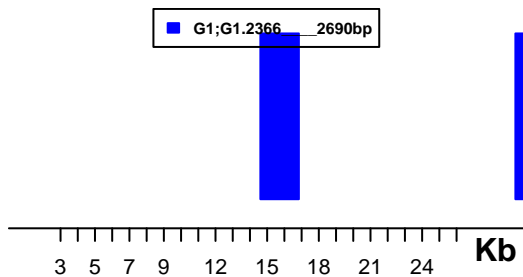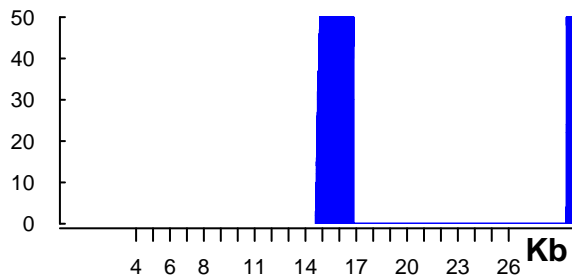

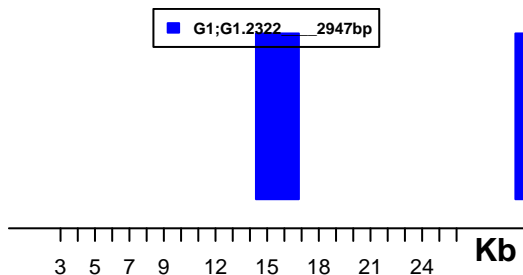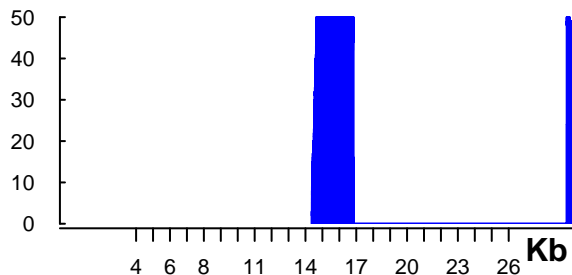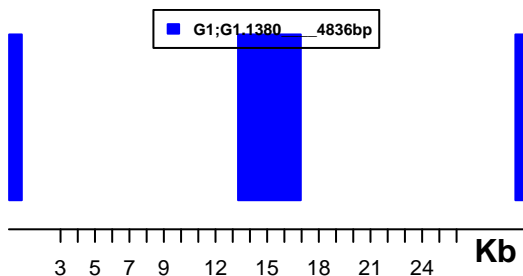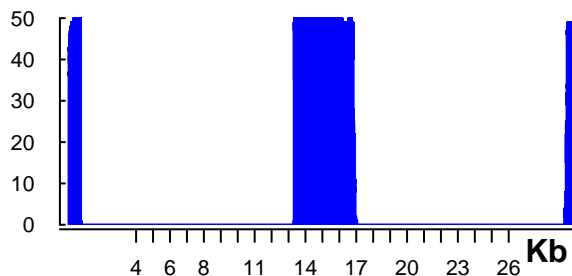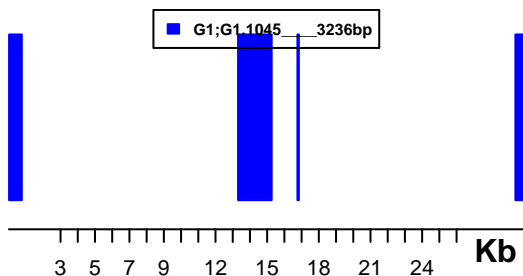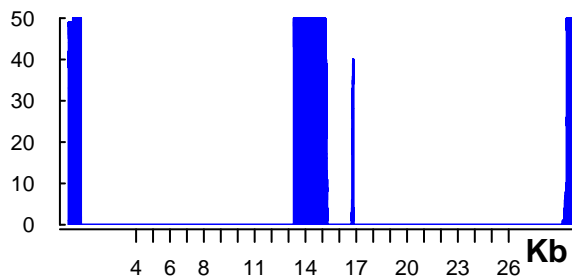

Supplement: Supplementary file 7 — Supplementary Data 5 [file 42003_2022_4058_MOESM7_ESM.zip › experiment1/Files_used_for_the_analysis_of_the_manuscript_experiment1/RNA_MODELS_WITH_COVERAGE_experiment_1_passage_30.pdf]

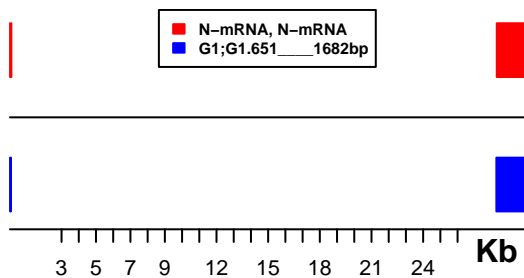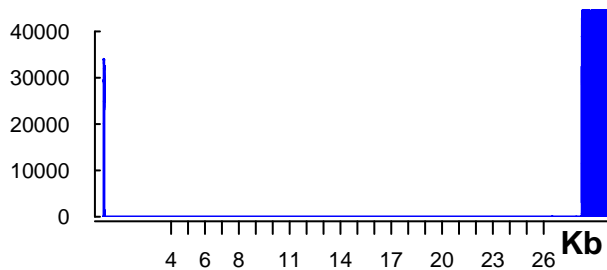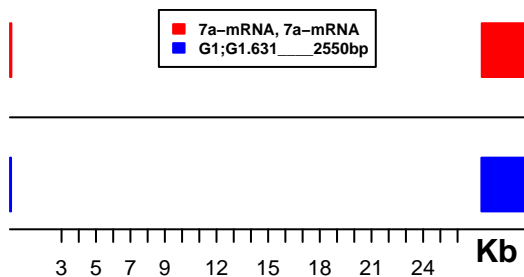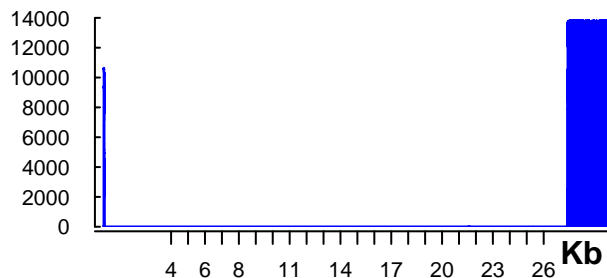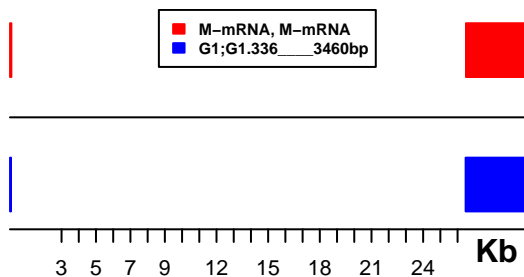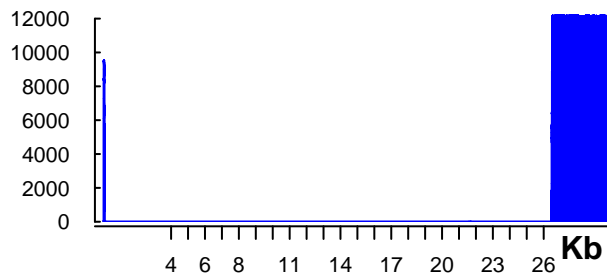

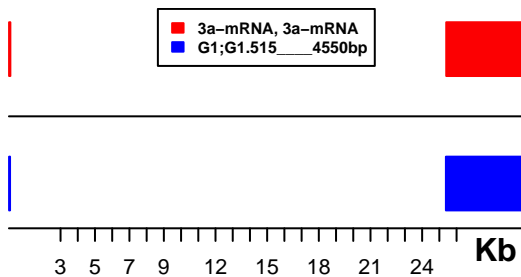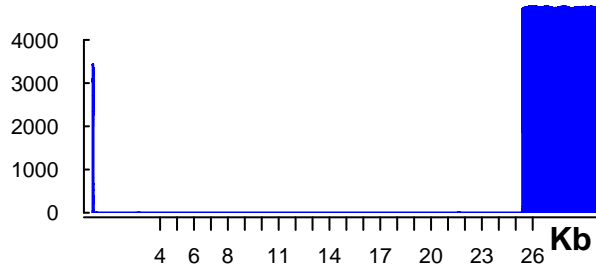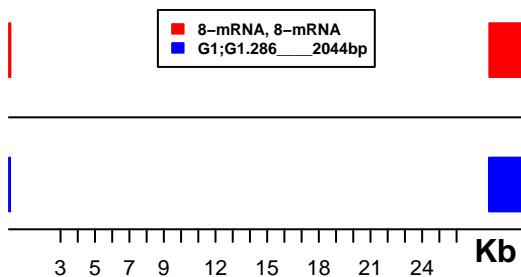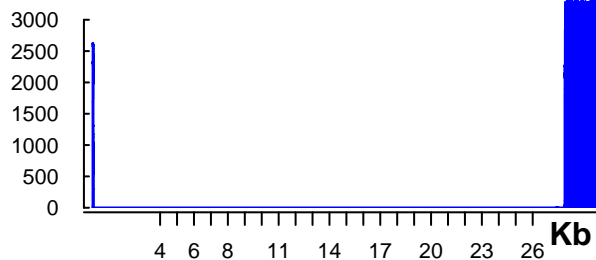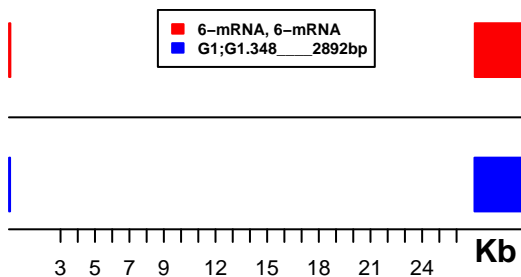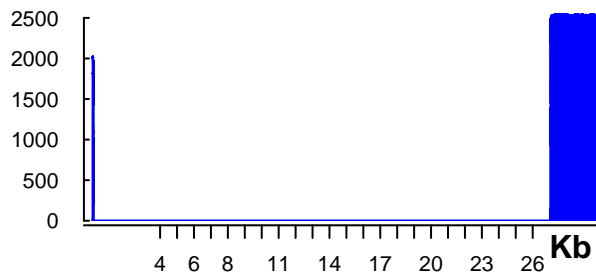

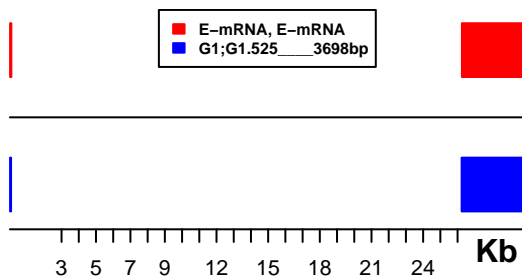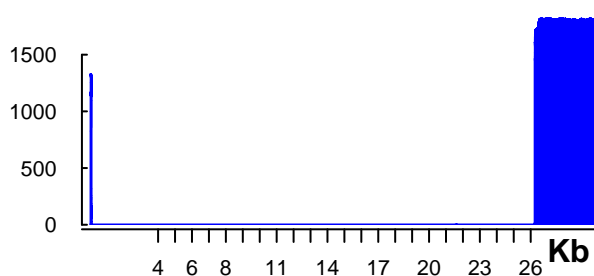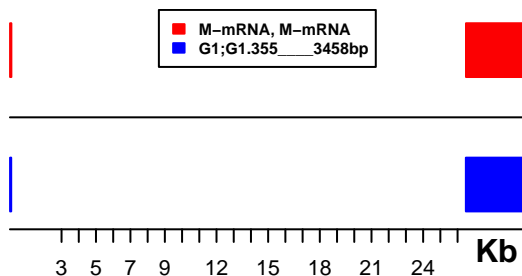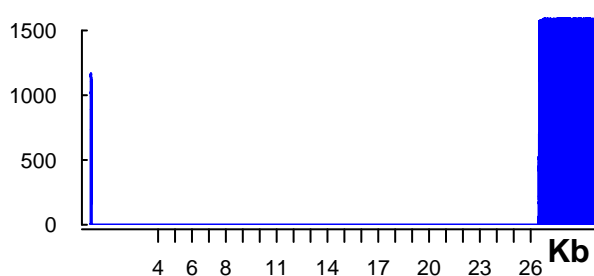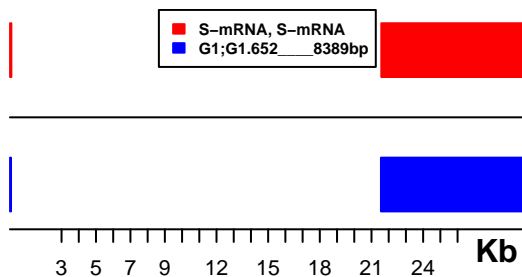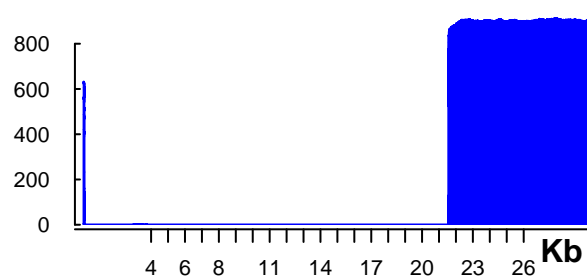

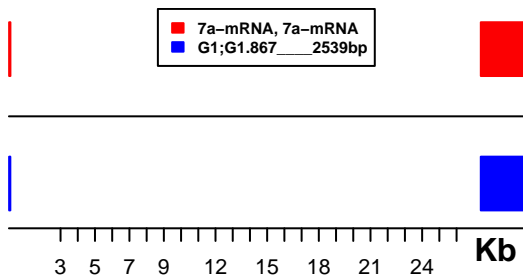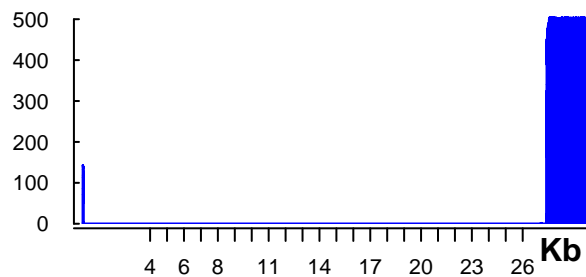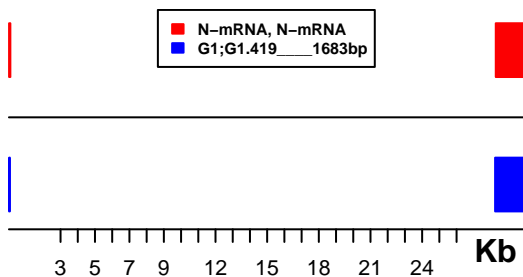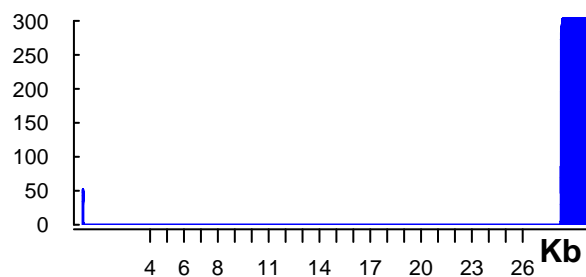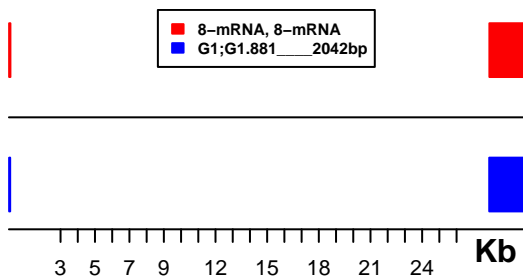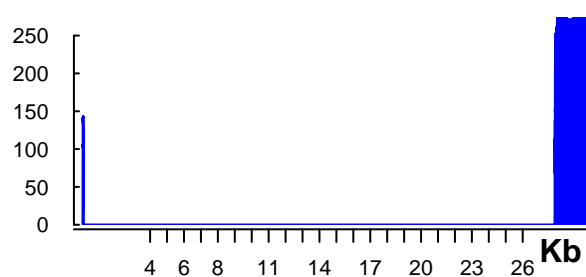

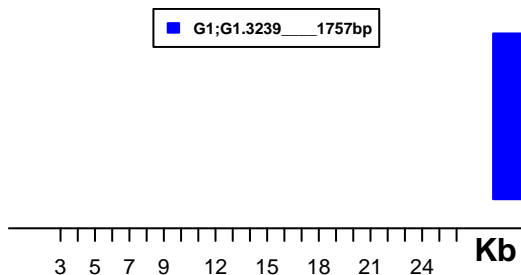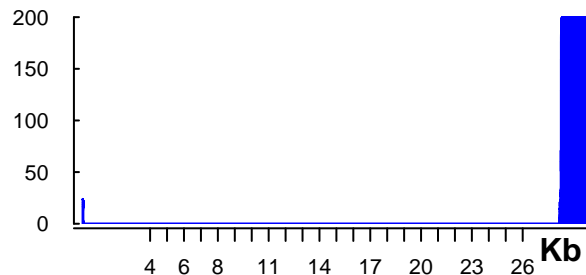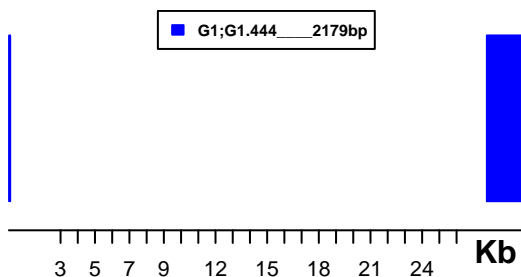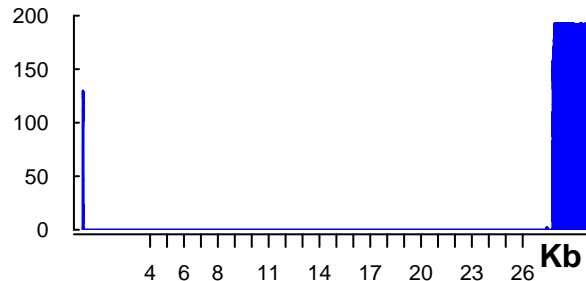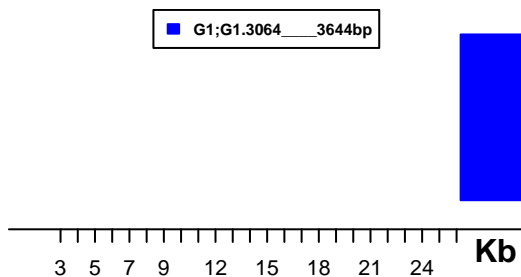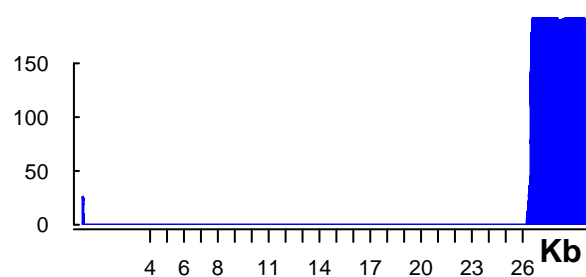

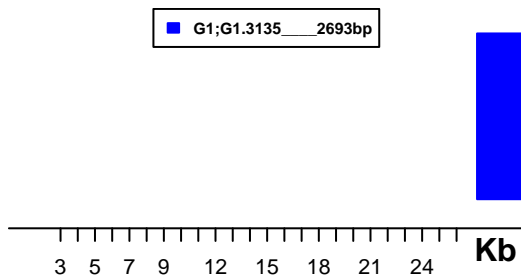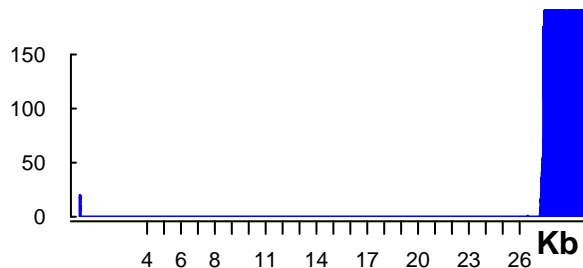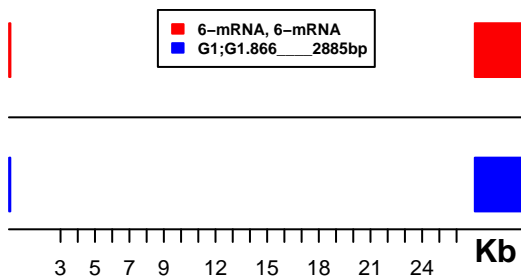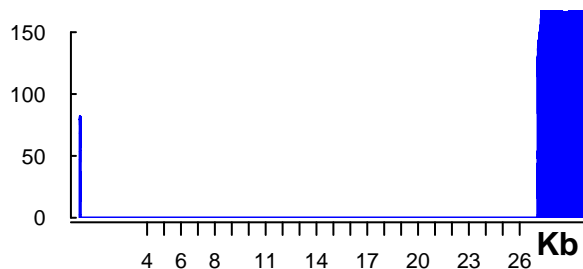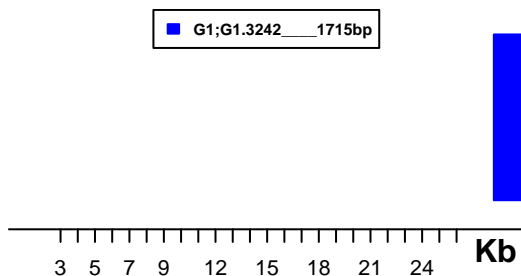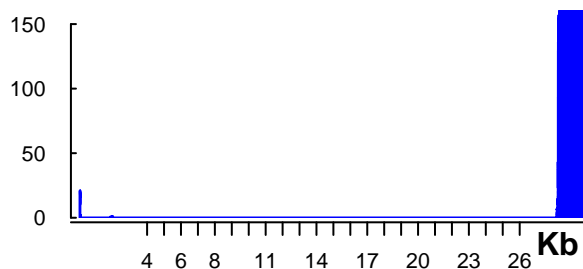

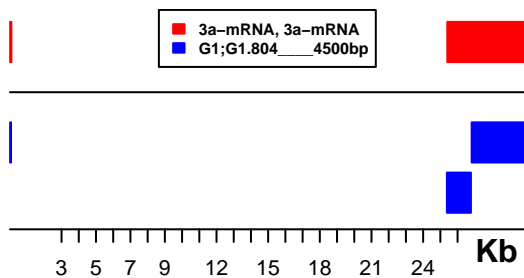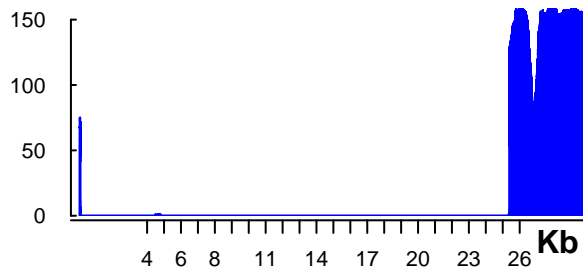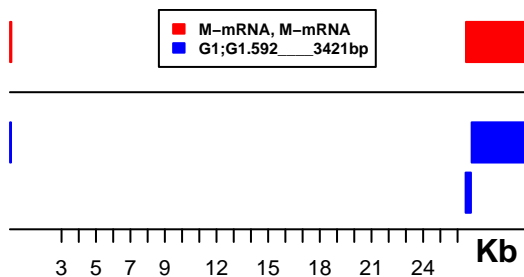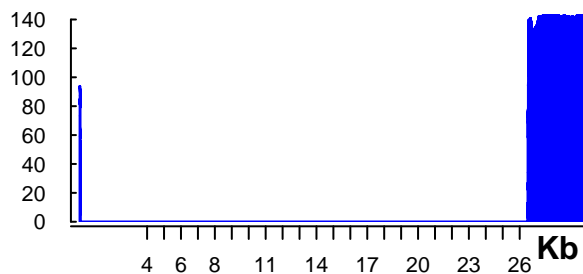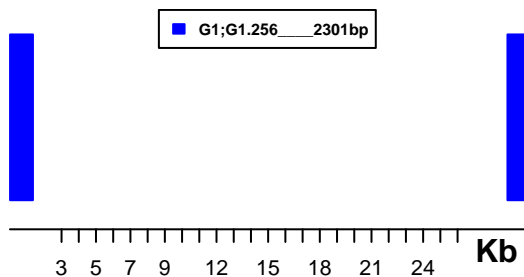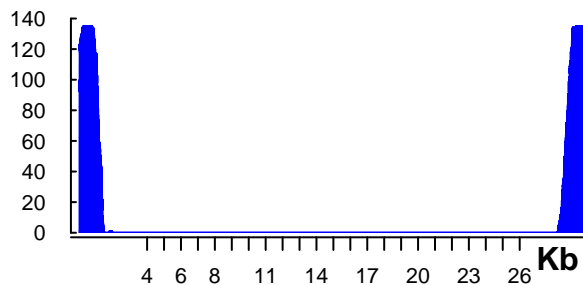

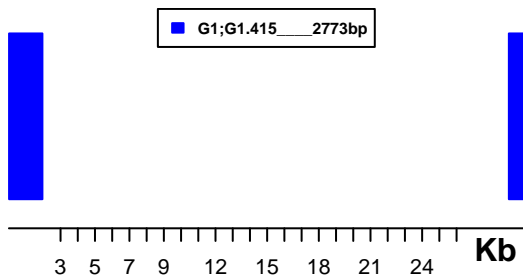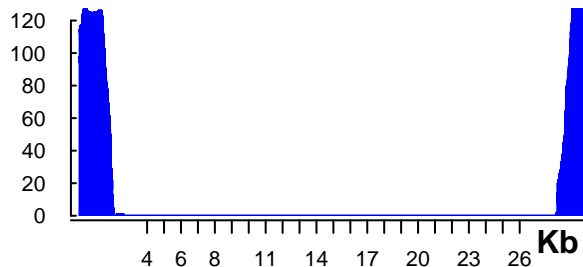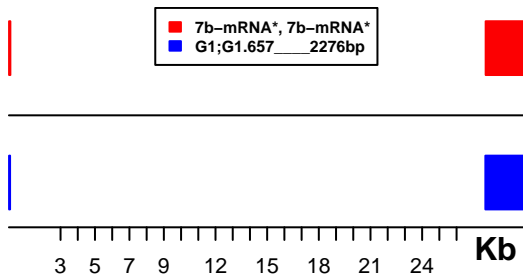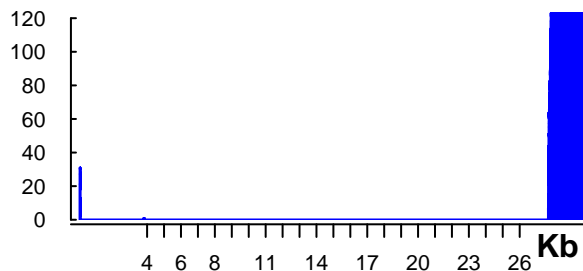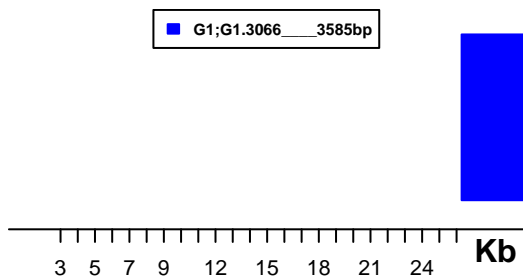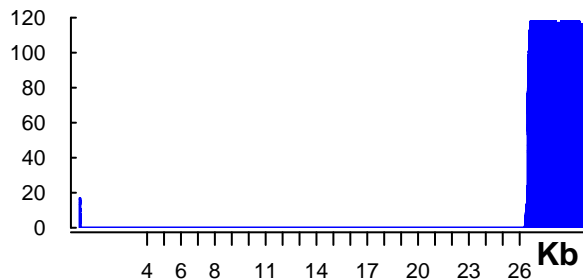

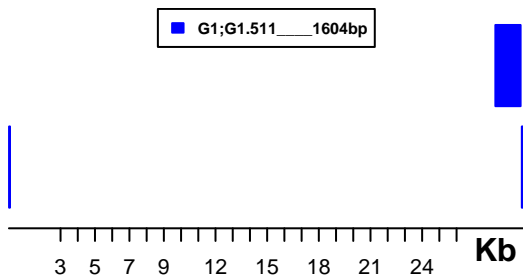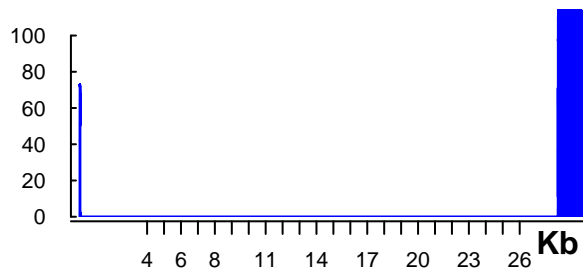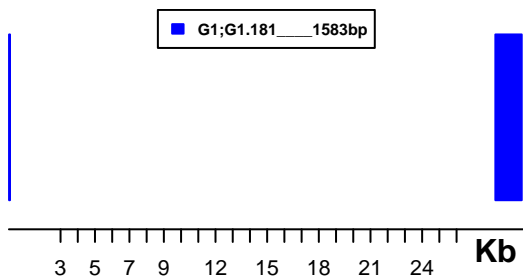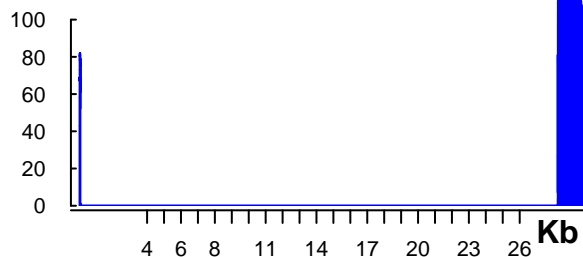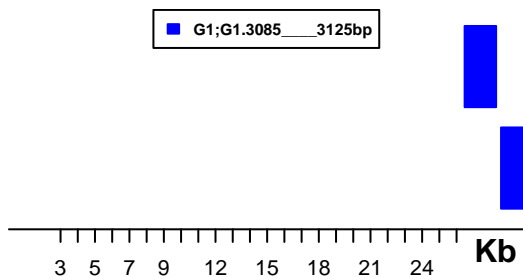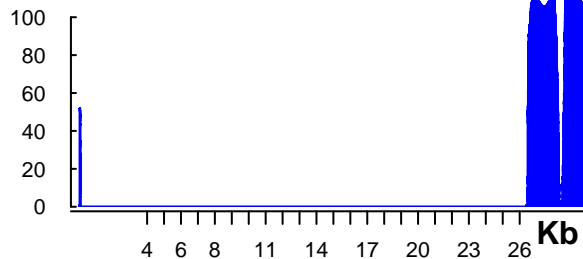

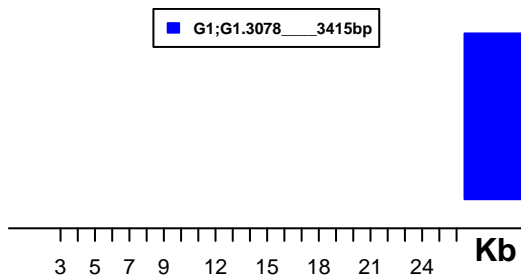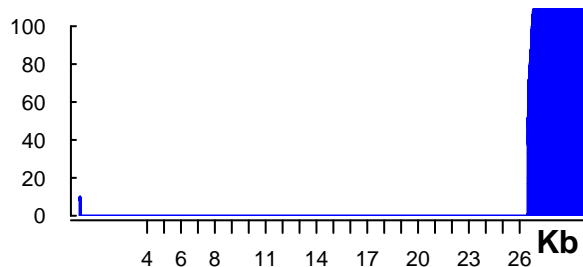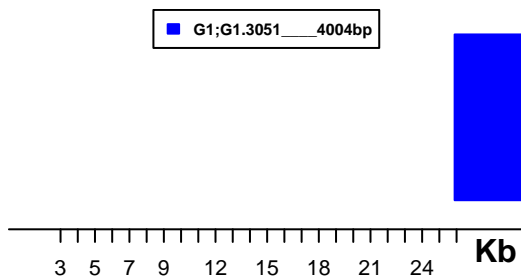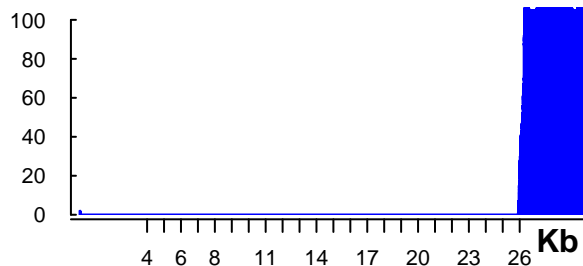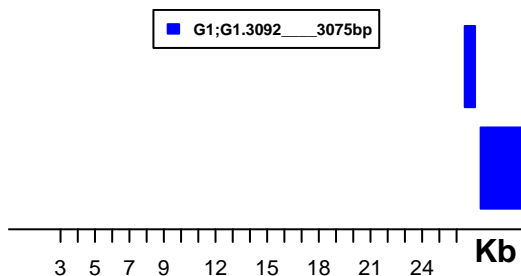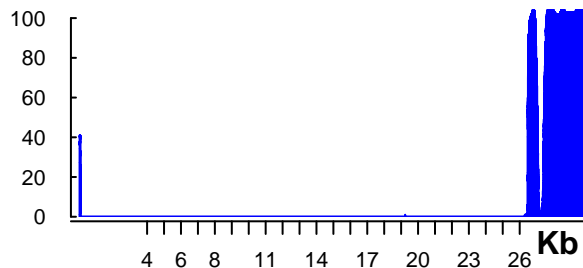

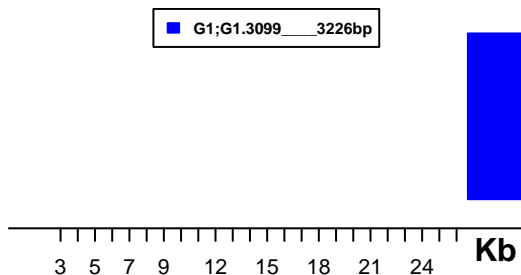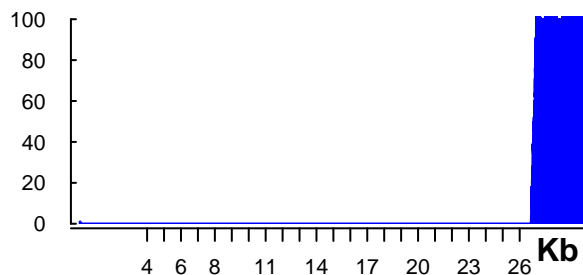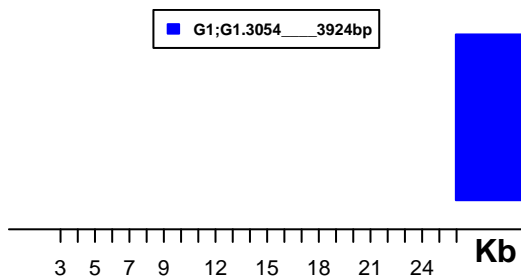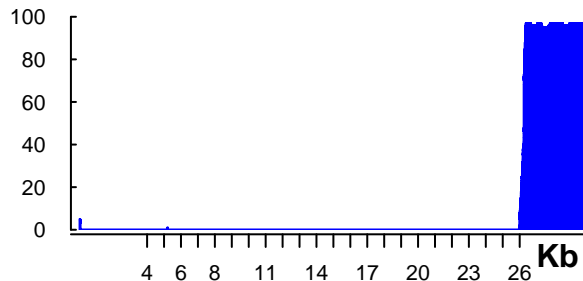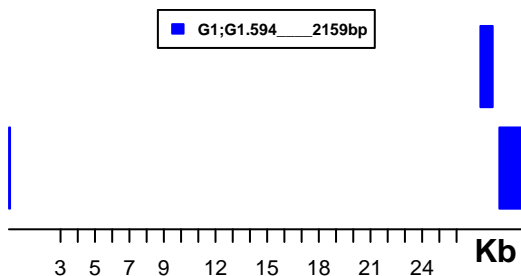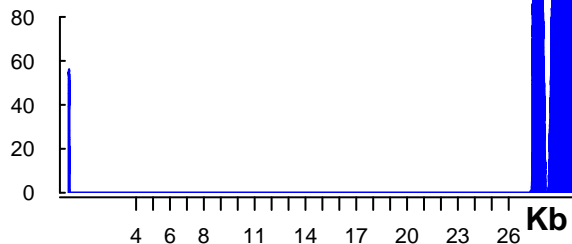

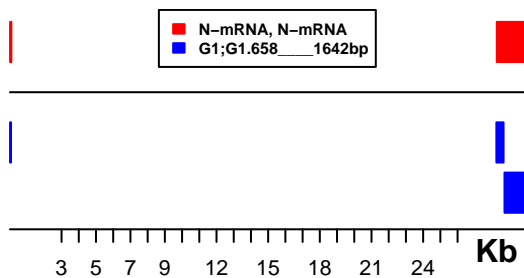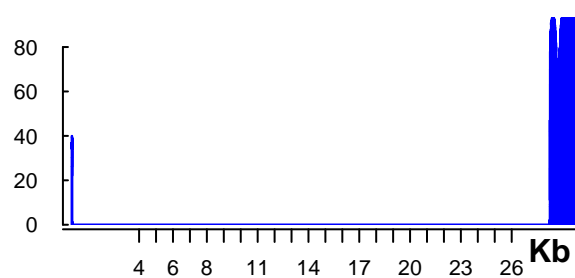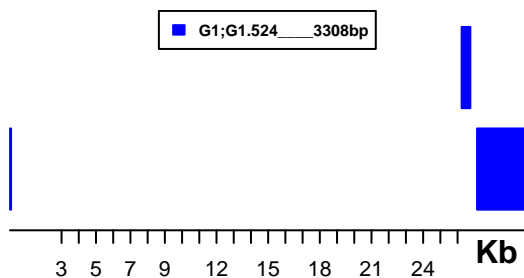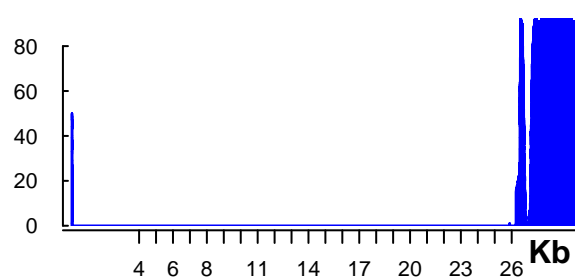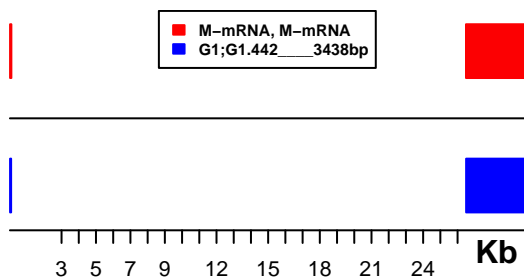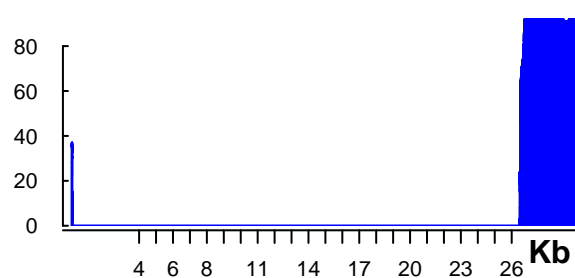

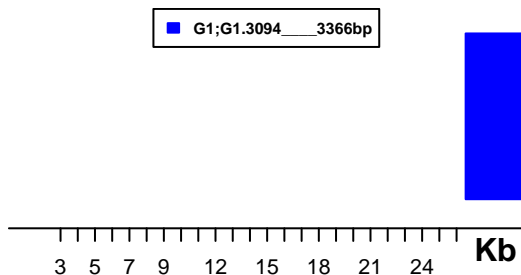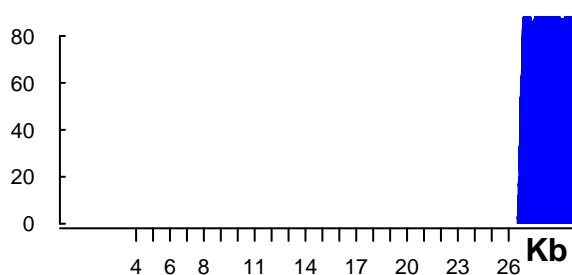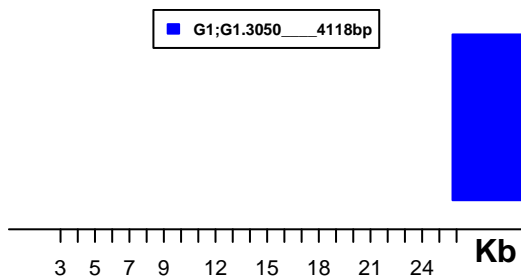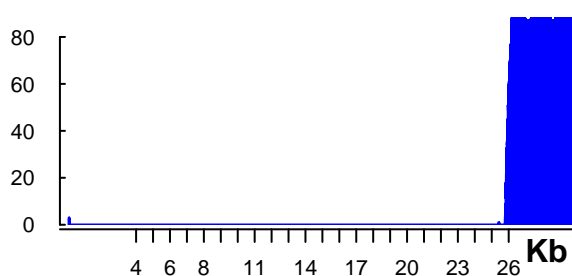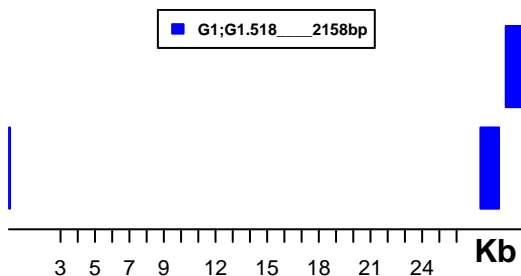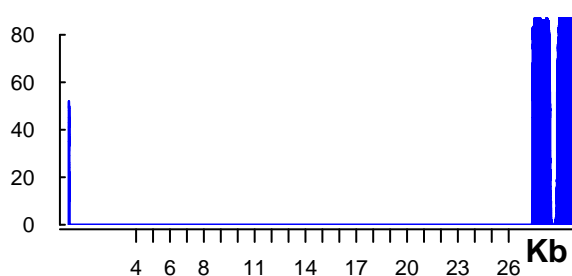

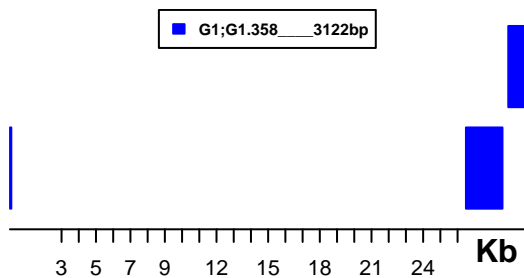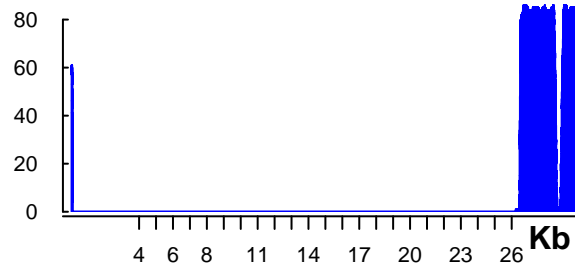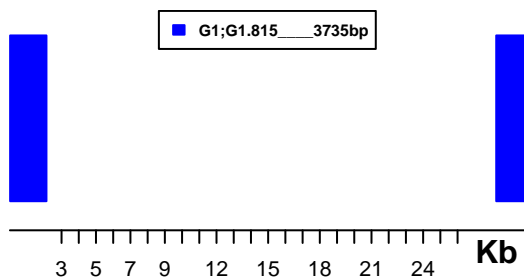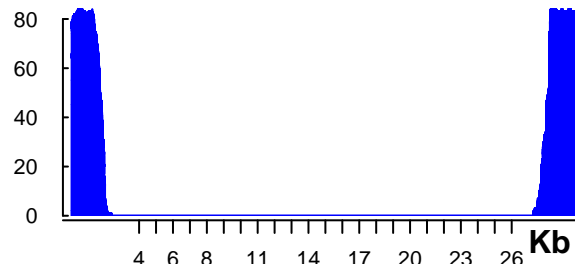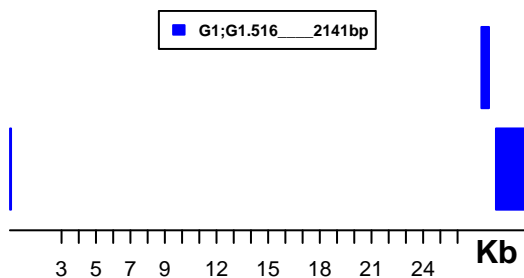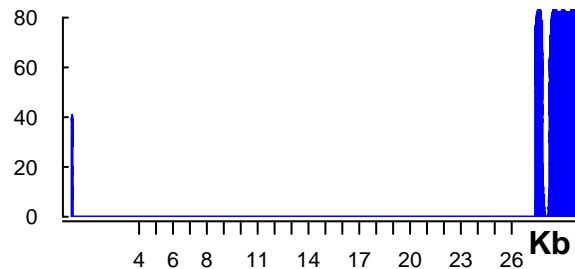

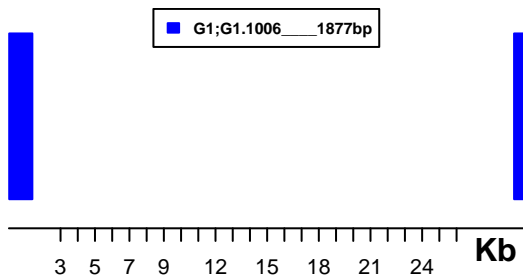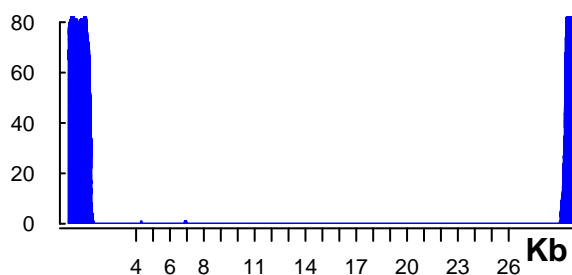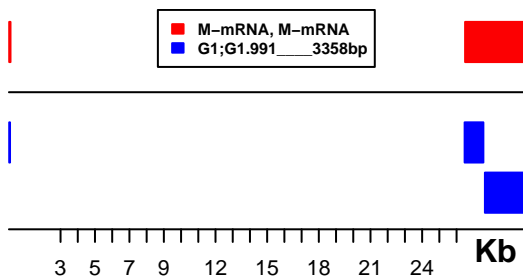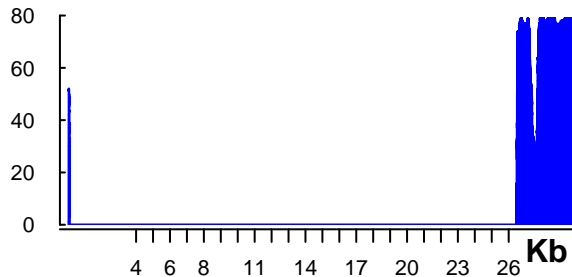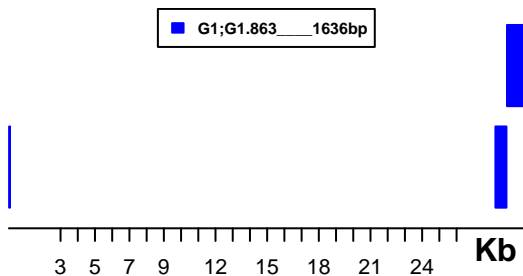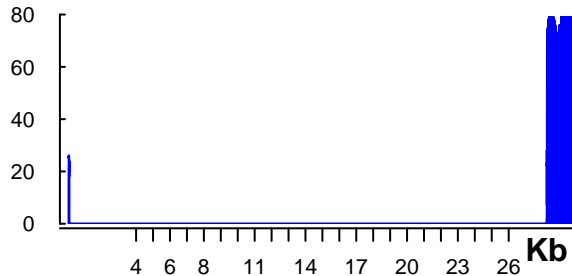

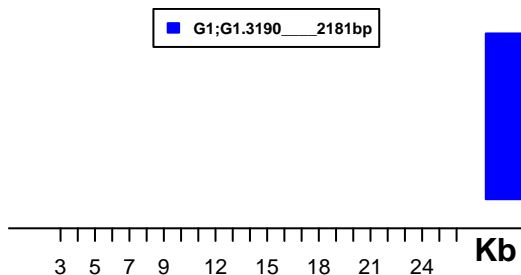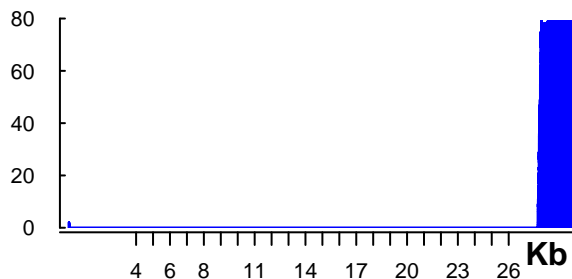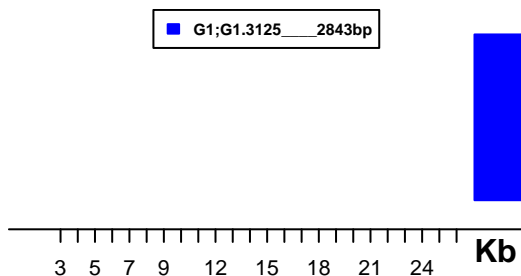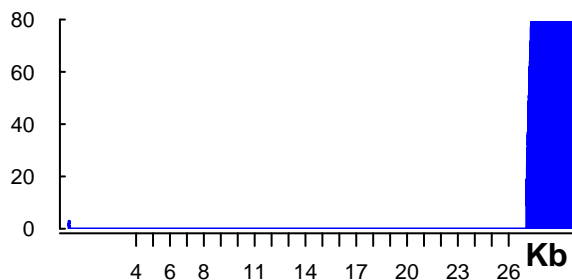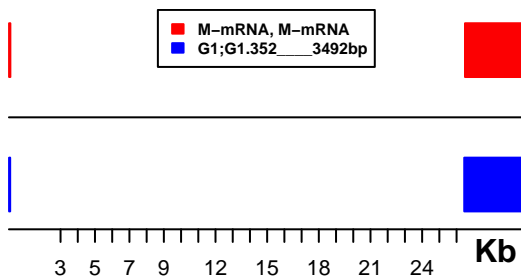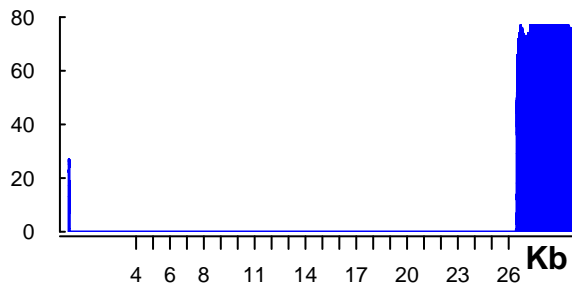

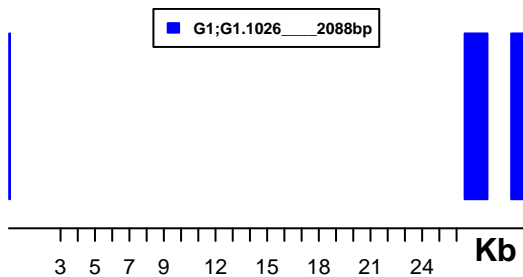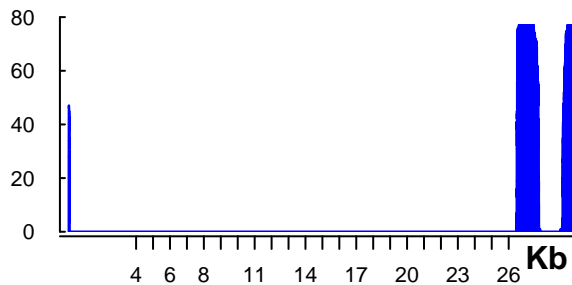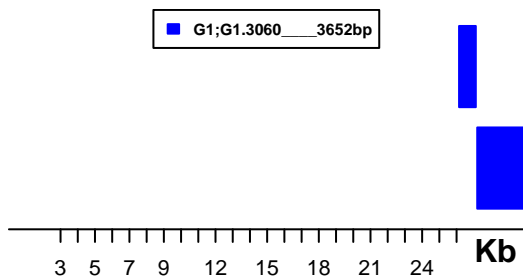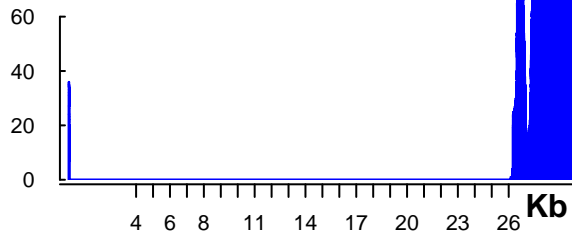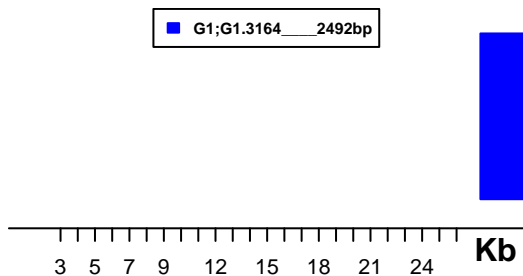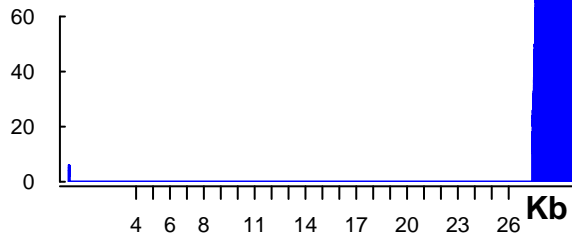

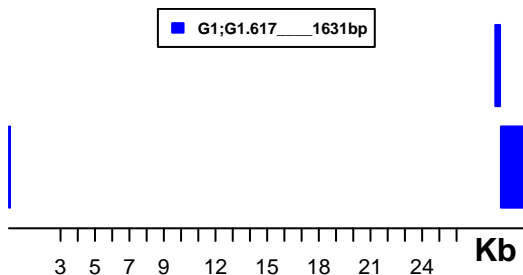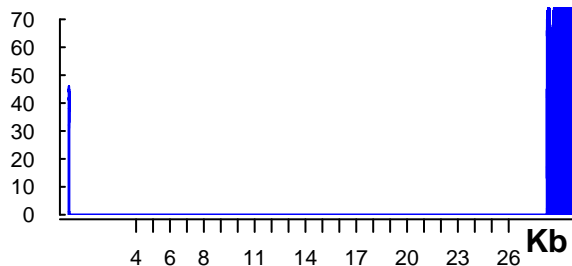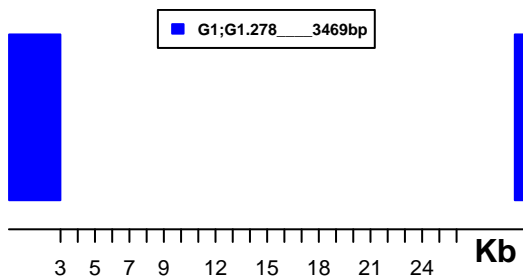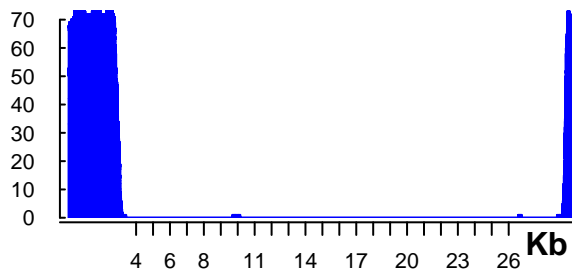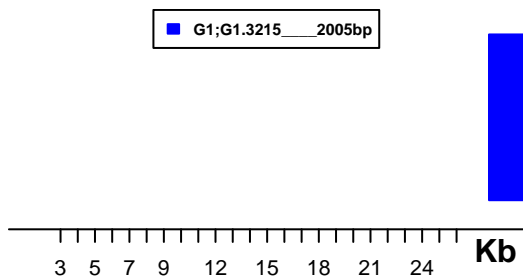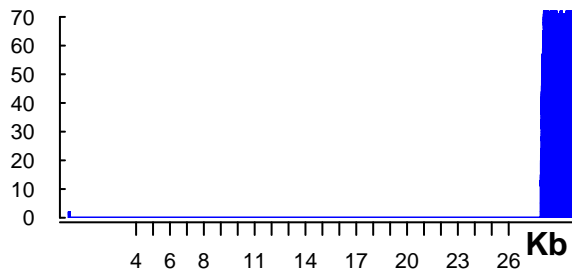

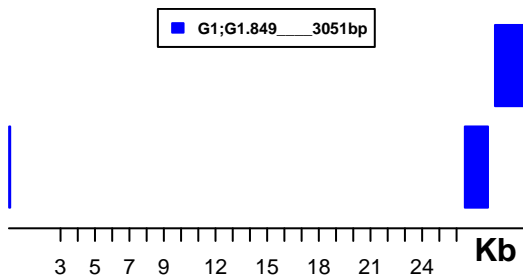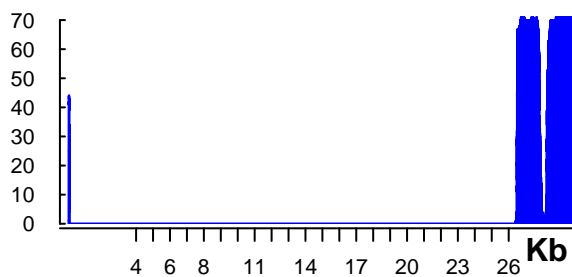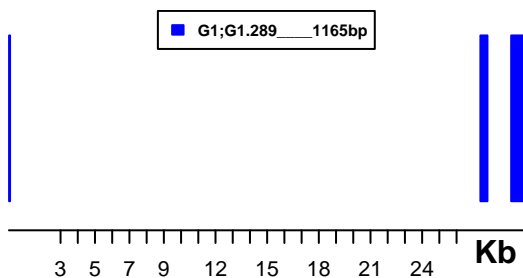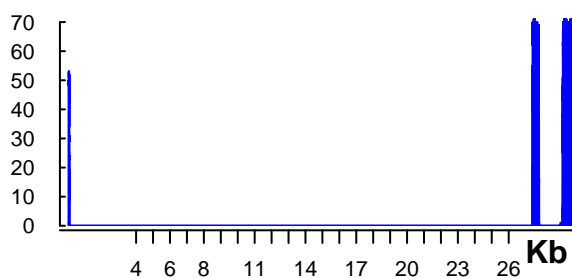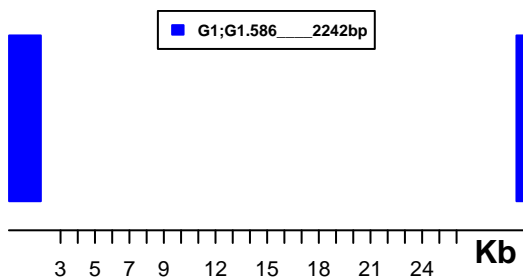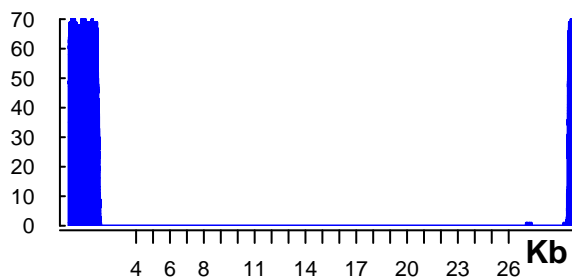

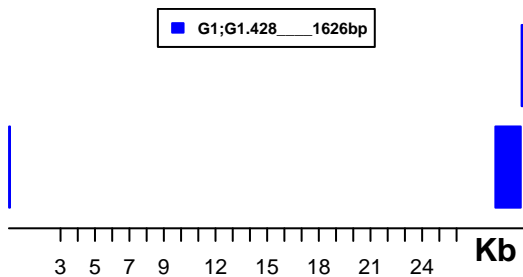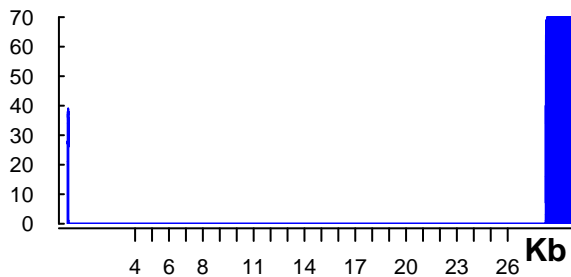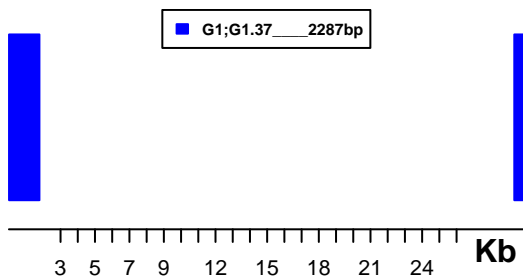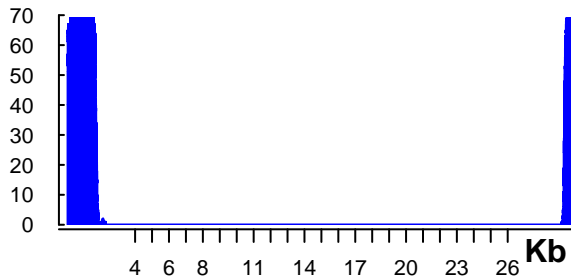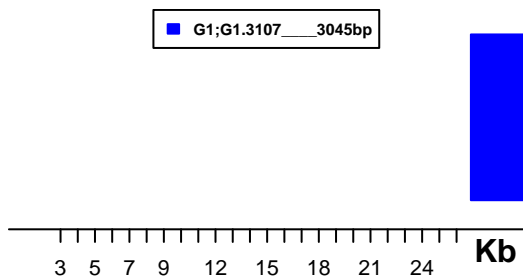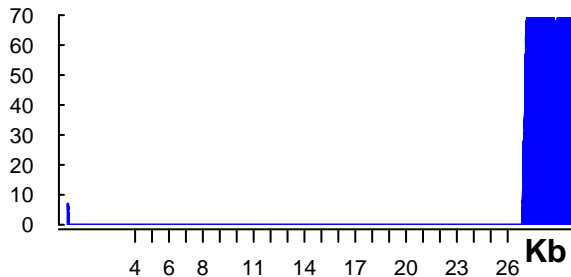

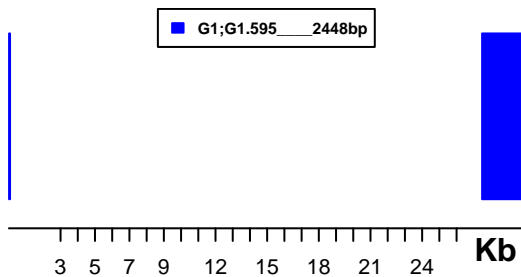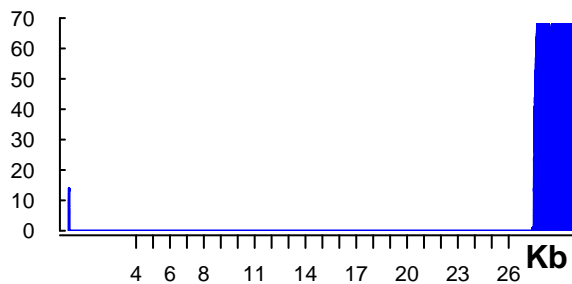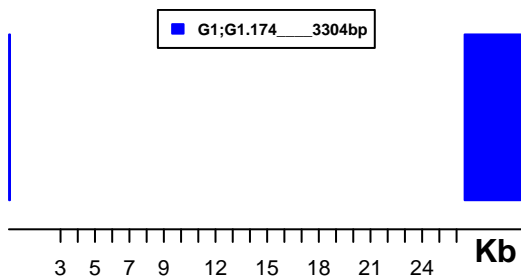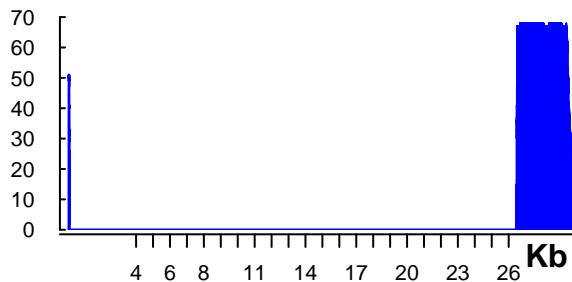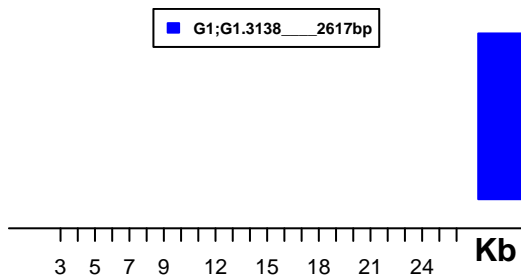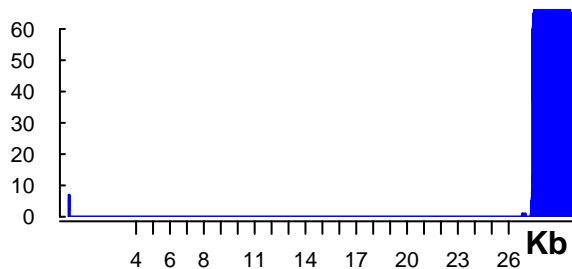

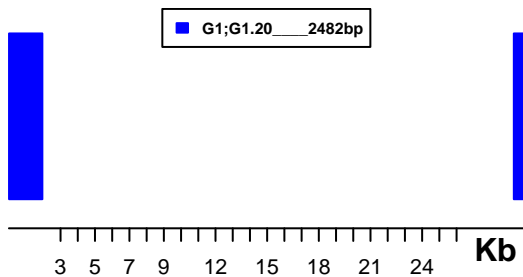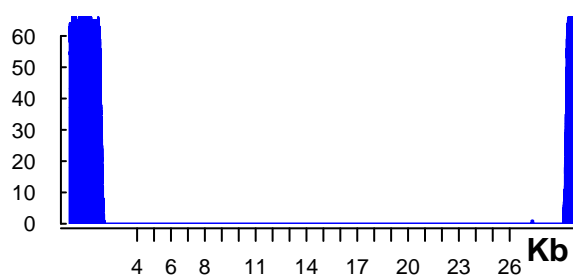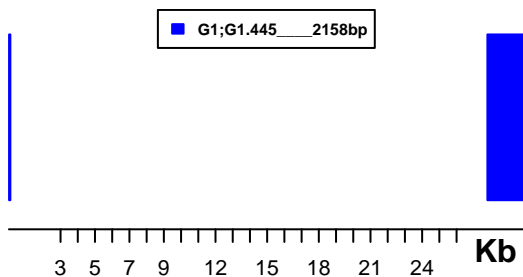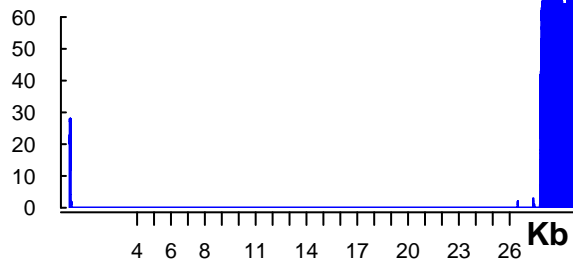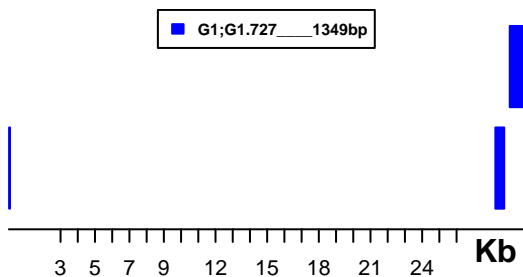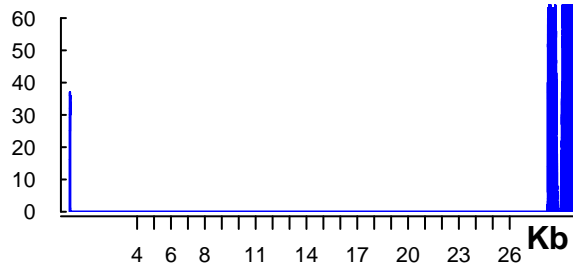

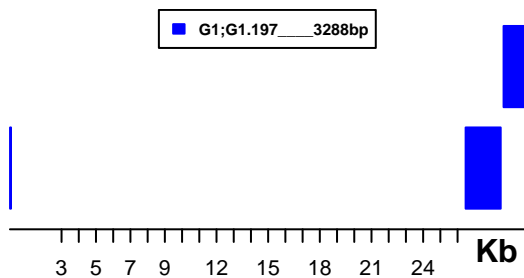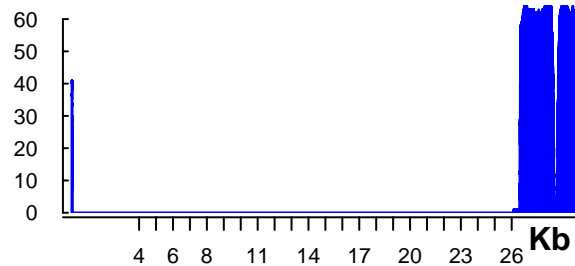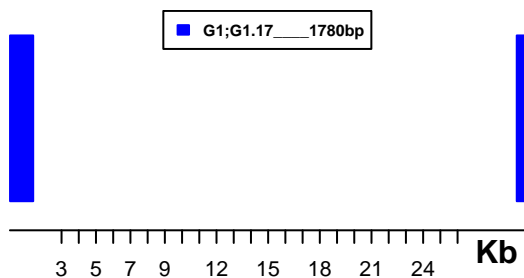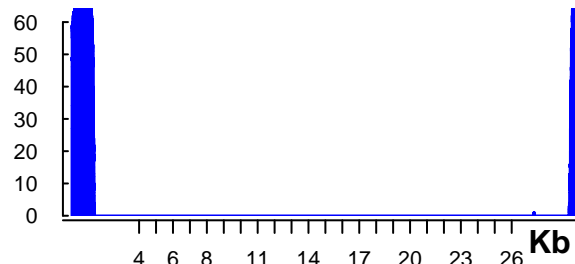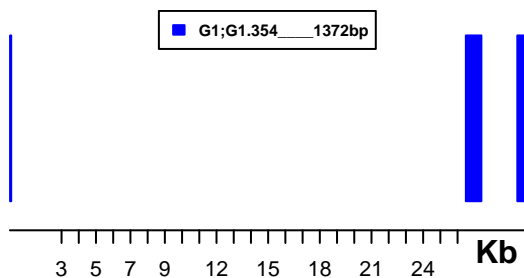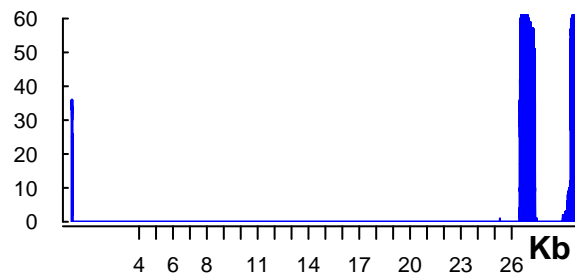

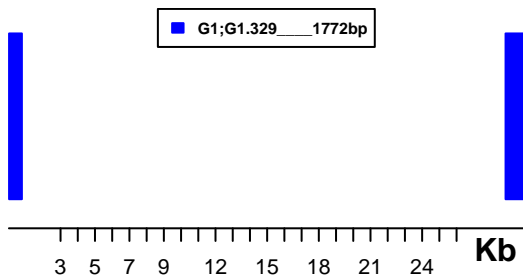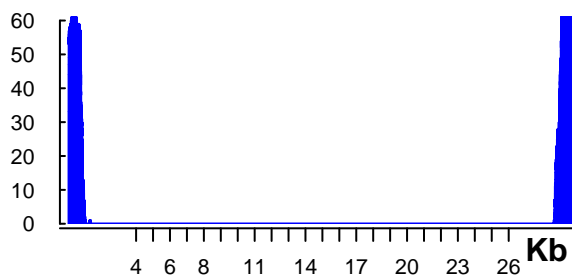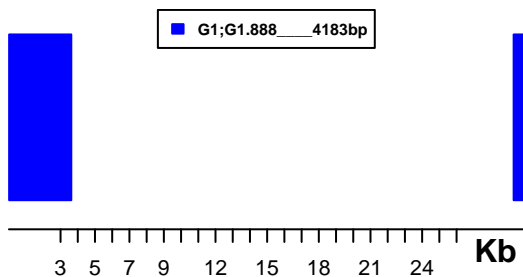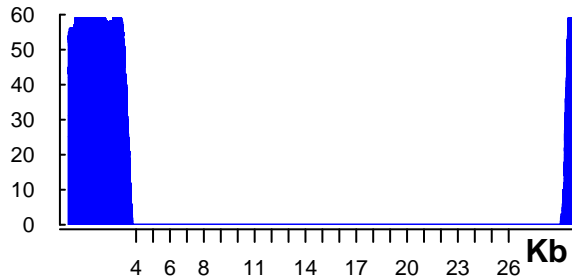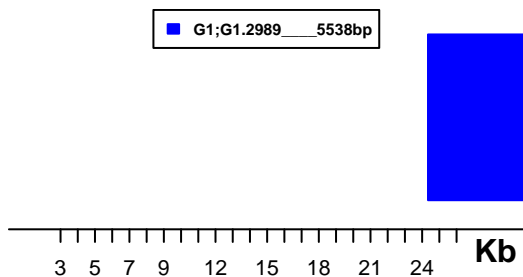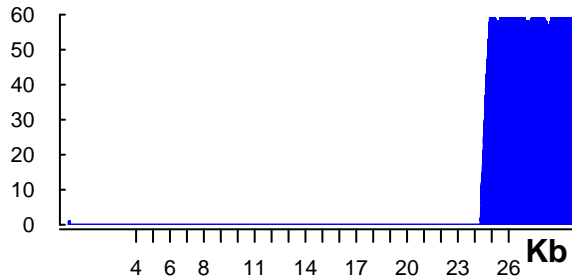

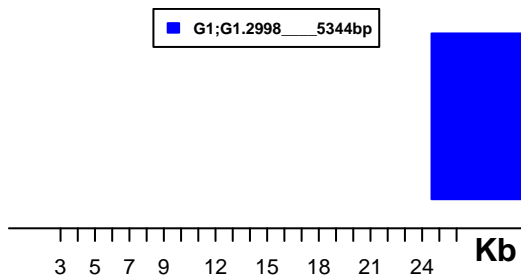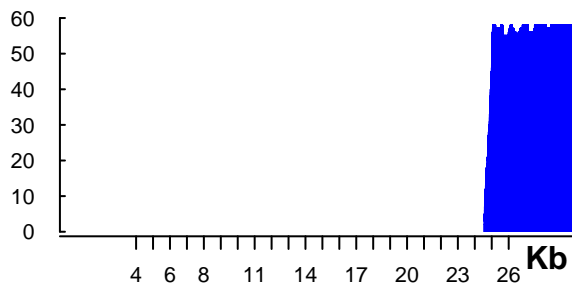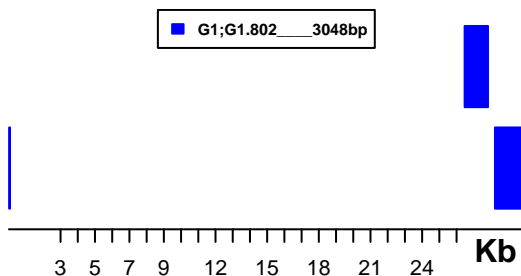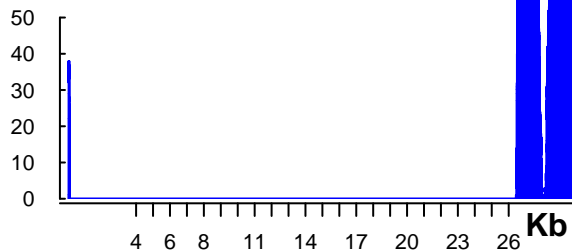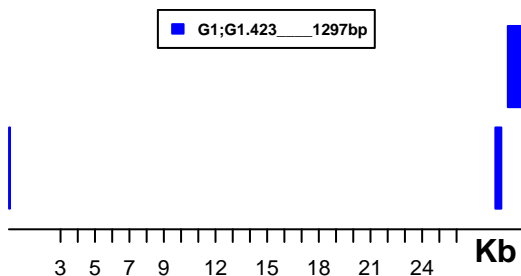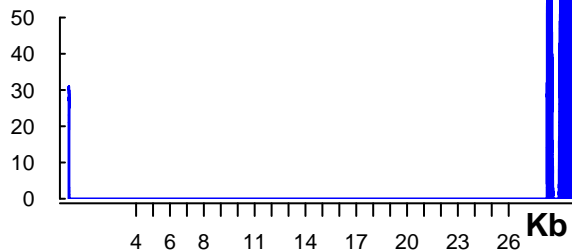

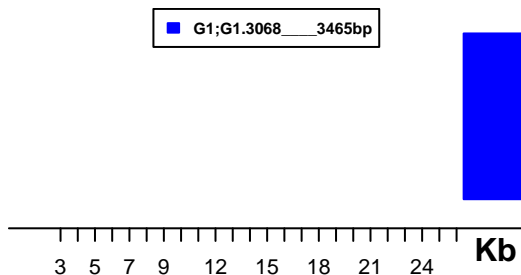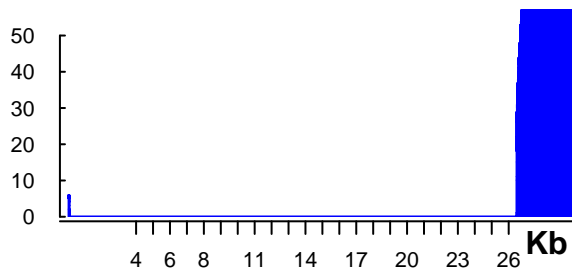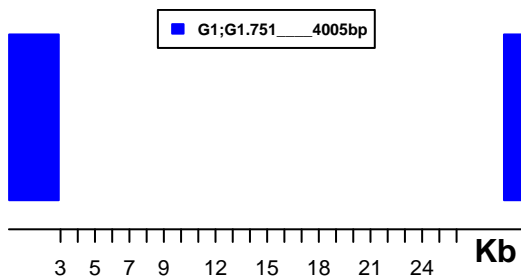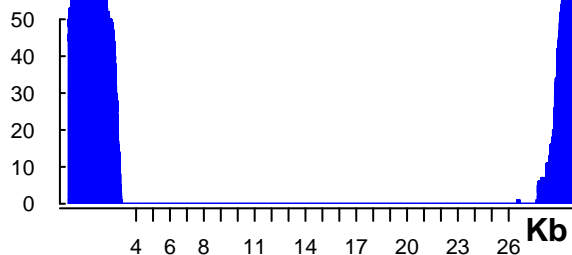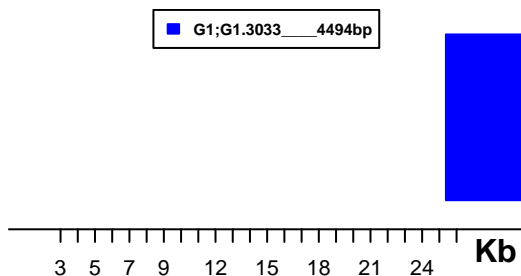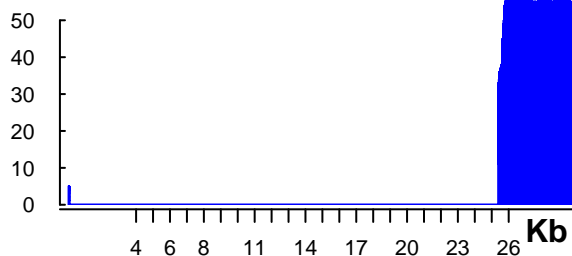

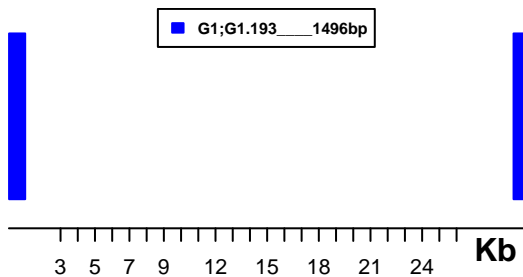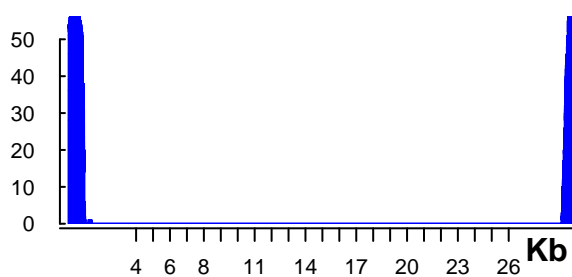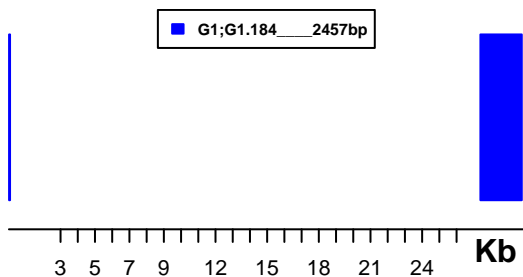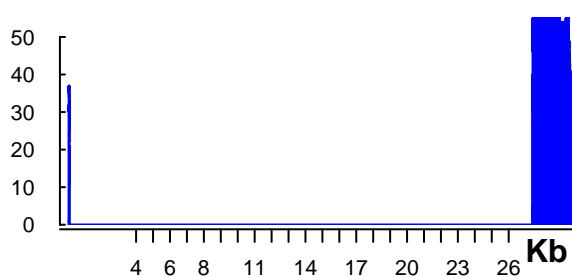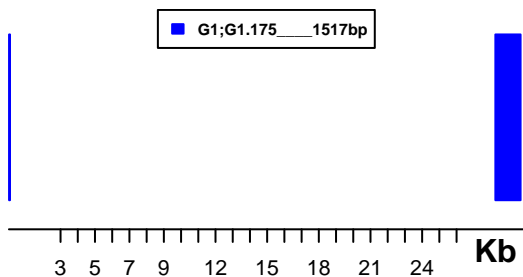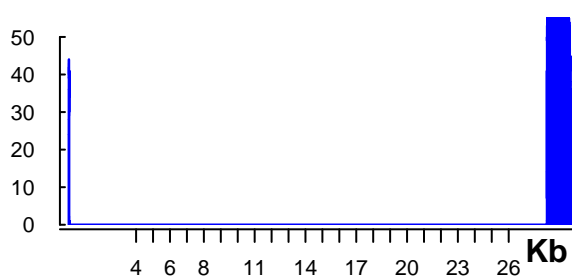

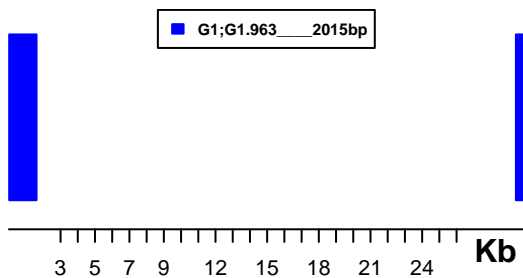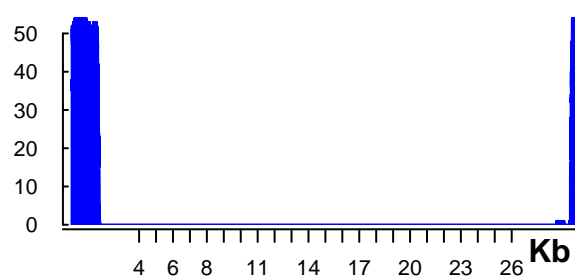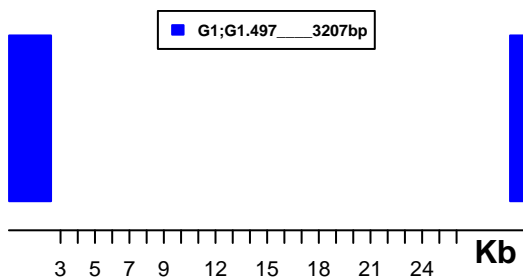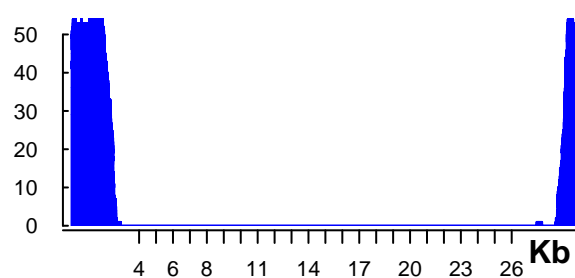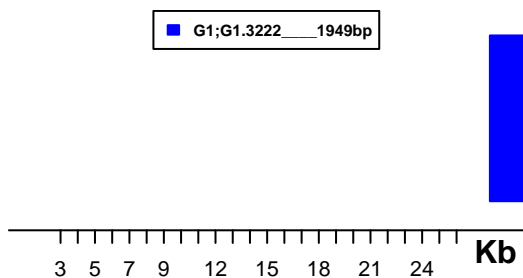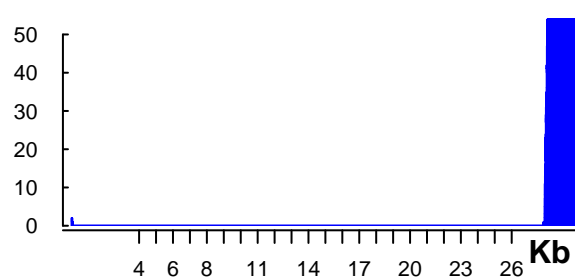

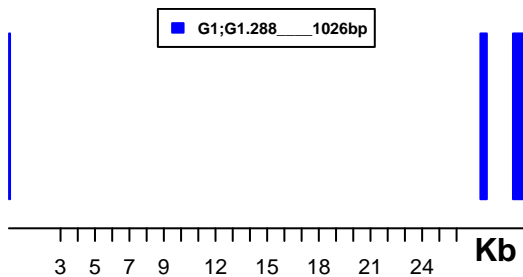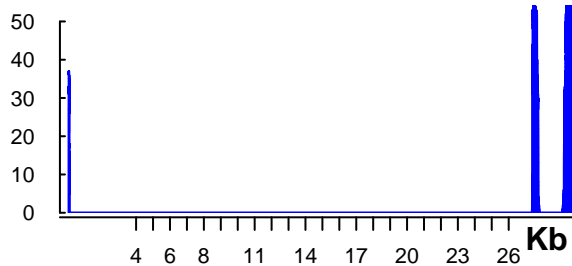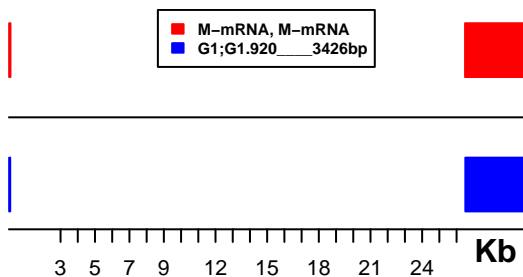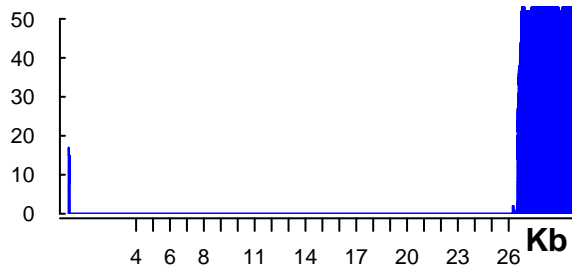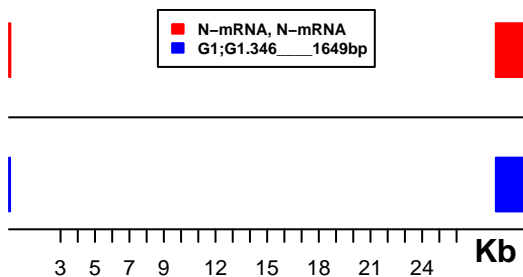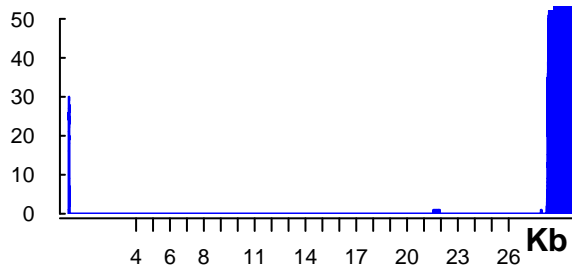

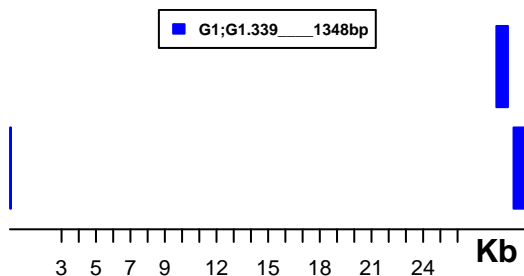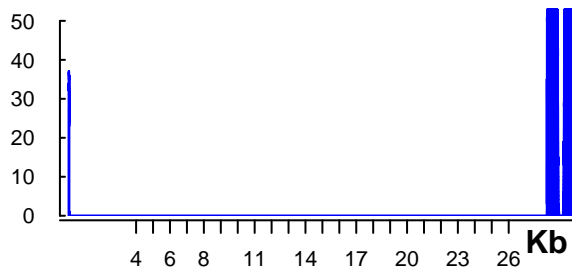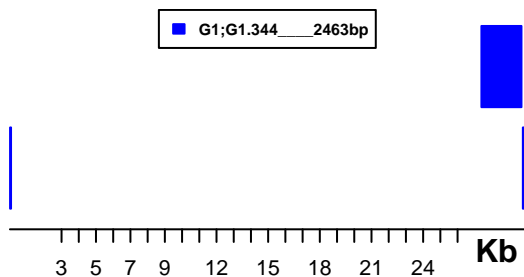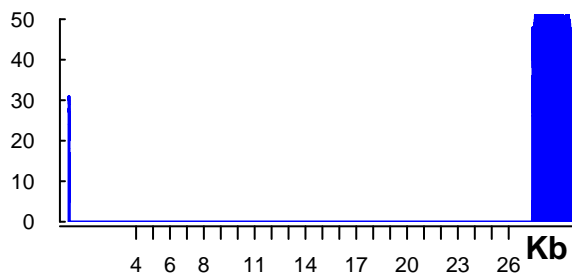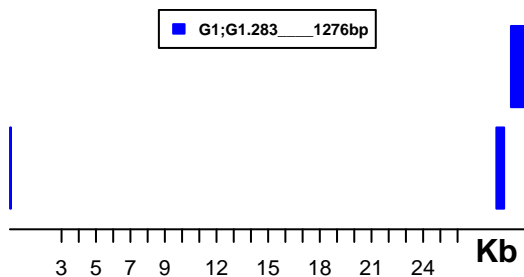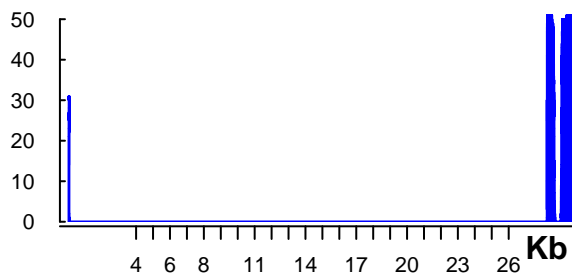

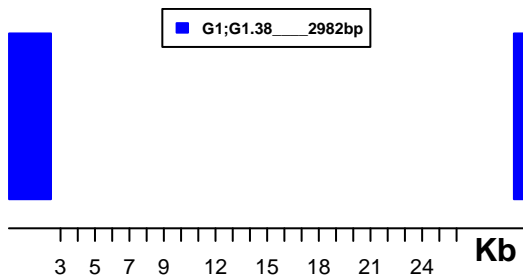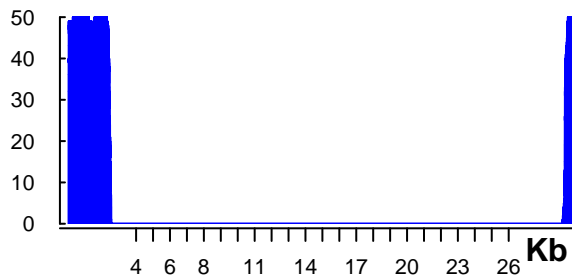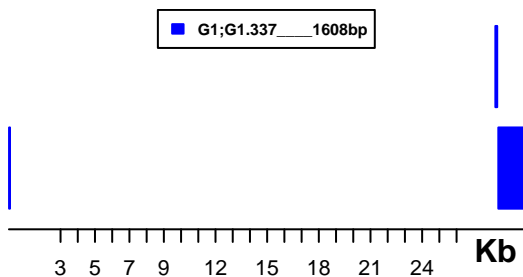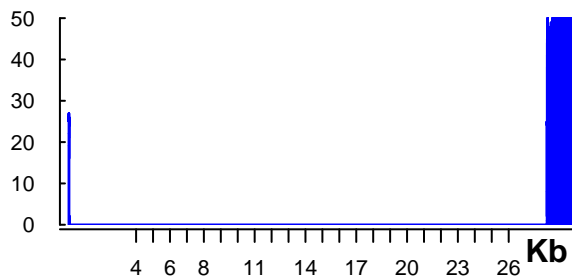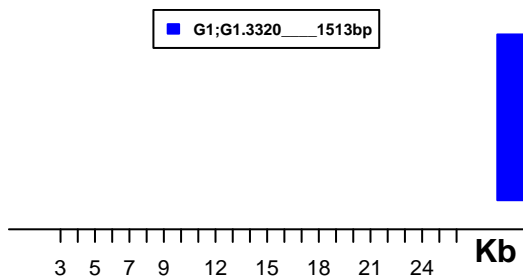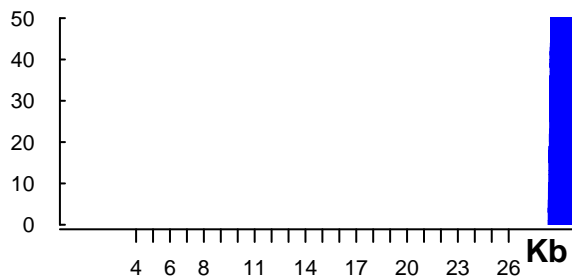

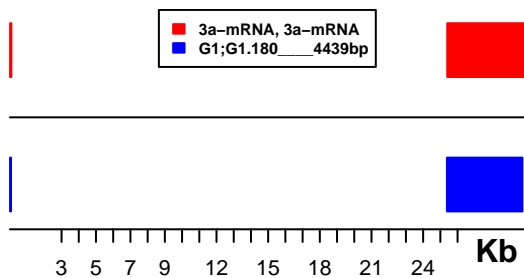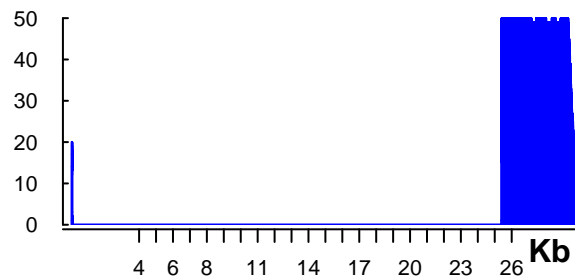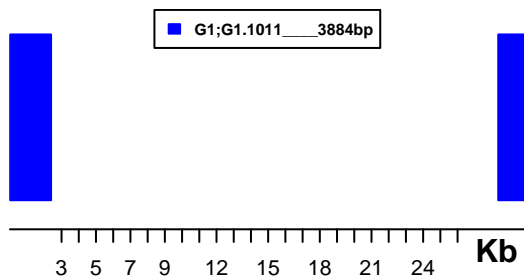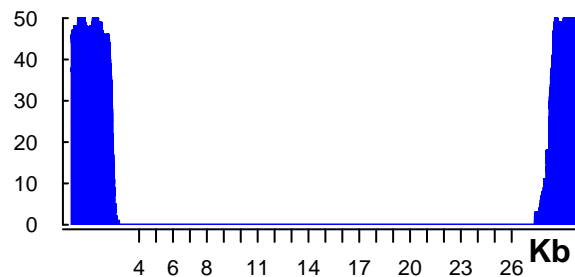

Supplement: Supplementary file 7 — Supplementary Data 5 [file 42003_2022_4058_MOESM7_ESM.zip › experiment2/Files_used_for_the_analysis_of_the_manuscript_experiment2/RNA_MODELS_WITH_COVERAGE_experiment_2_passage_1.pdf]

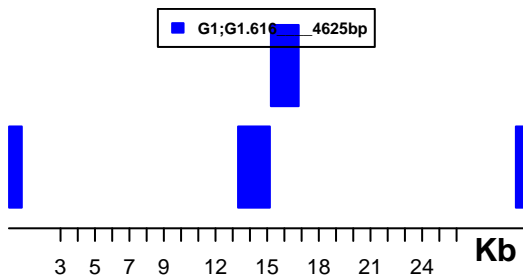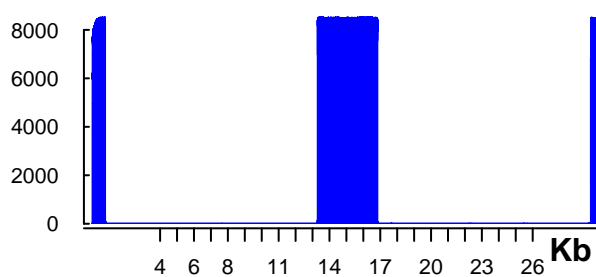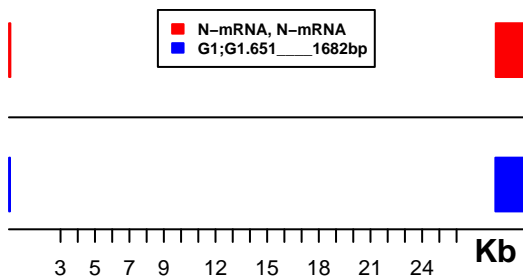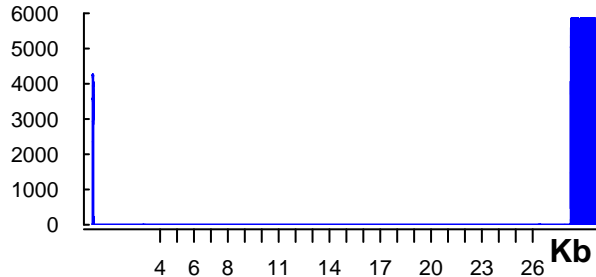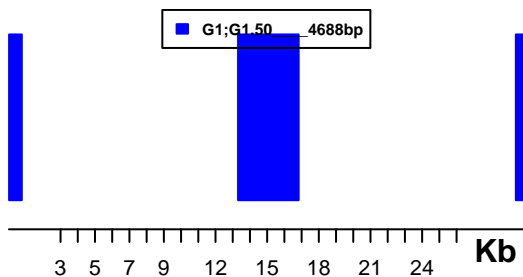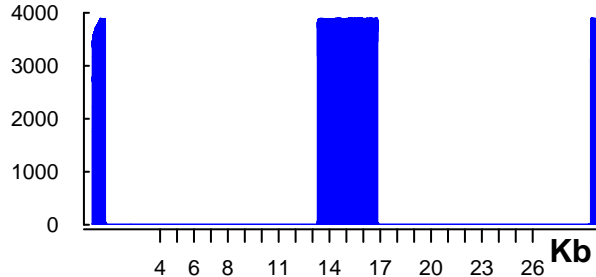

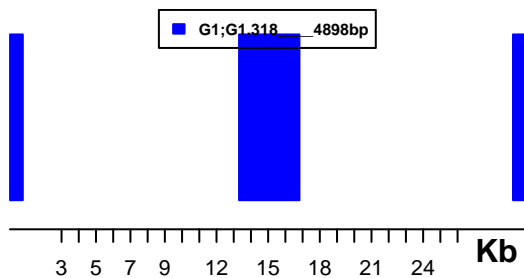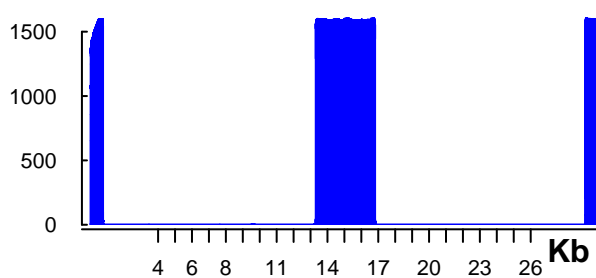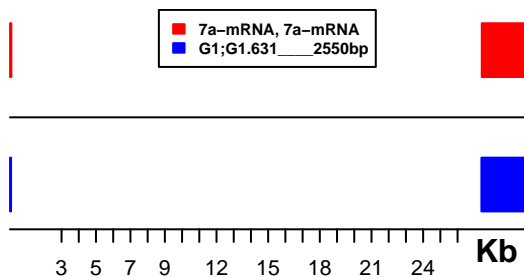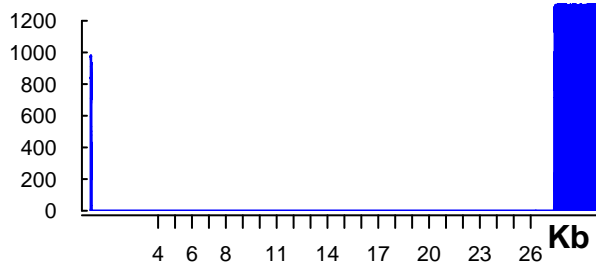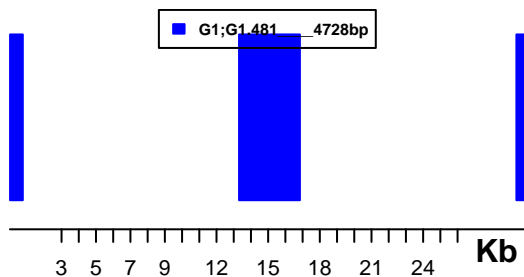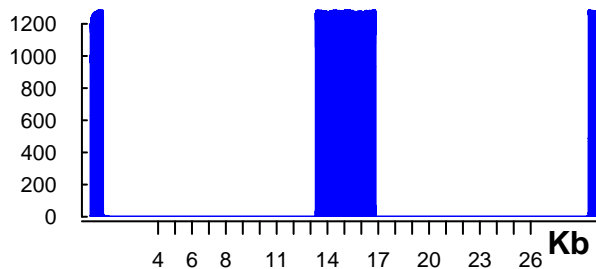

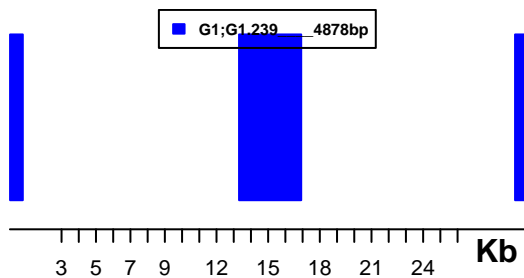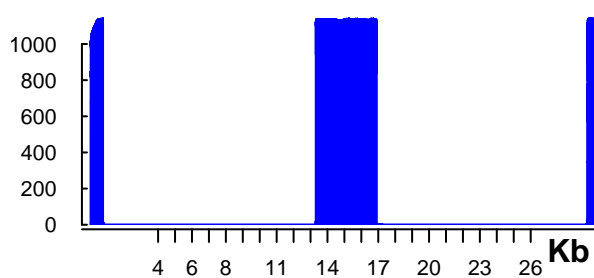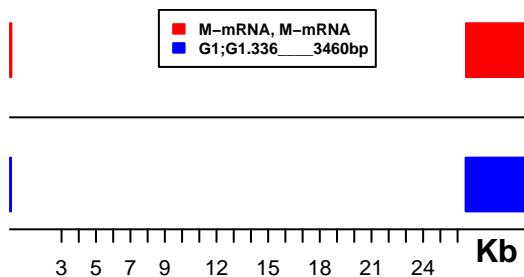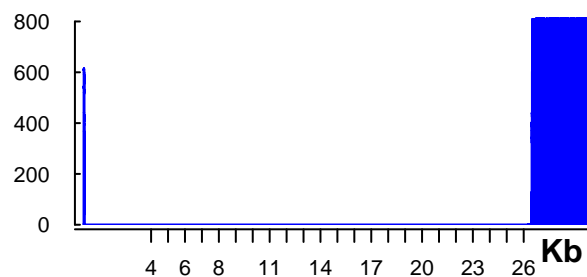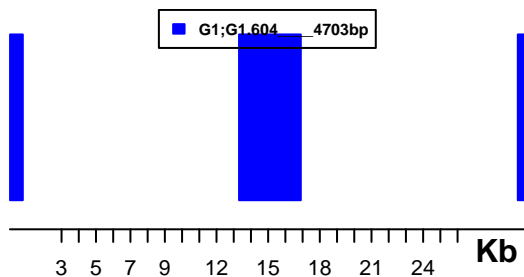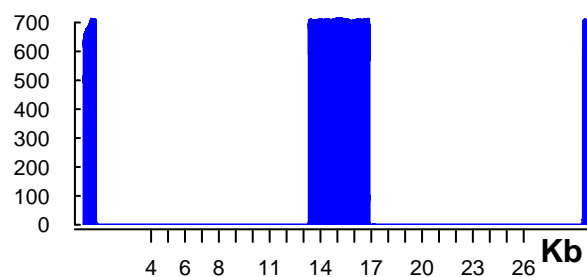

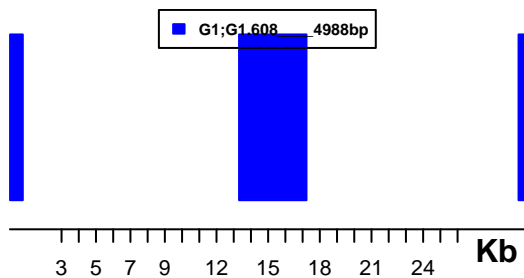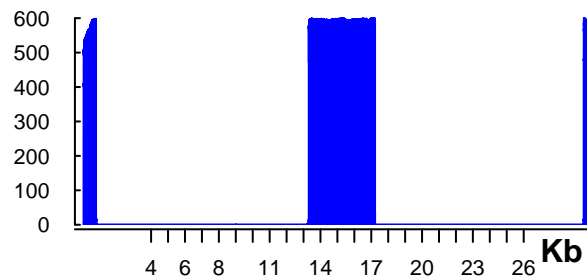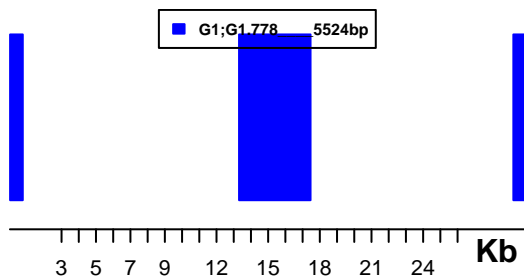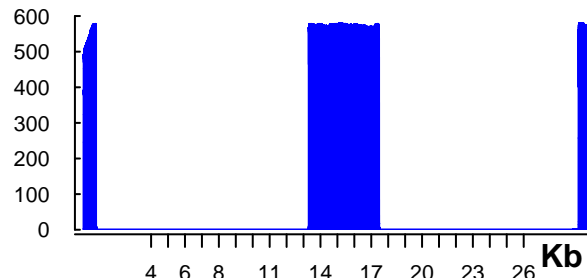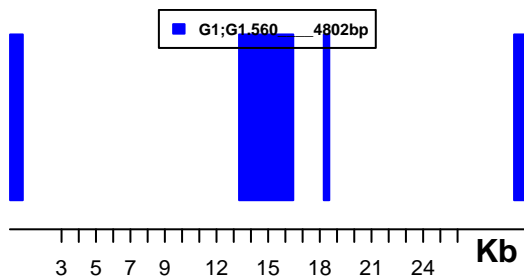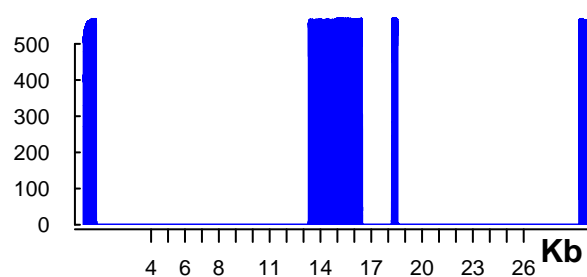

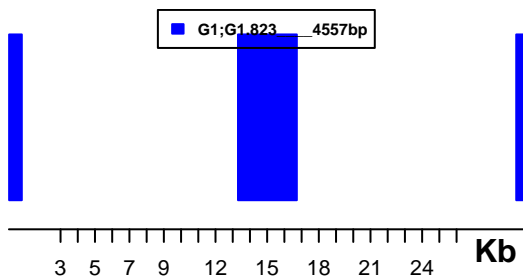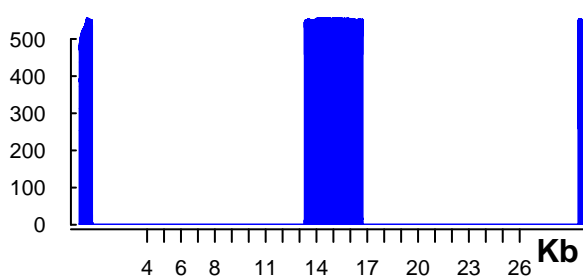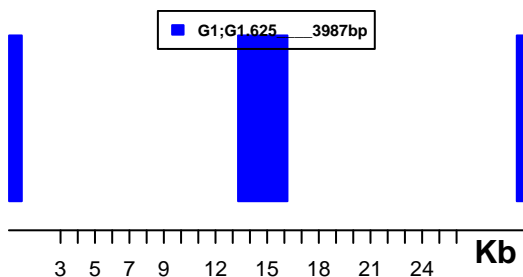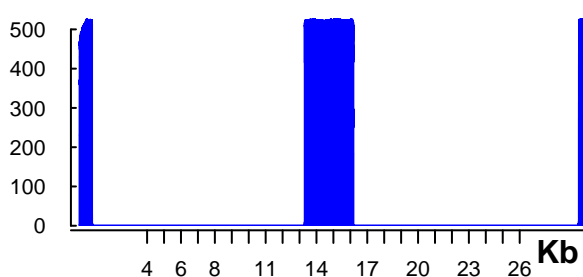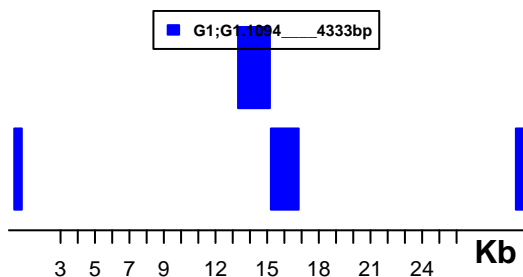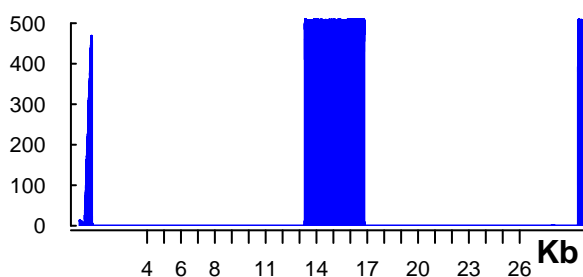

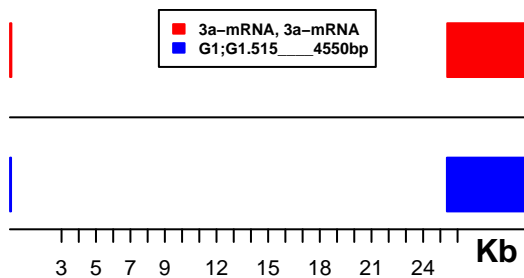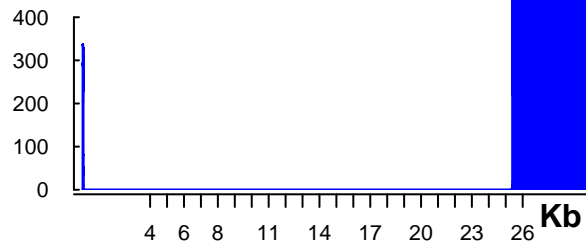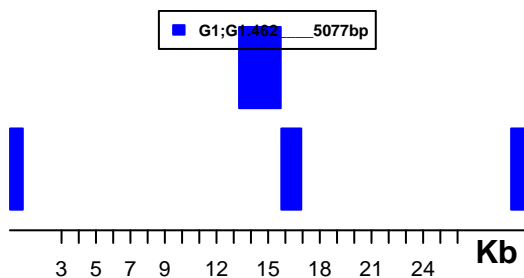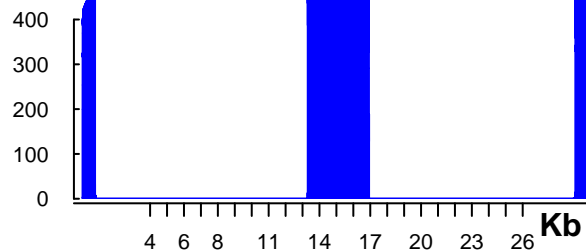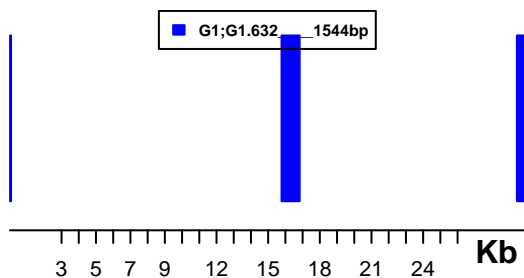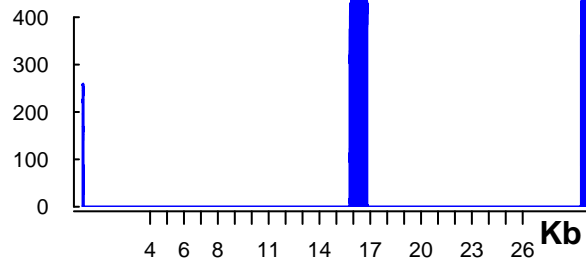

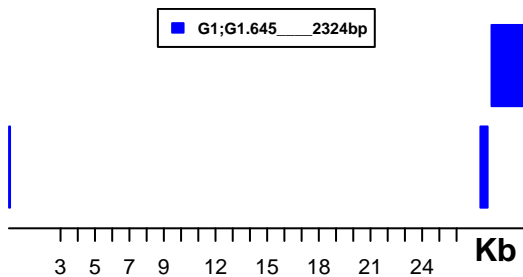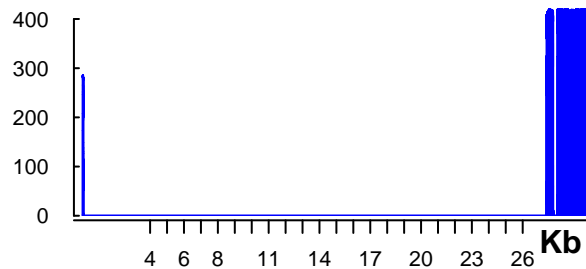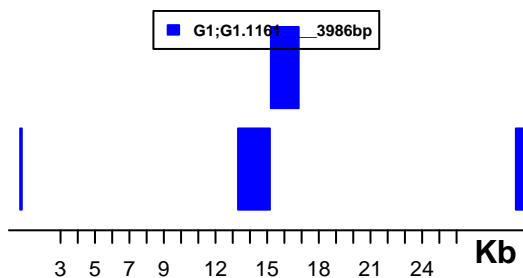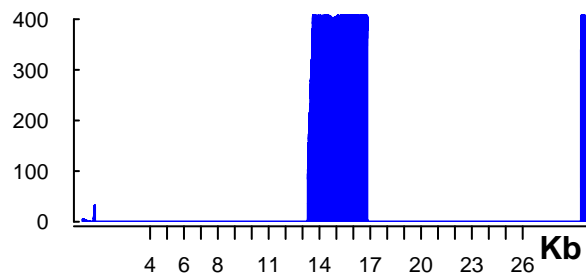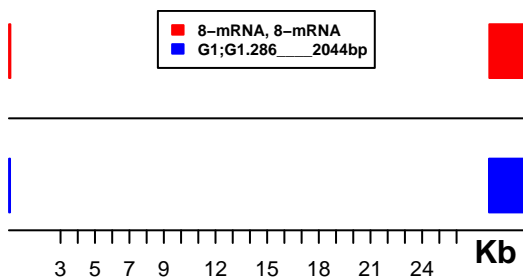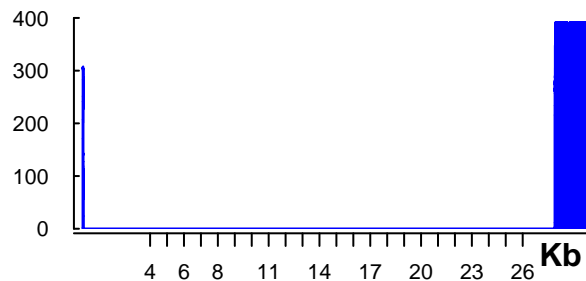

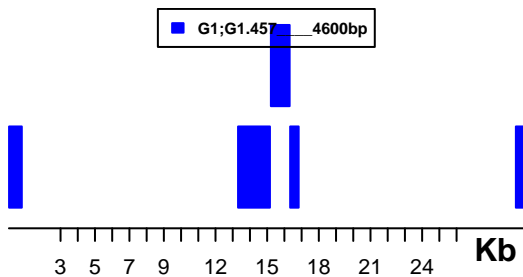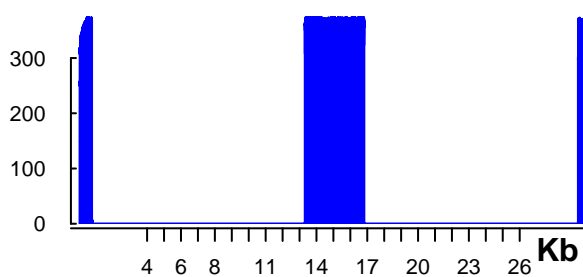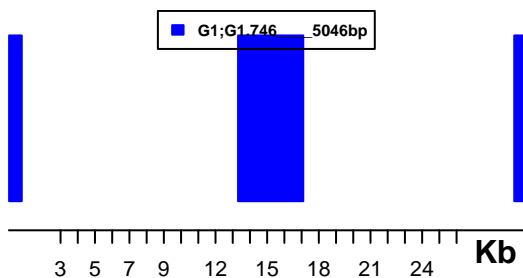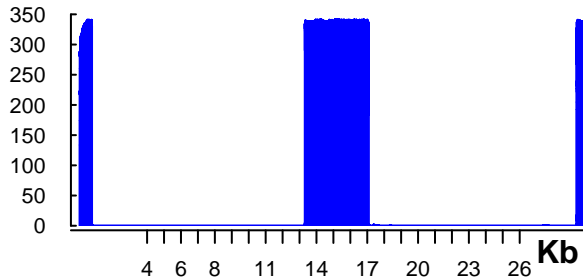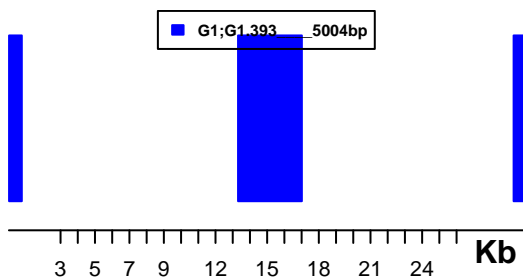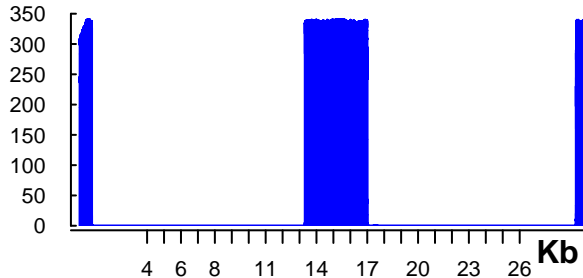

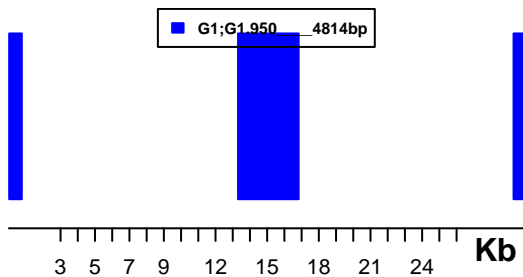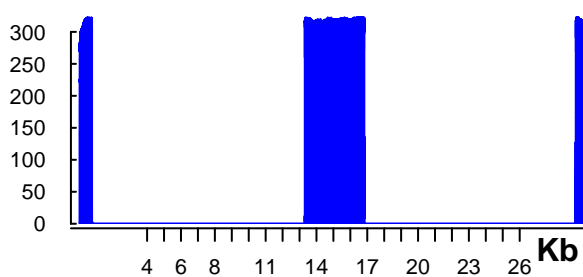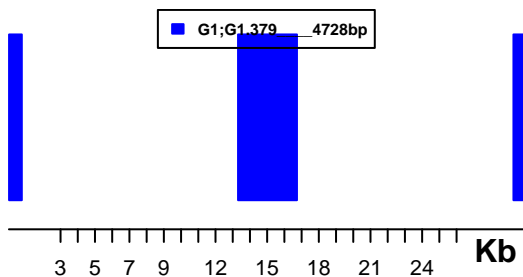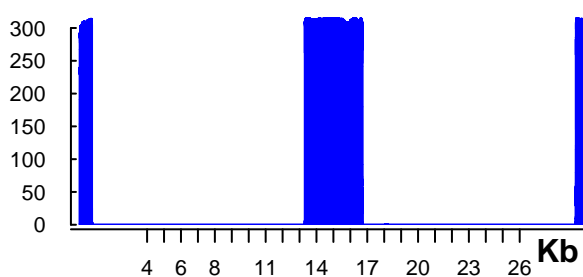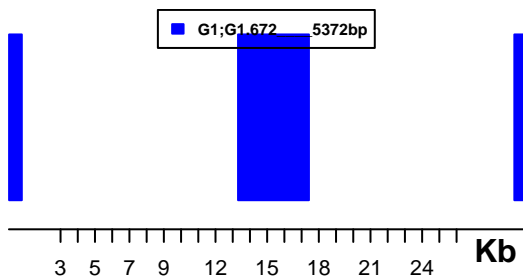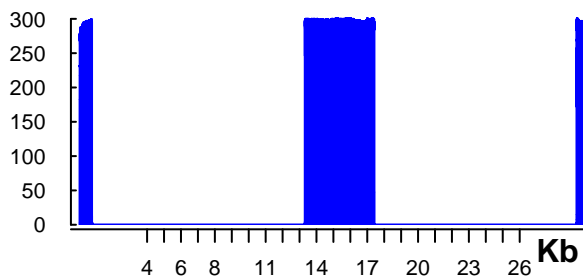

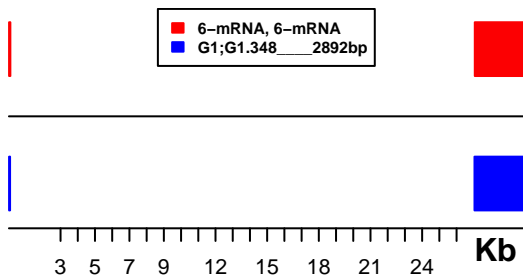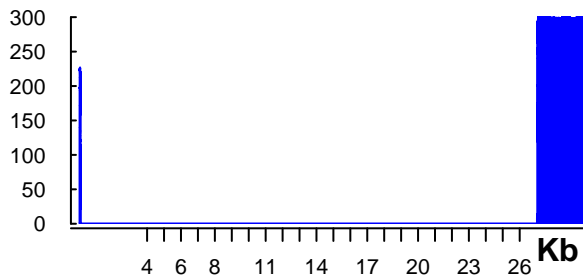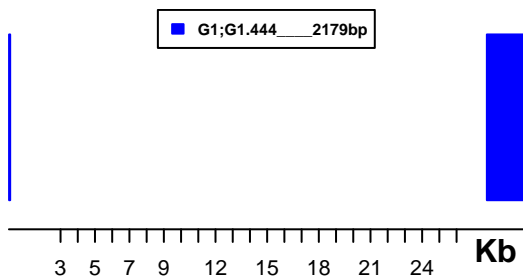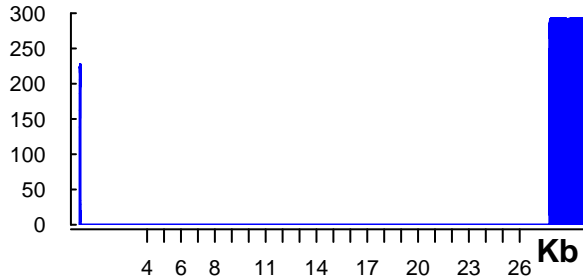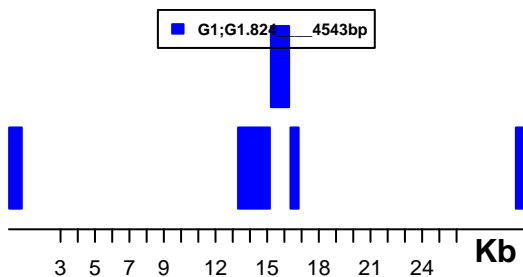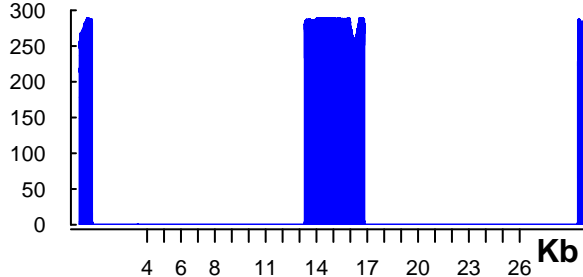

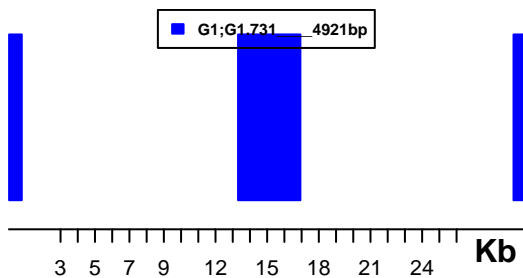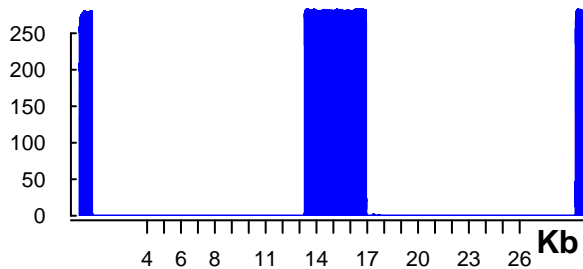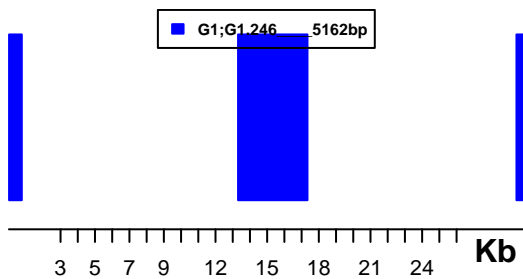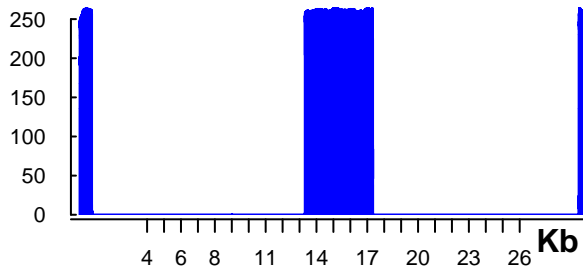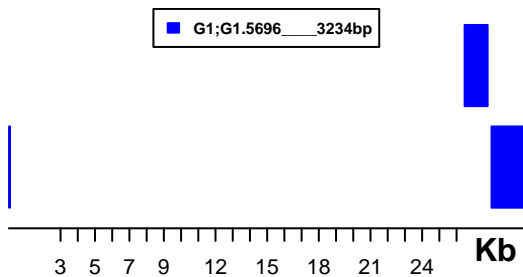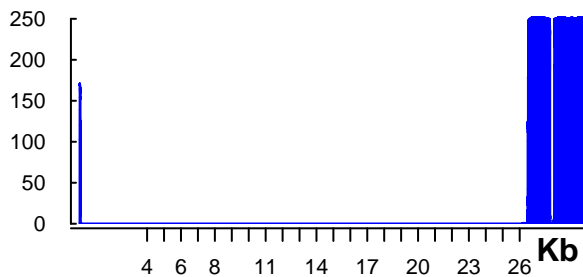

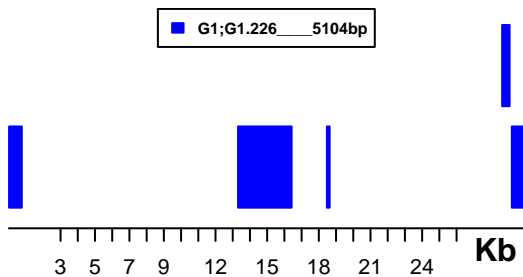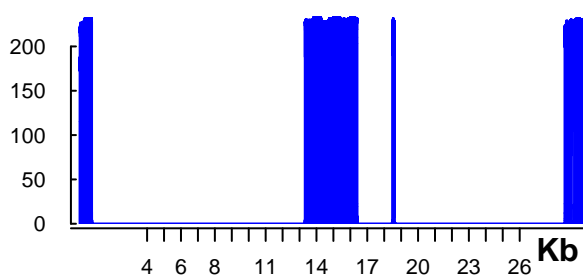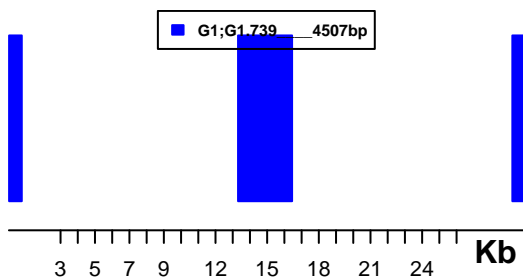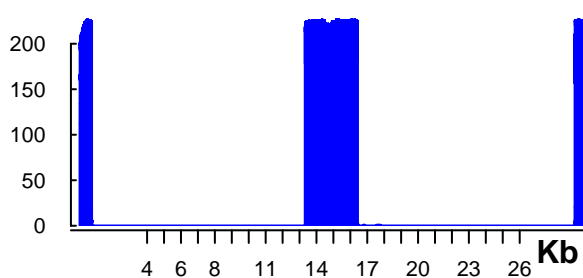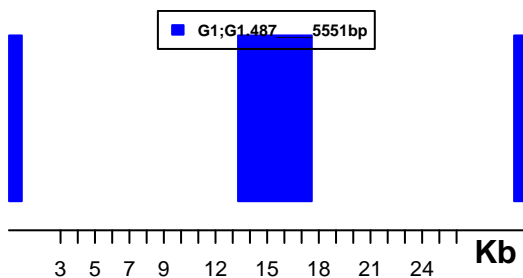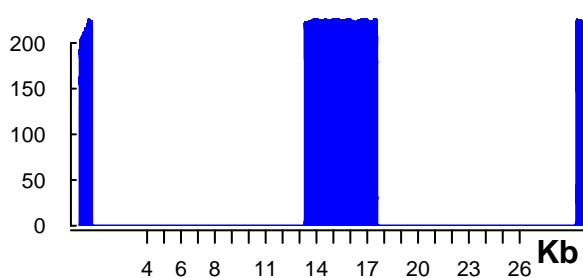

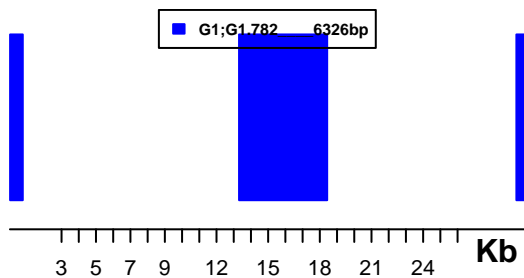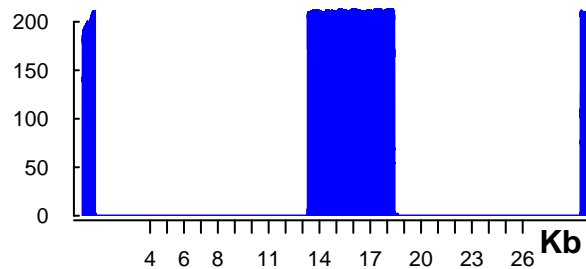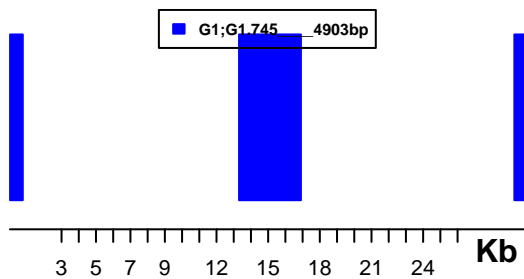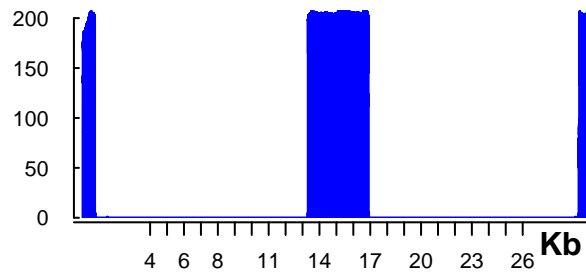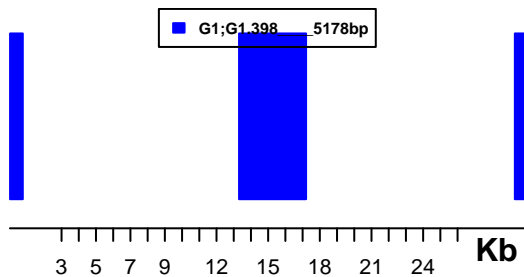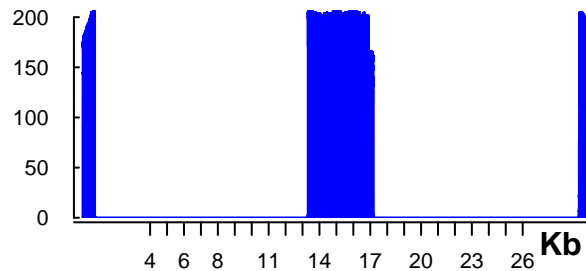

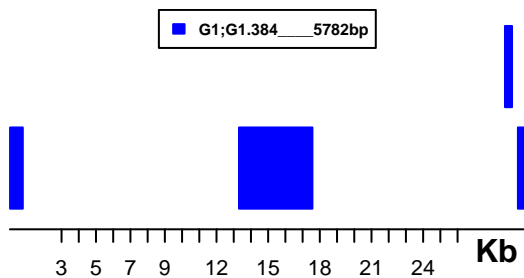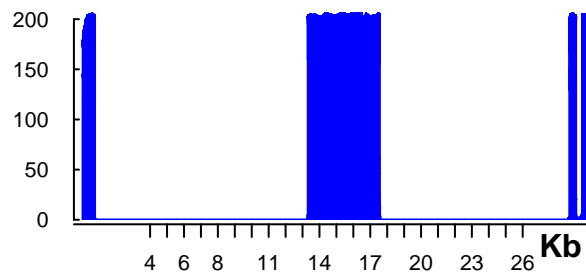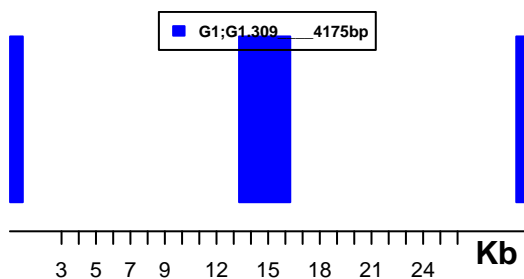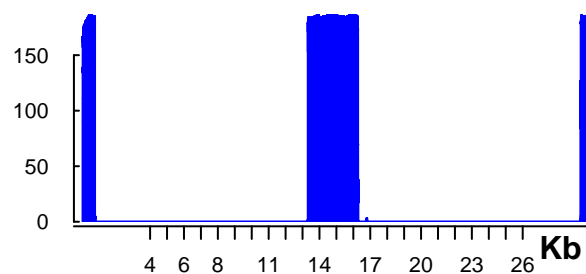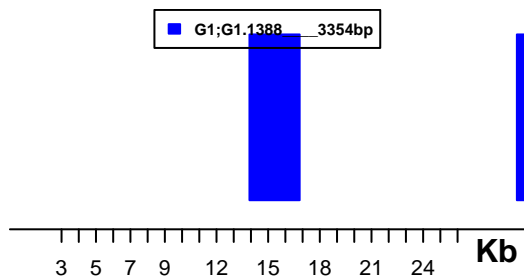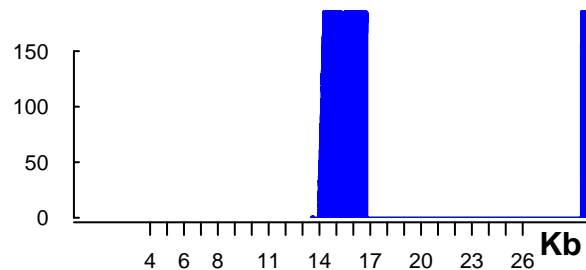

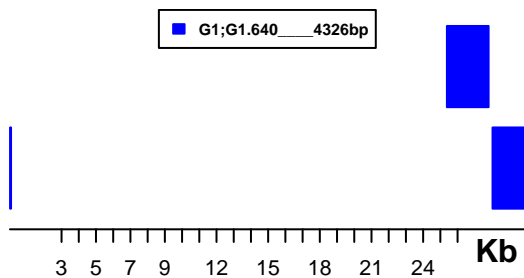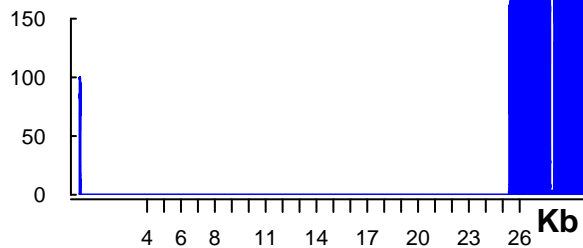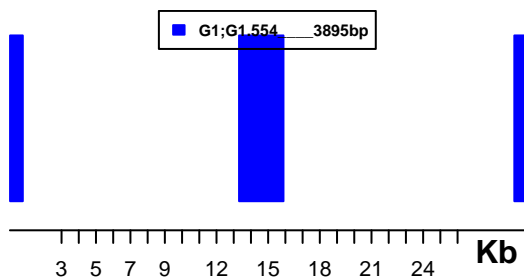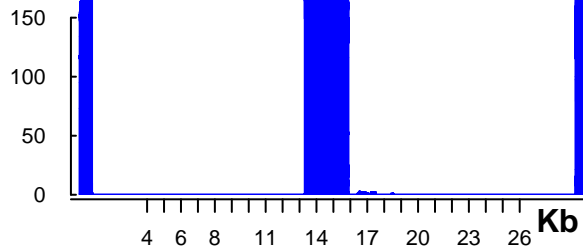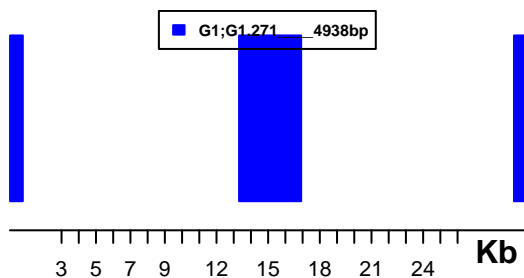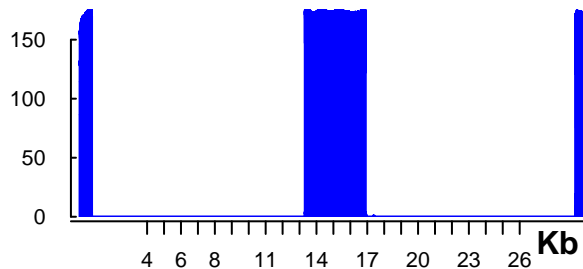

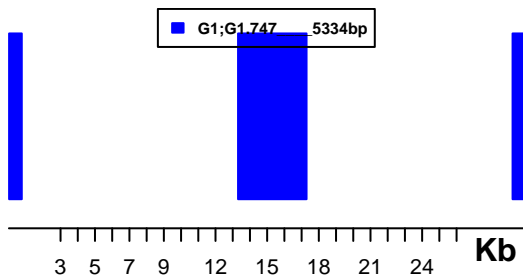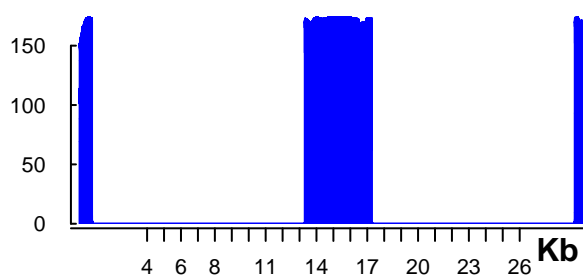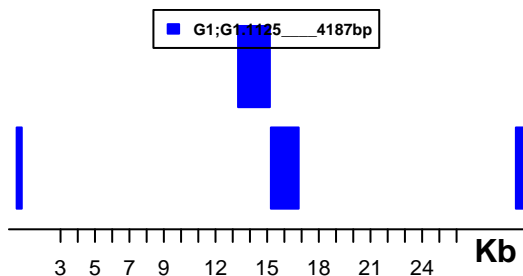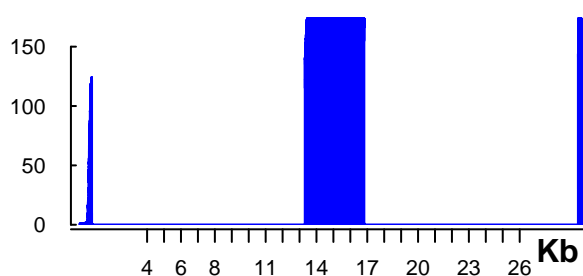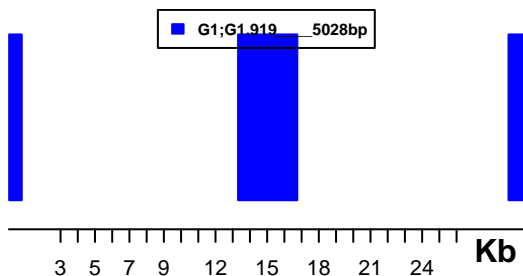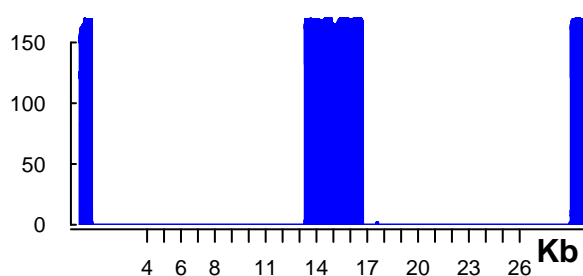

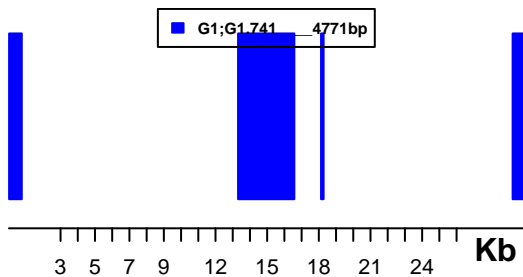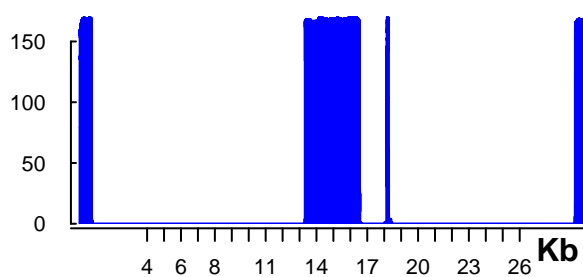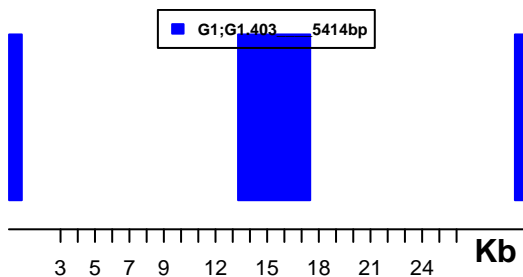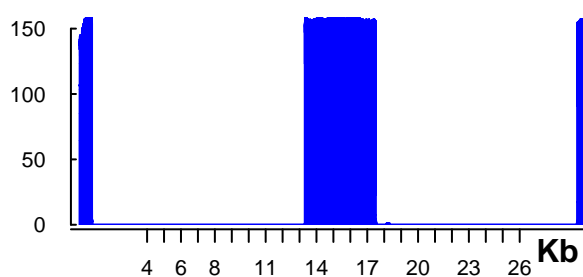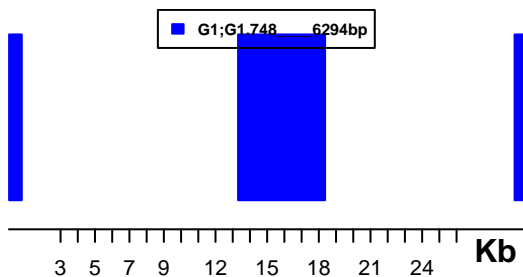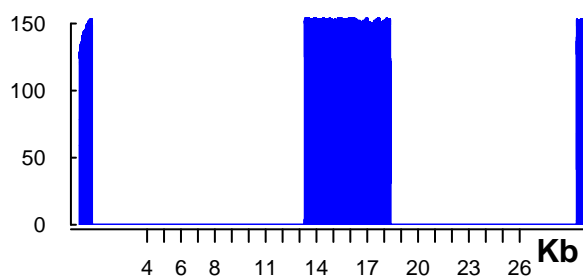

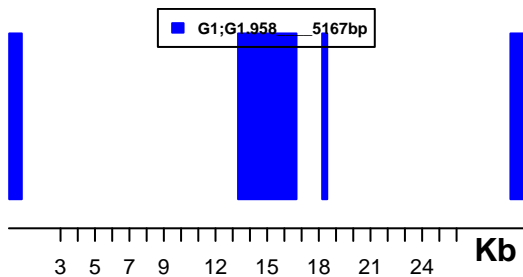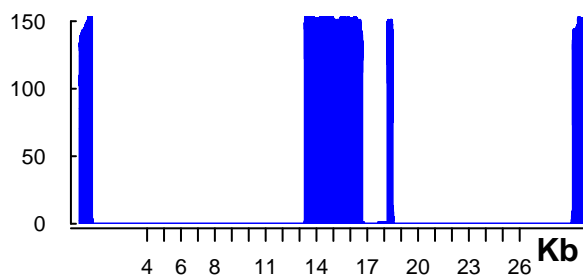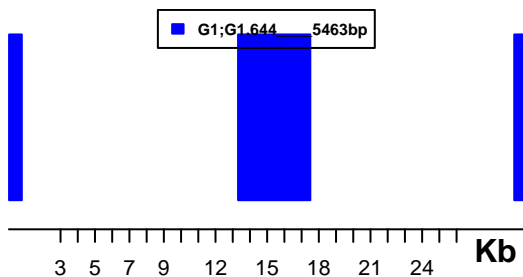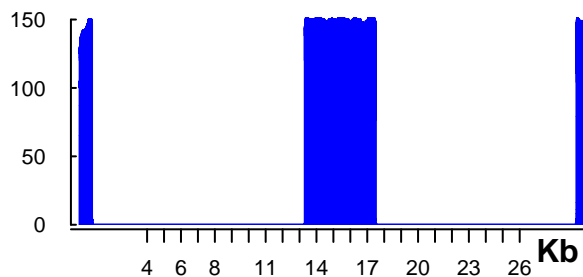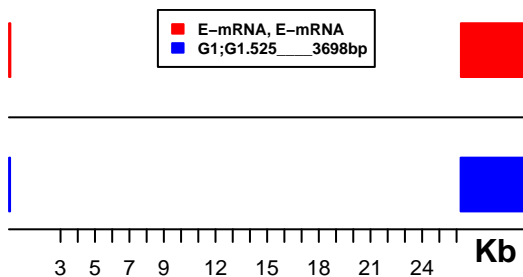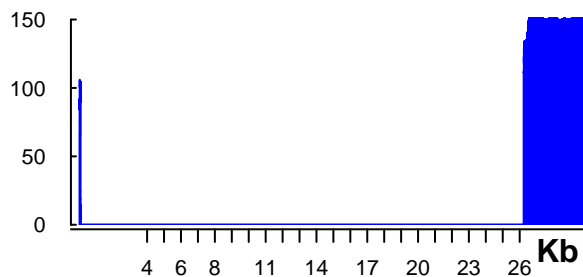

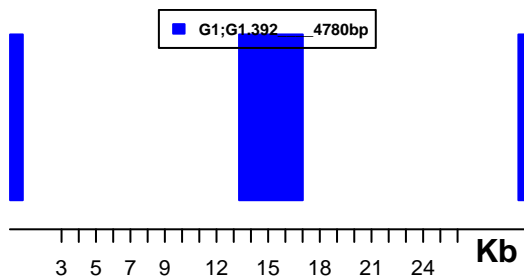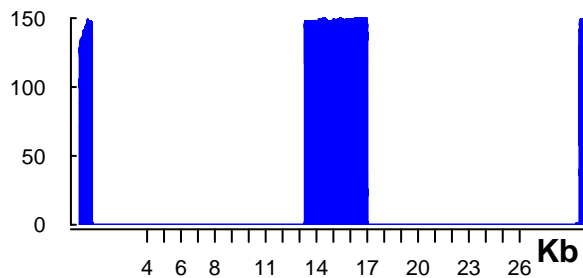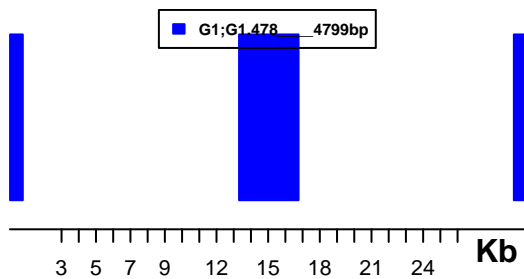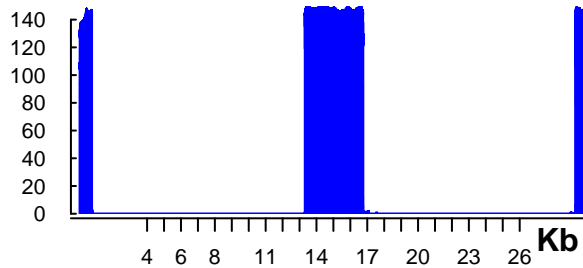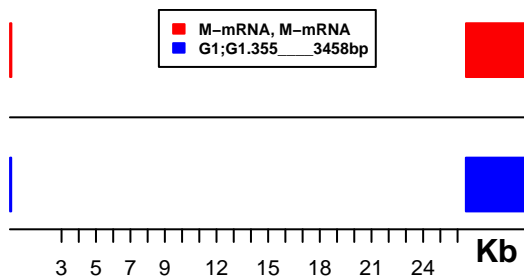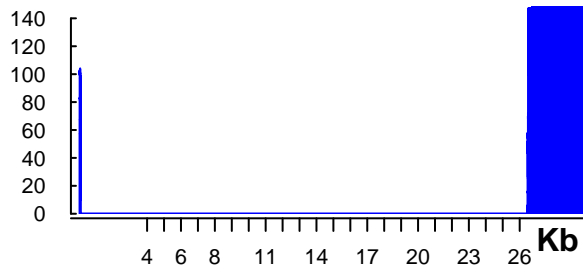

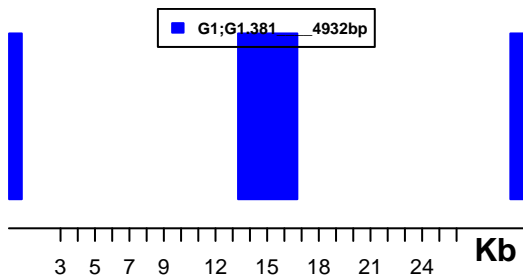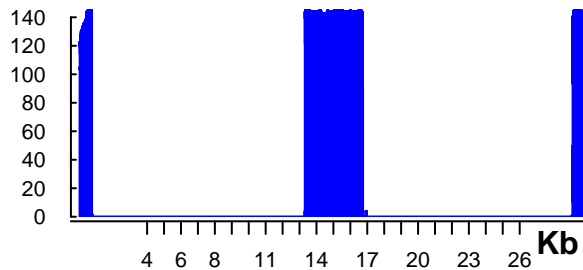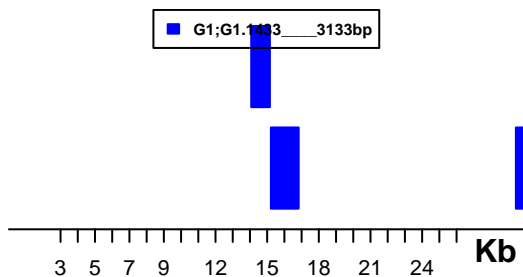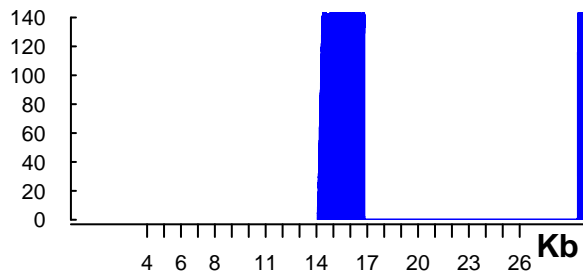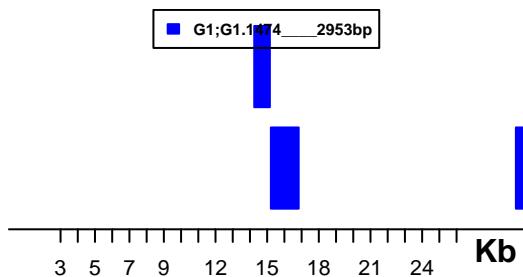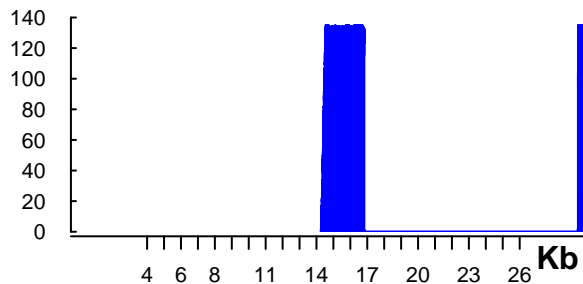

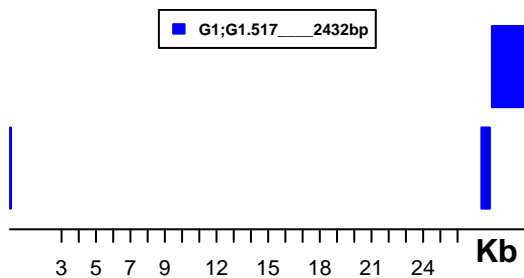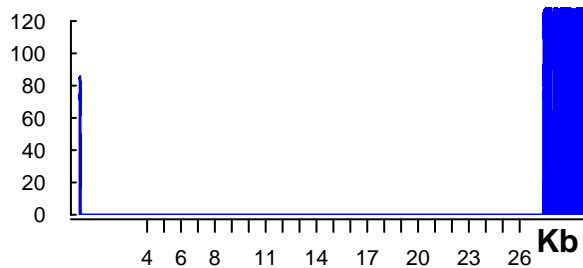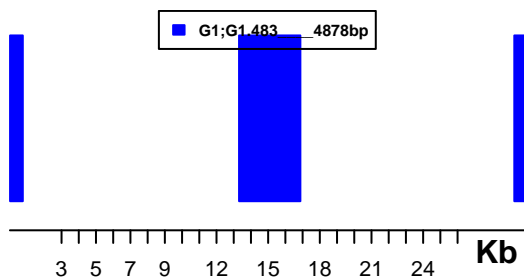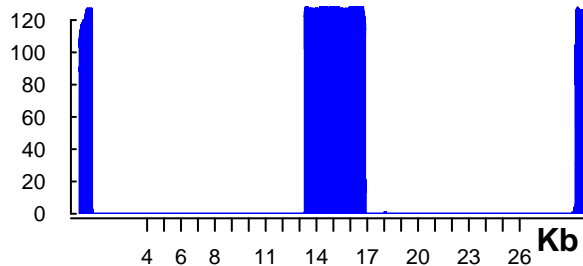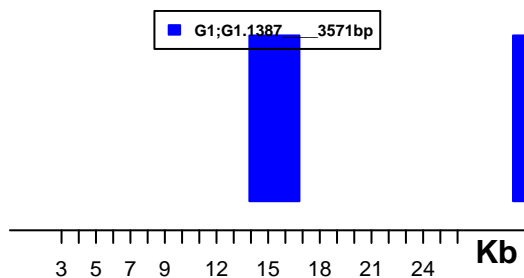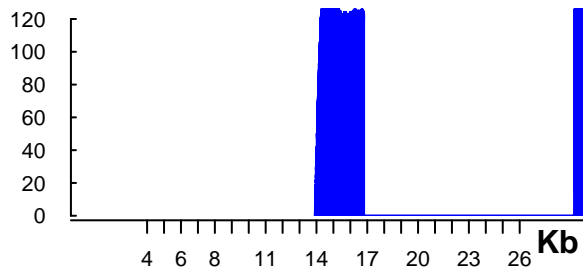

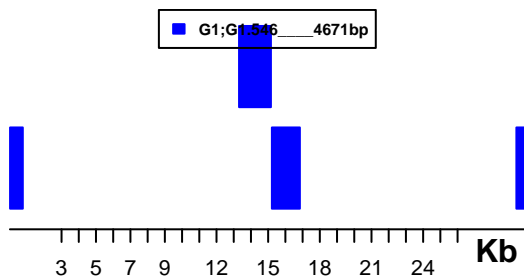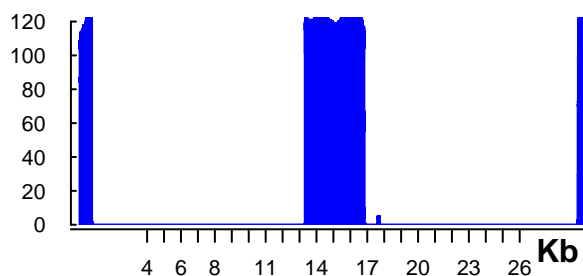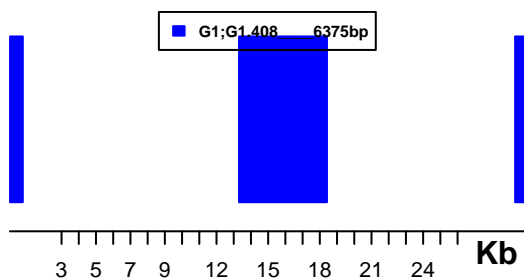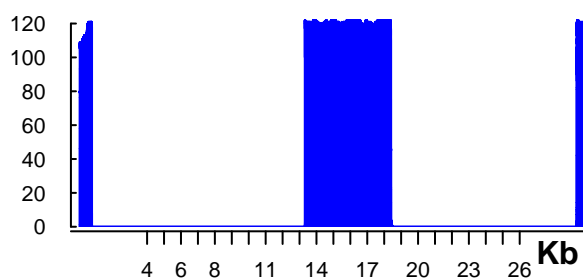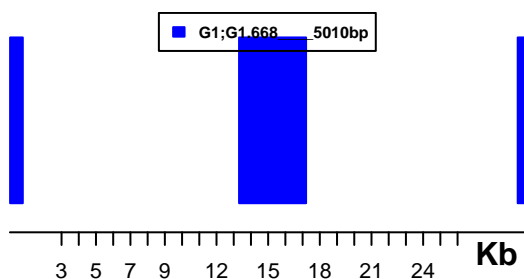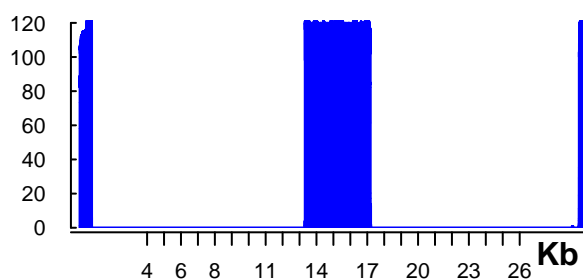

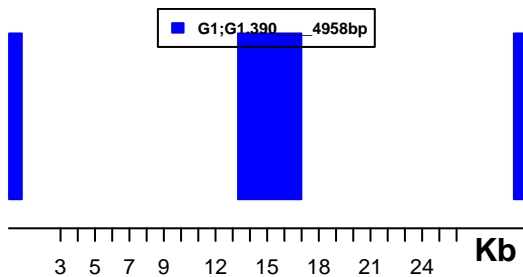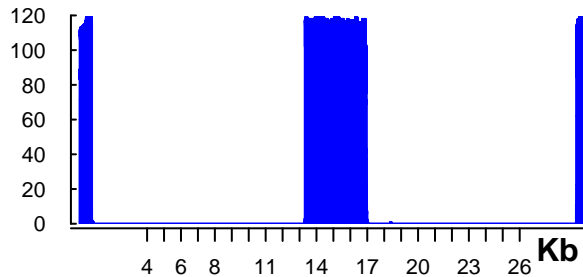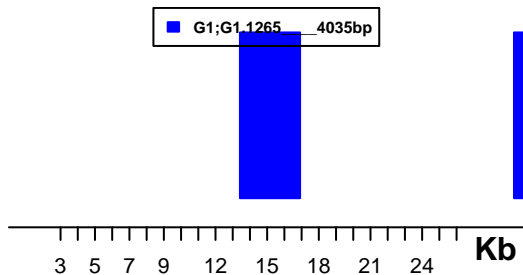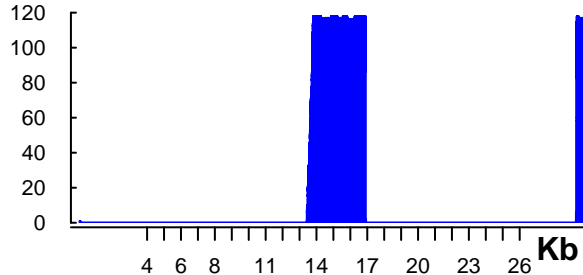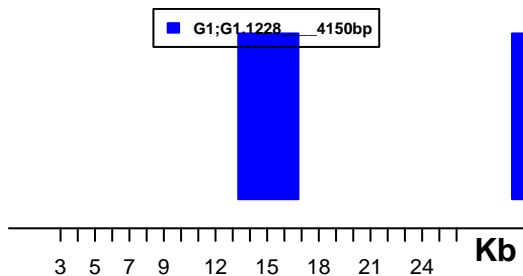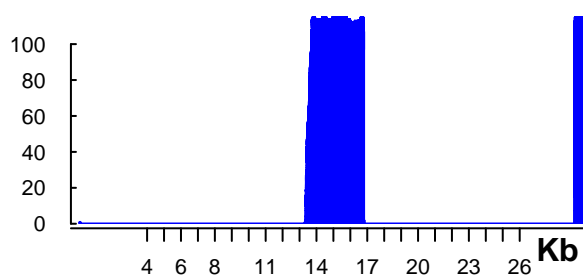

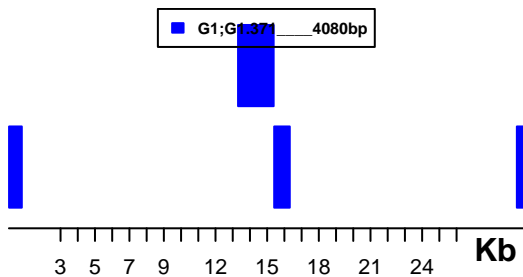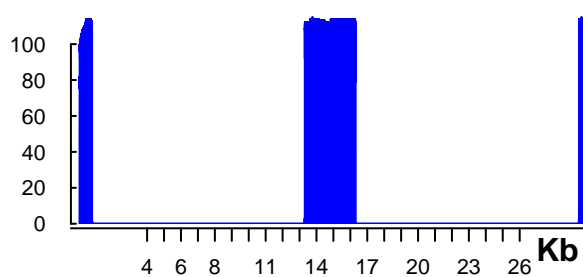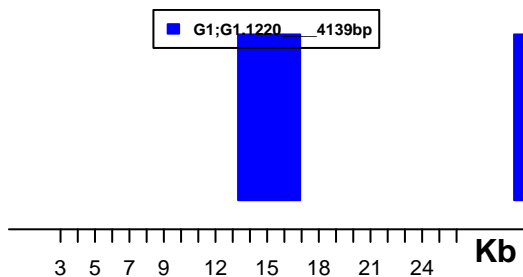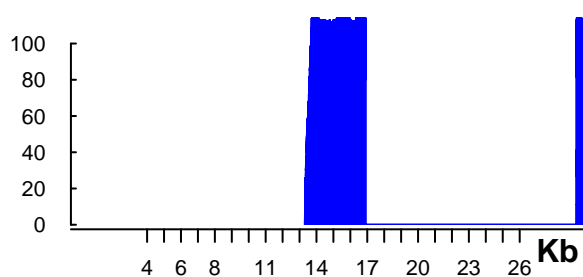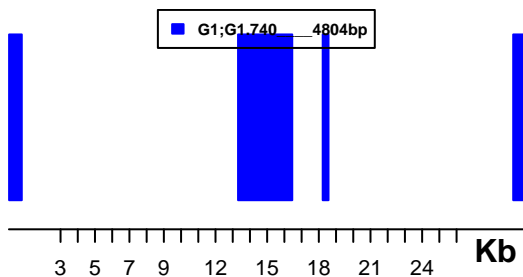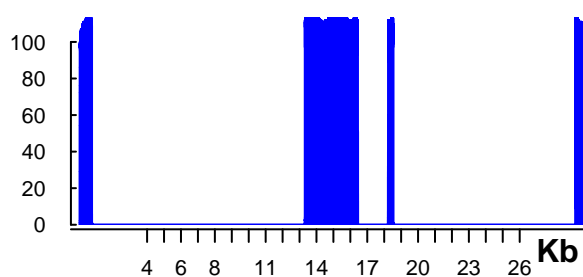

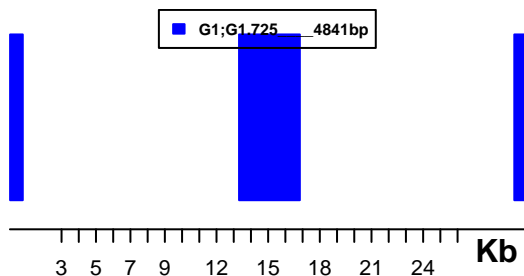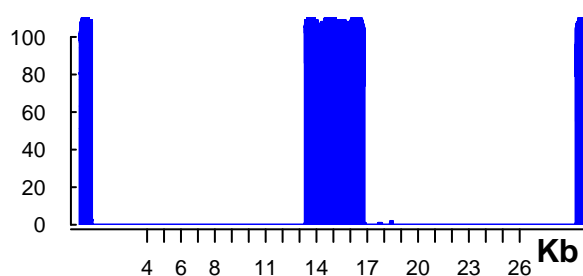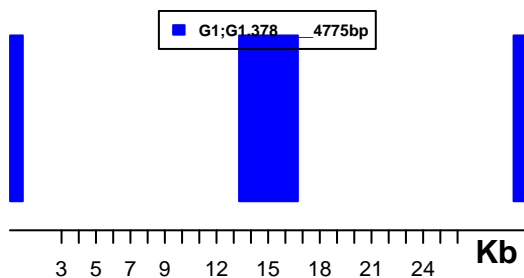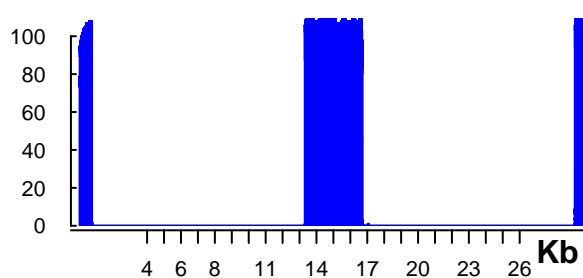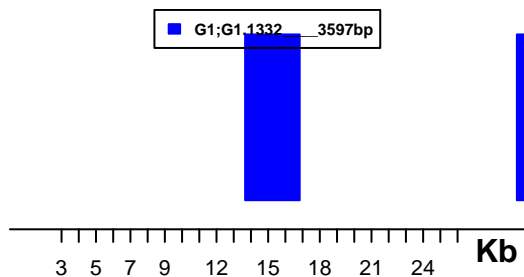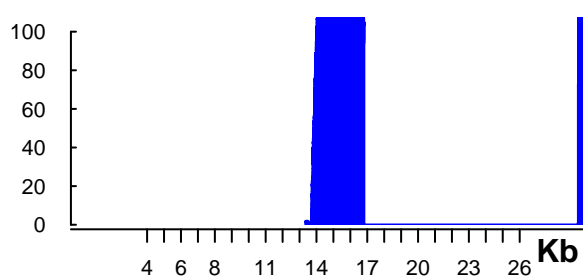

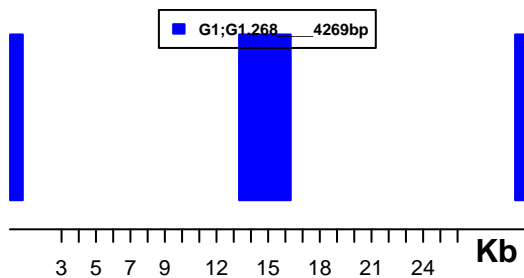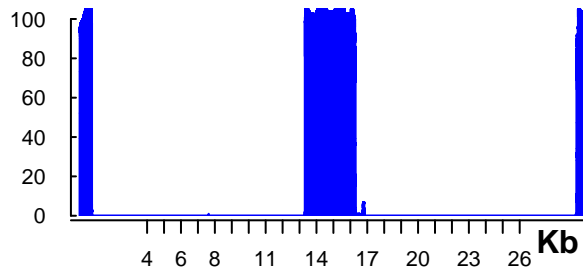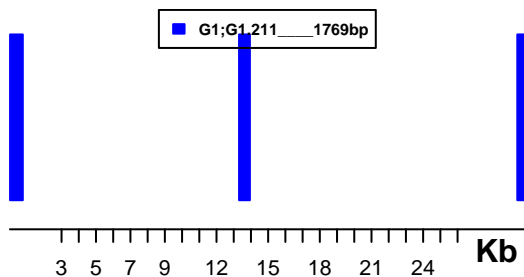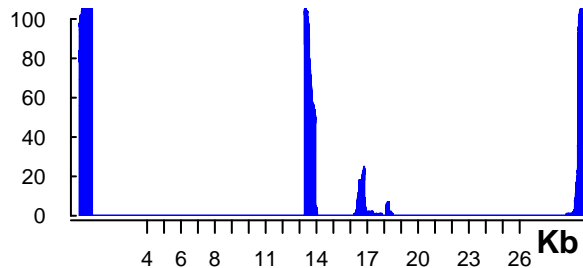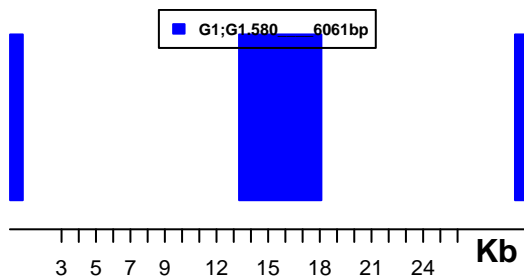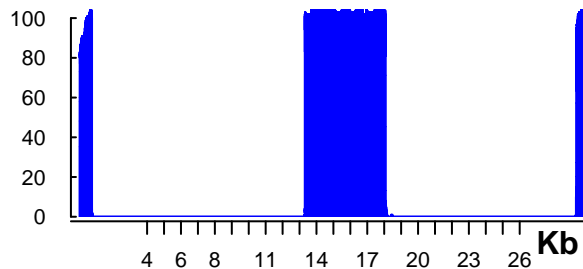

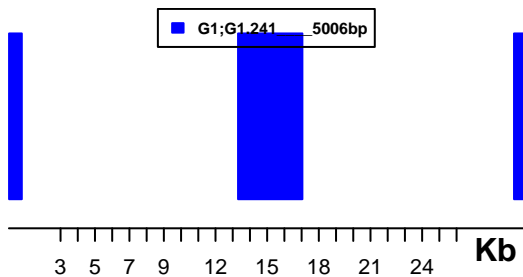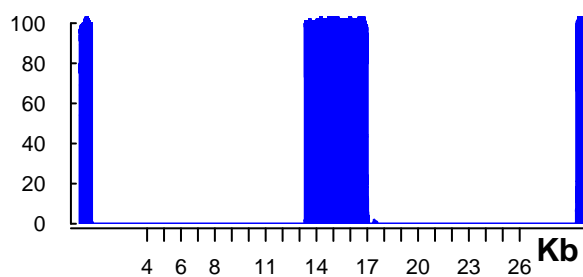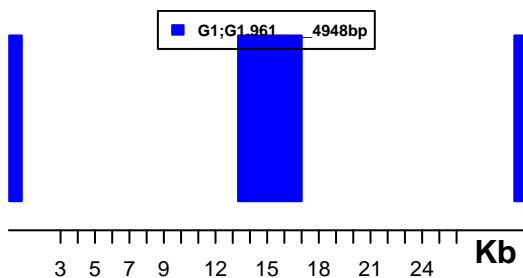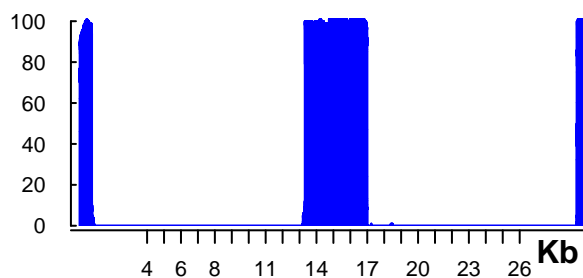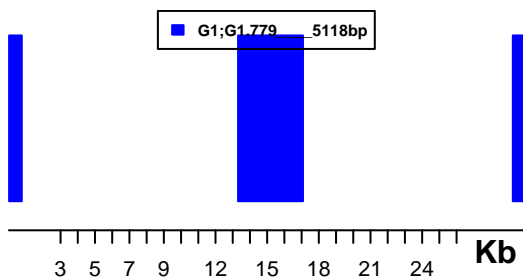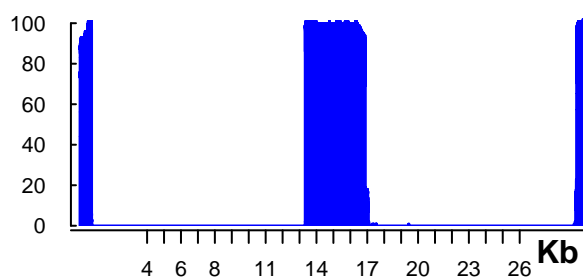

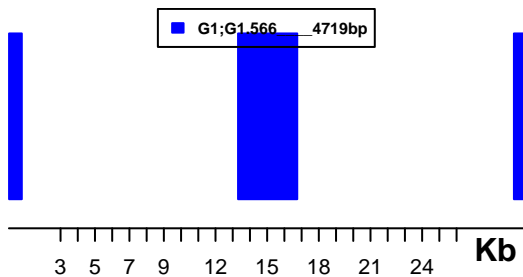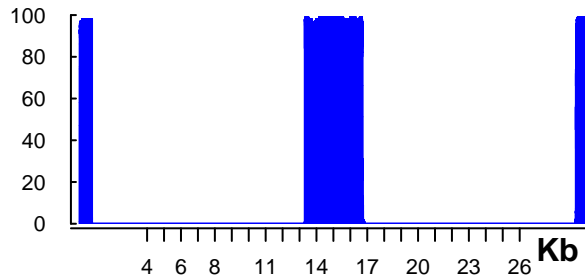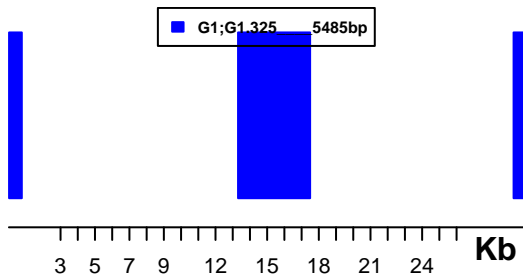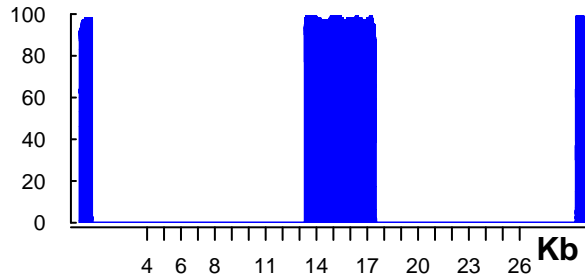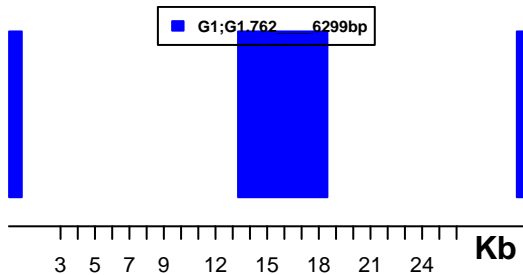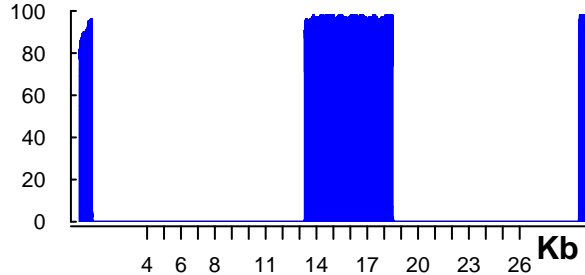

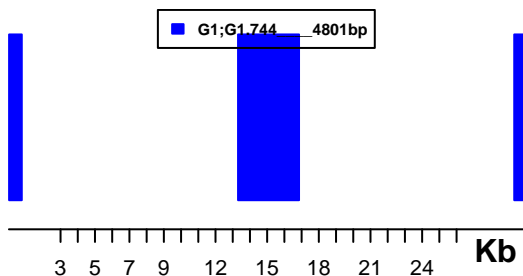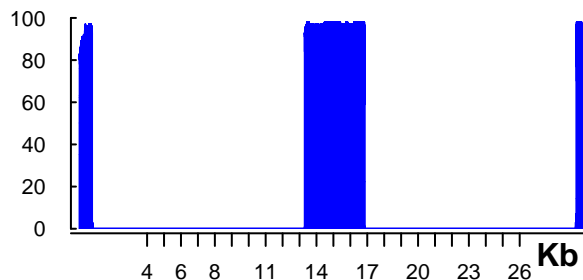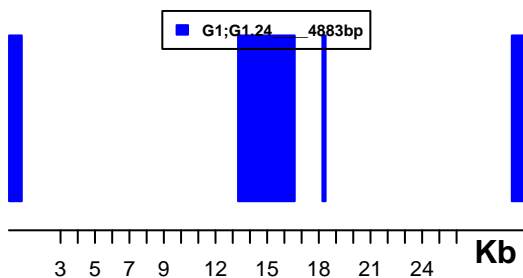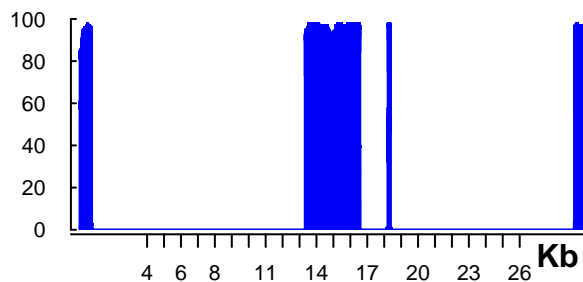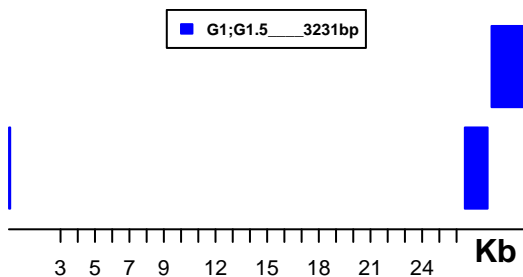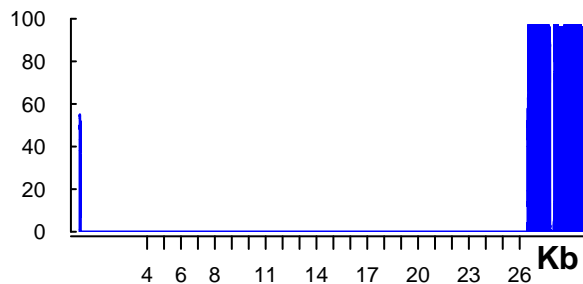

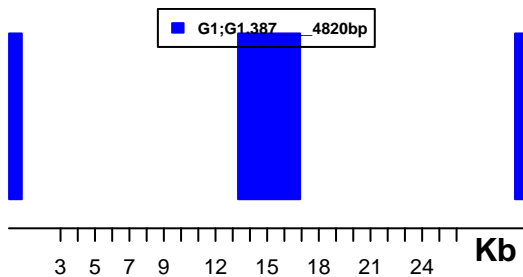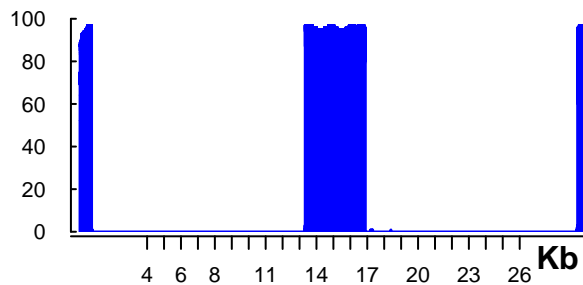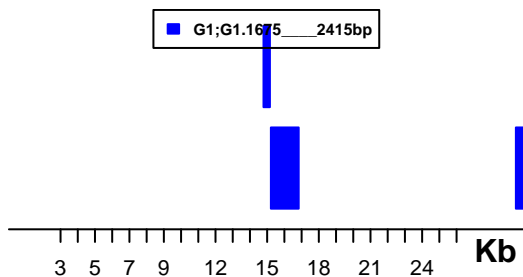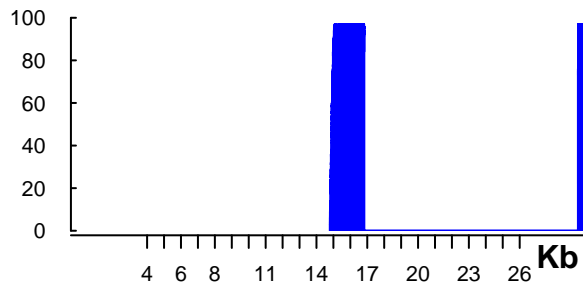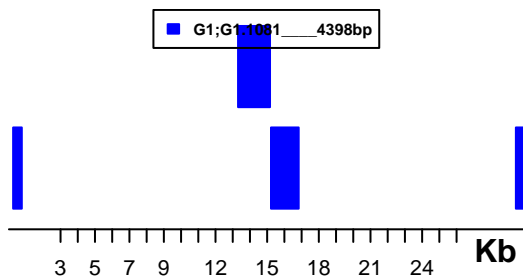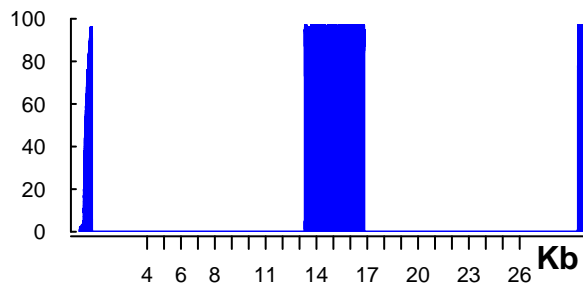

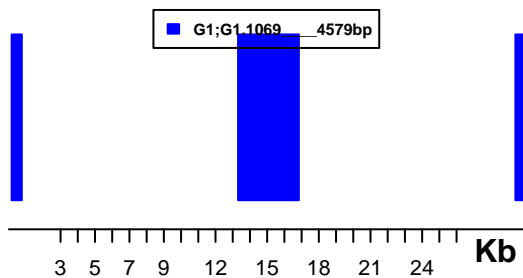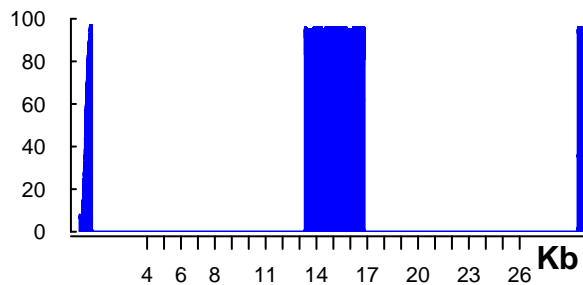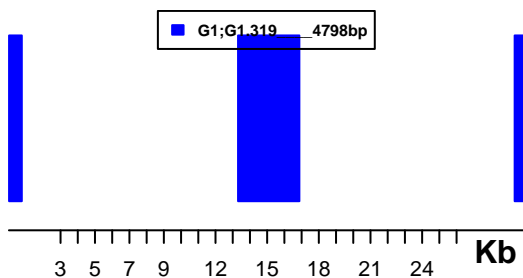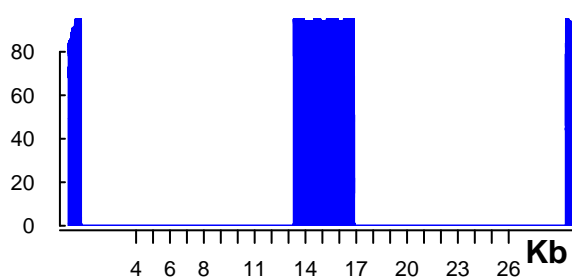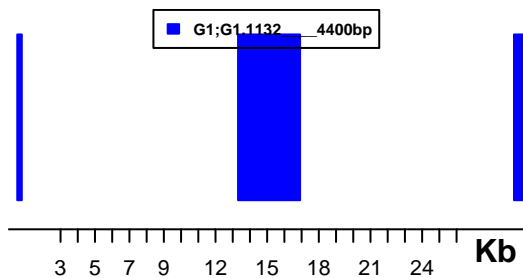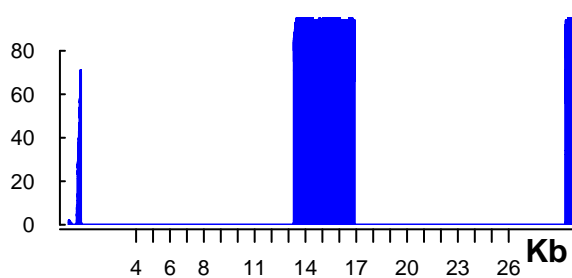

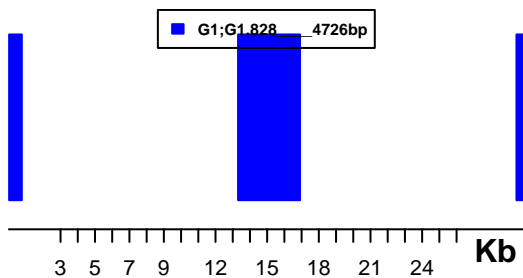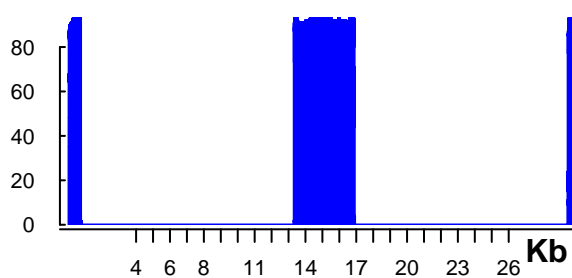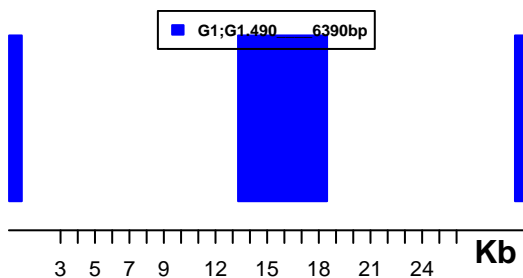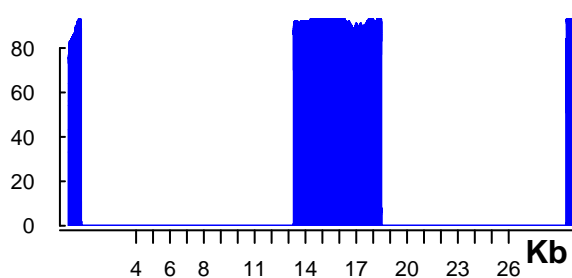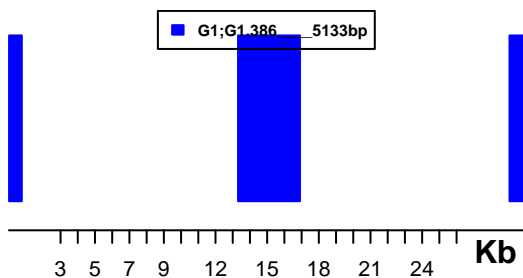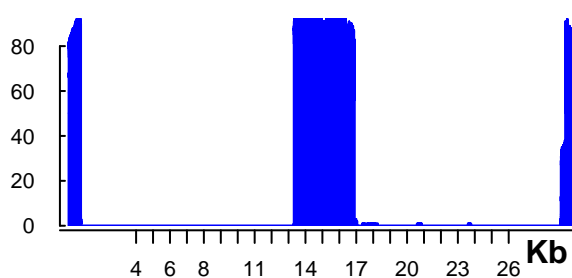

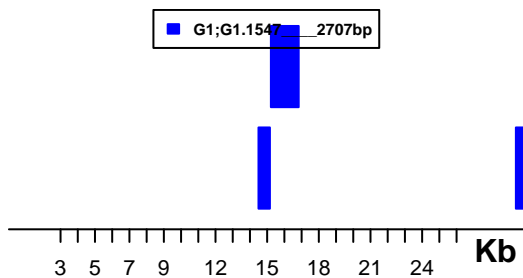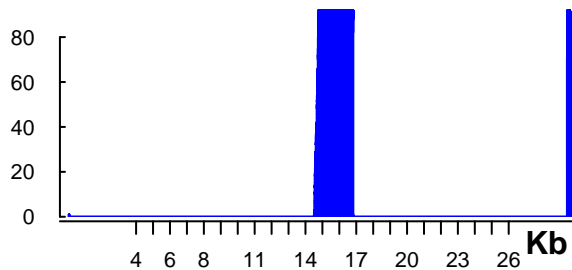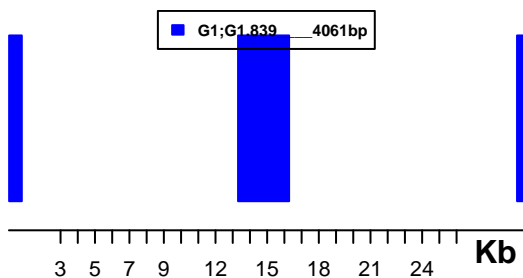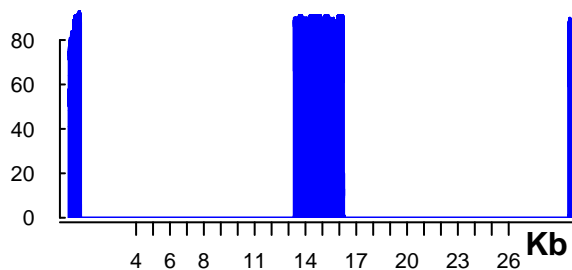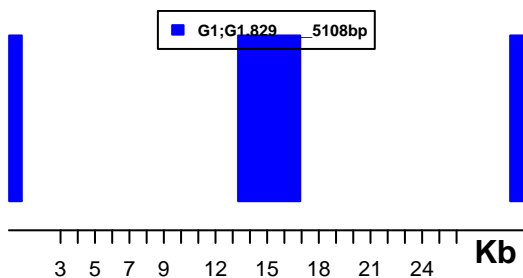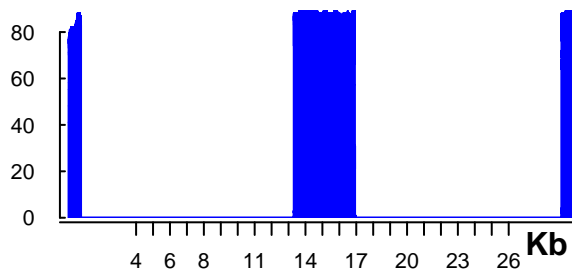

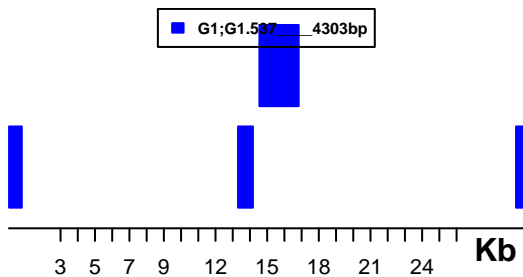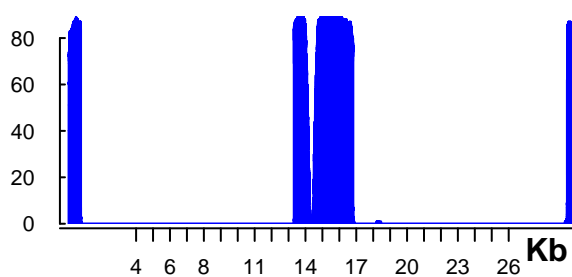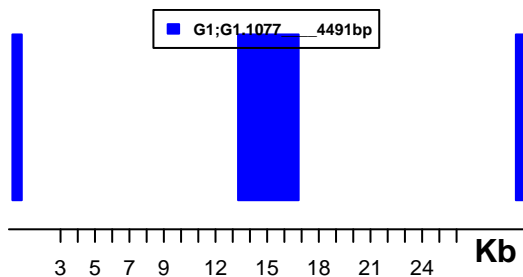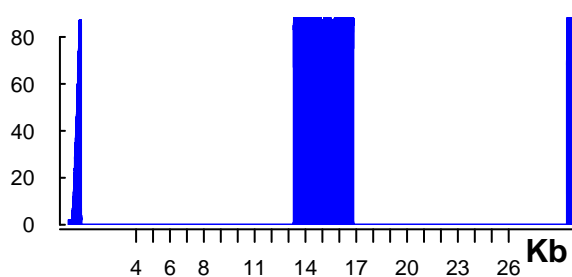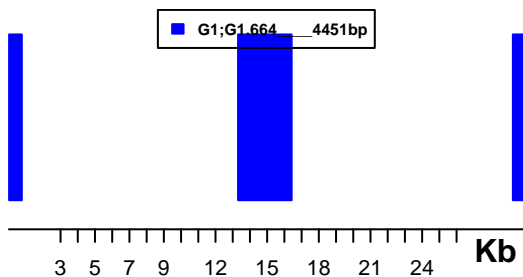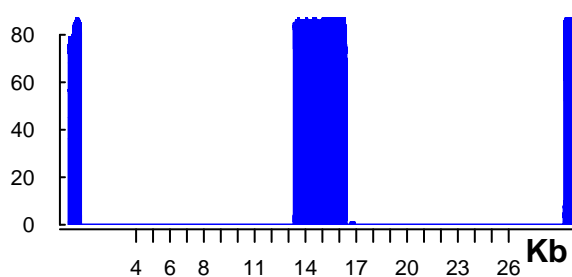

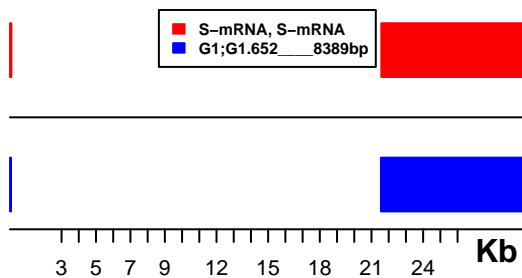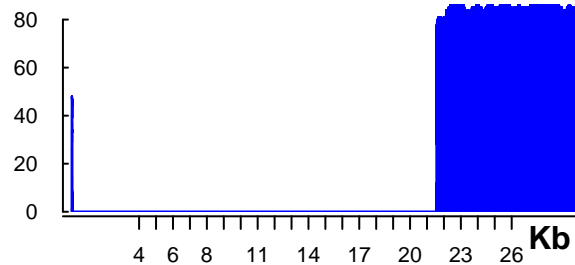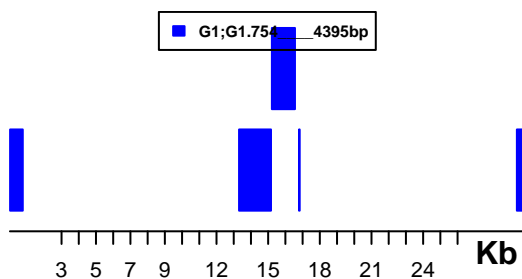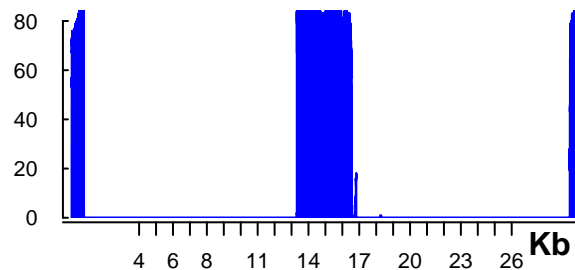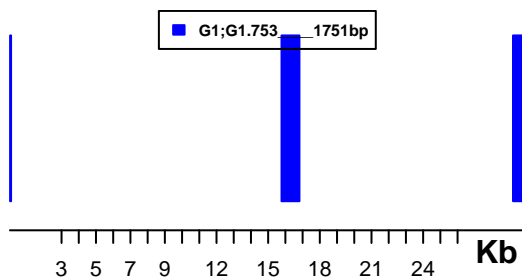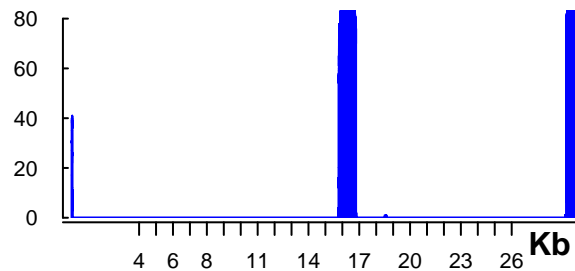

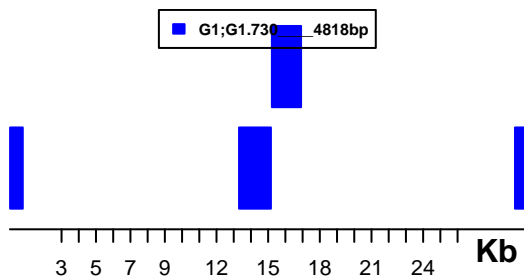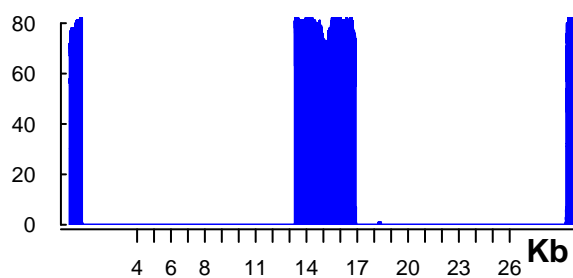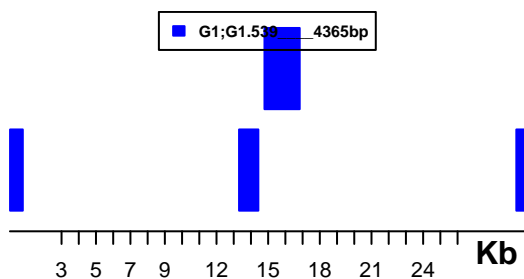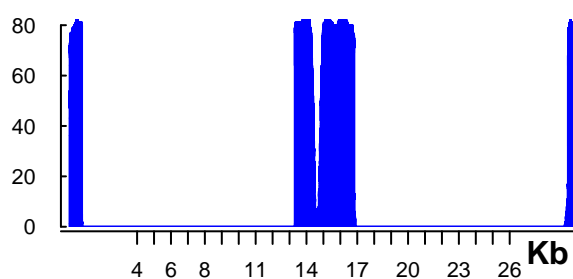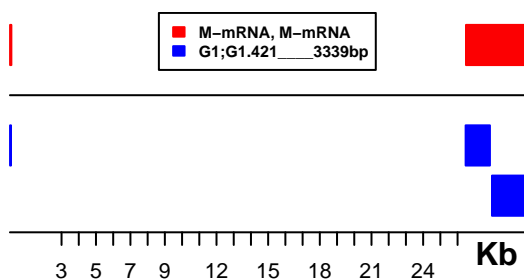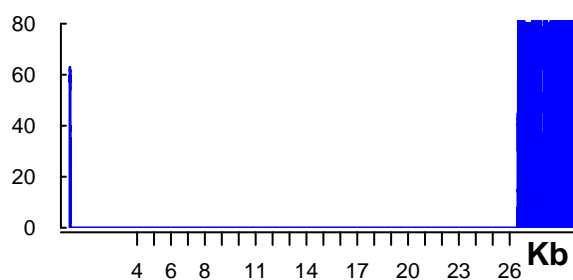

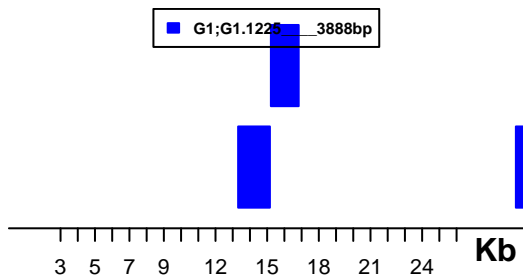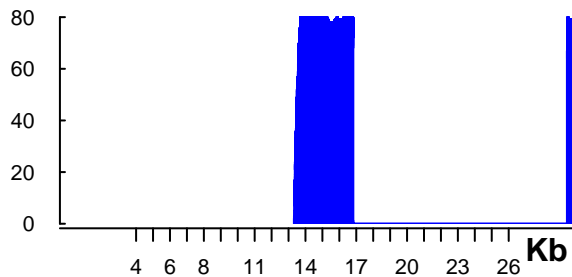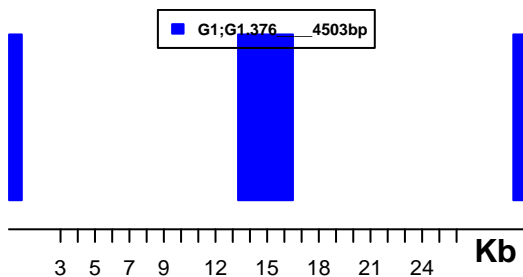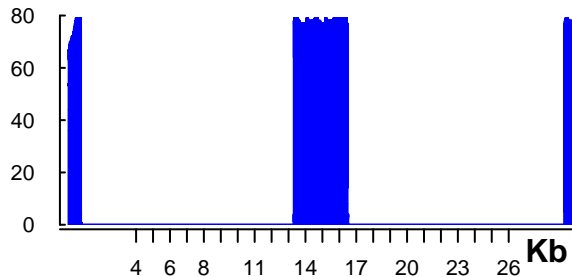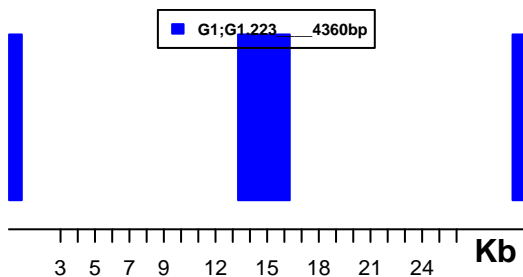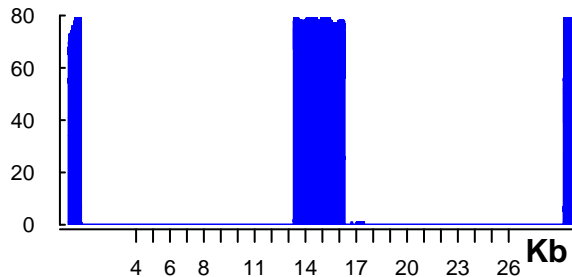

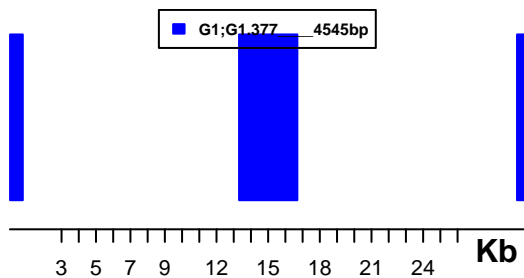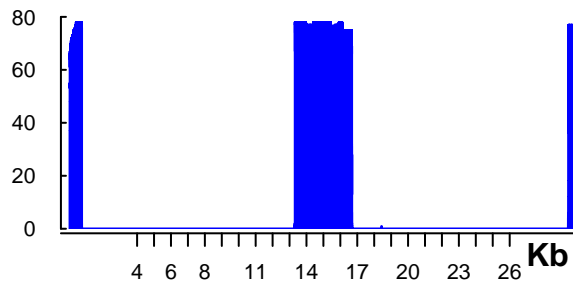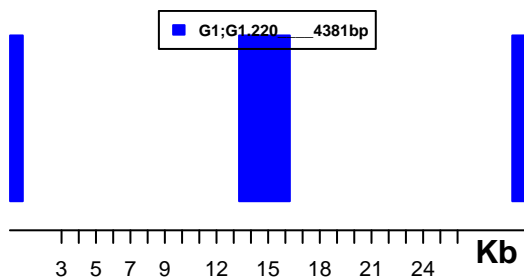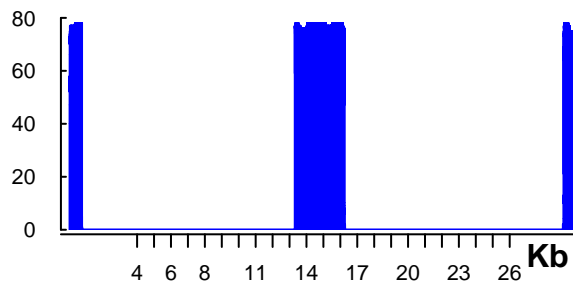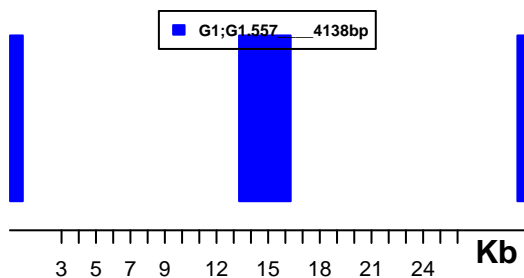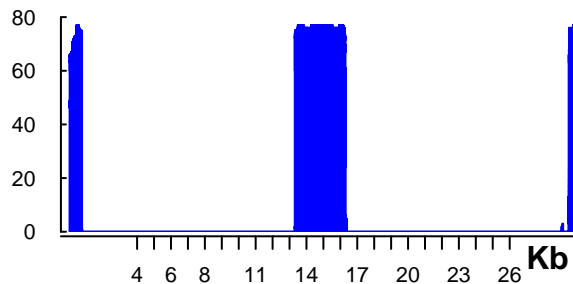

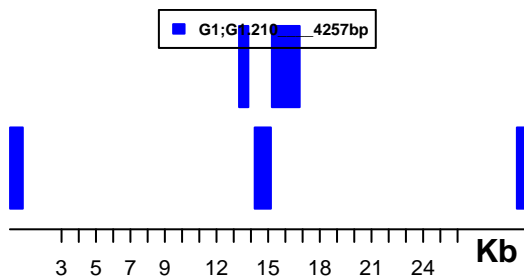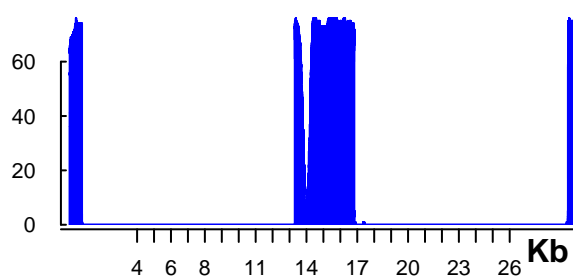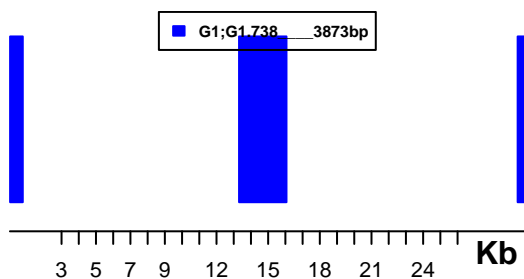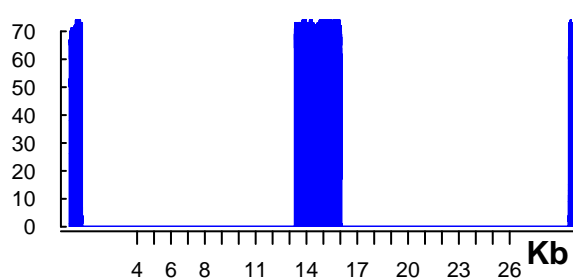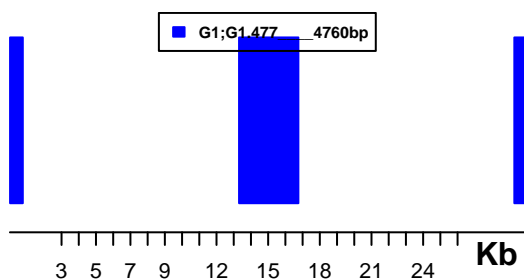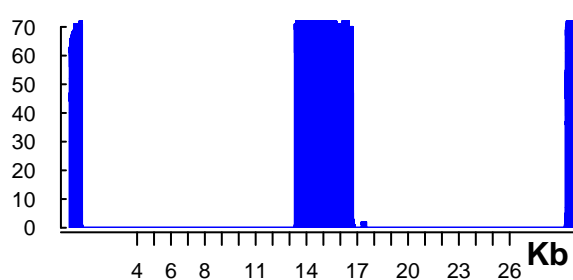

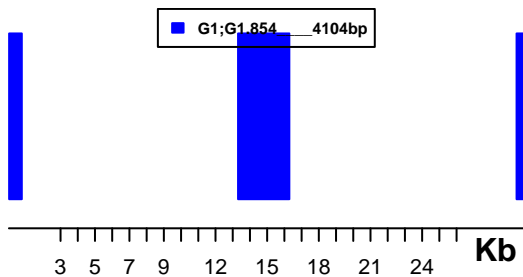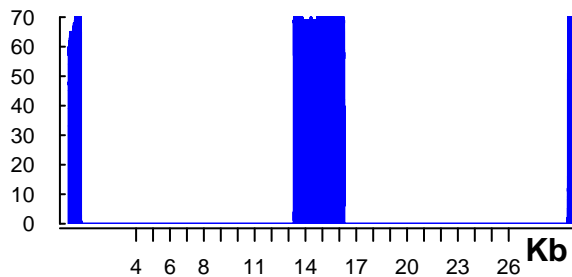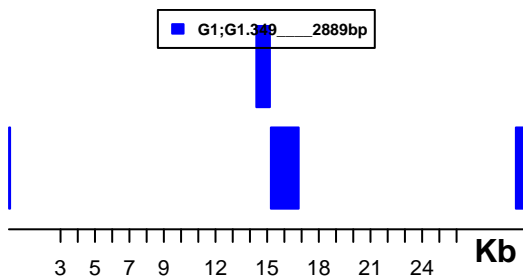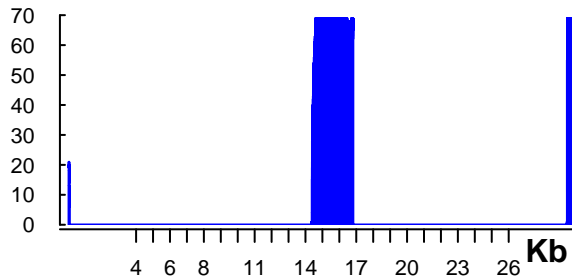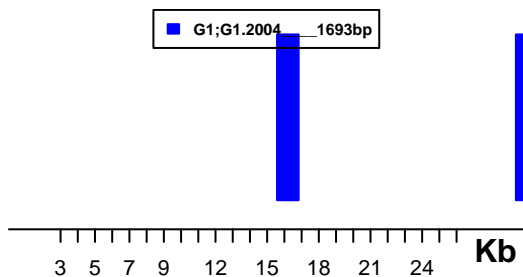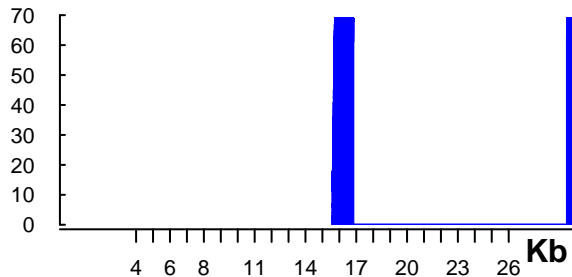

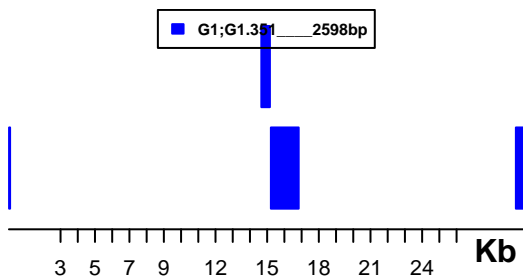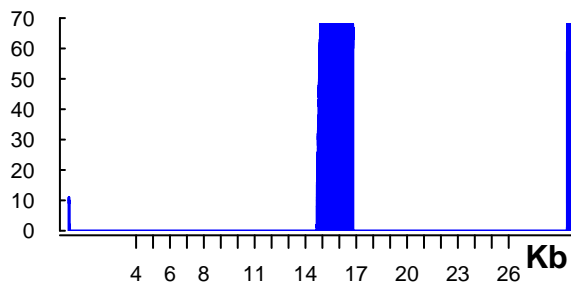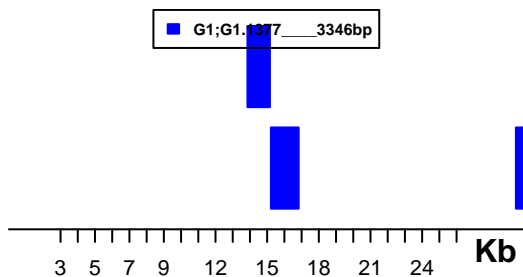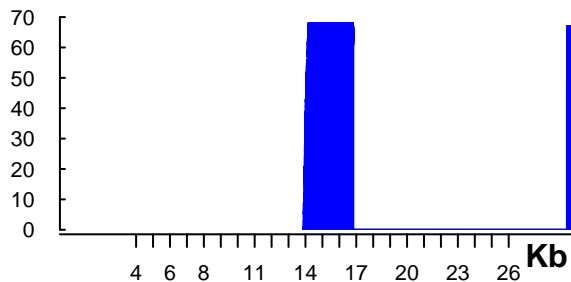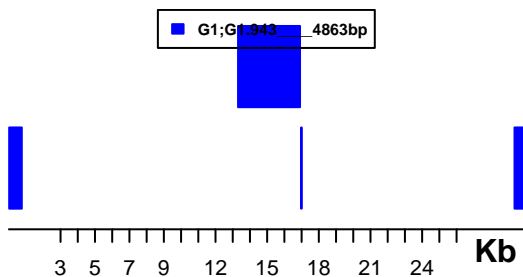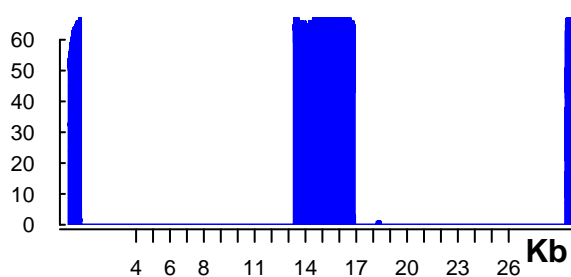

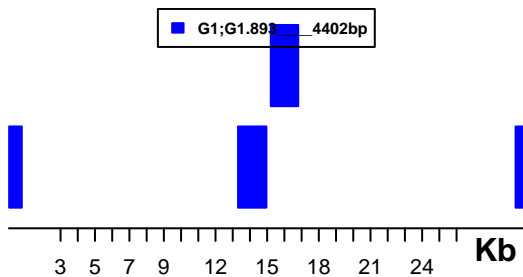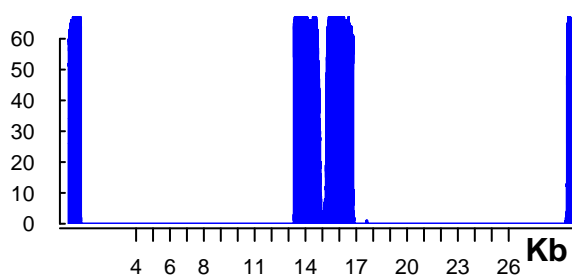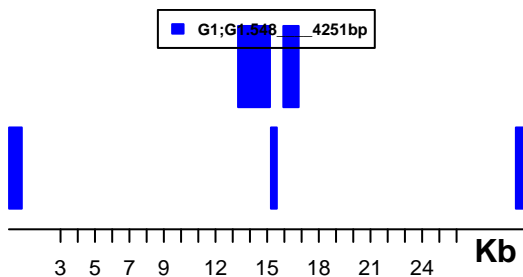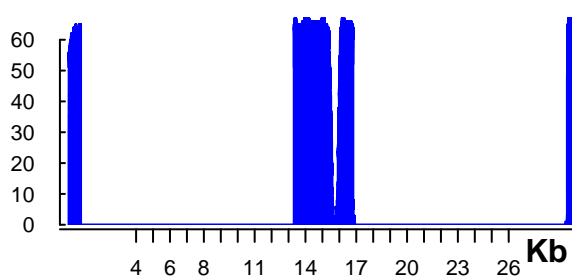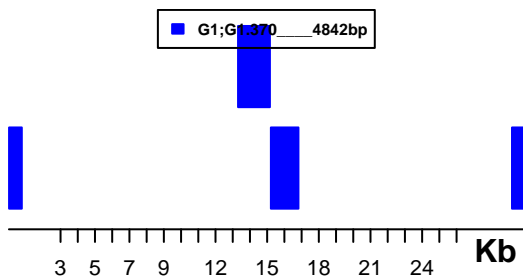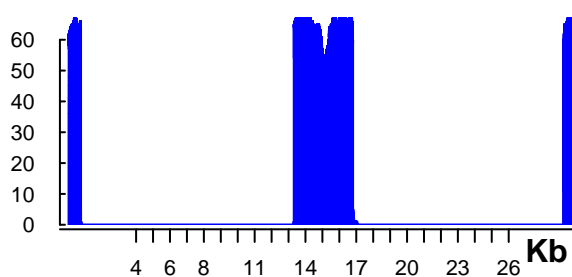

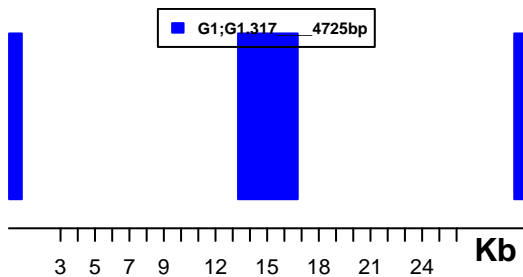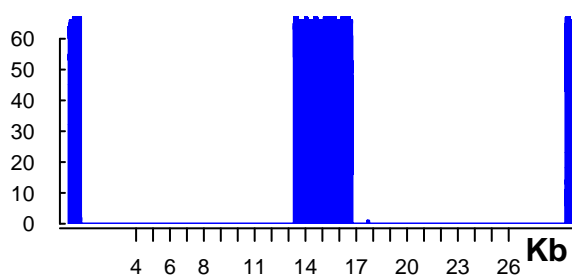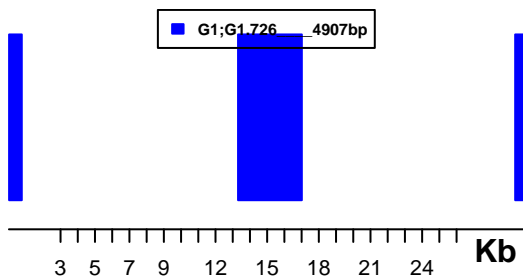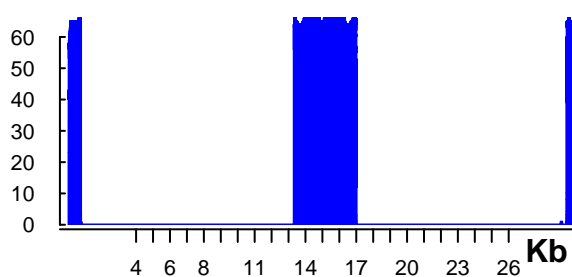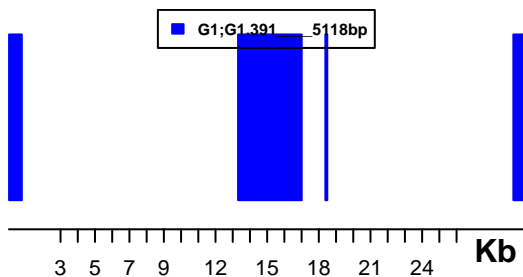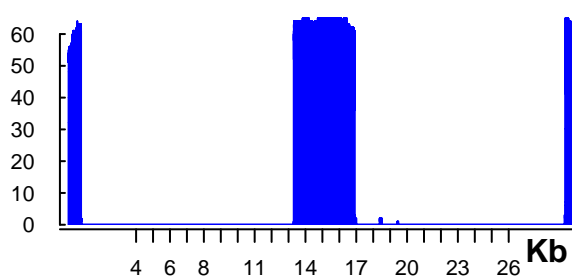

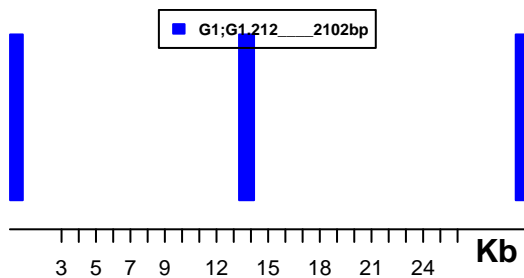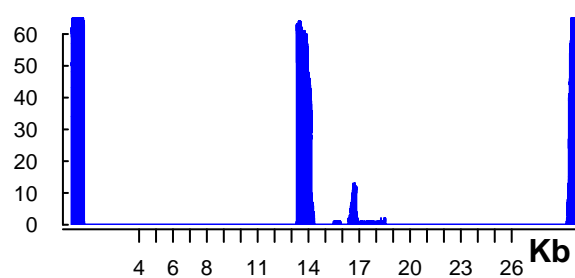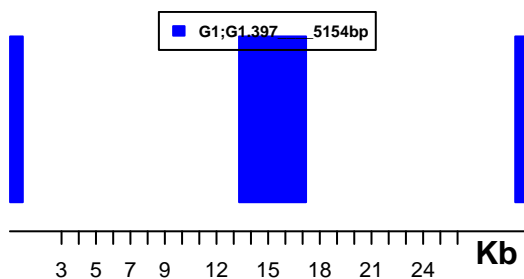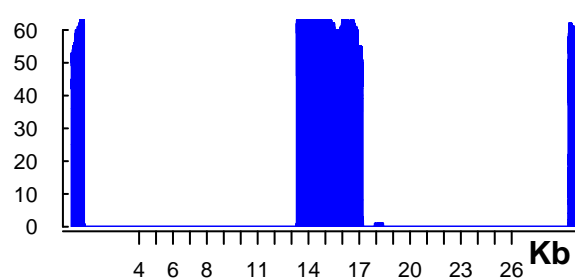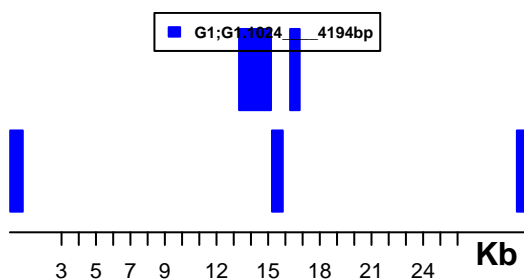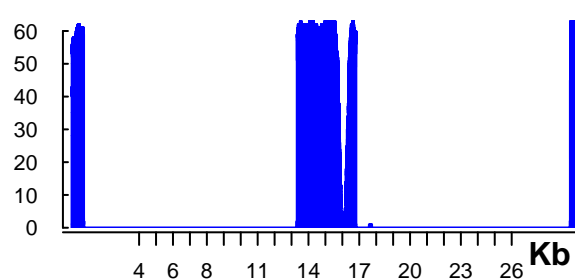

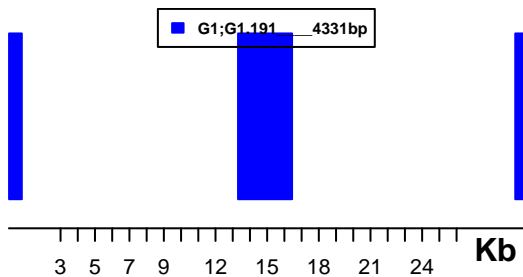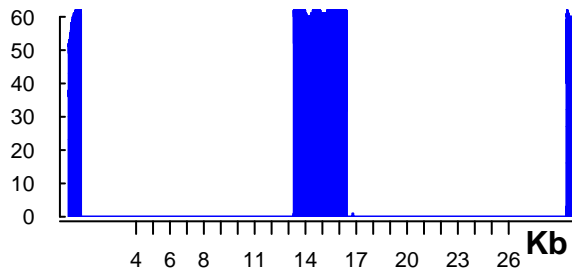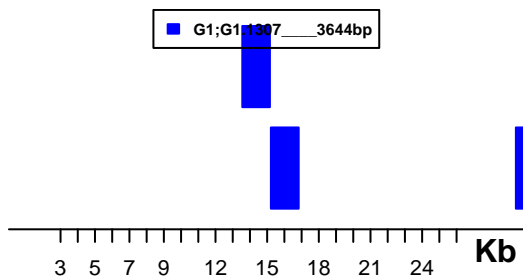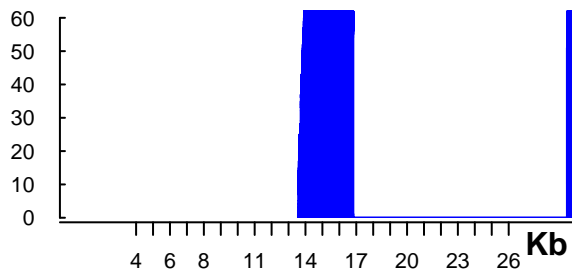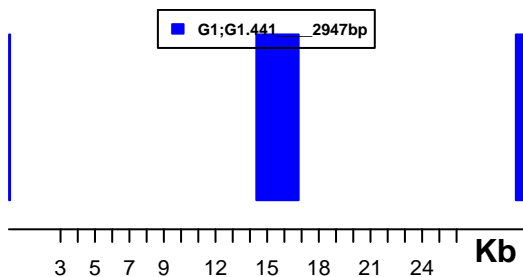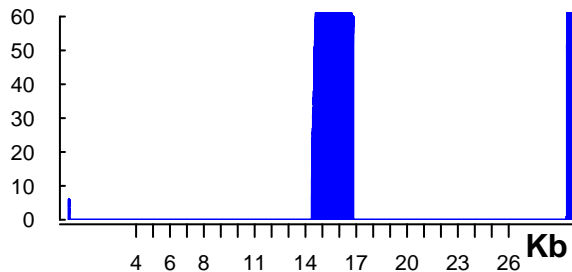

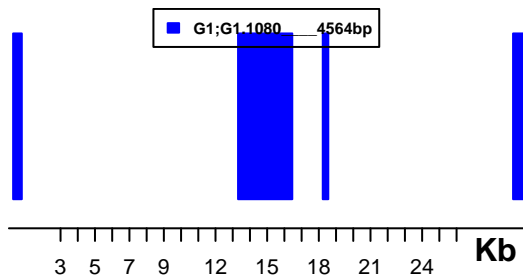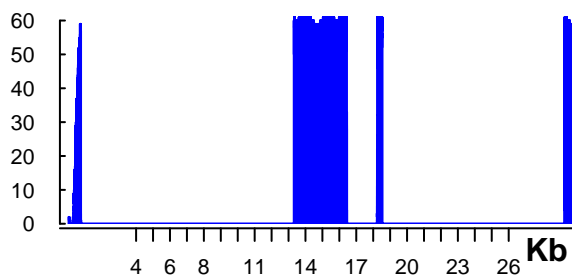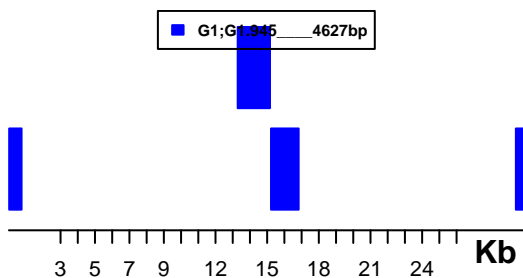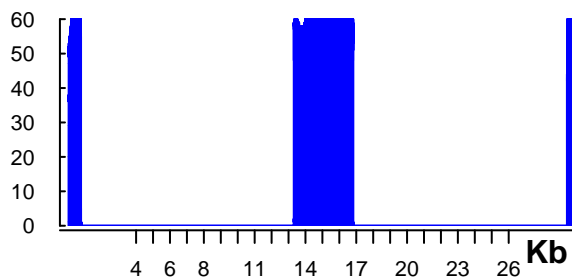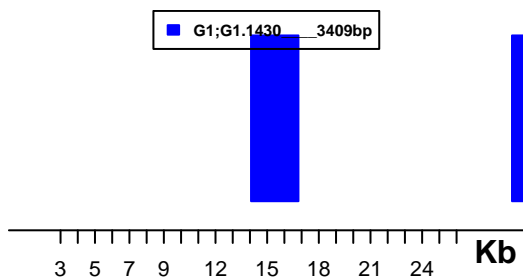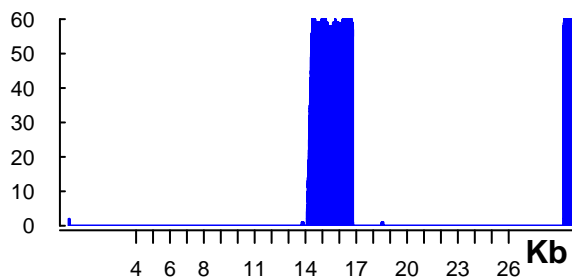

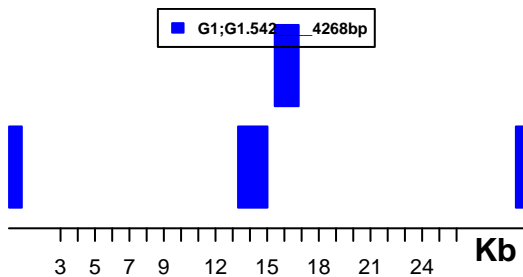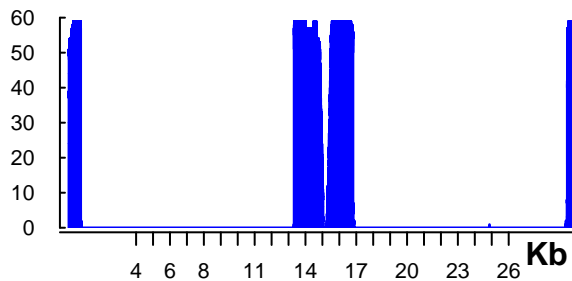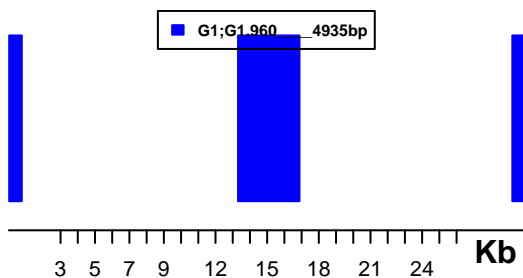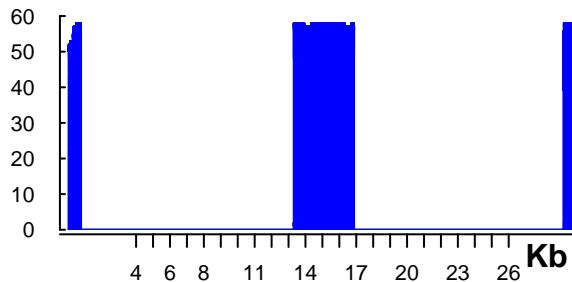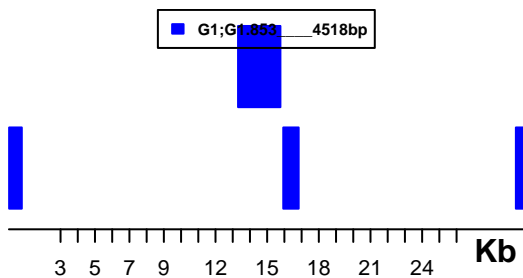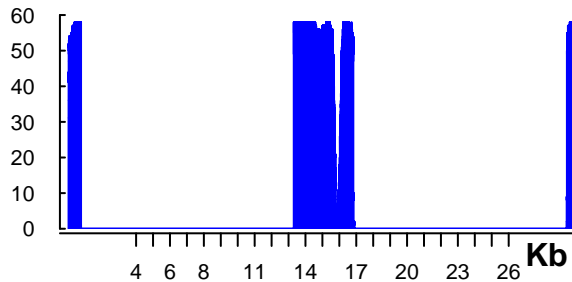

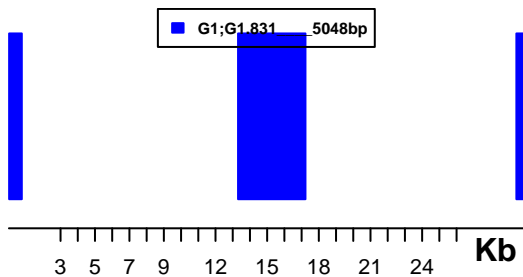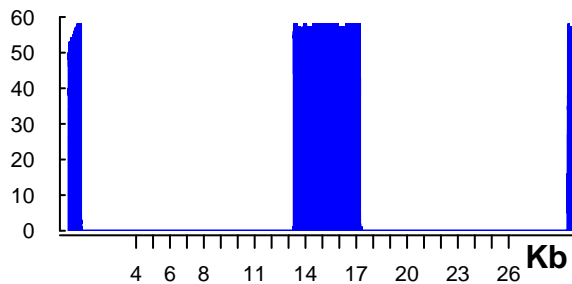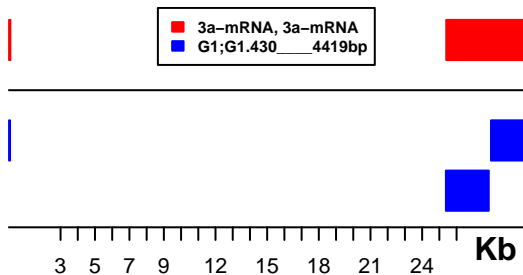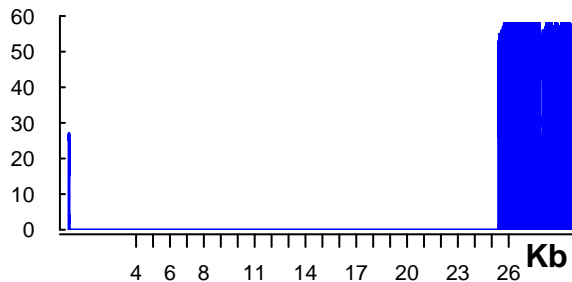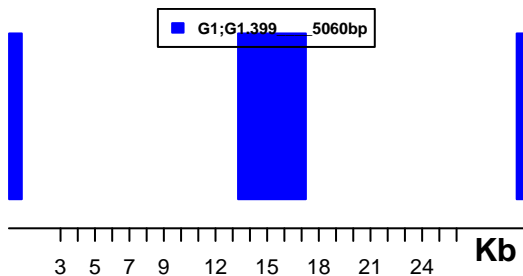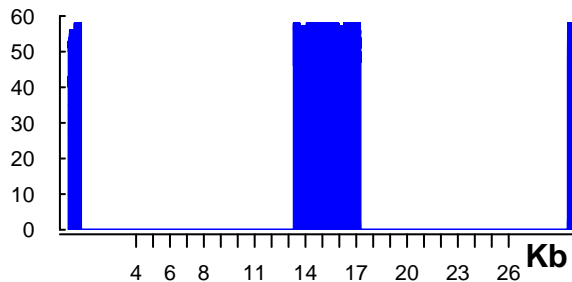

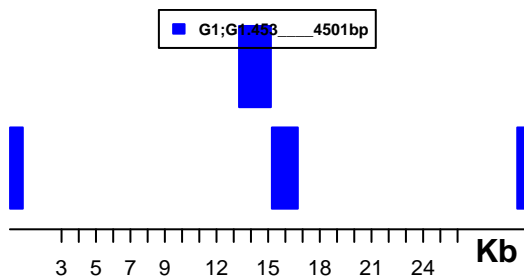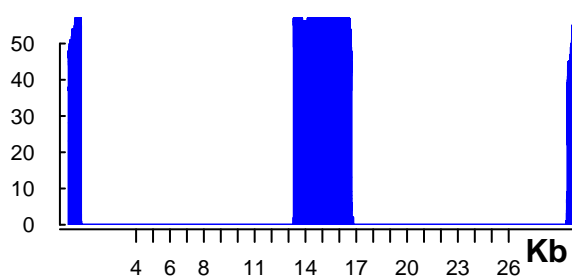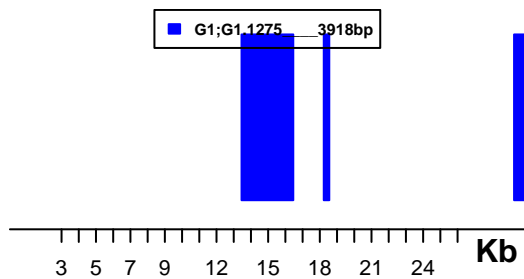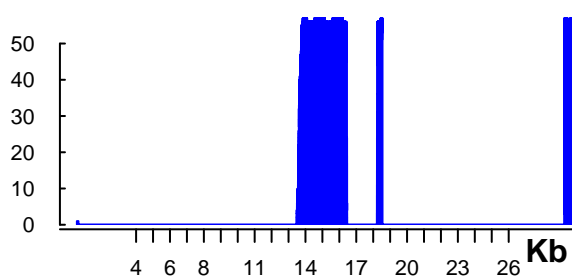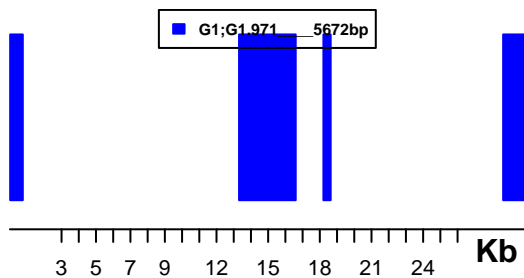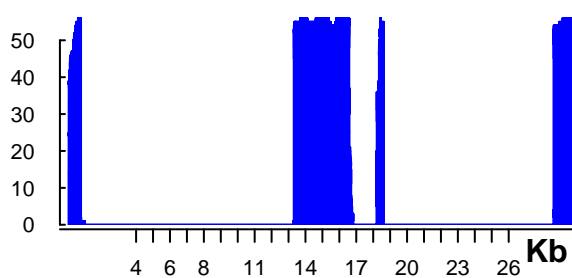

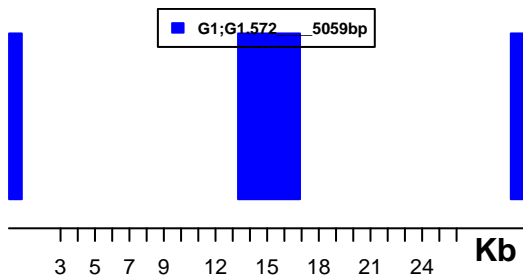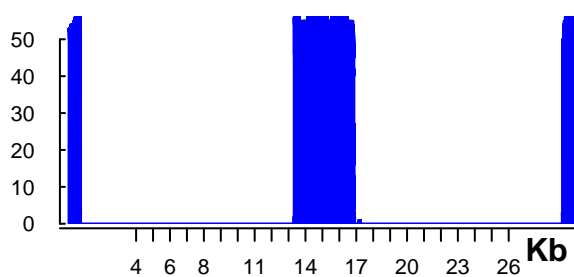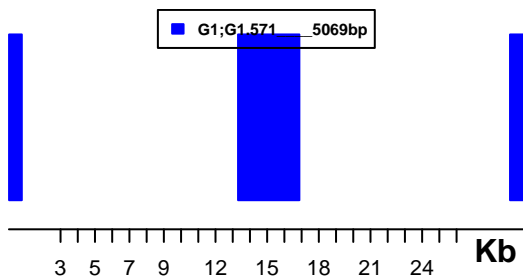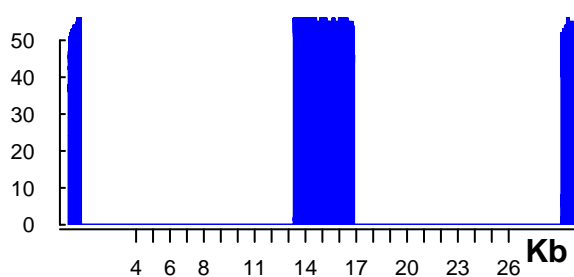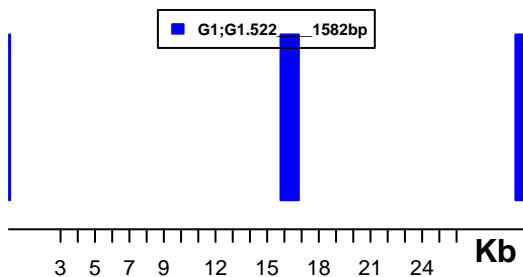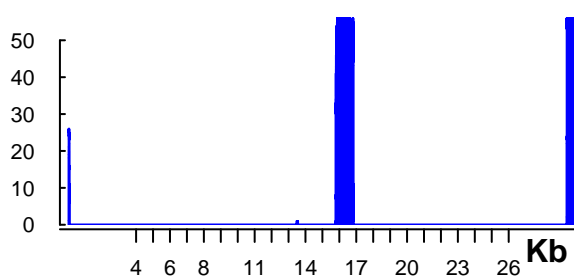

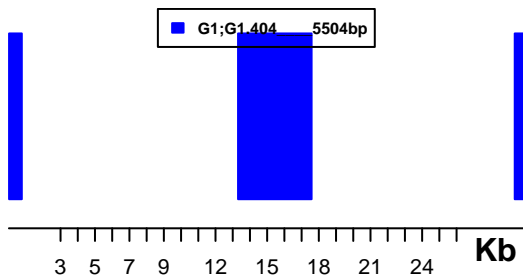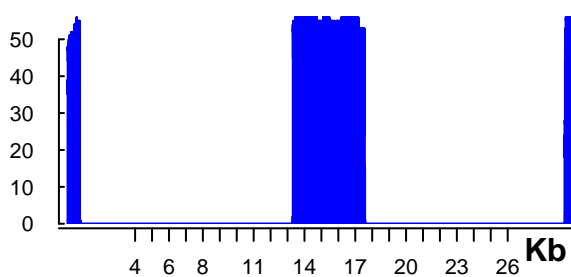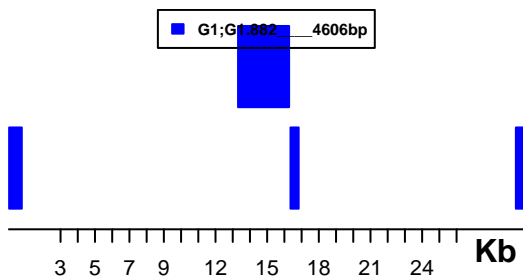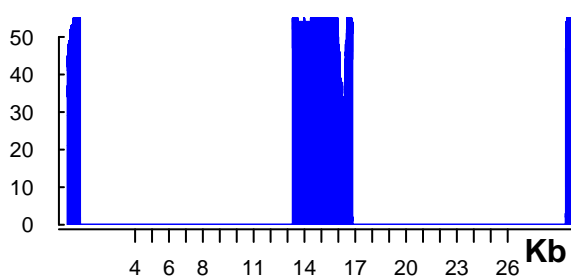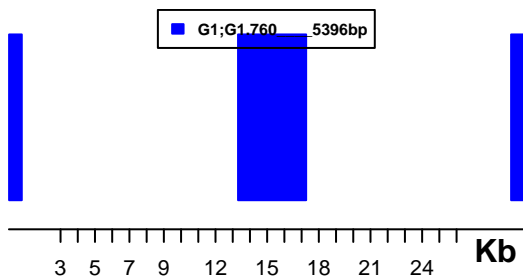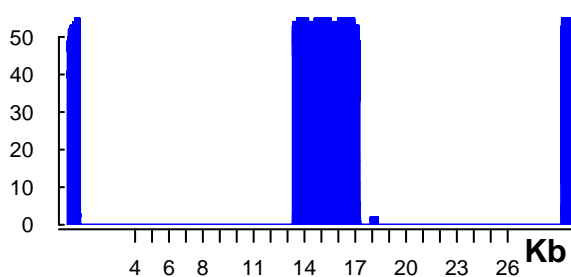

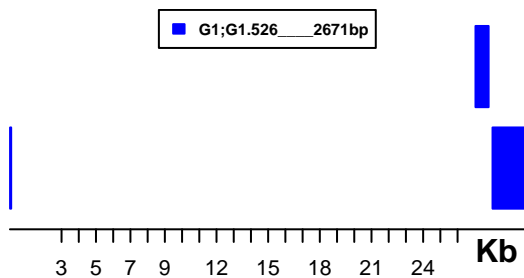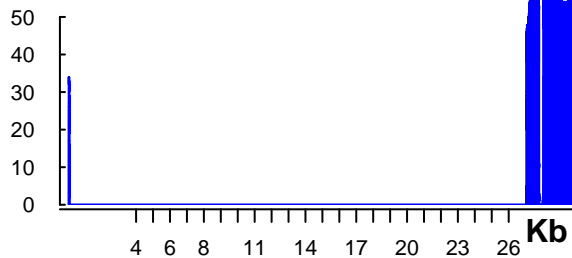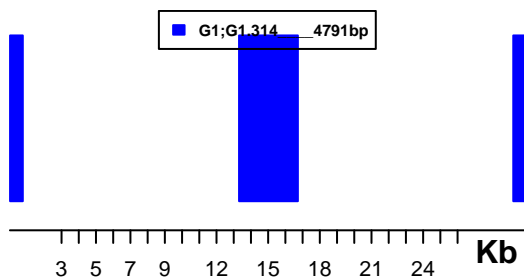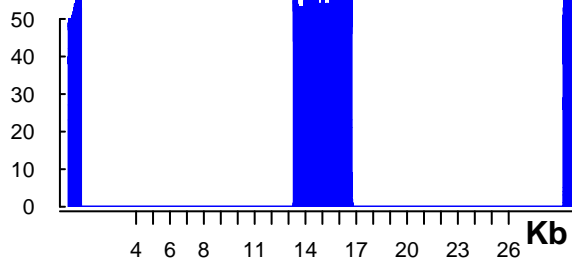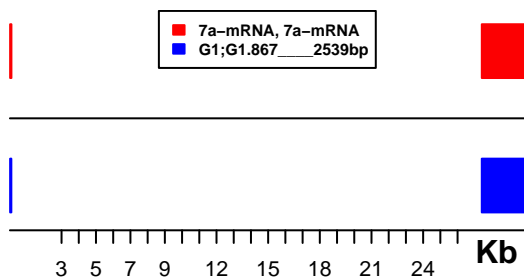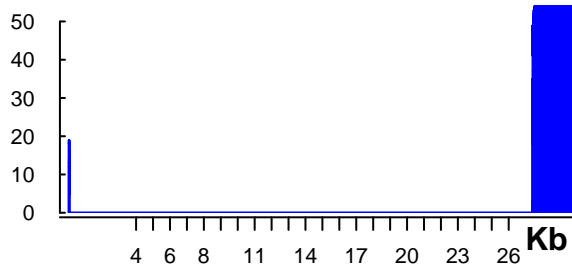

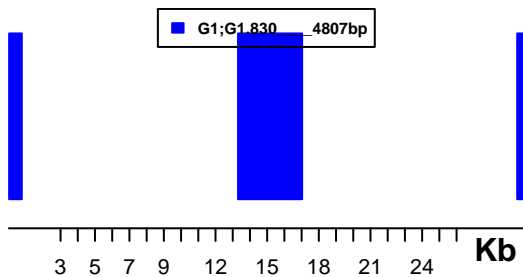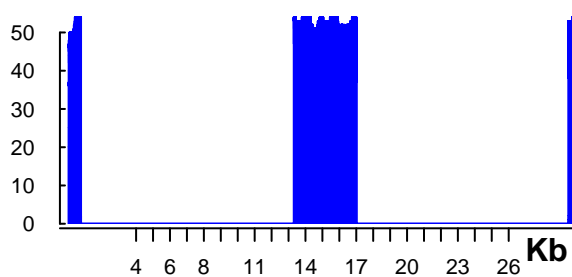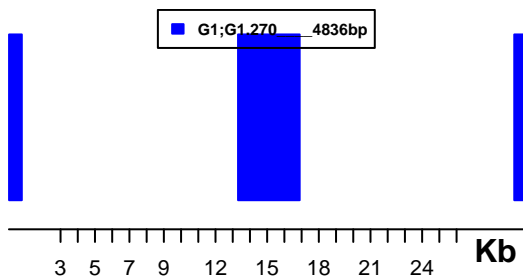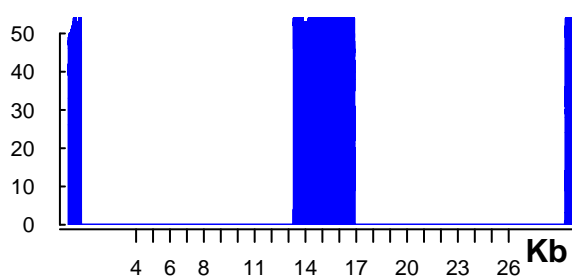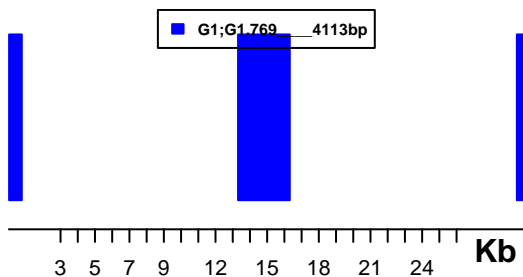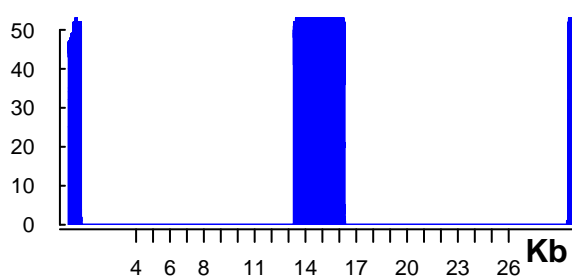

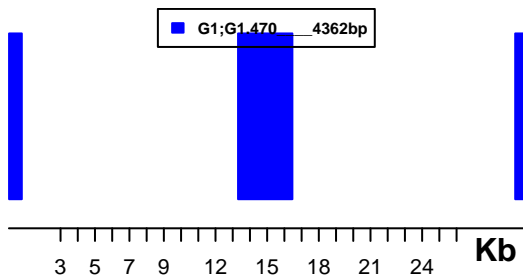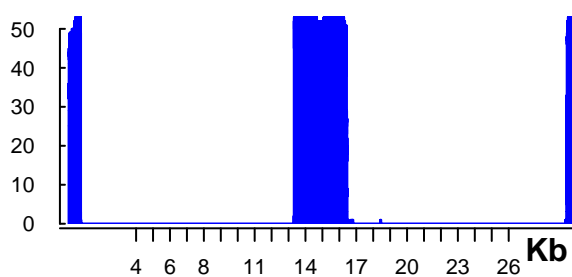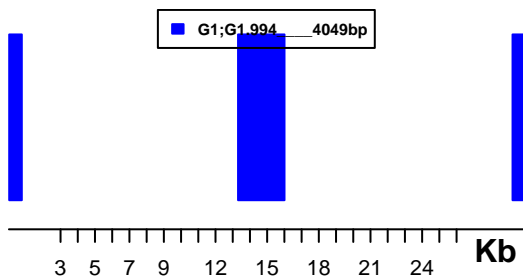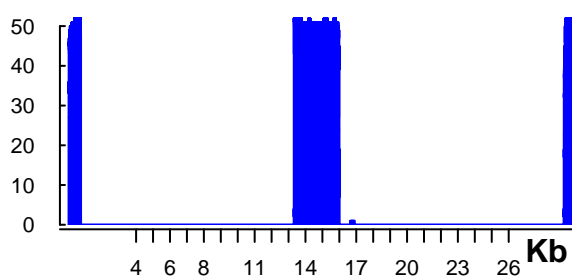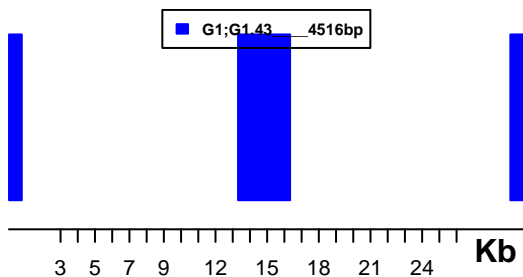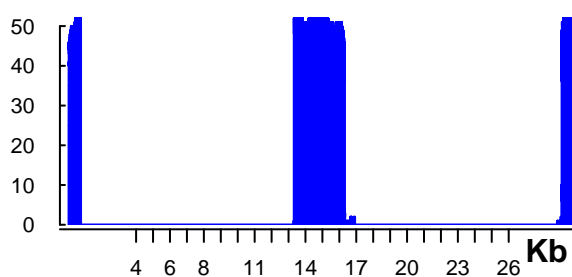

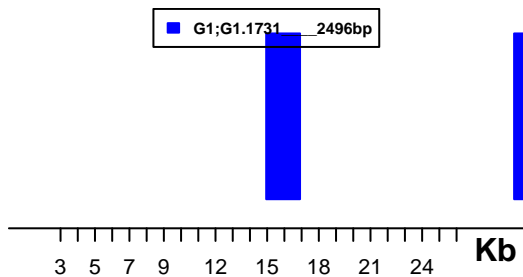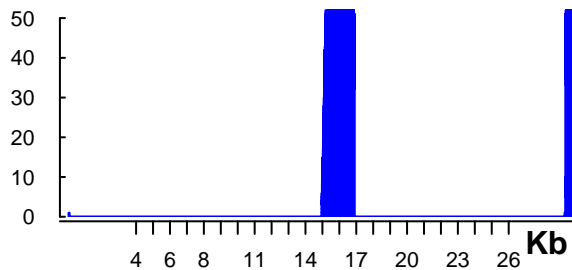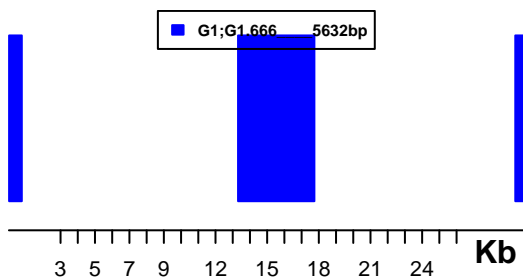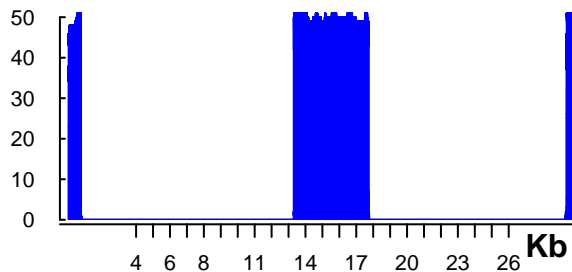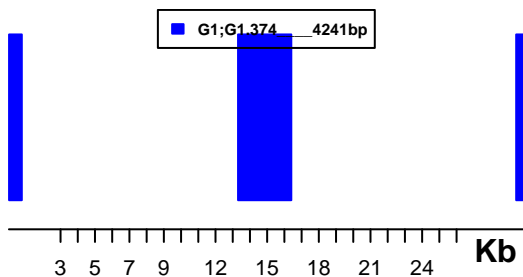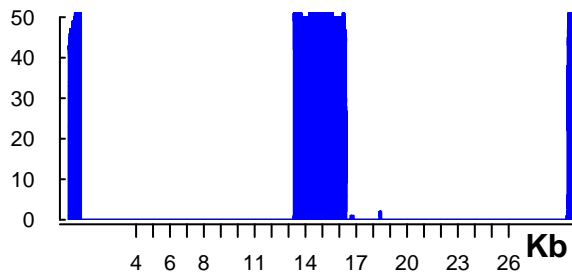

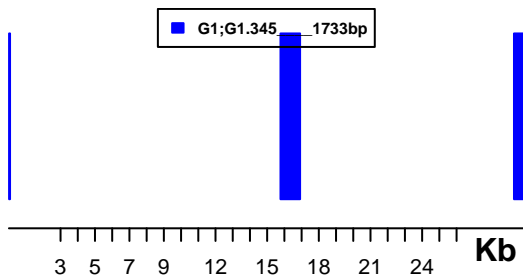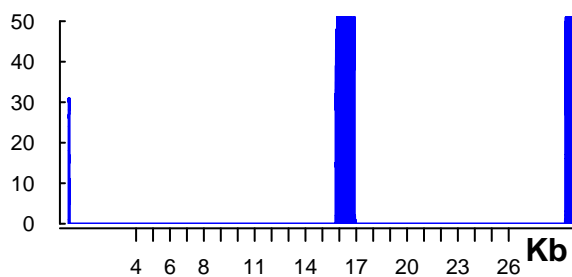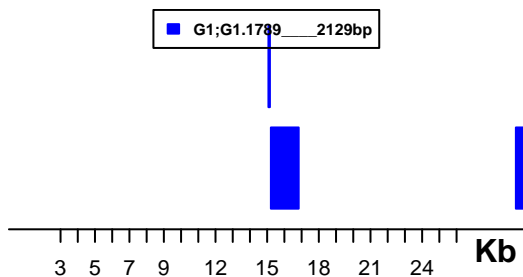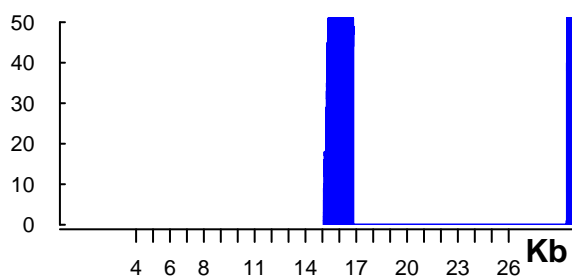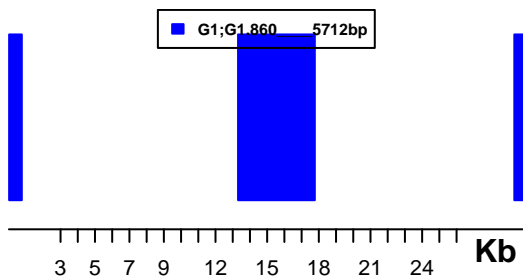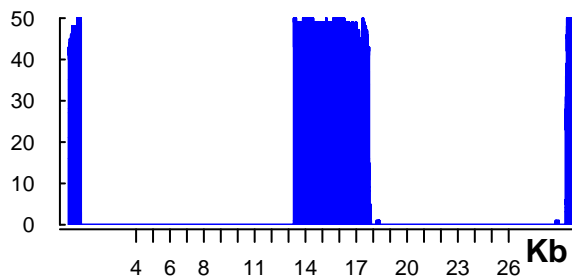

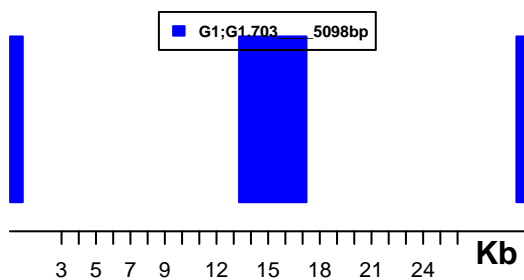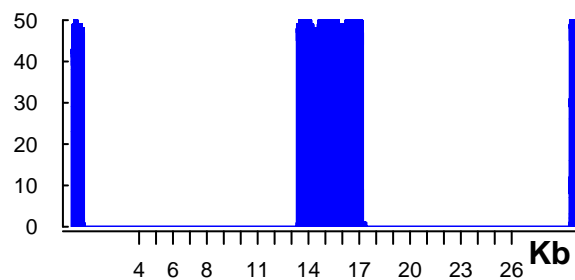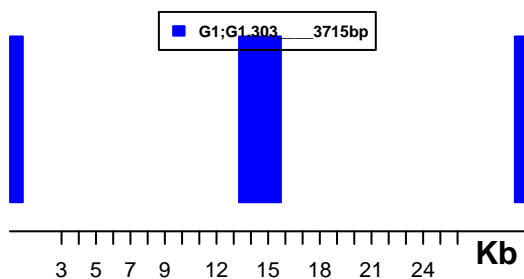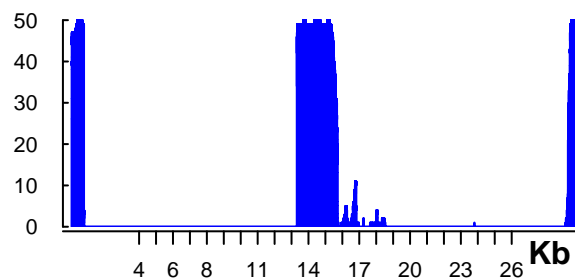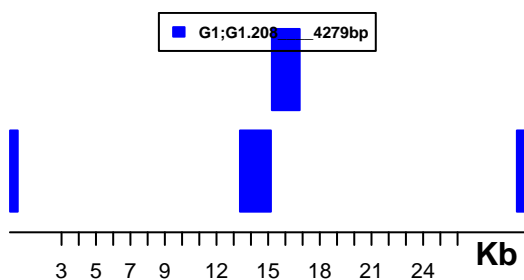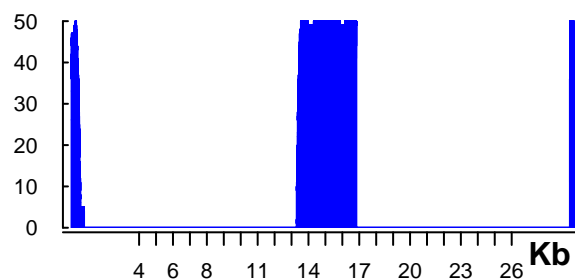

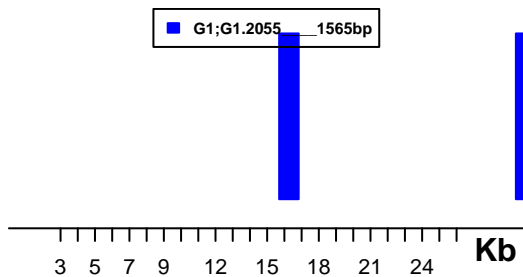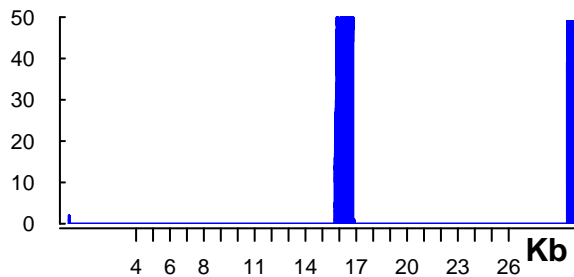

Supplement: Supplementary file 7 — Supplementary Data 5 [file 42003_2022_4058_MOESM7_ESM.zip › experiment2/Files_used_for_the_analysis_of_the_manuscript_experiment2/RNA_MODELS_WITH_COVERAGE_experiment_2_passage_29.pdf]

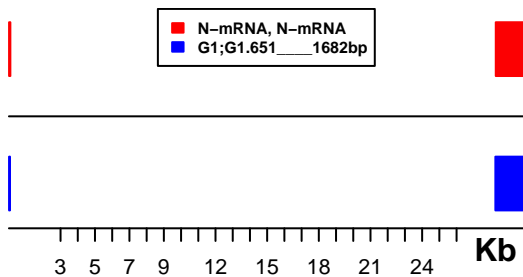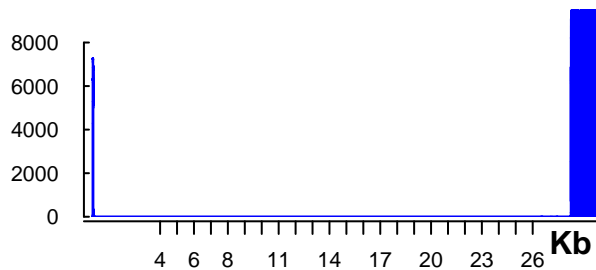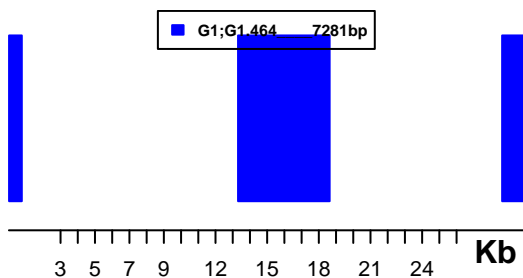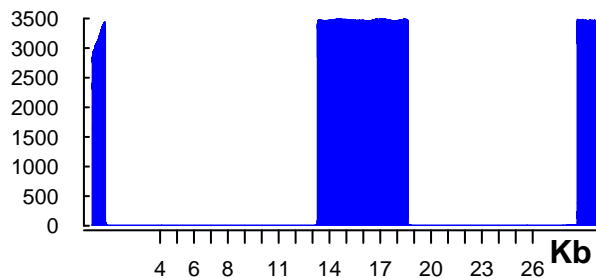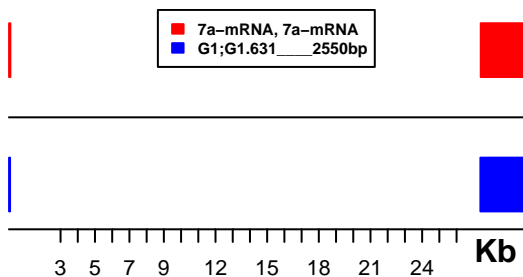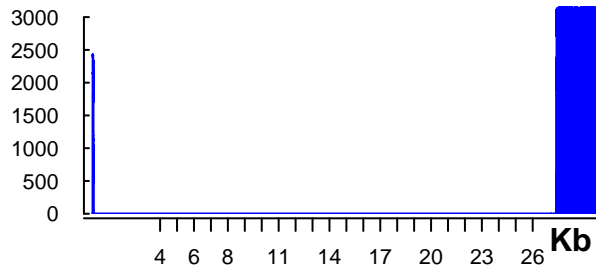

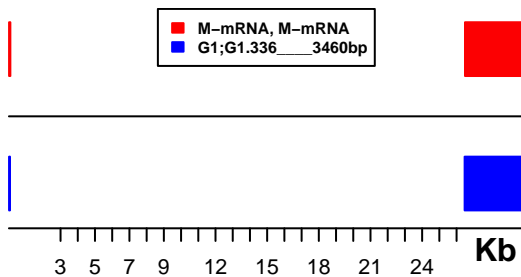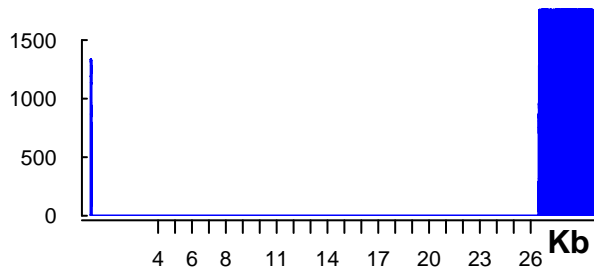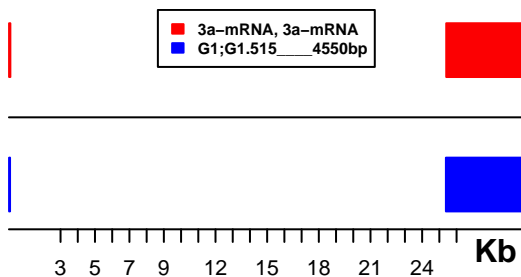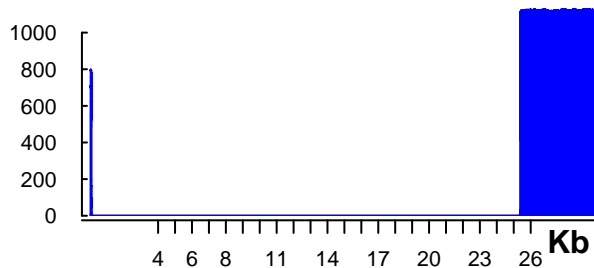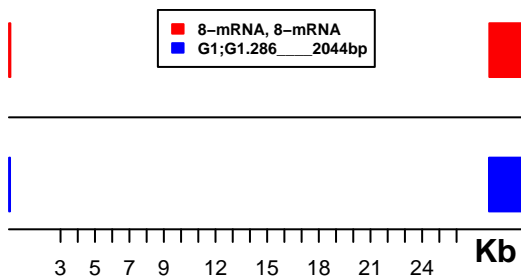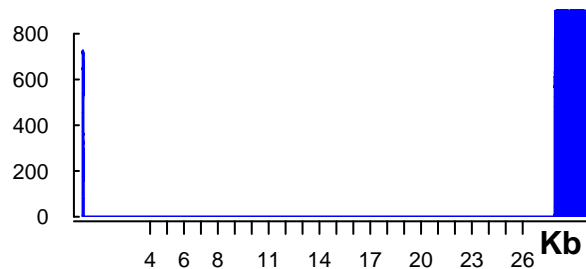

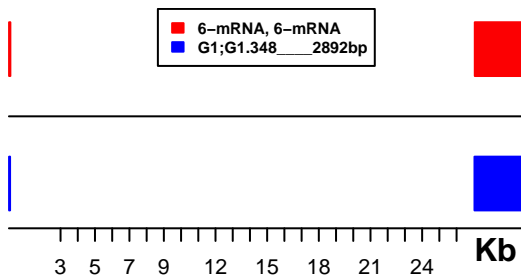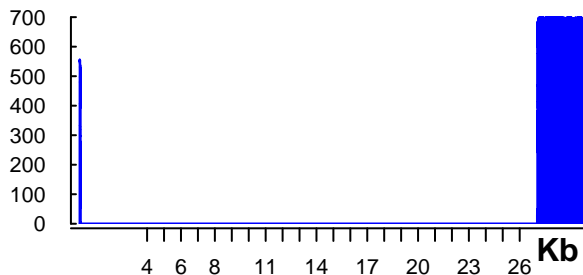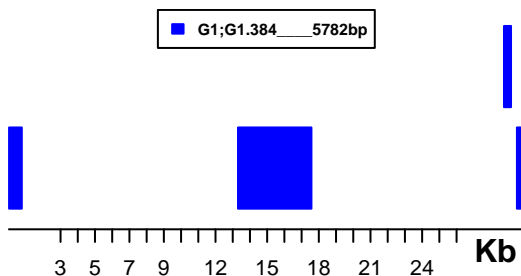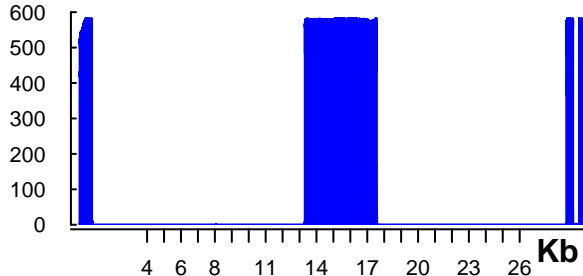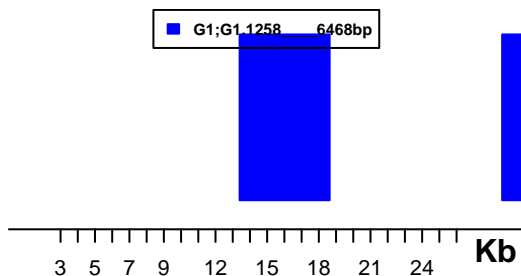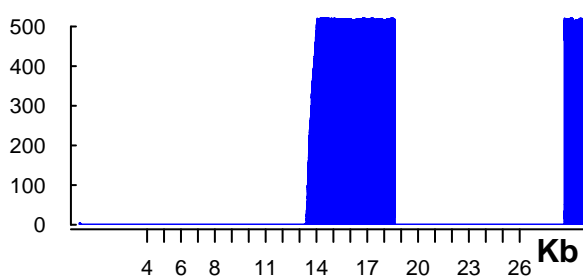

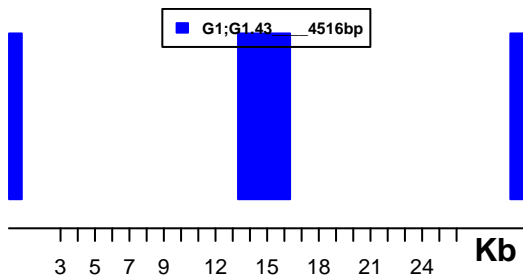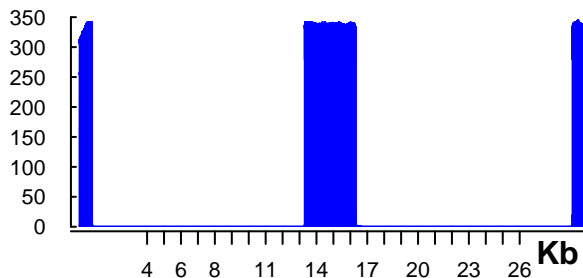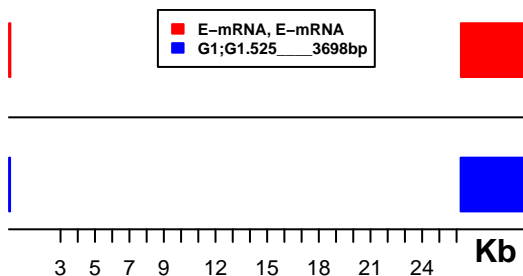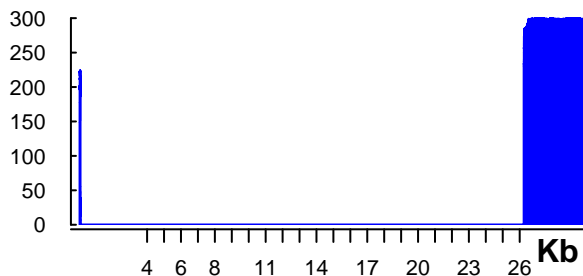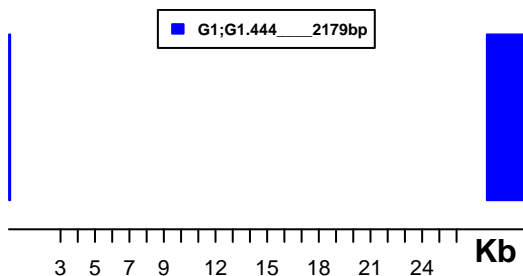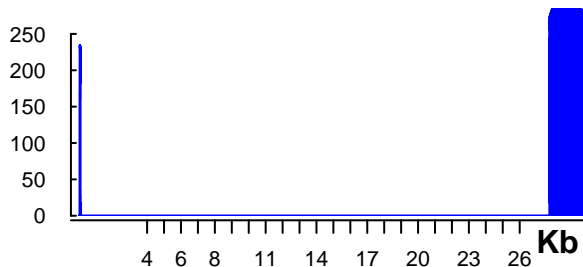

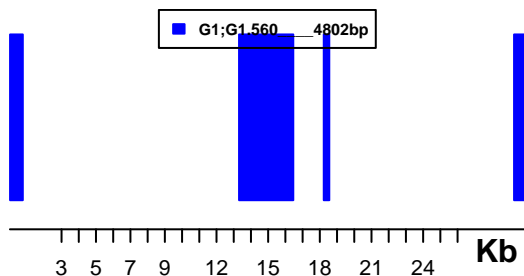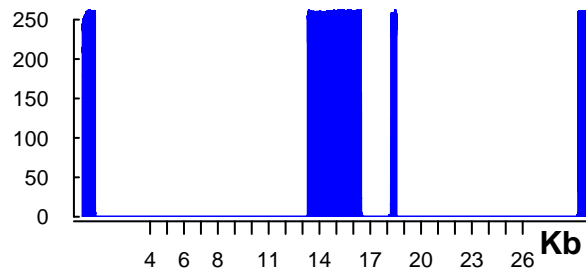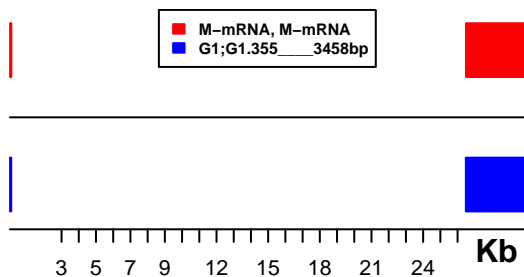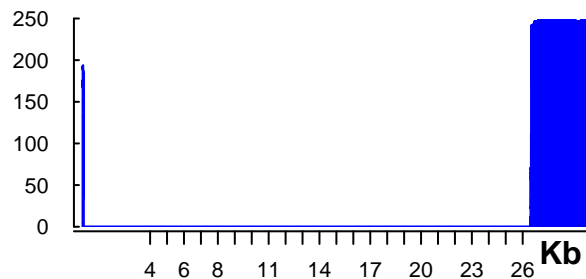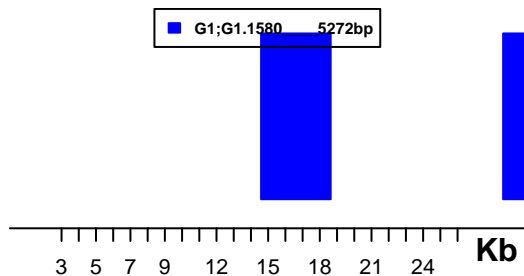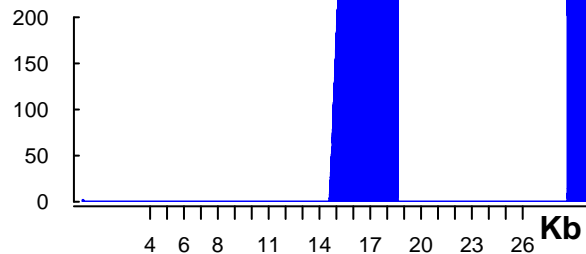

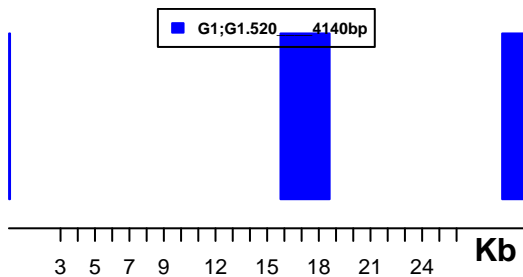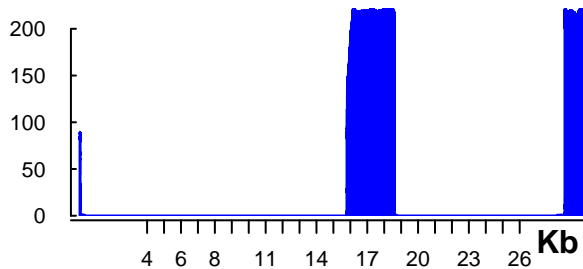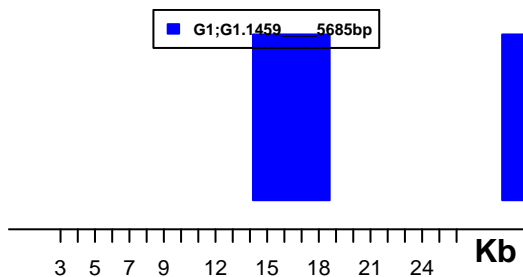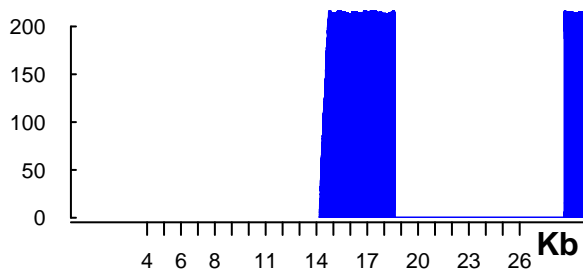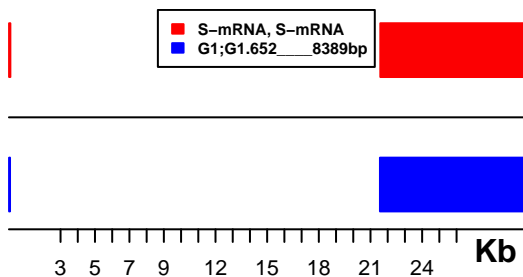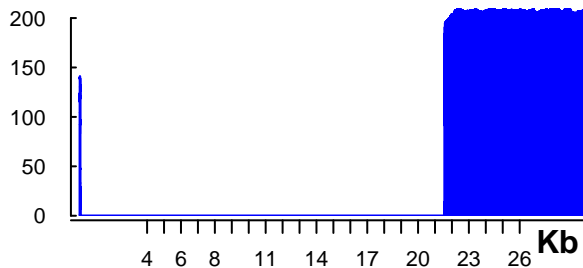

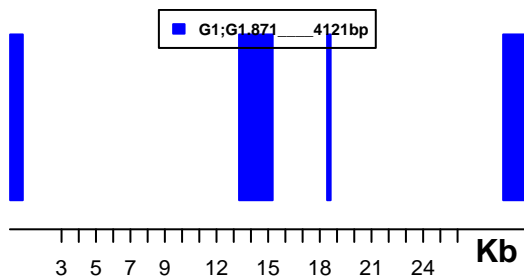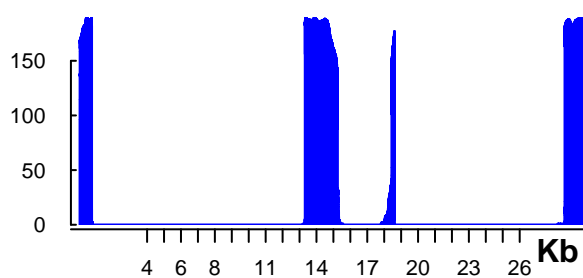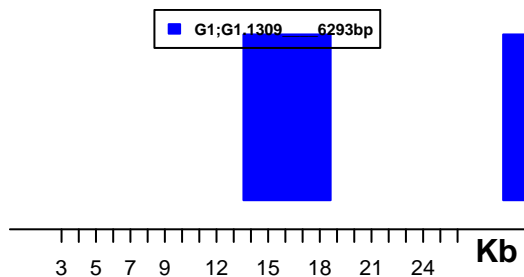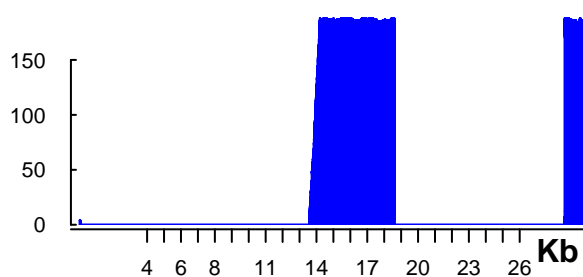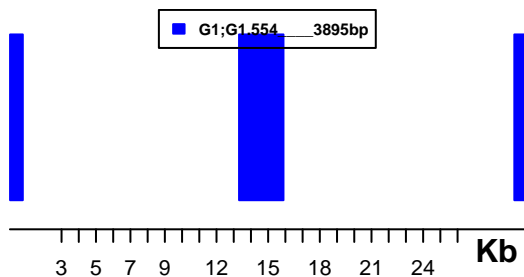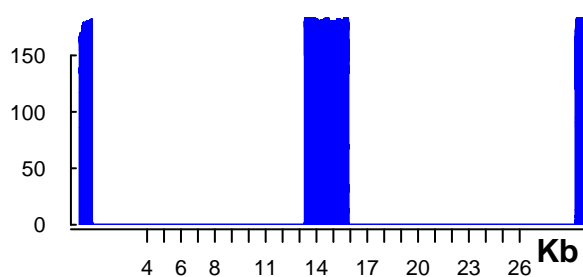

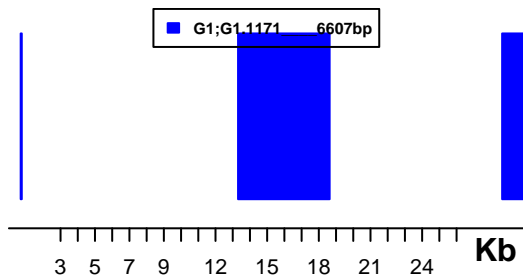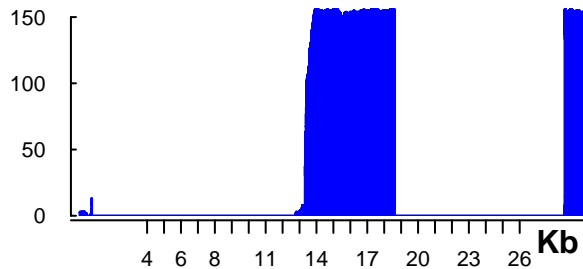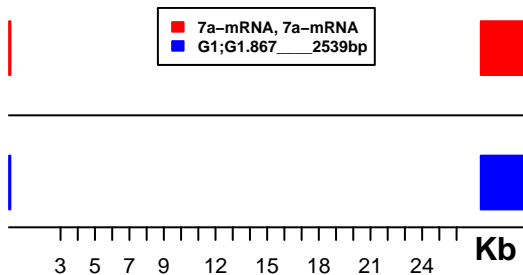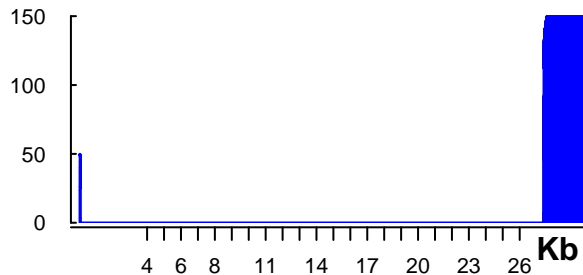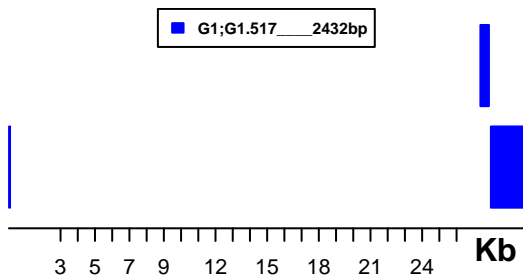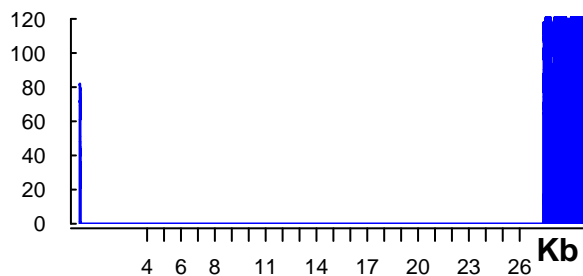

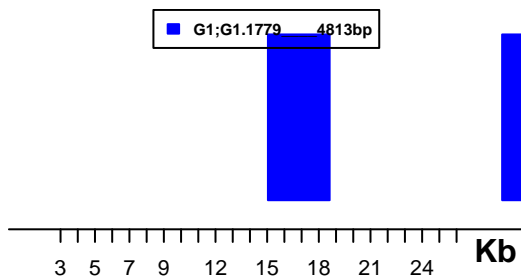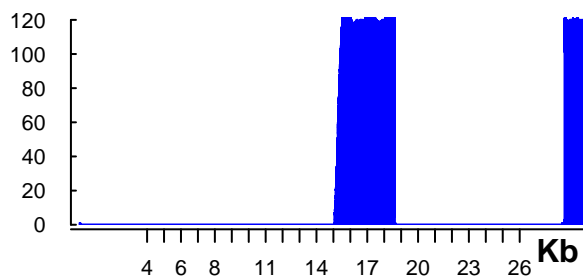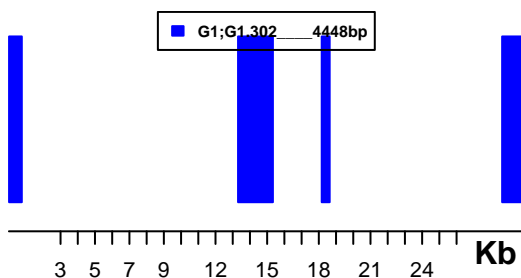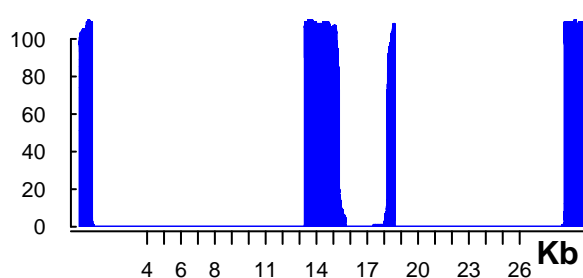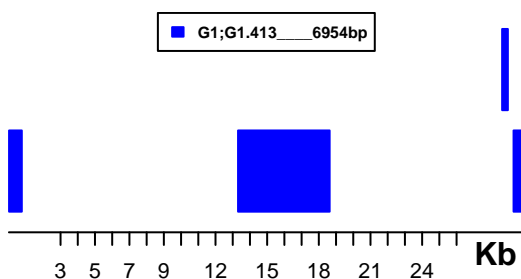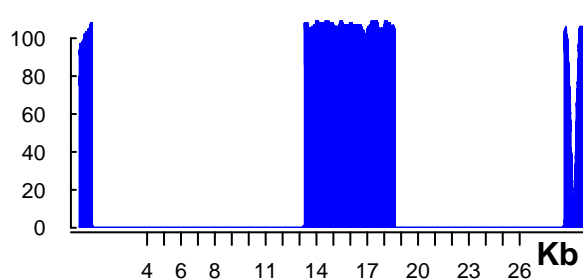

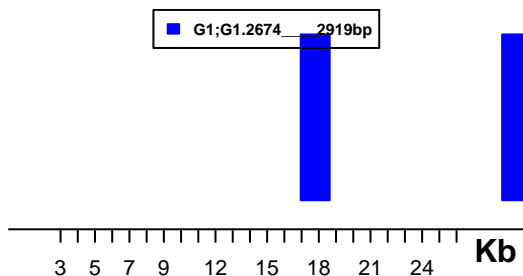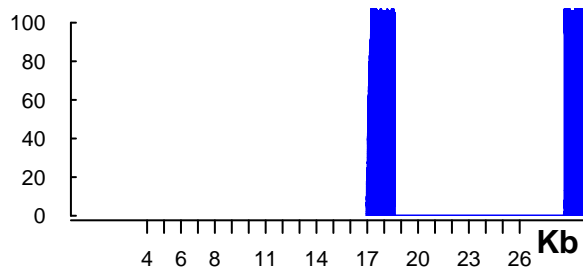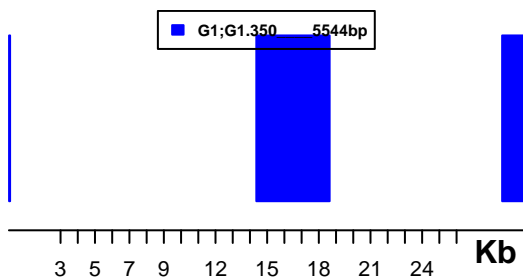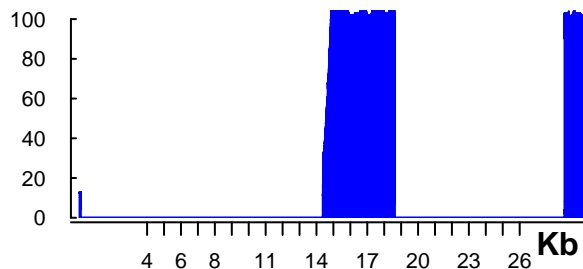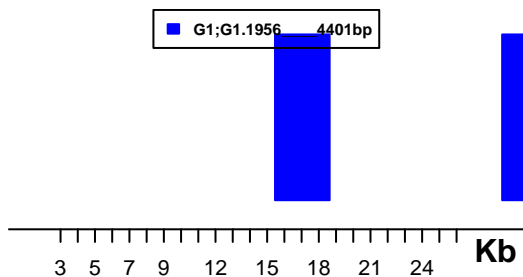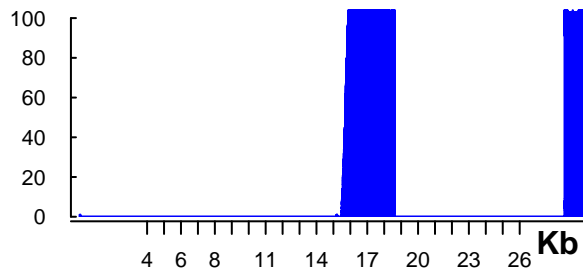

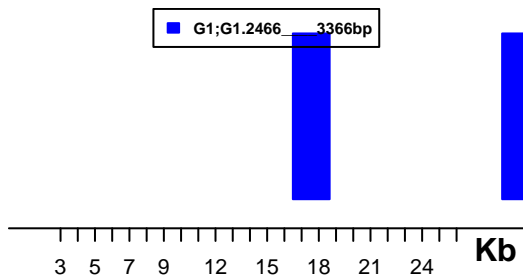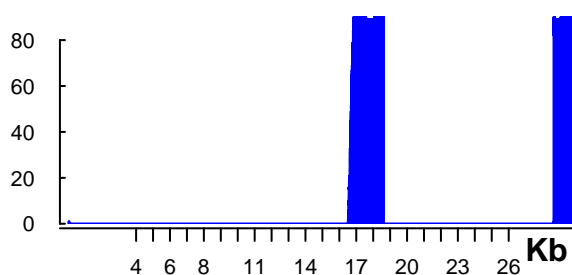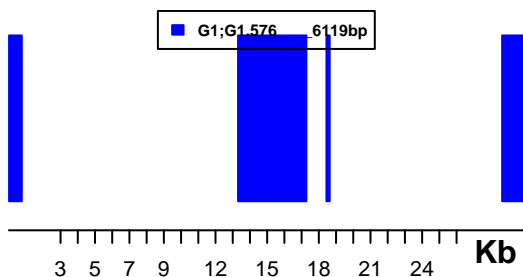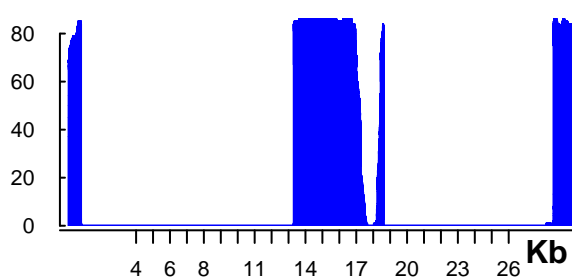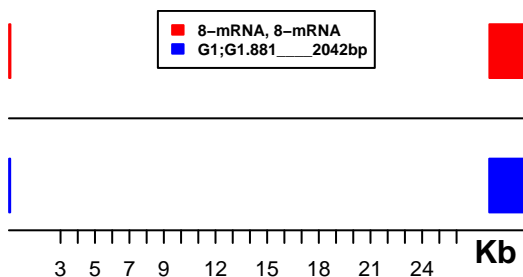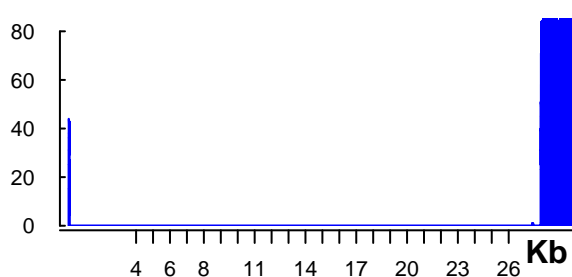

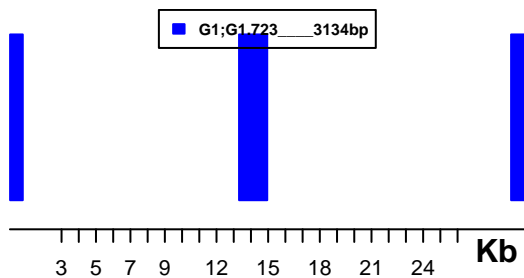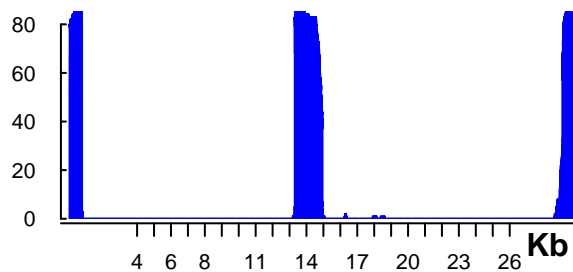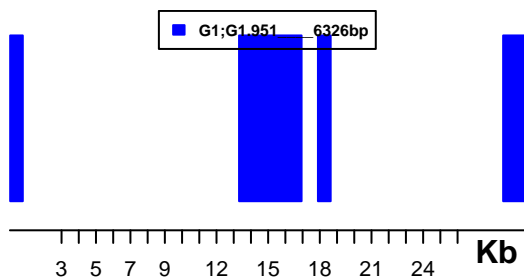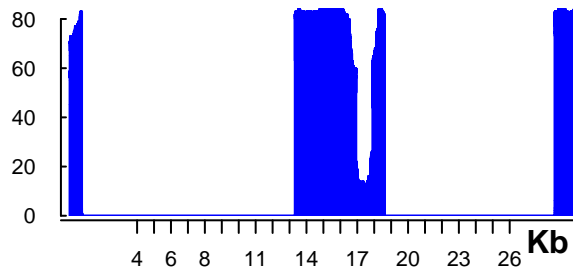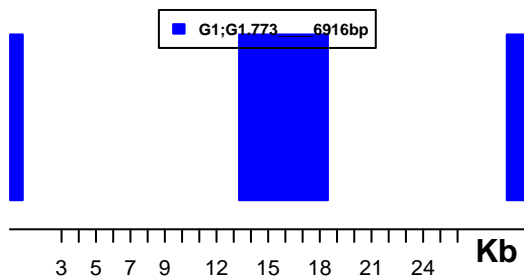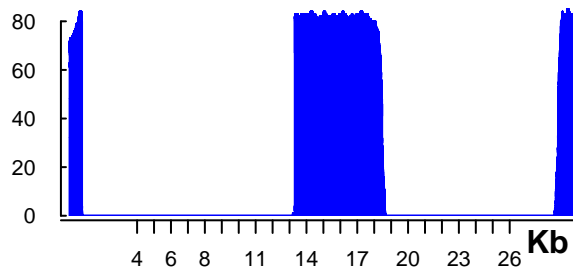

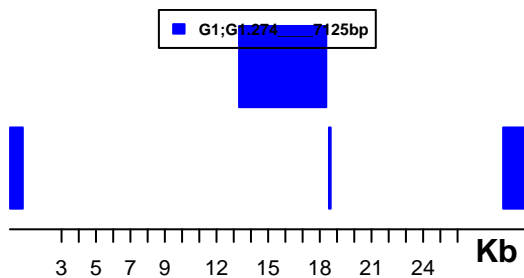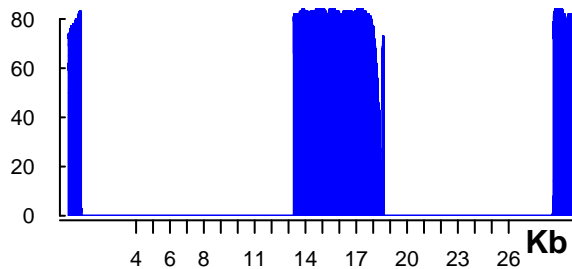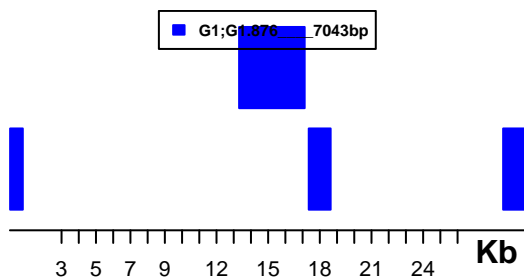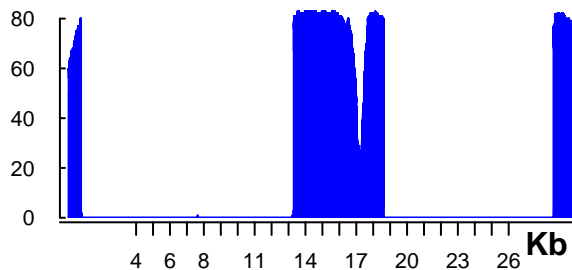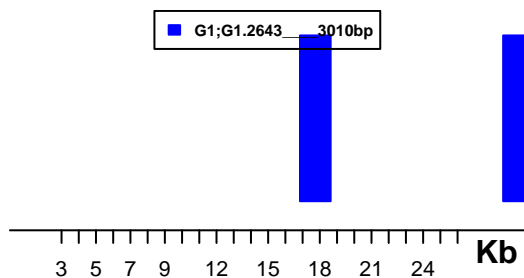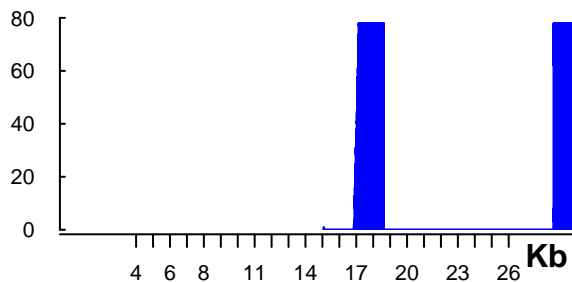

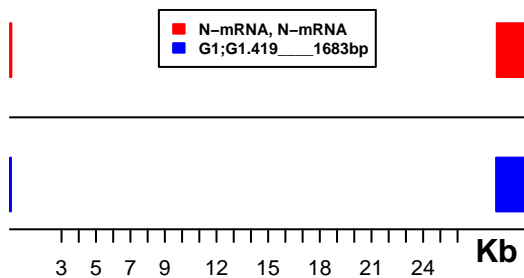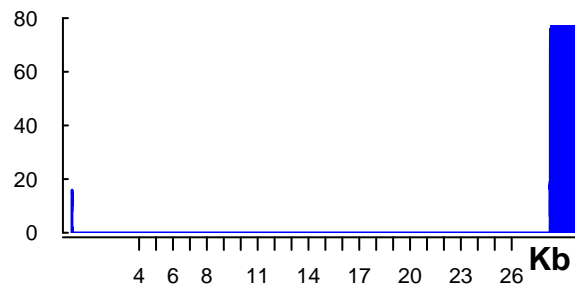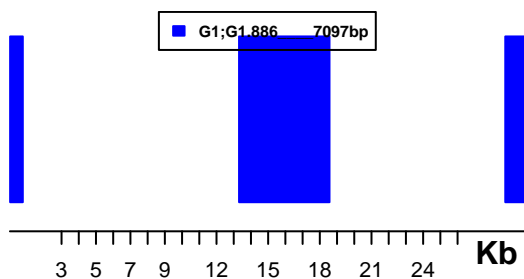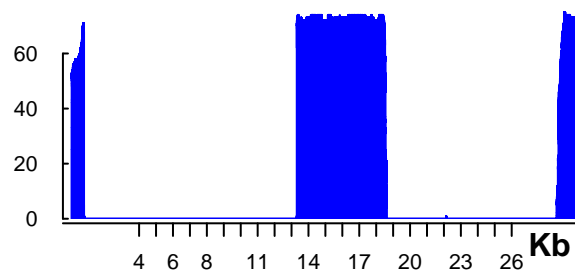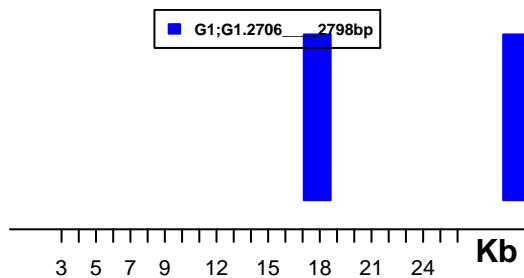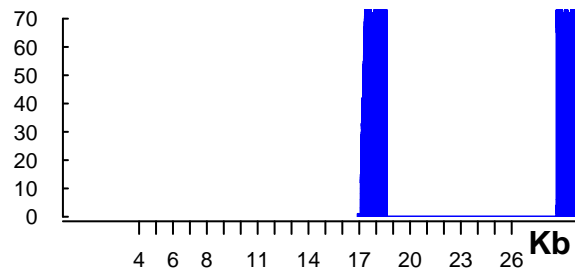

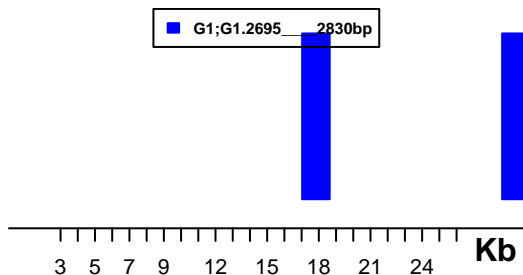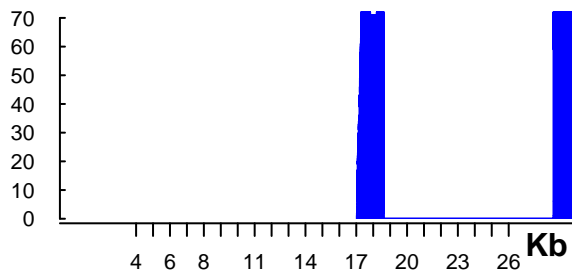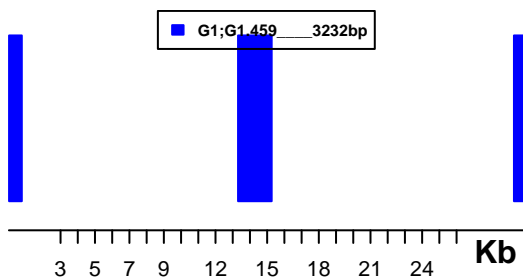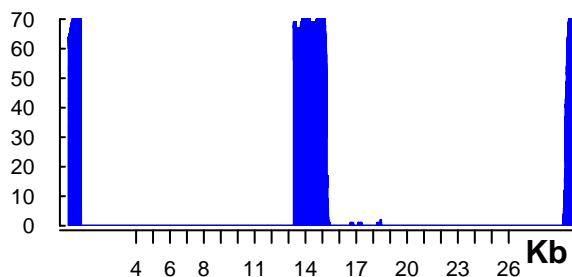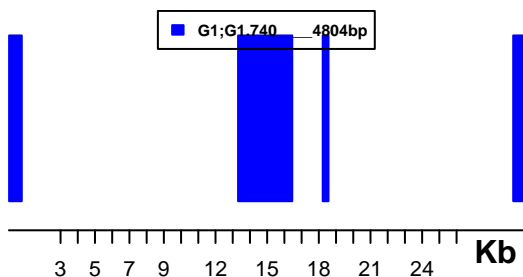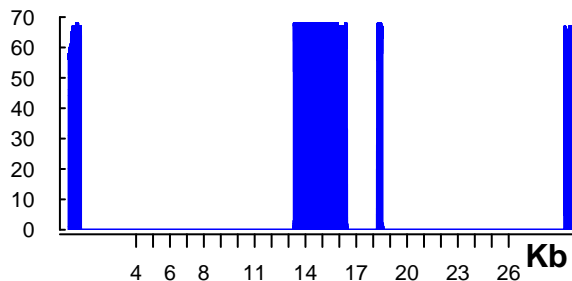

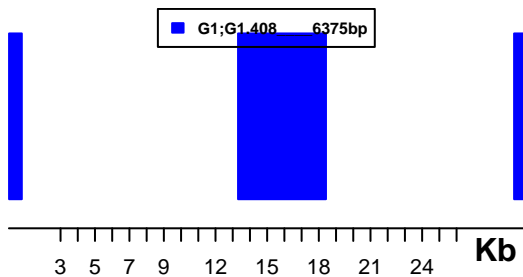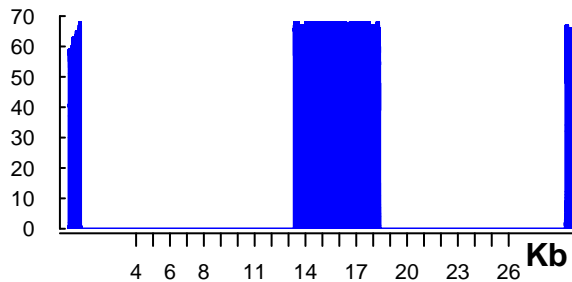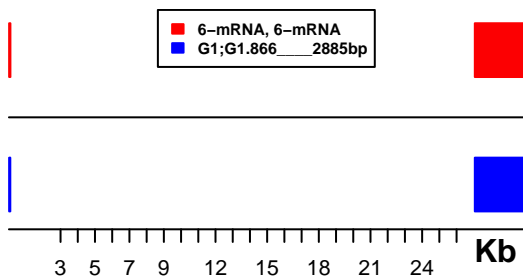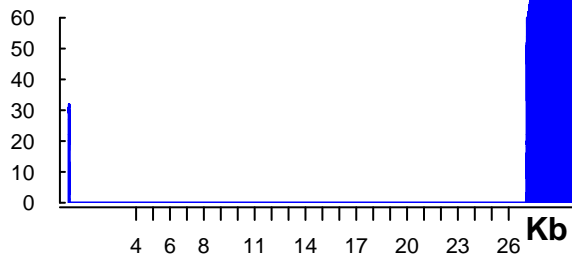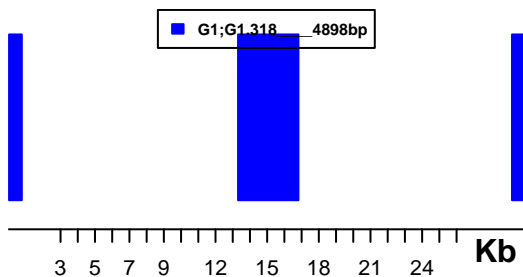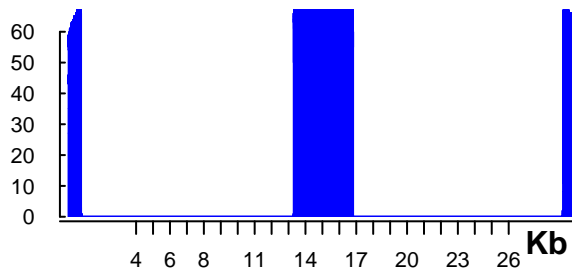

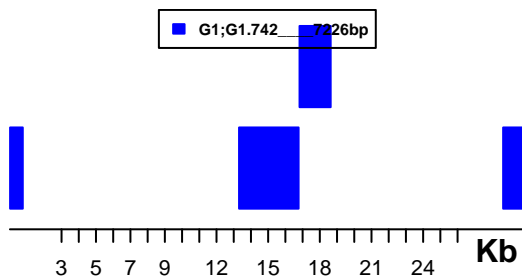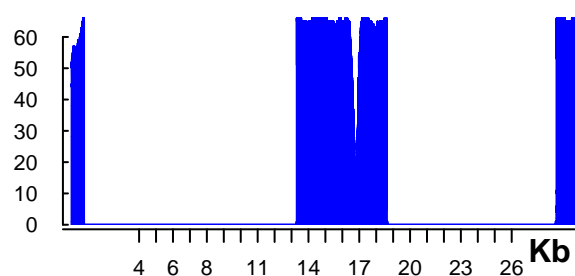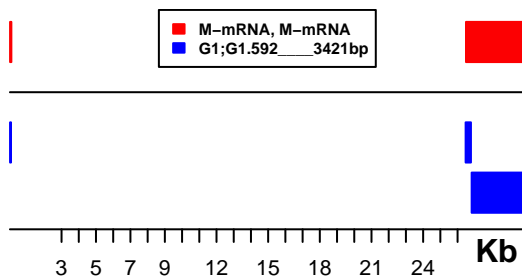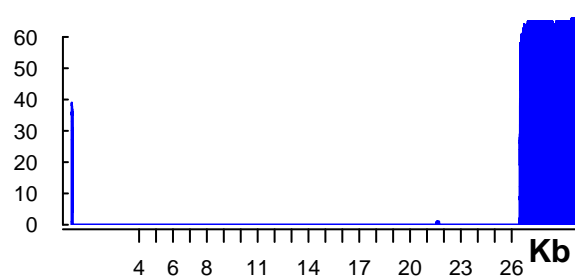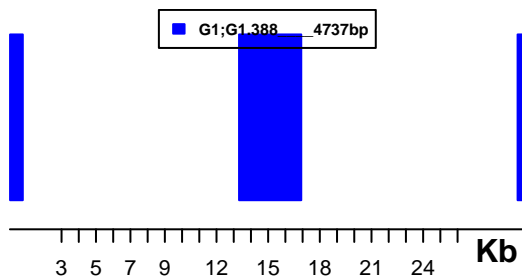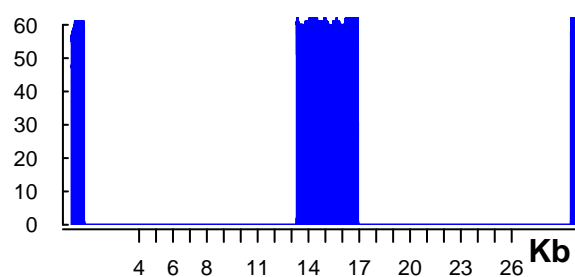

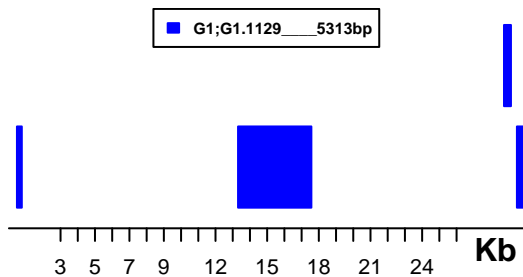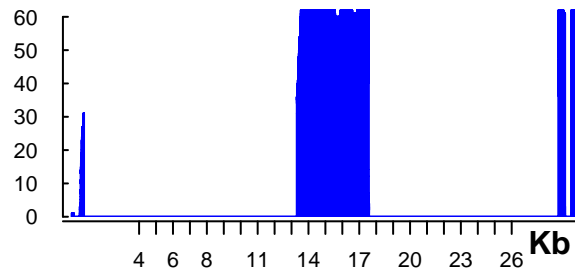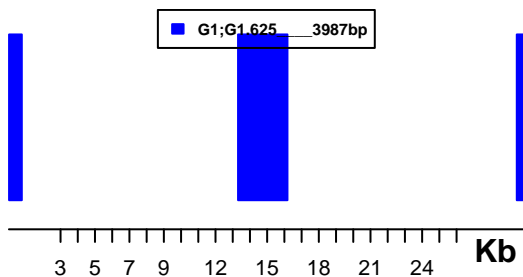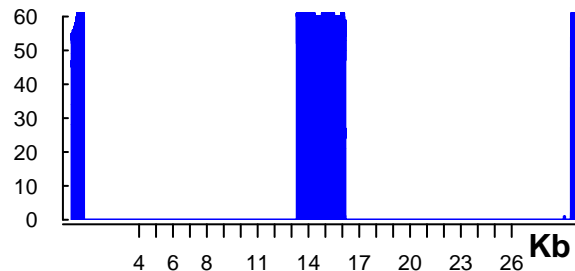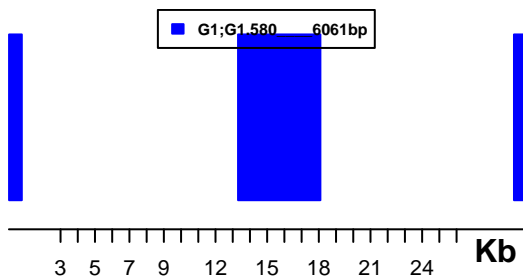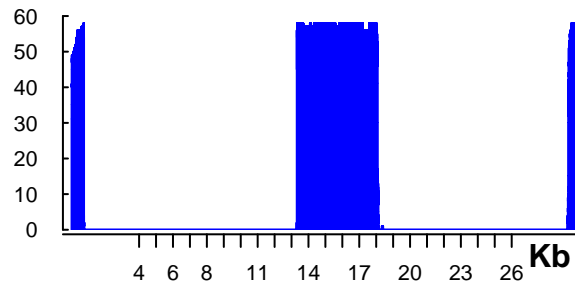

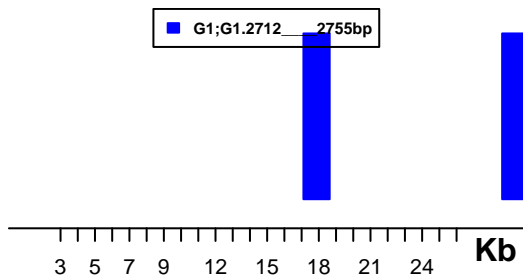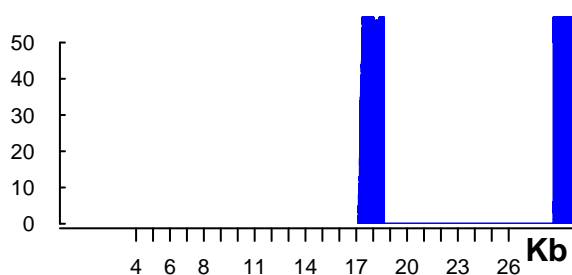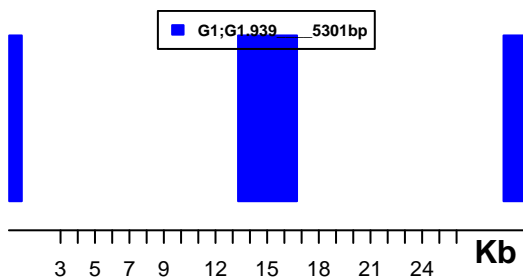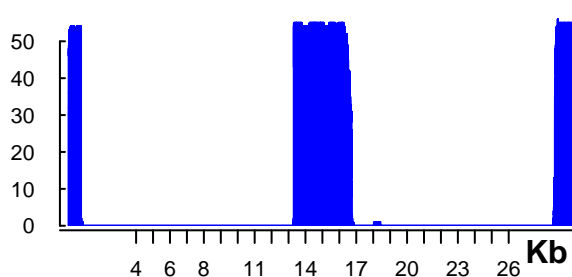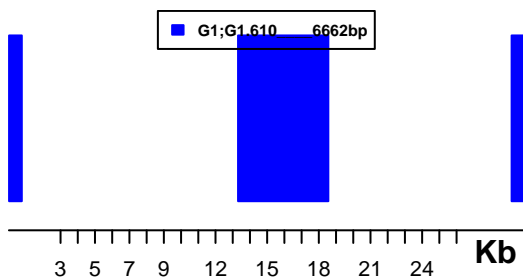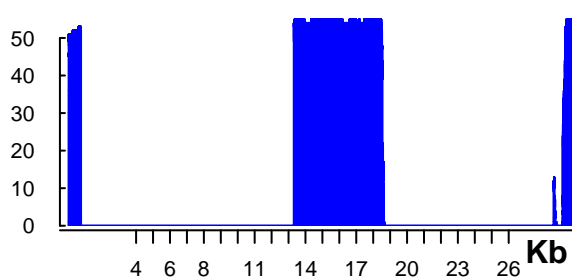

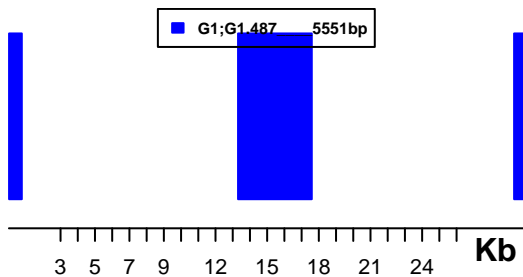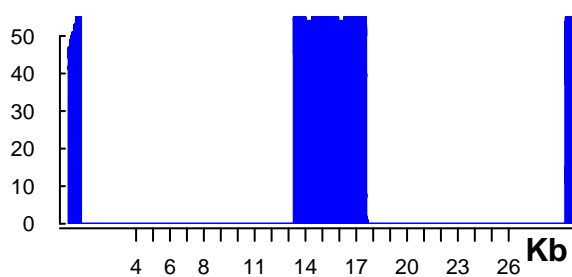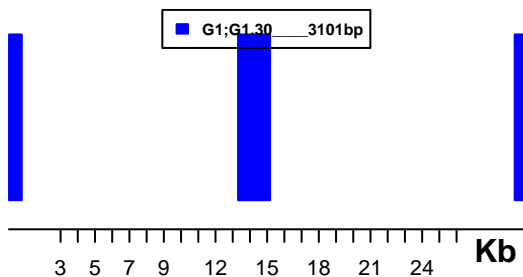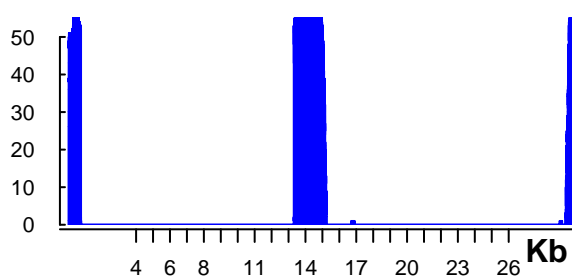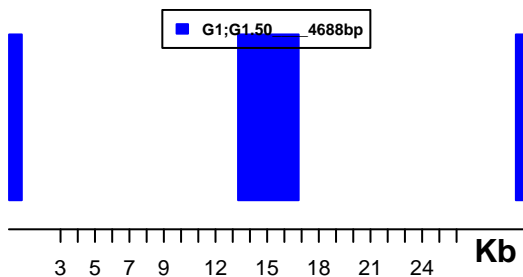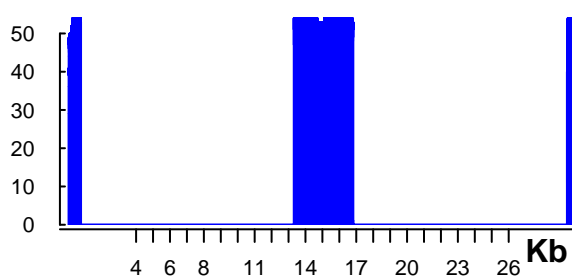

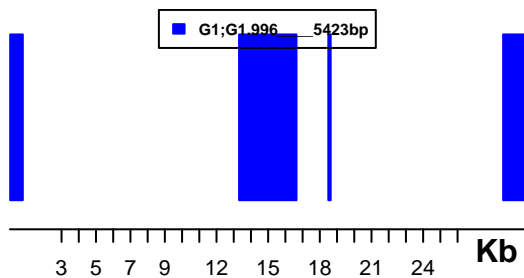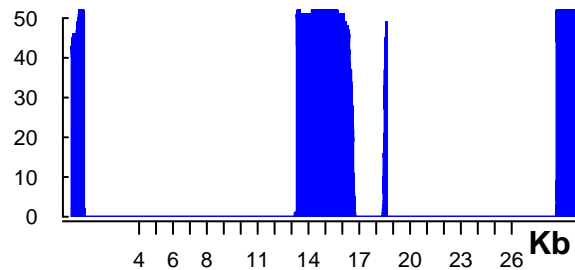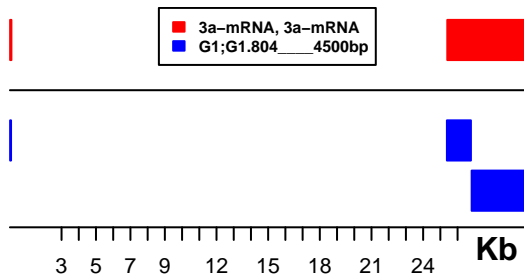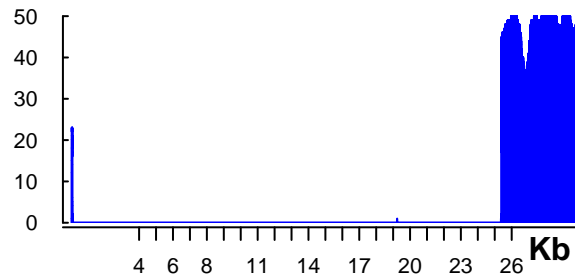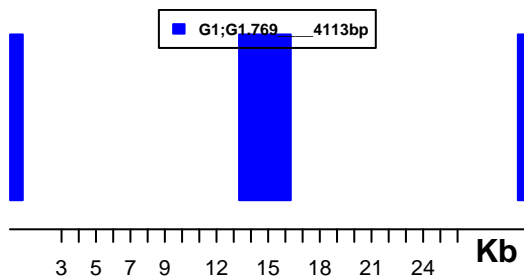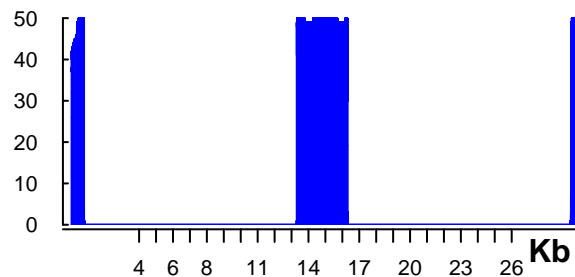

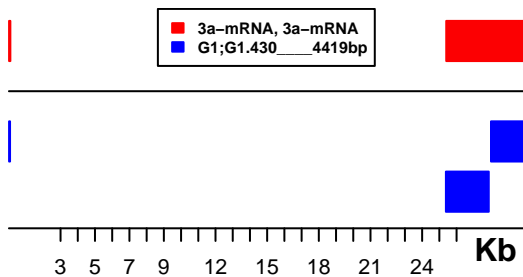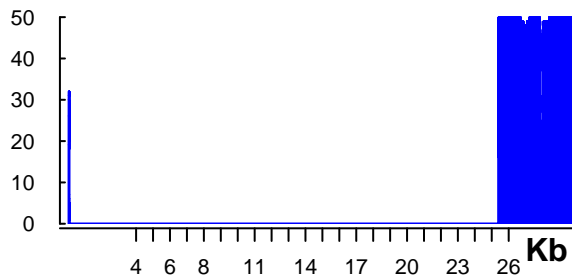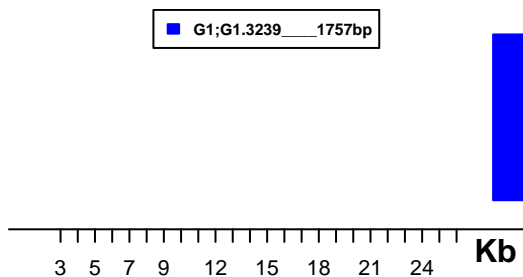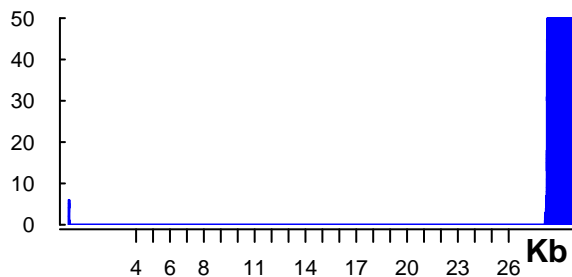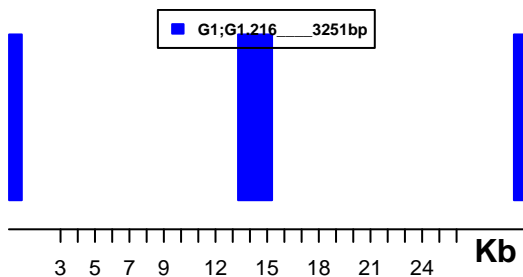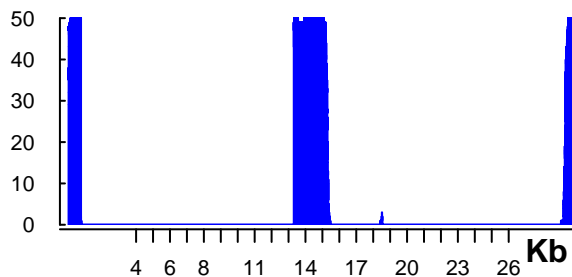

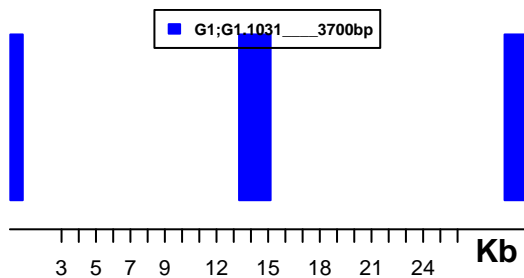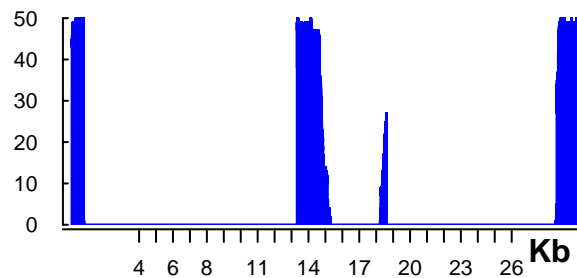

Supplement: Supplementary file 7 — Supplementary Data 5 [file 42003_2022_4058_MOESM7_ESM.zip › experiment2/Files_used_for_the_analysis_of_the_manuscript_experiment2/RNA_MODELS_WITH_COVERAGE_experiment_2_passage_15.pdf]
