# Supplementary material for: Evolution of naturally arising SARS-CoV-2 defective interfering particles
Source: Commun Biol. 2022 Oct 27;5:1140. doi: 10.1038/s42003-022-04058-5 (PMC9610340; doi:10.1038/s42003-022-04058-5)
Supplement: Supplementary file 9 — RN-Reporting Summary [file 42003_2022_4058_MOESM9_ESM.pdf]

nature portfolio

Corresponding author(s): T Hobman and J Pelletier

Last updated by author(s): Sep 5, 2022

Reporting Summary

Nature Portfolio wishes to improve the reproducibility of the work that we publish. This form provides structure for consistency and transparency in reporting. For further information on Nature Portfolio policies, see our Editorial Policies and the Editorial Policy Checklist.

Statistics

For all statistical analyses, confirm that the following items are present in the figure legend, table legend, main text, or Methods section.

n/a	Confirmed	
☐	☒	The exact sample size (*n*) for each experimental group/condition, given as a discrete number and unit of measurement
☐	☒	A statement on whether measurements were taken from distinct samples or whether the same sample was measured repeatedly
☐	☒	The statistical test(s) used AND whether they are one- or two-sided<br>*Only common tests should be described solely by name; describe more complex techniques in the Methods section.*
☐	☒	A description of all covariates tested
☒	☐	A description of any assumptions or corrections, such as tests of normality and adjustment for multiple comparisons
☒	☐	A full description of the statistical parameters including central tendency (e.g. means) or other basic estimates (e.g. regression coefficient) AND variation (e.g. standard deviation) or associated estimates of uncertainty (e.g. confidence intervals)
☐	☒	For null hypothesis testing, the test statistic (e.g. *F*, *t*, *r*) with confidence intervals, effect sizes, degrees of freedom and *P* value noted<br>*Give P values as exact values whenever suitable.*
☒	☐	For Bayesian analysis, information on the choice of priors and Markov chain Monte Carlo settings
☒	☐	For hierarchical and complex designs, identification of the appropriate level for tests and full reporting of outcomes
☒	☐	Estimates of effect sizes (e.g. Cohen's *d*, Pearson's *r*), indicating how they were calculated

Our web collection on statistics for biologists contains articles on many of the points above.

Software and code

Policy information about availability of computer code

Data collection	All the Information is provided in the "Supplementary Information".
Data analysis	All the Information is provided in the "Supplementary Information".

For manuscripts utilizing custom algorithms or software that are central to the research but not yet described in published literature, software must be made available to editors and reviewers. We strongly encourage code deposition in a community repository (e.g. GitHub). See the Nature Portfolio guidelines for submitting code & software for further information.

Data

Policy information about availability of data

All manuscripts must include a data availability statement. This statement should provide the following information, where applicable:

- Accession codes, unique identifiers, or web links for publicly available datasets
- A description of any restrictions on data availability
- For clinical datasets or third party data, please ensure that the statement adheres to our policy

All data and materials used in the analyses is available to any researcher for purposes of reproducing or extending the analyses. Uncropped and unedited gels/blots are presented in Supplementary Figure 11. Plasmids can be obtained under a materials transfer agreement (MTA). All other data are available from the corresponding author on reasonable request. Requests should be made to J Pelletier (jerry.pelletier@mcgill.ca).

The fastq files of the raw sequencing data are deposited in the public repository – Sequence Read Archive database under the BioProject ID PRJNA850004 (https://www.ncbi.nlm.nih.gov/sra)48.

Human research participants

Policy information about studies involving human research participants and Sex and Gender in Research.

Reporting on sex and gender	N/A
Population characteristics	N/A
Recruitment	N/A
Ethics oversight	N/A

Note that full information on the approval of the study protocol must also be provided in the manuscript.

Field-specific reporting

Please select the one below that is the best fit for your research. If you are not sure, read the appropriate sections before making your selection.

☒ Life sciences ☐ Behavioural & social sciences ☐ Ecological, evolutionary & environmental sciences

For a reference copy of the document with all sections, see nature.com/documents/nr-reporting-summary-flat.pdf

Life sciences study design

All studies must disclose on these points even when the disclosure is negative.

Sample size	Sample size was not formally predetermined but was based on previous experience
Data exclusions	No data were excluded
Replication	Replications as indicted in the manuscript for each experiment
Randomization	N/A
Blinding	N/A

Reporting for specific materials, systems and methods

We require information from authors about some types of materials, experimental systems and methods used in many studies. Here, indicate whether each material, system or method listed is relevant to your study. If you are not sure if a list item applies to your research, read the appropriate section before selecting a response.

Materials & experimental systems

n/a	Involved in the study	
☐	☒	Antibodies
☐	☒	Eukaryotic cell lines
☒	☐	Palaeontology and archaeology
☒	☐	Animals and other organisms
☒	☐	Clinical data
☒	☐	Dual use research of concern

Methods

n/a	Involved in the study	
☒	☐	ChIP-seq
☒	☐	Flow cytometry
☒	☐	MRI-based neuroimaging

Antibodies

Antibodies used	Antibodies used in this study were: anti-Nsp1 (GeneTex, GTX135612), anti-Nsp10 (Pro-Sci Inc, #9179), anti-FLAG (Sigma-Aldrich, #F1804), anti-RPL7 (Novus Biologicals, #NB100-2268), anti-GAPDH (Abcam, #ab8245), anti-[illegible]-actin (Abcam, #ab8226), anti-eEF2 (CST, #2332), and anti-hnRNPA1 (CST, #8443)
Validation	1) Anti-Nsp1: validated in Nsp1-transfected cells by the manufacturer and in Figs. 3d and 5b of the manuscript. This antibody reacts

Validation

with SARS-CoV-2 virus according to the manufacturer. Cross reactivity with other coronaviruses is not mentioned (https://www.genetex.com/Product/Detail/SARS-CoV-2-COVID-19-nsp1-antibody/GTX135612).
2) Anti-Nsp10: validated by the manufacturer by immunohistochemistry and western blot as well as in Fig. 5b of the manuscript. This antibody's species reactivity is viral according to the manufacturer (https://www.prosci-inc.com/product/sars-cov-2-covid-19-nsp10-antibody-9179/).
3) Anti-FLAG M2: as of the date this form was filled out, this antibody was referenced in 6557 citations according to https://www.citeab.com/antibodies/2304935-f1804-monoclonal-anti-flag-r-m2-antibody-produced-i. This antibody reacts with FLAG tag in all species according to the manufacturer (https://www.sigmaaldrich.com/CA/en/product/sigma/f1804?gclid=Cj0KCQjwwJuVBhCAARIsAOPwGAQYLXQhkjG3CYT5hlc40-sWYOTdnrCWy5Y8qn4G11v_oxFigZgSbskaAjcqEALw_wcB).
4) Anti-RPL7: validated by western blotting in PMID: 23990801 in mouse and PMID: 23125841 in human. This antibody reacts with human, mouse, rat, cow, chicken, and primate according to the manufacturer (https://www.novusbio.com/products/rpl7-antibody_nb100-2268).
5) Anti-GAPDH: referenced in 3486 publications according to the manufacturer and reacts with horse, chicken, guinea pig, hamster, cat, dog, pig, Xenopus laevis, fish, monkey, zebrafish, baboon, and Xenopus tropicalis according to the manufacturer. This antibody was also validated by the manufacturer via western blotting and immunofluorescence (https://www.abcam.com/gapdh-antibody-6c5-loading-control-ab8245.html).
6) Anti-B-actin: reference in 2331 publications according to the manufacturer and reacts with mouse, rat, rabbit, cow, dog, human, Xenopus laevis, fish, and Chinese hamster. This antibody is also predicted to work with sheep, chicken, guinea pig, pig, Drosophila melanogaster, monkey, zebrafish, and Rhesus monkey (https://www.abcam.com/beta-actin-antibody-mabcam-8226-loading-control-ab8226.html).
7) Anti-eEF2: referenced in 237 citations according to the manufacturer and reacts with human, mouse, rabbit, monkey, and Drosophila melanogaster (https://www.cellsignal.com/products/primary-antibodies/eef2-antibody/2332).
8) Anti-hnRNPA1: referenced by 29 citations and validated for western blotting by the manufacturer. This antibody reacts with human, mouse, rabbit, and monkey (https://www.cellsignal.com/products/primary-antibodies/hnrnp-a1-d21h11-rabbit-mab/8443).

Eukaryotic cell lines

Policy information about cell lines and Sex and Gender in Research

Cell line source(s)	Vero E6 and HEK-293T cells were obtained from ATCC. Both cells are of female origin.
Authentication	No formal authentication was carried out.
Mycoplasma contamination	Mycoplasma contamination was routinely tested (~ once a month) and cells were negative.
Commonly misidentified lines (See ICLAC register)	Cell lines used are not listed in the ICLAC database.
